# An AKAP-Lbc-RhoA interaction inhibitor promotes the translocation of aquaporin-2 to the plasma membrane of renal collecting duct principal cells

**DOI:** 10.1371/journal.pone.0191423

**Published:** 2018-01-26

**Authors:** Katharina Schrade, Jessica Tröger, Adeeb Eldahshan, Kerstin Zühlke, Kamal R. Abdul Azeez, Jonathan M. Elkins, Martin Neuenschwander, Andreas Oder, Mohamed Elkewedi, Sarah Jaksch, Karsten Andrae, Jinliang Li, Joao Fernandes, Paul Markus Müller, Stephan Grunwald, Stephen F. Marino, Tanja Vukićević, Jenny Eichhorst, Burkhard Wiesner, Marcus Weber, Michael Kapiloff, Oliver Rocks, Oliver Daumke, Thomas Wieland, Stefan Knapp, Jens Peter von Kries, Enno Klussmann

**Affiliations:** 1 Max Delbrück Center for Molecular Medicine Berlin (MDC), Berlin, Germany; 2 Structural Genomics Consortium, University of Oxford, Oxford, United Kingdom; 3 Leibniz-Forschungsinstitut für Molekulare Pharmakologie (FMP), Berlin, Germany; 4 Zuse Institute Berlin (ZIB), Germany; 5 University of Miami Miller School of Medicine, Miami, United States of America; 6 Institute of Experimental Pharmacology and Toxicology, Medical Faculty Mannheim, Heidelberg University, Heidelberg, Germany; 7 DZHK (German Centre for Cardiovascular Research), partner site Heidelberg/Mannheim, Germany; 8 Institute for Pharmaceutical Chemistry and Buchmann Institute, Goethe University, Frankfurt, Germany; 9 DKTK (German Cancer Center Network), partner site Frankfurt/Main, Germany; 10 DZHK (German Centre for Cardiovascular Research), partner site Berlin, Germany; University of Toledo, UNITED STATES

## Abstract

Stimulation of renal collecting duct principal cells with antidiuretic hormone (arginine-vasopressin, AVP) results in inhibition of the small GTPase RhoA and the enrichment of the water channel aquaporin-2 (AQP2) in the plasma membrane. The membrane insertion facilitates water reabsorption from primary urine and fine-tuning of body water homeostasis. Rho guanine nucleotide exchange factors (GEFs) interact with RhoA, catalyze the exchange of GDP for GTP and thereby activate the GTPase. However, GEFs involved in the control of AQP2 in renal principal cells are unknown. The A-kinase anchoring protein, AKAP-Lbc, possesses GEF activity, specifically activates RhoA, and is expressed in primary renal inner medullary collecting duct principal (IMCD) cells. Through screening of 18,431 small molecules and synthesis of a focused library around one of the hits, we identified an inhibitor of the interaction of AKAP-Lbc and RhoA. This molecule, Scaff10-8, bound to RhoA, inhibited the AKAP-Lbc-mediated RhoA activation but did not interfere with RhoA activation through other GEFs or activities of other members of the Rho family of small GTPases, Rac1 and Cdc42. Scaff10-8 promoted the redistribution of AQP2 from intracellular vesicles to the periphery of IMCD cells. Thus, our data demonstrate an involvement of AKAP-Lbc-mediated RhoA activation in the control of AQP2 trafficking.

## Introduction

Antidiuretic hormone (arginine-vasopressin, AVP) stimulates vasopressin V2 receptors (V2R) on the surface of renal collecting duct principal cells, and thereby triggers the redistribution of the water channel, aquaporin-2 (AQP2) from intracellular vesicles into the plasma membrane. The membrane insertion of AQP2 facilitates water reabsorption from primary urine and fine-tunes body water homeostasis [1–5]. Defects of the mechanism lead to diabetes insipidus (DI), a disease characterized by a massive loss of hypotonic urine and by polydipsia. DI can be caused by mutations in the V2 receptor or AQP2 genes or can be acquired, for example, as a consequence of lithium treatment of bipolar disorders. Molecular mechanisms underlying the AVP-induced redistribution of AQP2 are not well understood and targeted treatments of DI are not available. Thus, elucidating molecular mechanisms controlling AQP2 not only provides insight into the mechanism itself but can also pave the way to new concepts for the therapy of water balance disorders such as DI [6, 7].

On the molecular level, AVP stimulates synthesis of cAMP and activation of protein kinase A (PKA). The subsequent PKA-catalyzed phosphorylation of AQP2 at serine (S)256 is considered the key trigger for its redistribution into the plasma membrane [8–14]. In addition, AVP mediates phosphorylations of S264 and S269, which are associated with a predominant plasma membrane localization of AQP2 [15–20], and the dephosphorylation of S261 [15, 18, 21]. The dephosphorylation is associated with decreased poly-ubiquitination and proteasomal degradation and an enhanced abundance of AQP2, and thus contributes to the increase in water reabsorption from primary urine in response to AVP [22].

GTPases are molecular switches cycling between an inactive, GDP-bound and an active, GTP-bound state. RhoA is a small GTPase of the Rho family, which includes Rho, Cdc42 and Rac isoforms. We have previously shown that active RhoA maintains the F-actin cytoskeleton as a physical barrier hindering AQP2-bearing vesicles from reaching the plasma membrane of principal cells under resting conditions [23]. An elevation of cAMP causes PKA-mediated phosphorylation of RhoA, its increased interaction with Rho guanosine nucleotide dissociation inhibitor (GDI) and thus RhoA inhibition [24]. The inhibition is associated with de-polymerization of F-actin and the redistribution of AQP2 to the plasma membrane [14, 23, 25]. How RhoA in resting cells is maintained in an active form is unclear.

The exchange of GDP for GTP and thus activation of GTPases is catalyzed by guanine nucleotide exchange factors (GEFs). The A-kinase anchoring protein (AKAP)-lymphoid blast crisis (Lbc) is a member of the AKAP family of scaffolding proteins. The common property of AKAPs is their ability to directly bind PKA and tether the AKAP-PKA complex to defined cellular compartments to coordinate cellular signaling events spatially and temporally. We had shown that the tethering of PKA by AKAPs is a prerequisite for the AVP/PKA-induced redistribution of AQP2 to the plasma membrane and that PKA is anchored to AQP2-bearing vesicles by AKAP18δ [12, 13, 26, 27]. AKAP-Lbc is the only AKAP which possesses a GEF activity. It selectively activates RhoA but not the other Rho family members, Rac and Cdc42 [28]. The GEF activity is conferred by a GEF domain comprising a tandem Dbl homology (DH) and pleckstrin homology (PH) domain [29–31]. Two distinct pockets of the DH domain directly interact with RhoA and catalyze the exchange of GDP for GTP. PH domains can control the localization and/or the activity of DH domains [29]. The PH domain of AKAP-Lbc does not affect its activity [31]. Upon an elevation of cAMP in HEK293 cells, AKAP-Lbc-bound PKA phosphorylates AKAP-Lbc at S1565 recruiting a 14-3-3 protein and inhibiting the GEF activity [32]. The rat orthologue of AKAP-Lbc, Rt31, is expressed in primary rat inner medullary collecting duct principal (IMCD) cells, a model for the AVP-induced redistribution of AQP2, and forms a complex with RhoA [5, 33]. However, whether AKAP-Lbc-mediated activation of RhoA plays a role in the control of AQP2, in particular in resting cells, is unknown.

## Materials and methods

### Antibodies

The following antibodies were used for Western blotting: AKAP-Lbc (Abcam; #ab99377; 1:1000), Aquaporin-2 (AQP2) (Santa Cruz; #9882,C17; 1:1000), Hsp90 (Stressgen; #SPA-830; 1:1000), Pan-Cadherin (Abcam; #ab6528; 1:1000), RhoA (Santa Cruz; #sc-418, 1:1000), Cdc42 (Cell Signaling, #2462; 1:1000 for Western blot, 1:250 for PAK-1 pull down), Rac-1 (BD Transduction Laboratories, #610650, 1:1000 for Western blot, 1:250 for PAK-1 pull down), anti-Flag, M2 monoclonal antibody (Sigma, 2 μg/ml), anti-G12QL antibody (rabbit; Gramsch, 1:1000), Peroxidase (POD)-anti-goat IgG (Jackson ImmunoResearch Laboratories; #705-035-147; 1:10,000), POD-anti-mouse IgG (Jackson ImmunoResearch Laboratories; #715-035-151; 1:10,000), POD-F(ab')2-anti-rabbit IgG (Jackson ImmunoResearch Laboratories; #711-036-152; 1:10,000). The following antibodies were used in HTRF assays: GST antibody, coupled to terbium (Donor; Cisbio Bioassays, Codolet, France; #MAb GSS11), His_6_-tag antibody, coupled to XL665 (Acceptor; Cisbio Bioassays; #MAb HIS-1). In immunofluorescence microscopy, AQP2 was detected with antibody H27 (1: 600) [12, 34] and Cy3-anti-Rabbit IgG (Jackson ImmunoResearch Laboratories; #211-165-109; 1:300). Nuclei were detected with 4’, 6-Diamidine-2’-phenylindole dihydrochloride (DAPI; Roche Diagnostics GmbH; Mannheim, Germany; #10236276001; 1:100) and F-actin was visualized using Alexa Fluor 647-Phalloidin (Invitrogen; Darmstadt, Germany); #A22287; 1:30).

### Protein expression and purification

Purified His_6_-RhoA (human RhoA, NP_001655.1, NCBI) and AKAP-Lbc/DHPH (aa 1923–2336 of human AKAP-Lbc; NP_006729.4, NCBI) were used in the GEF assay. Purified GST-RhoA (human RhoA, NP_001655.1) and His_6_-AKAP-Lbc/DHPH (aa 1972–2342 of human AKAP-Lbc; NP_006729.4) were used in the HTRF assay (Fig 2 in S2 in S1 File).

RhoA was expressed as an N-terminal His_6_-tag fusion from a pET30α plasmid (kindly provided by D. Diviani, University of Lausanne), and AKAP-Lbc/DHPH was expressed from a pPal7 plasmid in *E*. *coli* Rosetta (DE3). Bacteria were grown in LB medium and protein expression was induced at an optical density at 600 nm of 0.7–0.9 with 1 mM isopropyl-β-D-thiogalactoside (IPTG). The cells were grown overnight at 16 °C, centrifuged and suspended in protein purification buffer (150 mM NaCl, 10 mM Na_2_HPO_4_, pH 7.4, 5 mM EDTA) and lysed using a fluidiser (Microfluidics; Newton, US). Lysates were centrifuged (40,000xg, 30 min, 4 °C), and the soluble extract was filtered and purified with the Profinia_TM_ affinity chromatography protein purification system (BioRad, Munich, Germany) according to manufacturer’s instructions. His_6_-RhoA was eluted in buffer containing 300 mM KCl, 50 mM KH_2_PO_4_, pH 8.0, 250 mM imidazole and AKAP-Lbc/DHPH in buffer containing 100 mM Na_3_PO_4_, pH 7.2, 100 mM NaCl. Relevant fractions were pooled, proteins concentrated and snap-frozen in liquid nitrogen. The final yield was 20–30 mg protein/l bacterial culture.

GFP-AKAP-Lbc-His_6_ was isolated from HEK293-EBNA cells. 24 h post transfection, the medium volume was doubled and peptone added to a final concentration of 0.5%. After 6 days, cells were lysed and the supernatant concentrated in Amicon tubes (10,000 molecular weight cut-off) to a final volume of 12.5 ml. The soluble extract was filtered and subjected to gel filtration (Superdex200 column). The protein was eluted with gel filtration buffer (50 mM HEPES, pH 7.4, 300 mM NaCl; flow rate 1 ml/min). Relevant fractions were pooled, the protein concentrated and snap-frozen in liquid nitrogen.

### Guanine nucleotide exchange (GEF) assay

His_6_-RhoA and AKAP-Lbc/DHPH (2 μM each) were incubated with the fluorescently labeled nucleotide analogue 2’/3’-*O*-(*N*-Methyl-anthraniloyl)-GTP (mant-GTP) in 1 μM concentration in GEF buffer (40mM Tris, pH 7.5; 100 mM NaCl; 20 mM MgCl_2_; 100 μg/ml bovine serum albumine (BSA)). Fluorescence was recorded for 15 min in a 384-well plate in a microplate reader (Tecan, Safire; Durham, US) at 440 nm (360 nm excitation wavelength).

### High-throughput screening

**Fig 1:**
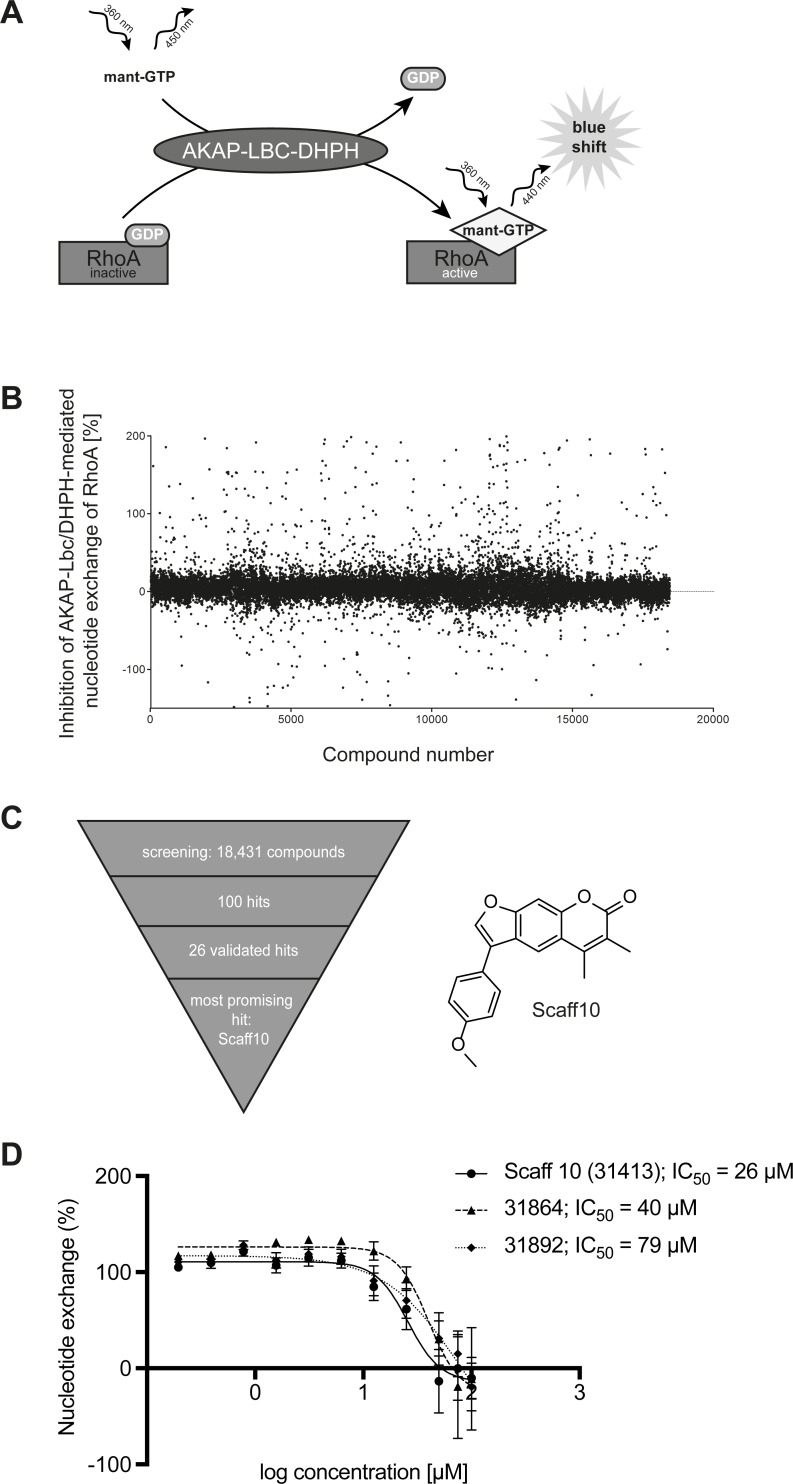
Identification of a small molecule inhibitor of the AKAP-Lbc-mediated activation of RhoA. (A) Principle of the nucleotide exchange assay for the screening. The DHPH domain of the RhoA-specific guanine nucleotide exchange factor (GEF) AKAP-Lbc and RhoA were generated as recombinant proteins. The DHPH domain-catalyzed exchange of GDP for the fluorescent mant-GTP was monitored at the emission wavelength of RhoA-bound mant-GTP at 440 nm. (B) The fluorescence signal intensity changes were determined for all samples including positive and negative controls and percent inhibitions calculated based on the controls. The screening data quality was evaluated as distribution of percent inhibition of all tested compounds and by calculating the Z’-factors for each plate, which were found to be 0.51 on average. (C) Using the GEF assay from (A), 18,431 small molecules were screened for inhibitors of the nucleotide exchange; 100 hits were identified of which 26 candidates were validated. The most promising hit was the compound Scaff10 (structure indicated). (D) Nucleotide exchange assay with Scaff10 and two further hits (for details see S1 Table). IC_50_ values for the inhibition are indicated.

The ChemBioNet [35] and CBB2 compound libraries at the Leibniz-Forschungsinstitut für Molekulare Pharmakologie (FMP), in total 18,431 compounds, were screened using the nucleotide exchange assay depicted in Fig 1A. All library compounds are accessible at http://www.fmp-berlin.de, and further information on every compound is available at http://www.ncbi.nlm.nih.gov/pccompound. For all dispensing and washing steps an EL406 washer/dispenser combination (BioTek Instruments, Bad Friedrichshall, Germany) was used. The screen was performed in a 384-well plate format. Obtained data were automatically analyzed as described [35–37]. *Z score* describes the distance of a sample signal to the mean of all other samples on the plate in units of standard deviation. Percent inhibition describes the relative strength of the signal compared to means of the control samples on the plate (where samples in column 23 received only DMSO and were set to 0% inhibition, and samples in column 24 did not receive GEF and were set to -100%). Samples with a Z score < -3 were considered as potential inhibitors (655 samples). For quality control purposes, Z’-factors describing the effective signal window were calculated for each plate based on the control samples. The Z’-factor was on average 0.51, indicating reliable screening conditions. Potential inhibitors were subjected to a second round of GEF assay for confirming concentration-dependent effects. Compound identity and purity of samples used in this secondary screen were validated by mass spectrometric analysis.

### Synthesis of compounds

Starting materials and solvents for synthesis were purchased from Sigma-Aldrich Chemie GmbH (Munich, Germany), Otava Chemicals (Ontario, CA), Fluka (Munich, Germany), Acros (Geel, Belgium), and ALFA Aesar (Karlsruhe, Germany). For high-performance liquid chromatography (HPLC) and liquid chromatography-mass spectrometry (LC/MS) measurements, acetonitrile (ACN) and methanol (HPLC grade) from J. T. Baker (Center Valley, US) and purified water (Milli-Q-Plus from Millipore) were used. Deuterated solvents for nuclear magnetic resonance (NMR) spectroscopy were purchased from Deutero GmbH (Kastellaun, Germany).

**Fig 2:**
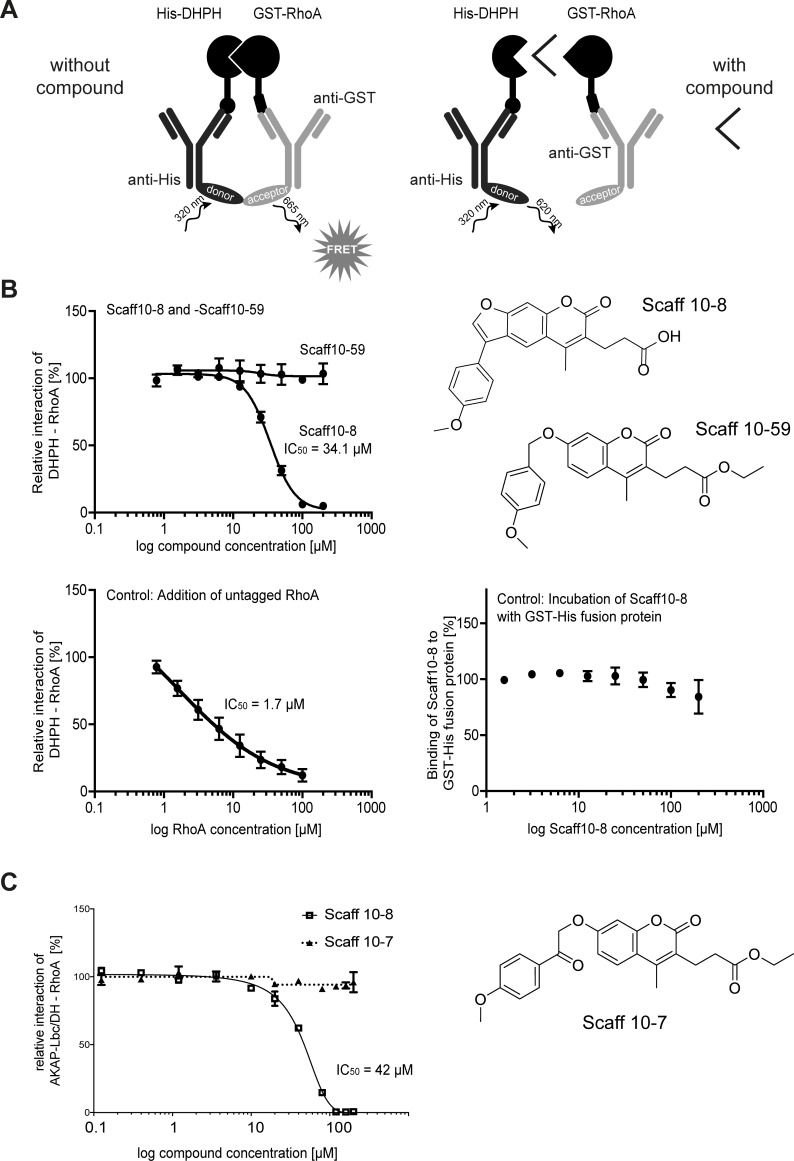
The small molecule Scaff10-8 inhibits the interaction of the DH domain of AKAP-Lbc and RhoA. (A). Principle of homogenous time-resolved fluorescence (HTRF) assay. Recombinant His-AKAP-Lbc/DHPH and GST-RhoA were generated. The tags are recognized by specific antibodies coupled to fluorescent dyes. When the two proteins interact, donor and acceptor dyes of the antibodies are in close proximity, energy transfer occurs upon excitation at 320 nm and a FRET signal is detected (665 nm). If a compound disrupts the interaction of the DHPH domain and RhoA, the proximity of the fluorescent moieties remains insufficient for a FRET signal to occur. (B) In the HTRF from (A), Scaff10-8 inhibited the interaction of AKAP-Lbc/DHPH and RhoA with an IC_50_ = 34.1 μM whereas Scaff10-59 did not (example of a negative Scaff10 derivative). As expected, excess untagged Rho decreases the binding between GST-RhoA and His-AKAP-Lbc/DHPH in a dose-dependent manner (lower left panel). Scaff10-8 does not interact with a His-GST fusion protein, excluding any interference of the tags or antibodies used in this assay with Scaff10-8. n = 3–15 independent experiments in duplicate; IC_50_ values are indicated. (C) The influence of Scaff10-8 on the interaction of RhoA with the recombinant DH domain of AKAP-Lbc in the absence of the PH domain was determined by Alpha screen. Scaff10-7/59 was included as negative controls as they did not interfere with interaction of the DHPH domain and RhoA in the HTRF shown in Fig 2. n = 3–15 independent experiments in duplicate. The IC_50_ value is indicated.

The synthesis of all compounds is described in detail in the Supporting Information. The chemical synthesis of Scaff10 derivatives followed the scheme shown below in Fig 2. Under neutral reaction conditions, acetophenones (1) were transformed into the corresponding α-iodoketones (2) by addition of iodine in methanol (a). Addition of Na_2_SO_3_-solution (b) reduced excessive iodine to iodide. 7-Hydroxycoumarin derivatives were synthesised based on the Pechmann condensation: in an initial reaction step, resorcinol (3) and diethyl-2-acetylglutarat (4) reacted to the corresponding phenolester in ethanolic HCl (c). Following a Michael addition, the enol form of the acid catalysed keto-enol tautomerisation was nucleophilically attacked by the aromatic system. Finally, rearomatisation and acid-induced condensation yielded the product of the 7-hydroxycoumarin derivative ethyl 3-(7-hydroxy-4-methyl-2-oxo-chromen-3-yl)propanoate (5a). In the presence of potash in excess in acetone (d), 7-hydroxycoumarins (5) reacted with α-haloketones (2) in a Williamson ether synthesis at 55°C overnight to the corresponding substituted 2-oxoethers (6) [38], representing class 6 Scaff10 derivatives. After ester saponification to the carboxylate by adding 1 M sodium hydroxide, Scaff10 derivatives of class 7 were formed. Reaction conditions ranged from 55–95°C and from 0.3-16 hours (e). Cyclisation of Scaff10 derivatives of class 7 to the corresponding furocoumarin derivatives (8) (Scaff10 derivative class 8) was carried out under further heating in NaOH solution (60–110°C) for 0.75–10 h (f). The reaction conditions required for the synthesis of Scaff10 derivatives of class 7 and 8 are indicated.

Scaff10 derivatives were classified into uncondensed (6, 7) and furan (8) derivatives (opening of the furan in position *g* of the coumarin structure). Class 6 and 7 differ in position 3 of the coumarin structure: class 6 is substituted with either a propionic acidester or with a methyl group whereas class 7 exhibits a carboxylic group at a C1-C5 spacer between coumarin and carboxylic acid at this position.

Solubility of Scaff10 derivatives was assessed nephelometrically. Scaff10 derivatives are moderately to poorly soluble in aqueous solutions due to their large number of aromatic rings having lipophilic character. Details about NMR, LC/MS and HPLC characterization steps are given in the Supplemental Information.

### Homogeneous time-resolved fluorescence (HTRF) assay

The assay was essentially carried out as described [39]. Scaff10 derivatives were added to His_6_-AKAP-Lbc/DHPH protein (250 nM final concentration) in HTRF buffer (PBS; 4 mM MgCl_2_; 0.05% Tween 20) in 384 well plates (Proxiplate-384 Plus, white, #6008289; Perkin Elmer LAS, Rodgau, Germany). Concentrations of Scaff10 derivatives ranged between 1.56–200 μM (2% DMSO final concentration); HTRF buffer with 2% DMSO served as a control. GST-RhoA (30 nM final concentration) in HTRF buffer and anti-GST antibody coupled to terbium (donor) and anti-His_6_-tag antibody coupled to XL665 (acceptor) were added to all wells (final antibody concentration: 2 μg/ml). After 1 h incubation at room temperature in the dark, FRET signals were recorded at 620 and 665 nm using a Genios Pro plate reader (Tecan Austria GmbH, Grödig, AT) and the ratios of absorbance of 650 nm to 620 nm were calculated. Wells containing no compound were set to 100% interaction of AKAP-Lbc/DHPH with RhoA, wells containing GST-RhoA only (without His_6_-AKAP-Lbc/DHPH) were set to 0% interaction. To exclude any interference of the tags and/or antibodies with Scaff10 derivatives, a His_6_-GST fusion protein replaced the two tagged proteins in a control assay. Wells containing His_6_-GST fusion protein were set to 100% interaction of His_6_-GST, wells containing no fusion protein were set to 0% interaction.

### Alpha screen assay

Assays were performed according to the manufacturer’s protocol (PerkinElmer) with minor modifications. All reagents were diluted in buffer containing 25 mM Hepes, pH 7.4, 100 mM NaCl, 0.1% bovine serum albumin, 0.05% CHAPS and 50 μM GDP and allowed to equilibrate to room temperature before addition to plates. The assays were run in 20 μL volumes in low-volume 384-well plates (ProxiPlate-384 Plus, PerkinElmer, USA) at RT. The proteins used were N-terminally His6-tagged AKAP-Lbc-DH domain and C-terminally biotinylated RhoA. To determine ideal assay concentrations of the two proteins, 4 μL volumes of RhoA were incubated with 4 μL His-AKAP-Lbc-DH domain (0–16 μM of each; final assay concentrations: 0–3.2 μM) in 4 μL buffer for 30 min at RT in foil-sealed plates. 8 μL of assay buffer containing donor and acceptor beads at 1:400 dilution was then added, the plate was incubated in the dark for 60 min and then read on a PheraStar FS plate reader (BMG Labtech, Germany) using an AlphaScreen^TM^ 680 excitation/570 emission filter set.

For compound IC_50_ measurement, a dilution series of the compound dissolved in DMSO in 0.4 μL total volume (final assay concentrations 200 μM to 0.125 μM) were made into the assay plate using an Echo dispenser (Labcyte, California, USA). 11.6 μL of a solution containing 100 nM RhoA and 200 nM AKAP13 in assay buffer was added and incubated with the compounds for 30 min at RT in foil-sealed plates. 8 μL of assay buffer containing donor and acceptor beads at 1:400 dilution was added. The plate was incubated in the dark for 60 min and then read on a PheraStar FS plate reader. Alternatively, for counter-screening of the compounds, 11.6 μL of 75 nM biotinylated and His6-tagged linker peptide (PerkinElmer) was added instead of the protein solution.

### Microscale thermophoresis (MST)

**Fig 3:**
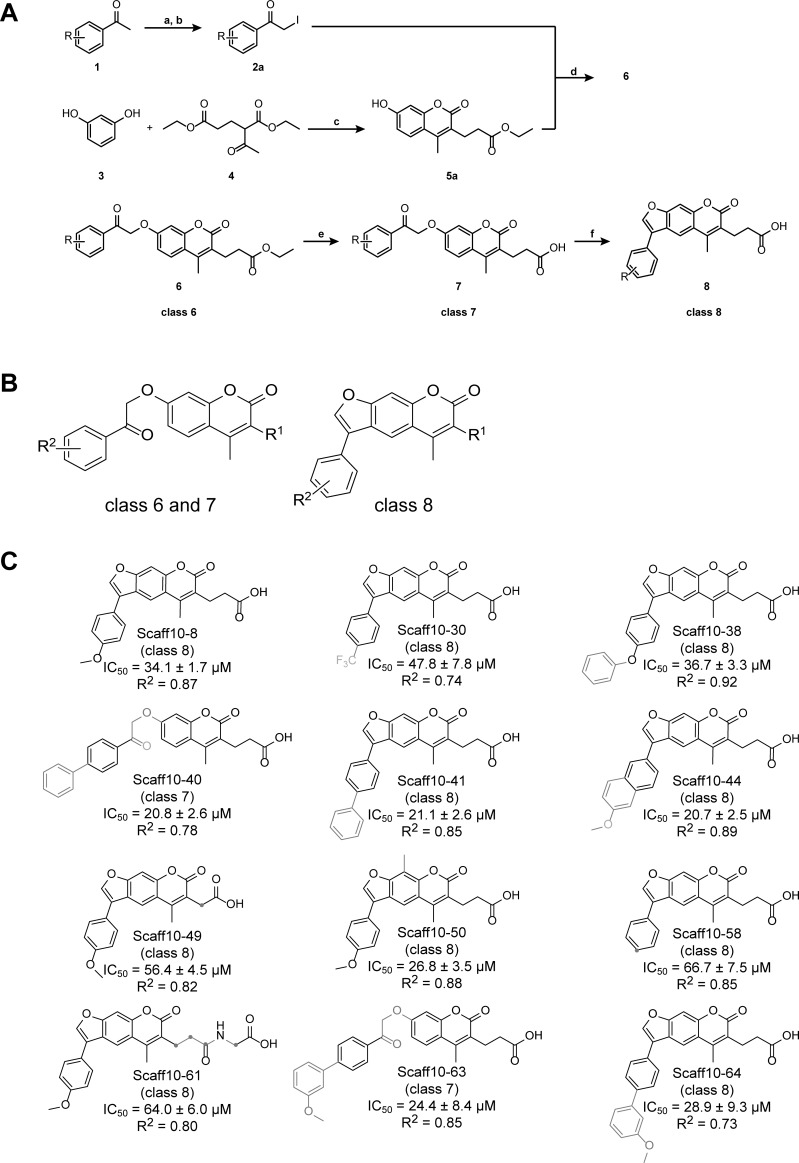
Scaff10 derivatives. (A) Overview of the synthesis of Scaff10 derivatives. By addition of iodine in methanol (a), acetophenone derivatives (1) reacted with the corresponding α-iodoketones (2a), removal of excessive iodine by addition of Na_2_SO_3_-solution (b). Resorcinol (3) and diethyl-2-acetylglutarat (4) were transformed into the 7-hydroxycoumarin derivative ethyl 3-(7-hydroxy-4-methyl-2-oxo-chromen-3-yl)propanoate (5a) in ethanolic HCl (c). In the presence of excess potash, 5 reacted with α-haloketone derivatives (2) in a Williamson ether synthesis at 55°C in acetone (d) to 6. Saponification to 7 was carried out in 1 M NaOH at 55–95°C and from 0.3–16 h (e). Final cyclization of ketones to furocoumarin derivatives (8) was carried out upon further heating in NaOH solution (60–110°C) for various times (0.75–10 h) (f). R is indicated in Table 1 and (C). (B) Compounds of the generalized structures 6–8 from (A) were allocated into classes 6–8, respectively. (C) Structures of Scaff10 derivatives, which inhibit the AKAP-Lbc/DHPH-RhoA interaction in the homogenous time-resolved fluorescence (HTRF) assay depicted in Fig 3A and B. IC_50_ values (μM ± SEM) were obtained from n = 3–15 independent HTRF experiments carried out in duplicate (S3 Table). Structural differences compared to Scaff10-8 are shown in grey. R^2^ indicates the coefficient of determination.

The final concentration of GFP-AKAP-Lbc/DHPH was set to 150 nM, 95% LED and 80% MST laser power. The final concentration of Red-NHS-labelled RhoA (647-RhoA; labelling performed according to manufacturer’s instructions; Monolith_TM_ Protein Labeling Kit RED-NHS (Amine Reactive; #MO-L001; NanoTemper Technologies GmbH, Munich, Germany) was set to 100 nM, 20% LED and 20% MST laser power. Thereby, the fluorescence intensity measured for each fluorescent protein was comparable (100–150 fluorescence units). Final concentrations of Scaff10 derivatives ranged from 12.21 nM to 200 μM, control wells received 2% DMSO. For controls, His_6_-AKAP-Lbc/DHPH and unlabeled RhoA were added in concentrations of 17.70 nM– 290 μM and 36.01 nM– 590 μM, respectively, in MST buffer (without DMSO). The samples were incubated in the dark for 1 h incubation at room temperature, and measured with 30 sec laser-on and 5 sec laser-off time at a Monolith NT.115 device (NanoTemper). Measured fluorescence (F) was normalised (F_hot_/F_cold_ = F_steady state_/F_initial state_, Fig 3). Values of thermophoresis + T-jump were related to average of each measurement row (16 capillaries). Final values are given as ‰.

### Isothermal titration calorimetry (ITC)

ITC measurements were essentially carried out as previously described [40]. The stabilizing RhoA mutant F25N [41] was purified as a GST-RhoA fusion in its GDP-bound form (final gel filtration purification step in 20 mM HEPES, pH 7.5, 150 mM NaCl, 2 mM DTT, 50 μM GDP). In order to exchange GDP against GTPγS, GST-RhoA loaded with GDP was incubated for 20 min in ITC nucleotide exchange buffer (50 mM HEPES, pH 7.5, 20 mM EDTA) at 25°C followed by three dilution and concentration steps in Amicon tubes (10,000 MWCO) using ITC dialysis buffer (50 mM HEPES, pH 7.4, 150 mM NaCl, 5 mM MgCl2, 2 mM β-mercaptoethanol). The nucleotide-loading state was confirmed by HPLC. All proteins used in ITC measurements were dialyzed overnight against ITC dialysis buffer. Protein concentrations were determined with NanoDrop ND-1000. GDP or GTPγS (final concentration 2 mM) and DMSO (2%) were added to the proteins prior to the ITC measurements. Scaff10-8 was dissolved in the same buffer (including 2% DMSO and 2 mM GDP or GTPγS). ITC measurements were performed on a VP-ITC (GE Healthcare, München, DE) at 15°C. Titrations were conducted using 14.8 μM Scaff10-8 in the cell and 235 μM (GST control), 222 μM (GST-RhoA, GDP-bound) or 210 μM (GST-RhoA, GTPγS-bound) in the syringe. The cell was stirred at 351 rpm and a total of 27 x 10 μl injections were added to the cell, separated by 240 s of equilibration. The data were fitted using the Microcal Origin software (version 7.0) and a one-site binding model. We consistently observed a binding number close to N = 0.5 (RhoA/Scaff10-8). This observation may be due to the binding of two molecules of Scaff10-8 per one molecule of GST-RhoA for both the GDP-bound and GTPγS-bound form of GST-RhoA, or due to some inaccuracy in the determination of protein or compound concentrations. The data were thus fitted using a fixed binding number of N = 0.5.

### MTS cytotoxicity assay

The potential cytotoxicity of Scaff10-8 on primary IMCD and several permanent cell lines (MCF7, H9C2, HEK293, MCD4) was assessed using the tetrazolium compound-based CellTiter 96 AQueous One Solution Cell Proliferation (3-(4,5-dimethylthiazol-2-yl)-5-(3-carboxymethoxyphenyl)-2-(4-sulfophenyl)-2H-tetrazolium) (MTS) assay according to the instructions of the manufacturer. In brief, the cells were seeded into 96 well plates (5,000 cells per well) and cultured in their appropriate medium Scaff10-8 (100 μM, 30 μm and 3 μm, 0.3% final DMSO concentration) were added, DMSO (0.3%) was included as a control. Staurosporine (1 μM) was included as a positive control. All conditions were tested in quadruplicate. At the indicated time points, the MTS reagent was added. Absorption was determined 4 h after addition of MTS reagent at 450 nm wavelength. The MST reagent absorbance at 450 nm is proportional to the amount of living cells. A blank measurement determined the medium absorption (background). Values were normalized to untreated cells after 24 h for each cell line.

### Luciferase assay

Luciferase reporter gene assays were performed with the Dual Luciferase Reporter Assay System (Promega) according to manufactures protocol and as previously reported [42]. In brief, HEK293 cells were seeded and co-transfected with the indicated plasmids together with pSRE.L reporter plasmid encoding firefly luciferase (kindly provided from Dr J. Mao and Dr. D. Wu, Rochester, NY) and pRL-TK control vector (Promega, Mannheim, Germany) encoding renilla luciferase. The transfected cells were further cultured in DMEM with 0.5% FCS for 24 h, lysed with passive lysis buffer (Promega). Luciferase activities was measured an Envision Instrument (Perkin Elmer, Rodgau, Germany) in white 96-well plates. N-terminally flag-tagged LARG and PDZ-RhoGEF are encoded in the vector pCMV2b, kindly provided by Stefan Offermanns (Max-Planck-Institut für Herz- und Lungenforschung, Bad Nauheim, Germany; [43]). Western blotting for the detection of Flag was carried out with primary anti-Flag, M2 monoclonal antibody (Sigma, 2 μg/ml). G12QL was in pCDNA3, purchased from cDNA.org. In Western blots, the protein was detected with primary polyclonal antibody (rabbit; Gramsch, 1:1000). The vector pEGFP-N1 encoding GFP-tagged AKAP-Lbc was kindly provided by Dr. J. Scott (University of Washington, Seattle, USA; [28]). AKAP-Lbc was detected with a specific anti-AKAP-Lbc antibody (Abcam; #ab99377; 1:1000). For transfection in the luciferase assay, 30 ng of each AKAP-Lbc, LARG or PDZ-RhoGEF, and 10 ng of G12QL were transfected.

### Detection of AQP2 and F-actin in primary IMCD cells

Primary rat renal inner medullary collecting duct (IMCD) cells were obtained and cultured as previously described [34, 44]. AQP2 was detected with specific rabbit (H27 antibody) and secondary Cy3-coupled antibodies, and F-actin was visualized with Alexa Fluor 647-Phalloidin by laser scanning microscopy (LSM780; Zeiss Jena, Germany) as described [23].

### Biotinylation of cell surface proteins

The assay was performed according to Bogum et al. [44]. IMCD cells were treated with Scaff10-8 at a concentration of 30 μM for 1 or 24 h (0.3% DMSO final concentration). DMSO (0.3%) was used as a control for the 24 h of incubation. Cells were stimulated with forskolin (30 min, 30 μM). Cells were washed twice with ice-cold PBS and incubated with biotinylation buffer (500 μg Biotin per well; 10 mM triethanolamine; 150 mM NaCl; 1 mM MgCl_2_; 0.1 mM CaCl_2_; pH 8.0) for 60 min at 4°C. After two washing steps with biotinylation buffer, cells were incubated for 10 min with quenching buffer (50 mM NH_4_Cl in PBS) at 4°C. Lysates were prepared using biotinylation lysis buffer 1 (SLB; 0.5% Triton X-100; 0.2% BSA; Complete mini EDTA-free). Lysates were sonicated and incubated for 20 min at 37°C. Cell debris was removed by centrifugation (30 min, 15,000x g, 4°C). Lysates were incubated with 80 μl streptavidin agarose beads at 4°C overnight. Beads were washed three times with biotinylation lysis buffer 1 and 2 (SLB; 0.5% Triton X-100; Complete mini EDTA-free). Proteins were eluted using sample buffer and analyzed by Western blotting.

### Western blotting and determination of RhoA, Cdc42 and Rac1 activities

Western blotting for the detection of RhoA and Hsp90 and a Rhotekin pull down for the detection of active, GTP-bound RhoA were adapted from a procedure previously described by us [23, 45]. In brief, IMCD cells were cultured as described [34], DBcAMP and nystatin were removed on day twelve after seeding by exchange of medium for medium without the compounds. On the following day, Scaff10-8 was added to a final concentration of 30 μM or, as a control, the solvent DMSO (0.3%) for 1 h. Then cells were treated with forskolin (30 μM) for an additional 30 min, and subsequently incubated at 4°C in ice-cold Rhotekin buffer (50 mM Tris, pH 7.2; 1% (w/v) Triton X-100; 0.5% sodium deoxycholate; 500 mM NaCl; 10 mm MgCl_2_; PhosSTOP EASY (PhosSTOP EASY pack, Roche Diagnostics, Mannheim, DE; #REF04906837001), Complete mini EDTA-free (Complete mini EDTA-free, Roche Diagnostics, Mannheim, DE; #REF0693159001), for 10 min and lysed. Lysates (300–400 μg protein) were incubated with 300 μl of Rhotekin beads as previously described [24] Proteins were eluted from beads with Laemmli buffer and analyzed by Western blotting. Active RhoA was related to RhoA in the input fraction and the ratio to the loading control, Hsp90.

The (p21) binding domain (PBD) of p21 activated kinase 1 protein (PAK-1) spans amino acids 67–150 of PAK-1 and binds specifically to the GTP-bound Rac-1 and Cdc42. The PBD was prepared as described [46] and used for selective precipitation of the activated GTPases from IMCD cells. The PBD was expressed as a GST-fusion protein in DH5α E-coli cells, the cells were lysed using a French press, and the fusion protein was purified with Glutathione Sepharose 4 Fast Flow GST-tagged protein purification resin kit according to the manufacturer’s instructions (GE Healthcare; #17-5132-01). The PAK-1-PBD pull down was carried out with 400–500 μg protein from IMCD cell lysates. Proteins were eluted from the beads by boiling with Laemmli sample buffer, and analyzed by Western blotting. The membranes were first probed for Cdc42, and the GTP-bound active fraction of the protein was related to total Cdc42, and to the loading control GAPDH. The membranes were re-probed for Rac-1 accordingly to determine the active GTP-bound Rac-1 fraction.

### Statistics

Statistical analyses were carried out using GraphPad Prism 5.0 software and a one-way ANOVA combined with a Bonferroni post hoc comparison test in order to evaluate statistical significance.

## Results

### A novel small molecule inhibits the AKAP-Lbc-induced activation of RhoA and the interaction of the two proteins

We aimed to develop a small molecule that prevents RhoA activation by interfering with the interaction of AKAP-Lbc and RhoA. We initiated the search for inhibitors by screening of a library of 18,431 small molecules (ChemBioNet library [35]) using a guanine nucleotide exchange assay for RhoA activation after addition of the DHPH domain of AKAP-Lbc (AKAP-Lbc/DHPH; Fig 1A and 1B). The screen identified 655 inhibitors (S1 Table in S1 File); 100 of the primary hits and 252 additional structurally related compounds from the library (1, 10 and 100 μM) were tested for dosage-dependent activity using the same assay. We were able to determine IC_50_ values for 26 compounds. 23 initial hits were autofluorescent and thus not further considered (S2 Table in S1 File). Compound 31413 (Scaff10; Fig 1C) inhibited the AKAP-Lbc/DHPH-induced RhoA activation with the lowest IC_50_ values in the primary screen and secondary assays (IC_50_ = 26 μM; Fig 1D).

Initial cell-based experiments were carried out to gain insight into the specificity of Scaff10. We expressed AKAP-Lbc and various other RhoGEFs in HEK293 cells: ARHGEF25 (p63RhoGEF), Kalirin (Duet), Trio, RhoGEF, RhoGEF2, ARHGEF12 (LARG), MCF2L (Ost1), and ARHGEF11 (PDZRhoGEF). Scaff10 selectively inhibited the AKAP-Lbc-mediated activation of RhoA (Fig A in S1 in S1 File). AKAP-Lbc is selectively activated by the G protein G_12_ [28]. A constitutively active version of G_12_ (G_12_QL) increased the level of active RhoA in HEK293 cells transiently expressing AKAP-Lbc. While Scaff10 abolished this activation, it did not affect RhoA activation through constitutively active G_q_ (G_q_RC; Fig B in S1 in S1 File). The data indicated specific interference of Scaff10 with AKAP-Lbc signaling. Based on these observations, Scaff10 served as a starting point for the development of a small molecule inhibitor of the AKAP-Lbc-RhoA interaction. For this, a focused library around Scaff10 comprising 54 compounds was synthesized and the structure-activity relationship elucidated.

### The Structure-Activity Relationship (SAR) of Scaff10 derivatives

SARs indicate the chemical moieties of a small molecule that determine its inhibitory effects. Our focused library of 54 compounds around Scaff10 (Table 1, S3 Table in S1 File, Fig 3) and our homogeneous time-resolved fluorescence (HTRF) assay (Fig 2A) was used to obtain insight into the structure activity relationship of the identified 4-methoxyphenylfuorochromenone Scaff10 (Figs 1C and 2B; S3 Table). Twelve out of the 55 compounds inhibited the AKAP-Lbc/DHPH–RhoA interaction in our HTRF assay with IC_50_ values ranging from 20–65 μM (Fig 2B, S3 Table in S1 File; recombinant proteins are shown in Fig 2 in S2 in S1 File). Scaff10-8 inhibited the interaction with an IC_50_ = 34.1 μM (Fig 2B). Alpha screen assays demonstrated the inhibitory effect of Scaff10-8 on the interaction of the DH domain of AKAP-Lbc and RhoA in the absence of the PH domain (IC_50_ = 42 μM; Fig 2C).

**Table 1:** List of substituents R^1^ and R^2^ of Scaff10 derivatives.

Scaff10 derivative	class	R^1^	R^2^	IC_50_ ± SEM μM	log P
Scaff10	8	-CH_3_	4-methoxyphenyl-	-	4.60
Scaff10-6	7	-C_2_H_4_COOH	4-methoxyphenylethanone-	-	3.65
Scaff10-7	6	-C_2_H_4_COOC_2_H_5_	4-methoxyphenylethanone	-	4.22
Scaff10-8	8	-C_2_H_4_COOH	4-methoxyphenyl-	34.1 ± 1.7	4.36
Scaff10-26	8	-C_2_H_4_CON(CH_3_)_2_	4-methoxyphenyl-	-	4.02
Scaff10-30	8	-C_2_H_4_COOH	4-(trifluoromethyl)phenyl-	47.8 ± 7.8	5.19
Scaff10-32	8	-C_2_H_4_COOH	4-pyridyl-	-	3.10
Scaff10-35	7	-C_2_H_4_COOH	4-(dimethylamino)phenylethanone-	-	3.83
Scaff10-38	8	-C_2_H_4_COOH	4-phenoxyphenyl-	36.7 ± 3.3	5.81
Scaff10-40	7	-C_2_H_4_COOH	4-biphenyl-	20.8 ± 2.6	5.19
Scaff10-41	8	-C_2_H_4_COOH	4-biphenyl-	29.1 ± 2.6	5.77
Scaff10-43	7	-C_2_H_4_COOH	6-methoxynaphthyl-ethanone-	-	4.56
Scaff10-44	8	-C_2_H_4_COOH	6-methoxy-2-naphthyl-	20.7 ± 2.5	5.27
Scaff10-46	8	-CH_3_	4-biphenyl-	-	6.13
Scaff10-47	8	-CH_3_	6-methoxy-2-naphthyl-	-	5.51
Scaff10-48	8	-C_2_H_4_COOCH_3_	4-methoxyphenyl-	-	4.59
Scaff10-49	8	-CH_2_COOH	4-methoxyphenyl-	56.4 ± 4.5	3.90
Scaff10-51	8	-C_2_H_4_CONHOH	4-methoxyphenyl-	-	3.78
Scaff10-58	8	-C_2_H_4_COOH	Phenyl-	66.7 ± 7.5	4.38
Scaff10-59	6	-C_2_H_4_COOC_2_H_5_	4-methoxyphenyl-	-	4.55
Scaff10-61	8	-C_2_H_4_CONHCH_2_COOH	4-methoxyphenyl-	64.0 ± 6.0	3.46
Scaff10-63	7	-C_2_H_4_COOH	3'-methoxy-1,1'-biphenyl-4-ethanone-	24.4 ± 8.4	5.17
Scaff10-64	8	-C_2_H_4_COOH	4-(3‘-methoxyphenyl)phenyl-	28.9 ± 9.3	5.75

Scaff10 derivatives were synthesized and the active compounds, i.e. inhibitors of the interaction of the DHPH domain of AKAP-Lbc and RhoA, were allocated into classes 6–8 (Fig 3A and 3B). IC_50_ values were obtained in HTRF assays (see Fig 2 and S3 Table). log P values were calculated.

During synthesis, classes 6–8 of Scaff10 derivatives were defined (Fig 3A). All inhibitors of the AKAP-Lbc/DHPH-RhoA interaction were from classes 7 and 8 (Fig 3B and 3C). The carboxylic group at a C2-spacer at R^1^ seemed to be required for the inhibitory effect (R^1^ and R^2^ are shown in Table 1). R^1^ in class 6 compounds was a propionic ester, which rendered the molecules inactive (no inhibitory effect of compounds in class 6, e.g. Scaff10, Scaff10-46 and Scaff10-47). The polar character as electron pair donor of the carboxylate in classes 7 and 8 was not the reason of the inhibitory effect, as a replacement against a dimethylamide (Scaff10-26) or a methylester (Scaff10-48) resulted in a complete loss of the inhibitory effect. Thus, the negative charge of the carboxylate seems highly relevant for the inhibitory effect. For further analysis of the impact of the negative charge, the carboxylic acid was substituted with hydroxamic acid. Since the quantity of negatively charged molecules depends on the pK_a_ value of the chemical function, the differences in the pK_a_ values should result in an altered inhibitory effect (estimated pK_a_ value for Scaff10-8 (carboxylic acid) ~ 5; cinnamic acid (pK_a_ = 4.44), propionic acid (pK_a_ = 4.88); estimated pK_a_ value for Scaff10-51 (hydroxamic acid) ~ 9, compare to acetohydroxamic acid (pK_a_ = 8.7)). The increase of the pK_a_ value of Scaff10-51 results in an almost complete loss of activity. In addition, the length of the spacer between carboxylic acid and coumarin at R^1^ seemed relevant: shortening the spacer from two to one carbon atoms increased the IC_50_ value to 56 ± 5 μM (Scaff10-49), extension to 5 atoms *via* an amide bond at R^1^ also increased the IC_50_ value (64 ± 6 μM for Scaff10-61). Hence an optimal length of the spacers between carboxylic acid and coumarin at R^1^ was between 2 and 4 atoms.

Remarkably, most of the uncondensed derivatives of class 7 were inactive, except Scaff10-40 (IC_50_ = 21 ± 3 μM) and Scaff10-63 (IC_50_ = 24 ± 8 μM). The R^2^ substituent of the two compounds is highly flexible, resulting in many conformers and a consequently increased probability of a bioactive conformation fitting into the targeted binding pocket. The steric fixation of molecules of class 8 was beneficial in most cases. As R^2^ substituents, potentially charged groups such as the tertiary amine group at the *para*-position of the substitution of the phenyl ring (Scaff10-35), a trifluoromethyl substituent (Scaff10-30) or a 4-pyridyl substituent (Scaff10-32) at the phenyl ring leads to reduced (trifluoromethyl substituent) or complete loss of activity (tertiary amine, 4-pyridyl). A methoxy substituent in *para*-position of the phenyl ring at R^2^ enhanced the inhibitory effect (Scaff10-8 and Scaff10-58). In addition, an extension of the R^2^ substituent by aromatic moieties, e.g. Scaff10-38, Scaff10-41, Scaff10-44 and Scaff10-64, was beneficial or did not change the inhibitory potency. Rather bulky substituents at this position, such as a naphthyl substituent, were tolerated only for condensed compounds (Scaff10-44).

In conclusion, inhibition of the AKAP-Lbc/DHPH-RhoA interaction by Scaff10 derivatives required a negatively charged group like a carboxylic acid at a C2 to C4 spacer at position 3 of the coumarin indicating interaction with positively charged amino acids such as lysine, arginine or histidine in the binding pocket. In addition, a long, lipophilic and flexible moiety at R^2^ with a methoxy substituent at the *para*-position adds to the inhibitory effect; condensation to furan mostly improves the inhibitory effect.

### Scaff10-8 binds to GDP- and GTP-bound RhoA

**Fig 4:**
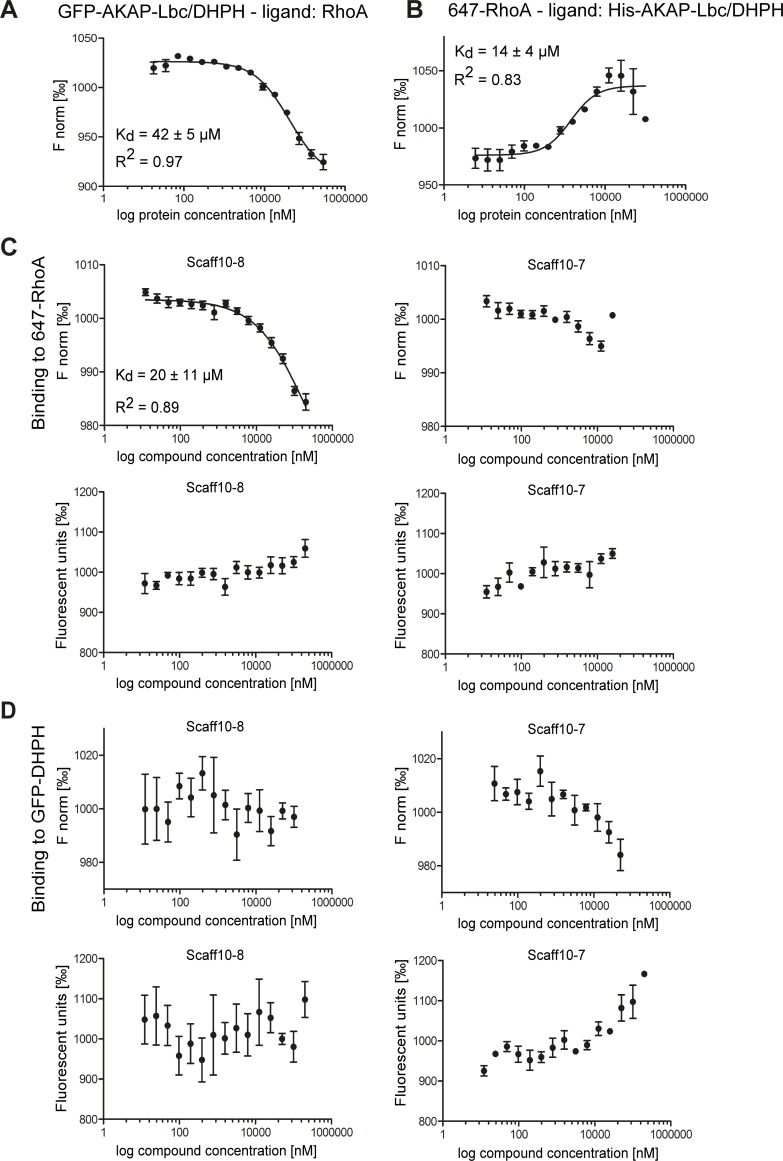
The Scaff10 derivative Scaff10-8 binds to RhoA but not to the DHPH domain of AKAP-Lbc. (A-D) Microscale thermophoresis (MST) takes advantage of the phenomenon of directed movement of particles in a temperature gradient. Binding events lead to changes in the hydration shell of biomolecules and a relative change of movement of the molecular complex along a temperature gradient. Using such changes, binding affinities can be determined [47]. MST assays were carried out with (A) the recombinant DHPH domain of AKAP-Lbc fused with GFP and RhoA as a ligand and (B) fluorescent 647-RhoA and the His-tagged AKAP-Lbc/DHPH domain as a ligand. (C) MST assays for the analysis of the binding of Scaff10-8 to 647-RhoA. Upper panels: The concentration of fluorescent 647-RhoA remained constant and Scaff10-8 (left) and Scaff10-7 as a negative control (right) were titrated in increasing concentrations. Lower panels: Values of fluorescence corresponding to upper panels. (D) MST assay showing no binding of Scaff10-8 or Scaff10-7 to GFP-DHPH. Upper panels: The concentration of fluorescent GFP-AKAP-Lbc/DHPH remained constant and Scaff10-8 (left) and Scaff10-7 (right) were titrated in increasing concentrations. Lower panels: Values of fluorescence corresponding to upper panels. The K_D_ value for the binding of Scaff10-8 to 647-RhoA is 20 ± 11 μM. F norm = normalized fluorescence (fluorescence steady state/fluorescence initial state) indicated in ‰. n = 3–5. Mean ± SEM.

Microscale thermophoresis (MST) was used to identify the target of Scaff10 derivatives [47]. Since the readout of MST is fluorescence emission, recombinant GFP-tagged AKAP-Lbc/DHPH and RED-NHS-labeled RhoA (647-RhoA) were generated. GFP-AKAP-Lbc/DHPH concentration-dependently bound unlabeled RhoA with a K_D_ value of 42 ± 5 μM (Fig 4A). 647-RhoA bound His-AKAP-Lbc/DHPH (protein also used for the HTRF assay) with a K_D_ value of 14 ± 4 μM (Fig 4B). The shape of the binding curve was inverted because of the altered ratios of RhoA to AKAP-Lbc/DHPH: F_norm_ values became high when AKAP-Lbc/DHPH was present in excess, whereas F_norm_ values decrease when RhoA was present in excess.

The target of Scaff10-8 (and Scaff10-38, Fig 3 in S3 in S1 File) is RhoA, as Scaff10-8 bound to 647-RhoA with a K_D_ value of 20 ± 11 μM (Fig 4C). Scaff10-38 bound to 647-RhoA with a similar K_D_ value (Fig 3 in S3 in S1 File). Scaff10-7 and also other compounds from the different Scaff10 classes (e.g. Scaff10-48 from class 8, Fig 3 in S3 in S1 File) that were inactive in our HTRF assay, did not bind 647-RhoA, and neither Scaff10-8, Scaff10-7, Scaff10-38 or Scaff10-48 bound GFP-AKAP-Lbc/DHPH (Fig 4D, Fig 3 in S3 in S1 File). The concentration of the fluorescent molecule remained constant in the samples during each run. Based on this, the decreasing values for Scaff10-7 (Fig 4D) and of other HTRF-negative compounds such as Scaff10-48 (Fig 3 in S3 in S1 File) were not considered as binding events, but as a direct interaction with GFP of the DHPH domain, which influences the F_norm_ value concentration-dependently.

**Fig 5:**
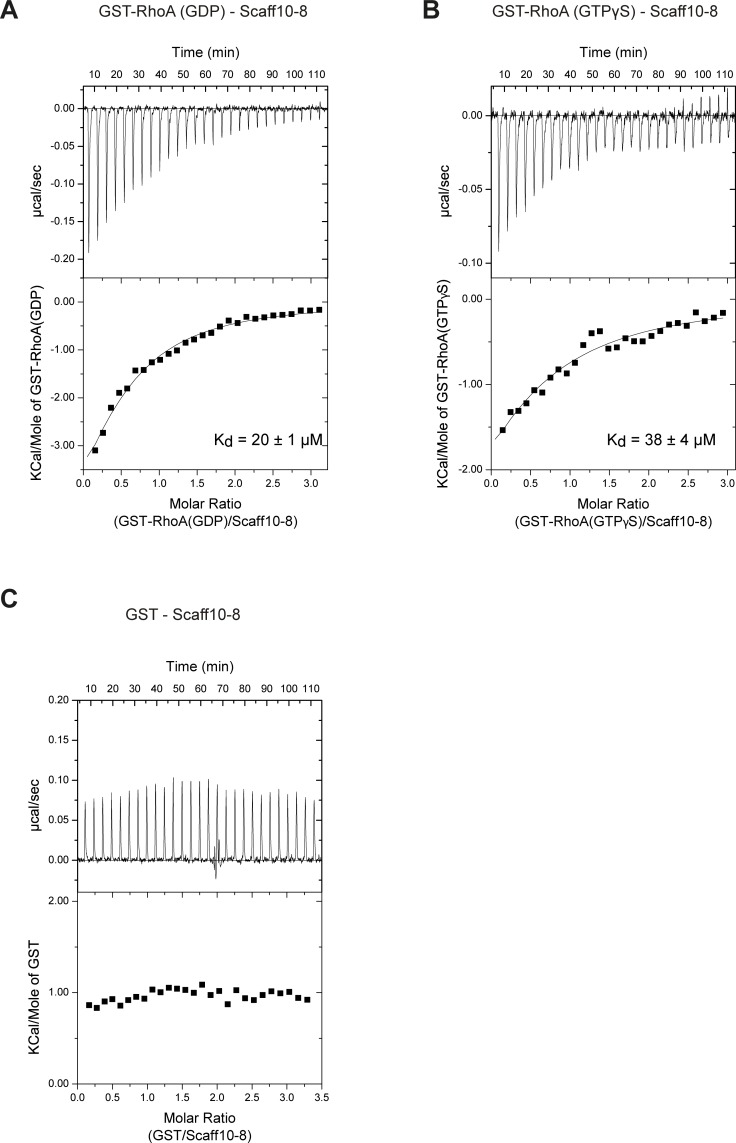
Scaff10-8 binds to GDP- and GTP-bound RhoA. Isothermal titration calorimetry (ITC) measurements using recombinant GST-tagged RhoA in its (A) GDP- and (B) GTP-bound form and Scaff10-8 were performed (GDP and GTPγS, 2 mM each). K_D_ values are indicated. (C) As a control, the measurements were carried out with GST alone.

We used isothermal titration calorimetry (ITC) assays to confirm the interaction of Scaff10-8 with RhoA (Fig 5A and 5B). Indeed, Scaff10-8 bound GST-RhoA loaded with GDP with a K_D_ = 20 ± 1 μM or GTPγS with a K_D_ = 38 ± 4 μM. Since GTP-GDP exchange is associated with large conformational rearrangements of the switch regions, the similar K_D_ values suggest that interaction with Scaff10-8 does not directly involve these elements and that it was independent of the conformational change. Scaff10-8 specifically interacted with RhoA, as it did not bind to the GST control (Fig 5C).

### Scaff10-8 does not affect viability and inhibits the AKAP-Lbc-mediated activation of RhoA in cells

**Fig 6:**
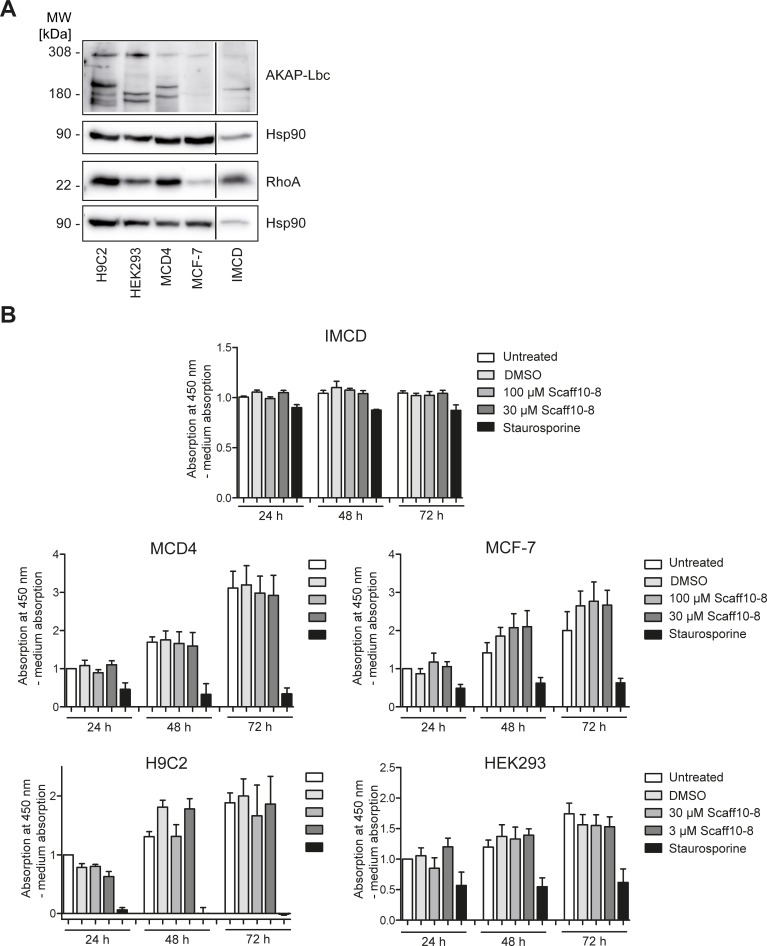
Scaff10-8 is not cytotoxic. (A) Western blot showing protein expression of AKAP-Lbc and RhoA in five different cell lines and in primary rat inner medullary collecting duct (IMCD) cells: All cells express full-length AKAP-Lbc (308 kDa) and shorter splice variants. RhoA (22 kDa) is also expressed in all cells. MW = molecular weight. (B) Cytotoxicity was assessed after 24–72 h of incubation with Scaff10-8 (3, 30, 100 μM). Staurosporine as inducer of apoptosis was used as a positive control. Absorption was determined at 450 nm and was related to medium absorption (blank). DMSO was used as a control. Normalised to untreated cells after 24 h, three independent experiments with four data points each are shown. n = 3. Statistically significant differences were determined applying one-way ANOVA with posthoc Bonferroni. Mean ± SEM is plotted.

Prior to the analysis of effects of Scaff10-8 on the localization of AQP2 in primary rat inner medullary collecting duct (IMCD) cells, potential cytotoxic effects and specificity of the compound were evaluated. For this, IMCD cells, mouse collecting duct MCD4 cells (another model for renal principal cells [44, 48]), Human embryonic kidney (HEK)293, breast carcinoma (MCF7) and cardiac myocytes (H9C2) that all express AKAP-Lbc and RhoA (Fig 6A) were incubated with Scaff10-8 in concentrations of 3–100 μM and an MTS cytotoxicity assay was carried out (Fig 6B). The compound did not influence the viability of these cells. A further experiment based on quantitation of ATP as an indicator of metabolically active cells confirmed that Scaf10-8 did not affect the viability of cells in concentrations of up to 100 μM (Fig 4 in S4 in S1 File). Using MCD4 cells and a mass spectrometry approach, we confirmed the uptake of Scaff10-8 into cells (data not shown).

**Fig 7:**
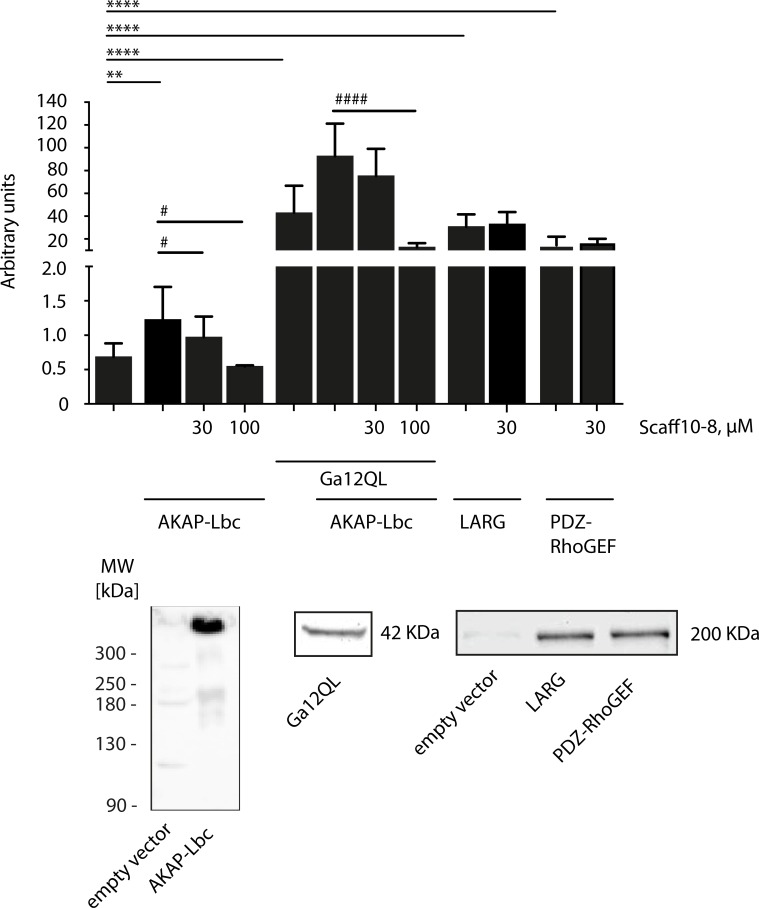
Scaff10-8 inhibits the AKAP-Lbc-mediated activation of RhoA in cells. In HEK293 cells, full length AKAP-Lbc was transiently expressed alone or in combination with the constitutively active version, Gα12QL, of the G protein α subunit Gα12. Gα12 selectively activates the GEF activity of AKAP-Lbc [28]. Where indicated, the RhoGEFs LARG and PDZ-RhoGEF were expressed. The cells were left untreated or incubated with Scaff10-8 in concentrations of 30 or 100 μM. The lower panels show detection of the indicated proteins by Western blotting. n = 3. Statistically significant differences were determined applying one-way ANOVA with posthoc Bonferroni. Mean ± SEM is plotted.

In order to test whether Scaff10-8 would, as the mother compound Scaff10, have specificity towards inhibition of AKAP-Lbc-mediated RhoA activation, we expressed AKAP-Lbc in the absence or presence of a constitutively active form of the α-subunit of G12, Gα12QL, or the RhoGEFs LARG and PDZ-RhoGEF in HEK293 cells and analyzed the effect of Scaff10-8 on the activation of RhoA. Fig 7 shows that only the AKAP-Lbc-mediated activation of RhoA was inhibited, whereas LARG and PDZ-RhoAGEF-mediated RhoA activation was not influenced, indicating that Scaff10-8 has a similar selectivity as Scaff10.

### Scaff10-8 promotes the redistribution of AQP2 to the periphery of primary IMCD cells

**Fig 8:**
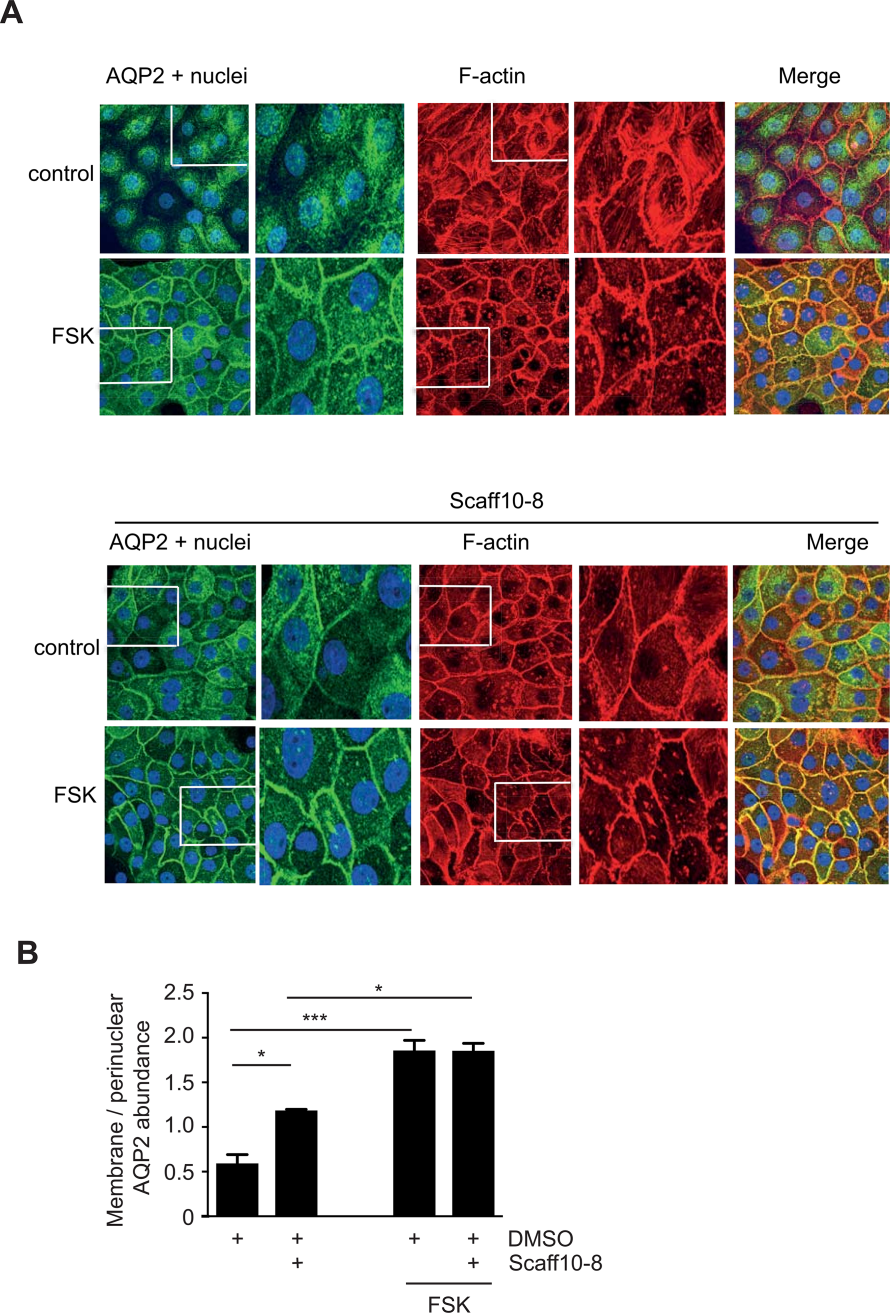
Scaff10-8 promotes the redistribution of AQP2 to the plasma membrane of primary IMCD cells, which is independent of cAMP elevation and associated with depolymerization of F-actin. (A) Upper panel. IMCD cells were treated with DMSO (1%; control), the solvent of Scaff10-8, or forskolin (10 μM, 30 min) to stimulate cAMP synthesis. Lower panels: The cells were treated for 1 hour with Scaff10-8 (30 μM) alone (control) or with Scaff10-8 and forskolin. Forskolin (10 μM) was added for the final 30 min of Scaff10-8 incubation. AQP2 (green) was detected with specific primary and Cy3-coupled secondary antibodies, F-actin (red) with Alexa Fluor 647-Phalloidin and nuclei with DAPI (blue). Signals were visualized using a laser scanning microscope. Representative images are shown. n = 3. The magnified views were derived from the indicated boxes. (B) The signal intensities arising from intracellular and plasma membrane AQP2 were recorded, related to nuclear signal intensities, and the ratios of plasma membrane to intracellular fluorescence signal intensities were calculated (n = 30 cells per condition). Ratios > 1 indicate a predominant localization of AQP2 at the plasma membrane. Statistically significant differences were calculated using one-way ANOVA with posthoc Bonferroni. Mean ± SEM; *, p ≤ 0.05; *** p ≤ 0.001.

To investigate whether Scaff10-8 affected the localization of AQP2, primary IMCD cells were treated with Scaff10-8 and AQP2 was visualized using immunofluorescence microscopy (Fig 8). In resting IMCD cells, AQP2 was located mainly in the perinuclear region. As previously demonstrated, stimulation of cAMP synthesis with the adenylyl cyclase activator forskolin induced its redistribution to the plasma membrane. Incubation with Scaff10-8 (30 μM, 1 h) caused the translocation of AQP2 to the plasma membrane in the absence of forskolin, while it did not enhance the forskolin-induced redistribution. In control cells, polymerized F-actin appeared mostly as fiber structures and elevation of cAMP through forskolin caused depolymerization as previously described [23]. F-actin disappeared and dot-like structures became apparent, presumably representing shorter fragments of F-actin or G-actin [23] (Fig 8). In cells challenged with 30 μM Scaff10-8 alone, F-actin also decreased and more dot-like structures were present. Scaff10-8 did not influence the effect of forskolin on F-actin. The data argue for a Scaff10-8-induced decrease of F-actin, which is in line with an inhibition of RhoA. In MCD4 cells, Scaff10-8 also induced cAMP-independently the translocation of AQP2 to the plasma membrane (Fig 5 in S5 in S1 File). Scaff10-7 and Scaff10-59, compounds without inhibitory effects in our HTRF and Alpha screen assays, did not affect the location of AQP2 in MCD4 cells (Fig 5 in S5 in S1 File). Due to the lack of an effect of the latter compounds on AQP2, we refrained from testing them on primary IMCD cells.

**Fig 9:**
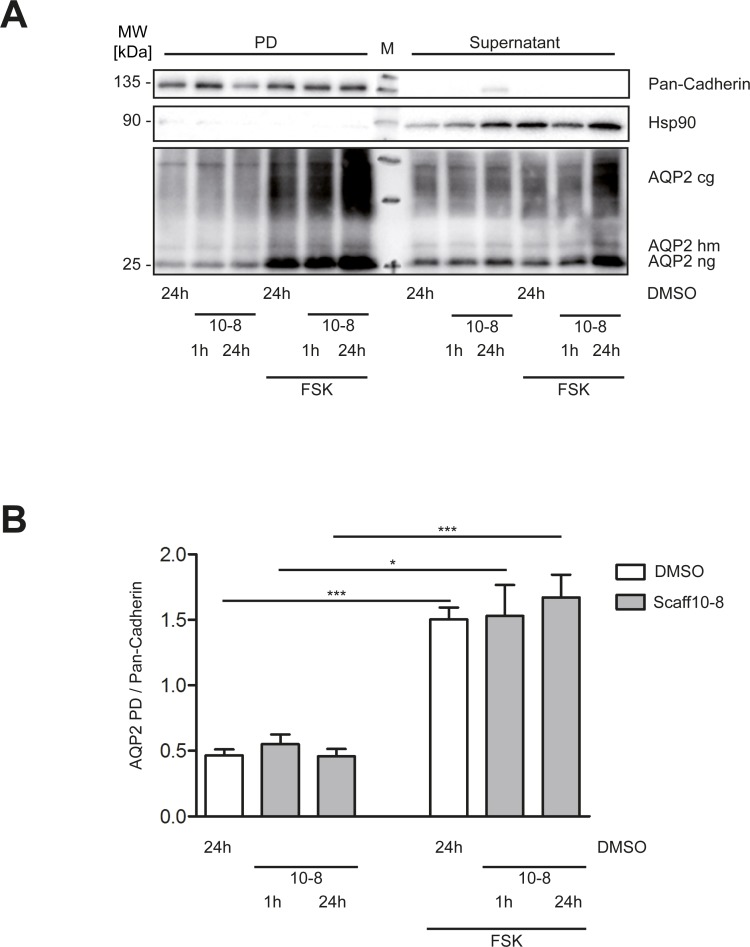
Scaff10-8 does not promote the insertion of AQP2 into the plasma membrane of IMCD cerlls. (A) IMCD cells were treated with either compound (30 μM) or DMSO for 1 or 24 h. Membrane proteins were biotinylated followed by precipitation with streptavidin agarose beads. Pulldown (PD) and supernatant fractions were separated by 12% SDS-PAGE. Pan-Cadherin was used as a loading control for the PD fraction, Hsp90 for the supernatants. A representative Western blot is shown. (B) Signals from A were quantified by densitometric analysis. The amount of AQP2 in the PD fractions were related to Pan-Cadherin. MW = molecular weight. M = molecular weight standard. cg = complex glycosylated; hm = high mannose glycosylated; ng = non-glycosylated. 10–8 = Scaff10-8. n = 3. Mean ± SEM is plotted.

Since the immunofluorescence microscopy experiments indicated a Scaff10-8-induced redistribution of AQP2 to the plasma membrane, we investigated whether AQP2 inserted into the membrane. However, surface biotinylation experiments revealed that this was not the case. Forskolin alone induced the membrane insertion of AQP2 into the plasma membrane of IMCD cells but not Scaff10-8 (Fig 9). Thus, Scaff10-8 appears to promote the redistribution of AQP2 to a near-plasma membrane region of the IMCD cells but not its insertion.

**Fig 10:**
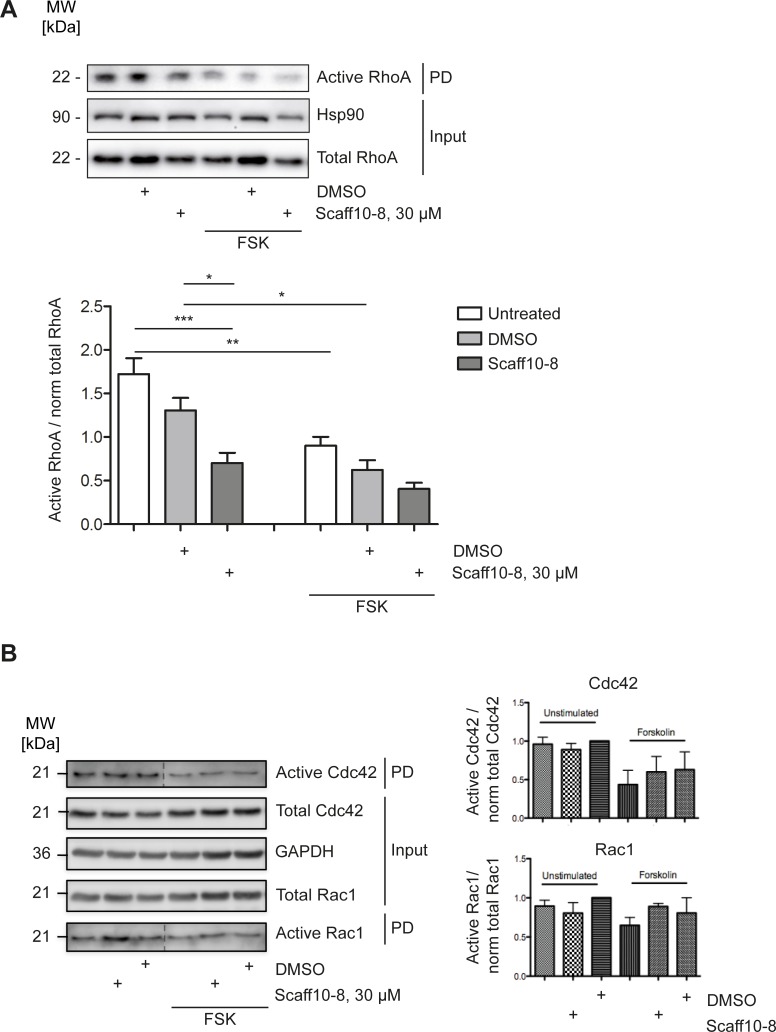
Scaff10-8 inhibits activation of RhoA in primary IMCD cells. IMCD cells were incubated with Scaff10-8 (30 μM, 1 h). DMSO (1%), the solvent of Scaff10-8, served as a control. The cells were lysed, (A) active RhoA was precipitated with the RhoA binding domain of Rhotekin coupled to sepharose beads, (B) active Cdc42 and Rac1 were precipitated with with GST fused to the (p21) binding domain (PBD) of p21 activated kinase 1 protein (PAK-1) coupled to sepharose beads. Inputs and pulldown fractions were separated by SDS-PAGE. Hsp90 or GAPDH were used as loading controls. Representative Western blots from 3–5 independent experiments are shown. Signals were semiquantitatively analyzed by densitometry. The amount of active RhoA was related to normalized RhoA (total RhoA to Hsp90 (Input)). Accordingly, active Cdc42 and Rac1 were related to normalized Cdc42 and Rac1, respectively (total RhoA to GAPDH (Input)). PD, Pulldown. n = 3–5. Statistically significant differences were determined using one-way ANOVA with posthoc Bonferroni. Mean ± SEM is plotted. *, p ≤ 0.05; **, p ≤ 0.01; *** p ≤ 0.001.

Next, we investigated the effect of Scaff10-8 on RhoA activity. In order to measure RhoA activity in IMCD cells, Rhotekin pull down assays were performed (Fig 10). They revealed that Scaff10-8 alone decreased RhoA activity as forskolin did. Scaff10-8 did not significantly enhance the inhibitory effect of forskolin. The inhibitory effect of Scaff10-8 on RhoA explains the observed depolymerization of F-actin and the AVP/cAMP-independent redistribution of AQP2 to the periphery of the IMCD cells.

The Rho GTPases, Cdc42, and Rac1 are crucial regulators of the cytoskeleton [49]. In order to determine whether Scaff10-8 non-selectively affects these members of the Rho family in addition to RhoA, we precipitated the active GTP-bound forms using GST fused to the (p21) binding domain (PBD) of p21 activated kinase 1 protein (PAK-1). Fig 10B shows that Scaff10-8 did not influence activities of Cdc42 or Rac1 underpinning the selectivity for interference with AKAP-Lbc/RhoA signaling.

## Discussion

We report here a novel small molecule, Scaff10-8. We were able to demonstrate that this compound bound RhoA and inhibited its interaction with AKAP-Lbc. In IMCD cells, Scaff10-8 caused inhibition of RhoA and promoted the redistribution of AQP2 to the periphery of the cells in the absence of AVP. This effect of Scaff10-8 was associated with depolymerization of F-actin. The results concur with our previous observation that a basal RhoA activity in resting IMCD cells maintains F-actin as a physical barrier preventing AQP2-bearing vesicles from reaching the plasma membrane [23]. Our data indicate that under resting conditions a basal GEF activity of AKAP-Lbc maintains a pool of RhoA in an active state. The decrease of RhoA activity upon challenge with AVP is due to PKA phosphorylation of RhoA at S188 and its consequent inhibition [23–25, 45]. In addition, PKA phosphorylation and thus inhibition of the GEF activity of AKAP-Lbc as it occurs in HEK293 cells is likely to contribute to RhoA inhibition [32]. This argues for several layers of control ensuring a tight regulation of RhoA and thereby of the localization of AQP2.

We have previously shown that prostaglandin E_2_ antagonizes the AVP-induced redistribution of AQP2 through stimulation of EP_3_ receptors [45]. The inhibitory effect was independent of EP_3_ receptor coupling to the inhibitory G protein G_i_ and inhibition of adenylyl cyclase and cAMP generation. The EP_3_ receptor-mediated inhibition was due to RhoA activation and formation of F-actin-containing stress fibers. Stimulation of EP_3_ receptor subtypes α and β activates the G protein G_12_ in addition to G_i_ [50]. Since the GEF activity of AKAP-Lbc is selectively activated by G_12_ [28], AKAP-Lbc may not only maintain a basal RhoA activity in resting IMCD cells, but may be part of the signaling cascade controlling the inhibitory effects of prostaglandin E_2_ on diuresis.

Scaff10-8 did not promote the membrane insertion of AQP2 in IMCD cells. We had previously observed that direct inhibition of RhoA with C3 toxin promotes the depolymerization of F-actin and the redistribution of AQP2 to the plasma membrane [23]. Whether C3 toxin induces the insertion into the plasma membrane was not evaluated. However, there is ample evidence from other studies that inhibition of Rho causes membrane insertion of the water channel. For the treatment of water balance disorders, in particular, forms of DI where a redistribution of AQP2 into the plasma membrane independent of AVP and the V2R is desirable, treatment with statins as a measure for interference with Rho activity has been suggested. Statins block HMG-CoA reductase and thereby inhibit lipid modification and activity of RhoA, and also enhance AQP2 expression [51]. For example, simvastatin controls AQP2 trafficking in porcine LLC-PK1 cells stably expressing AQP2, and urinary concentration in Brattleboro rats through downregulation of Rho activity and inhibition of endocytosis. Statins inhibit clathrin-mediated endocytosis [51, 52]. In hypercholesterolemic patients, simvastatin reduced diuresis and increased urine osmolality [53, 54]. Scaf10-8 may not induce the membrane insertion of AQP2 because interference with a single RhoGEF may not be sufficient to inactivate the relevant RhoA pool to a degree that leads to membrane insertion. The peripheral F-actin cytoskeleton may be under the control of one or more other GEF/s and, in the presence of Scaff10-8, may still constitute a physical barrier for AQP2-bearing vesicles preventing them from reaching the plasma membrane. Cdc42 and Rac1 are most likely not involved in maintaining peripheral F-actin as a barrier, as Scaff10-8 did not affect their activity.

Recently, a virtual screening identified small molecule inhibitors of the AKAP-Lbc/DHPH-RhoA interaction [55]. In the docked structural models, negatively charged molecules targeted a cluster of positively charged residues of the AKAP-Lbc/DHPH domain. One of the molecules, A13, reduced proliferation, migration and invasion of prostate cancer cells. This is in line with previous findings showing that AKAP-Lbc/DHPH alone is constitutively active and promotes cell transformation and cancer cell proliferation [56]. However, another small molecule, C646, almost identical to A13, was identified as a pan-assay interference compound (PAINS) [57]. PAINS are compounds that tend to nonspecifically react with various targets and are often false positive hits in high-throughput screenings. We, therefore, refrained from testing A13 in IMCD cells. Additional small molecules targeting the binding sites of RhoA in AKAP-Lbc were recently identified through an *in silico* approach. However, it is unclear whether these *in silico* hits inhibit the interaction with RhoA [58]. We, therefore, also did not test the *in silico*-derived molecules in IMCD cells.

The AKAP-Lbc-mediated RhoA activation is associated with the development of cardiac hypertrophy [59] and cancer [60]. Therefore, the development of an inhibitor of the interaction has clinical implications. However, the target of Scaff10-8 is RhoA and not AKAP-Lbc. Since a drug binding RhoA would be expected to cause side effects, Scaff10-8 is apparently not suitable for further development towards a drug candidate, although, in principle, targeting Rho is possible. For instance, the Rho inhibitor VX-210 is currently being clinically tested for the treatment of traumatic cervical spinal cord injury (ClinicalTrials.gov Identifier: NCT02669849). Inhibitors of the Rho effector, Rho kinase (ROCK; Fasudil and Ripasudil), which is ubiquitously expressed, have been approved for the treatment of glaucoma and vasculature-related diseases [61].

In summary, with our novel small molecule, Scaff10-8, that targets the interaction of a Rho-specific GEF with RhoA to interfere with RhoA activity, we provide a novel research tool for studying functions of the AKAP-Lbc-RhoA interaction. Using Scaff10-8, we have identified the first RhoGEF involved in the control of AQP2 trafficking.

## Supporting information

S1 File **S1 Fig. Scaff10 specifically inhibits the Gα12-AKAP-Lbc-mediated activation of RhoA.** (A) Full length AKAP-Lbc (AKAP13) and the indicated GEFs were transiently expressed in HEK293 cells. Upper panel: Scaff10 concentration-dependently inhibits the AKAP-Lbc-mediated stress response element-induced luciferase activity but at a concentration of 50 μM does not affect increases in luciferase activity induced by other GEFs. n = 3; statistically significant differences were not found, as confirmed by statistical analysis using ANOVA. (B) Scaff10 inhibits Gα12- but not Gαq-mediated RhoA activation. The indicated Gα12 and Gαq-mutants were transiently co-expressed with AKAP-Lbc and p63RhoGEF in HEK293 cells as indicated. The cells were left untreated or incubated with Scaff10 (50 μM). The fraction of active RhoA was determined using Rhotekin pulldown assays. Shown is one representative out of three experiments. All GEF constructs were in pCMV3a and Myc-tagged (30 ng per transfection). Expression of AKAP-Lbc, LARG and PDZ-RhoGEF is shown in Fig 7 of the main manuscript. **S2 Fig.** (A) **The sequence of the AKAP-Lbc/DHPH domain** (5,914–7,005 bp, NCBI accession number CCDS32320.1) was mutated in order to remove rare codons and genetically fused with GFP and a His_6_ tag. Substituted base pairs are indicated in red. (B) **Purified recombinant proteins for subsequent assays.** SDS-PAGE followed by Coomassie staining: His6-AKAP-Lbc/DHPH (46 kDa) and GST-RhoA (48 kDa) were used for the HTRF assay and ITC; untagged RhoA (22 kDa) and GST were used for ITC (28 kDa). 2 μg of each protein were loaded. **S3 Fig. Scaff10 derivatives, Scaff10-8 and Scaff10-38 inhibiting the AKAP-Lbc-RhoA interaction in our HTRF assay bound RhoA, not the AKAP-Lbc/DHPH domain.** Results of MST assays. (A) Upper panel: The concentration of fluorescent 647-RhoA remained constant whereas Scaff10-8, Scaff10-38, Scaff10-7 and Scaff10-48 (ligands) were added in increasing concentrations. The Kd values for the binding of Scaff10-8 and Scaff10-38 to RhoA are 20.2 ± 11.1 μM and 19.5 ± 4.1 μM, respectively. Lower panel: Corresponding values of fluorescence to upper panel. (B) Upper panel: The concentration of fluorescent GFP-AKAP-Lbc/DHPH remained constant whereas Scaff10-8, Scaff10-38, Scaff10-7 and Scaff10-48 (ligands) were added in increasing concentrations. Lower panel: Corresponding values of fluorescence to upper panel. (B) F norm = normalised fluorescence (fluoresence steady state/fluorescence initial state) indicated in ‰. Compound concentration [nM] refers to the appropriate ligand. n = 3–5. Mean ± SEM is plotted. Kd values are indicated ± SEM. R^2^ indicates the coefficient of determination. The results of the Scaff10-8 and Scaff10-7 derivatives are shown in the main manuscript and are here included for direct comparison. **S4 Fig. Scaff10-8 does not affect viability of cells.** The viability of the indicated cells was determined using a CellTiter-Glo^®^Luminescent Cell Viability Assay (Promega, Wisconsin, USA) according to the manufacturer's instructions. The assay is based on the quantification of ATP as an indicator of metabolically active cells. The cells were seeded, grown for 24 h and treated with the different concentrations of Scaff 10–8. Viability was assessed after 1 and 24 h incubation with Scaff10-8 in the indicated concentrations. An equal volume of CellTiter-Glo Reagent was added, plates were shaken and luminescence monitored over time with the plates held at 22°C. Mono-oxygenation of luciferin is catalyzed by luciferase in the presence of Mg^2+^, ATP and molecular oxygen. DMSO was used as a control. Relative luminescent signals (RLU) was measured and normalized to untreated cells, n = 3. Statistically significant differences were determined applying one-way ANOVA with posthoc Bonferroni. Mean ± SD is plotted. **S5 Fig.** Scaff10-8 but not Scaff10-7 or Scaff10-59 induced the translocation of AQP2 to the plasma membrane of MCD4 cells independent of cAMP elevation. MCD4 cells were left untreated (A) or incubated with 30 (B) or 3 μM (C) Scaff10-8 and stimulated with forskolin (FSK). DMSO was used as a control (A). As negative controls, the cells were incubated with Scaff10-7 and Scaff10-59 (30 μM each), which were inactive in our HTRF assay. AQP2 stained in green, nuclei stained in blue, F-actin stained in red. Scale bar indicates 20 μm. Representative images are shown. n = 3. **S1 Table.** A guanine nucleotide exchange assay for RhoA activation after addition of the DHPH domain of AKAP-Lbc identified 655 inhibitors in a screen of 18,431 compounds. The table shows chemical structures of the compounds. **S2 Table.** Compounds tested for dose-dependent inhibition of AKAP-Lbc-mediated RhoA activation using a guanine nucleotide exchange assay. Of the primary hits 100 and 252 additional structurally related compounds from the library were tested for activity (1, 10 and 100 μM). **S3 Table. Overview of all 55 Scaff10 derivatives**. Structures labeld with ^1^ were commercially obtained. MW = molecular weight. logP = calculated partition coefficient (using Biovia Draw V.16.1), indicates a compounds’s distribution in a biphasic system consisting of ocatanol-water. Disruption of the interaction of RhoA and AKAP-Lbc/DHPH (PPI) and IC_50_ values refer to HTRF assays (Fig 3 main manuscript), IC_50_ values ± SEM; all HTRF experiments were performed in duplicate; X-axis = compound concentration [μM], Y-axis = relative interaction of AKAP-Lbc/DHPH and RhoA (%). Solubility was assessed nephelometrically in DMEM + 10% FCS + Scaff10 derivative with either 0.3 or 2% DMSO final concentration; values in μM indicate maximal solubility of the compound under the respective condition. Yield in % indicates total yield of the last step of chemical synthesis for each of Scaff10 derivatives. n.d. = not determined. n.i. = not indicated.
(PDF)
