# Supplementary material for: An AKAP-Lbc-RhoA interaction inhibitor promotes the translocation of aquaporin-2 to the plasma membrane of renal collecting duct principal cells
Source: PLoS One. 2018 Jan 26;13(1):e0191423. doi: 10.1371/journal.pone.0191423 (PMC5786306; doi:10.1371/journal.pone.0191423)
Supplement: S1 File — S1 Fig. Scaff10 specifically inhibits the Gα12-AKAP-Lbc-mediated activation of RhoA. (A) Full length AKAP-Lbc (AKAP13) and the indicated GEFs were transiently expressed in HEK293 cells. Upper panel: Scaff10 concentration-dependently inhibits the AKAP-Lbc-mediated stress response element-induced luciferase activity but at a concentration of 50 μM does not affect increases in luciferase activity induced by other GEFs. n = 3; statistically significant differences were not found, as confirmed by statistical analysis using ANOVA. (B) Scaff10 inhibits Gα12- but not Gαq-mediated RhoA activation. The indicated Gα12 and Gαq-mutants were transiently co-expressed with AKAP-Lbc and p63RhoGEF in HEK293 cells as indicated. The cells were left untreated or incubated with Scaff10 (50 μM). The fraction of active RhoA was determined using Rhotekin pulldown assays. Shown is one representative out of three experiments. All GEF constructs were in pCMV3a and Myc-tagged (30 ng per transfection). Expression of AKAP-Lbc, LARG and PDZ-RhoGEF is shown in Fig 7 of the main manuscript. S2 Fig. (A) The sequence of the AKAP-Lbc/DHPH domain (5,914–7,005 bp, NCBI accession number CCDS32320.1) was mutated in order to remove rare codons and genetically fused with GFP and a His6 tag. Substituted base pairs are indicated in red. (B) Purified recombinant proteins for subsequent assays. SDS-PAGE followed by Coomassie staining: His6-AKAP-Lbc/DHPH (46 kDa) and GST-RhoA (48 kDa) were used for the HTRF assay and ITC; untagged RhoA (22 kDa) and GST were used for ITC (28 kDa). 2 μg of each protein were loaded. S3 Fig. Scaff10 derivatives, Scaff10-8 and Scaff10-38 inhibiting the AKAP-Lbc-RhoA interaction in our HTRF assay bound RhoA, not the AKAP-Lbc/DHPH domain. Results of MST assays. (A) Upper panel: The concentration of fluorescent 647-RhoA remained constant whereas Scaff10-8, Scaff10-38, Scaff10-7 and Scaff10-48 (ligands) were added in increasing concentrations. The Kd values for the binding of Scaff10-8 an [file pone.0191423.s001.pdf]

Supplemental information

An AKAP-Lbc-RhoA interaction inhibitor promotes the translocation of aquaporin-2 to the plasma membrane of renal collecting duct principal cells

Katharina Schrade[1¶], Jessica Tröger[1¶], Adeeb Eldahshan[1¶], Kerstin Zühlke[1], Kamal R. Abdul Azeez[2], Jonathan M. Elkins[2], Martin Neuenschwander[3], Andreas Oder[3], Mohamed Elkewedi[1], Sarah Jaksch[1], Karsten Andrae[4], Jinliang Li[5], Joao Fernandes[1], Paul Markus Müller[1], Stephan Grunwald[1], Stephen F. Marino[1], Tanja Vukićević[1], Jenny Eichhorst[3], Burkhard Wiesner[3], Marcus Weber[4], Michael Kapiloff[5], Oliver Rocks[1], Oliver Daumke[1], Thomas Wieland[6,7], Stefan Knapp[2,8,9], Jens Peter von Kries[3], Enno Klussmann[1,10*]

Affiliations

[1]Max Delbrück Center for Molecular Medicine Berlin (MDC), Germany

[2]Structural Genomics Consortium, University of Oxford, United Kingdom

[3]Leibniz-Forschungsinstitut für Molekulare Pharmakologie (FMP), Berlin, Germany

[4]Zuse Institute Berlin (ZIB), Germany

[5]University of Miami Miller School of Medicine, Miami, USA

[6]Institute of Experimental Pharmacology and Toxicology, Medical Faculty Mannheim, Heidelberg University, Germany

[7]DZHK (German Centre for Cardiovascular Research), partner site Heidelberg/Mannheim, Germany

[8]Institute for Pharmaceutical Chemistry and Buchmann Institute, Goethe University, Frankfurt, Germany

[9]DKTK (German Cancer Center Network), partner site Frankfurt/Main, Germany

[10]DZHK (German Centre for Cardiovascular Research), partner site Berlin, Germany

Running Title: Disruption of AKAP-Lbc-RhoA interaction with small molecules

***Corresponding author**

enno.klussmann@mdc-berlin.de

Content

1 **Results** 5

1.1 **Supplementary figures 1-5** 5

1.2 **Supplementary Tables** 11

1.2.1 **Suppl. Tab. 1** 11

1.2.2 **Suppl. Tab. 2** 106

1.2.3 **Suppl. Tab. 3** 159

2 **Material and Methods** 172

2.1 **Cell lines** 172

2.2 **Plasmids** 172

2.3 **Luciferase reporter assays** 173

2.4 **Chemical synthesis** 173

2.4.1 **^{1}H NMR** 173

2.4.2 **LC/MS** 173

2.4.3 **HPLC** 174

3 **References** 175

4 **Medicinal Chemistry** 176

4.1 **Chemical synthesis** 176

4.1.1 Iodo-1-[4-(3-methoxyphenyl)phenyl]ethanone (**2a**) 176

4.1.2 Ethyl 3-(7-hydroxy-4-methyl-2-oxo-chromen-3-yl)propanoate (**5a**) 179

4.1.3 [2-(4-Methoxyphenyl)-2-oxo-ethoxy]-3,4-dimethyl-chromen-2-one (**AE168**) 182

4.1.4 3-(4-Methoxyphenyl)-5,6-dimethyl-furo[3,2-*g*]chromen-7-one (**Scaff10**) 185

4.1.5 7-[2-(4-Methoxyphenyl)-2-oxo-ethoxy]chromen-2-one (**AE167**) 188

4.1.6 3-(4-Methoxyphenyl)furo[3,2-*g*]chromen-7-one (**Scaff10-1**) 191

4.1.7 3,4-Dimethyl-7-[2-oxo-2-(3-pyridyl)ethoxy]chromen-2-one (**Scaff10-2**) 194

4.1.8 5,6-Dimethyl-3-(3-pyridyl)furo[3,2-*g*]chromen-7-one (**Scaff10-3**) 197

4.1.9 7-[2-[6-(4-Fluorophenyl)-2-methyl-3-pyridyl]-2-oxo-ethoxy]-3,4-dimethyl-chromen-2-one (**Scaff10-4**) 200

4.1.10 3-[6-(4-Fluorophenyl)-2-methyl-3-pyridyl]-5,6-dimethyl-furo[3,2-*g*]chromen-7-one (**Scaff10-5**) 203

4.1.11 3-[7-[2-(4-Methoxyphenyl)-2-oxo-ethoxy]-4-methyl-2-oxo-chromen-3-yl]propanoic acid (**Scaff10-6**) 206

4.1.12 Ethyl 3-[7-[2-(4-methoxyphenyl)-2-oxo-ethoxy]-4-methyl-2-oxo-chromen-3-yl]propanoate (**Scaff10-7**) 209

4.1.13 3-[3-(4-Methoxyphenyl)-5-methyl-7-oxo-furo[3,2-*g*]chromen-6-yl] propanoic acid (**Scaff10-8**) 212

4.1.14 *N*-[2-[3-[7-[2-(4-Methoxyphenyl)-2-oxo-ethoxy]-4-methyl-2-oxo-chromen-3-yl]propanoylamino]ethyl]-5-(2-oxo-1,3,3*a*,4,6,6*a*-hexahydrothieno[3,4-*d*]imidazol-4-yl)pentanamide (**Scaff10-9**) 215

4.1.15 Ethyl 3-[7-[2-[6-(4-fluorophenyl)-2-methyl-3-pyridyl]-2-oxo-ethoxy]-4-methyl-2-oxo-chromen-3-yl]propanoate (**Scaff10-10**) 217

4.1.16 3-[7-[2-[6-(4-Fluorophenyl)-2-methyl-3-pyridyl]-2-oxo-ethoxy]-4-methyl-2-oxo-chromen-3-yl]propanoic acid (**Scaff10-11**)....220
4.1.17 3,4-Dimethyl-7-[2-(2-naphthyl)-2-oxo-ethoxy]chromen-2-one (**Scaff10-12**)....222
4.1.18 3,4-Dimethyl-7-[2-oxo-2-(4-phenylphenyl)ethoxy]chromen-2-one (**Scaff10-13**)....225
4.1.19 3,4-Dimethyl-7-[2-oxo-2-(4-phenoxyphenyl)ethoxy]chromen-2-one (**Scaff10-14**)....228
4.1.20 7-[2-(6-Methoxy-2-naphthyl)-2-oxo-ethoxy]-3,4-dimethyl-chromen-2-one (**Scaff10-15**) 231
4.1.21 Ethyl 3-[4-methyl-2-oxo-7-[2-oxo-2-(4-phenoxyphenyl)ethoxy]chromen-3-yl]propanoate (**Scaff10-17**)....234
4.1.22 3-[5-Methyl-3-(2-naphthyl)-7-oxo-furo[3,2-*g*]chromen-6-yl]propanoic acid (**Scaff10-25**) 237
4.1.23 3-[3-(4-Methoxyphenyl)-5-methyl-7-oxo-furo[3,2-*g*]chromen-6-yl]-*N*,*N*-dimethyl-propanamide (**Scaff10-26**)....239
4.1.24 3-[3-(4-Methoxyphenyl)-5-methyl-7-oxo-furo[3,2-*g*]chromen-6-yl]-*N*-(3-pyridyl)propanamide (**Scaff10-27**)....242
4.1.25 Ethyl 3-[4-methyl-2-oxo-7-[2-oxo-2-[4-(trifluoromethyl)phenyl]ethoxy]chromen-3-yl]propanoate (**Scaff10-28**)....245
4.1.26 3-[4-Methyl-2-oxo-7-[2-oxo-2-[4-(trifluoromethyl)phenyl]ethoxy]chromen-3-yl]propanoic acid (**Scaff10-29**)....248
4.1.27 3-[5-Methyl-7-oxo-3-[4-(trifluoromethyl)phenyl]furo[3,2-*g*]chromen-6-yl]propanoic acid (**Scaff10-30**)....251
4.1.28 Ethyl 3-[4-methyl-2-oxo-7-[2-oxo-2-(4-pyridyl)ethoxy]chromen-3-yl]propanoate (**Scaff10-31**)....253
4.1.29 3-[5-Methyl-7-oxo-3-(4-pyridyl)furo[3,2-*g*]chromen-6-yl]propanoic acid (**Scaff10-32**) 256
4.1.30 Ethyl 3-[7-[2-[4-(dimethylamino)phenyl]-2-oxo-ethoxy]-4-methyl-2-oxo-chromen-3-yl]propanoate (**Scaff10-34**)....258
4.1.31 3-[7-[2-[4-(Dimethylamino)phenyl]-2-oxo-ethoxy]-4-methyl-2-oxo-chromen-3-yl]propanoic acid (**Scaff10-35**)....261
4.1.32 3-(4-Methyl-2-oxo-7-[2-oxo-2-(4-phenoxyphenyl)ethoxy]chromen-3-yl)propanoic acid (**Scaff10-37**)....264
4.1.33 3-[5-Methyl-7-oxo-3-(4-phenoxyphenyl)furo[3,2-*g*]chromen-6-yl]propanoic acid (**Scaff10-38**)....266
4.1.34 Ethyl 3-[4-methyl-2-oxo-7-[2-oxo-2-(4-phenylphenyl)ethoxy]chromen-3-yl]propanoate (**Scaff10-39**)....269
4.1.35 3-[4-Methyl-2-oxo-7-[2-oxo-2-(4-phenylphenyl)ethoxy]chromen-3-yl]propanoic acid (**Scaff10-40**)....272
4.1.36 3-[5-Methyl-7-oxo-3-(4-phenylphenyl)furo[3,2-*g*]chromen-6-yl]propanoic acid (**Scaff10-41**)....275
4.1.37 Ethyl 3-(7-[2-(6-methoxy-2-naphthyl)-2-oxo-ethoxy]-4-methyl-2-oxo-chromen-3-yl)propanoate (**Scaff10-42**)....278
4.1.38 3-(7-[2-(6-Methoxy-2-naphthyl)-2-oxo-ethoxy]-4-methyl-2-oxo-chromen-3-yl)propanoic acid (**Scaff10-43**)....281
4.1.39 3-[7-Methoxy-6-[1-(6-methoxy-2-naphthyl)vinyl]-4-methyl-2-oxo-chromen-3-yl]propanoic acid (**Scaff10-44**)....284

4.1.40 3-[3-[6-(4-Fluorophenyl)-2-methyl-3-pyridyl]-5-methyl-7-oxo-furo[3,2-*g*]chromen-6-yl]propanoic acid (**Scaff10-45**) 287
4.1.41 5,6-Dimethyl-3-(4-phenylphenyl)furo[3,2-*g*]chromen-7-one (**Scaff10-46**) 290
4.1.42 3-(6-Methoxy-2-naphthyl)-5,6-dimethyl-furo[3,2-*g*]chromen-7-one (**Scaff10-47**) 293
4.1.43 Methyl 3-[3-(4-methoxyphenyl)-5-methyl-7-oxo-furo[3,2-*g*]chromen-6-yl]propanoate (**Scaff10-48**) 296
4.1.44 3-[3-(4-Methoxyphenyl)-5-methyl-7-oxo-furo[3,2-*g*]chromen-6yl]propanehydroxamic acid (**Scaff10-51**) 299
4.1.45 Ethyl 3-[7-[2-(4-hydroxyphenyl)-2-oxo-ethoxy]-4-methyl-2-oxo-chromen-3-yl]propanoate (**Scaff10-52**) 302
4.1.46 Ethyl 3-[7-[2-[4-[2-(4-hydroxyphenyl)-2-oxo-ethoxy]phenyl]-2-oxo-ethoxy]-4-methyl-2-oxo-chromen-3-yl]propanoate (**Scaff10-52d**) 305
4.1.47 Ethyl 3-(4-methyl-2-oxo-7-phenacyloxy-chromen-3-yl)propanoate (**Scaff10-56**).......308
4.1.48 3-(5-Methyl-7-oxo-3-phenyl-furo[3,2-*g*]chromen-6-yl)propanoic acid (**Scaff10-58**) ..311
4.1.49 Ethyl 3-[7-[(4-methoxyphenyl)methoxy]-4-methyl-2-oxo-chromen-3-yl]propanoate (**Scaff10-59**) 314
4.1.50 3-[7-[(4-Methoxyphenyl)methoxy]-4-methyl-2-oxo-chromen-3-yl]propanoic acid (**Scaff10-60**) 317
4.1.51 2-[3-[3-(4-Methoxyphenyl)-5-methyl-7-oxo-furo[3,2-*g*]chromen-6-yl]propanoylamino]acetic acid (**Scaff10-61**) 320
4.1.52 Ethyl 3-[7-[2-[4-(3-methoxyphenyl)phenyl]-2-oxo-ethoxy]-4-methyl-2-oxo-chromen-3-yl]propanoate (**Scaff10-62**) 323
4.1.53 3-[7-[2-[4-(3-Methoxyphenyl)phenyl]-2-oxo-ethoxy]-4-methyl-2-oxo-chromen-3-yl]propanoic acid (**Scaff10-63**) 326
4.1.54 3-[3-[4-(3-Methoxyphenyl)phenyl]-5-methyl-7-oxo-furo[3,2-*g*]chromen-6-yl]propanoic acid (**Scaff10-64**) 329

1 Results

1.1 Supplementary figures 1-5

Supplemental Figure 1

A

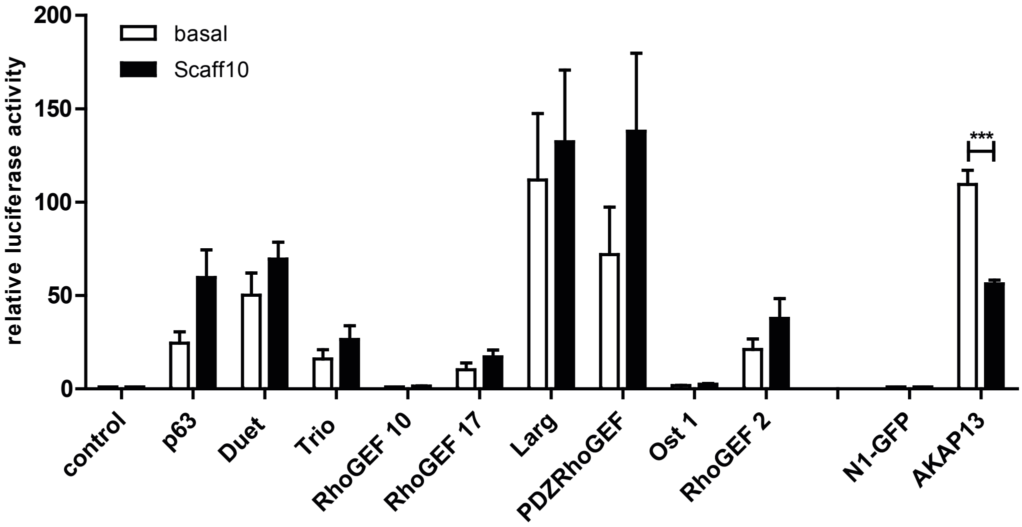

B

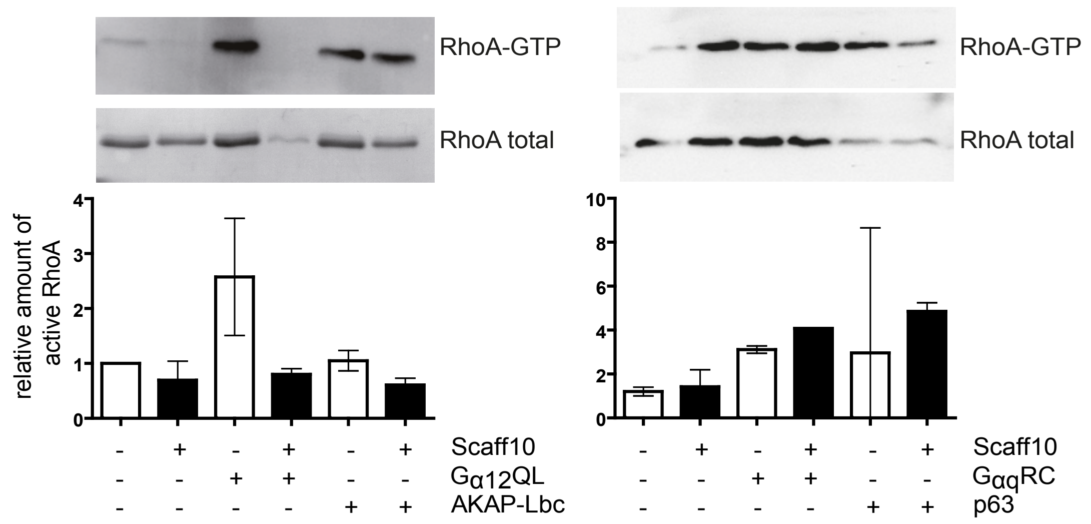

Supplemental Figure 1. Scaff10 specifically inhibits the Gα12-AKAP-Lbc-mediated activation of RhoA. (A) Full length AKAP-Lbc (AKAP13) and the indicated GEFs were transiently expressed in HEK293 cells. Upper panel: Scaff10 concentration-dependently inhibits the AKAP-Lbc-mediated stress response element-induced luciferase activity but at a concentration of 50 µM does not affect increases in luciferase activity induced by other GEFs. n=3; statistically significant differences were not found, as confirmed by statistical analysis using ANOVA. (B) Scaff10 inhibits Gα12- but not Gαq-mediated RhoA activation. The indicated Gα12 and Gαq-mutants were transiently co-expressed with AKAP-Lbc and p63RhoGEF in HEK293 cells as indicated. The cells were left untreated or incubated with Scaff10 (50 µM). The fraction of active RhoA was determined using Rhotekin pulldown assays as described [1]. Shown is one representative out of three experiments. All GEF constructs were in pCMV3a and Myc-tagged (30 ng per transfection). Expression and specificity of the assay for Rho were previously determined through inhibition of RhoA with C3 toxin [2-4]. Expression of AKAP-Lbc, LARG and PDZ-RhoGEF is shown in Figure 7 of the main manuscript.

Supplemental Figure 2

A

```
TCC AAA CAG CTG GAA GCA GAG TCT TGG AGT CGG ATA ATA GAC AGC AAG TTT CTA AAA CAG CAA AAG AAA GAT GTG GTC
                                              ↓   ↓
                                              C   C
AAA CGG CAA GAA GTA ATA TAT GAG TTG ATG CAG ACA GAG TTT CAT CAT GTC CGC ACT CTC AAG ATC ATG AGT GGT GTG
                  ↓   ↓
                  G   C
TAC AGC CAG GGG ATG ATG GCG GAT CTG CTT TTT GAG CAG CAG ATG GTA GAA AAG CTG TTC CCC TGT TTG GAT GAG CTG
ATC AGT ATC CAT AGC CAA TTC TTC CAG AGG ATT CTG GAG CGG AAG AAG GAG TCT CTG GTG GAT AAA AGT GAA AAG AAC
TTT CTC ATC AAG AGG ATA GGG GAT GTG CTT GTA AAT CAG TTT TCA GGT GAG AAT GCA GAA CGT TTA AAG AAG ACA TAT
                                                                                  ↓ ↓ ↓
                                                                                  G C G
GGC AAG TTT TGT GGG CAA CAT AAC CAG TCT GTA AAC TAC TTC AAA GAC CTT TAT GCC AAG GAT AAG CGT TTT CAA GCC
TTT GTA AAG AAG AAG ATG AGC AGT TCA GTT GTT AGA AGG CTT GGA ATT CCA GAG TGC ATA TTG CTT GTA ACT CAG CGG
ATT ACC AAG TAC CCA GTT TTA TTC CAA AGA ATA TTG CAG TGT ACC AAA GAC AAT GAA GTG GAG CAG GAA GAT CTA GCA
CAG TCC TTG AGC CTG GTG AAG GAT GTG ATT GGA GCT GTA GAC AGC AAA GTG GCA AGT TAT GAA AAG AAA GTG CGT CTC
AAT GAG ATT TAT ACA AAG ACA GAT AGC AAG TCA ATC ATG AGG ATG AAG AGT GGT CAG ATG TTT GCC AAG GAA GAT TTG
AAA CGG AAG AAG CTT GTA CGT GAT GGG AGT GTG TTT CTG AAG AAT GCA GCA GGA AGG TTG AAA GAG GTT CAA GCA GTT
                      ↓   ↓
                      G   G
CTT CTC ACT GAC ATT TTA GTT TTC CTT CAA GAA AAA GAC CAG AAG TAC ATC TTT GCA TCA TTG GAC CAG AAG TCA ACA
GTG ATC TCT TTA AAG AAG CTG ATT GTG AGA GAA GTG GCA CAT GAG GAG AAA GGT TTA TTC CTG ATC AGC ATG GGG ATG
ACA GAT CCA GAG ATG GTA GAA GTC CAT GCC AGC TCC AAA GAG GAA CGA AAC AGC TGG ATT CAG ATC ATT CAG GAC ACA
```

B

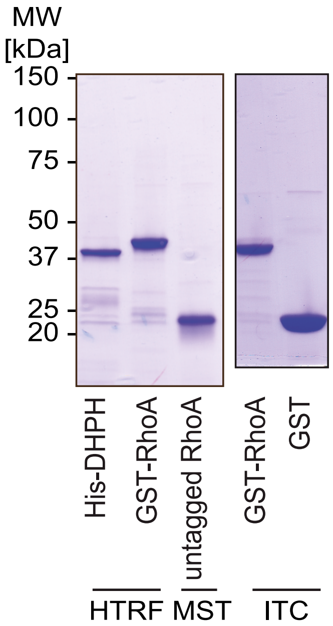

Supplemental Figure 2. (A) The sequence of the AKAP-Lbc/DHPH domain (5,914-7,005 bp, NCBI accession number CCDS32320.1) was mutated in order to remove rare codons and genetically fused with GFP and a His_6 tag. Substituted base pairs are indicated in red. (B) Purified recombinant proteins for subsequent assays. SDS-PAGE followed by Coomassie staining: His6-AKAP-Lbc/DHPH (46 kDa) and GST-RhoA (48 kDa) were used for the HTRF assay and ITC; untagged RhoA (22 kDa) and GST were used for ITC (28 kDa). 2 µg of each protein were loaded.

Supplemental Figure 3

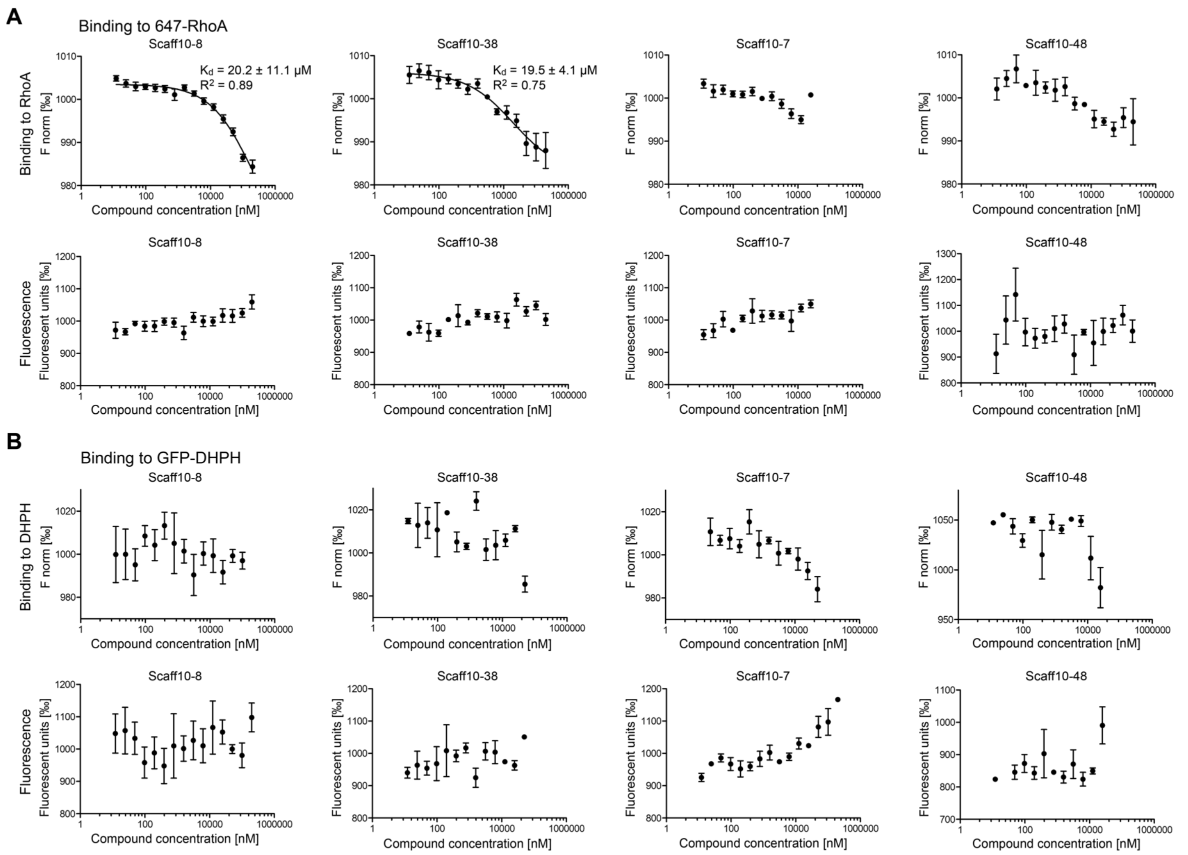

Supplemental Figure 3. Scaff10 derivatives, Scaff10-8 and Scaff10-38 inhibiting the AKAP-Lbc-RhoA interaction in our HTRF assay bound RhoA, not the AKAP-Lbc/DHPH domain. Results of MST assays. (A) Upper panel: The concentration of fluorescent 647-RhoA remained constant whereas Scaff10-8, Scaff10-38, Scaff10-7 and Scaff10-48 (ligands) were added in increasing concentrations. The Kd values for the binding of Scaff10-8 and Scaff10-38 to RhoA are 20.2 ± 11.1 µM and 19.5 ± 4.1 µM, respectively. Lower panel: Corresponding values of fluorescence to upper panel. (B) Upper panel: The concentration of fluorescent GFP-AKAP-Lbc/DHPH remained constant whereas Scaff10-8, Scaff10-38, Scaff10-7 and Scaff10-48 (ligands) were added in increasing concentrations. Lower panel: Corresponding values of fluorescence to upper panel. (B) F norm = normalised fluorescence (fluoresence steady state/fluorescence initial state) indicated in ‰. Compound concentration [nM] refers to the appropriate ligand. n = 3-5. Mean ± SEM is plotted. Kd values are indicated ± SEM. R^2 indicates the coefficient of determination. The results of the Scaff10-8 and Scaff10-7 derivatives are shown in the main manuscript and are here included for direct comparison.

Supplemental Figure 4

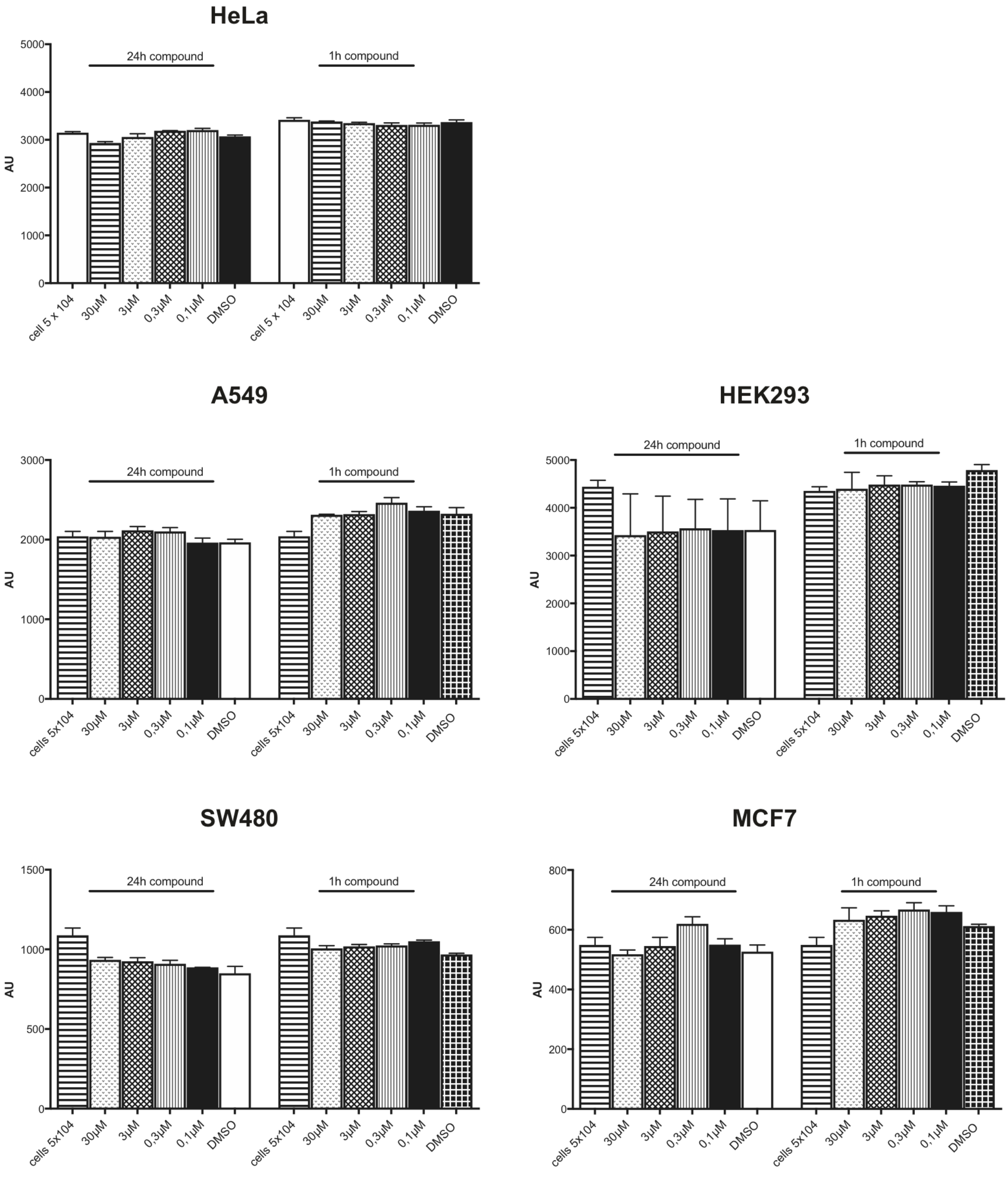

Supplemental Figure 4. Scaff10-8 does not affect viability of cells. The viability of the indicated cells was determined using a CellTiter-Glo®Luminescent Cell Viability Assay (Promega, Wisconsin, USA) according to the manufacturer's instructions. The assay is based on the quantification of ATP as an indicator of metabolically active cells. The cells were seeded, grown for 24 h and treated with the different concentrations of Scaff 10-8. Viability was assessed after 1 and 24 h incubation with Scaff10-8 in the indicated concentrations. An equal volume of CellTiter-Glo® Reagent was added, plates were shaken and luminescence monitored over time with the plates held at 22 °C. Mono-oxygenation of luciferin is catalyzed by luciferase in the presence of Mg^{2+}, ATP and molecular oxygen. DMSO was used as a control. Relative luminescent signals (RLU) was measured and normalized to untreated cells, n = 3. Statistically significant differences were determined applying one-way ANOVA with posthoc Bonferroni. Mean ± SD is plotted.

Supplemental Figure 5

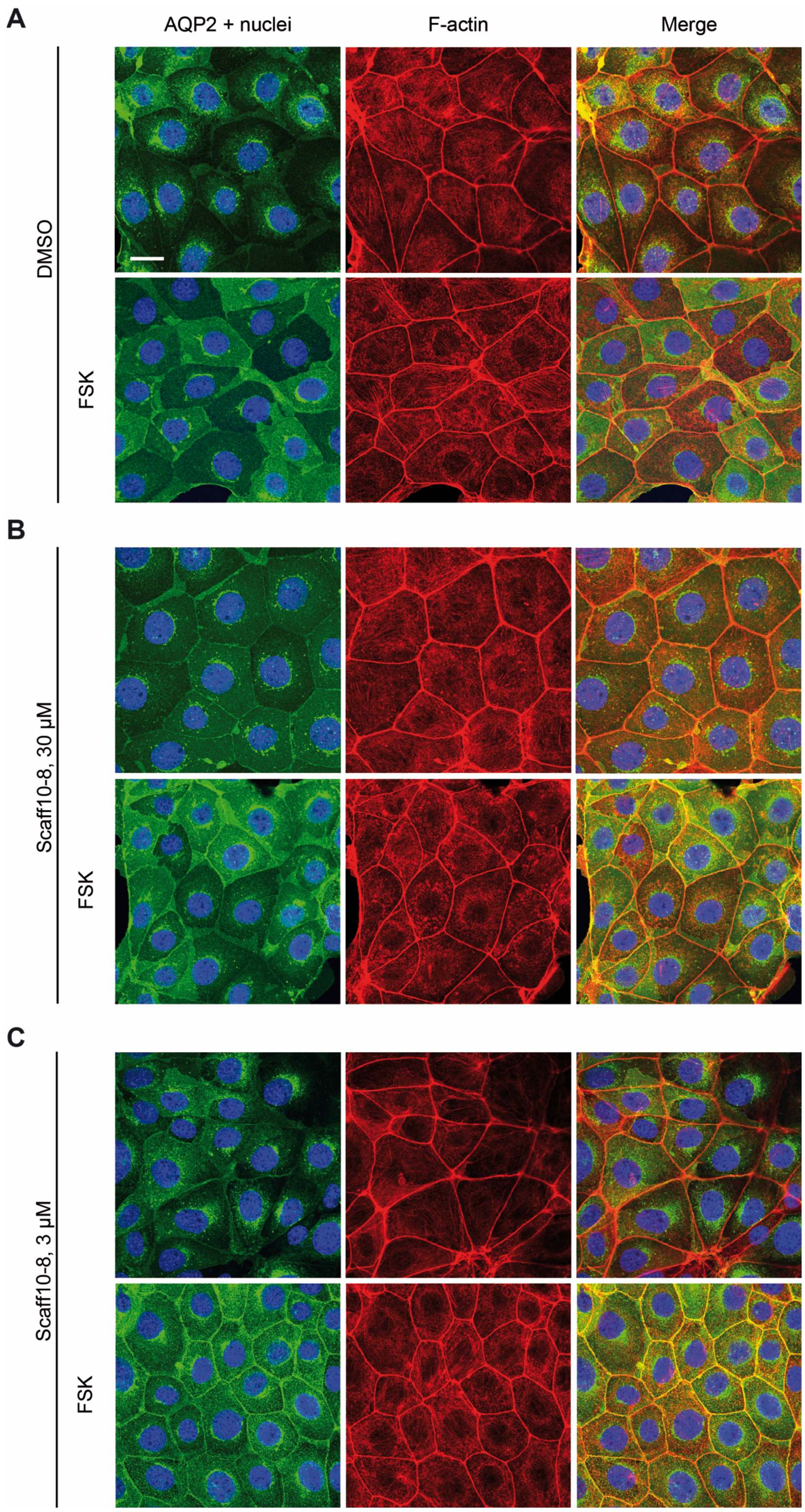

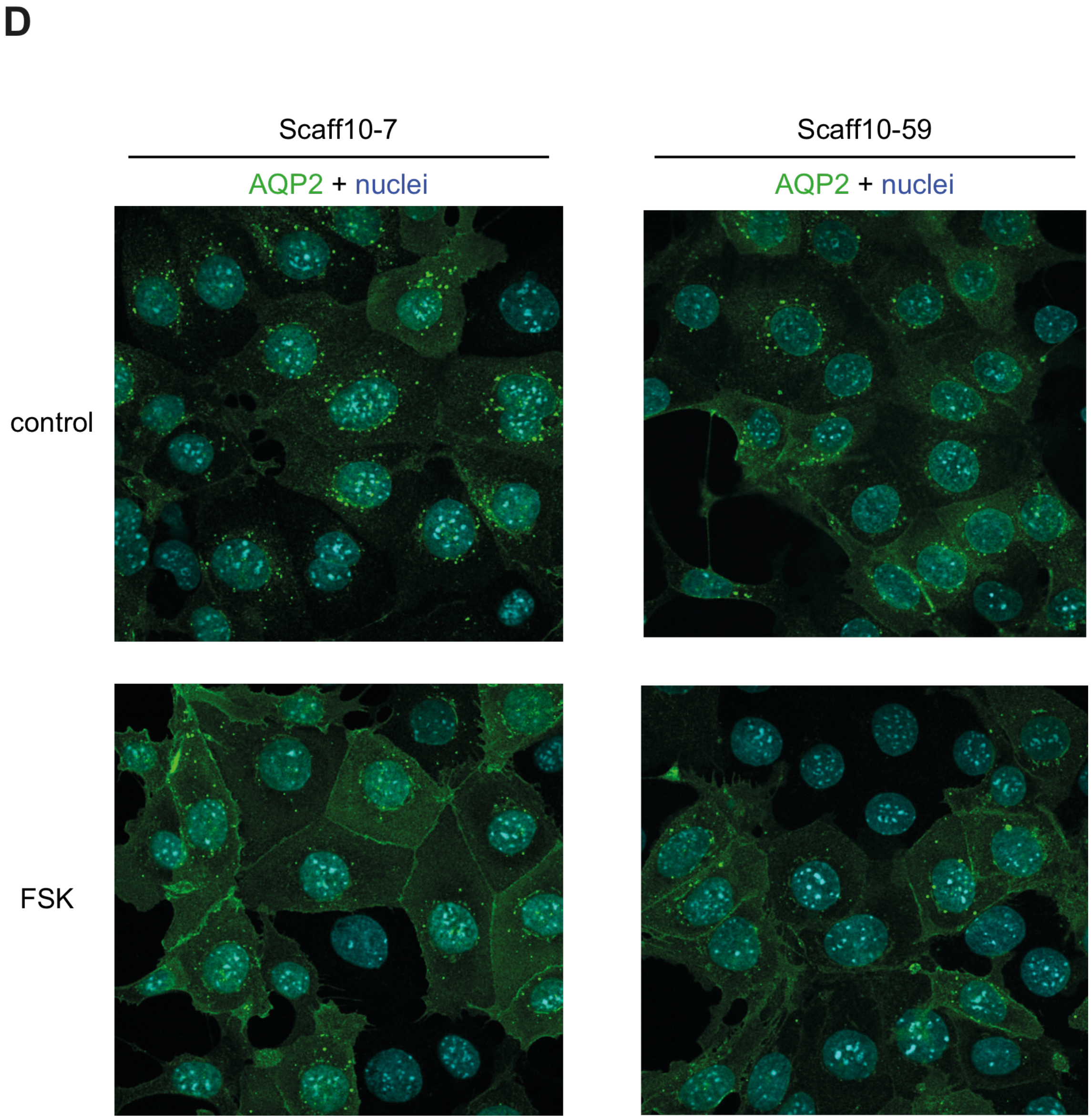

Supplemental Figure 5. Scaff10-8 but not Scaff10-7 or Scaff10-59 induced the translocation of AQP2 to the plasma membrane of MCD4 cells independent of cAMP elevation. MCD4 cells were left untreated (A) or incubated with 30 (B) or 3 µM (C) Scaff10-8 and stimulated with forskolin (FSK). DMSO was used as a control (A). As negative controls, the cells were incubated with Scaff10-7 and Scaff10-59 (30 µM each), which were inactive in our HTRF assay. AQP2 stained in green, nuclei stained in blue, F-actin stained in red. Scale bar indicates 20 µm. Representative images are shown. n = 3.

1.2 Supplementary Tables

1.2.1 Suppl. Tab. 1

Comp_ID	z_score	ResidualPerce	PNG_Image	Selected_for_Validation
27239	-359.1	-5460.0	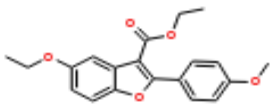	
28204	-374.3	-4229.0	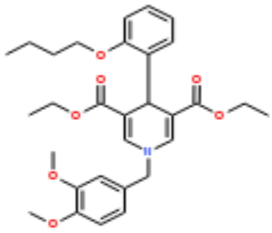	
27243	-275.6	-4168.0	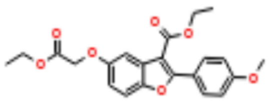	
29986	-454.7	-4017.0	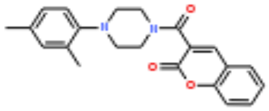	
27241	-245.5	-3702.0	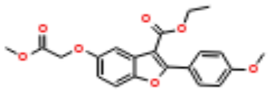	
28166	-308.9	-3471.0	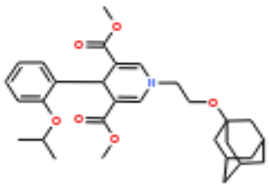	
30293	-351.8	-3083.0	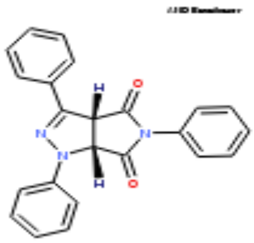	

34149	-228.5	-2785.0	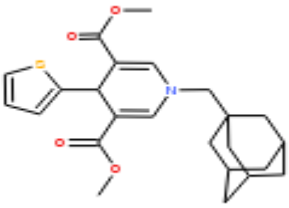		
29988	-302.5	-2641.0	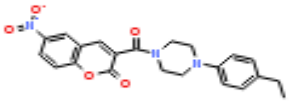		
28200	-199.4	-2205.0	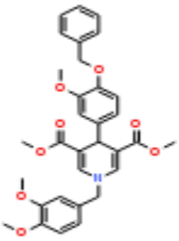		
27245	-143.0	-2117.0	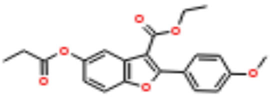		yes
31865	-186.1	-1997.0	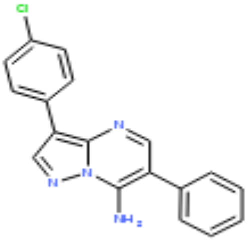		
32682	-159.5	-1463.0	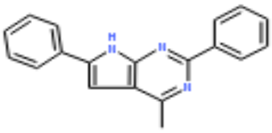		
30988	-136.3	-1365.0	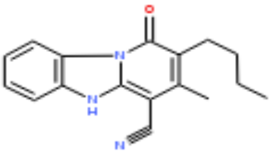		

36680	-93.1	-1349.0	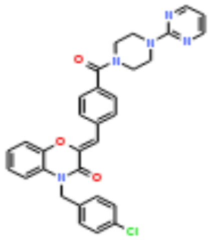		
38123	-103.0	-1346.0	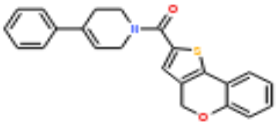		
28144	-123.4	-1325.0	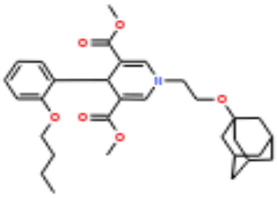		
29960	-147.7	-1243.0	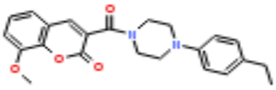		
29953	-146.6	-1233.0	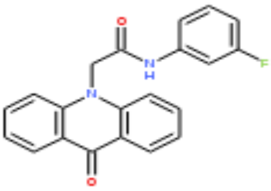		yes
29964	-144.6	-1215.0	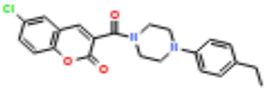		
27205	-82.1	-1174.0	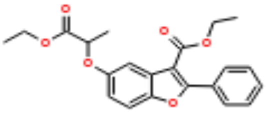		

27213	-78.3	-1116.0	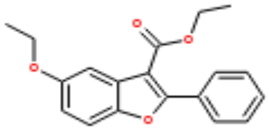		
31859	-105.7	-1094.0	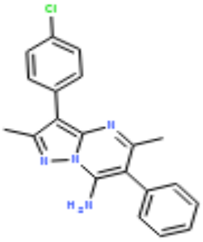		
28751	-123.3	-1034.0	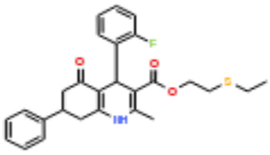		
32254	-117.2	-987.0	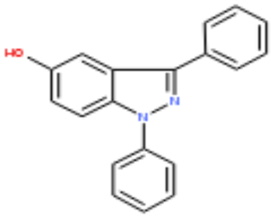		
32883	-105.6	-935.0	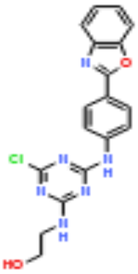		
28687	-111.9	-929.0	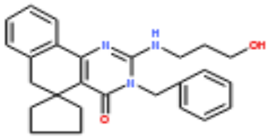		
33542	-69.4	-886.0	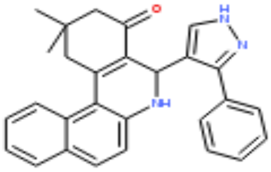		

26490	-128.5	-876.0	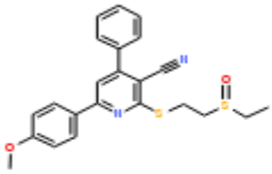		
31891	-83.7	-848.0	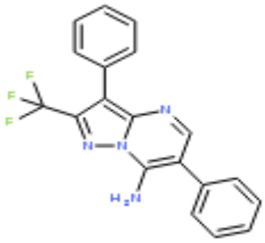		
27235	-60.7	-843.0	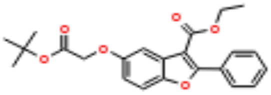		
30677	-100.1	-813.0	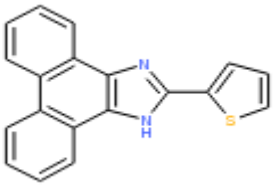		
28170	-76.8	-786.0	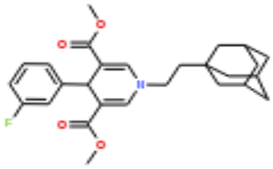		
35955	-49.0	-755.0	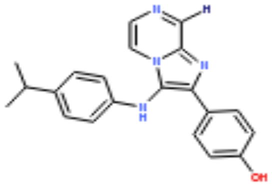		yes
28683	-92.5	-752.0	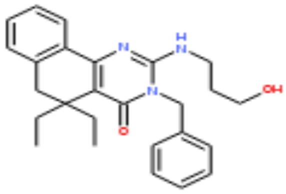		

28172	-72.4	-736.0	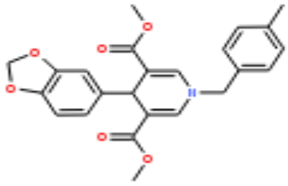		
102315	-110.9	-728.0	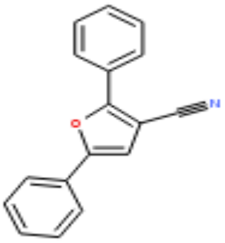		
30839	-86.4	-688.0	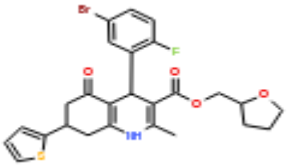		
30867	-79.5	-626.0	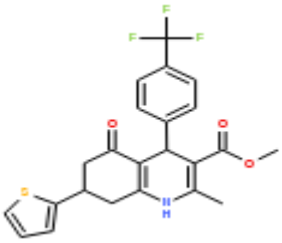		
35899	-41.4	-624.0	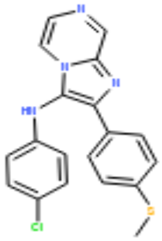		yes
27237	-46.4	-622.0	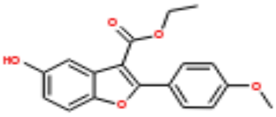		
32767	-71.6	-602.0	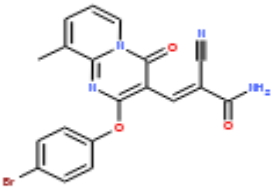		

31863	-61.7	-600.0	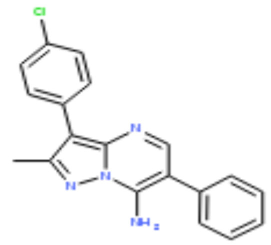		
27207	-44.6	-595.0	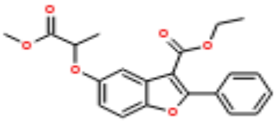		
36841	-44.1	-588.0	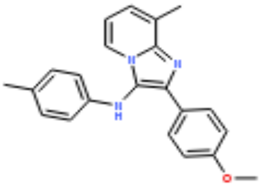		
34462	-52.6	-587.0	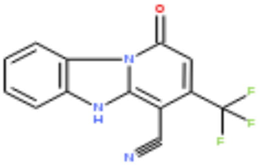		
28176	-58.9	-580.0	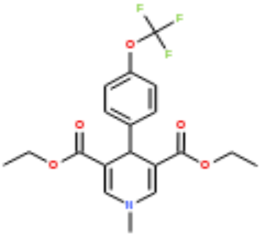		
31297	-58.5	-580.0	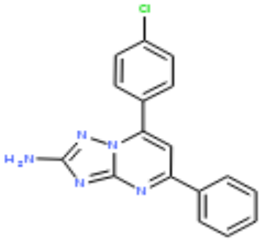		
26732	-44.3	-561.0	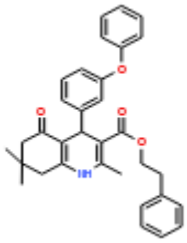		

38121	-46.9	-560.0	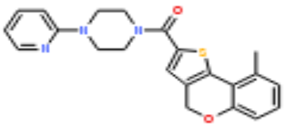	
28855	-58.9	-544.0	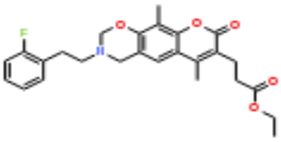	
28196	-53.6	-517.0	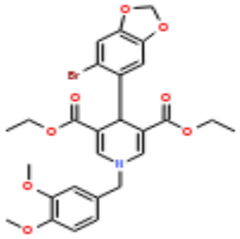	
36648	-39.4	-516.0	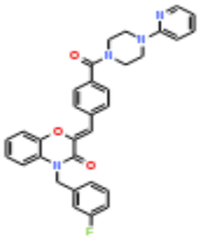	
30195	-67.2	-515.0	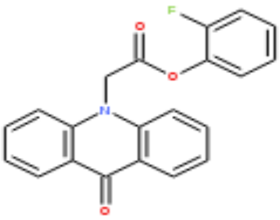	
27853	-46.6	-514.0	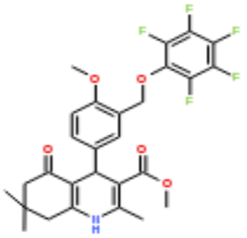	
30701	-66.2	-506.0	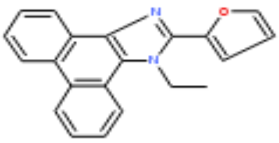	

35860	-34.5	-504.0	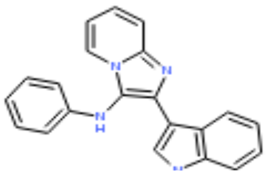		
25001	-57.2	-499.0	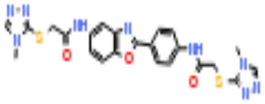		
30865	-63.3	-480.0	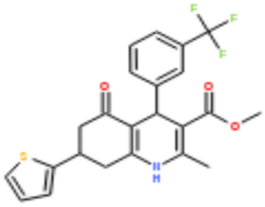		
36846	-35.7	-458.0	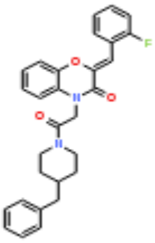		
28777	-60.2	-457.0	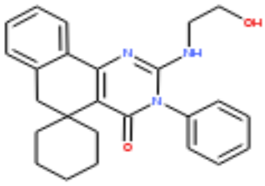		
27572	-41.2	-451.0	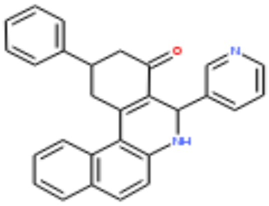		
36265	-31.4	-442.0	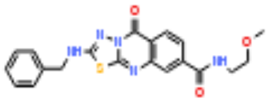		

28407	-45.0	-419.0	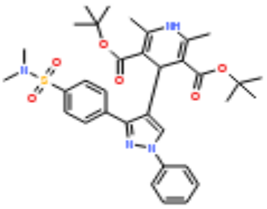		
39624	-59.1	-411.0	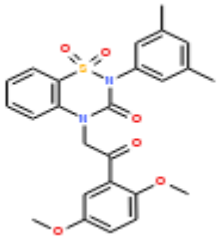		
36650	-32.2	-403.0	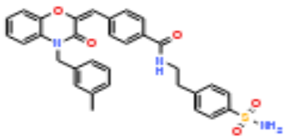		
39465	-58.0	-402.0	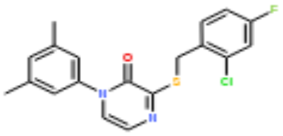		
26756	-33.3	-399.0	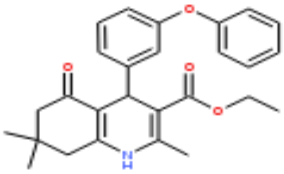		
101618	-53.8	-377.0	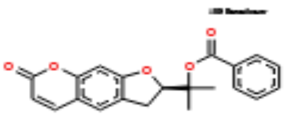		yes
33896	-36.3	-376.0	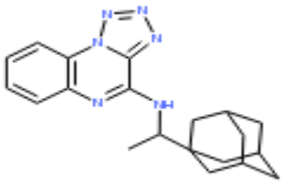		

27201	-30.2	-371.0	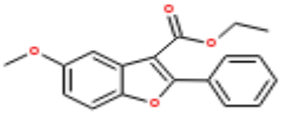		
36466	-27.2	-371.0	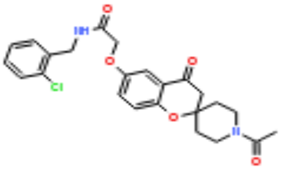		yes
36330	-26.6	-361.0	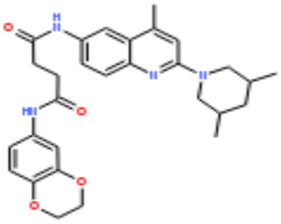		
28853	-41.7	-357.0	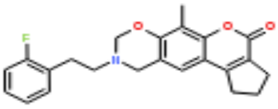		
35915	-26.1	-357.0	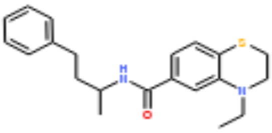		
29962	-49.1	-352.0	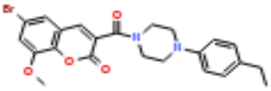		
33140	-29.3	-349.0	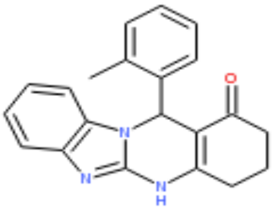		

36472	-25.2	-336.0	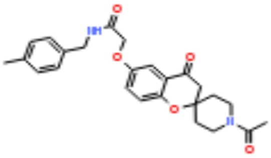		yes
27849	-32.7	-333.0	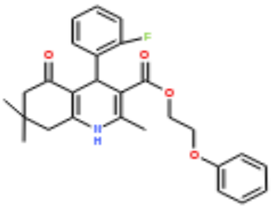		
102327	-57.9	-333.0	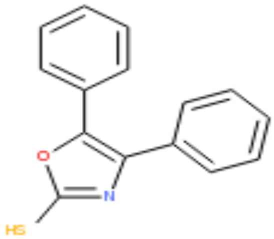		
30024	-45.8	-321.0	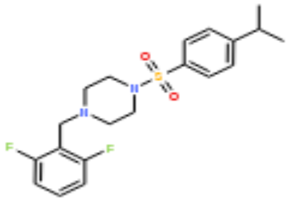		
30329	-46.0	-321.0	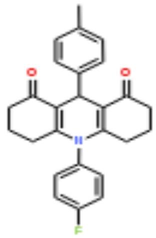		
29852	-46.6	-319.0	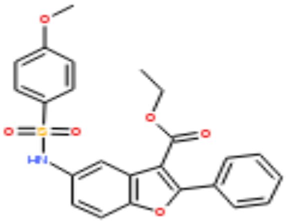		
35080	-28.4	-314.0	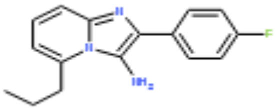		

31240	-37.9	-310.0	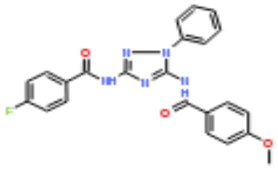		
31102	-37.8	-309.0	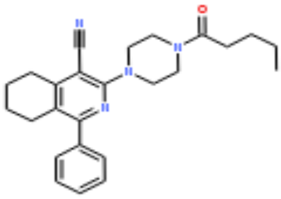		
24501	-37.2	-300.0	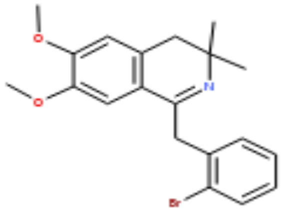		
26760	-26.2	-295.0	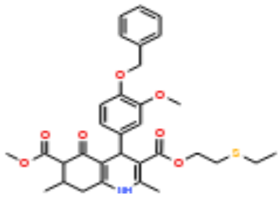		yes
24997	-37.3	-291.0	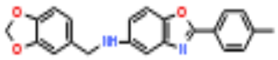		
35897	-22.1	-289.0	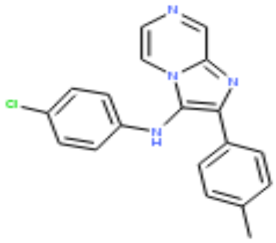		yes
30703	-41.8	-286.0	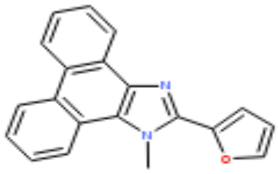		

28479	-41.2	-283.0	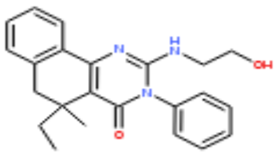		
27177	-24.1	-278.0	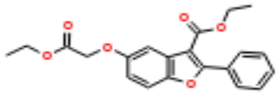		
27883	-28.3	-277.0	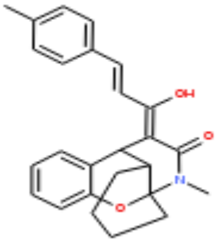		
38629	-26.1	-277.0	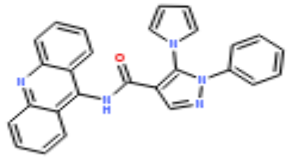		
25183	-35.9	-276.0	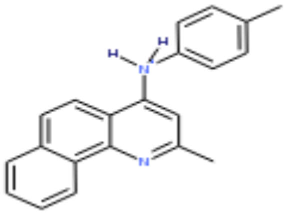		
28477	-40.5	-276.0	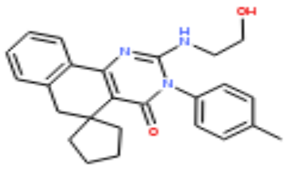		
32548	-39.1	-265.0	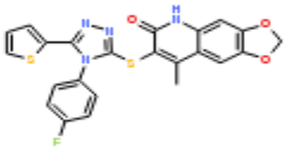		

100450	-49.9	-262.0	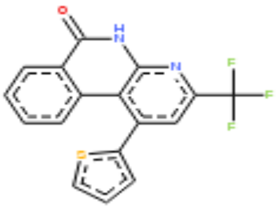		
27881	-27.0	-259.0	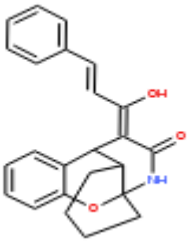		
31259	-32.9	-257.0	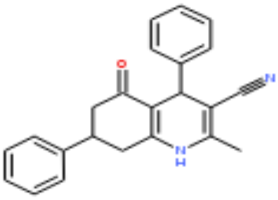		
36373	-20.4	-256.0	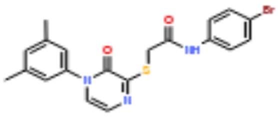		
35925	-20.2	-255.0	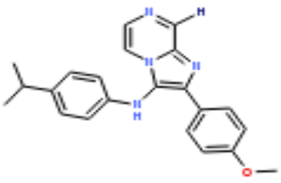		yes
36418	-20.0	-248.0	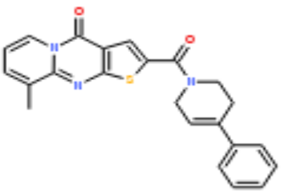		
35904	-19.6	-245.0	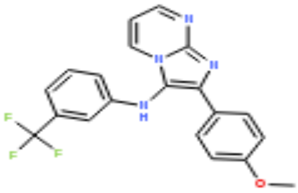		

27179	-21.9	-244.0	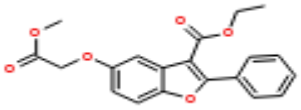	
33238	-22.4	-244.0	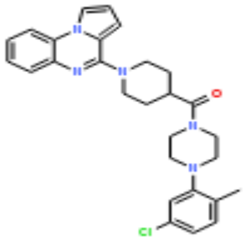	
29854	-37.9	-242.0	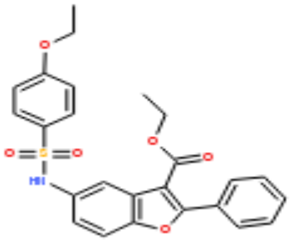	
39467	-38.5	-234.0	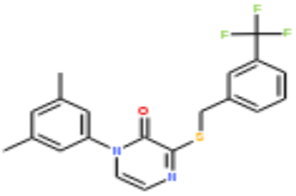	
24909	-30.7	-232.0	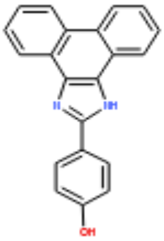	
28749	-35.0	-226.0	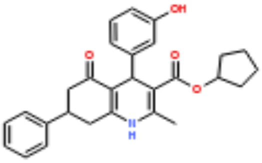	
31418	-27.7	-225.0	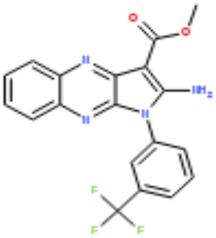	

27501	-23.5	-218.0	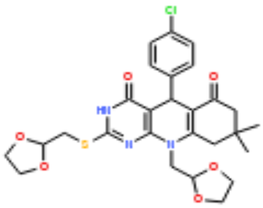		
36134	-18.0	-217.0	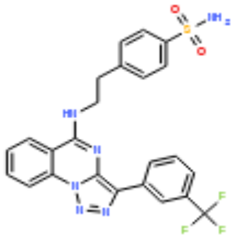		
33892	-24.0	-216.0	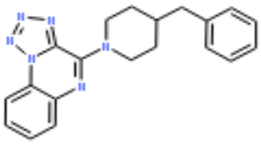		
31517	-26.6	-212.0	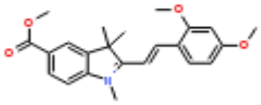		
27523	-23.0	-210.0	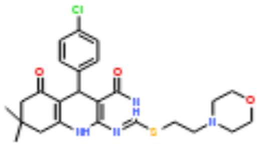		
37129	-20.0	-204.0	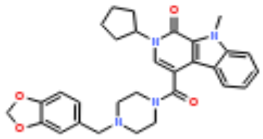		
31476	-25.7	-202.0	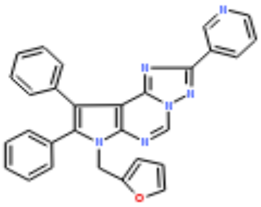		

25335	-34.7	-199.0	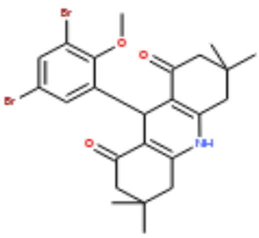	
28698	-31.4	-193.0	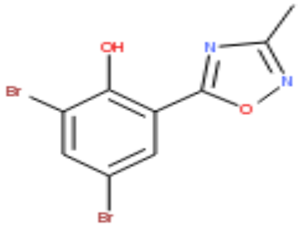	
36332	-16.7	-192.0	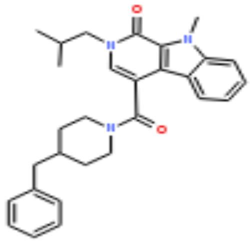	
26901	-18.9	-190.0	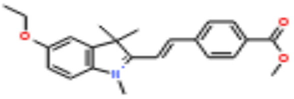	yes
35867	-16.4	-189.0	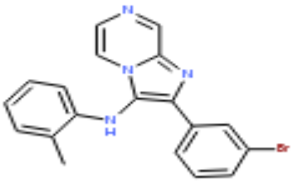	yes
36654	-18.4	-189.0	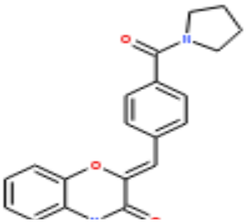	
37406	-17.6	-189.0	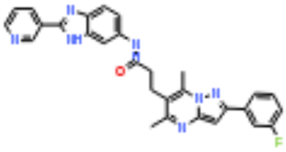	

30107	-31.1	-188.0	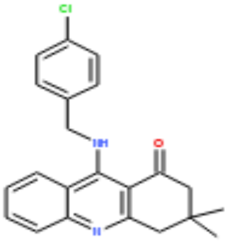		
36760	-17.9	-183.0	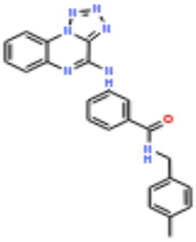		
30814	-30.4	-182.0	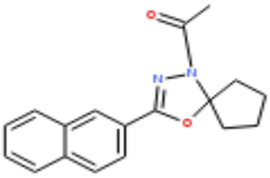		
32399	-30.0	-181.0	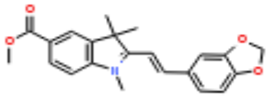		
34738	-21.2	-180.0	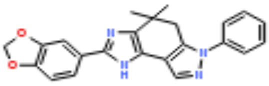		
24526	-25.6	-178.0	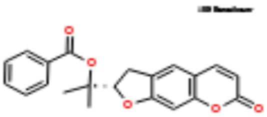		yes
24843	-25.6	-178.0	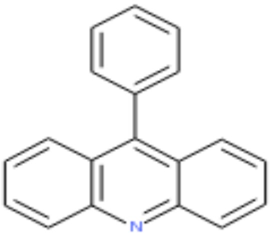		

26888	-17.9	-175.0	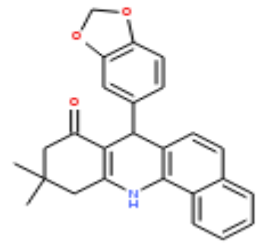		
28194	-23.5	-170.0	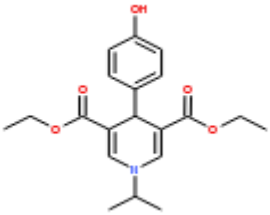		
33240	-17.5	-170.0	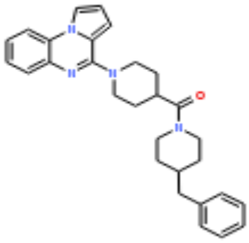		
31359	-22.9	-169.0	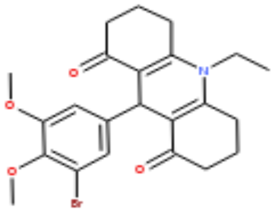		yes
34937	-18.4	-169.0	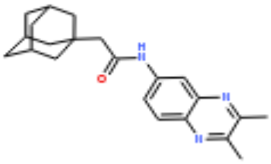		
28779	-28.3	-165.0	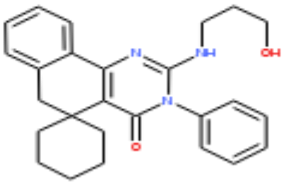		
26572	-33.3	-158.0	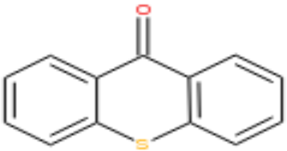		

29850	-28.5	-158.0	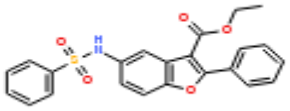		
36674	-16.2	-156.0	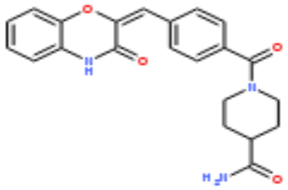		
31885	-22.1	-155.0	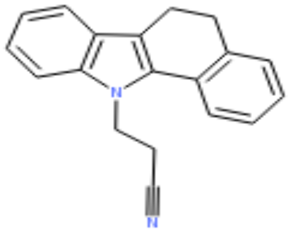		
38324	-17.6	-155.0	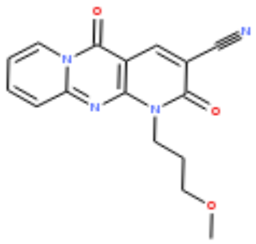		
24070	-22.9	-154.0	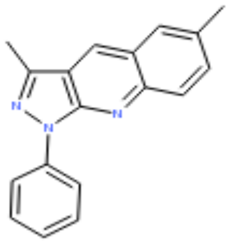		
31324	-21.6	-154.0	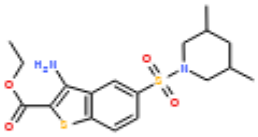		
30085	-26.7	-149.0	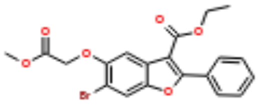		yes

35998	-13.8	-144.0	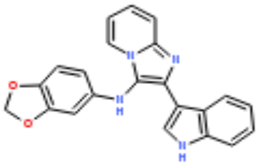		
101117	-23.1	-132.0	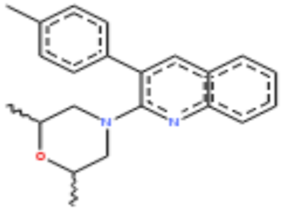		
39461	-26.4	-130.0	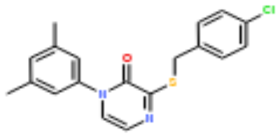		
31238	-20.9	-128.0	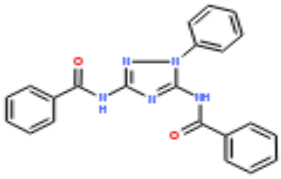		
32363	-24.0	-127.0	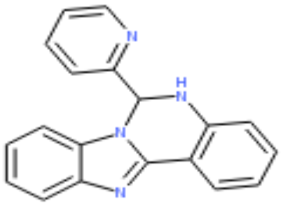		
33809	-16.9	-124.0	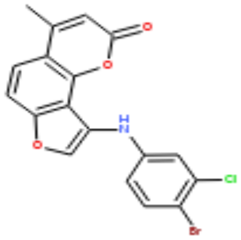		
38438	-15.5	-124.0	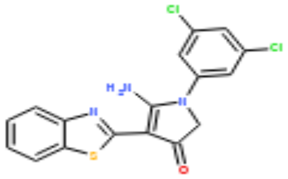		

102153	-29.8	-124.0	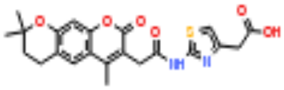		
31430	-18.7	-121.0	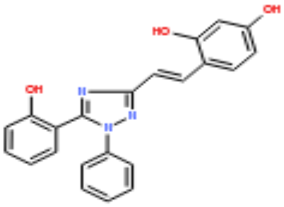		
25329	-25.2	-120.0	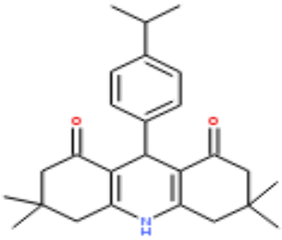		yes
34892	-15.0	-120.0	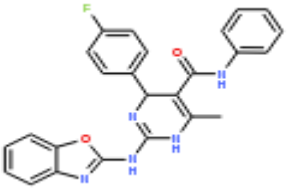		
27704	-15.9	-116.0	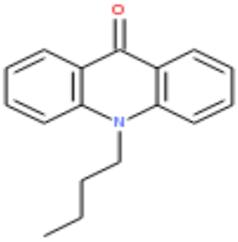		
30063	-23.0	-115.0	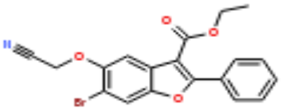		yes
31204	-19.7	-115.0	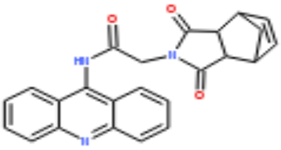		

24371	-19.5	-113.0	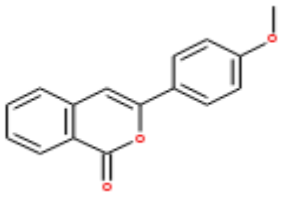		
38651	-14.6	-112.0	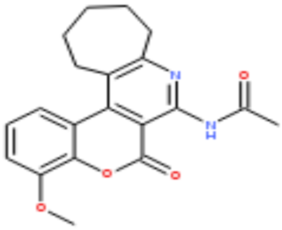		
27399	-15.5	-111.0	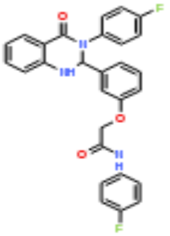		
34551	-15.8	-111.0	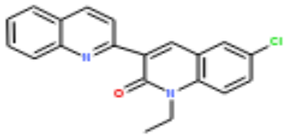		
101125	-21.0	-111.0	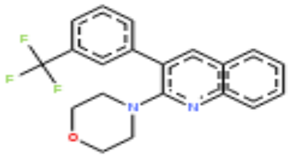		
29856	-23.2	-110.0	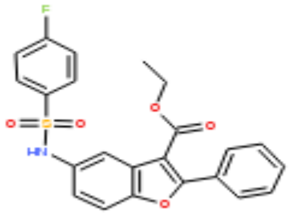		
24034	-18.5	-106.0	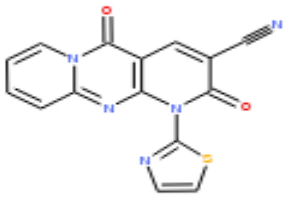		

35913	-11.6	-106.0	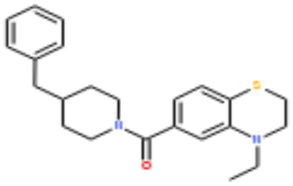		
28137	-17.8	-104.0	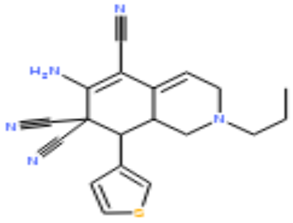		
38028	-14.2	-101.0	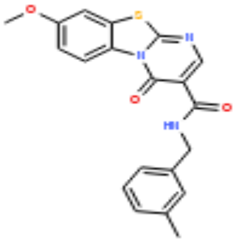		
36502	-11.2	-99.0	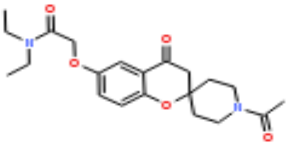		
30986	-18.1	-98.0	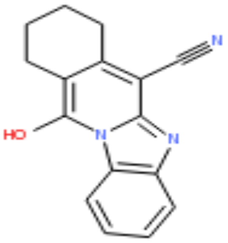		
25792	-23.4	-96.0	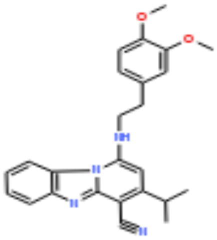		yes
35909	-11.0	-96.0	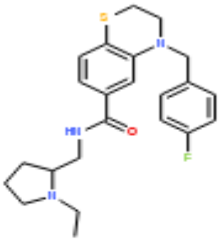		yes

30052	-20.8	-95.0	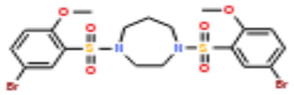		
39463	-22.4	-95.0	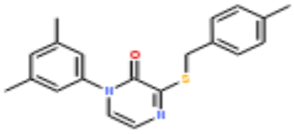		
38649	-13.4	-94.0	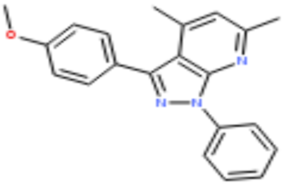		
30833	-20.5	-93.0	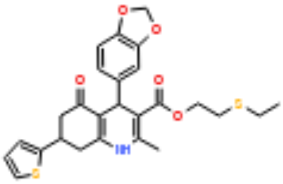		
27608	-14.0	-92.0	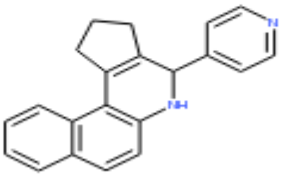		
31530	-16.2	-92.0	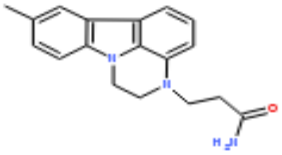		
36538	-10.8	-92.0	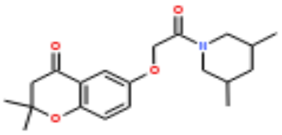		yes

32751	-19.5	-91.0	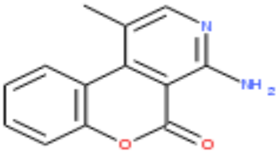		
30022	-20.2	-90.0	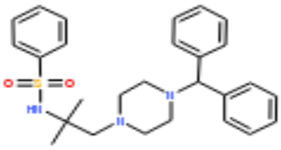		
36263	-10.6	-88.0	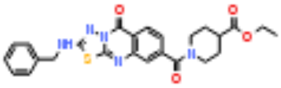		
36267	-10.5	-87.0	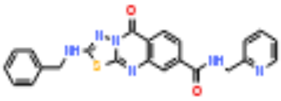		
36415	-10.5	-87.0	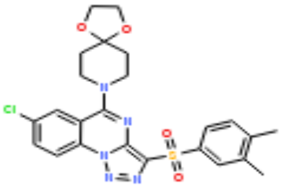		
24405	-16.9	-86.0	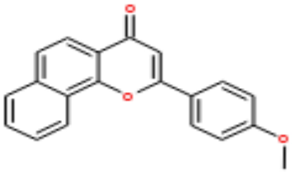		
30721	-19.7	-86.0	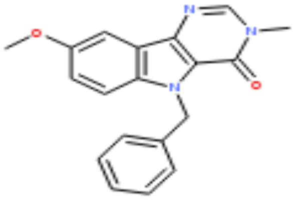		

31882	-15.9	-86.0	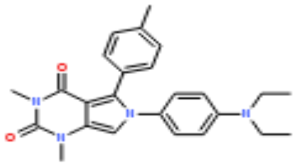	
38387	-12.8	-85.0	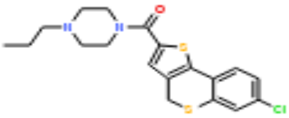	
26570	-23.5	-84.0	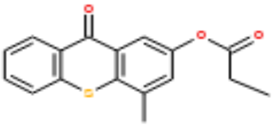	
101188	-18.2	-83.0	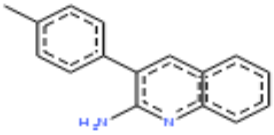	
102136	-24.3	-83.0	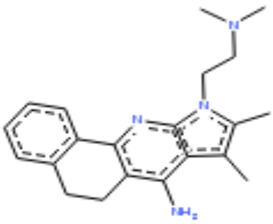	
34713	-13.6	-82.0	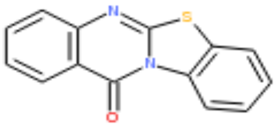	
36540	-10.2	-82.0	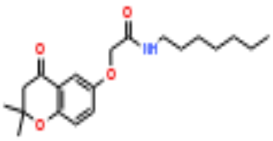	yes

38355	-12.6	-82.0	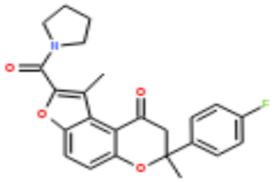		yes
37846	-13.3	-78.0	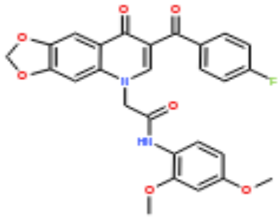		
31752	-15.1	-77.0	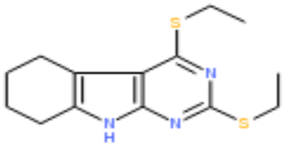		
40186	-20.8	-77.0	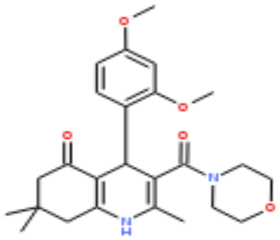		
35917	-9.8	-76.0	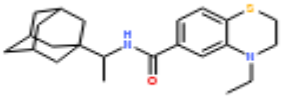		
101109	-17.5	-76.0	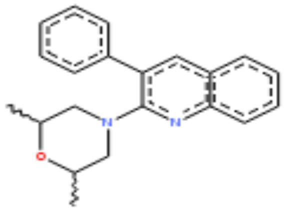		
39501	-20.0	-75.0	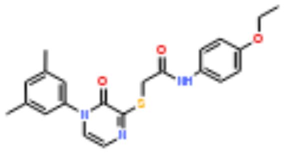		

35905	-9.8	-74.0	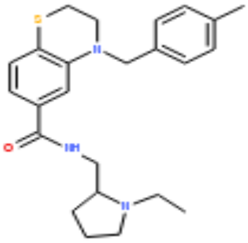		yes
35937	-9.5	-70.0	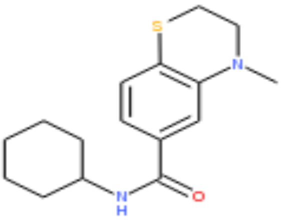		
37165	-11.0	-69.0	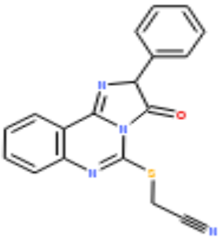		
29966	-17.7	-68.0	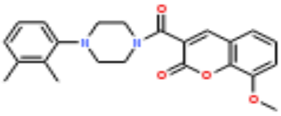		
32593	-17.6	-68.0	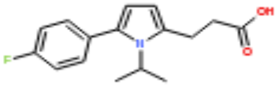		
32988	-17.1	-68.0	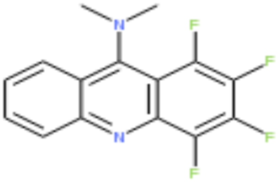		
34297	-12.7	-68.0	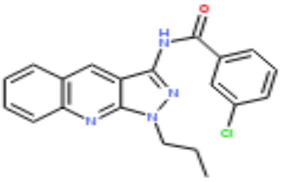		

36890	-10.5	-66.0	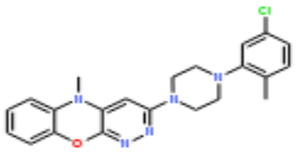		
36500	-9.2	-64.0	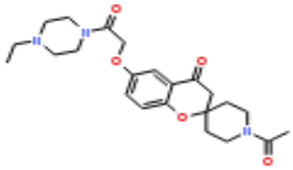		
36405	-9.1	-63.0	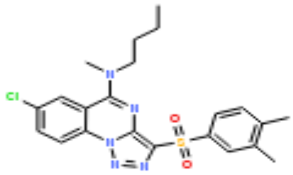		
38030	-11.4	-63.0	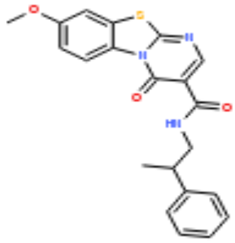		
30083	-17.1	-62.0	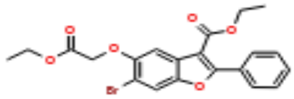		yes
23953	-14.4	-61.0	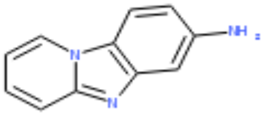		
36181	-9.0	-61.0	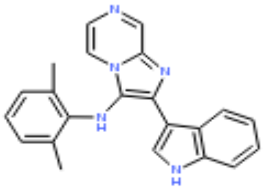		yes

36026	-8.9	-60.0	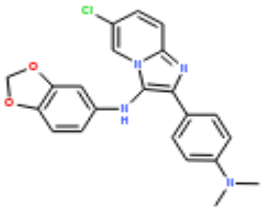		
31142	-14.5	-59.0	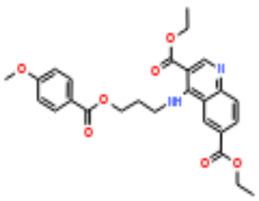		
25950	-18.6	-57.0	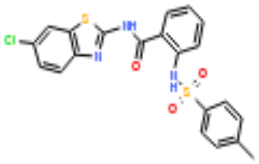		
29052	-14.2	-57.0	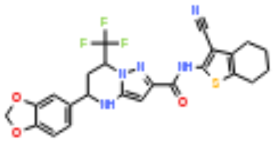		
36480	-8.7	-56.0	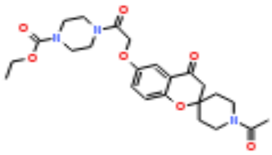		
26619	-19.5	-54.0	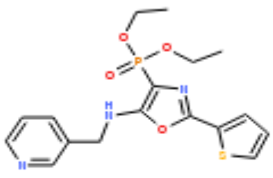		
27584	-11.2	-54.0	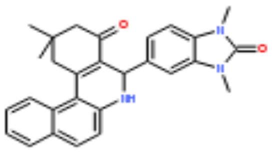		

31505	-12.8	-54.0	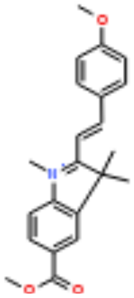		
26571	-19.4	-53.0	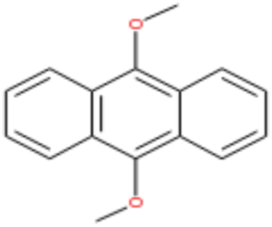		
26897	-9.6	-53.0	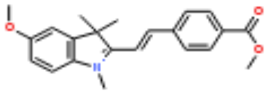		yes
27855	-11.1	-53.0	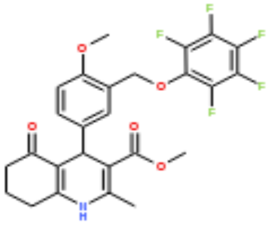		
28719	-16.1	-53.0	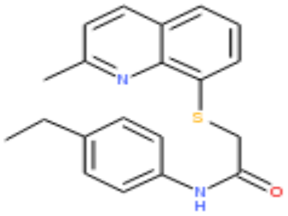		
26920	-9.6	-52.0	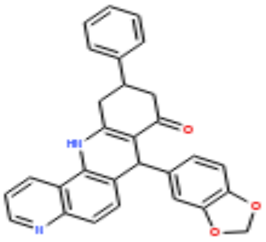		
29916	-16.0	-52.0	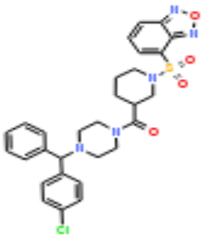		

102243	-20.3	-52.0	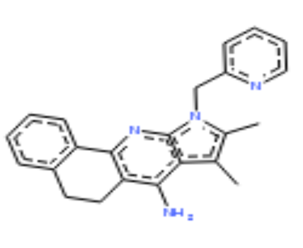	
26623	-19.1	-51.0	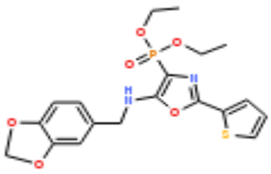	
26758	-9.3	-48.0	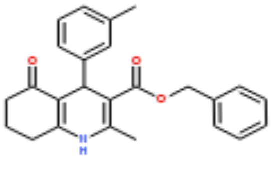	
30198	-15.2	-45.0	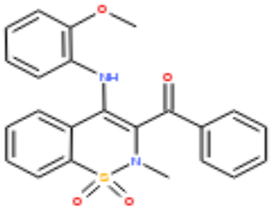	
33209	-9.2	-44.0	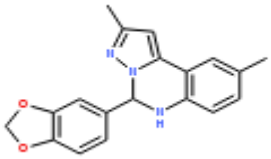	
29939	-15.0	-43.0	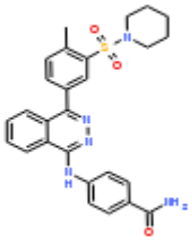	
36498	-7.8	-42.0	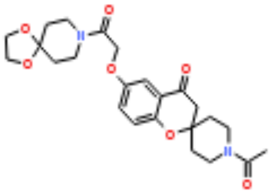	

28668	-14.6	-39.0	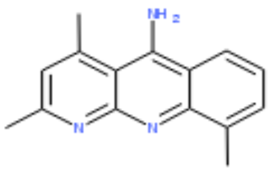	
31892	-11.8	-39.0	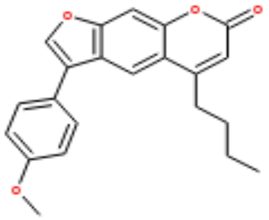	yes
35895	-7.7	-39.0	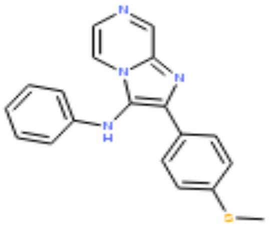	yes
24911	-12.5	-38.0	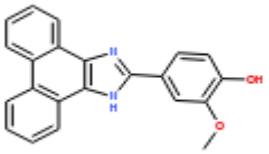	
36051	-7.7	-38.0	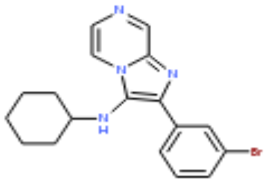	yes
31738	-11.6	-37.0	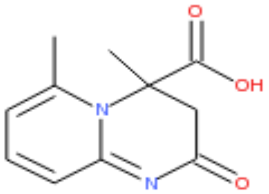	
34288	-10.2	-37.0	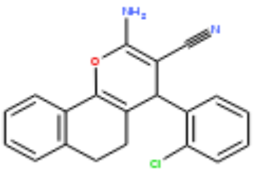	

24522	-12.2	-35.0	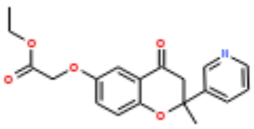		
31138	-12.2	-34.0	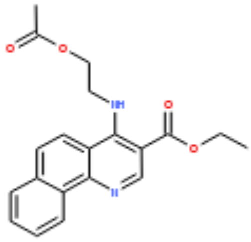		
34861	-9.1	-34.0	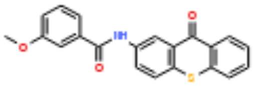		
36532	-7.4	-34.0	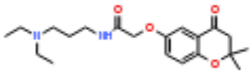		
38331	-9.3	-34.0	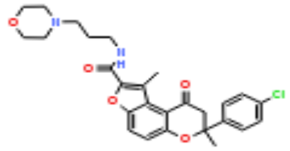		yes
101699	-15.2	-34.0	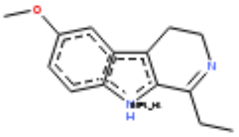		
27998	-9.6	-33.0	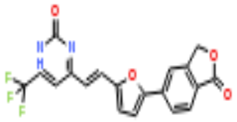		

35891	-7.4	-33.0	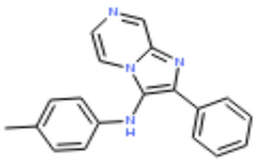		yes
26909	-8.2	-32.0	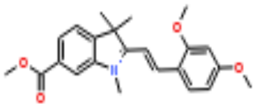		yes
27209	-8.2	-32.0	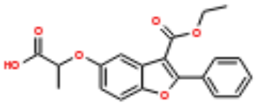		
26464	-16.5	-31.0	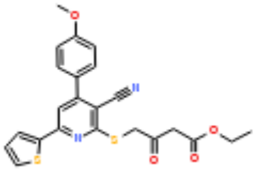		
33184	-8.1	-28.0	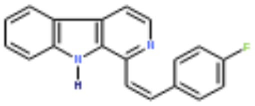		
30782	-13.2	-27.0	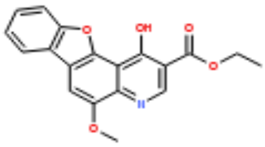		
101344	-12.5	-25.0	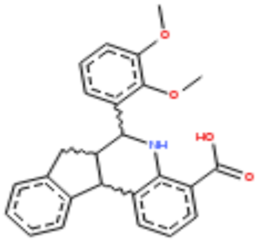		yes

101544	-14.3	-25.0	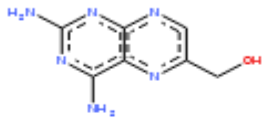		
26568	-15.6	-24.0	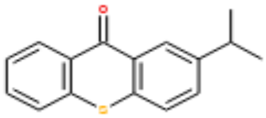		
39036	-15.0	-24.0	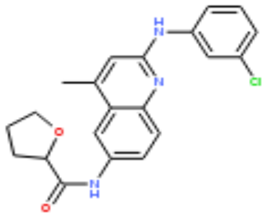		
27335	-7.7	-23.0	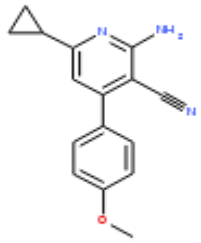		
28543	-12.7	-22.0	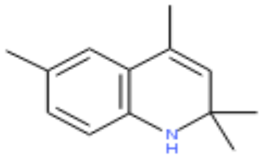		
35486	-8.1	-22.0	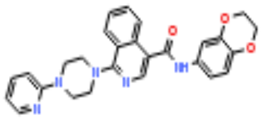		
27521	-8.7	-21.0	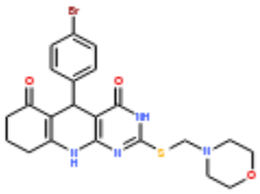		

25365	-13.3	-20.0	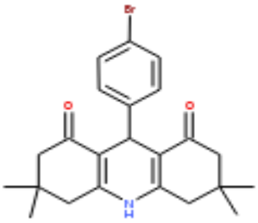		yes
30056	-12.4	-20.0	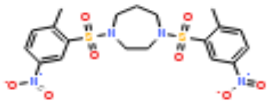		
28664	-12.4	-19.0	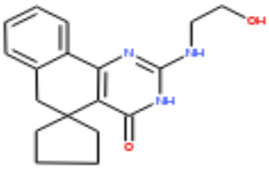		
35047	-8.0	-18.0	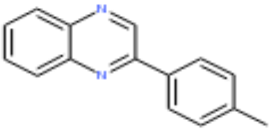		
38389	-8.0	-16.0	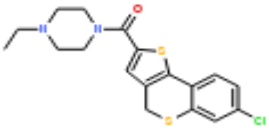		
101616	-13.2	-16.0	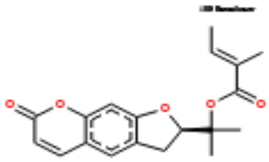		yes
36478	-6.3	-15.0	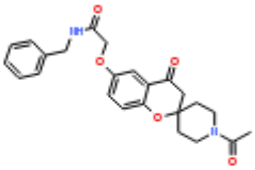		

34907	-7.7	-14.0	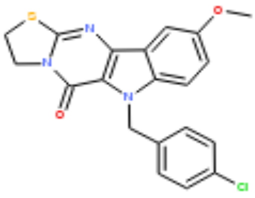		
36403	-6.2	-14.0	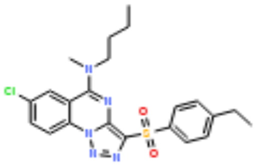		
100452	-15.5	-14.0	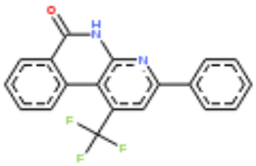		
102118	-15.1	-14.0	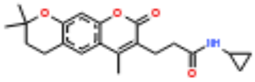		
28125	-10.0	-13.0	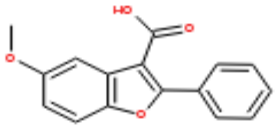		
35348	-7.5	-13.0	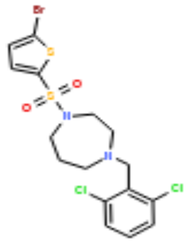		
36280	-6.2	-13.0	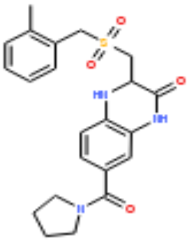		

38060	-7.9	-13.0	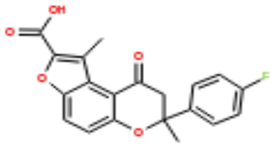	yes
35871	-6.1	-11.0	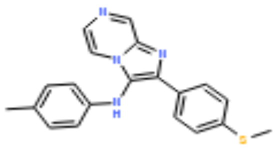	yes
37952	-8.4	-11.0	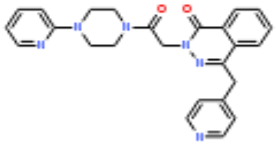	
101643	-12.6	-11.0	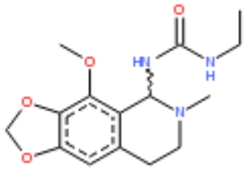	
31413	-9.0	-9.0	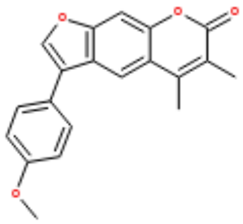	yes
32995	-10.9	-8.0	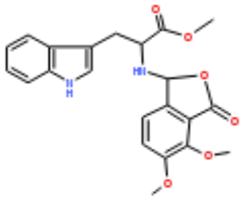	
34592	-7.9	-8.0	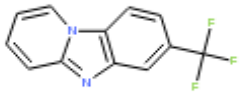	

36534	-5.9	-8.0	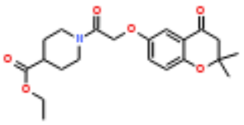		yes
36848	-6.6	-7.0	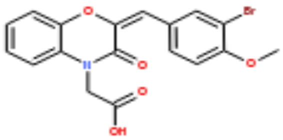		
35614	-6.8	-6.0	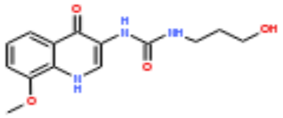		
35885	-5.8	-6.0	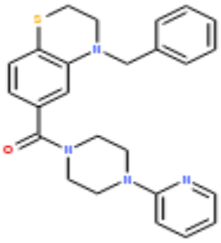		
100587	-14.3	-6.0	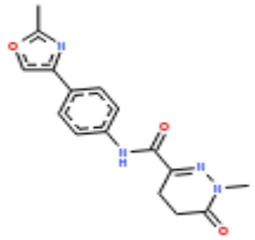		
29217	-11.1	-5.0	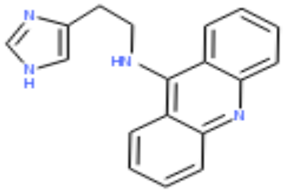		
33136	-6.6	-5.0	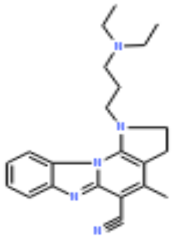		

31764	-8.6	-4.0	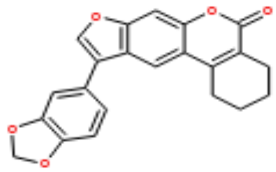		yes
26526	-12.8	-3.0	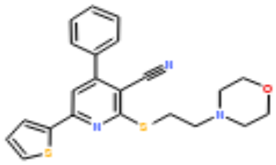		
35518	-6.6	-3.0	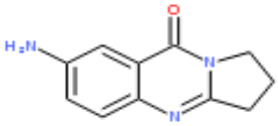		
36024	-5.7	-3.0	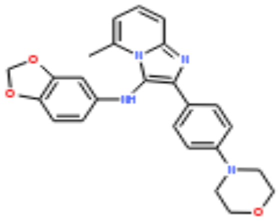		
26494	-12.7	-2.0	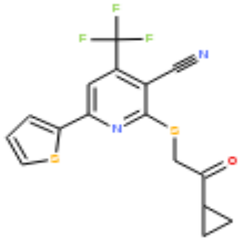		
38357	-7.0	-2.0	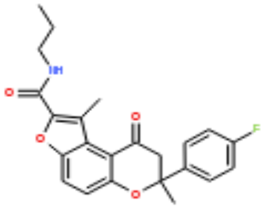		yes
24190	-8.8	-1.0	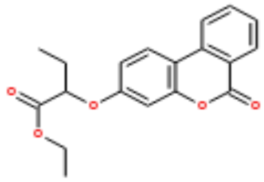		

38322	-6.9	-1.0	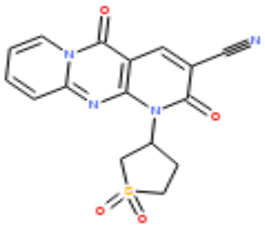		
35820	-6.4	0.0	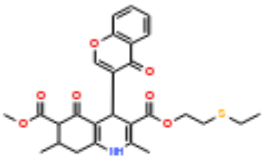		yes
36083	-5.4	0.0	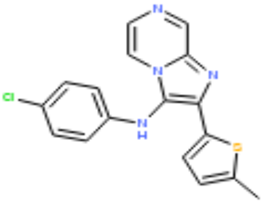		yes
31864	-8.2	1.0	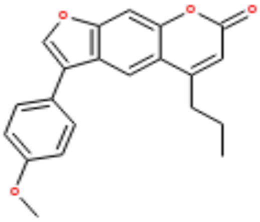		yes
34724	-7.2	1.0	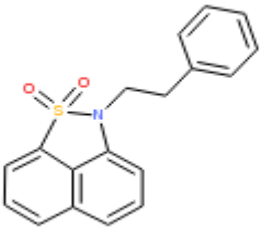		
25603	-10.7	2.0	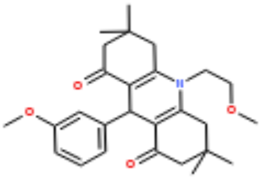		yes
33753	-7.1	2.0	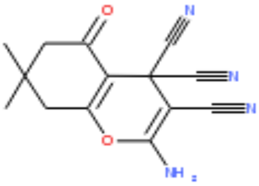		

102347	-13.0	2.0	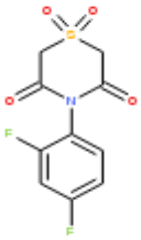		
34714	-7.1	3.0	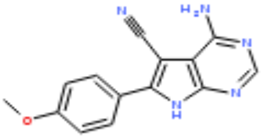		yes
36521	-5.2	3.0	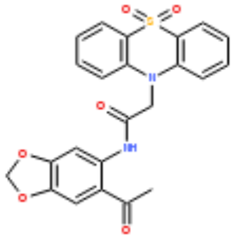		yes
100939	-12.1	3.0	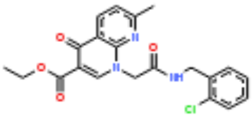		yes
24552	-8.5	4.0	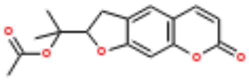		yes
35707	-6.1	4.0	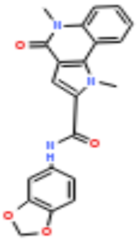		yes
26911	-5.6	5.0	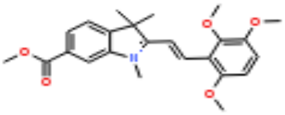		yes

31451	-7.7	5.0	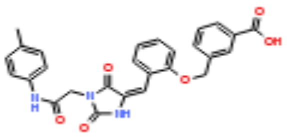	yes
101291	-9.5	5.0	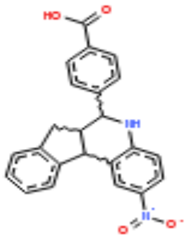	yes
25820	-11.1	6.0	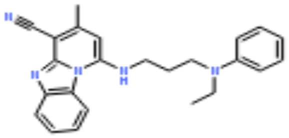	yes
25657	-10.9	7.0	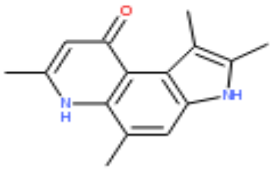	yes
30927	-8.3	7.0	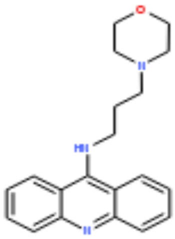	yes
25369	-10.0	8.0	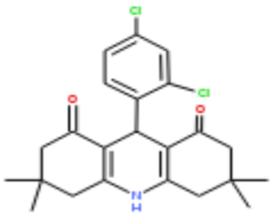	yes
36361	-4.9	8.0	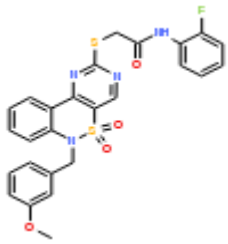	yes

38248	-6.4	8.0	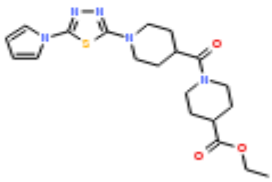		yes
27270	-5.6	9.0	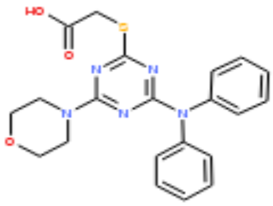		yes
29961	-9.3	9.0	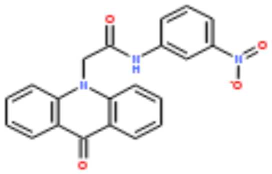		yes
29582	-9.8	10.0	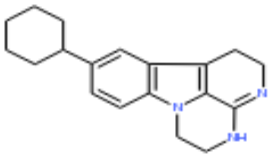		yes
30061	-9.2	10.0	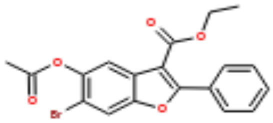		yes
35907	-4.8	11.0	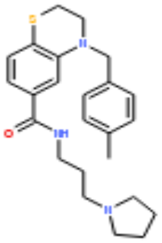		yes
38361	-6.1	11.0	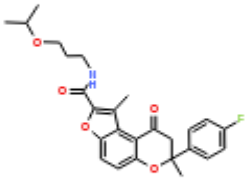		yes

34168	-6.3	12.0	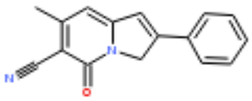	
30843	-8.8	13.0	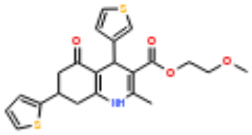	
36506	-4.6	13.0	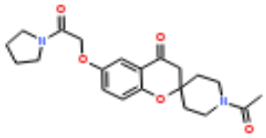	yes
38073	-6.0	13.0	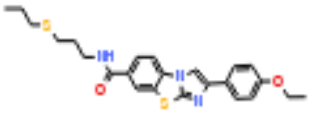	yes
27341	-5.3	14.0	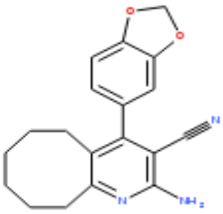	
28162	-7.6	14.0	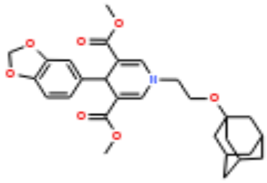	
28569	-8.8	14.0	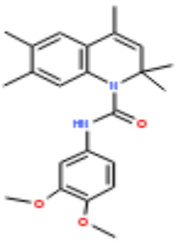	

29003	-7.7	14.0	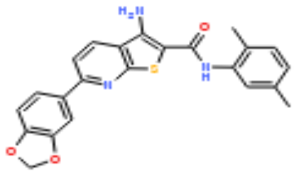		
33847	-6.2	14.0	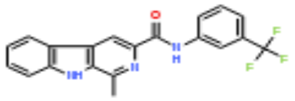		
35555	-5.5	14.0	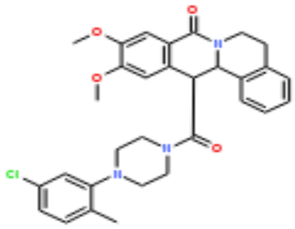		
36987	-5.4	15.0	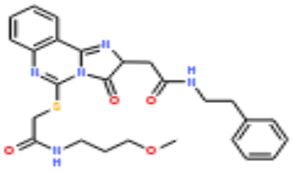		
25835	-9.8	16.0	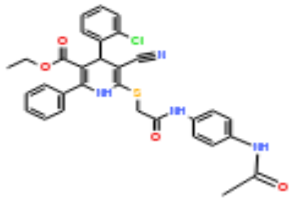		
29051	-7.5	16.0	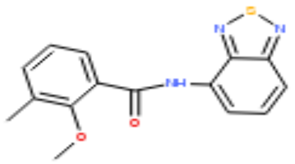		
29199	-8.7	16.0	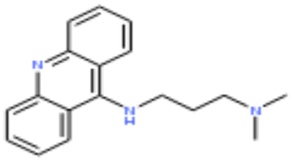		yes

34112	-6.0	16.0	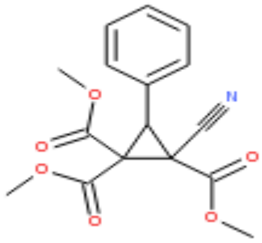		
37942	-6.4	16.0	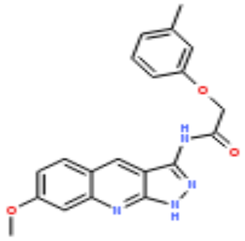		
24407	-7.2	17.0	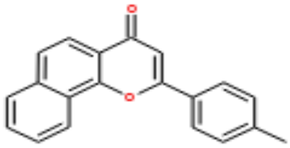		
33815	-6.0	17.0	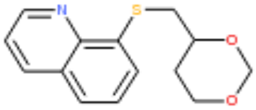		
24645	-7.2	18.0	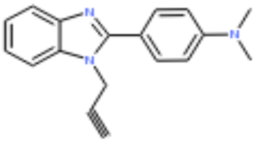		
30199	-8.2	18.0	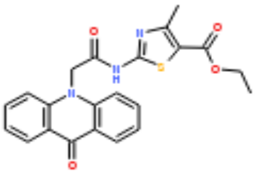		
36295	-4.4	18.0	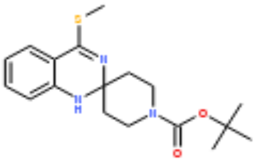		

27317	-4.9	19.0	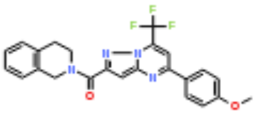		
33202	-5.0	19.0	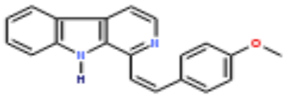		
38056	-5.6	19.0	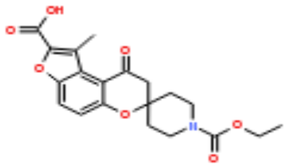		
39127	-9.7	19.0	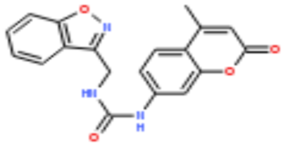		
26754	-4.7	20.0	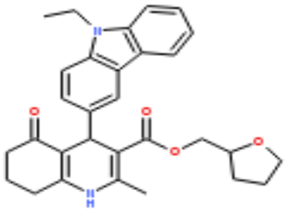		
30054	-8.1	20.0	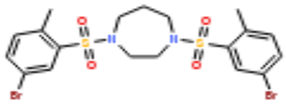		
30197	-8.0	20.0	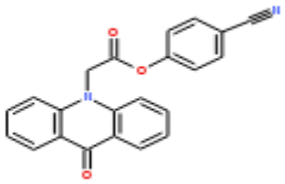		

36762	-4.8	21.0	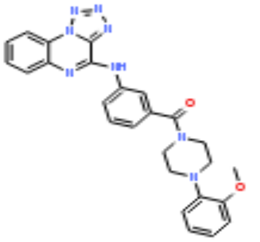		
28029	-5.4	22.0	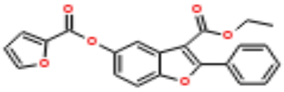		yes
28666	-7.8	22.0	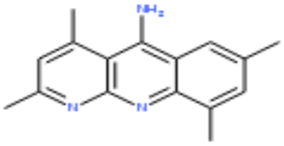		
26828	-4.4	23.0	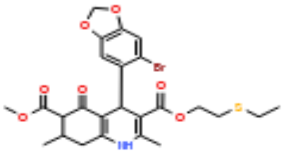		yes
27203	-4.6	23.0	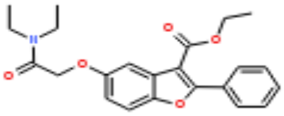		yes
33291	-4.7	23.0	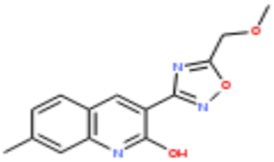		
35883	-4.1	23.0	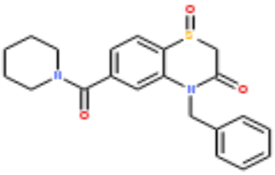		

36542	-4.1	23.0	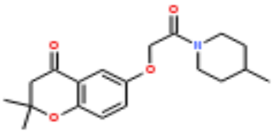	yes
36874	-4.7	23.0	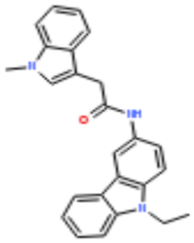	
37698	-5.9	23.0	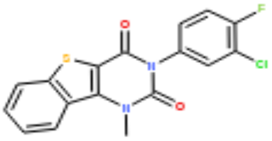	
38989	-9.4	23.0	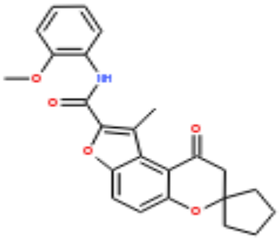	
26620	-9.2	24.0	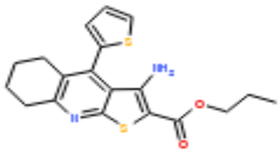	
27681	-5.3	24.0	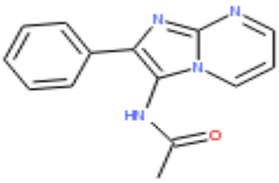	
36125	-4.1	24.0	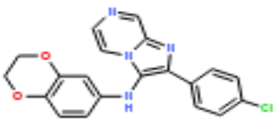	yes

36284	-4.0	24.0	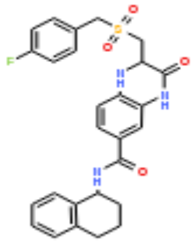		
36676	-4.7	24.0	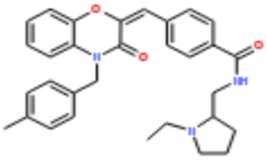		
38062	-5.2	24.0	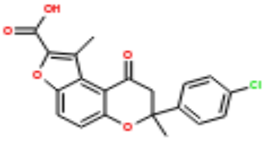		yes
27559	-5.2	25.0	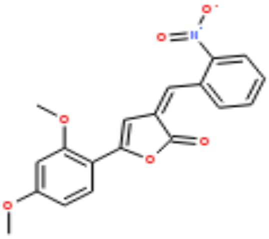		
38520	-5.2	25.0	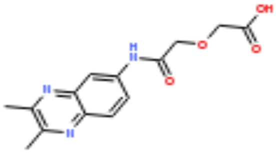		
39639	-8.3	25.0	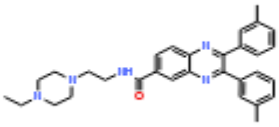		
30207	-7.4	26.0	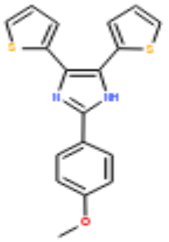		

36021	-4.0	26.0	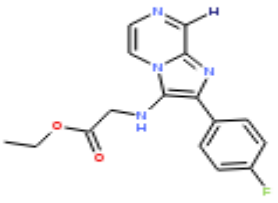	yes
38327	-5.1	26.0	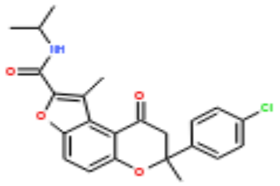	yes
33390	-5.1	27.0	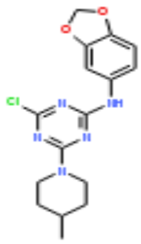	
38715	-8.9	27.0	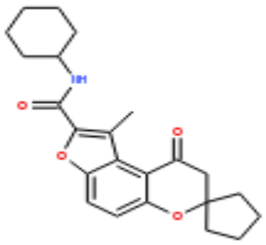	
30682	-7.2	28.0	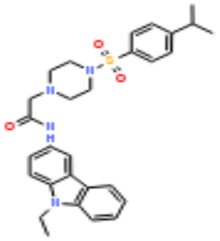	
31588	-5.7	28.0	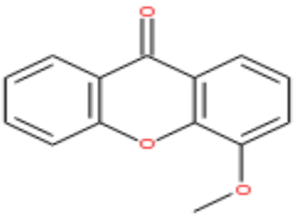	
33931	-5.1	28.0	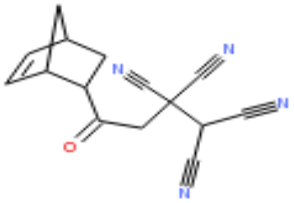	

34900	-4.8	28.0	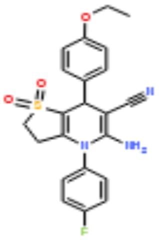		
35099	-4.9	28.0	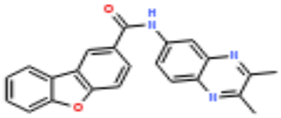		
38683	-8.8	28.0	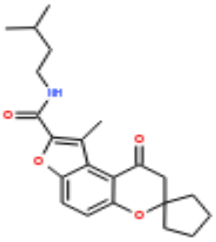		yes
25071	-6.6	29.0	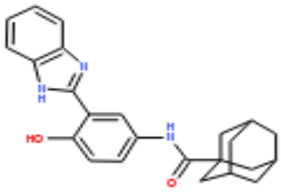		
31756	-5.7	29.0	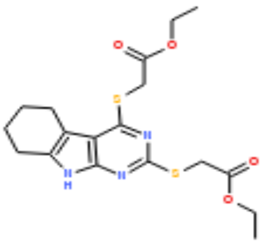		
35504	-4.4	29.0	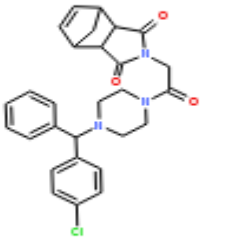		
37674	-5.4	29.0	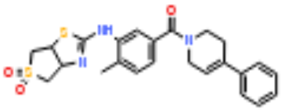		

24206	-5.9	30.0	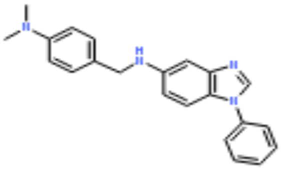		
28968	-6.2	30.0	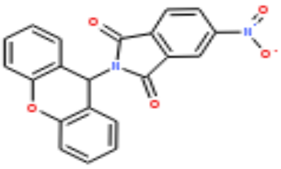		
31063	-6.2	30.0	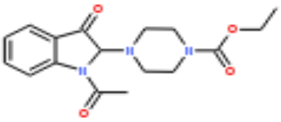		
31802	-5.6	30.0	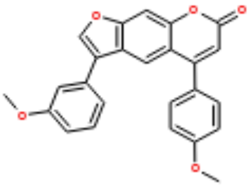		yes
35520	-4.3	30.0	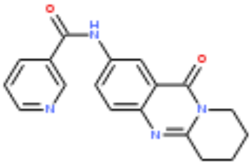		
24694	-6.0	31.0	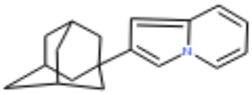		
39523	-7.7	31.0	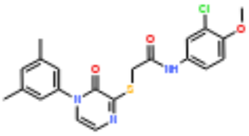		

24717	-5.9	32.0	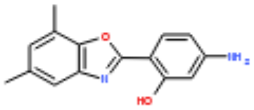		
27311	-4.1	32.0	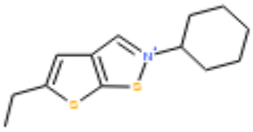		
35582	-4.2	32.0	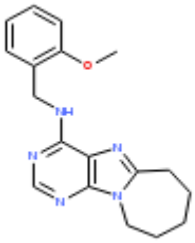		
33211	-4.1	33.0	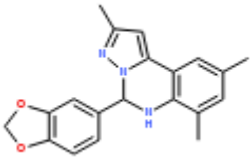		
33444	-4.7	33.0	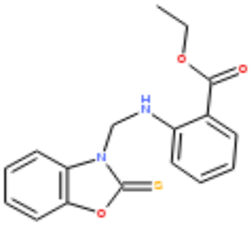		
33760	-4.8	33.0	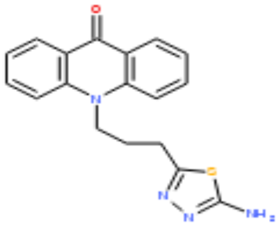		
24664	-5.8	34.0	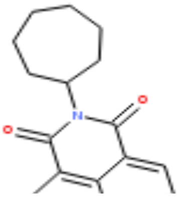		

30936	-5.8	34.0	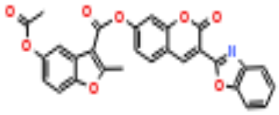		
35415	-4.3	34.0	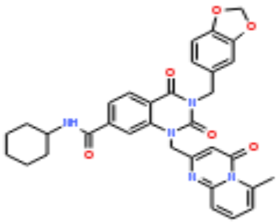		
38359	-4.5	34.0	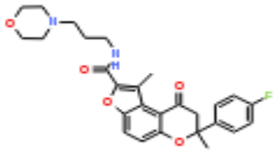		yes
39188	-7.9	34.0	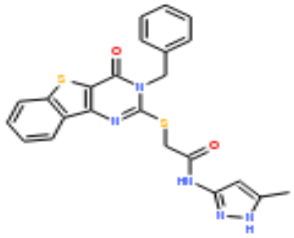		
24422	-5.5	35.0	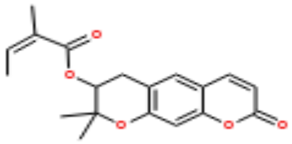		yes
27419	-4.4	35.0	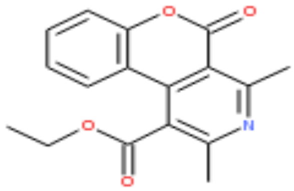		
35278	-4.3	35.0	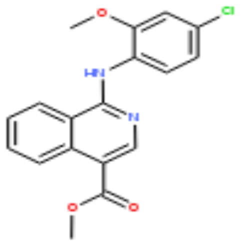		

35503	-3.9	35.0	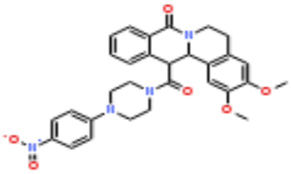		
36919	-4.0	35.0	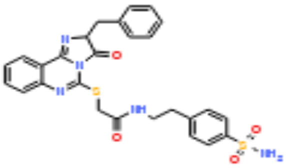		
35326	-4.2	36.0	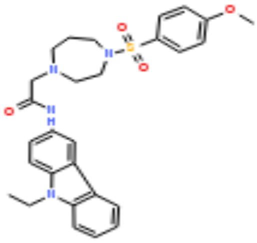		
35751	-3.9	36.0	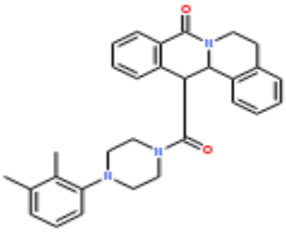		
35985	-3.4	36.0	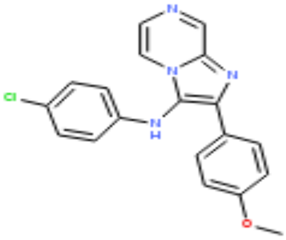		yes
24593	-5.4	37.0	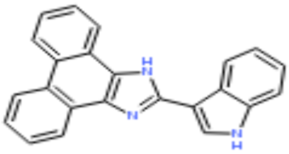		
27376	-3.8	37.0	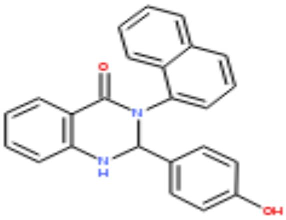		

27905	-4.2	37.0	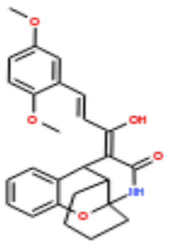		
29937	-6.1	37.0	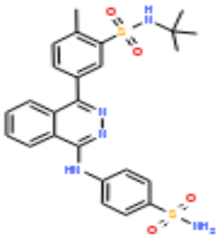		
36837	-3.8	37.0	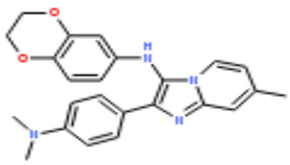		
27867	-4.1	38.0	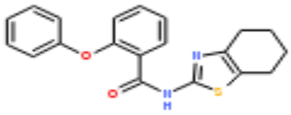		
29413	-6.2	38.0	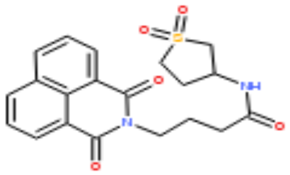		
31762	-4.9	38.0	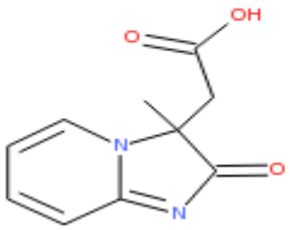		
33076	-3.8	38.0	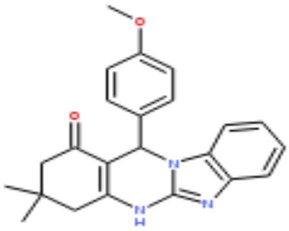		

36219	-3.2	38.0	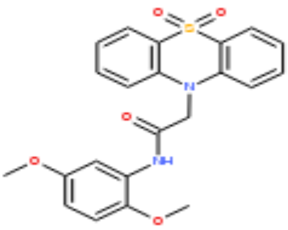	yes
38039	-4.2	38.0	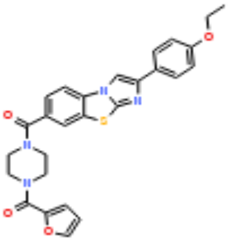	yes
38156	-4.3	38.0	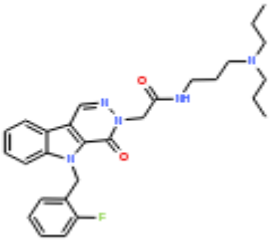	
100455	-8.2	38.0	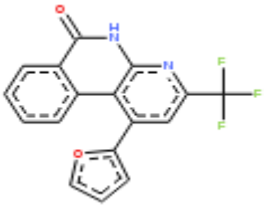	
29880	-6.0	39.0	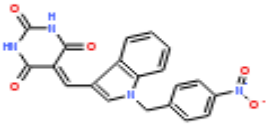	
31047	-5.3	39.0	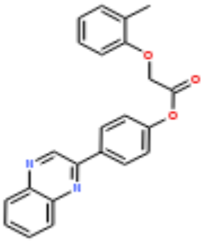	
31106	-5.4	39.0	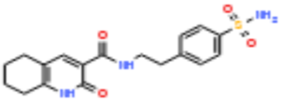	

31573	-4.8	39.0	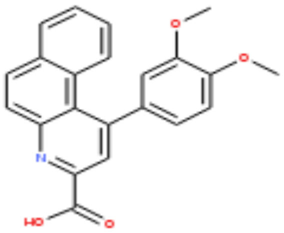		
33055	-3.7	39.0	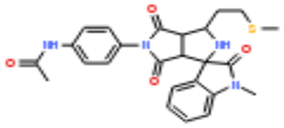		
35466	-4.0	39.0	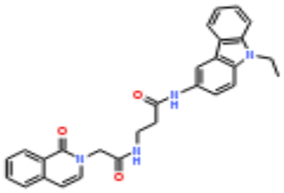		
35957	-3.2	39.0	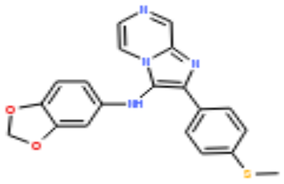		yes
100655	-8.1	39.0	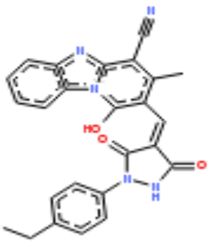		
26598	-7.1	40.0	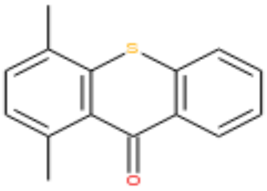		
27359	-3.6	40.0	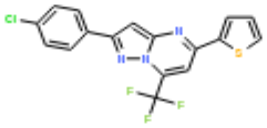		

31487	-4.7	40.0	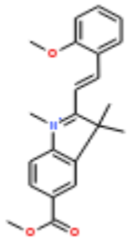		yes
32804	-6.0	40.0	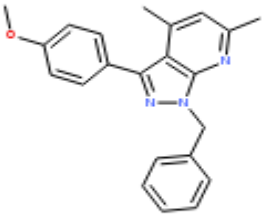		
30854	-5.6	41.0	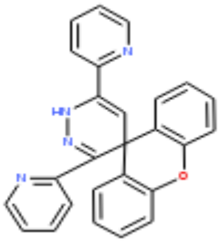		
38725	-7.1	41.0	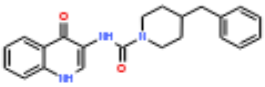		
40184	-6.7	41.0	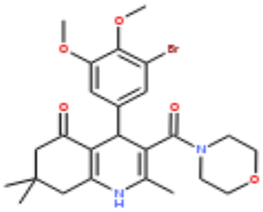		
27971	-3.8	42.0	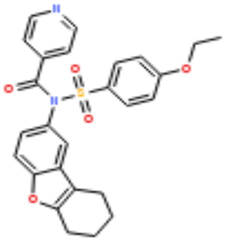		
33447	-4.1	42.0	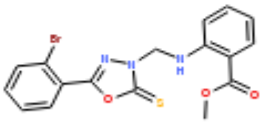		

35045	-3.9	42.0	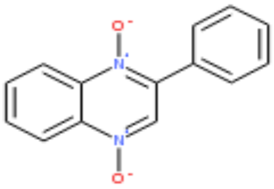		
35152	-3.8	42.0	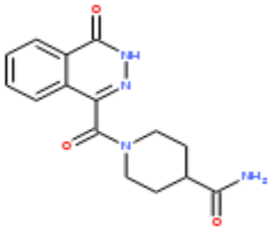		
35857	-3.0	42.0	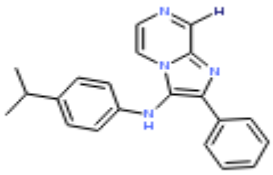		yes
36921	-3.5	42.0	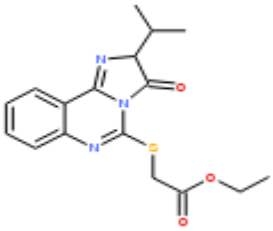		
37348	-3.6	42.0	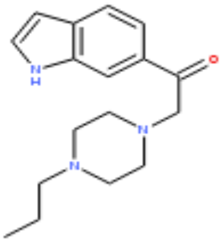		
37804	-4.4	42.0	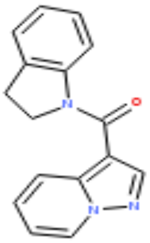		
38737	-7.0	42.0	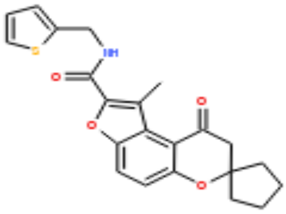		

101552	-6.7	42.0	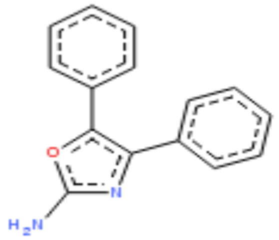		
25139	-5.3	43.0	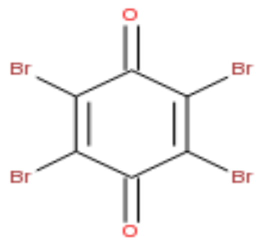		
26877	-3.0	43.0	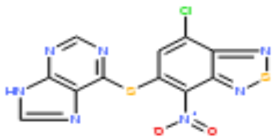		
33118	-3.5	43.0	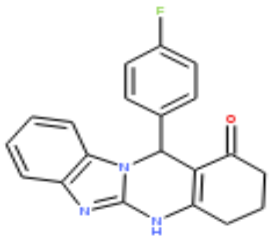		
35641	-3.4	43.0	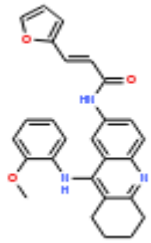		
35735	-3.4	43.0	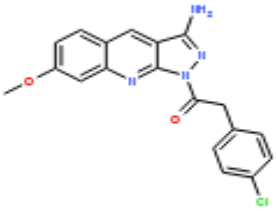		
36714	-3.4	43.0	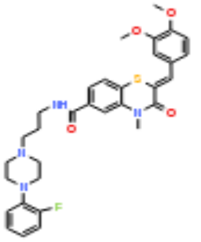		

36815	-3.4	43.0	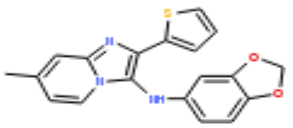		
101101	-5.7	43.0	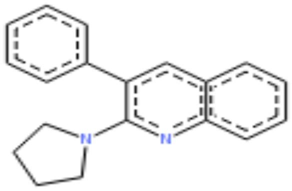		
29411	-5.6	44.0	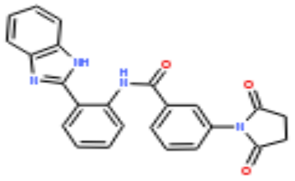		
29938	-5.4	44.0	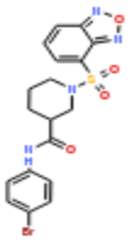		
30025	-5.3	44.0	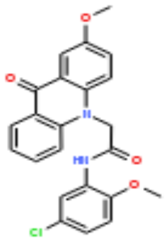		
31391	-4.3	44.0	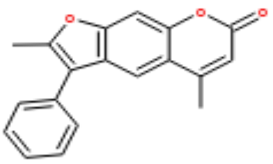		yes
31420	-4.4	44.0	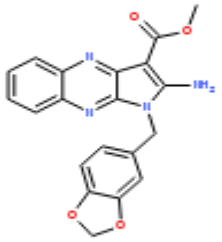		

35502	-3.3	44.0	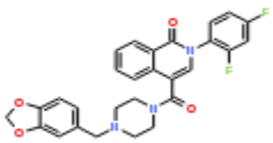		
36718	-3.4	44.0	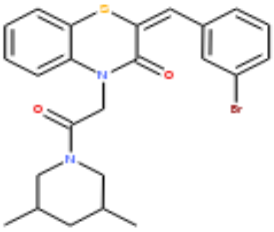		
36909	-3.4	44.0	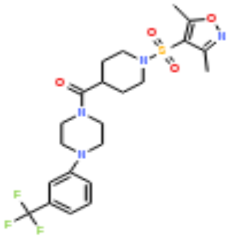		
101694	-6.4	44.0	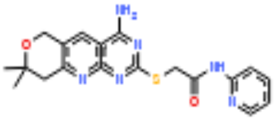		
27517	-3.6	45.0	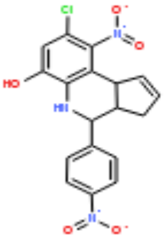		
27645	-3.7	45.0	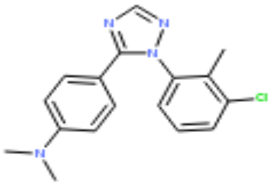		
33594	-3.9	45.0	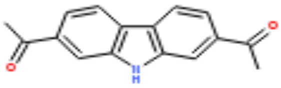		

34447	-3.8	45.0	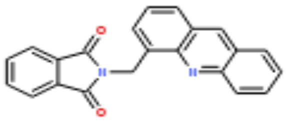		
36576	-3.3	45.0	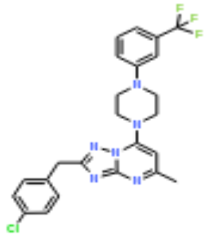		
40224	-6.3	45.0	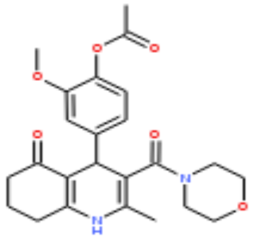		
26609	-6.3	46.0	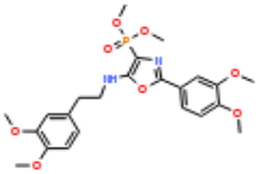		
27395	-3.6	46.0	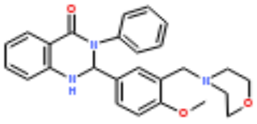		
30734	-5.1	46.0	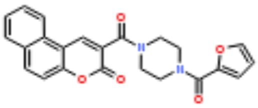		
31770	-4.2	46.0	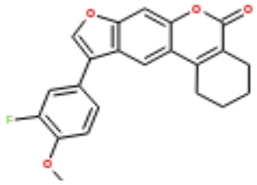		yes

33759	-3.8	46.0	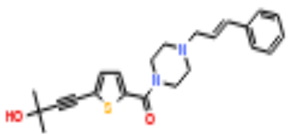		
34095	-3.6	46.0	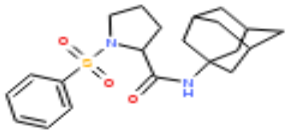		
34464	-3.7	46.0	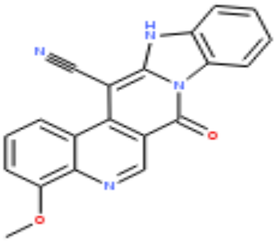		
35164	-3.6	46.0	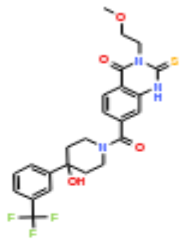		
101586	-6.3	46.0	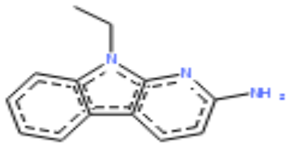		
27183	-3.1	47.0	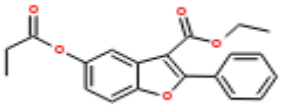		yes
27233	-3.1	47.0	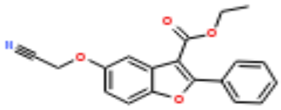		yes

27823	-3.5	47.0	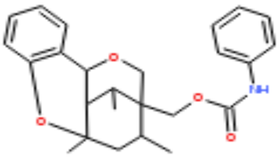		
32557	-5.1	47.0	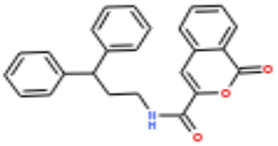		
34187	-3.6	47.0	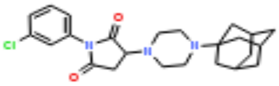		
35515	-3.1	47.0	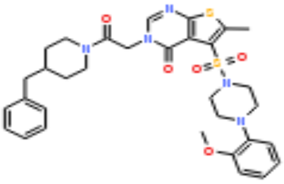		
37248	-3.2	47.0	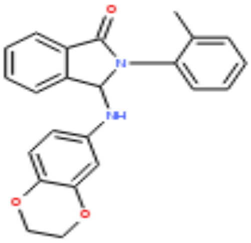		
37322	-3.3	47.0	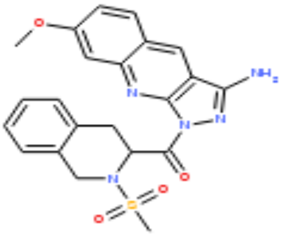		
38337	-3.6	47.0	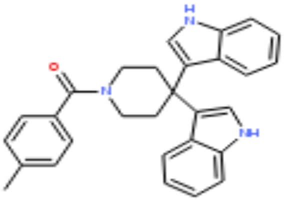		

27391	-3.1	48.0	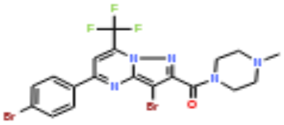		
30656	-4.9	48.0	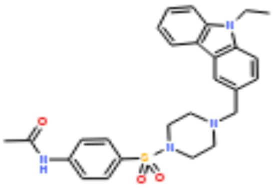		
37949	-4.0	48.0	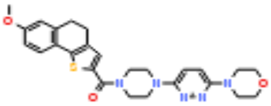		
39433	-5.7	48.0	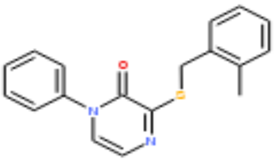		
23898	-4.2	49.0	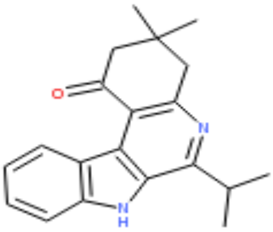		
24066	-4.2	49.0	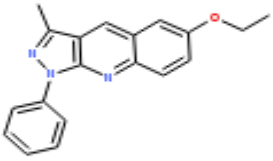		
24698	-4.3	49.0	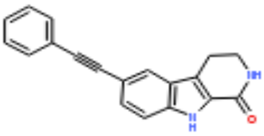		

27463	-3.4	49.0	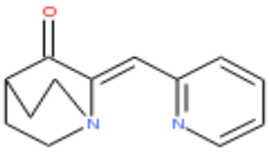		
27834	-3.3	49.0	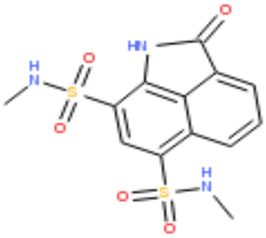		
27900	-3.3	49.0	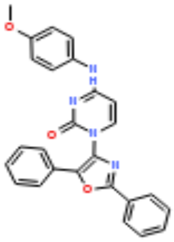		
30194	-4.8	49.0	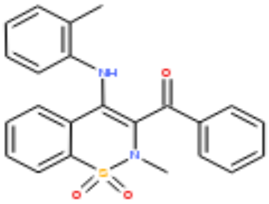		
31116	-4.4	49.0	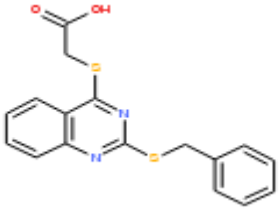		
31657	-3.9	49.0	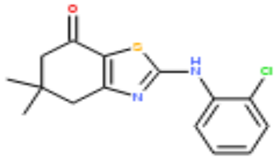		
31899	-3.9	49.0	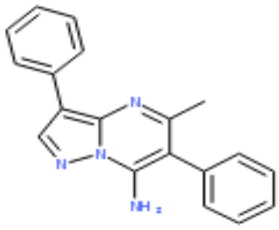		

33776	-3.6	49.0	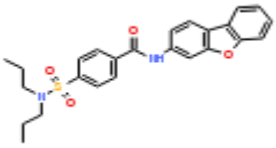		
35408	-3.4	49.0	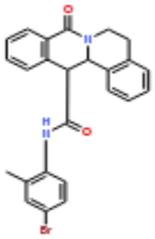		
37133	-3.1	49.0	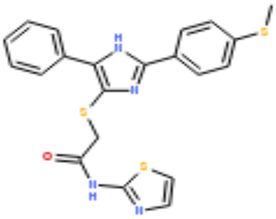		
37878	-4.0	49.0	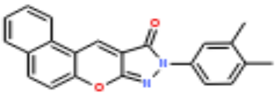		
38679	-6.1	49.0	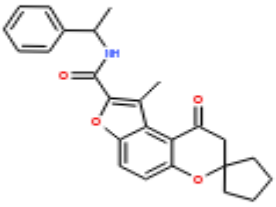		
39547	-5.5	49.0	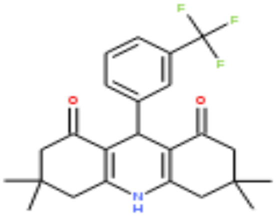		yes
27519	-3.3	50.0	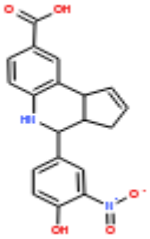		yes

27636	-3.3	50.0	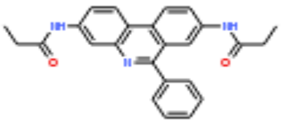		
32000	-4.5	50.0	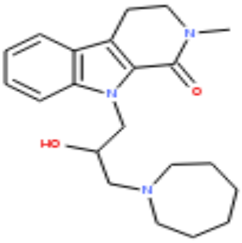		
32121	-4.6	50.0	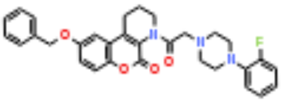		
34448	-3.4	50.0	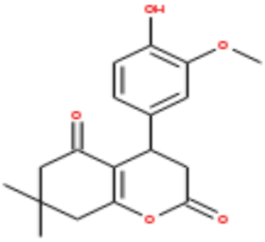		
34800	-3.3	50.0	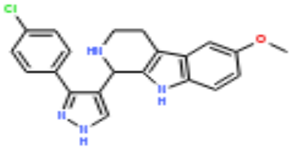		
35391	-3.3	50.0	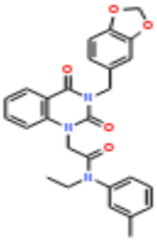		
38685	-6.0	50.0	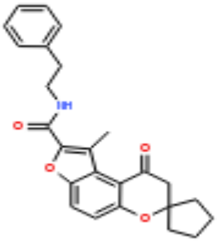		

24381	-4.1	51.0	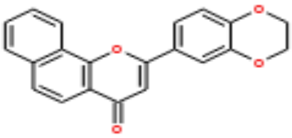	
25423	-5.0	51.0	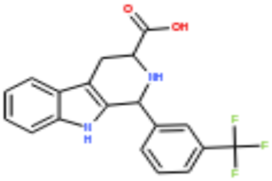	
28670	-4.7	51.0	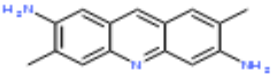	
38054	-3.3	51.0	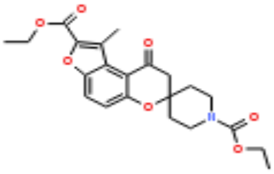	
38208	-3.3	51.0	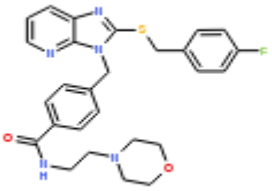	
38321	-3.4	51.0	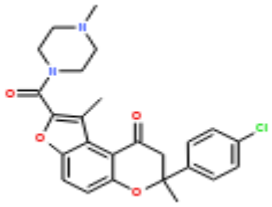	yes
38329	-3.3	51.0	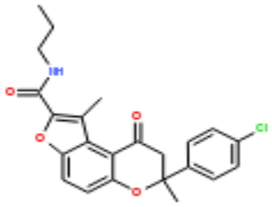	yes

27877	-3.0	52.0	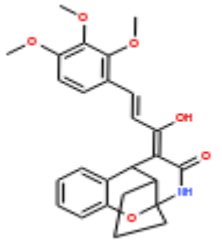		
27911	-3.1	52.0	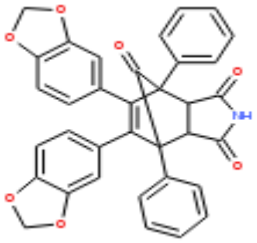		
28164	-4.3	52.0	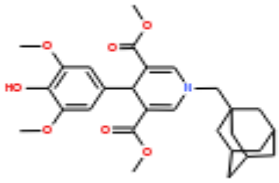		
29081	-4.2	52.0	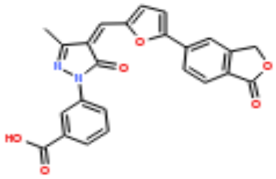		
29920	-4.5	52.0	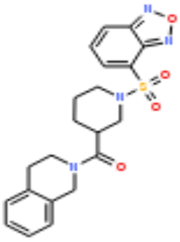		
30392	-4.7	52.0	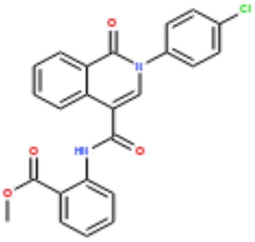		
34108	-3.1	52.0	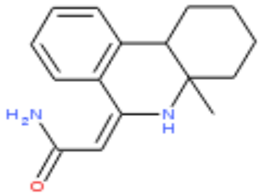		

34534	-3.2	52.0	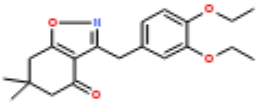		
38333	-3.3	52.0	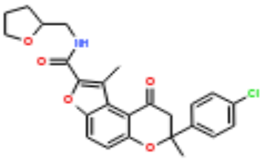		yes
27580	-3.1	53.0	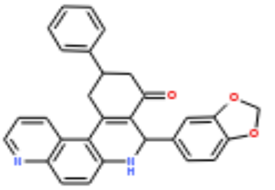		
28413	-4.2	53.0	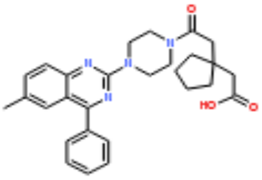		
28747	-4.5	53.0	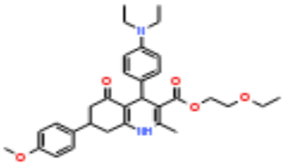		
31255	-4.0	53.0	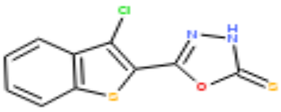		
37704	-3.6	53.0	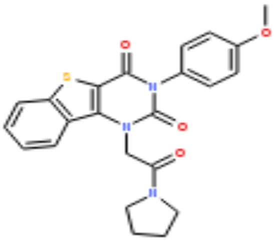		

38087	-3.2	53.0	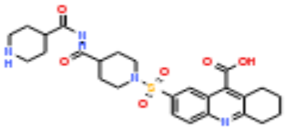		
38092	-3.2	53.0	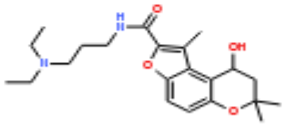		
38991	-5.6	53.0	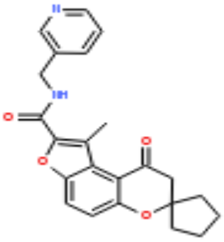		
40274	-5.3	53.0	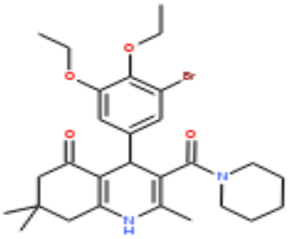		
100485	-6.0	53.0	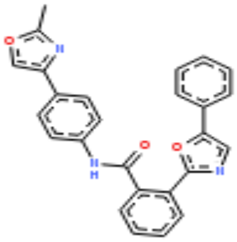		
28391	-4.2	54.0	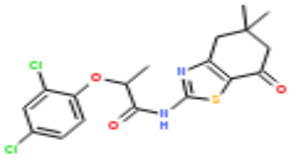		
29973	-4.3	54.0	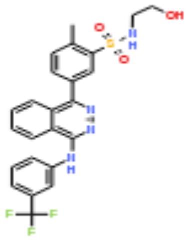		

31744	-3.5	54.0	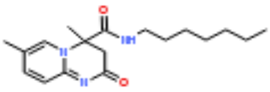		
31988	-4.1	54.0	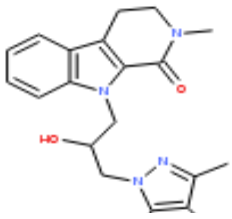		
32145	-4.1	54.0	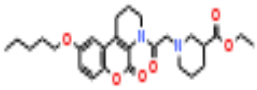		
33023	-4.7	54.0	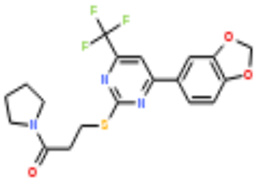		
34816	-3.1	54.0	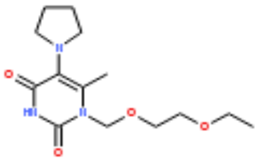		
35305	-3.0	54.0	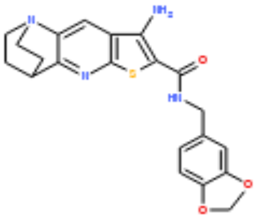		
38353	-3.2	54.0	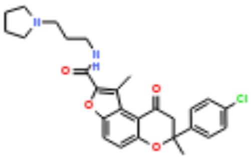		yes

38891	-5.5	54.0	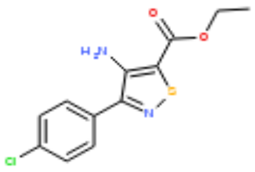		
100416	-6.0	54.0	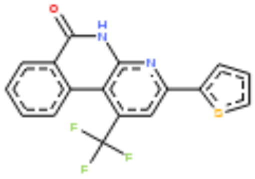		
30273	-4.4	55.0	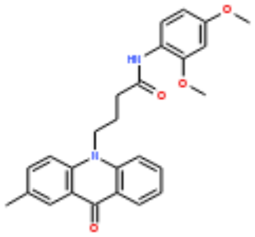		
31426	-3.4	55.0	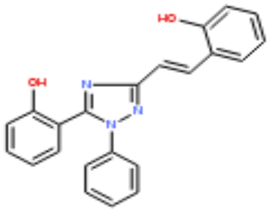		
31432	-3.4	55.0	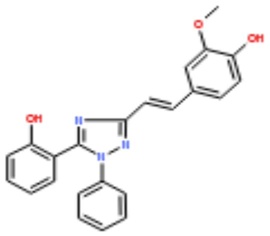		
32352	-4.3	55.0	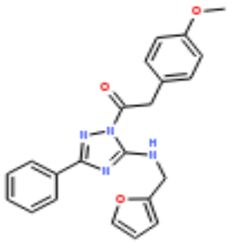		
33615	-3.2	55.0	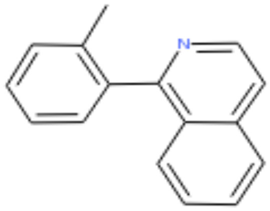		

33964	-3.1	55.0	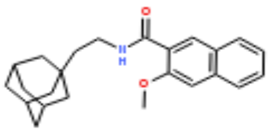		
34506	-3.0	55.0	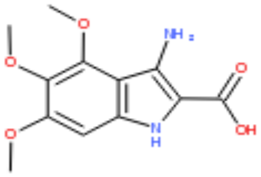		
34518	-3.0	55.0	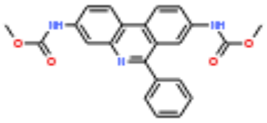		
39469	-4.9	55.0	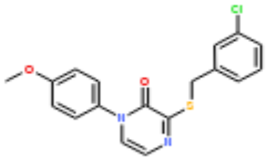		
25454	-4.4	56.0	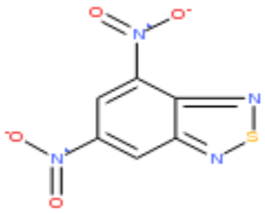		
25571	-4.4	56.0	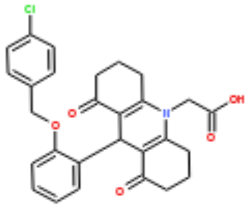		yes
32302	-3.9	56.0	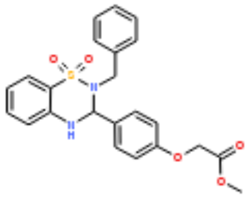		

32550	-4.1	56.0	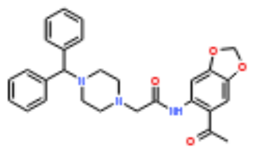		
39525	-4.7	56.0	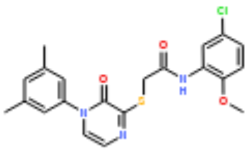		
40128	-5.1	56.0	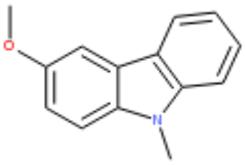		
24032	-3.5	57.0	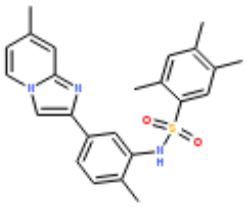		
25145	-4.0	57.0	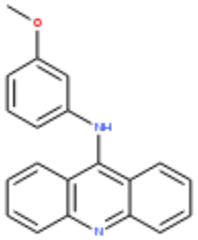		
31046	-3.7	57.0	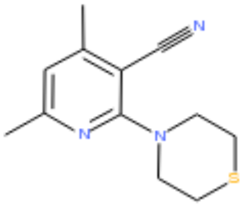		
32431	-4.0	57.0	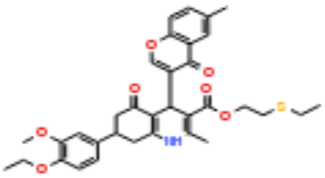		yes

33616	-3.1	57.0	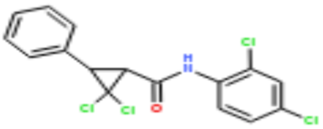	
37632	-3.3	57.0	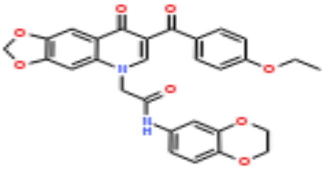	
37721	-3.3	57.0	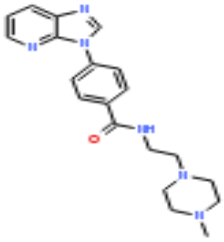	
101029	-4.9	57.0	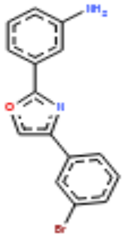	
101196	-4.4	57.0	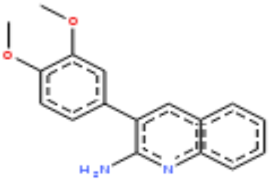	
101319	-4.3	57.0	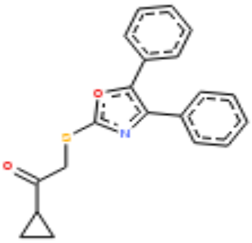	
23924	-3.4	58.0	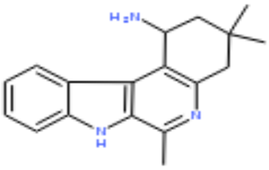	

24207	-3.4	58.0	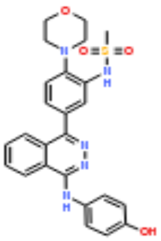		
28704	-3.9	58.0	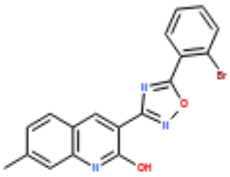		
29023	-3.6	58.0	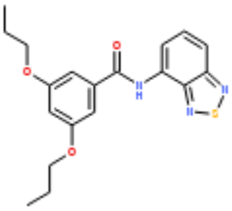		
31835	-3.1	58.0	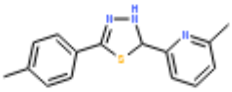		
38717	-4.9	58.0	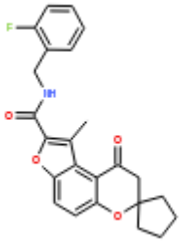		yes
39491	-4.5	58.0	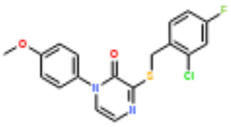		
24409	-3.4	59.0	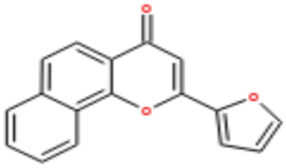		

25291	-4.0	59.0	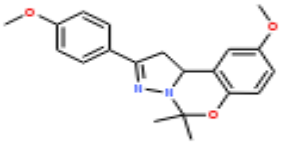	
26116	-4.7	59.0	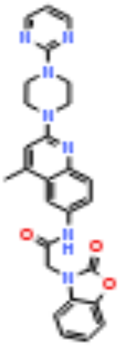	
28545	-3.8	59.0	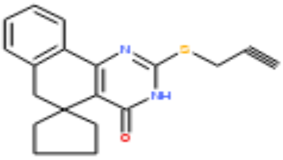	
32356	-3.8	59.0	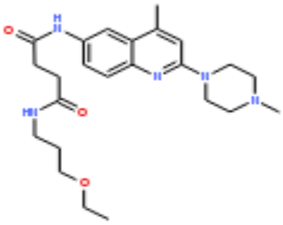	
25388	-3.9	60.0	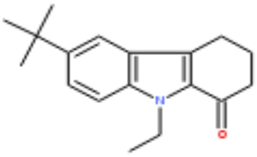	
28549	-3.7	60.0	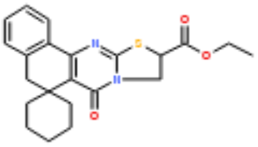	
29184	-3.7	60.0	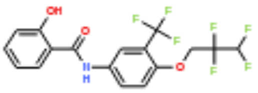	

32810	-4.0	60.0	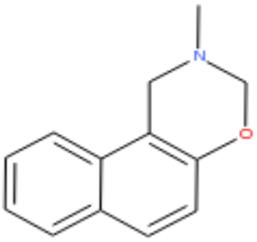		
37622	-3.1	60.0	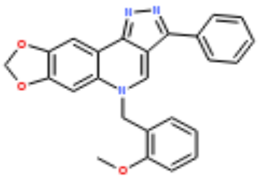		
38845	-4.7	60.0	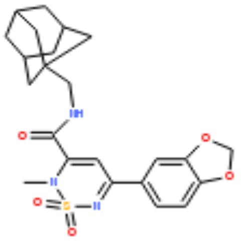		
39457	-4.3	60.0	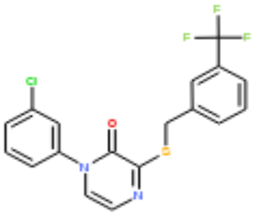		
100573	-5.2	60.0	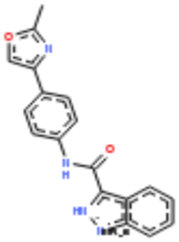		
101092	-4.1	60.0	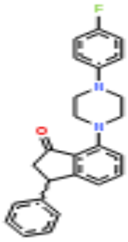		
24068	-3.1	61.0	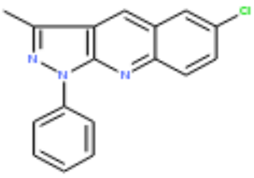		

25090	-3.5	61.0	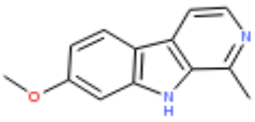		
25803	-4.4	61.0	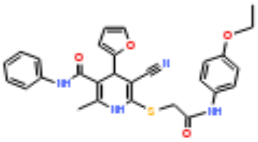		
30058	-3.5	61.0	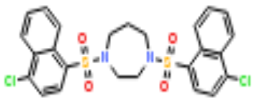		
30608	-3.5	61.0	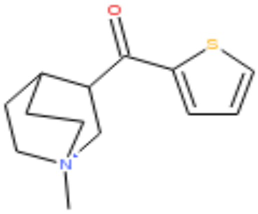		
31151	-3.3	61.0	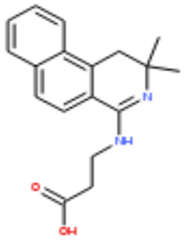		
39535	-4.1	61.0	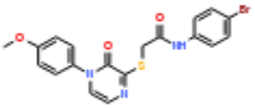		
25424	-3.6	62.0	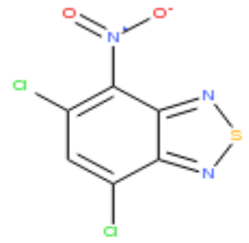		

30027	-3.4	62.0	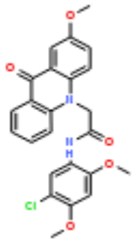		
30745	-3.4	62.0	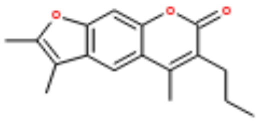		
32140	-3.2	62.0	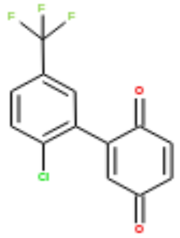		
40246	-4.3	62.0	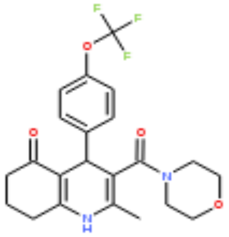		
26026	-4.1	63.0	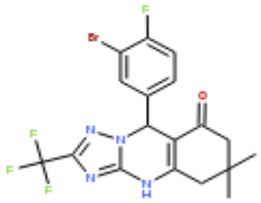		
28678	-3.4	63.0	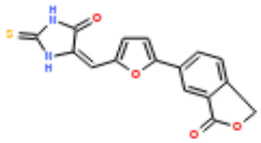		
30607	-3.2	63.0	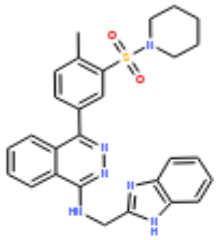		

31994	-3.0	63.0	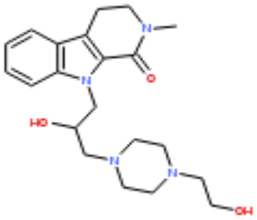		
32521	-3.4	63.0	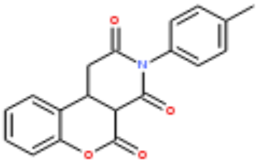		
32958	-3.7	63.0	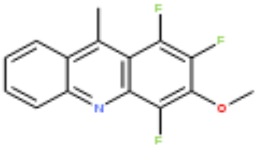		
26613	-3.8	64.0	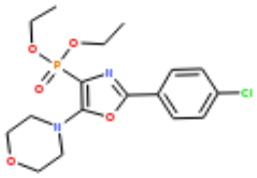		
30773	-3.1	64.0	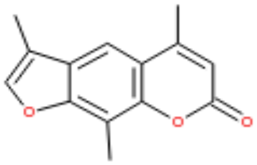		
30876	-3.1	64.0	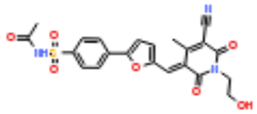		
30966	-3.0	64.0	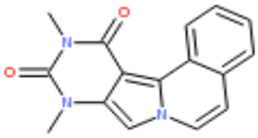		

39495	-3.9	64.0	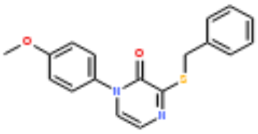		
40250	-4.1	64.0	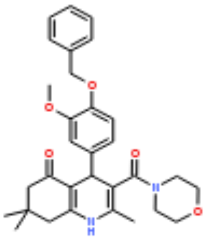		
101160	-3.6	64.0	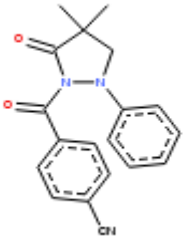		
25441	-3.2	65.0	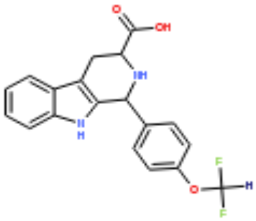		
26441	-3.7	65.0	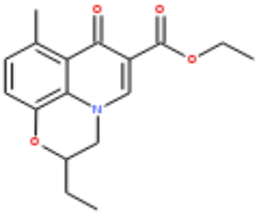		
29075	-3.0	65.0	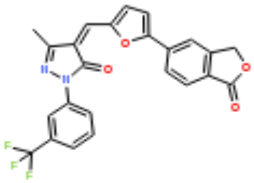		
29885	-3.1	65.0	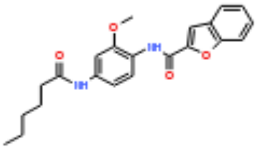		

30592	-3.0	65.0	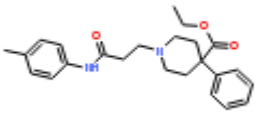		
38677	-4.0	65.0	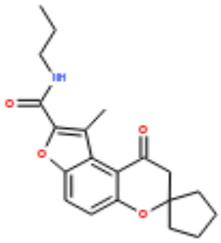		yes
29385	-3.1	66.0	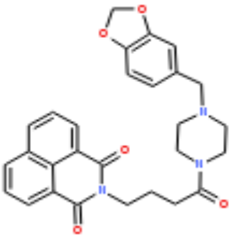		
30218	-3.2	66.0	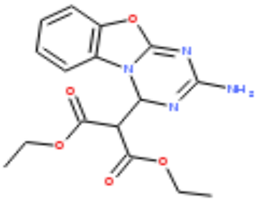		
30515	-3.2	66.0	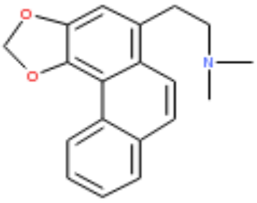		
32368	-3.0	66.0	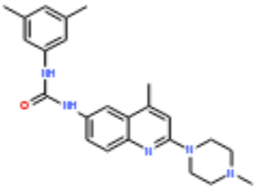		
38977	-3.9	66.0	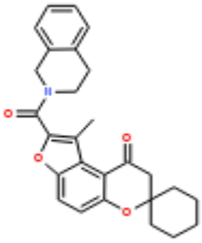		

29516	-3.3	67.0	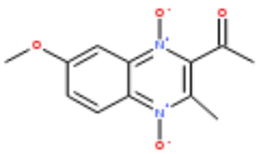		
29610	-3.4	67.0	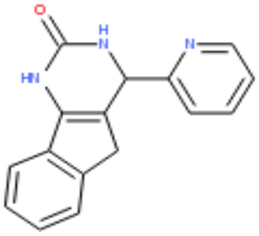		
101280	-3.3	67.0	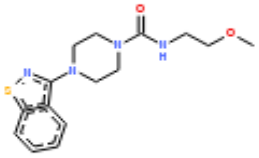		
25646	-3.5	68.0	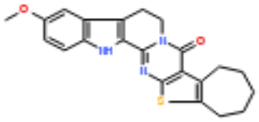		
32753	-3.1	68.0	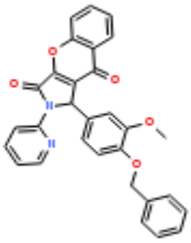		
26560	-3.0	70.0	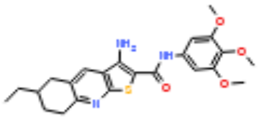		
38711	-3.4	70.0	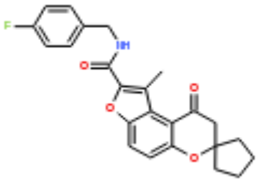		

40169	-3.4	70.0	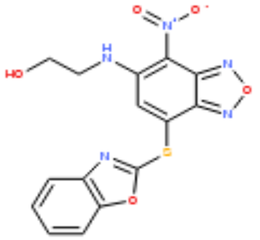		
39007	-3.3	71.0	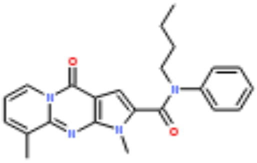		
100453	-3.6	71.0	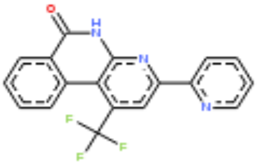		
39164	-3.0	73.0	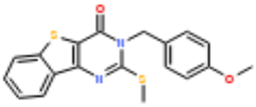		

1.2.2. Suppl. Tab. 2

Comp_ID	z_score	ResidualPercentActivity	ClosestNames	PNG_Image	IC50_uM	IC50_comment	FitIsValid
24181	0.1	96.0	24181,25337,25369,25371	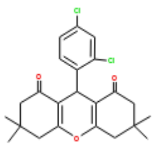			
24422	-5.5	35.0	24422,101616,24552,24550,24528,24526,101618	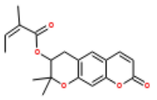	30		True
24526	-25.6	-178.0	24526,101618,24552,101616,24550,24422	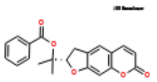		artifact, fluorescence bleaching	True
24528	-1.7	76.0	24528,24422,24550,24552,101616	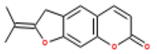			
24546	-0.2	92.0	24546	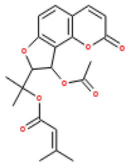			
24550	-1.4	79.0	24550,24552,101616,24526,101618,24422,100617,101626,24528	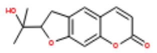			
24552	-8.5	4.0	24552,101616,24526,101618,24550,24422,100617,101626,24528	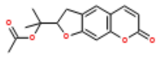			
25309	-2.1	75.0	25309,31379,25329,25541,25613,25343,25543,25365,31381,39547	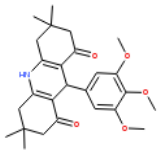			
25329	-25.2	-120.0	25329,25541,25343,25365,25543,39547,30331,25309,25371,25369,25615,25613,31379,40220	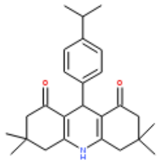			
25337	0.2	94.0	25337,24181,25339,25361,25365,25363	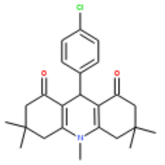			
25339	1.5	105.0	25339,25603,25575,25609,25337,25551,25607,34444,25363,30593	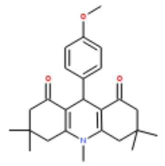			

25341	-2.0	75.0	25341	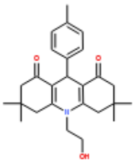			
25343			25343,25329,25541,25365,25543,39547,30331,25309,25371,25369,25615,25613,31379	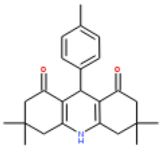			
25361	-0.7	87.0	25361,25337	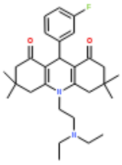			
25363	0.5	97.0	25363,25337,25339	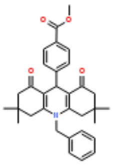			
25365	-13.3	-20.0	25365,25369,25371,25329,25541,25343,25615,40220,25613,39547,25543,31379,30331,25337,25309,31381,28824	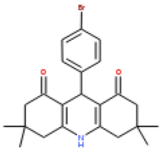			
25369	-10.0	8.0	25369,25371,25365,25329,25541,24181,25343,25615,40220,25613,39547,25543,31379	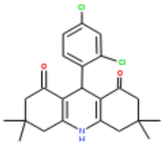			
25371			25371,25369,25365,25541,25329,25343,39547,25615,25613,40220,25543,31379,24181	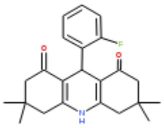			
25541			25541,25329,25343,39547,25365,25543,25371,30331,25309,25369,25615,25613,31379,40220	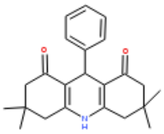			
25543			25543,25329,25541,25343,25365,25309,39547,30331,25613,25371,25369,25615,31379	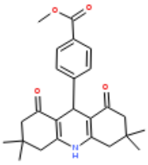			
25549	-1.9	76.0	25549,25551,30593,25573,25569,25609,25603,25575,30575,25571	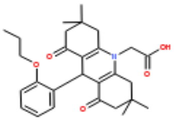			
25551	-1.6	79.0	25551,30593,25549,25573,25339,25609,25603,25575,30575,25569,25607,25605	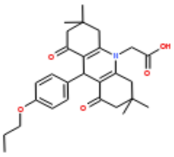			

25569	-1.2	82.0	25569,25573,25571,30597,25549,30595,25551	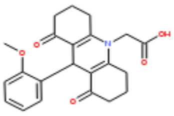			
25571	-4.4	56.0	25571,30597,25569,25573,25549	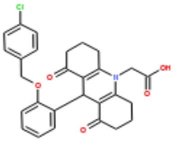			
25573	-2.3	73.0	25573,25569,25549,25551,25571,30593,30595,30597	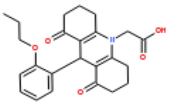			
25575	2.2	111.0	25575,25339,25603,25609,25551,30575,30593,25549,25607,25605	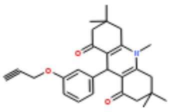			
25601	0.6	98.0	25601,25605,25607,30595	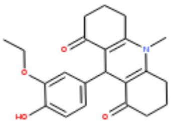			
25603	-10.7	2.0	25603,25339,25609,25575,25605,31357,25607,25551,30593,25549	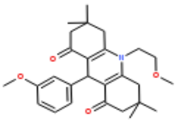			
25605	0.8	99.0	25605,25607,31357,25601,25603,25609,30593,25551,25575	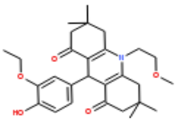			
25607	0.3	94.0	25607,25605,25609,31357,25601,25603,25339,30593,25551,25575	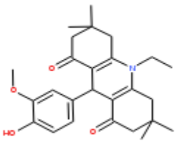			
25609	0.3	95.0	25609,25603,30593,25339,25607,25575,25605,25551,25549,30575,31357,31359	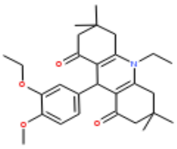			
25613			25613,31379,25365,25309,25615,25329,25371,25541,31381,25369,25343,25543,39547	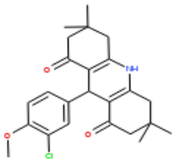			
25615	-0.3	90.0	25615,31381,25365,25329,25541,25371,25613,25343,25369,31379,39547,25543	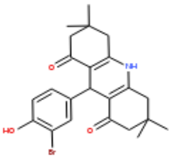			

25657	-10.9	7.0	25657	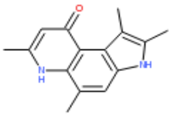			
25778	1.0	106.0	25778,25814,25782,25786	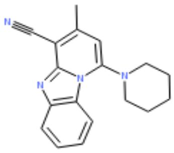			
25780			25780,25820,25790,25824,25784,25788,25812	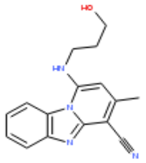			
25782	0.9	105.0	25782,25778,25814,25786	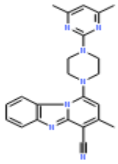			
25784	-0.0	97.0	25784,25812,25790,25820,25780,25824	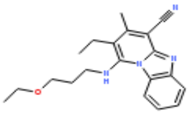			
25786	-0.1	97.0	25786,25814,25778,25782	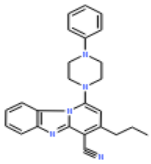			
25788			25788,25790,25824,25780	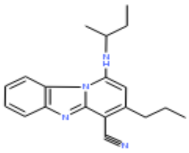			
25790			25790,25812,25788,25824,25784,25780,25820	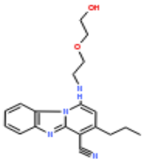			
25792	-23.4	-96.0	25792	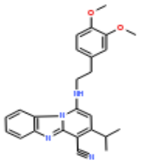			
25812	0.1	99.0	25812,25784,25790,25780	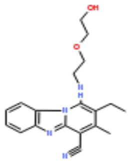			
25814	-2.8	74.0	25814,25778,25786,25782	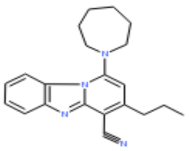			

25820	-11.1	6.0	25820,25780,25784,25790,25824	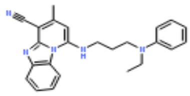	15		True
25824	-1.6	84.0	25824,25788,25790,25780,25784,25820	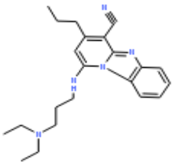			
26760	-26.2	-295.0	26760,26764,26828,35820	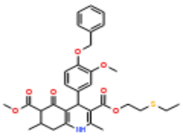		artifact, fluorescence bleaching	True
26764	-0.5	80.0	26764,26760,26828,35820	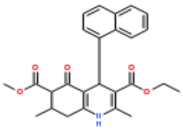			
26828	-4.4	23.0	26828,26832,26760,26764	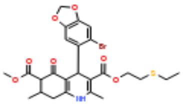		artifact, fluorescence bleaching	True
26832	-2.1	56.0	26832,26828	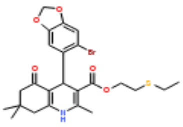		artifact, fluorescence bleaching	True
26897	-9.6	-53.0	26897,26901,26909,31487,26911,31483,31481	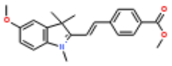			
26901	-18.9	-190.0	26901,26897,26909,31487,26911,31483,31481	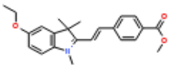		artifact, fluorescence bleaching	True
26909	-8.2	-32.0	26909,26911,31487,26897,26901,31483,31481	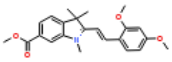		artifact, fluorescence bleaching	True
26911	-5.6	5.0	26911,26909,31487,26897,26901,31483,31481	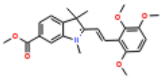		artifact, fluorescence bleaching	True
27175	-2.4	59.0	27175,27183,27245,27203,27233,30061,28029,30081,30083,30085,30063	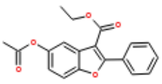			

27183	-3.1	47.0	27183,27245,27175,27203,27233,28029,30061,30081,30083,30085	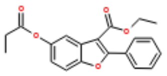		artifact, fluorescence bleaching	True
27203	-4.6	23.0	27203,27175,27183,27233,27245,28029,30083,30085,30061,30081,30063	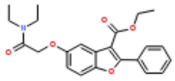			
27233	-3.1	47.0	27233,30063,27175,27203,27183,28029,27245,30081,30083,30061,30085	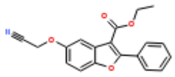		artifact, fluorescence bleaching	True
27245	-143.0	-2117.0	27245,27183,27175,27203,27233,28029,30081,30061,30085	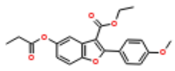			
27268	0.4	101.0	27268,27270	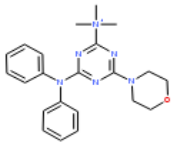			
27270	-5.6	9.0	27270,27268	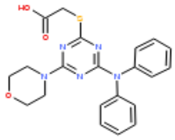			
27519	-3.3	50.0	27519	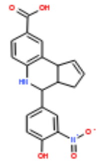	6.1		True
28029	-5.4	22.0	28029,27175,27183,27233,27203,27245,30081,30061	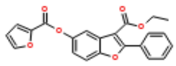		artifact, fluorescence bleaching	True
28764	-0.3	91.0	28764	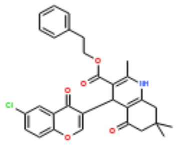			
28824	0.2	100.0	28824,30331,25365	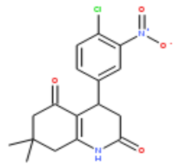			
29189	1.4	106.0	29189,29219	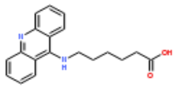			

29191	-0.5	89.0	29191,29195,29197	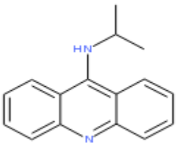			
29193	0.9	101.0	29193	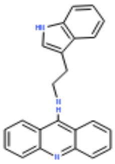			
29195	2.9	119.0	29195,29191,29197	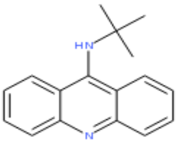			
29197	2.6	116.0	29197,29195,29191	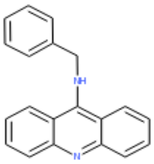			
29199	-8.7	16.0	29199,30927,29219	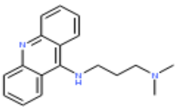			
29219	-1.1	83.0	29219,29199,29189	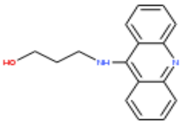			
29582	-9.8	10.0	29582,38442,31017,101707	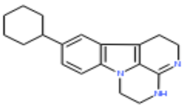			
29953	-146.6	-1233.0	29953,36213,36211,36247,29955,29963,29957,36209,36527,36249,36245,29961,36273,36519,36525,36243	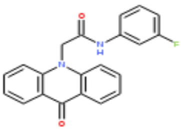		artifact, fluorescence bleaching	True
29955	-0.3	89.0	29955,36243,29953,36241,29957,29961,36213,36519,36525,36211,36247,29963,29965	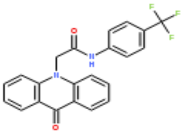			
29957	0.1	93.0	29957,36223,36215,36221,29953,29955,36219,36273,36217,36249	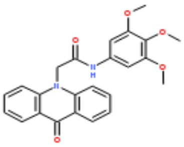	40		True
29959	-0.9	85.0	29959	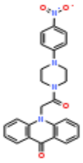			

29961	-9.3	9.0	29961,29985,29987,29965,29963,29955,29953	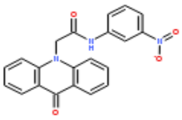			
29963	0.8	100.0	29963,29961,29965,29985,29987,29953,29955,36211,36247	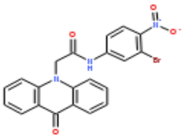			
29965	0.4	96.0	29965,29961,29985,29987,29963,29955	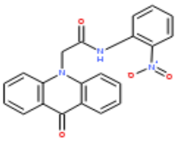			
29985	-2.1	73.0	29985,29961,29987,29965,29963	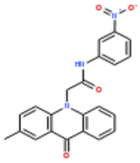			
29987	-1.2	82.0	29987,29985,29961,29965,29963	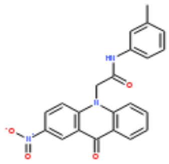			
30059	8.0	165.0	30059,30081,30061,30083,30063,30085	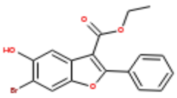			
30061	-9.2	10.0	30061,30081,30083,30085,30063,27175,27183,30059,27245,27203,27233,28029	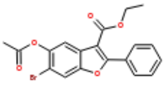	31		True
30063	-23.0	-115.0	30063,30081,30083,27233,30061,30085,30059,27175,27203	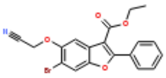		artifact, fluorescence bleaching	True
30067	-0.2	91.0	30067	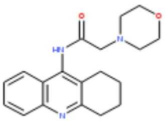			
30081	-2.0	75.0	30081,30061,30085,30083,30063,30059,27175,27245,27183,27233,27203,28029	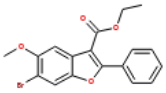		artifact, fluorescence bleaching	True
30083	-17.1	-62.0	30083,30085,30061,30081,30063,27203,27175,30059,27183,27233	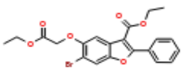		artifact, fluorescence bleaching	True

30085	-26.7	-149.0	30085,30083,30081,30061,30063,27203,27175,30059,27183,27233,27245	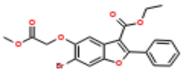		artifact, fluorescence bleaching	True
30331	-0.6	90.0	30331,25329,25541,25343,28824,25365,25543,39547	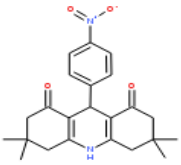			
30575	2.7	116.0	30575,30595,30593,31357,31359,25551,25575,25609,25549	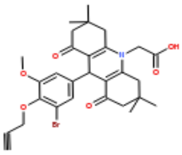			
30593	-0.8	85.0	30593,25551,25549,25609,25573,30575,25605,30595,25603,25575,25607,25339	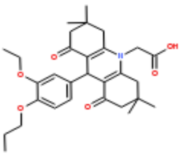			
30595	1.0	101.0	30595,31359,30575,30597,25569,25573,30593,25601	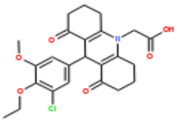			
30597	2.6	115.0	30597,25571,30595,25569,31359,25573	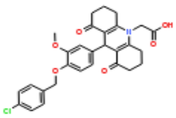			
30713	-2.3	71.0	30713,30747,31862,31868,31840,31894,31860,31447,31443,31870,31958,31864,31892,31866,31411,31872,31618,31391,31421	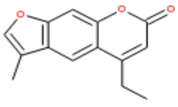	39		True
30747	-0.8	85.0	30747,30713,31868,31840,31892,31864,31447,31862,31443,31958,31860,31894,31890,31870,31411,31618,31391,31421	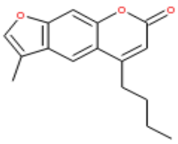			
30927	-8.3	7.0	30927,29199	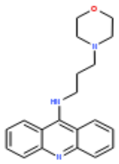			
31017	0.4	100.0	31017,29582	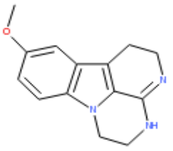			
31357	0.7	103.0	31357,25605,25607,25603,30575,25609,31381,31359	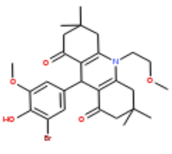			

31359	-22.9	-169.0	31359,30595,30575,30597,25609,31357	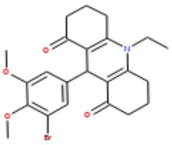				
31379	12.5	239.0	31379,25613,25309,31381,25365,25615,25371,25329,25541,25369,25343,25543	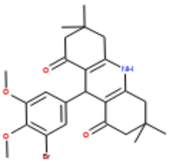				
31381	-1.9	72.0	31381,25615,31379,25613,25365,25309,31357	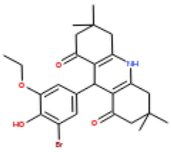				
31391	-4.3	44.0	31391,31409,31443,31447,31411,31840,31868,31441,31421,30713,31445,31862,30747,31417,31870	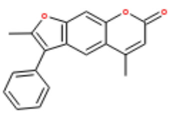				
31409	-0.1	94.0	31409,31391,31413,31858,31423,31443	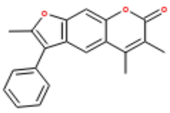				
31411	0.9	105.0	31411,31862,31892,31864,31443,31417,31413,31447,31421,31868,31840,31866,31872,31776,31890,31802,31441,31860,31894,31445,31415,31800,31391,31858,31620,31798,31870,31423,30713,31770,30747,31774,31618	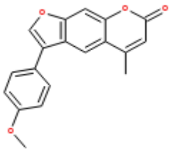		31		True
31413 = Scaff10	-9.0	-9.0	31413,31411,31415,31862,31423,31892,31864,31858,31443,31770,31417,31776,31447,31764,31866,31802,31409,31840,31868,31768,31872,31421,31419,31890,31800,31860,31894	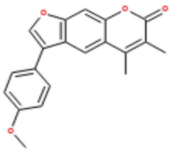		12		True
31415	-1.4	78.0	31415,31413,31872,31419,31890,31441,31445,31620,31411,31870,31766,31862,31423,31864,31892,31858	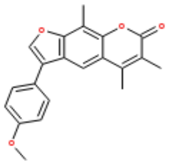				
31417	-2.0	72.0	31417,31866,31411,31421,31798,31443,31862,31770,31860,31864,31892,31894,31447,31800,31413,31868,31423,31840,31776,31802,31796,31872,31441,31890,31445,31768,31391	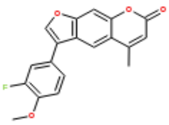				
31419	-0.2	92.0	31419,31423,31415,31441,31768,31421,31445,31870,31860,31894,31413,31766,31872,31890,31858	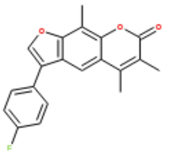				
31421	-0.5	89.0	31421,31894,31860,31443,31423,31417,31447,31411,31796,31866,31868,31840,31768,31862,31800,31441,31864,31892,31419,31445,31391,31858,31413,31798,31870,30713,31770,30747	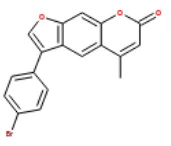				

31423	0.2	97.0	31423,31768,31419,31421,31894,31860,31413,31858,31443,31796,31770,31417,31447,31866,31409,31800,31840,31415,31411,31868,31764,31862,31864,31892	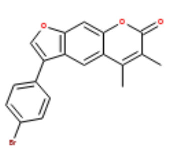		artifact, activator	True
31441	-1.8	74.0	31441,31445,31870,31443,31872,31447,31890,31411,31415,31840,31868,31419,31421,31391,31620,31862,31804,31417,31860,31864,31892,31894,31858,31766	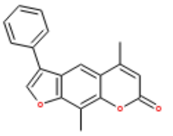	21		True
31443	-0.2	92.0	31443,31447,31840,31868,31411,31421,31441,31862,31391,31417,31892,31864,31860,31894,31445,31858,31870,31413,31866,31423,30713,31776,31802,31796,30747,31774,31872,31890,31409,31798,31768	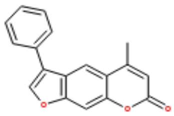			
31445	-0.0	94.0	31445,31441,31447,31870,31872,31443,31890,31415,31411,31421,31419,31620,31840,31868,31862,31417,31804,31892,31894,31864,31860,31391	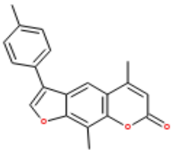			
31447	-1.2	81.0	31447,31443,31445,31411,31421,31868,31840,31441,31862,31417,31391,31894,31892,31864,31860,31870,30713,31858,31413,31866,30747,31423,31872,31776,31890,31802,31796,31774	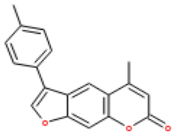			
31451	-7.7	5.0	31451	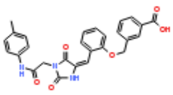			
31481	164.0	1985.0	31481,31483,31487,26897,26901,26909,26911	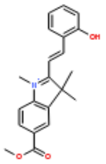	33		True
31483	142.3	1735.0	31483,31481,26897,31487,26901,26909,26911	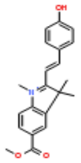	26		True
31487	-4.7	40.0	31487,26909,26897,26911,26901,31481,31483	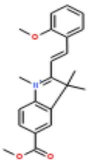	28		True
31618	0.1	94.0	31618,31958,31776,31802,31962,31620,31960,31800,31798,31956,31774,30713,31796,30747,31411	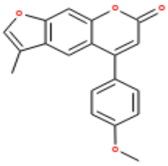			
31620	0.6	100.0	31620,31776,31802,31804,31800,31798,31618,31872,31962,31890,31415,31774,31445,31796,31411,31441,31956,31862,31870,31958,31864,31892	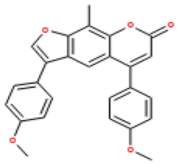			

31764	-8.6	-4.0	31764,31766,31768,31858,31770,31413,31774,31423,31840,31868,31864,31892,31862	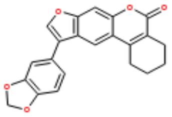	10		True
31766	-0.5	87.0	31766,31764,31832,31415,31804,31768,31419,31890,31858,31770,31870,31872,31441	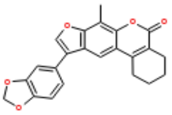			
31768	3.5	132.0	31768,31423,31770,31858,31764,31894,31860,31421,31419,31413,31796,31766,31840,31868,31866,31443,31800,31417,31864,31892	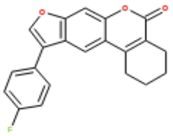			
31770	-4.2	46.0	31770,31768,31866,31417,31764,31413,31858,31423,31798,31892,31864,31862,31766,31800,31411,31860,31894,31421	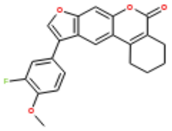	24		True
31774	-0.6	86.0	31774,31804,31802,31776,31796,31798,31800,31620,31958,31764,31443,31618,31447,31868,31840,31411,31960	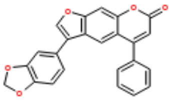			
31776	0.5	99.0	31776,31802,31800,31798,31620,31774,31796,31618,31411,31862,31958,31804,31864,31892,31413,31443,31417,31447,31866,31840,31868,31960,31962	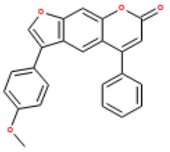			
31796	-1.7	74.0	31796,31800,31798,31776,31802,31774,31421,31894,31860,31958,31423,31620,31443,31804,31417,31768,31447,31866,31840,31868,31618,31962	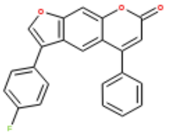			
31798	-0.1	92.0	31798,31800,31776,31802,31796,31417,31866,31774,31620,31770,31618,31804,31411,31958,31421,31862,31443,31860,31864,31892,31894	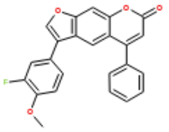			
31800	0.2	95.0	31800,31796,31798,31776,31802,31620,31417,31618,31774,31421,31866,31411,31860,31894,31862,31423,31864,31892,31413,31770,31804,31958,31768	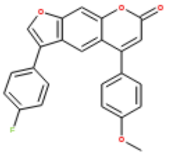	27		True
31802	-5.6	30.0	31802,31776,31800,31798,31774,31620,31796,31618,31411,31804,31862,31958,31864,31892,31413,31443,31417,31447,31866,31840,31868,31960,31962	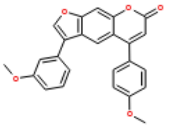			
31804	-1.2	79.0	31804,31774,31620,31802,31962,31776,31796,31766,31798,31441,31445,31956,31800,31870,31958	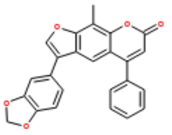			

31832	-0.5	87.0	31832,31766	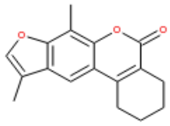			
31840	-0.6	86.0	31840,31868,31892,31864,31443,31862,31860,31894,31447,31858,30747,31870,31411,31421,31890,31866,31441,30713,31417,31391,31872,31445,31413,31423,31768,31776,31764,31802,31796,31774	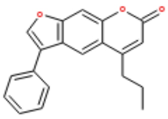			
31858	-1.1	81.0	31858,31868,31840,31768,31413,31443,31764,31423,31770,31892,31864,31447,31862,31409,31411,31860,31894,31421,31766,31415,31870,31441,31419,31866	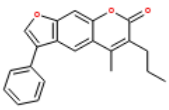		artifact, fluorescence bleaching	True
31860	-0.9	83.0	31894,31860,31421,31866,31862,31868,31423,31840,31443,31417,31892,31864,31768,31447,31796,31411,31870,30713,31800,31872,31419,31858,30747,31441,31890,31445,31413,31770,31798	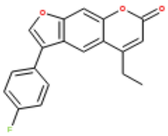			
31862	-2.1	69.0	31862,31892,31864,31411,31866,31872,31868,31840,31894,31860,31413,31443,31890,31417,31447,31870,30713,31776,31421,31802,31858,30747,31800,31415,31441,31445,31770,31620,31798,31423,31391,31764	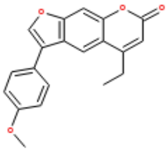			
31864	-8.2	1.0	31892,31864,31862,31411,31868,31840,31890,31866,31872,31413,31443,31417,31894,31860,31447,30747,31776,31421,31858,31802,31870,30713,31770,31800,31415,31441,31764,31445,31620,31798,31423,31768	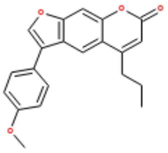	17		True
31866	-1.7	74.0	31866,31417,31862,31894,31860,31892,31864,31411,31770,31798,31421,31868,31840,31872,31443,31800,31447,31890,31413,31870,31423,31776,30713,31802,31796,31768,31858	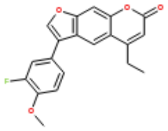			
31868	1.1	106.0	31840,31868,31892,31864,31443,31862,31860,31894,31447,31858,30747,31870,31411,31421,31890,31866,31441,30713,31417,31391,31872,31445,31413,31423,31768,31776,31764,31802,31796,31774	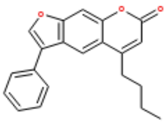			
31870	1.0	104.0	31870,31441,31872,31445,31890,31868,31840,31443,31862,31894,31860,31447,31415,31892,31864,31419,30713,31866,31411,31421,31858,31620,30747,31804,31766,31391	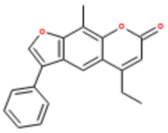			
31872	1.0	104.0	31872,31890,31870,31862,31415,31441,31864,31892,31445,31411,31620,31866,31840,31868,31860,31894,31447,31413,31443,31417,31419,30713,31766	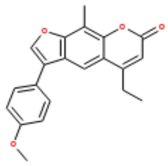	14		True
31890	0.1	94.0	31890,31872,31892,31864,31870,31862,31415,31441,31445,31868,31411,31840,31620,31866,31447,31413,31443,30747,31766,31417,31860,31894,31419	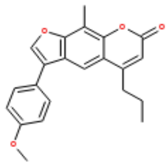			

31892	-11.8	-39.0	31892,31864,31862,31411,31868,31840,31890,31866,31872,31413,31443,31417,31894,31860,31447,30747,31776,31421,31858,31802,31870,30713,31770,31800,31415,31441,31764,31445,31620,31798,31423,31768	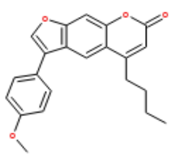	11		True	
31894	0.3	96.0	31894,31860,31421,31866,31862,31868,31423,31840,31443,31417,31892,31864,31768,31447,31796,31411,31870,30713,31800,31872,31419,31858,30747,31441,31890,31445,31413,31770,31798	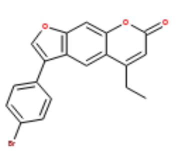				
31956	0.9	103.0	31956,31962,31958,31618,31620,31804,31960	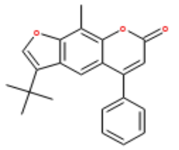				
31958	-0.2	90.0	31958,31618,31962,31960,31956,31776,31796,31802,31774,30713,30747,31798,31620,31800,31804	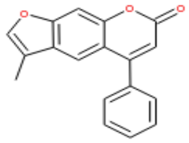				
31960	-2.7	62.0	31960,31958,31618,31962,31776,31956,31802,31774	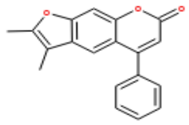	37		True	
31962	1.2	106.0	31962,31956,31958,31618,31620,31804,31960,31776,31796,31802	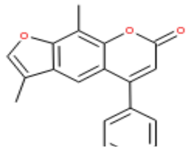				
32431	-4.0	57.0	32431,35820	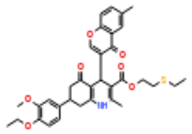	20		True	
32662	1.7	109.0	32662,101343,101291,101346,101347,101344	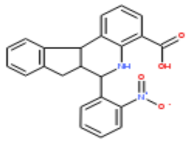				
34444	-2.1	66.0	34444,25339	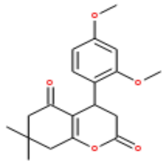				
34714	-7.1	3.0	34714	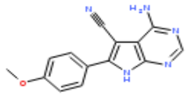				
35673	-0.2	89.0	35673,35701,35705	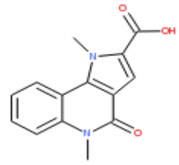				

35699	0.8	103.0	35699	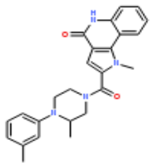			
35701	1.8	118.0	35701,35703,35733,35711,35673,35707	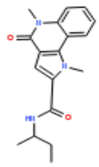			
35703	0.2	95.0	35703,35701	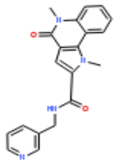			
35705	-0.4	87.0	35705,35673	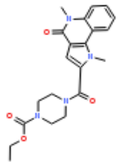			
35707	-6.1	4.0	35707,35701	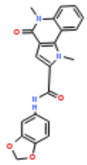			
35709	1.1	108.0	35709	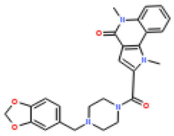			
35711	-0.7	82.0	35711,35729,35701,35733	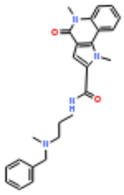			
35729	0.8	104.0	35729,35711	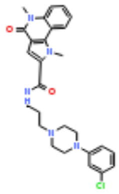			
35733	-0.6	84.0	35733,35701,35711	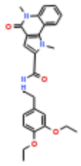			
35820	-6.4	0.0	35820,32431,26760,26764	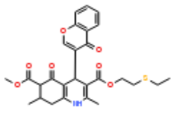			

35857	-3.0	42.0	35857,35891,35955,35925,35961,35897,35953,35861,35862,35865,35895,35967,35921,35889,35871,35867,35959,35859,35927,35997,36123,35903,36127,35901,35863,35964,35870,35902,35991,35999,35893,36089,35869,35933,35965,35864,35963,36087,36179,35987,35935,35931,35923,35899,35985,36145,359	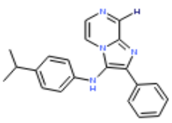			
35858	-0.5	86.0	35858,36181,36179,36183,36185,35991,35989,35868,36085,35863,35870	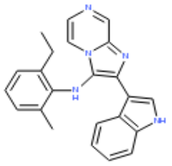			
35859	-1.1	75.0	35859,35861,35867,35897,35891,36149,35870,35863,35893,35857,35987,35991,35985,35931,35899,36091,36063,36061,35929,36125,35961,35869,36151,36181,36049,36051,36083,36087,35871,35889,35959,35921,36017,36019,36153,35935,35997,35989,36059	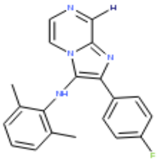			
35861	0.8	109.0	35861,35867,35859,35897,36149,35891,35857,35987,35931,35899,35985,35869,35870,35863,36087,35961,35929,35893,36125,35935,36059,36151,36083,35959,35889,35871,35921,35991,36153,35997,36091,35955,35903,36127,35865,36049,36051,35895,35901,35964,36185,36063,36061,35902,36019,36017,359	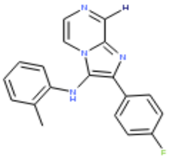			
35862	-0.5	86.0	35862,35864,36063,35868,36083,35857,36059,36115,36147,36061,35955,36085,35891,35925	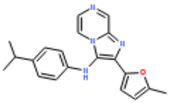			
35863	-2.6	50.0	35863,35889,35865,36091,35901,36087,35933,35959,35921,35871,35859,35997,35891,35895,35870,35893,35927,36089,35925,35935,35861,35989,35903,36127,36181,35867,35857,35991,35999,36093,35869,36123,35858,35963,36063,36061,35897,35961,35923,35899,35931,35985,36145,35957,35965	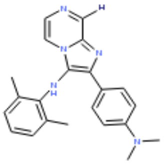			
35864	6.0	198.0	35864,35862,36063,35868,36083,36059,35891,36061,36115,36147,36085,35857,35897,35961	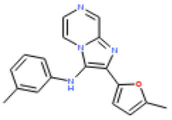			
35865	-1.6	66.0	35865,35933,35889,35895,35863,35927,35901,36089,35999,36123,36093,35959,35921,35871,35923,35925,35899,35931,35997,35985,35957,36145,35891,35965,35857,36087,36119,36091,36127,35903,35967,36125,35929,36117,36121,35963,36177,36183,35897,35961,35935,35953,35995,35861,35955	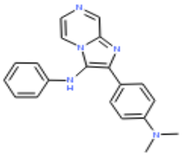			
35867	-16.4	-189.0	35867,35861,35859,35897,36149,35891,35857,35987,35899,35931,35985,36151,35869,36087,35863,35870,36125,35929,36153,35961,35893,35935,36059,36049,36051,36083,35959,35921,35889,35871,35991,35997,36091,35955,35903,36127	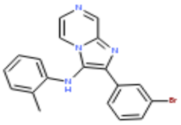			
35868	-1.5	68.0	35868,36063,36085,35862,35864,36061,36059,35991,36083,35858,35870	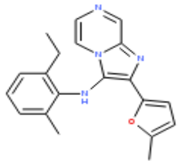			
35869	-1.0	77.0	35869,35891,35861,35867,36087,35863,35857,35859,35935,35871,35889,35921,35959,35870,35893,35897,35961	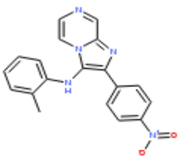			

35870	1.2	115.0	35870,36061,35964,35859,35893,35891,36063,35863,36059,36085,35861,36181,35867,35857,35991,36091,35868,35858,36177,35961,35897,35869,36149	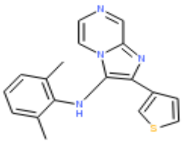			
35871	-6.1	-11.0	35871,35959,35921,35997,35927,35925,35895,35889,35999,35933,36127,35903,35963,35891,35931,35923,35957,35899,35985,36145,36093,35865,35863,35897,35961,35935,35953,36123,35965,35857,35901,35995,36121,36087,36125,36117,36179,35929,36155,36089,35989,36091,35955,35861,36119,35867,361	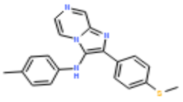			
35877	0.5	104.0	35877,35907,35909	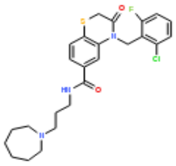			
35889	1.6	122.0	35889,35865,35959,35921,35871,35863,35997,35933,35901,35891,36127,35903,35927,35925,35895,35963,36087,35961,35897,35999,36089,36091,36093,35857,35931,35957,35923,35899,36145,35985,36123,36119,36179,35953,35935,35955,35861,35965,35867,36117,35929,36125,35859,35995,35869,36121	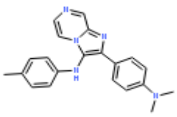		artifact, activator	True
35891	-7.4	-33.0	35891,35857,35961,35897,35959,35921,35871,35889,35997,35861,36127,35903,35867,35859,35955,35895,35865,35864,35870,35967,35863,35963,35893,36179,35927,35925,36123,35869,35964,35901,36087,35902,35999,35991,35935,36089,36059,36091,35933,35965,35862,36063,36061,35987,35953,35985,359	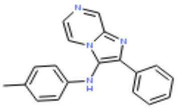			
35893	0.7	108.0	35893,35991,36149,35961,35902,35870,35859,35935,35891,35955,35989,35967,35863,35861,36151,35963,35867,36061,36063,35857,36153,36025,36091,35965,36181,35987,35897,35953,35995,36059,35869	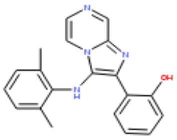			
35895	-7.7	-39.0	35895,35927,35999,35933,35959,35871,35921,35957,35985,35931,35925,35923,35899,36145,35865,35997,36123,36093,35965,35889,36089,35891,35903,36127,36121,35857,35863,35901,35963,35929,36117,36125,35953,35935,36155,35995,36177,35967,36119,36087,36183,35961,35897,35989,36091,35861,359	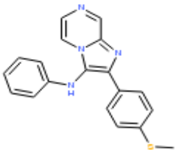			
35897	-22.1	-289.0	35897,35861,35867,35859,35891,35987,35961,35985,35899,35931,35857,35921,35889,35871,35959,35929,36125,35997,36149,36083,35903,36127,36153,35955,36151,35963,35925,35927,35895,35865,35864,35901,35863,35964,35967,36017,36019,35870,36179,36185,35902,35999,35893,36155,35869	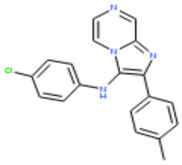			
35899	-41.4	-624.0	35931,35985,35899,35927,35895,35999,35929,36125,35933,36155,35959,35871,35921,35925,35923,36145,35957,35897,35997,36093,35865,35987,36123,35861,35965,35889,35867,35859,36121,35903,36127,35901,35963,36117,36185,36153,35953,36089,35995,36151,35891,36083,36119,35863,36177,35857,359	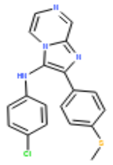			
35901	-2.1	59.0	35901,35865,35889,35995,35863,35933,35964,35902,35895,35871,35959,35921,35927,35997,36089,35925,35891,36087,35857,35999,35931,35899,35985,36093,36091,36123,35903,35965,36127,35963,35897,35953,35935,35957,35923,36145,35861,36119	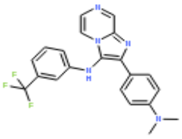			
35902	-2.1	59.0	35902,35995,35964,35893,35901,35961,36151,35955,36149,35967,36153,35891,35965,35963,35857,35987,35953,35935,35991,35897,36155,35861	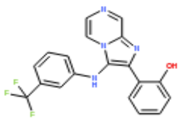			

35903	-1.2	74.0	35903,35997,36127,35957,35923,35959,35871,35921,35929,35889,35999,35891,36123,35927,36115,35925,36145,35895,35963,35897,35961,36117,36125,35933,35865,36119,36177,35857,35863,35899,35931,35985,36121,36087,35901,35935,35953,36089,36147,36091,36093,35955,35861,35965,35867,36157	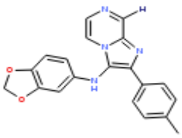			
35905	-9.8	-74.0	35905,35909,35907	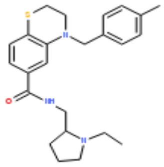			
35907	-4.8	11.0	35907,35905,35909,35877	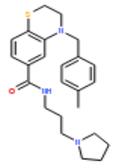			
35909	-11.0	-96.0	35909,35905,35907,35877	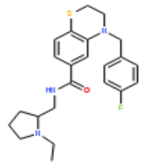			
35921	0.6	104.0	35871,35959,35921,35997,35927,35925,35895,35889,35999,35933,36127,35903,35963,35891,35931,35923,35957,35899,35985,36145,36093,35865,35863,35897,35961,35935,35953,36123,35965,35857,35901,35995,36121,36087,36125,36117,36179,35929,36155,36089,35989,36091,35955,35861,36119,35867,361	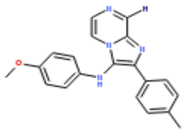			
35923	-0.5	85.0	35957,35923,35999,35927,36145,35895,35903,35929,35997,35933,35921,35871,35959,35899,35925,35931,35985,36123,36093,35865,36127,36121,36117,36125,36115,35965,36177,35889,36119,35963,35953,36155,36089,35891,35901,35863,36157,35857,35935,35995,36147	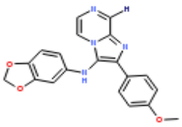			
35925	-20.2	-255.0	35925,35871,35959,35921,35927,35895,35999,35997,35933,35953,35857,36145,35985,35957,35931,35923,35899,35889,35955,36093,35865,36127,35903,35963,35891,36123,35965,35863,35901,35935,35995,36121,36117,36125,35929,35961,35897,36155,36089,35989,35862,36087,36119,36177	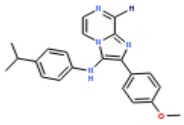			
35927	0.4	102.0	35927,35895,35999,35933,35959,35871,35921,35985,36145,35957,35931,35925,35923,35899,35997,35865,36093,36123,35965,35889,35903,36127,36121,35963,35929,36125,36117,36089,36155,35953,35891,35857,36177,35863,35901,36119,35935,35995,35967,35961,35897,35955,36087,36157,35987,36183	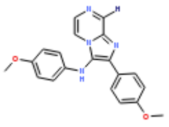			
35929	-1.4	70.0	35929,36125,35985,35957,35931,35923,35899,35903,35999,36123,35897,35927,36145,35997,35895,36127,35987,36117,36115,36155,35933,35861,35865,36119,36177,35959,35921,35871,35867,35859,35925,36121,36159,35889,36153,36089,36093,36151	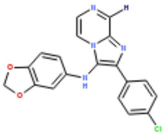			
35931	-0.1	94.0	35931,35985,35899,35927,35895,35999,35929,36125,35933,36155,35959,35871,35921,35925,35923,36145,35957,35897,35997,36093,35865,35987,36123,35861,35965,35889,35867,35859,36121,35903,36127,35901,35963,36117,36185,36153,35953,36089,35995,36151,35891,36083,36119,35863,36177,35857,359	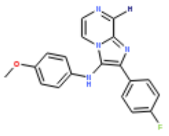			
35933	-0.1	93.0	35933,35927,35865,35895,35999,36093,35959,35921,35889,35871,35899,35923,35925,35931,35985,36145,35957,35997,35863,35901,36089,36123,35965,36119,36127,35903,36121,35963,36117,35929,36125,35953,36155,35891,36087,36177,35857,35935,35995,36091,35967	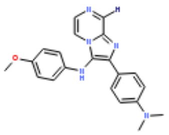			

35934	-2.2	57.0	35934	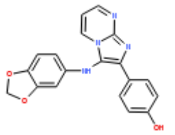			
35935	0.3	100.0	35935,35963,35965,35989,35953,35995,36121,35921,35959,35871,35961,35893,35997,35895,35863,35991,35927,35925,36149,35861,35955,35891,36087,35889,35867,35967,35902,36117,35999,35869,35933,35903,36127,35865,35901,35857,35859,36155,35985,35957,35931,35923,35899,36145,36091	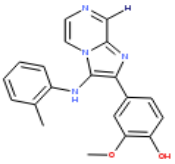			
35953	-0.5	86.0	35953,35963,35965,35935,35995,35925,35955,35989,36121,35857,35921,35871,35959,35961,35927,35997,35895,35999,36117,35933,35889,35967,35902,36155,36145,35923,35931,35985,35899,35957,35891,35903,36127,35865,35901,35991,35987,35893,36093	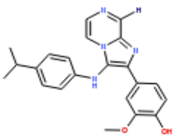			
35955	-49.0	-755.0	35955,35961,35857,35953,35967,35987,35991,35925,35891,35963,35893,36117,35902,35965,35897,35935,35995,35862,35959,35921,35889,35871,36153,35997,35989,35927,36149,35861,36151,36121,35903,36127,35865,35895,35867	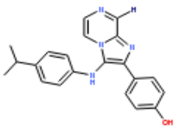		artifact, fluorescence bleaching	True
35957	-3.2	39.0	35957,35923,35999,35927,36145,35895,35903,35929,35997,35933,35921,35871,35959,35899,35925,35931,35985,36123,36093,35865,36127,36121,36117,36125,36115,35965,36177,35889,36119,35963,35953,36155,36089,35891,35901,35863,36157,35857,35935,35995,36147	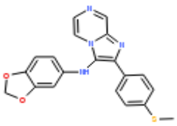			
35959	5.0	181.0	35871,35959,35921,35997,35927,35925,35895,35889,35999,35933,36127,35903,35963,35891,35931,35923,35957,35899,35985,36145,36093,35865,35863,35897,35961,35935,35953,36123,35965,35857,35901,35995,36121,36087,36125,36117,36179,35929,36155,36089,35989,36091,35955,35861,36119,35867,361	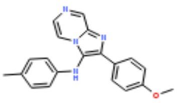			
35961	3.7	158.0	35961,35955,35891,35967,35963,35897,35987,35893,35991,35857,35921,35889,35871,35959,35902,36117,35997,35953,35935,35903,35965,36127,36149,35995,35861,35867,36153,35859,35925,35927,35989,36151,36121,35864,35865,35895,35863,35870,36179,35999,35869	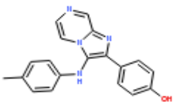			
35963	2.3	134.0	35963,35935,35953,35965,35995,35921,35871,35959,35989,35961,36121,35997,35889,35927,35925,35891,35955,35895,35903,36127,35999,36117,35933,35967,35902,35897,35893,36155,35985,35957,35931,35923,35899,36145,35865,35901,35863,35857,35991,35987,36093,36123	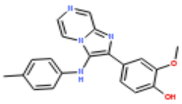			
35964	-0.0	94.0	35964,35902,35870,35901,35891,35995,35857,36177,35897,35861	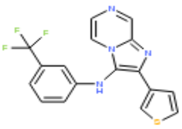			
35965	3.3	151.0	35965,35963,35953,35935,35995,36121,35895,35989,35927,35967,35999,36117,35933,35865,35871,35921,35959,35961,35957,35985,35931,35925,35923,35997,35899,36145,36155,35955,36123,35902,35987,36093,35891,35901,35889,35857,35893,36089,36151,35903,36127,35863	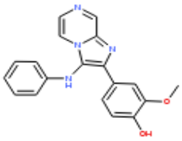			
35967	0.2	98.0	35967,35987,35961,35955,35965,36117,35991,35891,36151,35857,36153,35893,35902,35865,35895,35963,35953,35935,35927,35995,36123,36121,35999,35897,36089,36149,35933,35861	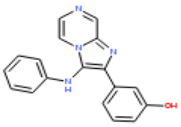			

35985	-3.4	36.0	35931,35985,35899,35927,35895,35999,35929,36125,35933,36155,35959,35871,35921,35925,35923,36145,35957,35897,35997,36093,35865,35987,36123,35861,35965,35889,35867,35859,36121,35903,36127,35901,35963,36117,36185,36153,35953,36089,35995,36151,35891,36083,36119,35863,36177,35857,359	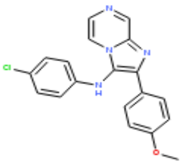			
35987	0.8	109.0	35987,36153,36151,35967,35961,35897,35955,35985,35899,35931,36155,35861,36149,35867,35859,35929,36125,36117,35965,35902,35891,36083,35963,35857,35991,36185,35893,35953,35995,35927	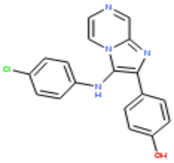			
35989	6.0	199.0	35989,35935,35963,35991,35953,35965,35995,35893,36121,35863,35858,35959,35921,35871,35961,35925,35997,35955,35895,35859	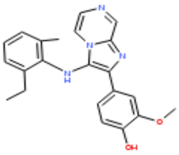			
35991	3.4	153.0	35991,35989,35893,35961,35955,35967,35859,35935,36085,35868,35891,35858,35863,35870,35857,36149,35861,35963,35867,35902,35987,35953,36091	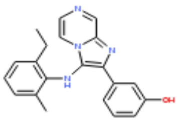			
35995	3.4	154.0	35995,35965,35963,35953,35935,35902,35901,35989,36121,35895,35921,35871,35964,35959,35961,35997,35925,35927,35955,35967,36117,35999,36155,35931,35985,35899,35933,35891,35865,35889,35857,35987,35893,35957,35923,36145	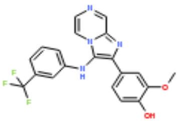			
35997	-1.1	75.0	35997,35959,35871,35921,36127,35903,35999,35889,35927,35957,35925,35923,36145,35895,35963,35891,36123,35933,35899,35985,35931,35865,36121,35863,35961,35897,36117,35929,36125,35953,35935,36093,35965,35857,36177,35901,36119,35995,36087,36179,36115,36147,36089,35989,36091,35955,358	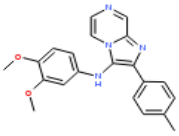			
35999	-0.8	80.0	35999,35927,36145,35923,35957,35895,35997,35933,35921,35871,35959,35899,35925,35931,35985,36123,35865,36093,35903,36127,36121,36125,35929,36117,35965,35889,36177,36119,35963,36089,35953,36155,35891,35901,36157,35863,35857,35935,36115,36147,35995,36159,35967,35961,35897	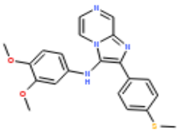			
36017	1.2	116.0	36017,36019,36049,36051,36029,36025,36021,36027,36031,35859,35897,35861	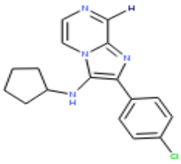			
36019	-1.6	66.0	36017,36019,36049,36051,36029,36025,36021,36027,36031,35859,35897,35861	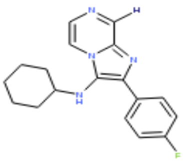			
36021	-4.0	26.0	36021,36023,36017,36019,36049,36051	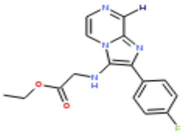			
36023	-0.5	86.0	36023,36021,36027	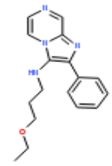	34		True

36025	0.2	99.0	36025,36029,36049,36051,36017,36019,35893,36031	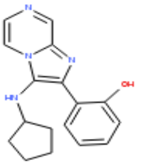			
36027	-0.2	92.0	36027,36089,36091,36093,36087,36119,36017,36019,36049,36051,36023	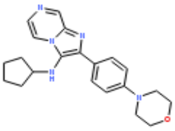			
36028	-0.9	79.0	36028,36083	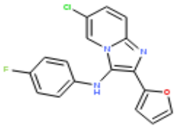			
36029	0.5	103.0	36029,36025,36049,36051,36017,36019,36153,36151,36149,36155	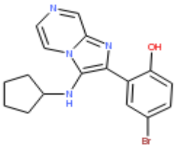			
36031	-0.2	91.0	36031,36017,36019,36025	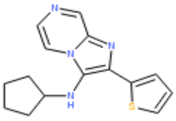			
36049	0.7	107.0	36049,36051,36017,36019,36029,36025,35867,35859,36021,36027,35861	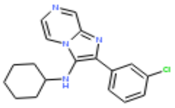			
36051	-7.7	-38.0	36049,36051,36017,36019,36029,36025,35867,35859,36021,36027,35861	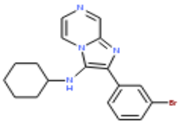			
36059	1.3	118.0	36059,36061,36085,36063,35864,35868,35862,35870,35861,35891,35867,36157,36083,35857,35859,35893	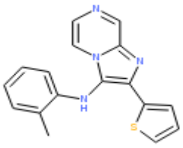			
36061	-0.3	89.0	36061,36059,36085,36063,35868,35870,35864,35862,35859,35893,35891,35863,36157,35861	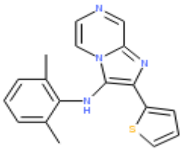			
36063	-0.4	88.0	36063,35868,35864,36061,35862,36059,36085,36083,35870,35859,35893,36115,36147,35891,35863,35861	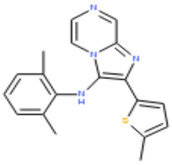			
36083	-5.4	0.0	36083,35862,35864,36063,35897,36147,36115,35861,35868,35867,36153,35987,35859,35985,35931,35899,36059,36151,36028,36159	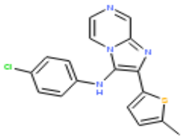			

36085	-0.1	93.0	36085,36061,35868,36059,36063,35870,35991,35862,35864,35858	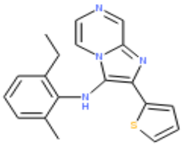			
36087	0.7	106.0	36087,36091,36089,36093,35863,36119,35889,35865,35891,35861,36127,35901,35921,35871,35959,35867,35935,35997,36027,35869,35933,36123,35903,35895,35857,35859,35925,35927	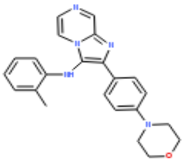			
36089	2.0	129.0	36089,36093,36087,36119,36091,35865,35933,36123,35895,35889,35927,36027,35863,35901,35999,36145,35891,36127,35921,35959,35871,35857,36117,36125,35985,35997,35957,35931,35925,35923,35899,35965,35903,36177,35967,35929	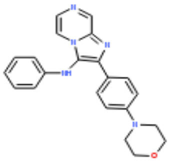			
36091	0.1	97.0	36091,36087,36089,35863,36093,36119,35889,35865,35859,36027,35891,36127,35901,35959,35921,35871,35870,35893,35997,35933,35861,36123,35903,35895,35867,35857,35991,35935	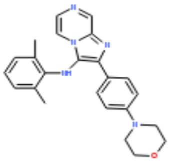			
36093	0.5	103.0	36093,36089,36119,35933,35927,35895,35999,36087,36145,35865,36091,35921,35871,35959,35899,35923,35925,35931,35957,35985,35889,35997,36123,36027,36121,35965,36127,35901,35863,36125,36117,35903,35963,36177,35929,35953,36155	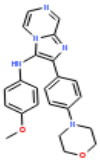			
36115	-1.3	73.0	36115,36147,35903,36157,35923,35957,36083,35864,35862,36159,35929,36063,35999,35997,36123,36127,36177,36145	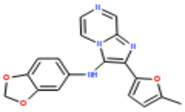			
36117	1.4	119.0	36117,36121,36123,36145,36127,36125,36177,35999,36119,35965,35967,35961,35927,35923,35997,35957,35955,35895,35903,36157,35963,35987,35929,36147,35953,35933,36159,35865,35921,35871,35959,35935,36155,36089,35899,35931,35925,35985,35995,36093,35889	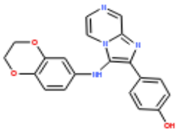			
36119	0.8	108.0	36119,36089,36093,36123,36145,36127,36087,36125,36117,36091,36177,35999,35933,35865,36121,35927,35957,35997,35923,35895,35903,35889,36157,35929,36027,36147,36159,35921,35959,35871,35925,35985,35899,35931,35901	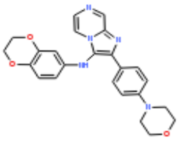			
36121	1.1	114.0	36121,36117,35965,36145,35963,36123,35953,35999,35935,35995,36127,36125,35927,35997,35923,35957,35895,36177,36119,35933,35989,35959,35871,35921,36155,35985,35931,35899,35925,36093,35903,35865,36157,35967,35929,35961,36147,35955,36159,35889	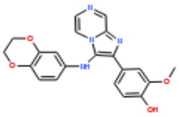			
36123	-2.2	56.0	36123,36145,36127,36117,36125,36177,35999,36119,35895,36121,35927,35923,35957,35997,35865,35903,36157,35929,36089,36147,35933,36159,35871,35959,35921,35985,35931,35925,35899,35891,36093,35965,35857,35889,36087,35901,35863,35967,36115,36091,35963	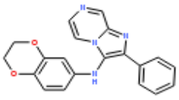			
36125	-4.1	24.0	36125,35929,36123,36145,35985,35931,35899,36127,36117,36177,35999,36119,36159,36121,35897,35927,35923,35957,35997,35895,35903,36157,35987,36147,36155,35933,35861,35865,35959,35871,35867,35921,35859,36089,35925,36093,35889,36153,36151	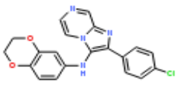			

36127	-0.8	80.0	36127,35997,36123,35903,36145,35959,35871,35921,36125,36117,36177,35889,35999,36119,35891,36121,35927,36147,35957,35925,35923,35895,36157,35963,35961,35897,35929,35933,35865,36159,35857,35863,36087,36089,35899,35985,35931,36091,36093,35901,35953,35935,36115,35861,35955,35965,358	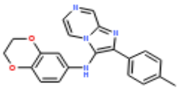			
36145	0.4	101.0	36145,35999,36123,35927,35957,35923,35895,36127,36121,36117,36125,35997,35933,35959,36177,35871,35921,36119,35985,35899,35925,35931,36093,35865,35903,36157,35929,36147,35965,36159,35889,36089,35963,35953,36155,35891,35901,35863,35857,35935,36115,35995	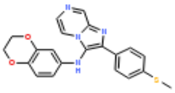			
36147	0.6	104.0	36147,36115,36157,36159,36123,36127,36177,36145,36083,35864,35862,36117,36125,36119,36063,35999,35997,36121,35903,35957,35923	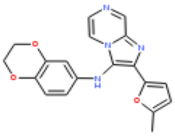			
36149	-0.9	79.0	36149,36151,35867,36153,35861,35893,35859,36155,35987,35897,35902,35961,35935,36029,35991,35955,35891,35967,35870	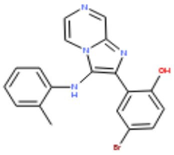			
36151	0.1	96.0	36151,36153,36149,35987,36155,35967,35902,35867,36029,35893,35897,35861,36185,35961,35859,35985,35931,35899,35955,35965,36083,36125,35929	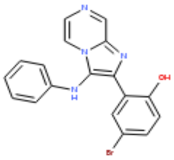			
36153	-0.3	90.0	36153,36151,35987,36155,36149,35967,36029,35897,35902,35867,35893,35961,35899,35931,35985,35955,35861,36083,36185,36125,35929,35859	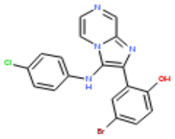			
36155	-0.8	81.0	36155,36153,35899,35931,35985,36151,36149,35987,35927,35895,35965,35999,36125,35929,35933,36121,35963,35871,35921,35959,36117,35953,35925,35923,36145,35957,35995,36029,35902,35897,35935,35997,36093	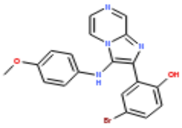			
36157	0.1	96.0	36157,36147,36159,36177,36123,36145,36115,36127,36125,36117,36119,35999,36059,36121,36061,35997,35957,35923,35927,35903	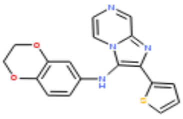			
36159	-1.5	68.0	36159,36157,36147,36125,36123,36177,36115,36145,36127,36117,36119,35929,35999,36121,36083	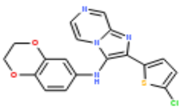			
36177	0.6	106.0	36177,36123,36145,36127,36157,36117,36125,36119,35999,36147,36159,36121,35927,35923,35957,35997,35903,35895,35929,35933,35865,35964,35959,35921,35871,35870,36115,36089,35985,35931,35925,35899,36093	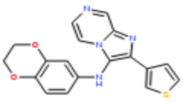			
36179	19.2	428.0	36179,36181,36183,35858,36185,35891,35871,35889,35921,35959,35997,35857,35897,35961	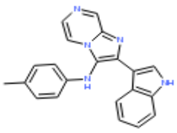			

36181	-9.0	-61.0	36181,35858,36179,36183,36185,35863,35870,35859,35893,35891	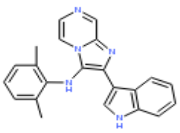	15		True
36183	2.8	143.0	36183,36179,36185,36181,35858,35865,35895,35927	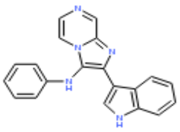			
36185	19.8	438.0	36185,36183,36179,36181,35858,35899,35931,35985,36151,35897,35987,36153,35861	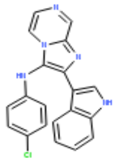			
36209	0.1	93.0	36527,36209,36245,36213,36247,36211,36241,36249,36255,36273,36251,36519,36525,36243,29953,36215,36523,36217,36253,36517,36223,36221,36219	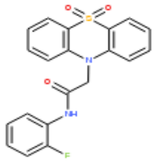			
36211	0.4	99.0	36247,36211,36213,36527,36209,29953,36249,36245,36273,36525,36519,36243,36241,36223,36215,36255,36251,36253,36221,36523,36517,36217,29955,36219,29963	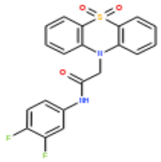			
36213	-0.5	82.0	36213,36247,36211,36527,36209,29953,36249,36245,36525,36519,36243,36241,36215,36273,36255,36251,36253,36223,36221,36523,36217,36517,29955,36219	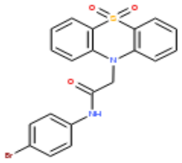			
36215	-1.4	68.0	36215,36221,36219,36223,36217,36273,36255,36525,36519,29957,36213,36251,36253,36247,36243,36211,36241,36249,36209,36527,36523,36517,36521,36245	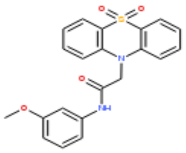			
36217	0.6	102.0	36217,36219,36215,36221,36249,36223,36255,36273,36519,36525,36251,36241,36527,36209,36213,36523,36253,36247,36517,36243,36211,36245,29957	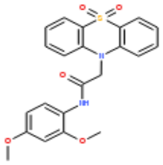			
36219	-3.2	38.0	36219,36217,36215,36221,36223,36249,36273,36255,29957,36525,36523,36519,36251,36521,36241,36527,36213,36209,36253,36247,36517,36243,36211,36245	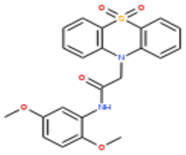			
36221	-1.1	72.0	36221,36215,36219,36223,36217,36273,36255,29957,36525,36519,36249,36213,36523,36251,36253,36247,36243,36211,36521,36241,36209,36527,36517,36245	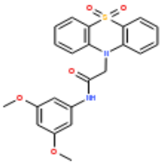			
36223	-2.0	58.0	36223,36215,36221,29957,36273,36219,36217,36249,36255,36525,36519,36247,36211,36521,36213,36251,36523,36243,36253,36241,36209,36527,36517,36245	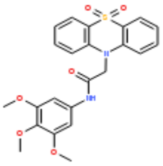			

36241	1.4	115.0	36241,36243,36527,29955,36209,36213,36525,36519,36251,36211,36247,36245,36215,36255,36273,36523,36253,36517,36217,36223,36221,36219,36249	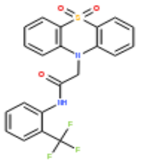			
36243	1.3	114.0	36243,36241,29955,36525,36213,36519,36211,36247,36527,36273,36255,36215,36209,36251,36253,36245,36223,36221,36523,36217,36517,36219,29953	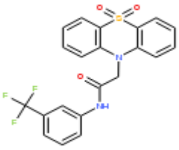			
36245	-0.0	91.0	36245,36527,36209,36213,36247,36211,36525,36523,36241,36255,36273,36519,36251,36243,36249,36215,29953,36517,36253,36217,36223,36221,36219	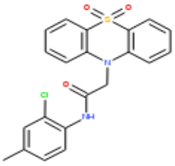			
36247	-0.1	90.0	36247,36211,36213,36527,36209,29953,36249,36245,36273,36525,36519,36243,36241,36223,36215,36255,36251,36253,36221,36523,36517,36217,29955,36219,29963	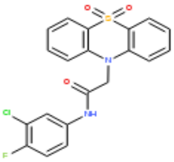			
36249	-0.9	76.0	36249,36213,36219,36211,36217,36247,36527,36209,36223,36215,36221,29953,36245,36273,36241,29957,36255,36251,36519,36523,36525	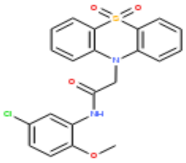			
36251	0.1	94.0	36251,36525,36519,36241,36527,36273,36255,36209,36213,36215,36523,36211,36517,36217,36253,36247,36243,36245,36219,36221,36223,36275,36249,36521	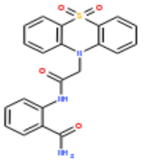			
36253	-0.0	91.0	36253,36255,36525,36519,36213,36215,36273,36251,36211,36243,36247,36241,36527,36223,36221,36209,36523,36517,36217,36245,36219	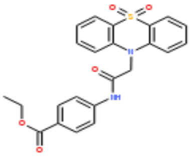			
36255	-1.2	72.0	36255,36215,36273,36221,36525,36519,36217,36253,36527,36223,36219,36213,36209,36251,36243,36247,36211,36241,36245,36523,36517,36521,36249	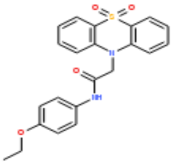			
36273	0.6	102.0	36273,36255,36223,36215,36221,36519,36525,36211,36247,36213,36219,36209,36527,36251,36217,36243,36253,36241,36521,36245,29957,36523,36517,36249,29953	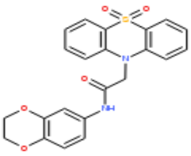			
36275	-0.3	87.0	36275,36517,36251,36519,36523,36525	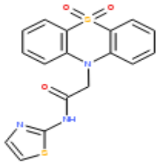			
36297	0.5	100.0	36297	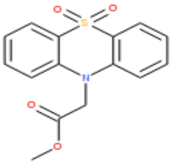			

36361	-4.9	8.0	36361,36363,36365	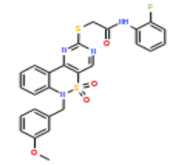		artifact, fluorescence bleaching	True
36363	-1.4	69.0	36363,36365,36361	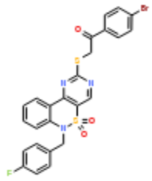			
36365	1.2	112.0	36365,36363,36361	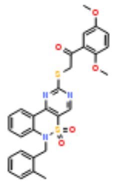			
36466	-27.2	-371.0	36466,36476,36472,36474,36468,36470,36530,36506,36512	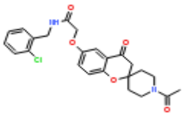			
36468	-0.1	89.0	36468,36466,36472,36476,36474,36470,36530	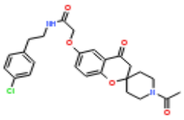			
36470	-2.1	56.0	36470,36472,36474,36476,36466,36468,36506	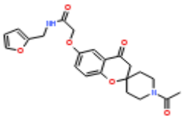			
36472	-25.2	-336.0	36472,36474,36476,36466,36470,36468,36506,36512,36504,36530	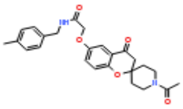			
36474	-2.1	56.0	36474,36472,36476,36466,36470,36468,36506,36512,36504,36530	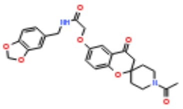			
36476	-0.7	80.0	36476,36472,36466,36474,36470,36468,36506,36512,36504,36530	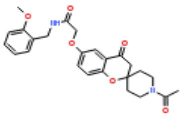	48		True
36504	4.4	166.0	36504,36506,36512,36536,36542,36530,36538,36534,36544,36472,36476,36474	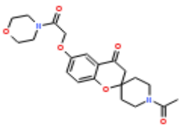			
36506	-4.6	13.0	36506,36504,36536,36512,36542,36530,36538,36534,36544,36472,36476,36474,36466,36470	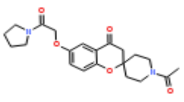			

36512	-2.3	53.0	36512,36506,36504,36530,36534,36536,36542,36538,36544,36472,36476,36474,36466	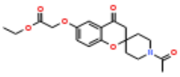			
36517	0.1	94.0	36517,36525,36519,36275,36251,36241,36527,36273,36255,36215,36213,36209,36523,36243,36247,36253,36217,36211,36245,36223,36221,36219	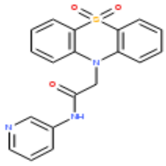			
36519	0.2	96.0	36519,36525,36213,36215,36273,36255,36251,36211,36253,36243,36247,36241,36527,36223,36221,36209,36523,36217,36517,36245,36219,29955,36275,29953,36249,36521	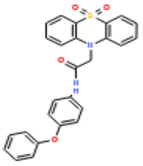			
36521	-5.2	3.0	36521,36223,36273,36215,36219,36221,36255,39687,36251,36519,36523,36525	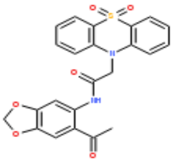			
36523	-0.2	88.0	36523,36525,36245,36519,36251,36241,36527,36273,36255,36223,36221,36219,36215,36213,36209,36243,36247,36253,36217,36517,36211,36275,36249,36521	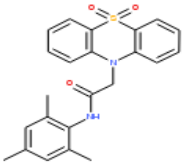			
36525	0.8	105.0	36525,36519,36243,36213,36215,36273,36255,36523,36251,36211,36253,36247,36241,36245,36527,36223,36221,36209,36517,36217,36219,29955,36275,29953,36249,36521	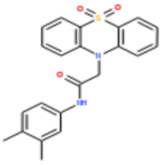			
36527	0.6	102.0	36527,36209,36245,36213,36247,36211,36241,36249,36255,36273,36251,36519,36525,36243,29953,36215,36523,36217,36253,36517,36223,36221,36219	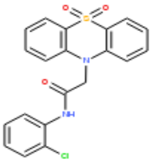			
36530	-0.5	84.0	36530,36506,36512,36504,36536,36542,36472,36466,36538,36534,36476,36474,36468	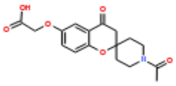			
36534	-5.9	-8.0	36534,36542,36536,36512,36538,36506,36544,36504,36530	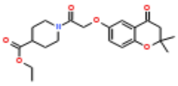			
36536	-1.9	60.0	36536,36542,36506,36538,36534,36504,36512,36544,36530	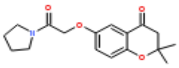			
36538	-10.8	-92.0	36538,36536,36542,36534,36506,36504,36512,36544,36530	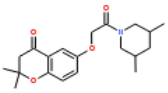			

36540	-10.2	-82.0	36540	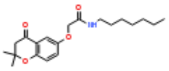			
36542	-4.1	23.0	36542,36536,36534,36544,36538,36506,36504,36512,36530	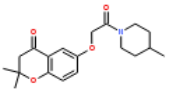			
36544	-1.6	64.0	36544,36542,36536,36534,36538,36506,36512,36504	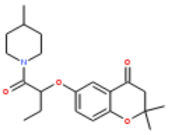			
38039	-4.2	38.0	38039	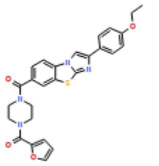			
38043	0.2	101.0	38043,38073	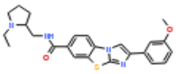			
38058	1.0	112.0	38058,38355,38060,38062,38321,38323,38327,38329,38357,38361,38333,38325,38335,38363,38353,38359,38331	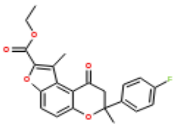			
38060	-7.9	-13.0	38060,38062,38058,38327,38355,38329,38357,38321,38323,38325,38361,38363,38353,38333,38335,38359,38331	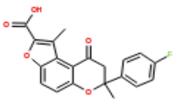			
38062	-5.2	24.0	38060,38062,38058,38327,38355,38329,38357,38321,38323,38325,38361,38363,38353,38333,38335,38359,38331	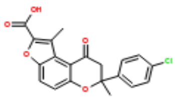		artifact, fluorescence bleaching	True
38067	-2.7	59.0	38067,38073	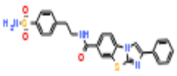			
38073	-6.0	13.0	38073,38043,38067	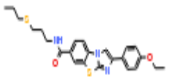		artifact, fluorescence bleaching	True

38242	0.9	110.0	38242,38244,38246	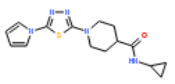			
38244	0.6	106.0	38244,38242,38246,38248	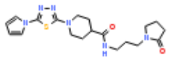			
38246	0.6	106.0	38246,38244,38242	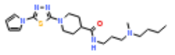			
38248	-6.4	8.0	38248,38244	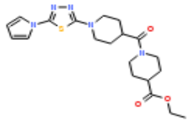			
38321	-3.4	51.0	38321,38323,38355,38058,38325,38335,38060,38062,38331,38359,38353,38363,38327,38329,38357,38361	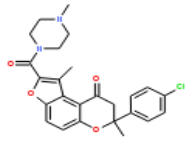			
38323	-2.2	67.0	38323,38321,38355,38058,38335,38331,38359,38060,38062,38353,38363,38325,38327,38329,38357,38361	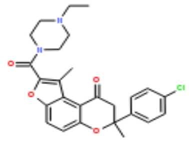			
38325	-1.6	75.0	38325,38335,38363,38353,38357,38329,38359,38331,38327,38361,38333,38321,38062,38060,38323,38058,38355	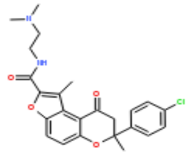			
38327	-5.1	26.0	38327,38357,38329,38361,38325,38363,38353,38333,38060,38062,38335,38058,38331,38359,38355,38323,38321,38717,38985	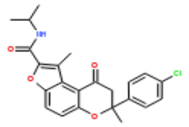	4.8		True
38329	-3.3	51.0	38329,38357,38361,38327,38363,38353,38325,38333,38359,38331,38335,38060,38062,38058,38677,38355,38683,38323,38321,38717,38985	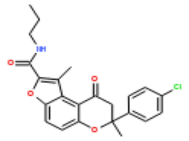			
38331	-9.3	-34.0	38359,38331,38335,38363,38353,38329,38357,38325,38361,38333,38327,38323,38321,38058,38062,38060,38355	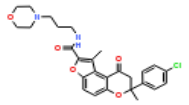	47		True
38333	-3.3	52.0	38333,38361,38329,38357,38327,38325,38335,38353,38363,38331,38359,38355,38058,38060,38062	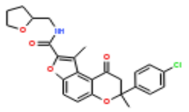			

38335	-2.6	61.0	38335,38359,38331,38325,38363,38353,38329,38357,38361,38333,38327,38323,38321,38058,38062,38060,38355	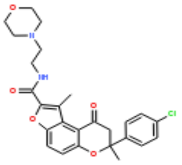	23		True
38353	-3.2	54.0	38363,38353,38359,38331,38329,38357,38325,38335,38361,38327,38333,38323,38062,38060,38321,38058,38355	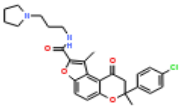			
38355	-12.6	-82.0	38355,38058,38321,38323,38060,38062,38327,38329,38357,38333,38361,38325,38335,38363,38353,38359,38331	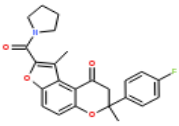			
38357	-7.0	-2.0	38329,38357,38361,38327,38363,38353,38325,38333,38359,38331,38335,38060,38062,38058,38677,38355,38683,38323,38321,38717,38985	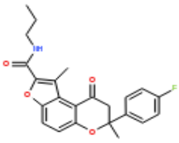			
38359	-4.5	34.0	38359,38331,38335,38363,38353,38329,38357,38325,38361,38333,38327,38323,38321,38058,38062,38060,38355	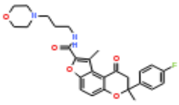			
38361	-6.1	11.0	38361,38333,38357,38329,38327,38363,38353,38359,38331,38325,38335,38058,38062,38060,38355,38323,38321	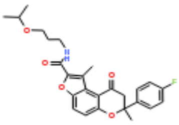			
38363	-1.1	83.0	38363,38353,38359,38331,38329,38357,38325,38335,38361,38327,38333,38323,38062,38060,38321,38058,38355	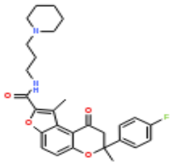			
38442	-0.9	86.0	38442,29582	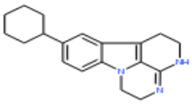			
38677	-4.0	65.0	38677,38683,38705,38717,38329,38357,38985	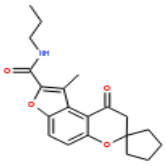			
38683	-8.8	28.0	38683,38677,38705,38717,38985,38329,38357	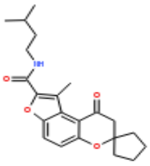		bad curve	True
38705	-1.8	83.0	38705,38677,38683,38717,38985	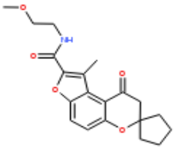			

38717	-4.9	58.0	38717,38985,38677,38683,38705,38327,38329,38357	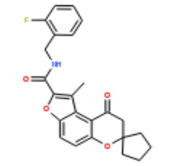			
38985	0.1	98.0	38985,38717,38683,38677,38705,38327,38329,38357	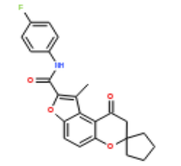			
39547	-5.5	49.0	39547,25541,25329,25343,25365,25371,25543,25369,25615,30331,25613,25309	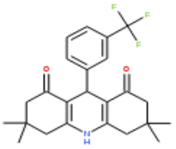			
39687	0.6	102.0	39687,36521	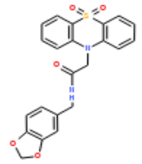			
40220	-0.7	92.0	40220,25365,25369,25371,25329,25541	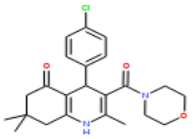			
100617	2.2	113.0	100617,101626,24550,24552	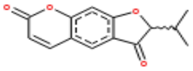			
100915	0.1	95.0	100915,100939,100969,100930,100952,100916,100926,100922,100921	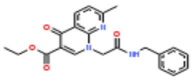			
100916	-0.3	92.0	100916,100922,100921,100926,100939,100930,100915,100952	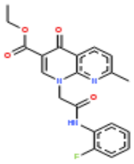			
100921	-0.4	91.0	100921,100922,100916,100926,100939,100930,100952,100915	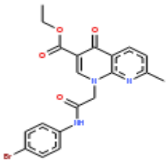			
100922	0.5	98.0	100922,100921,100916,100926,100939,100930,100915,100952	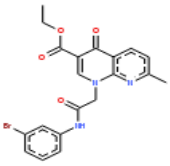			
100926	-2.8	73.0	100926,100916,100922,100921,100930,100915,100939	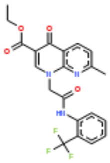			

100930	0.7	100.0	100930,100915,100921,100939,100916,100926,100922,100969,100952	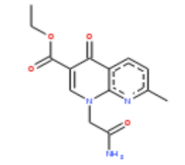			
100939	-12.1	3.0	100939,100915,100952,100916,100922,100969,100921,100930,100926	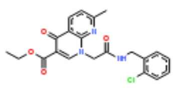			
100952	-1.0	87.0	100952,100969,100939,100915,100921,100922,100916,100930	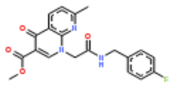			
100969	2.0	109.0	100969,100952,100915,100939,100930	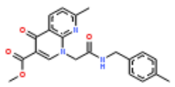			
101291	-9.5	5.0	101291,101343,32662,101346,101347	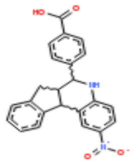			
101343	-0.3	98.0	101343,101291,32662,101346,101347,101344	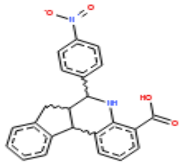			
101344	-12.5	-25.0	101344,32662,101343,101347	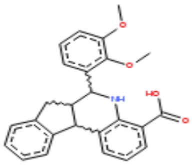			
101346	0.7	108.0	101346,101291,101347,101343,32662	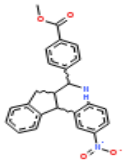			
101347	1.0	111.0	101347,101346,101291,101343,32662,101344	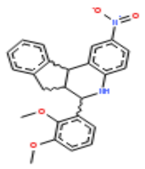			
101616	-13.2	-16.0	101616,24552,24422,24526,101618,24550,24528	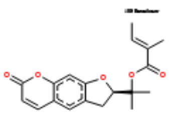			
101618	-53.8	-377.0	24526,101618,24552,101616,24550,24422	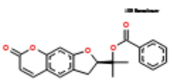			

101626	-0.8	94.0	100617,101626,24550,24552	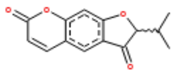			
101707	0.6	107.0	101707,29582	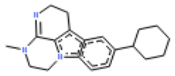			

1.2.3 Suppl. Tab. 3

Suppl. Tab. 3: Overview of all 55 Scaff10 derivatives. Structures labeld with [1] were commercially obtained. MW = molecular weight. logP = calculated partition coefficient (using Biovia Draw V.16.1), indicates a compounds's distribution in a biphasic system consisting of ocatanol-water. Disruption of the interaction of RhoA and AKAP-Lbc/DHPH (PPI) and IC_{50} values refer to HTRF assays (Figure 3 main manuscript), IC_{50} values ± SEM; all HTRF experiments were performed in duplicate; X-axis = compound concentration [µM], Y-axis = relative interaction of AKAP-Lbc/DHPH and RhoA (%). Solubility was assessed nephelometrically in DMEM + 10 % FCS + Scaff10 derivative with either 0.3 or 2 % DMSO final concentration; values in µM indicate maximal solubility of the compound under the respective condition. Yield in % indicates total yield of the last step of chemical synthesis for each of Scaff10 derivatives. n.d. = not determined. n.i. = not indicated.

				HTRF assay			Solubility			
**Scaff10 derivative**	**Structure**	**MW [g/mol]**	**log P**	**Disruption of the PPI**	**IC_{50} [µM]**	**Number of exp. in dupl.**	**2 % DMSO [µM]**	**0.3 % DMSO [µM]**	**Scaff10 class**	**Yield [%]**
AE168	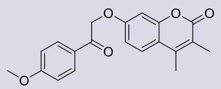	338.35	4.02	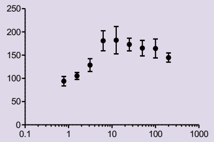	-	2	75	n.d.	6	62
Scaff10	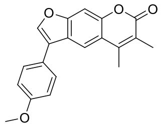	320.34	4.60	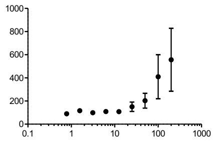	-	4	85	75	8	71
AE167	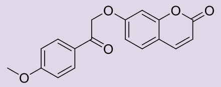	310.30	3.12	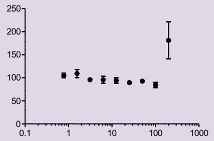	-	2	30	n.d.	6	35
Scaff10-1	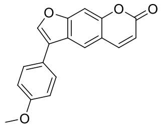	292.29	3.71	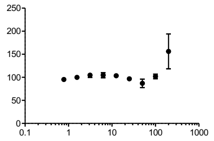	-	2	45	40	8	23
Scaff10-2	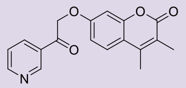	309.32	2.88	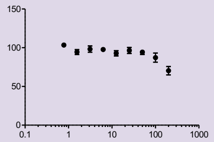	-	2	90	75	6	14

				HTRF assay			Solubility			
Scaff10 derivative	Structure	MW [g/mol]	log P	Disruption of the PPI	IC_{50} [µM]	Number of exp. in dupl.	2 % DMSO [µM]	0.3 % DMSO [µM]	Scaff10 class	Yield [%]
Scaff10-3	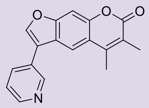	291.3	3.59	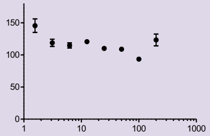	-	2	40	>150	8	n.i.
Scaff10-4	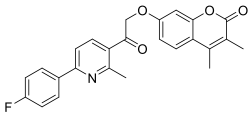	417.43	5.32	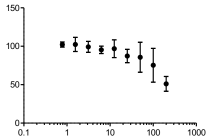	-	2	40	>150	6	n.i.
Scaff10-5	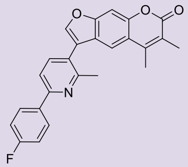	399.41	6.03	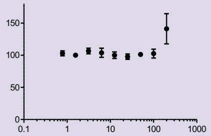	-	2	65	>150	8	71
Scaff10-6	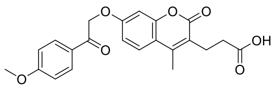	396.39	3.65	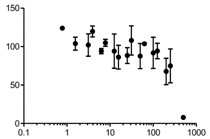	-	2	100	>150	7	n.i.
Scaff10-7	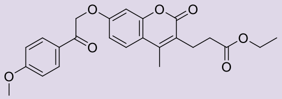	424.44	4.22	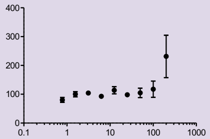	-	7	70	40	6	75

Scaff10 derivative	Structure	MW [g/mol]	log P	HTRF assay: Disruption of the PPI	HTRF assay: IC$_{50}$ [µM]	HTRF assay: Number of exp. in dupl.	Solubility: 2 % DMSO [µM]	Solubility: 0.3 % DMSO [µM]	Scaff10 class	Yield [%]
Scaff10-8	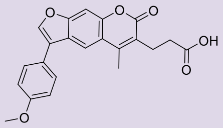	378.37	4.36	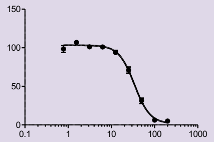	34.1±1.7	15	50	30	8	80
Scaff10-9	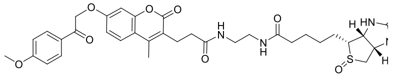	680.77	2.18	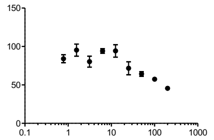	-	2	n.d.	n.d.	6	n.i.
Scaff10-10	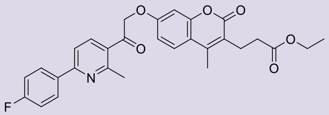	503.52	5.53	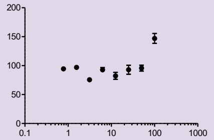	-	2	30	>150	6	66
Scaff10-11	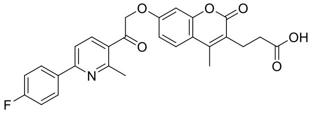	475.47	4.95	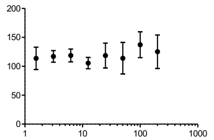	-	2	20	>150	7	n.i.
Scaff10-12	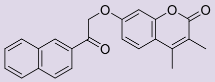	358.39	4.94	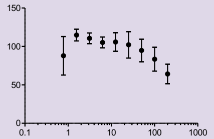	-	4	70	40	6	777

				HTRF assay			Solubility			
**Scaff10 derivative**	**Structure**	**MW [g/mol]**	**log P**	**Disruption of the PPI**	**IC_{50} [µM]**	**Number of exp. in dupl.**	**2 % DMSO [µM]**	**0.3 % DMSO [µM]**	**Scaff10 class**	**Yield [%]**
Scaff10-13	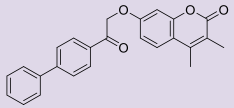	384.42	5.55	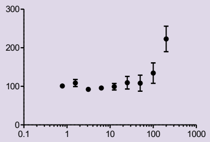	-	3	90	75	6	92
Scaff10-14	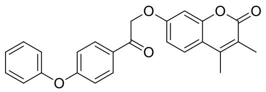	400.42	5.59	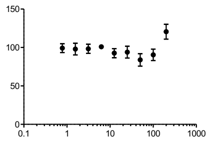	-	2	100	>150	6	97
Scaff10-15	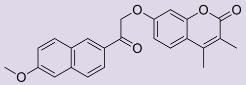	388.41	4.92	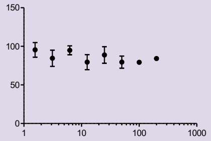	-	2	35	40	6	n.i.
Scaff10-17	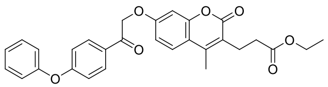	486.51	5.80	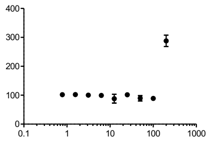	-	2	30	>30	6	n.i.
Scaff10-20	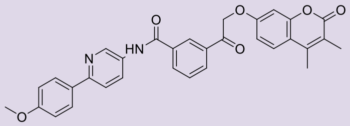	534.56	5.60	n.d.	n.d.	0	n.d.	n.d.	6	7

Scaff10 derivative	Structure	MW [g/mol]	log P	HTRF assay: Disruption of the PPI	HTRF assay: IC$_{50}$ [µM]	HTRF assay: Number of exp. in dupl.	Solubility: 2 % DMSO [µM]	Solubility: 0.3 % DMSO [µM]	Scaff10 class	Yield [%]
Scaff10-25	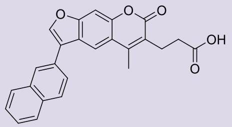	398.41	5.28	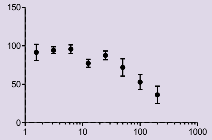	-	5	20	>30	8	22
Scaff10-26	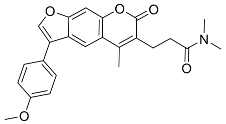	405.44	4.02	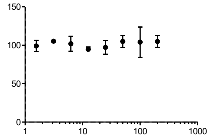	-	2	50	>150	8	75
Scaff10-27	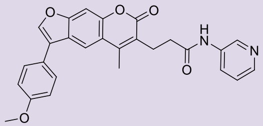	454.47	4.24	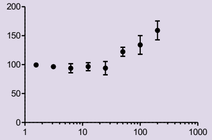	-	2	10	>30	8	79
Scaff10-28	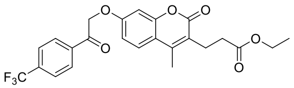	462.42	5.18	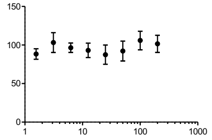	-	2	50	75	6	5
Scaff10-29	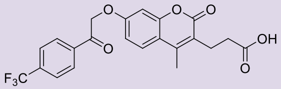	434.36	4.61	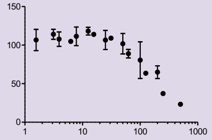	-	2	100	>30	7	18

				HTRF assay			Solubility			
**Scaff10 derivative**	**Structure**	**MW [g/mol]**	**log P**	**Disruption of the PPI**	**IC_{50} [µM]**	**Number of exp. in dupl.**	**2 % DMSO [µM]**	**0.3 % DMSO [µM]**	**Scaff10 class**	**Yield [%]**
Scaff10-30	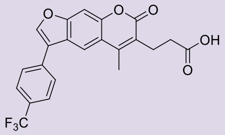	416.35	5.19.	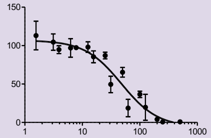	47.8±7.8	6	>100	n.d.	8	6
Scaff10-31	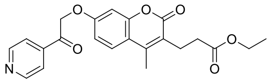	395.41	3.09	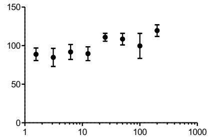	-	2	100	>150	6	n.i.
Scaff10-32	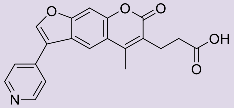	349.34	3.10	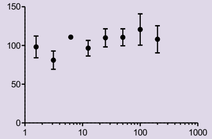	-	2	>100	>150	8	31
Scaff10-34	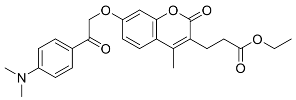	437.48	4.40	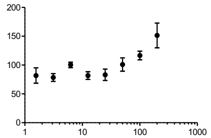	-	2	10	40	6	40
Scaff10-35	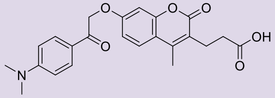	409.43	3.83	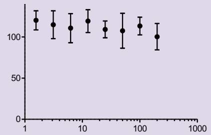	-	2	>100	>150	7	26

				HTRF assay			Solubility			
Scaff10 derivative	Structure	MW [g/mol]	log P	Disruption of the PPI	IC_{50} [µM]	Number of exp. in dupl.	2 % DMSO [µM]	0.3 % DMSO [µM]	Scaff10 class	Yield [%]
Scaff10-37	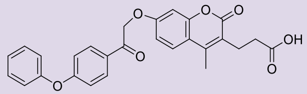	458.46	5.23	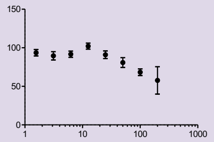	-	2	30	n.d.	7	7
Scaff10-38	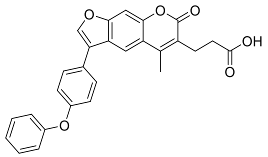	440.44	5.81	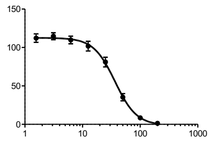	36.7±3.3	4	100	>150	8	9
Scaff10-39	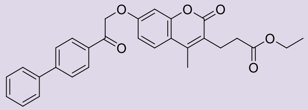	470.51	5.76	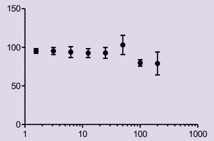	-	2	10	20	6	96
Scaff10-40	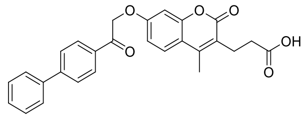	442.46	5.19	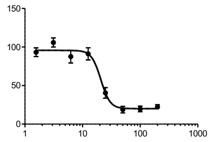	20.8±2.6	5	15	10	7	35
Scaff10-41	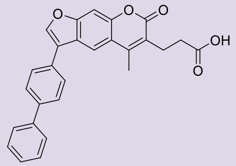	424.44	5.77	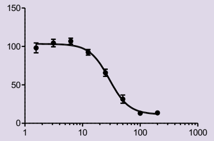	29.1±2.6	7	50	n.d.	8	34

				HTRF assay			Solubility			
Scaff10 derivative	Structure	MW [g/mol]	log P	Disruption of the PPI	IC_{50} [µM]	Number of exp. in dupl.	2 % DMSO [µM]	0.3 % DMSO [µM]	Scaff10 class	Yield [%]
Scaff10-42	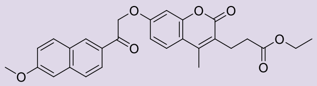	474.50	5.13	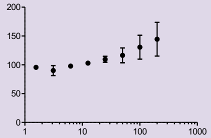	-	2	10	40	6	n.i.
Scaff10-43	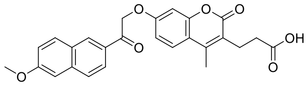	446.45	4.56	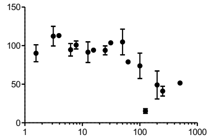	-	2	n.d.	n.d.	7	25
Scaff10-44	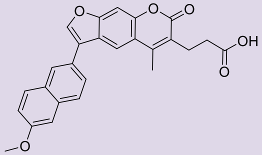	428.43	5.27	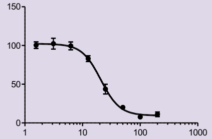	20.7±2.5	5	15	40	8	52
Scaff10-45	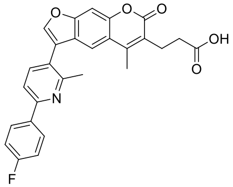	457.45	5.53	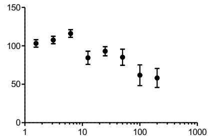	-	5	10	>150	8	24
Scaff10-46	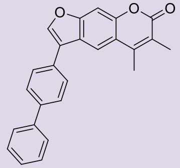	366.41	6.13	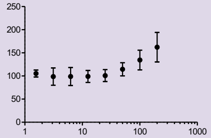	-	2	15	>150	8	25

				HTRF assay			Solubility			
**Scaff10 derivative**	**Structure**	**MW [g/mol]**	**log P**	**Disruption of the PPI**	**IC_{50} [µM]**	**Number of exp. in dupl.**	**2 % DMSO [µM]**	**0.3 % DMSO [µM]**	**Scaff10 class**	**Yield [%]**
Scaff10-47	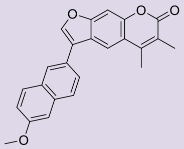	370.40	5.51	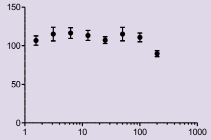	-	3	15	>150	8	n.i.
Scaff10-48	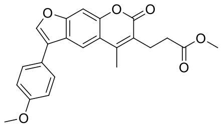	392.40	4.59	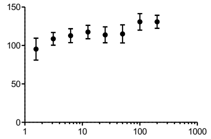	-	3	30	75	8	15
Scaff10-49[1]	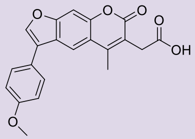	364.35	3.90	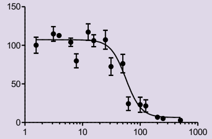	56.4±4.5	4	>100	>150	8	
Scaff10-50[1]	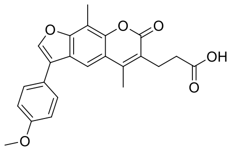	392.40	4.85	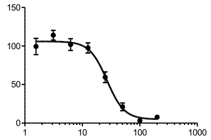	26.8±3.5	4	70	>150	8	
Scaff10-51	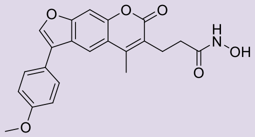	393.39	3.74	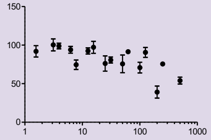	-	4	20	40	8	6

				HTRF assay			Solubility			
Scaff10 derivative	Structure	MW [g/mol]	log P	Disruption of the PPI	IC_{50} [µM]	Number of exp. in dupl.	2 % DMSO [µM]	0.3 % DMSO [µM]	Scaff10 class	Yield [%]
Scaff10-52	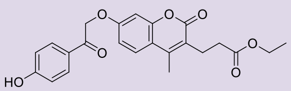	410.42	4.00	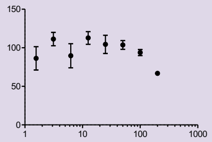	-	3	100	>150	6	n.i.
Scaff10-52 dimer[1]	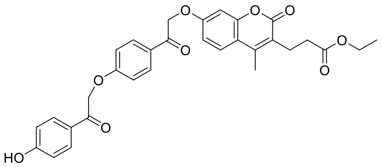	544.55	5.43	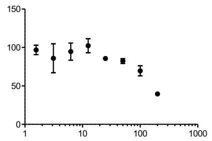	-	3	80	>150	6	n.i.
Scaff10-56	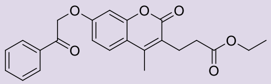	394.40	4.24	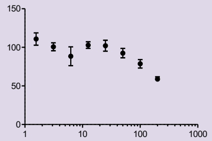	-	3	90	40	6	5
Scaff10-58	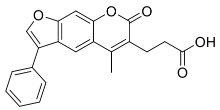	348.35	4.38	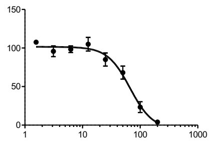	66.7±7.5	3	>100	>150	8	56
Scaff10-59	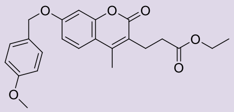	396.43	4.55	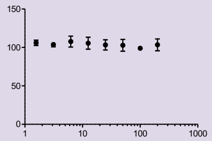	-	3	30	40	6	n.i.

Scaff10 derivative	Structure	MW [g/mol]	log P	HTRF assay: Disruption of the PPI	HTRF assay: IC$_{50}$ [µM]	HTRF assay: Number of exp. in dupl.	Solubility: 2 % DMSO [µM]	Solubility: 0.3 % DMSO [µM]	Scaff10 class	Yield [%]
Scaff10-60	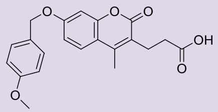	368.38	3.98	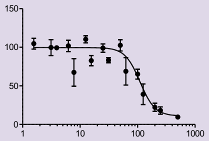	-	4	100	>150	7	n.i.
Scaff10-61	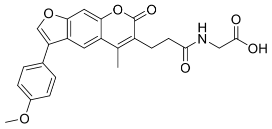	435.43	3.46	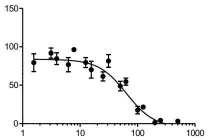	64.0±6.0	4	n.d.	>150	8	6
Scaff10-62	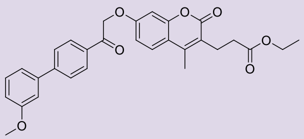	500.54	5.74	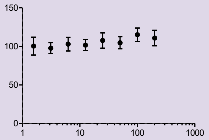	-	2	10	10	6	71
Scaff10-63	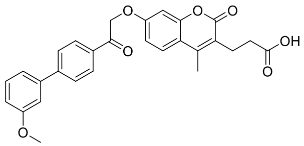	472.49	5.17	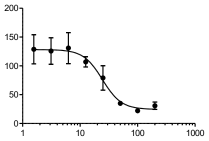	24.4±8.4	3	30	75	7	26

Scaff10 derivative	Structure	MW [g/mol]	log P	HTRF assay: Disruption of the PPI	HTRF assay: IC_{50} [µM]	HTRF assay: Number of exp. in dupl.	Solubility: 2 % DMSO [µM]	Solubility: 0.3 % DMSO [µM]	Scaff10 class	Yield [%]
Scaff10-64	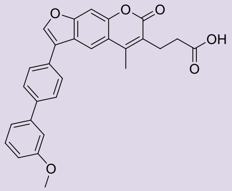	454.47	5.75	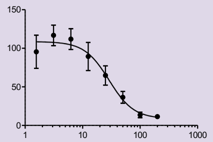	28.9±9.3	3	70	>150	8	21

2 Material and Methods

All chemicals used for preparing buffers and solutions were obtained from Sigma-Aldrich (Taufkirchen, DE) or Carl Roth GmbH & Co. KG (Karlsruhe, DE), unless indicated otherwise. All buffers were prepared with *A. dest.*

2.1 Cell lines

Human embryonic kidney cells (HEK293) were cultured in DMEM supplemented with 10 % fetal calf serum (FCS) and 1 % Penicillin/streptomycin (100 U/ml). Mouse collecting duct cells (MCD4) stably expressing human AQP2[5] were cultured in DMEM/F-12 supplemented with 5 % FCS and 5 µM dexamethasone. Rat embryonic myoblast cells (H9C2) were cultured in DMEM supplemented with 10 % FCS and 1 % Penicillin/streptomycin (100 U/ml). Human neuroblastoma cells (SH-SY5Y) were cultured in DMEM/F-12 supplemented with 10 % FCS and 1 % Penicillin/streptomycin (100 U/ml). Human breast adenocarcinoma cells (MCF-7) were cultured in DMEM supplemented with 10 % FCS and 1 % Penicillin/streptomycin (100 U/ml). Human embryonic kidney cells (HEK293-EBNA) stably expressing the Epstein Barr virus (EBV) EBNA-1 gene from pCMV/EBNA controlled by the CMV promotor[6, 7] is a suspension cell line and was cultured in Freestyle F17 supplemented with 0.5 % peptone and 1 % Penicillin/streptomycin (100 U/ml) and transfected using PEI. Inner medullary collecting duct (IMCD) cells from rats were prepared as described.[8, 9] In brief, 10 to 12 weeks old rats (Wistar Han, Charles River Laboratories International, Inc., Sulzfeld, DE) were anesthetized and decapitated. Inner medullae were dissected and digested enzymatically with hyaluronidase and collagenase. Cells were resuspended in fully supplemented medium (DMEM supplemented with 1% non-essential aminoacids, 1 % ultroser G, 500 µM DBcAMP, 20 U/ml nystatin, 0.25 µg/ml gentamicin, 1.5 g/l glucose, 100 mM NaCl and 100 mM urea) and seeded in collagen type IV (BD Biosciences, Heidelberg, DE; #356233)-coated dishes. Six (biotinylation) to 13 (Rhotekin assay) days after seeding, cells were used for experiments. 24 h before starting the experiment, cells were incubated in medium without DBcAMP and nystatin in order to increase the perinuclear localization of AQP2.

2.2 Plasmids

The AKAP-Lbc/DHPH sequence fused to an N-terminal GFP-tag and to C-terminal His_6-tag was subcloned into the pTT5 plasmid (ampicillin resistance) containing a secretion sequence derived from an immunoglobulin (human IgG_1).[10] The full-length sequence refers to a secretion sequence derived from an immunoglobulin (bp 1-56 of accession number

KT365995 of the NCBI database) – GFP (coding for bp 7756-8472 of pEGFP-N1-Pdx1, KU341334.1 of the NCBI database) – AKAP-Lbc/DHPH (bp 5914-7026 of accession number CCDS 32320.1 of the NCBI database) – His_6. 8 mutations (Supplemental Figure 2) were included into the AKAP-Lbc/DHPH sequence to remove bach-to-back rare codons in a row by mutagenesis using the KOD DNA polymerase and the following primers. Each primer pair inserted two mutations.

M1 Fw 5'- GTCGGATCATCGACAGCAAGTTTC-3‘
M1 Rv 5‘-GCTGTCGATGATCCGACTCCAAGACTCTGC-3‘
M2 Fw 5‘-GGCAAGAAGTGATCTATGAGTTGATGCAG-3‘
M2 Rv 5‘-CTCATAGATCACTTCTTGCCGTTTGACC-3‘
M3 Fw 5‘-GAATGCAGAACGGCTGAAGAAGACATATGGC-3‘
M3 Rv 5‘-CTTCTTCAGCCGTTCTGCATTCTCACC-3‘
M4 Fw 5‘-GAAGCTTGTGCGGGATGGGAGTGTGTTTC-3‘
M4 Rv 5‘-CTCCCATCCCGCACAAGCTTCTTCCGTTTCAAATC-3‘.

2.3 Luciferase reporter assays

Luciferase reporter assays were carried out according to the manufacture's protocol "*Dual-luciferase reporter assay*" (Promega). In brief, HEK293-Zellen were transiently transfected to express two luciferases and the indicated GEFs (Supplemental Figure 1). The serum response element (SRE-) promotor controls the firefly luciferase. Active RhoA stimulates *serum response factor* (SRF), which in turn binds to the SRE and stimulates expression of *firefly* luciferase. 20 hours post transfection the cells were exposed to Scaf10 and another 4 hours later they were lysed in Passive Lysis Buffer (Promega). Luciferase activity, i.e. luminescence signals, was measured in 96- well plates (*OptiPlate 96*, PerkinElmer) and the *LarII*- or *Stop-&Glow* substrate solutions (PerkinElmer) in a microtiter plate reader.

2.4 Chemical synthesis

2.4.1 ^{1}H NMR

^{1}H NMR spectra were recorded on a Bruker AV 300 (1H: 300 MHz). Chemical shifts are depicted in parts per million (ppm). As internal standard, the measured NMR-spectra were normalised to the solvent peak used for recording the spectrum (dimethylsulfoxide-d_6 (DMSO): 1H: 2.50 ppm).

2.4.2 LC/MS

All mass spectra were recorded on a QTrap 4000 (Applied Biosystems) connected to a Shimadzu UFLC system. Electrospray ionization (ESI) was used to carry out ionization of an

approximately 1 µM solution of the sample in pure ACN or ACN/H_2O (1:1). Values are depicted as atomic mass units m/z. The system is equipped with a Shimadzu LC-20 system (degaser Degasys DG-2410, Autosampler SIL-20A, Controller CBM-20A) with a DAD-UV-detector (SPD-M20A). LC/MS runs were performed on an analytical Nucleodur column (100-5 C18 ec, 100 Å, 5 µm, 50 x 4 mm, Macherey-Nagel). The flow rate was set to 1 ml/min and column temperature to 40 °C. Injection volumes were set between 10 µl and 20 µl with an approximate sample concentration of 50 µM. Analytical LC/MS runs were performed with the following gradient: water/0.1 % formic acid (v/v) (solvent A) and ACN/0.1 % formic acid (v/v) (solvent B), 5 % B to 95 % B. Overall run time was set to 5 (cellular uptake) or 11 minutes (min) (chemical synthesis), detection wavelength: 254 nm for chemical synthesis and 308 nm for cellular uptake (absorption maximum of Scaff10 derivatives). Purity was determined based on the chromatograms of an LC/MS run. All compounds used in *in vitro* or *in vivo* tests had a purity of >95%. Absorption maxima were extracted from spectra generated by the DAD detector during LC/MS analyses.

2.4.3 HPLC

The preparative HPLC system consisted of a Shimadzu LC-20 system (degasser Degasys DG-2410, Autosampler SIL-20A HT, Controller CBM-20A) with a DAD-UV-detector (SPD-M20A). Preparative separations were performed on a Nucleodur C-18 column (100 Å, 10 x 250 mm x 21 mm, Macherey-Nagel) with the flow rate set to 8.0 ml/min. Semi-preparative separations were performed on a Nucleodur C-18 column (100 Å, 10 µm 250 mm x 10 mm, Macherey-Nagel) with the flow rate set to 2.0 ml/min. Water/0.1 % acetic acid (v/v) (solvent A); ACN/0.1 % acetic acid (v/v) (solvent B).

3 References

1. Tamma G, Wiesner B, Furkert J, Hahm D, Oksche A, Schaefer M, et al. The prostaglandin E2 analogue sulprostone antagonizes vasopressin-induced antidiuresis through activation of Rho. J Cell Sci. 2003;116(Pt 16):3285-94. Epub 2003/06/28. doi: 10.1242/jcs.00640jcs.00640 [pii]. PubMed PMID: 12829746.

2. Lutz S, Freichel-Blomquist A, Yang Y, Rumenapp U, Jakobs KH, Schmidt M, et al. The guanine nucleotide exchange factor p63RhoGEF, a specific link between Gq/11-coupled receptor signaling and RhoA. J Biol Chem. 2005;280(12):11134-9. doi: 10.1074/jbc.M411322200. PubMed PMID: 15632174.
3. Mohl M, Winkler S, Wieland T, Lutz S. Gef10--the third member of a Rho-specific guanine nucleotide exchange factor subfamily with unusual protein architecture. Naunyn Schmiedebergs Arch Pharmacol. 2006;373(5):333-41. doi: 10.1007/s00210-006-0083-0. PubMed PMID: 16896804.
4. Pfreimer M, Vatter P, Langer T, Wieland T, Gierschik P, Moepps B. LARG links histamine-H1-receptor-activated Gq to Rho-GTPase-dependent signaling pathways. Cell Signal. 2012;24(3):652-63. doi: 10.1016/j.cellsig.2011.10.014. PubMed PMID: 22100544.
5. Bogum J, Faust D, Zuhlke K, Eichhorst J, Moutty MC, Furkert J, et al. Small-molecule screening identifies modulators of aquaporin-2 trafficking. J Am Soc Nephrol. 2013;24(5):744-58. doi: 10.1681/ASN.2012030295. PubMed PMID: 23559583; PubMed Central PMCID: PMC3636789.
6. DuBridge RB, Tang P, Hsia HC, Leong PM, Miller JH, Calos MP. Analysis of mutation in human cells by using an Epstein-Barr virus shuttle system. Mol Cell Biol. 1987;7(1):379-87. PubMed PMID: 3031469; PubMed Central PMCID: PMC365079.
7. Durocher Y, Perret S, Kamen A. High-level and high-throughput recombinant protein production by transient transfection of suspension-growing human 293-EBNA1 cells. Nucleic acids research. 2002;30(2):E9. PubMed PMID: 11788735; PubMed Central PMCID: PMC99848.
8. Maric K, Oksche A, Rosenthal W. Aquaporin-2 expression in primary cultured rat inner medullary collecting duct cells. Am J Physiol. 1998;275(5 Pt 2):F796-801. Epub 1998/11/14. PubMed PMID: 9815137.
9. Faust D, Geelhaar A, Eisermann B, Eichhorst J, Wiesner B, Rosenthal W, et al. Culturing primary rat inner medullary collecting duct cells. Journal of visualized experiments : JoVE. 2013;(76). doi: 10.3791/50366. PubMed PMID: 23852264.
10. Zhang J, Liu X, Bell A, To R, Baral TN, Azizi A, et al. Transient expression and purification of chimeric heavy chain antibodies. Protein expression and purification. 2009;65(1):77-82. doi: 10.1016/j.pep.2008.10.011. PubMed PMID: 19007889.

4 Medicinal Chemistry

4.1 Chemical synthesis

4.1.1 Iodo-1-[4-(3-methoxyphenyl)phenyl]ethanone (**2a**)

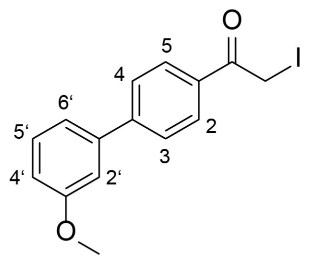

1-(3'-Methoxybiphenyl-4-yl)ethanone (**1**) (400 mg, 1.77 mmol) and iodine (1.8 g, 7.07 mmol) were solved in 10 ml methanol and refluxed for 2 h. 150 ml water and 75 ml ethyl acetate were added as well as 10 % Na_2SO_3-solution until iodine was completely reduced. The aqueous layer was extracted thrice with 75 ml ethyl acetate. The organic layers were collected and the product was dried under reduced pressure. Final product gave an orange-yellowish solid in 80 % yield (526 mg).

^{1}H NMR (300 MHz, DMSO-d_6): δ(ppm) = 2.56 –2.67 (s, 2H, -CO-CH_2-I), 3.75 –3.90 (m, 3H, -OCH_3), 6.96 –7.05 (d, J = 7.32 Hz, 1H, H-4’), 7.24 –7.35 (m, 2H, H-2’,-6’), 7.38 – 7.47 (t, J = 7.32 Hz, 1H, H-5’), 7.80 – 7.86 (d, J = 8.54 Hz, 2H, H-3, -4), 7.99 – 8.06 (d, J = 8.55 Hz, 2H, H-2, -5)

ESI-MS: calculated (cal.) 353.00, experimental (exp.) 352.9 ($[M+H]^+$)

HPLC: 84 % purity at 254 nm

t_R (HPLC): 7.32 min; λ_{max} = 253 nm and 303 nm

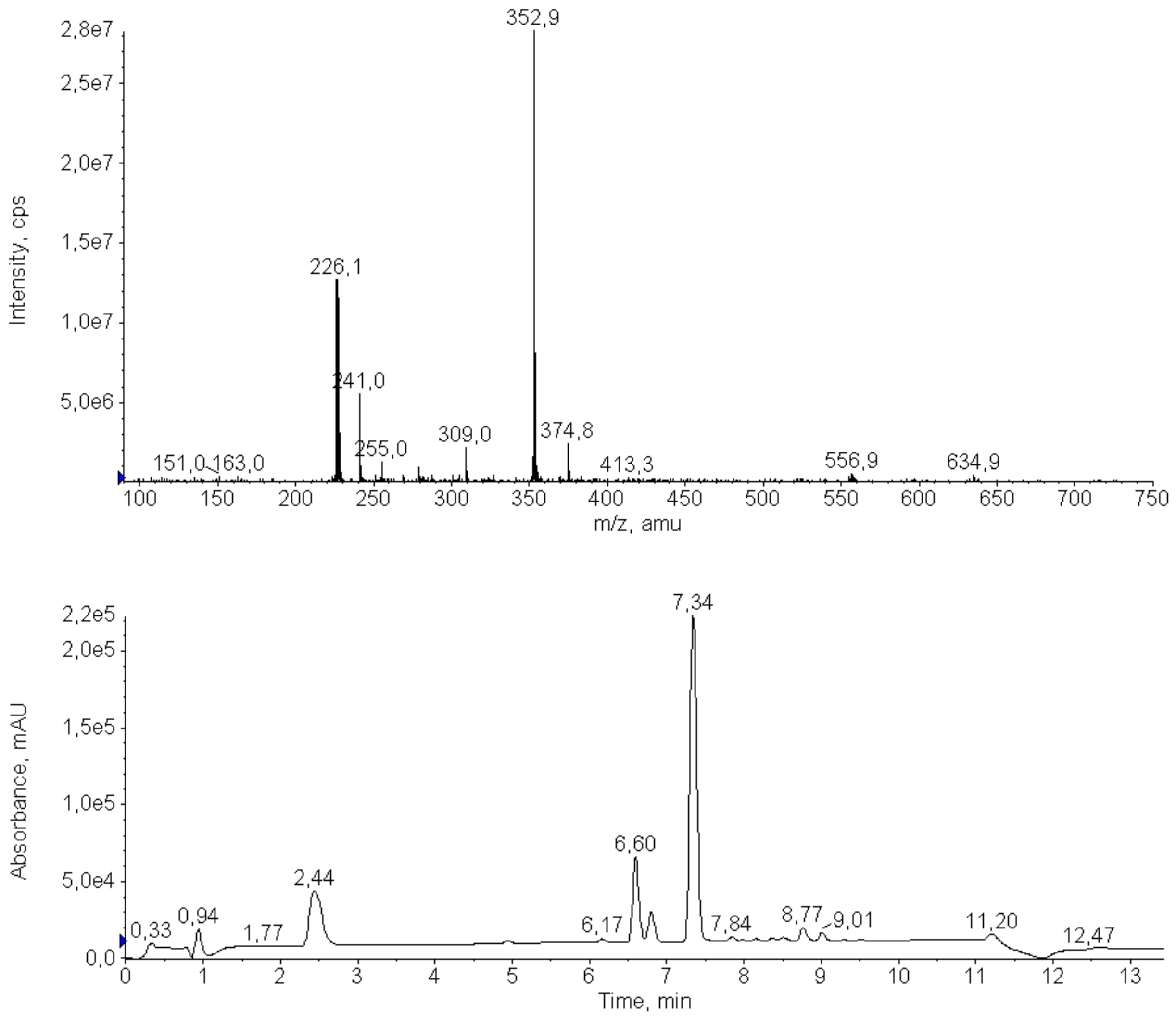

Supporting Information Figure 1: LC/MS analysis of **2a**. Upper panel: Mass spectrum. Lower panel: according chromatogram.

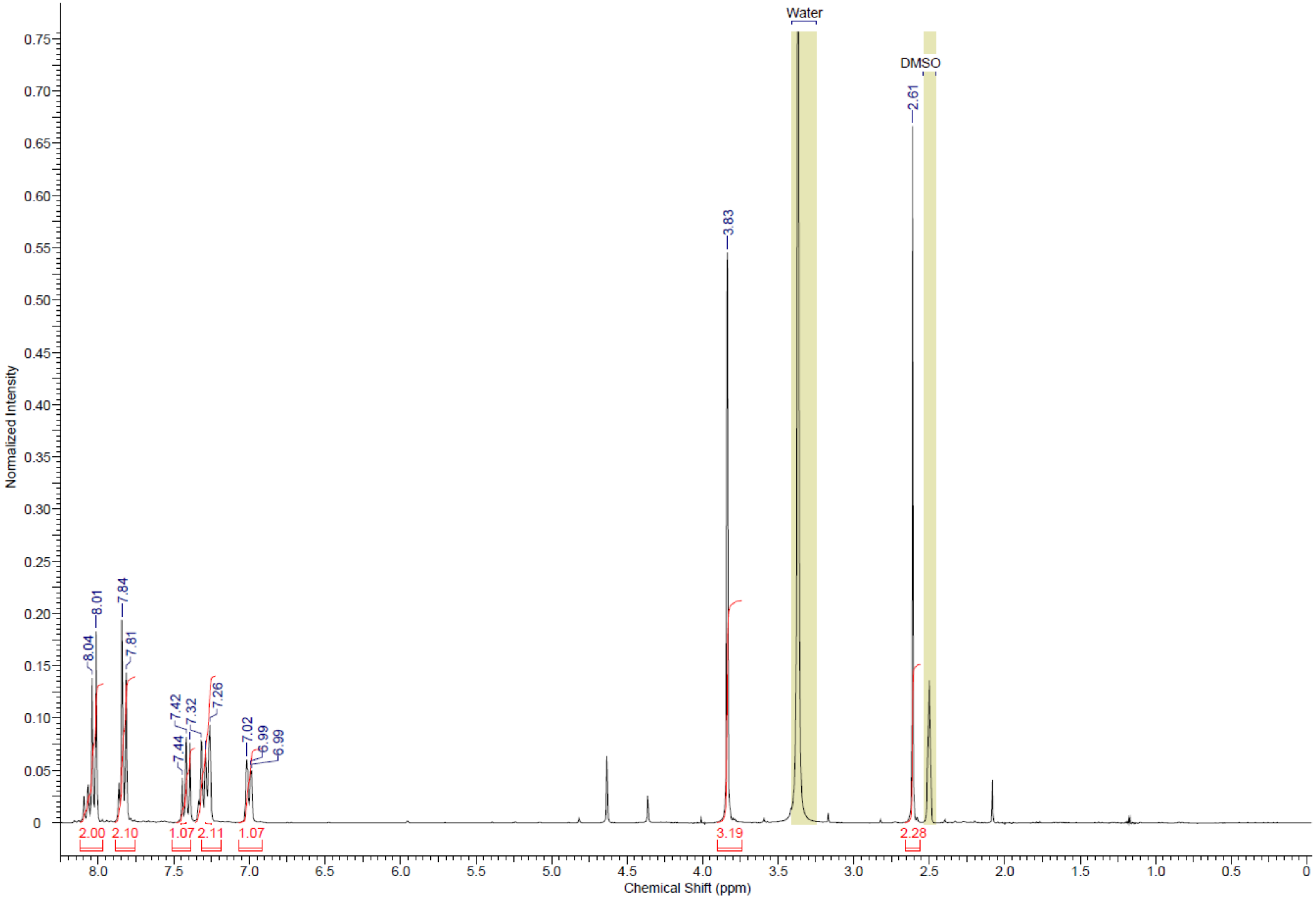

Supporting Information Figure 2: ^{1}H NMR spectrum of **2a**, 300 MHz, DMSO-d$_6$.

4.1.2 Ethyl 3-(7-hydroxy-4-methyl-2-oxo-chromen-3-yl)propanoate (**5a**)

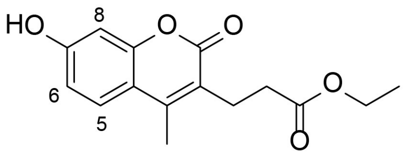

Resorcinol (**3**) (4 g, 36.3 mmol) and diethyl2-acetylglutarat (**4**) (8 ml, 37.2 mmol, 1.07 g/ml) were solved in 7 ml HCl (1.25 M) in ethanol under nitrogen atmosphere. The reaction mixture was cooled (0 °C) for 3 h and reacted overnight at room temperature. The solution was poured into 200 ml of ice water and the product was extracted thrice with 50 ml methylene chloride. The organic layers were collected and recrystallised in ethyl acetate. Yellowish crystals formed overnight at 8 °C. Final yield was 79 % (7.9 g).

^{1}H NMR (300 MHz, DMSO-d_6): δ(ppm) = 1.08 –1.25 (t, J = 7.32 Hz, 3H, -CH_2-C**H_3**), 2.35 – 2.42 (s, 3H, Ar-CH_3), 2.43 – 2.50 (t, J = 7.32 Hz, 2H, -CH_2-C**H_2**-COOEt), 2.74 - 2.87 (t, J = 7.32 Hz, 2H, -C**H_2**-CH_2-COOEt), 4.00 – 4.12 (q, J = 7.32 Hz, 2H, -C**H_2**-CH_3), 6.65 – 6.74 (s, 1H, H-8), 6.77 – 6.85 (dd, J = 2.44 Hz, 8.54 Hz, 1H, H-6), 7.59 – 7.69 (d, J = 2.44 Hz, 1H, H-5), 10.35 – 10.59 (s, 1H, OH)

ESI-MS: cal. 277.10, exp. 277.1 ($[M+H]^+$)

HPLC: 99 % purity at 254 nm

t_R (HPLC): 4.94 min; λ_{max} = 323 nm

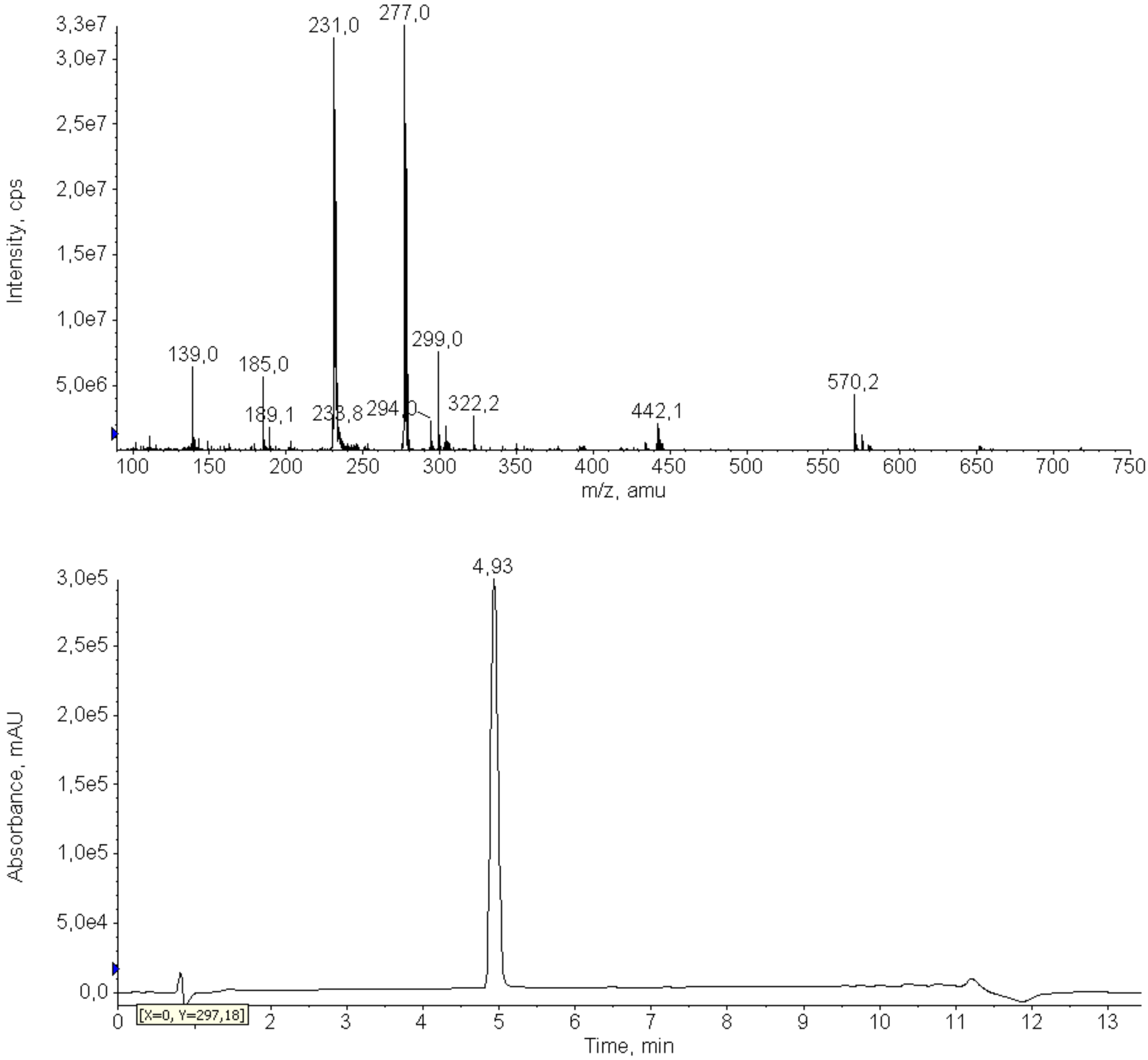

Supporting Information Figure 3: LC/MS analysis of **5a**. Upper panel: Mass spectrum. Lower panel: according chromatogram.

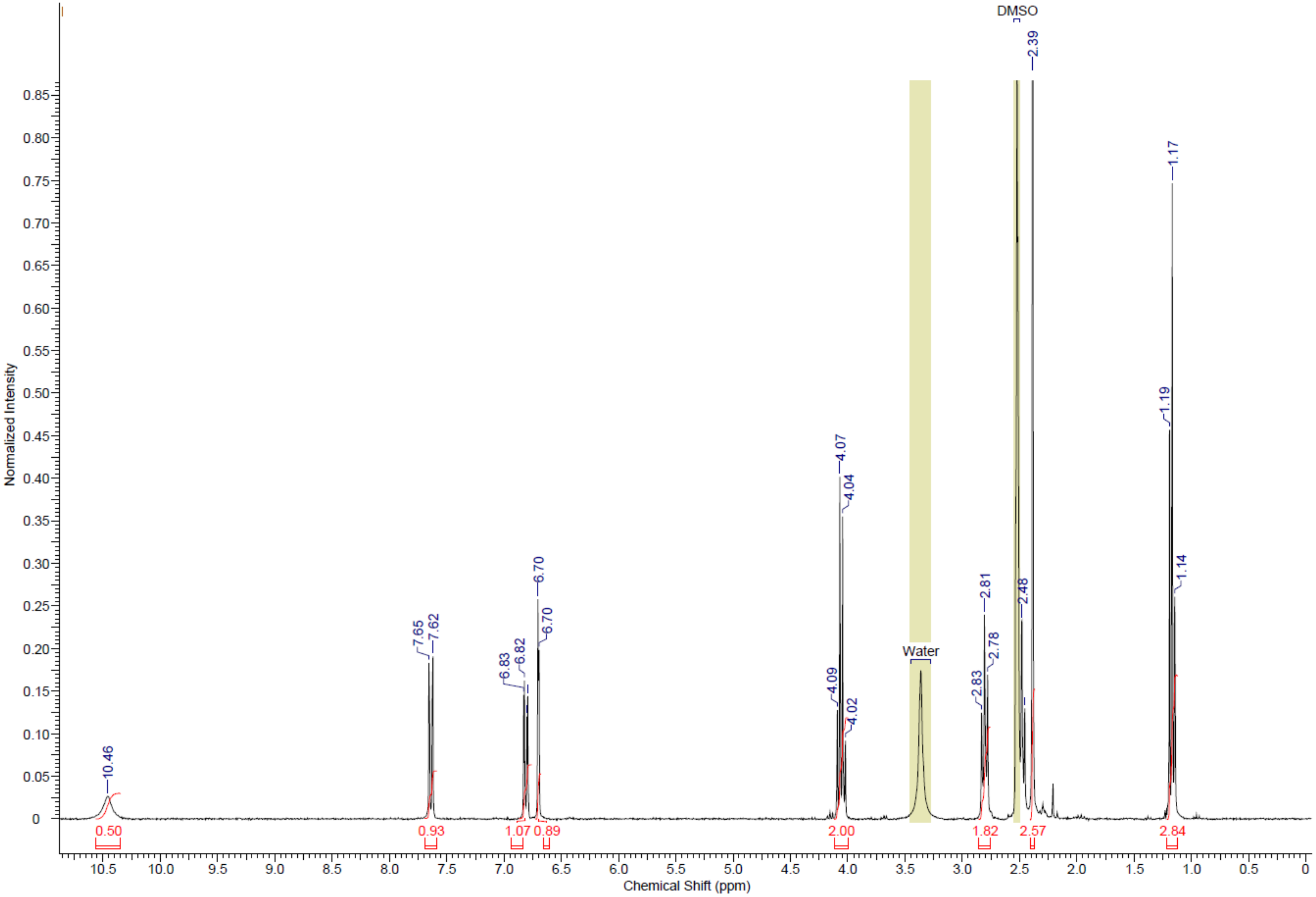

Supporting Information Figure 4: ^{1}H NMR spectrum of **5a**, 300 MHz, DMSO-d_6.

4.1.3 [2-(4-Methoxyphenyl)-2-oxo-ethoxy]-3,4-dimethyl-chromen-2-one (**AE168**)

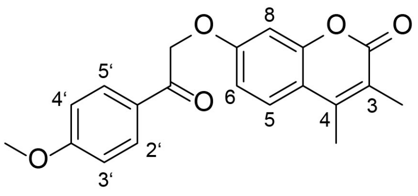

7-Hydroxy-3,4-dimethyl-chromen-2-one (**5b**) (200 mg, 1.05 mmol) was solved in acetone and preactivated with potash in excess (363 mg, 2.63 mmol) by 55 °C. The α-haloketone 2-bromo-1-(4-methoxyphenyl)ethanone (**2b**) was added (263 mg, 1.15 mmol). The mixture was refluxed at the same temperature. The final product after purification gave a white solid in 62 % yield (220 mg).

^{1}H NMR (300 MHz, DMSO-d_6): δ(ppm) = 1.20 – 1.28 (s, 3H, -C3-CH_3), 1.50 – 1.58 (s, 3H, -C4-CH_3), 2.98 – 3.08 (s, 3H, -OCH_3), 4.78 – 4.84 (s, 2H, -CO-C**H_2**-OAr), 6.12 – 6.19 (m, 2H, H-6, -8), 6.23 – 6.30 (d, J = 9.16 Hz, 2H, H-3’, -4’), 6.83 – 6.89 (d, J = 8.55 Hz, 1H, H-5), 7.15 – 7.22 (d, J = 9.16 Hz, 2H, H-2’, -5’)

ESI-MS: cal. 339.12, exp. 339.0 ($[M+H]^+$)

HPLC: 98 % purity at 254 nm

t_R (HPLC): 5.43 min; λ_{max} = 272 nm and 314 nm

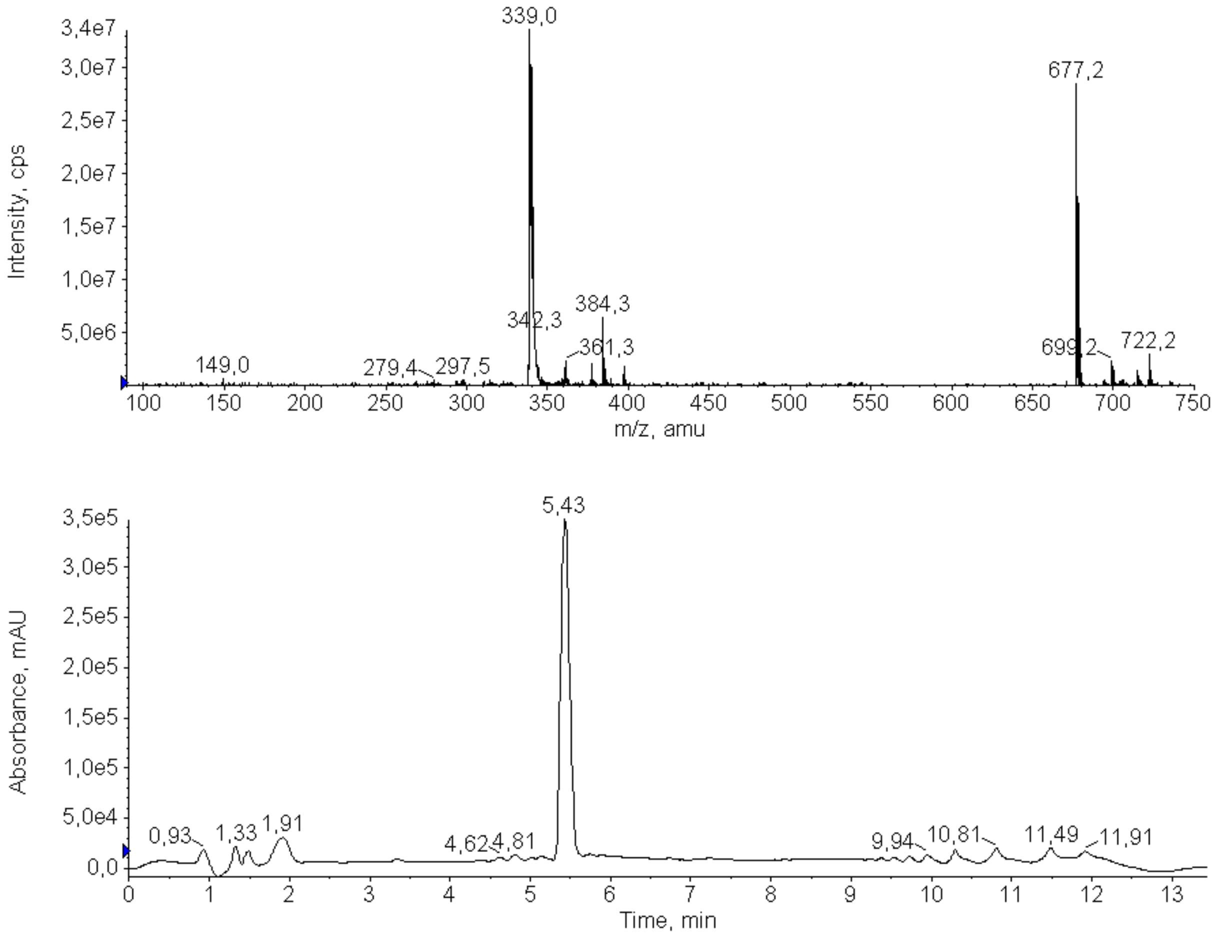

Supporting Information Figure 5: LC/MS analysis of **AE168**. Upper panel: Mass spectrum. Lower panel: according chromatogram.

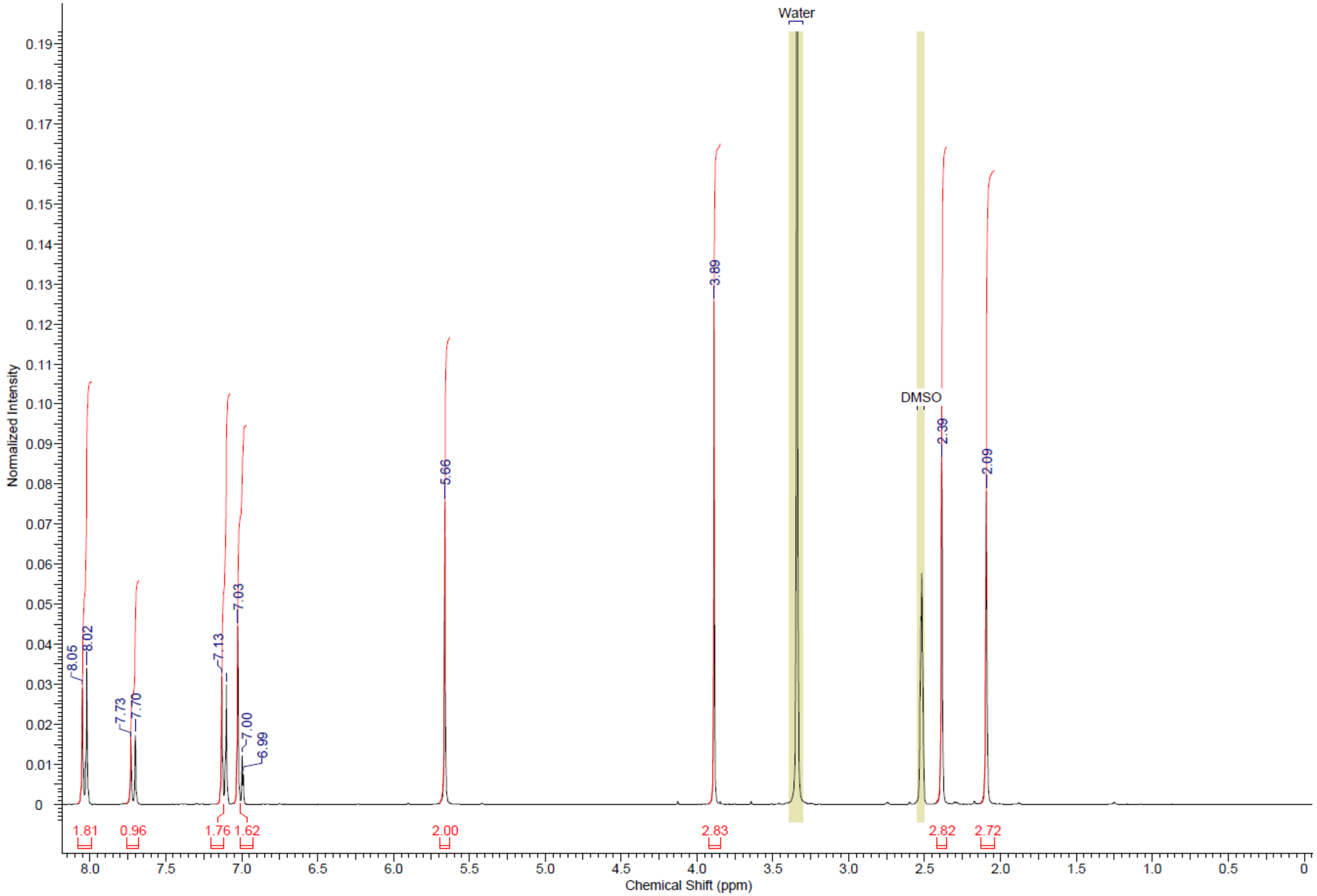

Supporting Information Figure 6: ^{1}H NMR spectrum of **AE168**, 300 MHz, DMSO-d_6.

4.1.4 3-(4-Methoxyphenyl)-5,6-dimethyl-furo[3,2-*g*]chromen-7-one (**Scaff10**)

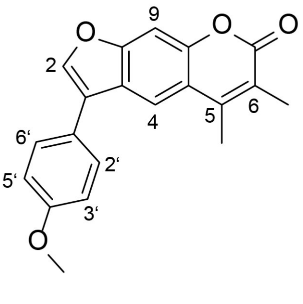

7-[2-(4-Methoxyphenyl)-2-oxo-ethoxy]-3,4-dimethyl-chromen-2-one (**AE168**) (250 mg, 0.74 mmol) was saponificated in 10 ml 1 M sodium hydroxide at 100 °C for 16 h. Dropwise addition of 1 M H_2SO_4 resulted in precipitation of the product. The precipitated solid was washed with water and recrystallised in ethanol. The final product gave a white solid in 71 % yield (168 mg).

^{1}H NMR (300 MHz, DMSO-d_6): δ(ppm) = 2.12 - 2.20 (s, 3H, -C6-CH_3), 2.48 – 2.53 (s, 3H, -C5-CH_3), 3.81 – 3.89 (s, 3H, -OCH_3), 7.09 – 7.16 (d, J = 9.16 Hz, 2H, H-3', -5'), 7.73 – 7.78 (m, 3H, H-9, -2', -6'), 8.11 – 8.14 (s, 1H, H-4), 8.36 – 8.38 (s, 1H, H-2)

ESI-MS: cal. 321.11, exp. 321.1 ($[M+H]^+$)

HPLC: 99 % purity at 254 nm

t_R (HPLC): 7.62 min; λ_{max} = 251 nm and 305 nm

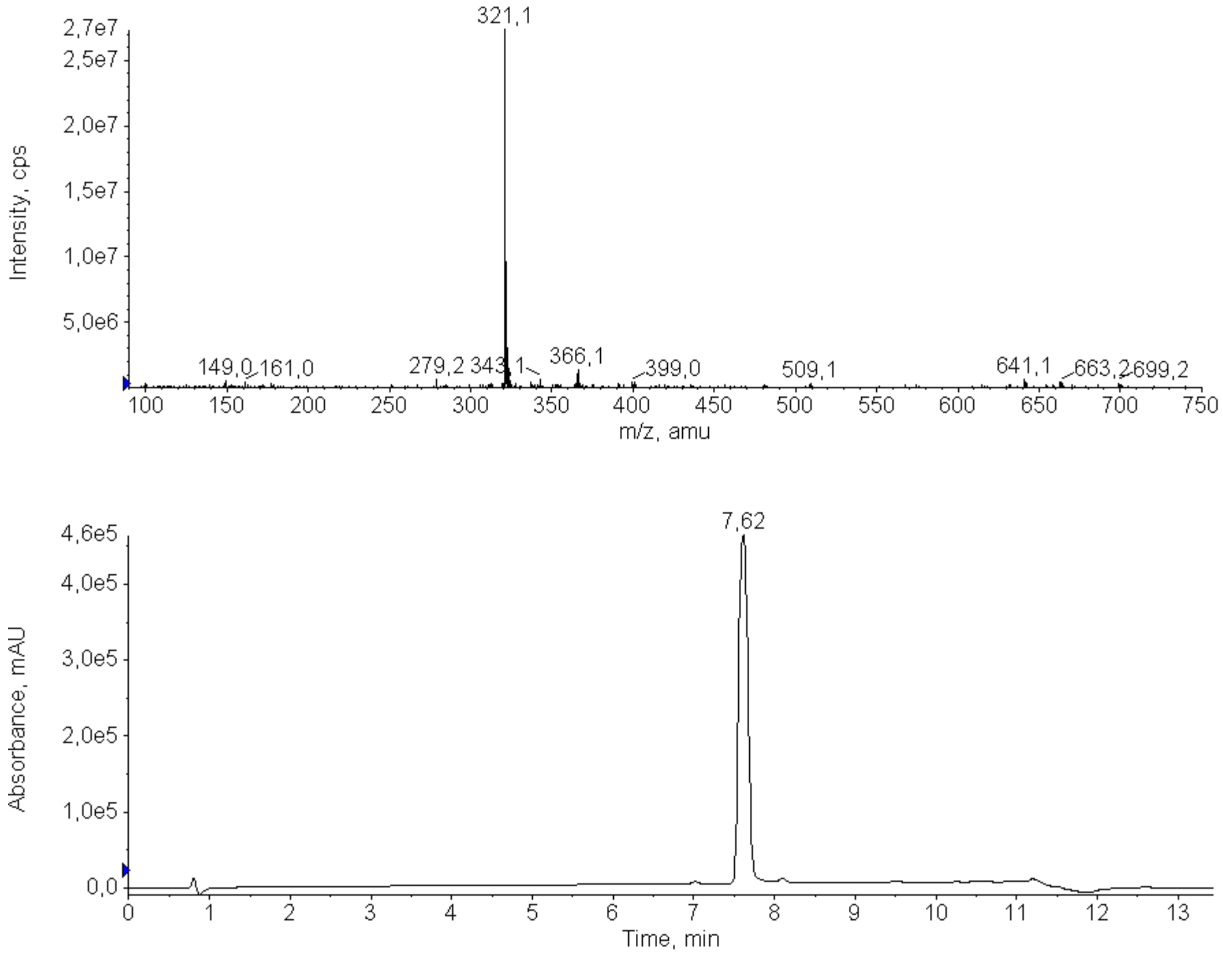

Supporting Information Figure 7: LC/MS analysis of **Scaff10**. Upper panel: Mass spectrum. Lower panel: according chromatogram.

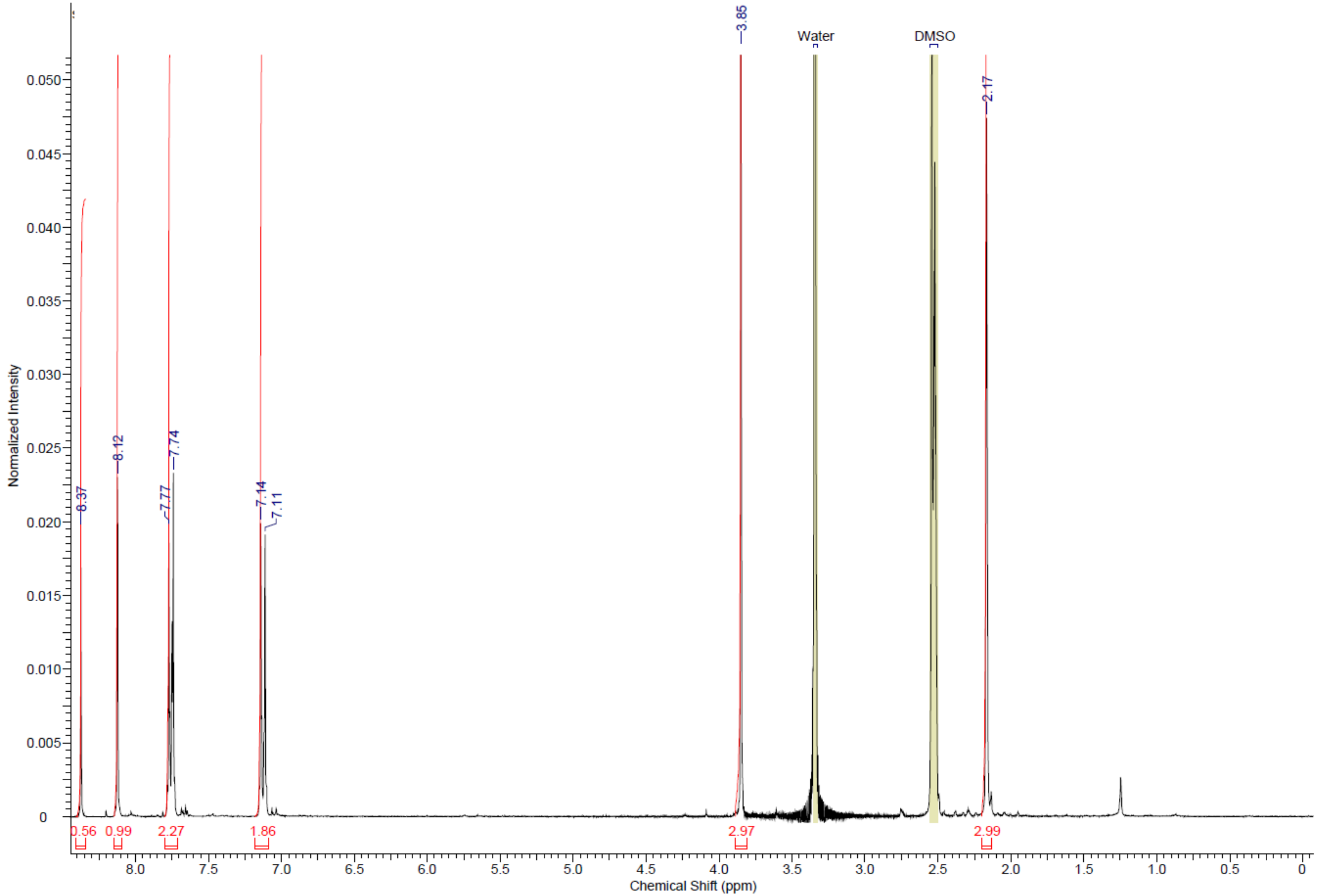

Supporting Information Figure 8: ^{1}H NMR spectrum of **Scaff10**, 300 MHz, DMSO-d_6.

4.1.5 7-[2-(4-Methoxyphenyl)-2-oxo-ethoxy]chromen-2-one (**AE167**)

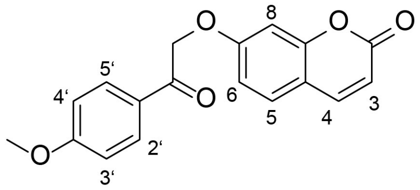

7-Hydroxychromen-2-one (**5c**) (1 g, 6.17 mmol) was solved in acetone and preactivated with potash in excess (2.142 g, 15.50 mmol) at 55 °C. The α-haloketone 2-bromo-1-(4-methoxyphenyl)ethanone (**2b**) was added (1.554 g, 6.84 mmol). The mixture was refluxed at the same temperature for 4 h. Dropwise addition of 1 M H_2SO_4 resulted in precipitation of the product. The precipitated solid was recrystallised in ethanol. The final product gave a white solid in 35 % yield (670 mg).

^{1}H NMR (300 MHz, DMSO-d_6): δ(ppm) = 3.86 – 3.91 (s, 3H, -OCH_3), 5.67 – 5.72 (s, 2H, -CO-C**H_2**-OAr), 6.28 – 6.35 (d, J = 9.77 Hz, 1H, H-3), 6.99 – 7.16 (m, 4H, H-6, -8, -3', -4'), 6.63 – 6.69 (d, J = 8.54 Hz, 1H, H-5), 7.99 – 8.07 (m, 3H, H-4, 2', -5')

ESI-MS: cal. 311.09, exp. 310.9 ($[M+H]^+$)

HPLC: 99 % purity at 254 nm

t_R (HPLC): 5.60 min; λ_{max} = 266 nm and 305 nm and 331 nm

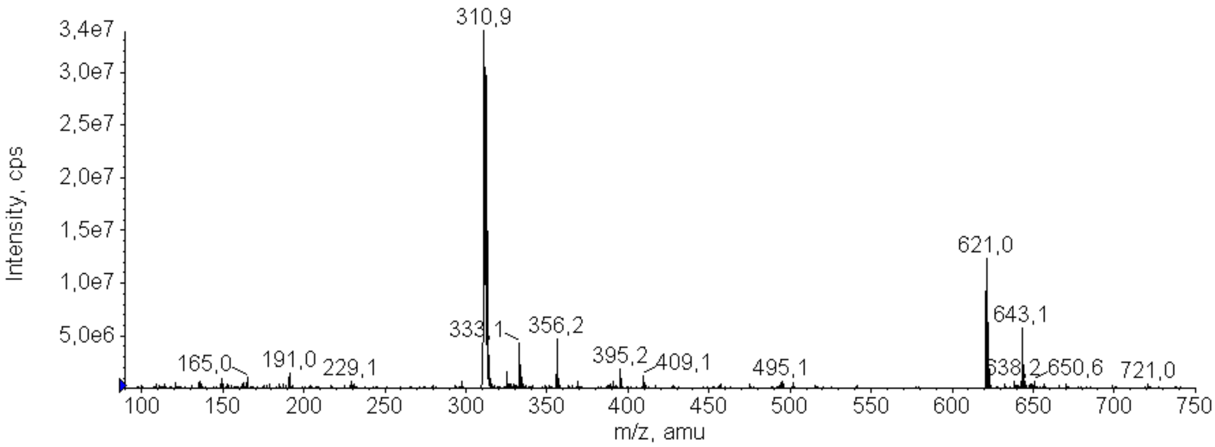

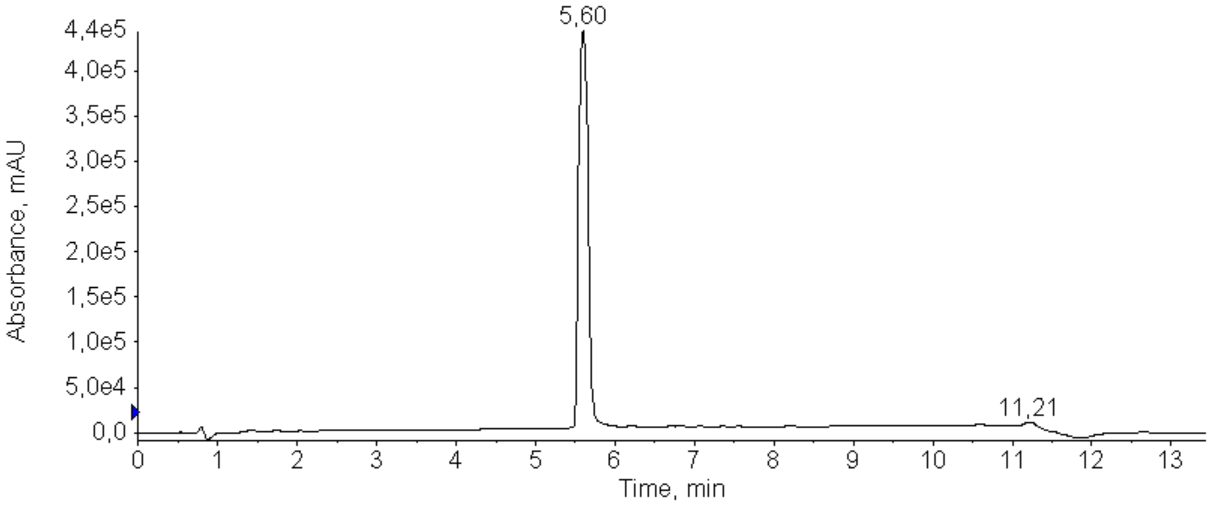

Supporting Information Figure 9: LC/MS analysis of **AE167**. Upper panel: Mass spectrum. Lower panel: according chromatogram.

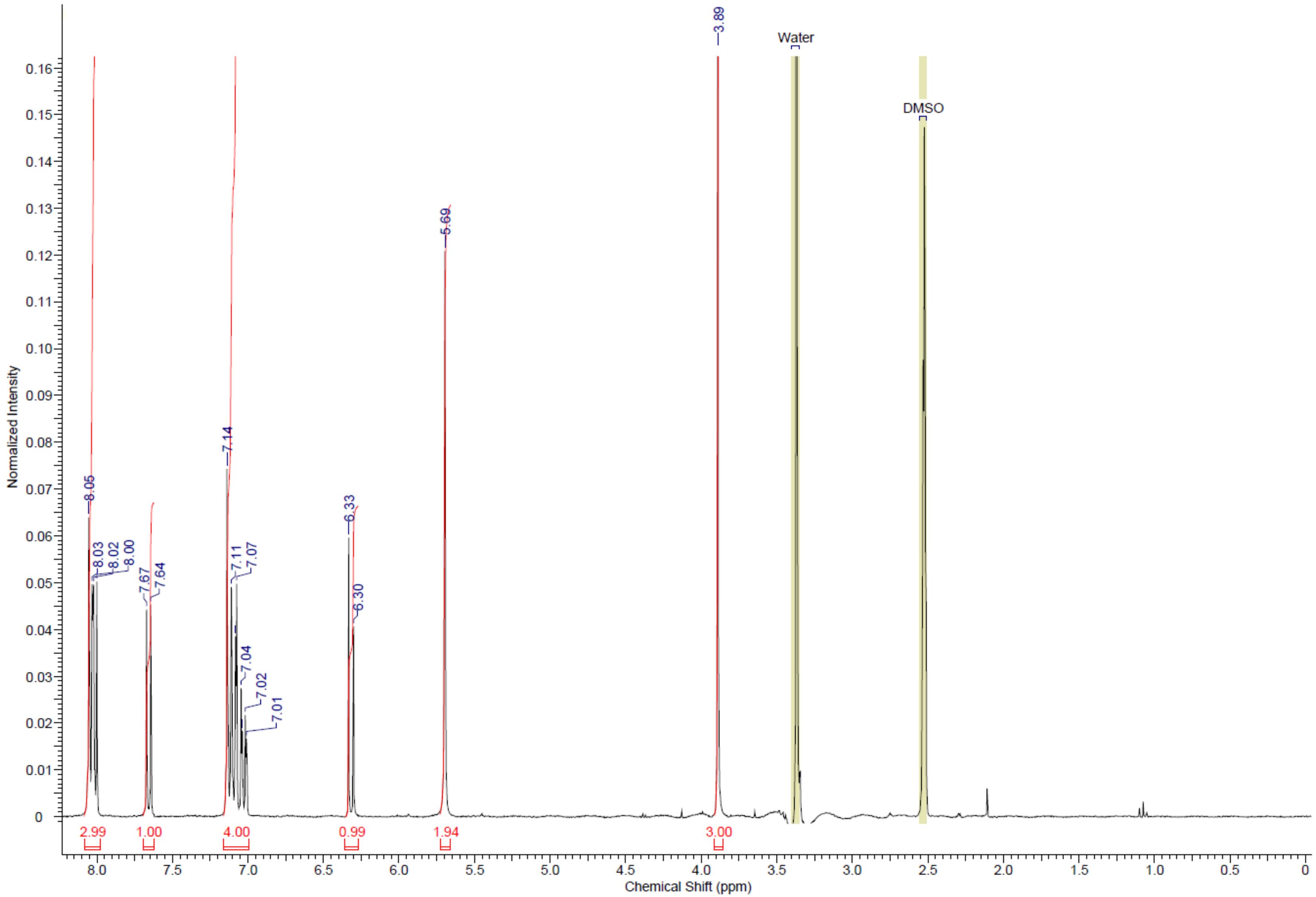

Supporting Information Figure 10: ^{1}H NMR spectrum of **AE167**, 300 MHz, DMSO-d_6.

4.1.6 3-(4-Methoxyphenyl)furo[3,2-*g*]chromen-7-one (**Scaff10-1**)

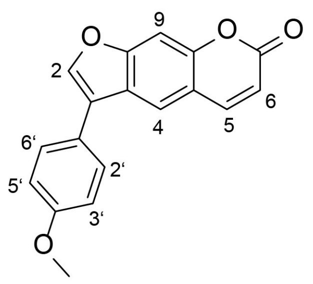

7-[2-(4-Methoxyphenyl)-2-oxo-ethoxy]chromen-2-one (**AE167**) (500 mg, 1.61 mmol) was saponificated in 10 ml 1 M sodium hydroxide at 100 °C for 3 h. Dropwise addition of 1 M H_2SO_4 resulted in precipitation of the product. The precipitated solid was washed with water and recrystallised in ethanol. The final product gave a white solid in 23 % yield (108 mg).

^{1}H NMR (300 MHz, DMSO-d_6): δ(ppm) = 3.77 – 3.97 (s, 3H, -OCH_3), 6.40 – 6.54 (d, J = 9.77 Hz, 1H, -C6-CH_3), 7.02 – 7.21 (d, J = 7.93 Hz, 2H, H-3', -5'), 7.64 – 7.76 (d, J = 7.93 Hz, 2H, H-2', -6'), 7.76 - 7.82 (s, 1H, H-9), 8.17 – 8.28 (d, J = 9.77 Hz, 1H, -C5-CH_3), 8.27 – 8.35 (s, 1H, H-4), 8.36 – 8.46 (s, 1H, H-2)

ESI-MS: cal. 293.04, exp. 293.0 ($[M+H]^+$)

HPLC: 98 % purity at 254 nm

t_R (HPLC): 6.75 min; λ_{max} = 253 nm and 304 nm

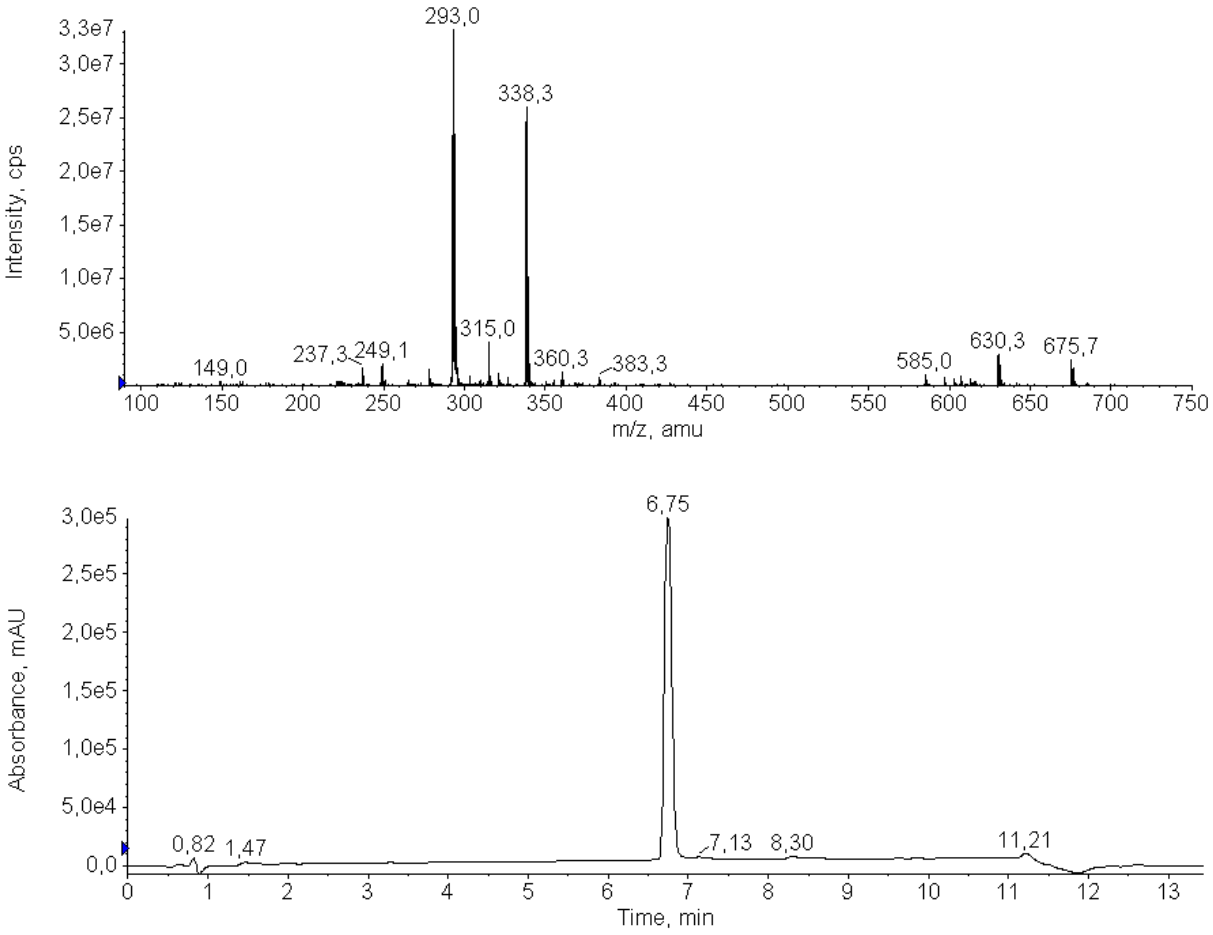

Supporting Information Figure 11.: LC/MS analysis of **Scaff10-1**. Upper panel: Mass spectrum. Lower panel: according chromatogram.

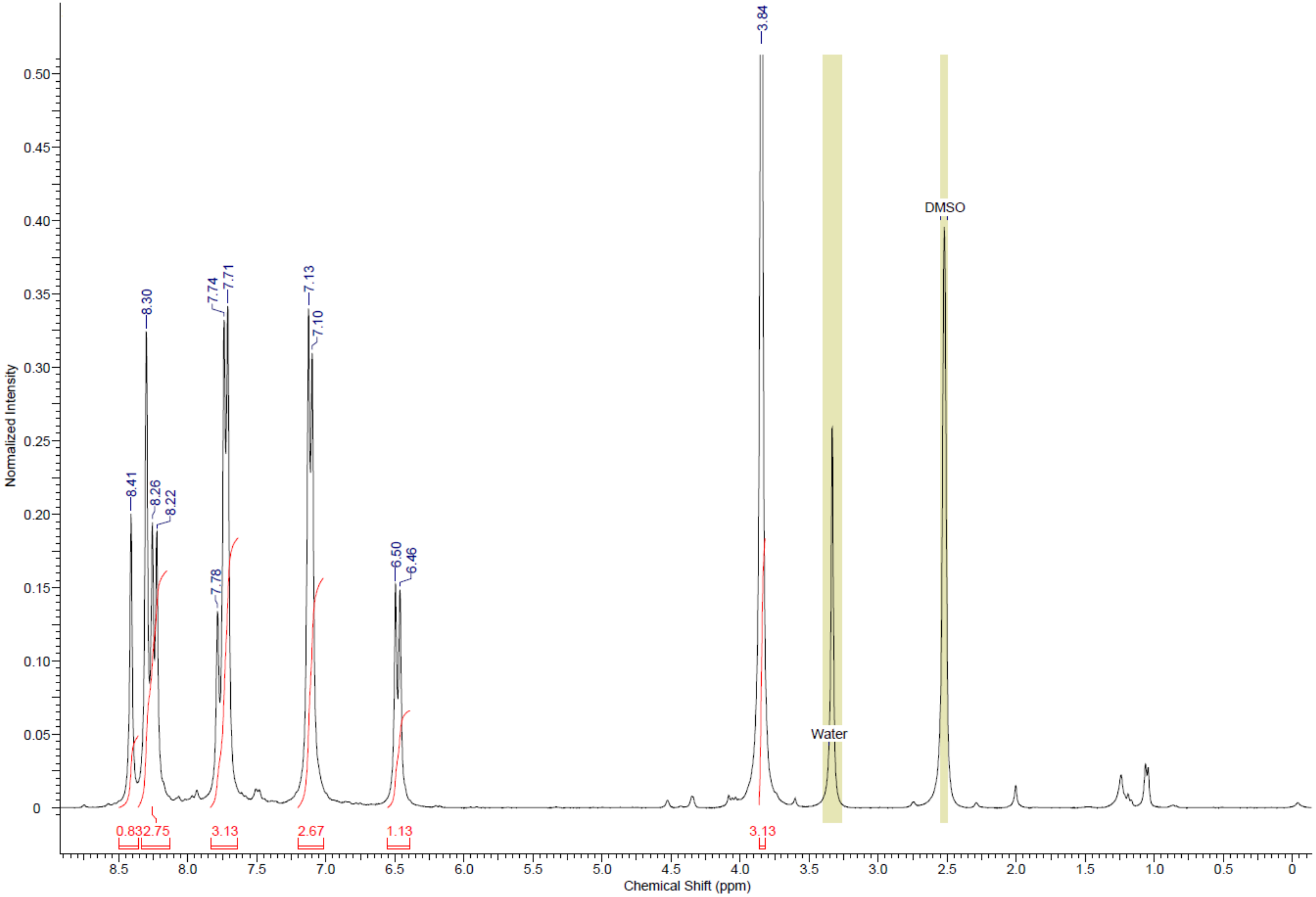

Supporting Information Figure 12: ^{1}H NMR spectrum of **Scaff10-1**, 300 MHz, DMSO-d_6.

4.1.7 3,4-Dimethyl-7-[2-oxo-2-(3-pyridyl)ethoxy]chromen-2-one (**Scaff10-2**)

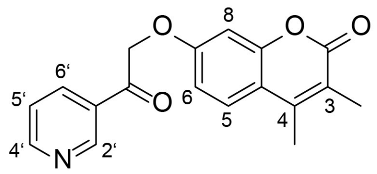

7-Hydroxy-3,4-dimethyl-chromen-2-one (**5b**) (200 mg, 1.05 mmol) was solved in acetone and preactivated with potash in excess (363 mg, 2.63 mmol) at 55 °C. The α-haloketone 2-bromo-1-(3-pyridyl)ethanone (**2c**) was added (323 mg, 1.61 mmol). The mixture was refluxed at the same temperature. The final product after purification gave an orange solid in 14 % yield (46 mg).

^{1}H NMR (300 MHz, DMSO-d_6): δ(ppm) = 1.94 – 2.11 (s, 3H, -C3-CH_3), 2.26 – 2.40 (s, 3H, -C4-CH_3), 5.69 – 5.84 (s, 2H, -CO-C**H₂**-OAr), 6.99 – 7.07 (d, J = 9.77 Hz, 1H, H-6), 7.07 – 7.12 (d, 1H, H-8), 7.60 – 7.74 (m, 2H, H-5, -5'), 8.34 – 8.42 (d, J = 7.33 Hz, 2H, H-6'), 8.82 – 8.89 (d, J = 3.66 Hz, 1H, H-4'), 9.18 9.24 (s, 1H, H-2')

ESI-MS: cal. 310.10, exp. 310.1 ($[M+H]^+$)

HPLC: 98 % purity at 254 nm

t_R (HPLC): 4.90 min; λ_{max} = 278 nm and 317 nm

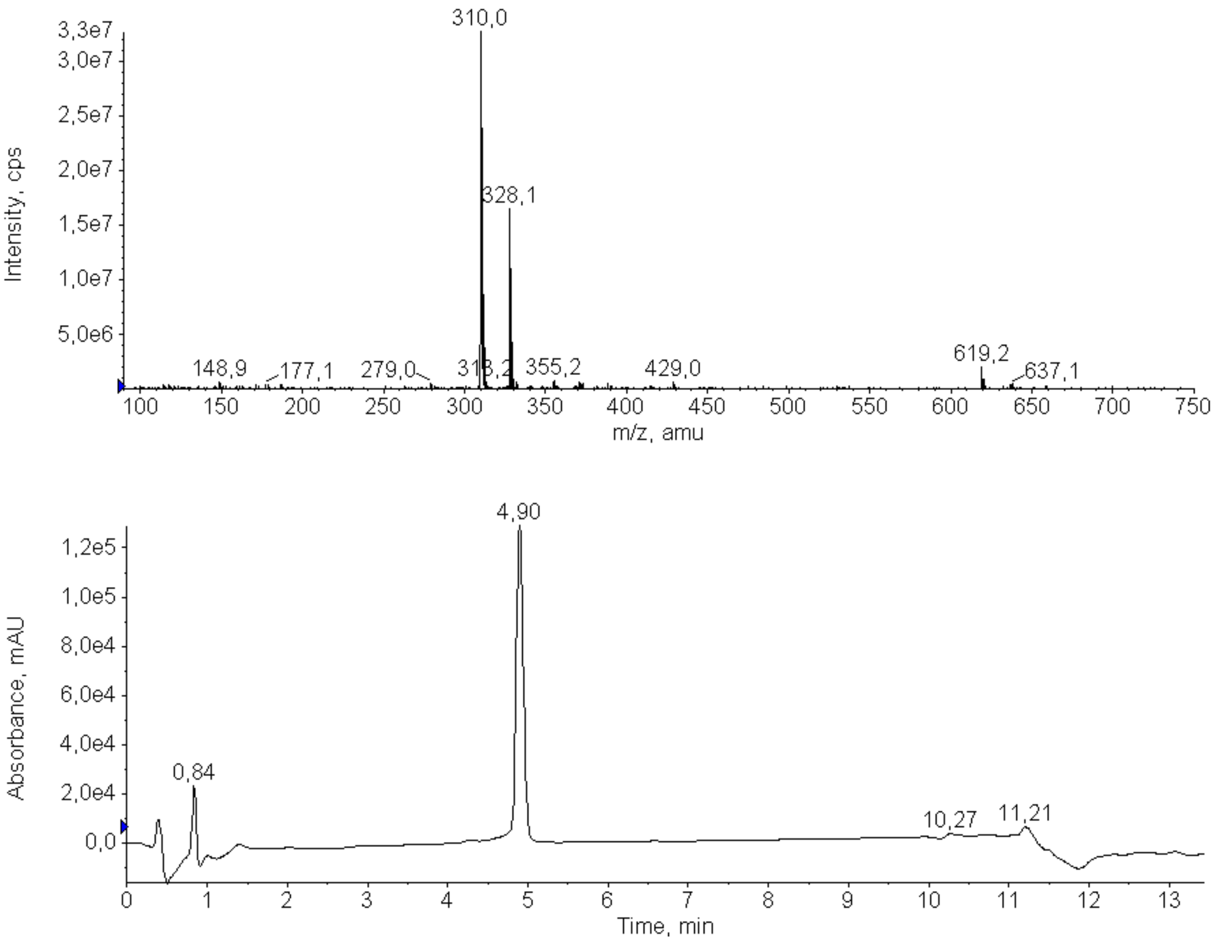

Supporting Information Figure 13: LC/MS analysis of **Scaff10-2**. Upper panel: Mass spectrum. Lower panel: according chromatogram.

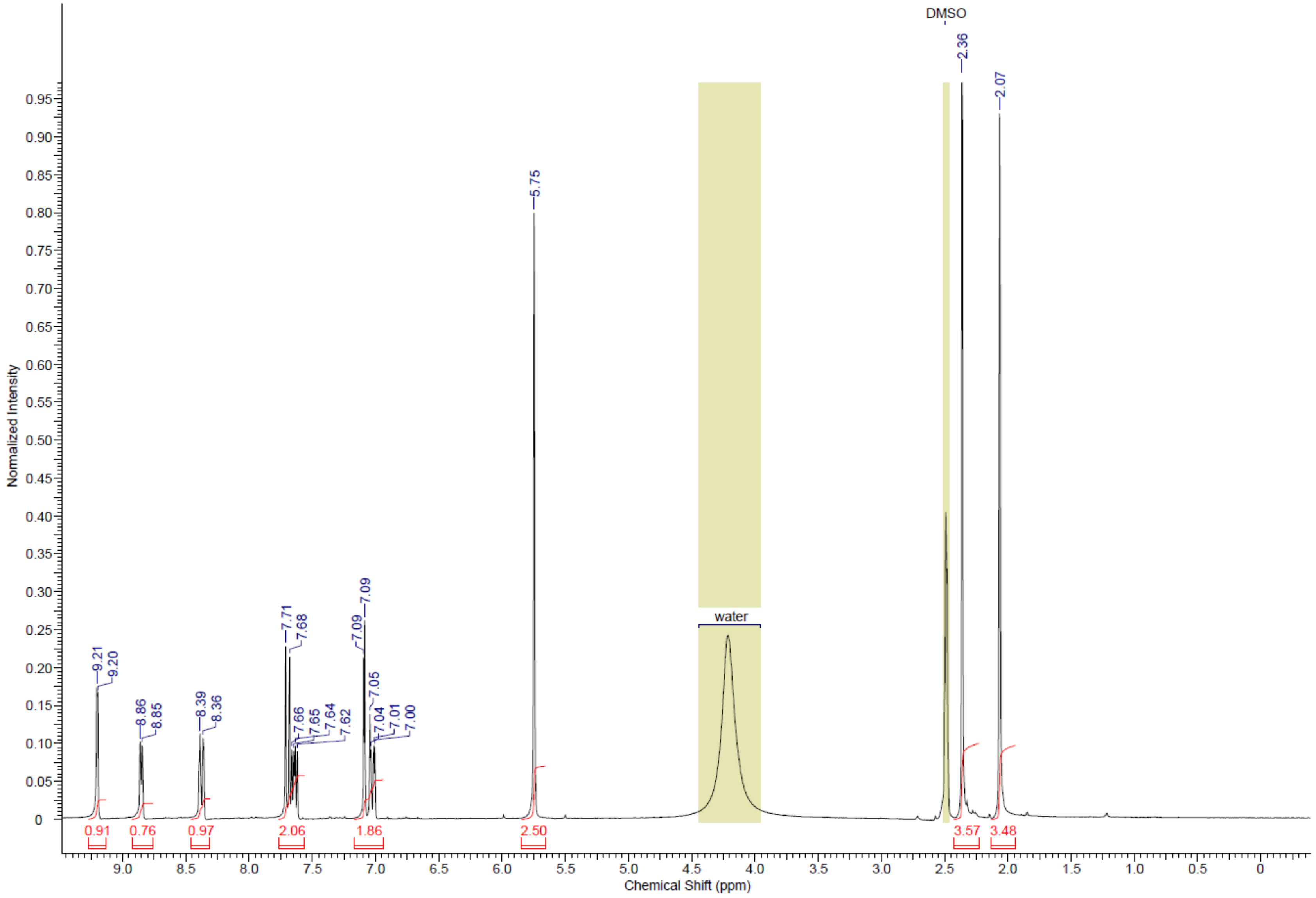

Supporting Information Figure 14: ^{1}H NMR spectrum of **Scaff10-2**, 300 MHz, DMSO-d$_6$.

4.1.8 5,6-Dimethyl-3-(3-pyridyl)furo[3,2-*g*]chromen-7-one (**Scaff10-3**)

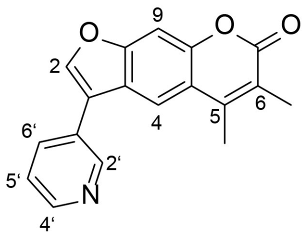

3,4-Dimethyl-7-[2-oxo-2-(3-pyridyl)ethoxy]chromen-2-one (**Scaff10-2**) (200 mg, 0.65 mmol) was saponificated in 1 M sodium hydroxide at 100 °C for 5 h. Dropwise addition of 1 M H_2SO_4 resulted in precipitation of the product. The precipitated solid was purified using preparative HPLC. The final product gave a yellowish solid.

^{1}H NMR (300 MHz, DMSO-d_6): δ(ppm) = 2.12 - 2.21 (s, 3H, -C6-CH_3), 2.55 – 2.62 (s, 3H, -C5-CH_3), 7.59 – 7.66 (m, 1H, H-5’), 7.81 - 7.85 (s, 1H, H-9), 8.19 – 8.24 (s, 1H, H-4), 8.28 – 8.35 (d, J = 8.54 Hz,1H, H-6’), 8.61 – 8.65 (s, 1H, H-2), 8.65 – 8.72 (d, J = 3.66 Hz, 1H, H-4’), 9.05 – 9.12(s, 1H, H-2’)

ESI-MS: cal. 292.09, exp. 292.1 ($[M+H]^+$)

HPLC: 98 % purity at 254 nm

t_R (HPLC): 4.55 min; λ_{max} = 297 nm and 323 nm

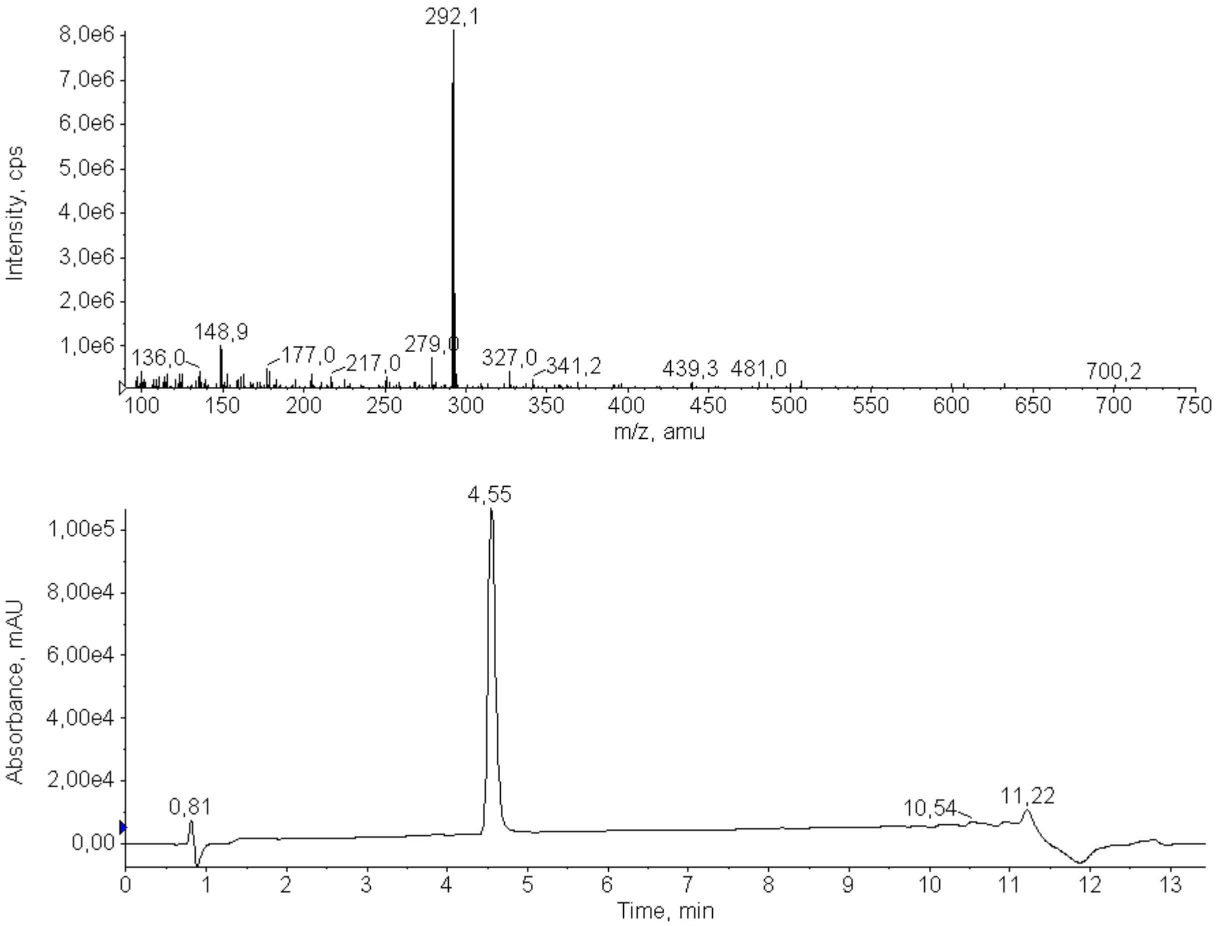

Supporting Information Figure 15: LC/MS analysis of **Scaff10-3**. Upper panel: Mass spectrum. Lower panel: according chromatogram.

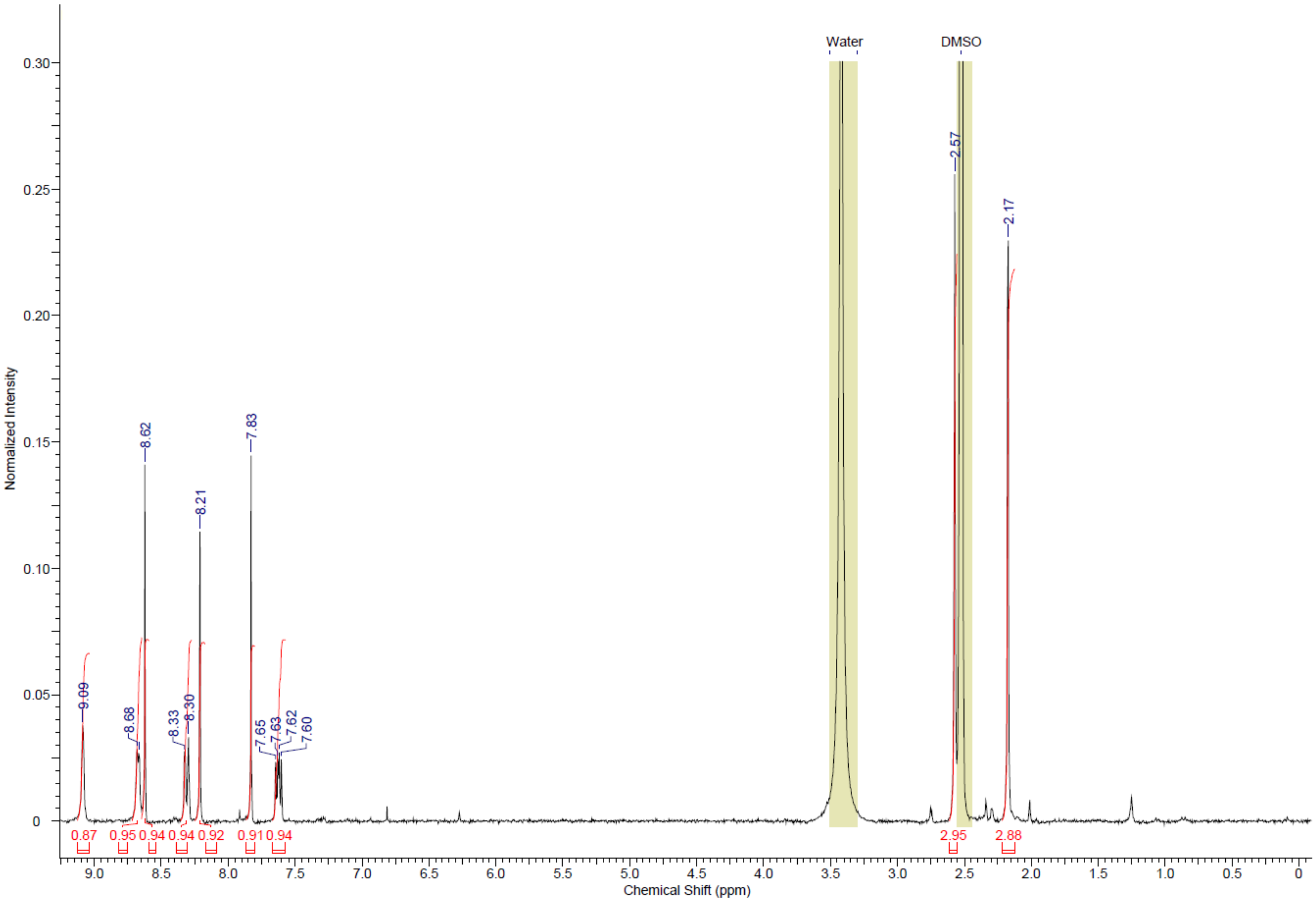

Supporting Information Figure 16: ^{1}H NMR spectrum of **Scaff10-3**, 300 MHz, DMSO-d_6.

4.1.9 7-[2-[6-(4-Fluorophenyl)-2-methyl-3-pyridyl]-2-oxo-ethoxy]-3,4-dimethyl-chromen-2-one (**Scaff10-4**)

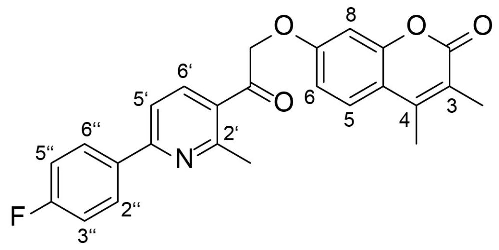

7-Hydroxy-3,4-dimethyl-chromen-2-one(**5b**) (200 mg, 1.05 mmol) was solved in acetone and preactivated with potash in excess (363 mg, 2.63 mmol) by 55 °C. The α-haloketone 2-bromo-1-(6-(4-fluorophenyl)-2-methylpyridin-3-yl)ethanone (**2d**) was added (355 mg, 1.15 mmol). The mixture was refluxed at the same temperature. The final product after purification gave a white solid.

^{1}H NMR (300 MHz, DMSO-d_6): δ(ppm) = 2.05 – 2.15 (s, 3H, -C3-CH_3), 2.37 – 2.44 (s, 3H, -C4-CH_3), 2.69 – 2.76 (s, 3H, -OCH_3), 5.63 – 5.69 (s, 2H, -CO-C**H_2**-OAr), 7.01 – 7.11 (m, 2H, H-6, -8), 7.34 – 7.44 (t, J = 8.54 Hz, 2H, H-3'', -5''), 7.71 – 7.76 (d, J = 9.77 Hz, 1H, H-5), 8.02 – 8.08 (d, J = 8.54 Hz, 1H, H-5'), 8.25 – 8.33 (dd, J = 4.88 Hz, 8.55 Hz, 2H, H-2'', -6''),

ESI-MS: cal. 418.14, exp. 418.1 ($[M+H]^+$)

HPLC: 98 % purity at 254 nm

t_R (HPLC): 7.51 min; λ_{max} = 267 nm and 310 nm

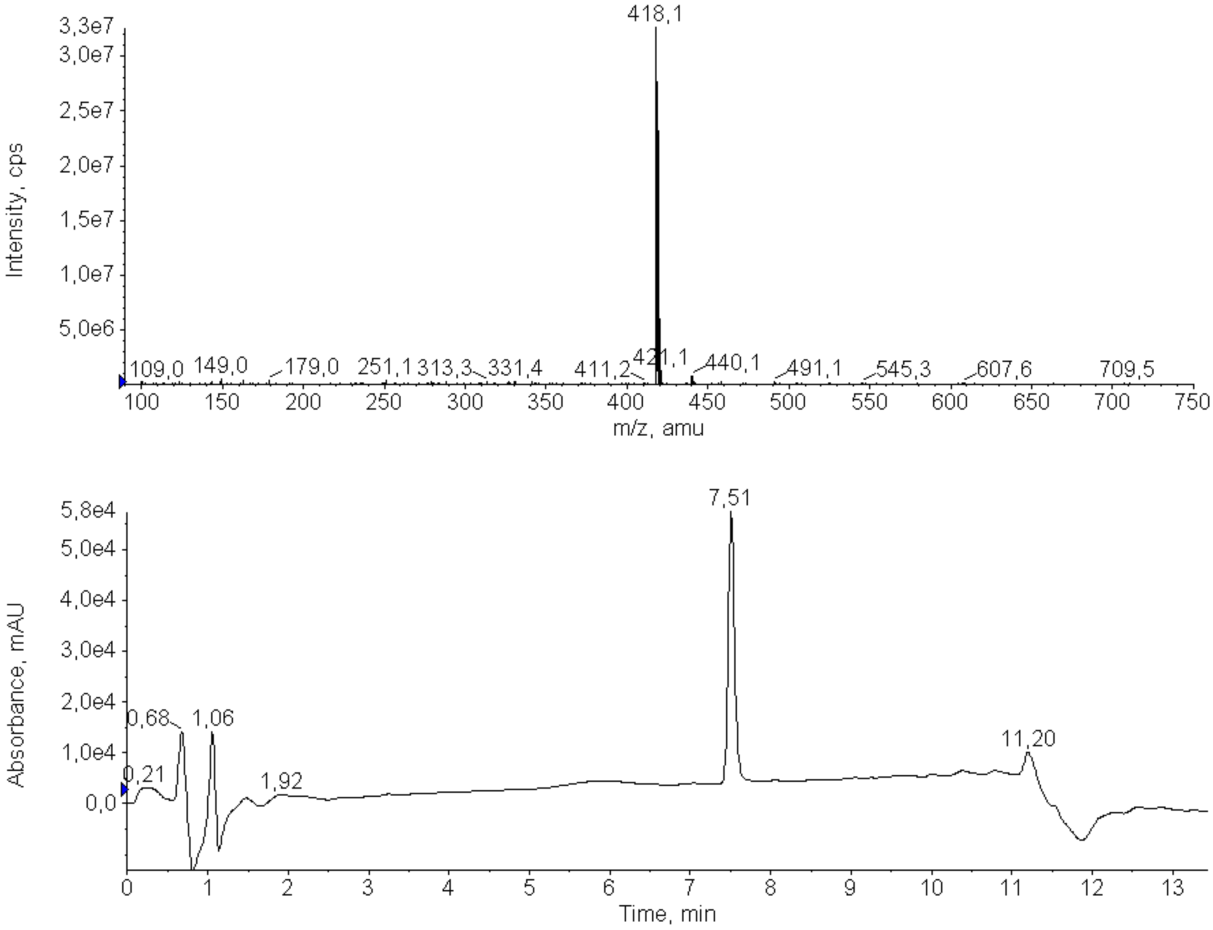

Supporting Information Figure 17: LC/MS analysis of **Scaff10-4**. Upper panel: Mass spectrum. Lower panel: according chromatogram.

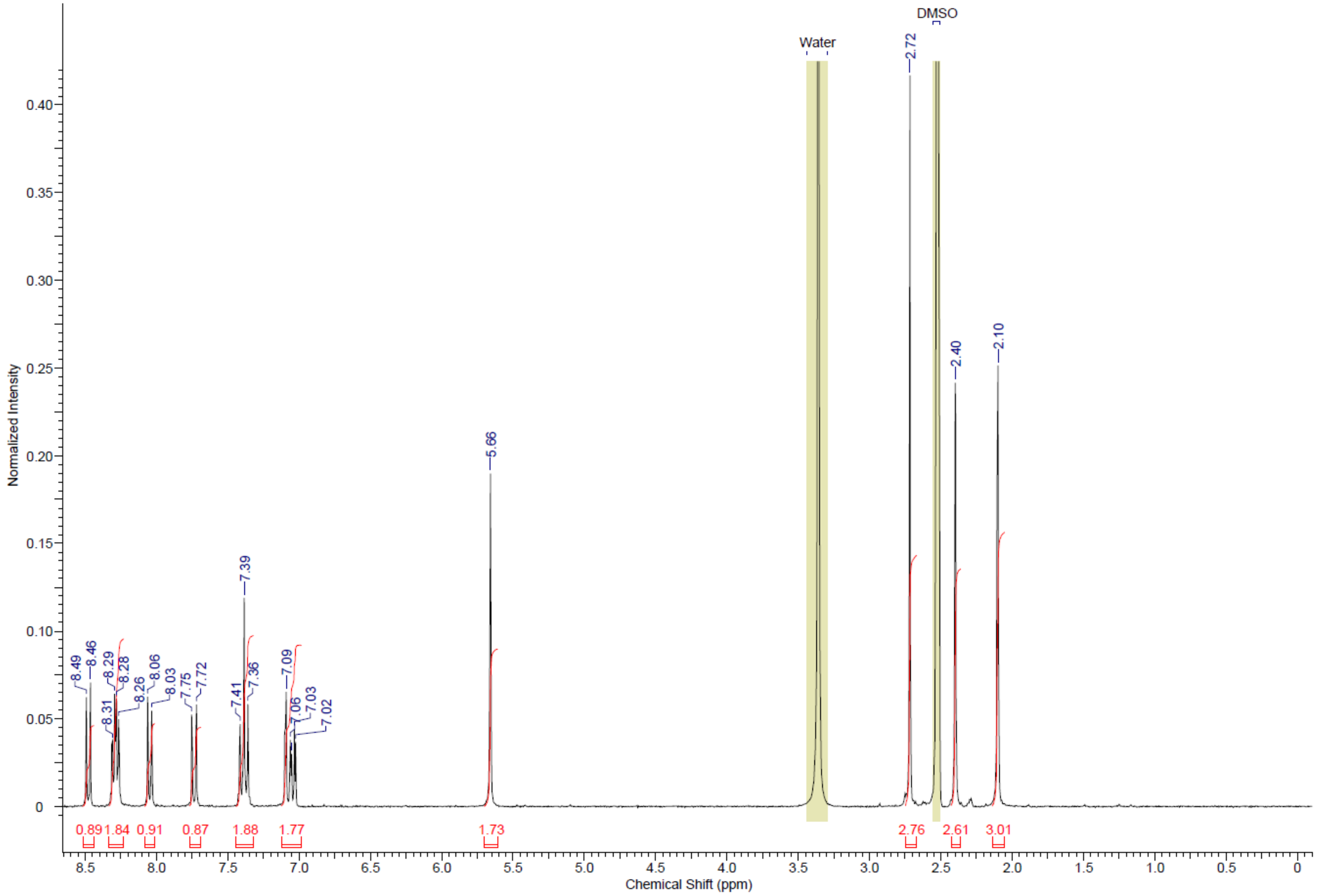

Supporting Information Figure 18: ^{1}H NMR spectrum of **Scaff10-4**, 300 MHz, DMSO-d$_6$.

4.1.10 3-[6-(4-Fluorophenyl)-2-methyl-3-pyridyl]-5,6-dimethyl-furo[3,2-*g*]chromen-7-one (**Scaff10-5**)

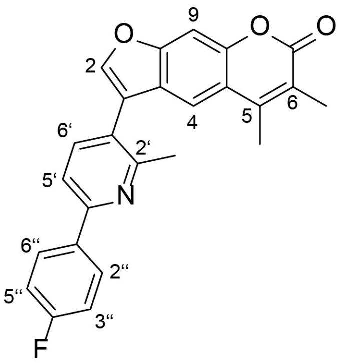

7-[2-[6-(4-Fluorophenyl)-2-methyl-3-pyridyl]-2-oxo-ethoxy]-3,4-dimethyl-chromen-2-one (**Scaff10-4**) (200 mg, 0.48 mmol) was saponificated in 5 ml 1 M sodium hydroxide at 115 °C for 5 h. Dropwise addition of 1 M H_2SO_4 resulted in precipitation of the product. After addition of 10 ml ACN, the precipitated solid was filtered. The final product gave a white solid in 71 % yield (136 mg).

^{1}H NMR (300 MHz, DMSO-d$_6$): δ(ppm) = 2.10 – 2.16 (s, 3H, -C6-CH_3), 2.40 – 2.48 (s, 3H, -C2'-CH_3), 2.54 – 2.62 (s, 3H, -C5-CH_3), 7.30 – 7.39 (m, 2H, H-5', -6'), 7.79 - 7.87 (d, J = 13.43 Hz, 2H, H-3'', -5''), 7.93 – 7.97 (m, 2H, H-4, -9), 8.18 – 8.26 (m, 2H, H-2'', -6''), 8.34 – 8.37 (s, 1H, H-2)

ESI-MS: cal. 400.13, exp. 400.1 ($[M+H]^+$)

HPLC: 96 % purity at 254 nm

t_R (HPLC): 8.11 min; λ_{max} = 245 nm and 299 nm

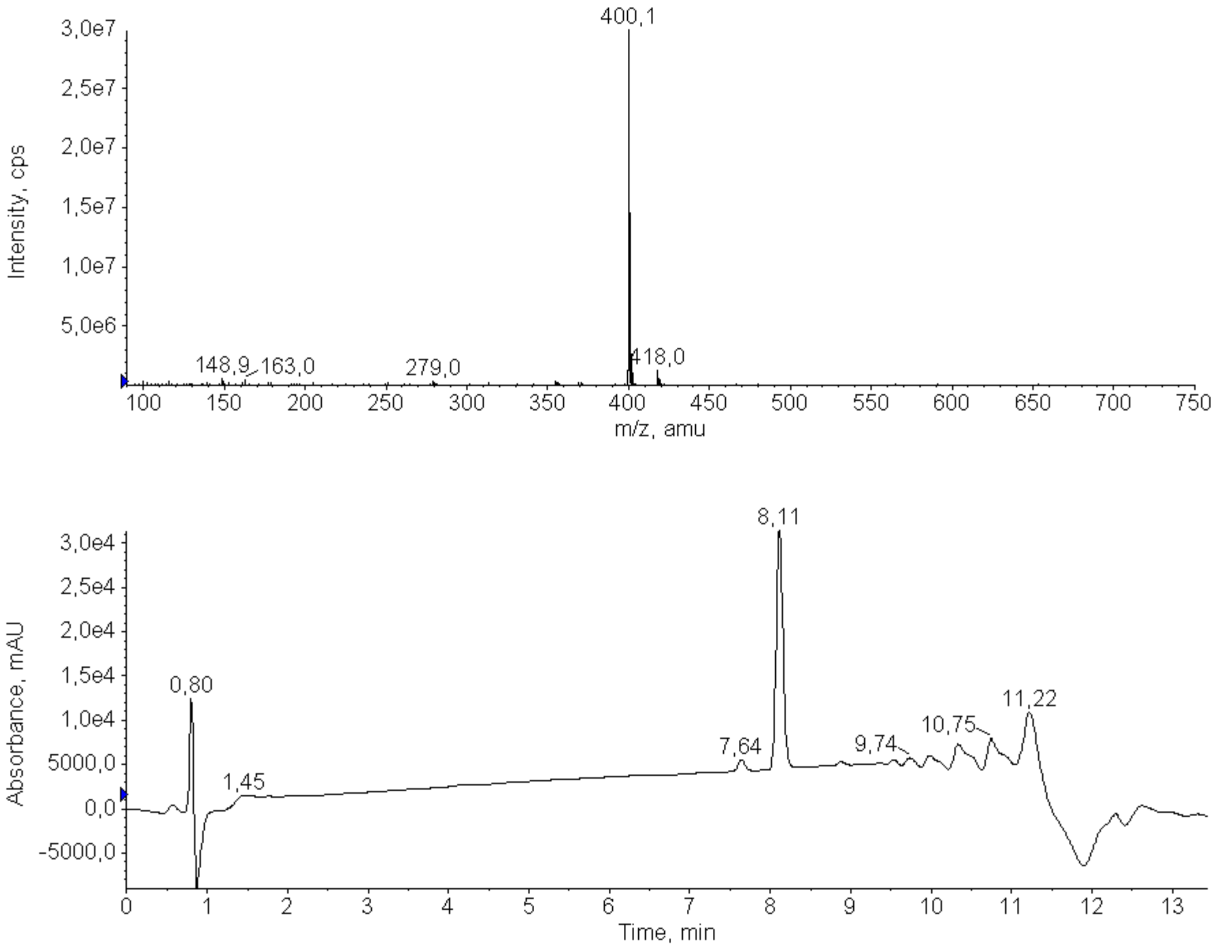

Supporting Information Figure 19: LC/MS analysis of **Scaff10-5**. Upper panel: Mass spectrum. Lower panel: according chromatogram.

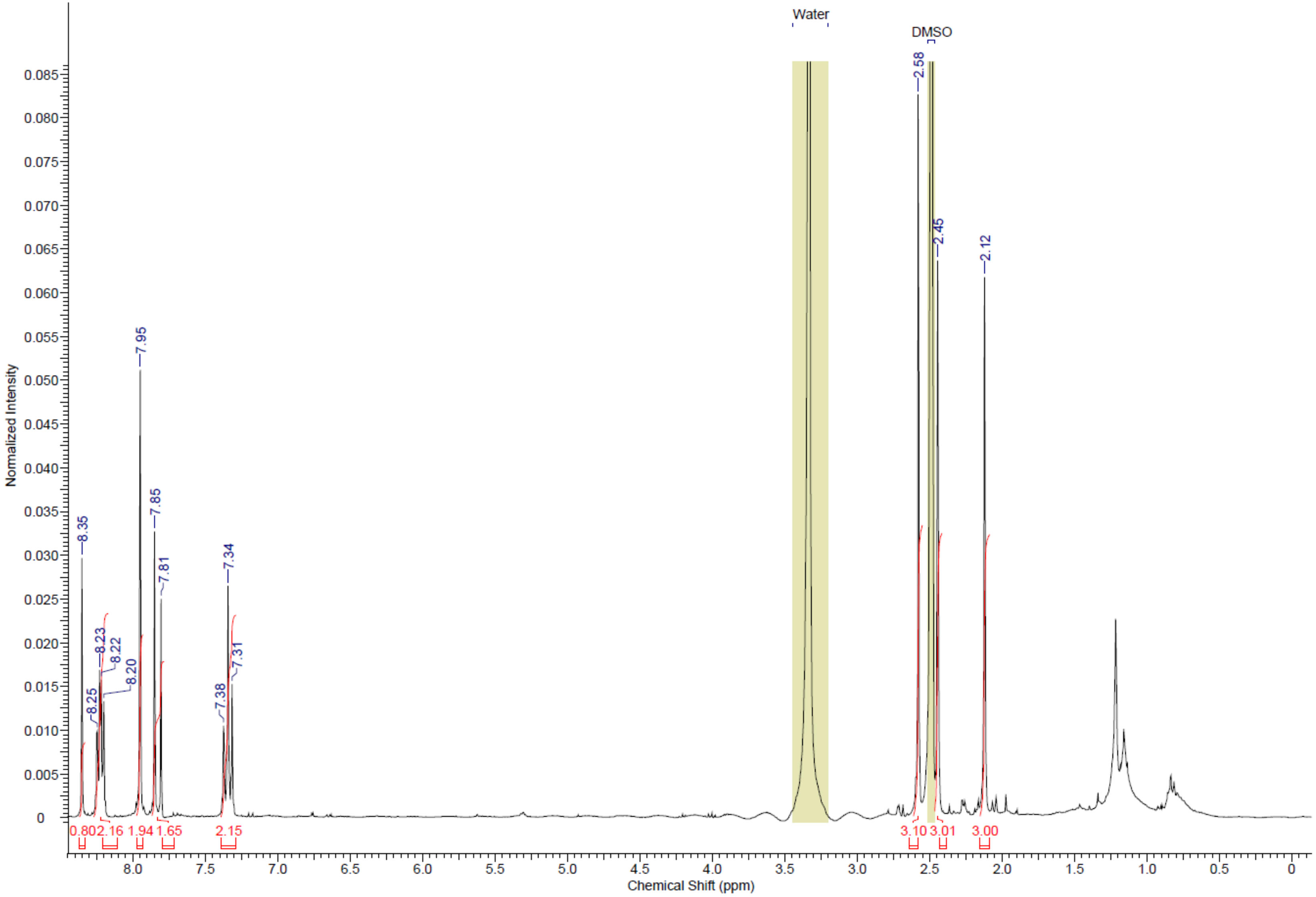

Supporting Information Figure 20: ^{1}H NMR spectrum of **Scaff10-5**, 300 MHz, DMSO-d_6.

4.1.11 3-[7-[2-(4-Methoxyphenyl)-2-oxo-ethoxy]-4-methyl-2-oxo-chromen-3-yl]propanoic acid (**Scaff10-6**)

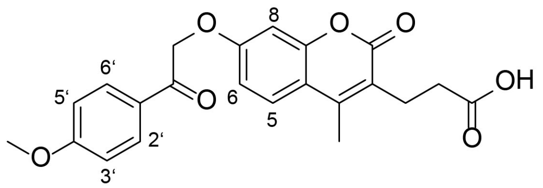

Ethyl 3-[7-[2-(4-methoxyphenyl)-2-oxo-ethoxy]-4-methyl-2-oxo-chromen-3-yl]propanoate (**Scaff10-7**) (100 mg, 0.24 mmol) was saponificated in a mixture of 6 ml dry acetone and 6 ml 1 M sodium hydroxide at 55 °C for 16 h. Dropwise addition of 1 M HCl resulted in precipitation of the product. The precipitated solid was purified using preparative HPLC. The product was obtained after lyophilisation a yellowish solid.

^{1}H NMR (300 MHz, DMSO-d_6): δ(ppm) = 1.91 – 2.01 (t, J = 8.54 Hz, 2H, -CH_2-C$\mathbf{H_2}$-COOH), 2.37 – 2.45 (s, 3H, Ar-CH_3), 2.63 – 2.73 (t, J = 8.54 Hz, 2H, -C$\mathbf{H_2}$-CH_2-COOH), 3.86 – 3.91 (s, 3H, -OCH_3), 5.64 – 5.69 (s, 2H, -CO-C$\mathbf{H_2}$-OAr), 6.97 – 7.03 (m, 2H, H-6, -8), 7.09 – 7.15 (d, J = 8.55 Hz, 2H, H-3’, -5’), 7.66 – 7.71 (d, J = 8.54 Hz , 1H, H-5), 8.02 – 8.07 (d, J = 8.54 Hz, 2H, H-2’, -6’)

ESI-MS: cal. 397.12, exp. 397.1 ([M+H]$^+$)

HPLC: 95 % purity at 254 nm

t_R (HPLC): 5.38 min; λ_{max} = 284 nm and 317 nm

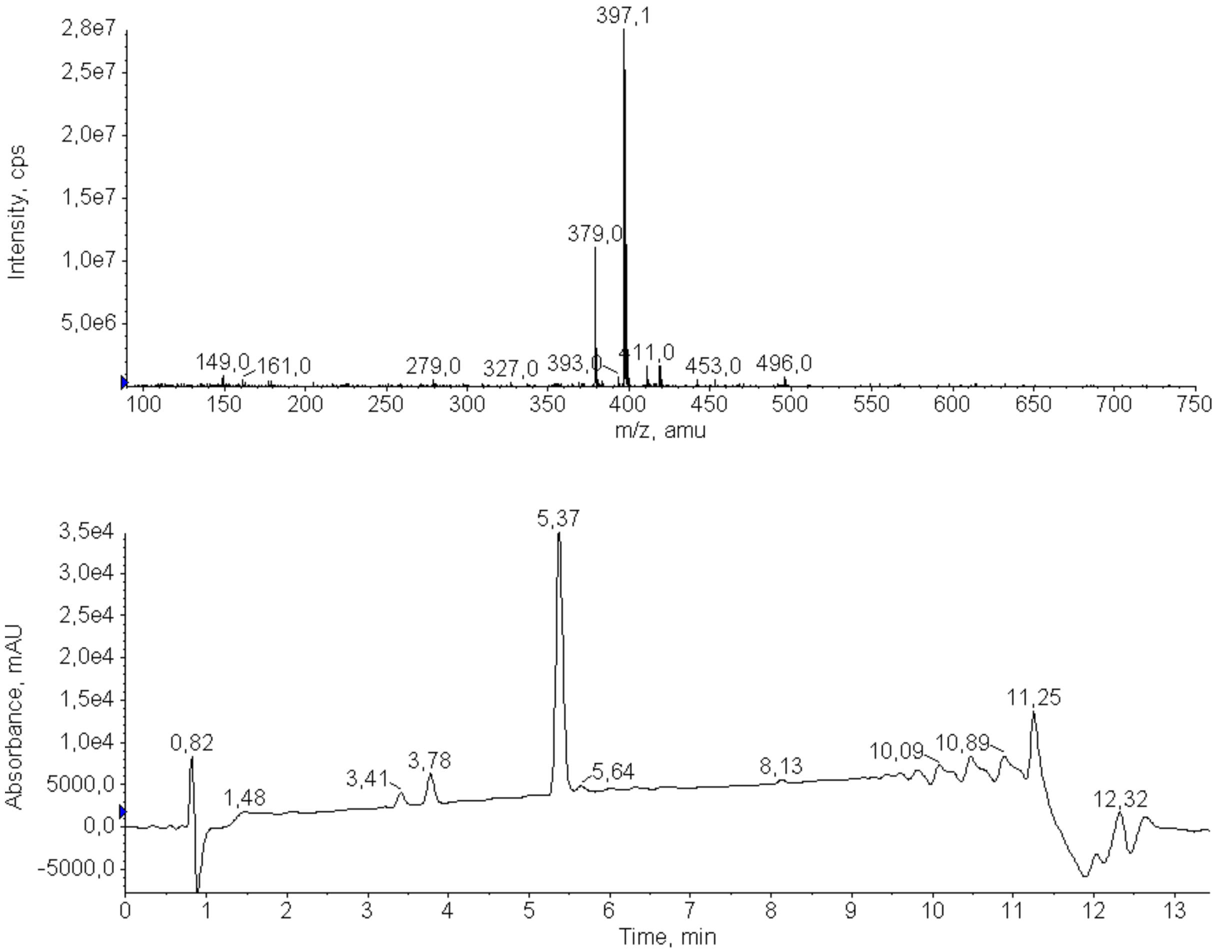

Supporting Information Figure 21: LC/MS analysis of **Scaff10-6**. Upper panel: Mass spectrum. Lower panel: according chromatogram.

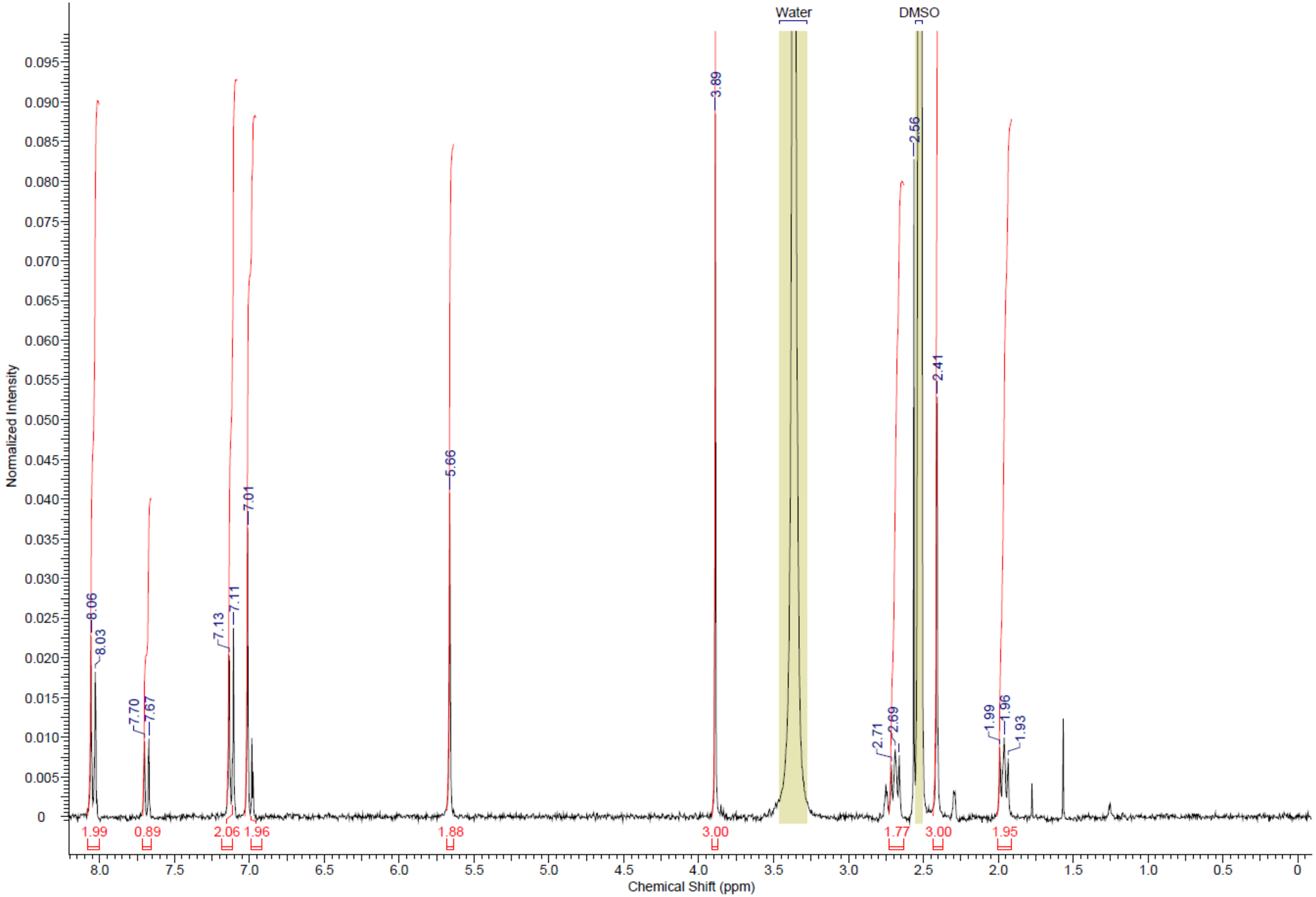

Supporting Information Figure 22: ^{1}H NMR spectrum of **Scaff10-6**, 300 MHz, DMSO-d_6.

4.1.12 Ethyl 3-[7-[2-(4-methoxyphenyl)-2-oxo-ethoxy]-4-methyl-2-oxo-chromen-3-yl]propanoate (**Scaff10-7**)

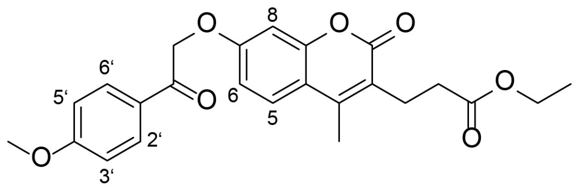

Ethyl 3-(7-hydroxy-4-methyl-2-oxo-chromen-3-yl)propanoate (**5a**) (100 mg, 0.36 mmol) was solved in acetone and preactivated with potash in excess (125 mg, 0.90 mmol) by 55 °C. The α-haloketone 2-bromo-1-(4-methoxyphenyl)ethanone (**2b**) was added (90 mg, 0.39 mmol). The mixture was refluxed at the same temperature for 16 h. The product was precipitated using 1 M H_2SO_4 and and recrystallised in ethanol. The final product gave a white solid in 75 % yield (114 mg).

^1H NMR (300 MHz, DMSO-d_6): δ(ppm) = 1.11 – 1.23 (t, J = 7.32 Hz, 3H, -CH_2-C**H_3**), 2.39 – 2.45 (s, 3H, Ar-CH_3), 2.46 – 2.53 (t, J = 7.32 Hz, 2H, -CH_2-C**H_2**-COOEt), 2.77 – 2.88 (t, J = 7.32 Hz, 2H, -C**H_2**-CH_2-COOEt), 3.86 – 3.92 (s, 3H, -OCH_3), 4.01 – 4.11 (q, J = 7.32 Hz, 2H, -C**H_2**-CH_3), 5.66 – 5.72 (s, 2H, -CO-C**H_2**-OAr), 6.99 – 7.07 (m, 2H, H6, -8), 7.09 – 7.16 (d, J = 8.55 Hz, 2H, H-3', -5'), 7.71 – 7.77 (d, J = 8.55 Hz, 1H, H-5), 8.01 – 8.07 (d, J = 9.77 Hz, 2H, H-2', -6')

ESI-MS: cal. 425.16, exp. 425.1 ($[M+H]^+$)

HPLC: 99 % purity at 254 nm

t_R (HPLC): 6.69 min; λmax = 284 nm and 318 nm

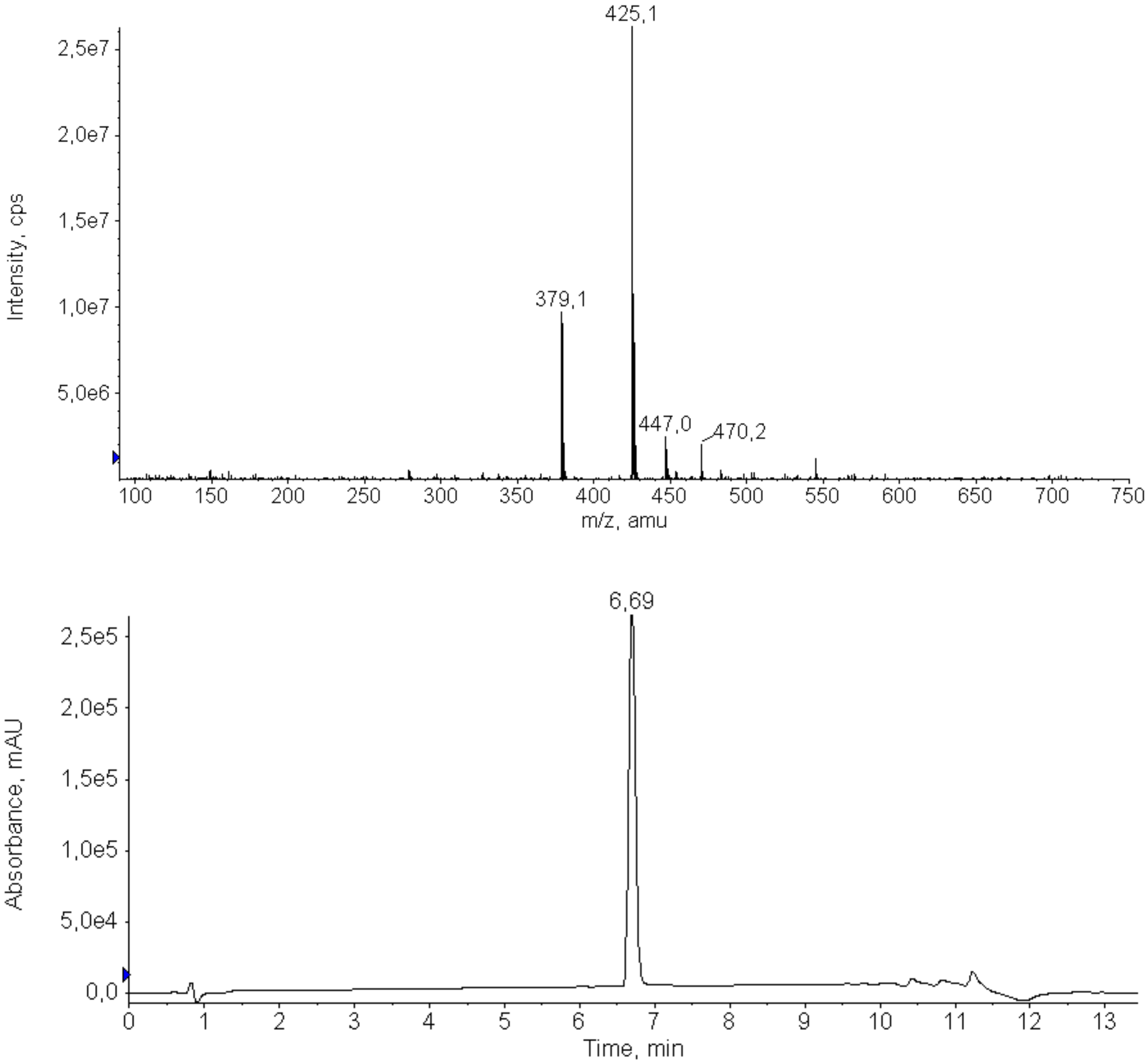

Supporting Information Figure 23.: LC/MS analysis of **Scaff10-7**. Upper panel: Mass spectrum. Lower panel: according chromatogram.

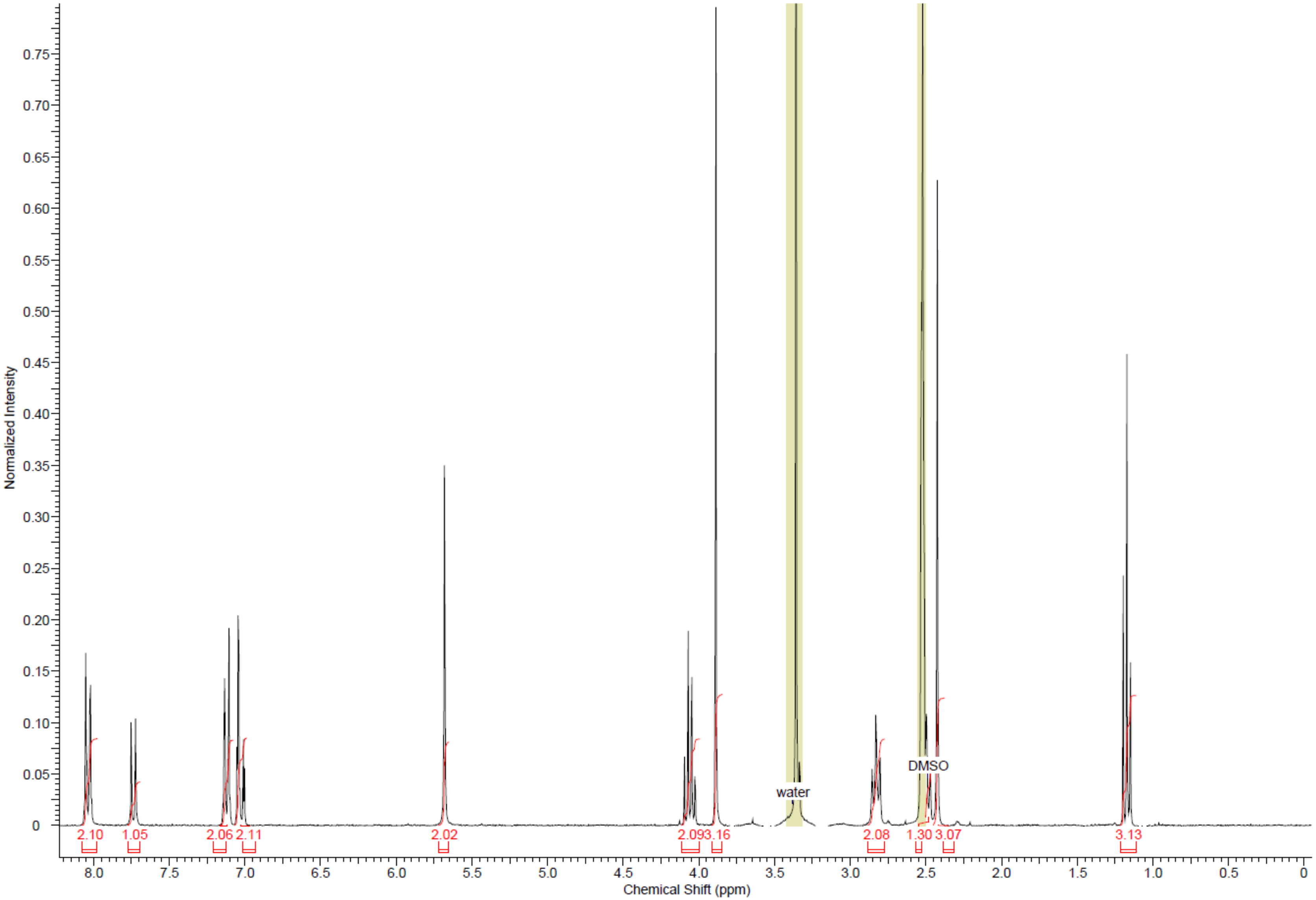

Supporting Information Figure 24: ^{1}H NMR spectrum of **Scaff10-7**, 300 MHz, DMSO-d_6.

4.1.13 3-[3-(4-Methoxyphenyl)-5-methyl-7-oxo-furo[3,2-*g*]chromen-6-yl] propanoic acid (**Scaff10-8**)

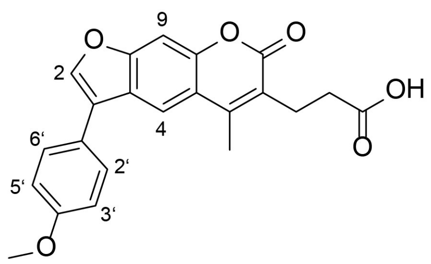

Ethyl 3-[7-[2-(4-methoxyphenyl)-2-oxo-ethoxy]-4-methyl-2-oxo-chromen-3-yl]propanoate (**Scaff10-7**) (200 mg, 0.47 mmol) was saponificated in a mixture of 6 ml dry acetone and 6 ml 1 M sodium hydroxide at 95 °C for 3 h. Dropwise addition of 1 M HCl resulted in precipitation of the product. The precipitated solid was filtered and purified using preparative HPLC with the following gradient: 40 % to 95 % ACN in H_2O in 80 min. The product eluted at t_R = 36.5 min and was obtained after lyophilisation as yellowish solid in 80 % yield (142 mg).

^{1}H NMR (300 MHz, DMSO-d_6): δ(ppm) = 2.39 - 2.46 (t, J = 7.32 Hz, 2H, -CH_2-C**H_2**-COOH), 2.54 – 2.60 (s, 3H, Ar-CH_3), 2.80 – 2.89 (t, J = 7.32 Hz, 2H, -C**H_2**-CH_2-COOH), 3.79 – 3.86 (s, 3H, -OCH_3), 7.07 - 7.15 (d, J = 8.55 Hz, 2H, H-3', -5'), 7.71 – 7.78 (m, 3H, H-9, -2', -6'), 8.11 – 8.15 (s, 1H, H-4), 8.34 – 8.40 (s, 1H, H-2)

ESI-MS: cal. 379.11, exp. 379.1 ($[M+H]^+$)

HPLC: 99 % purity at 254 nm

t_R (HPLC): 6.35 min; λ_{max} = 251 nm and 308 nm

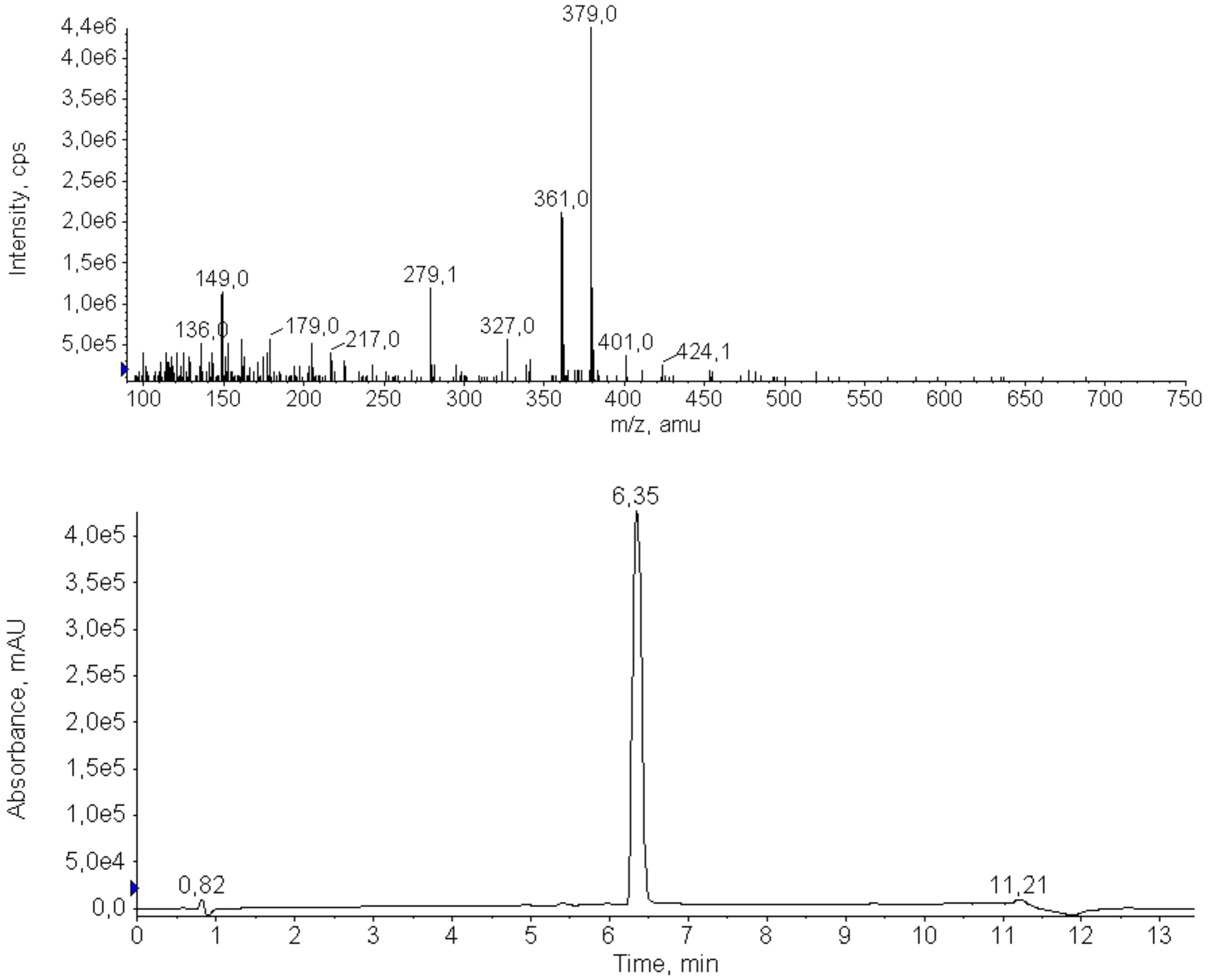

Supporting Information Figure 25: LC/MS analysis of **Scaff10-8**. Upper panel: Mass spectrum. Lower panel: according chromatogram.

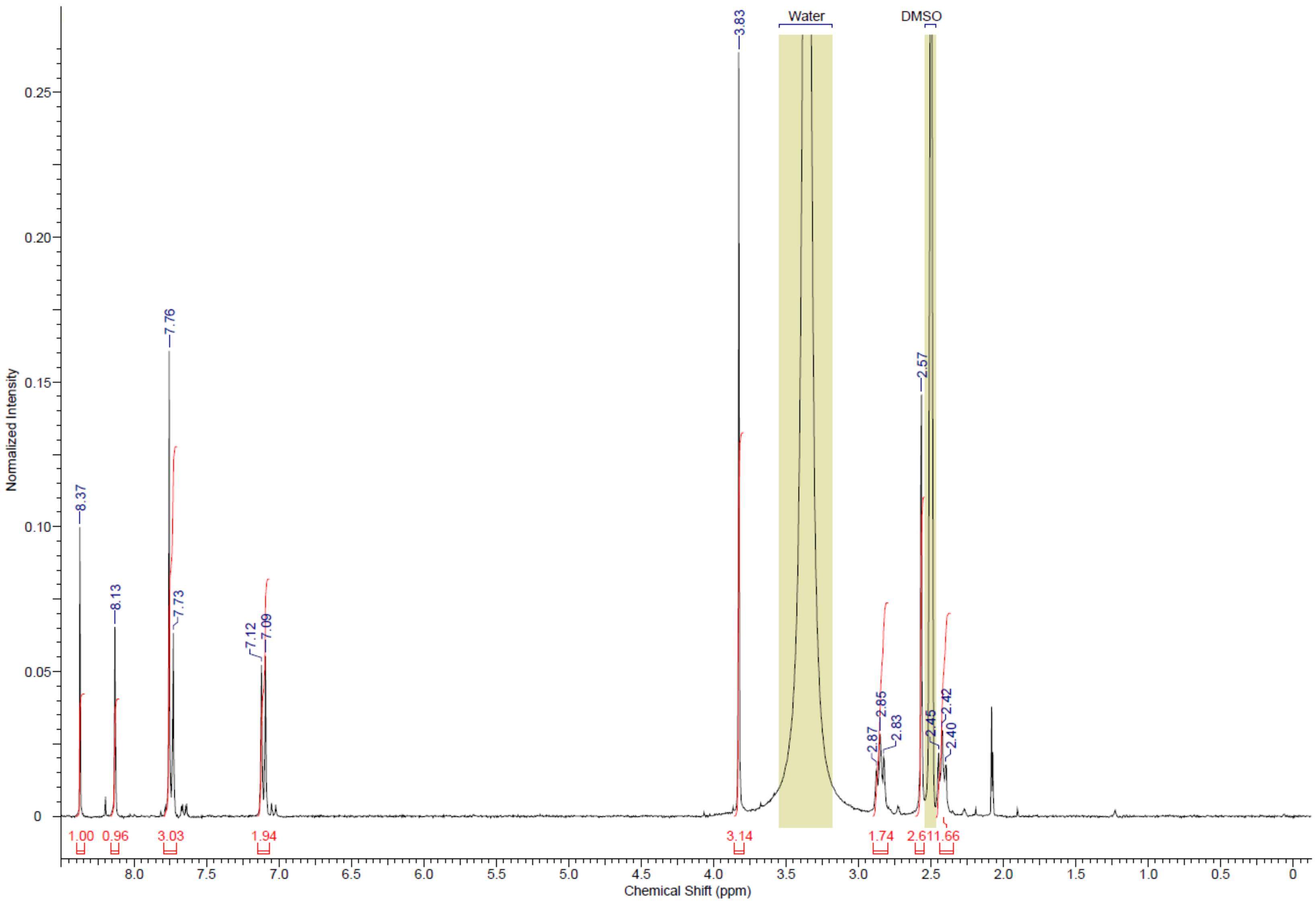

Supporting Information Figure 26: ^{1}H NMR spectrum of **Scaff10-8**, 300 MHz, DMSO-d_6.

4.1.14 *N*-[2-[3-[7-[2-(4-Methoxyphenyl)-2-oxo-ethoxy]-4-methyl-2-oxo-chromen-3-yl]propanoylamino]ethyl]-5-(2-oxo-1,3,3*a*,4,6,6*a*-hexahydrothieno[3,4-*d*]imidazol-4-yl)pentanamide (**Scaff10-9**)

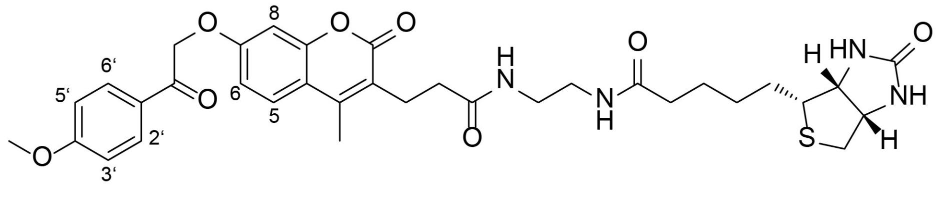

Ethyl 3-[7-[2-(4-methoxyphenyl)-2-oxo-ethoxy]-4-methyl-2-oxo-chromen-3-yl]propanoate (**Scaff10-7**) (10 mg, 0.03 mmol) was condensed with *N*-(2-aminoethyl)-5-(2-oxo-1,3,3a,4,6,6*a*-hexahydrothieno[3,4-*d*]imidazol-4-yl)pentanamide (8.60 mg, 0.03 mmol) catalysed by 1-[bis(dimethylamino)methylene]-1*H*-1,2,3-triazolo[4,5-*b*]pyridinium 3-oxid hexafluorophosphate (HATU) (11.4 mg, 0.03 mmol) and *N*,*N*-diisopropylethylamine (DIPEA) as base (12.6 mg, 0.12 mmol). The final product gave a white solid.

No ^{1}H NMR spectrum available.

ESI-MS: cal. 665.26, exp. 665.3 ($[M+H]^+$)

HPLC: 96 % purity at 254 nm

t_R (HPLC): 4.72 min; λ_{max} = 286 nm and 319 nm

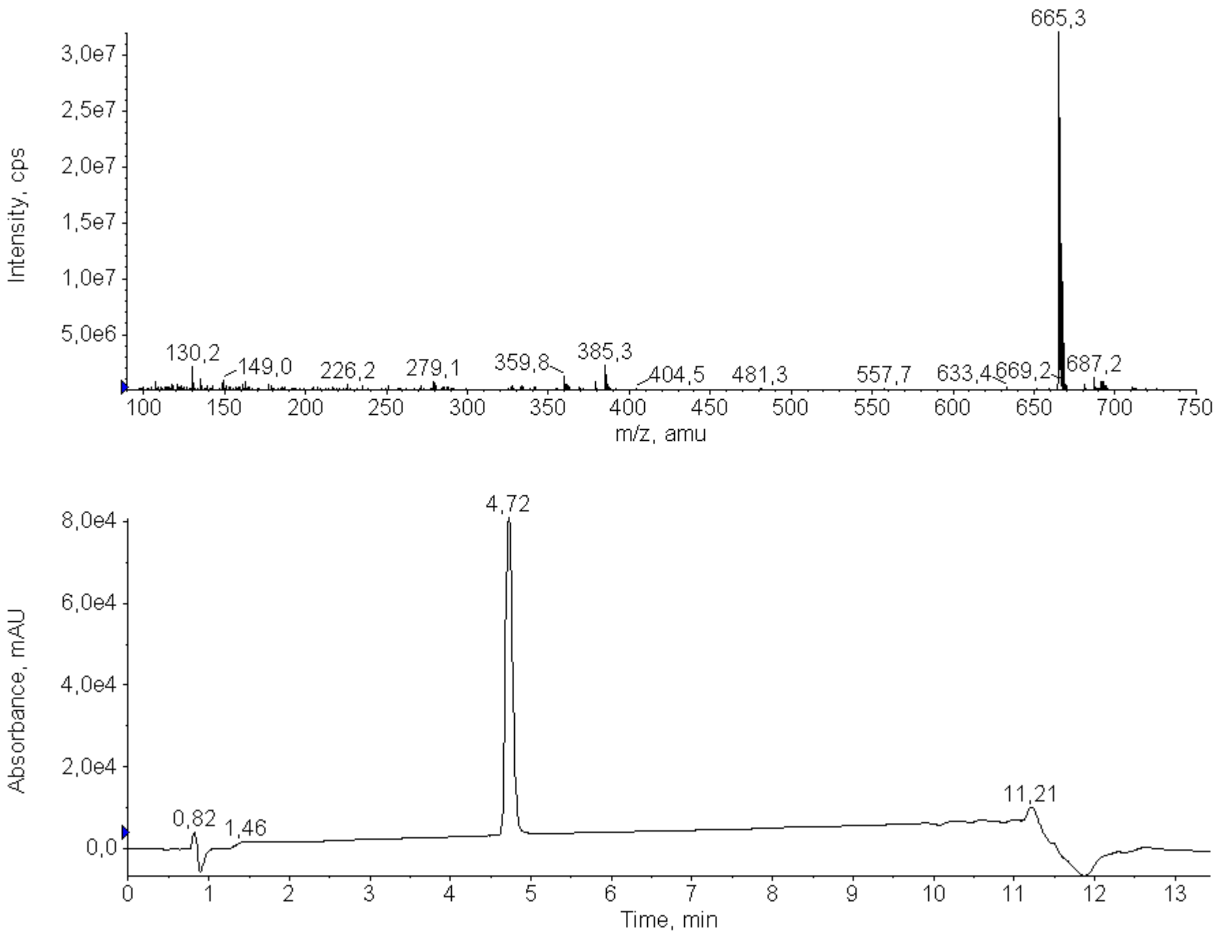

Supporting Information Figure 27: LC/MS analysis of **Scaff10-9**. Upper panel: Mass spectrum. Lower panel: according chromatogram.

4.1.15 Ethyl 3-[7-[2-[6-(4-fluorophenyl)-2-methyl-3-pyridyl]-2-oxo-ethoxy]-4-methyl-2-oxo-chromen-3-yl]propanoate (**Scaff10-10**)

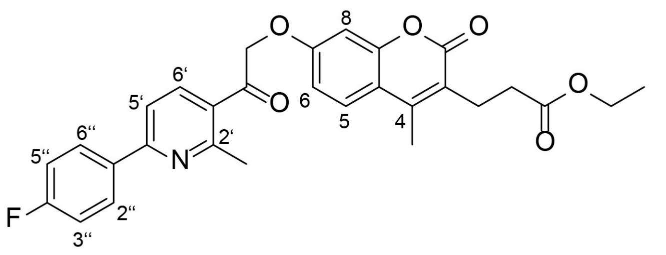

Ethyl 3-(7-hydroxy-4-methyl-2-oxo-chromen-3-yl)propanoate (**5a**) (100 mg, 0.36 mmol) was solved in 6 ml dry acetone and preactivated with potash in excess (125 mg, 0.90 mmol) by 55 °C. The α-haloketone 2-bromo-1-(6-(4-fluorophenyl)-2-methylpyridin-3-yl)ethanone (**2d**) was added (127 mg, 0.41 mmol). The mixture was refluxed at the same temperature for 16 h. The product was precipitated using 1 M H_2SO_4 and recrystallised in ethanol. The final product gave a white solid in 66 % yield (120 mg).

^{1}H NMR (300 MHz, DMSO-d_6): δ(ppm) = 1.10 – 1.21 (t, J = 7.32 Hz, 3H, -CH_2-C**H₃**), 2.04 – 2.36 (s, 3H, Ar-CH_3), 2.43 – 2.52 (t, J = 8.54 Hz, 2H, -CH_2-C**H₂**-COOEt), 2.63 – 2.75 (s, 3H, -C2'-CH_3), 2.76 – 2.87 (t, J = 8.54 Hz, 2H, -C**H₂**-CH_2-COOEt), 4.00 – 4.09 (q, J = 7.32 Hz, 2H, -C**H₂**-CH_3), 5.60 – 5.68 (s, 2H, -CO-C**H₂**-OAr), 6.99 – 7.06 (d, J = 8.55 Hz, 1H, H-6), 7.06 – 7.10 (s, 1H, H-8), 7.31 – 7.42 (t, J = 8.54 Hz, 2H, H-3'', -5''), 7.69 – 7.77 (d, J = 9.77 Hz, 1H, H-5'), 7.99 – 8.06 (d, J = 8.55 Hz, 1H, H-5), 8.22 – 8.31 (d, J = 8.55 Hz , 2H, H-2'', -6''), 8.42 – 8.49 (d, J = 9.77 Hz, 1H, H-6')

ESI-MS: cal. 504.18, exp. 504.1 ($[M+H]^+$)

HPLC: 97 % purity at 254 nm

t_R (HPLC): 7.28 min; λ_{max} = 310 nm

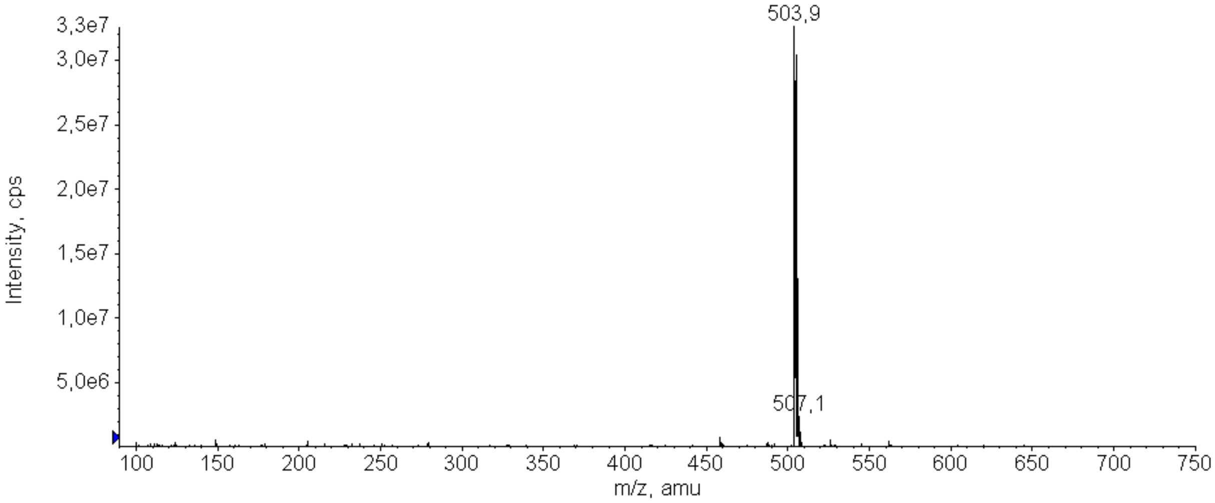

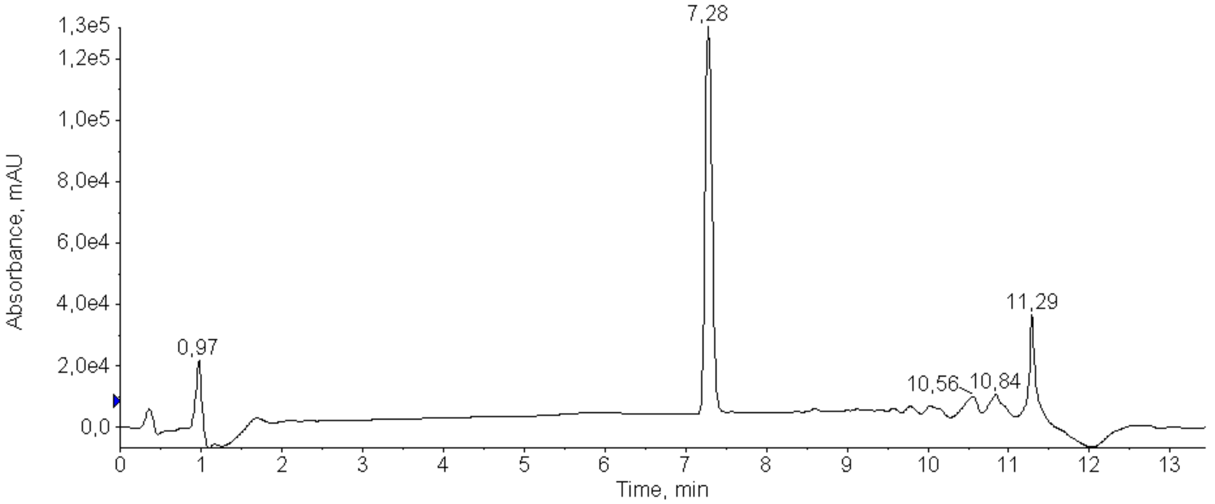

Supporting Information Figure 28: LC/MS analysis of **Scaff10-10**. Upper panel: Mass spectrum. Lower panel: according chromatogram.

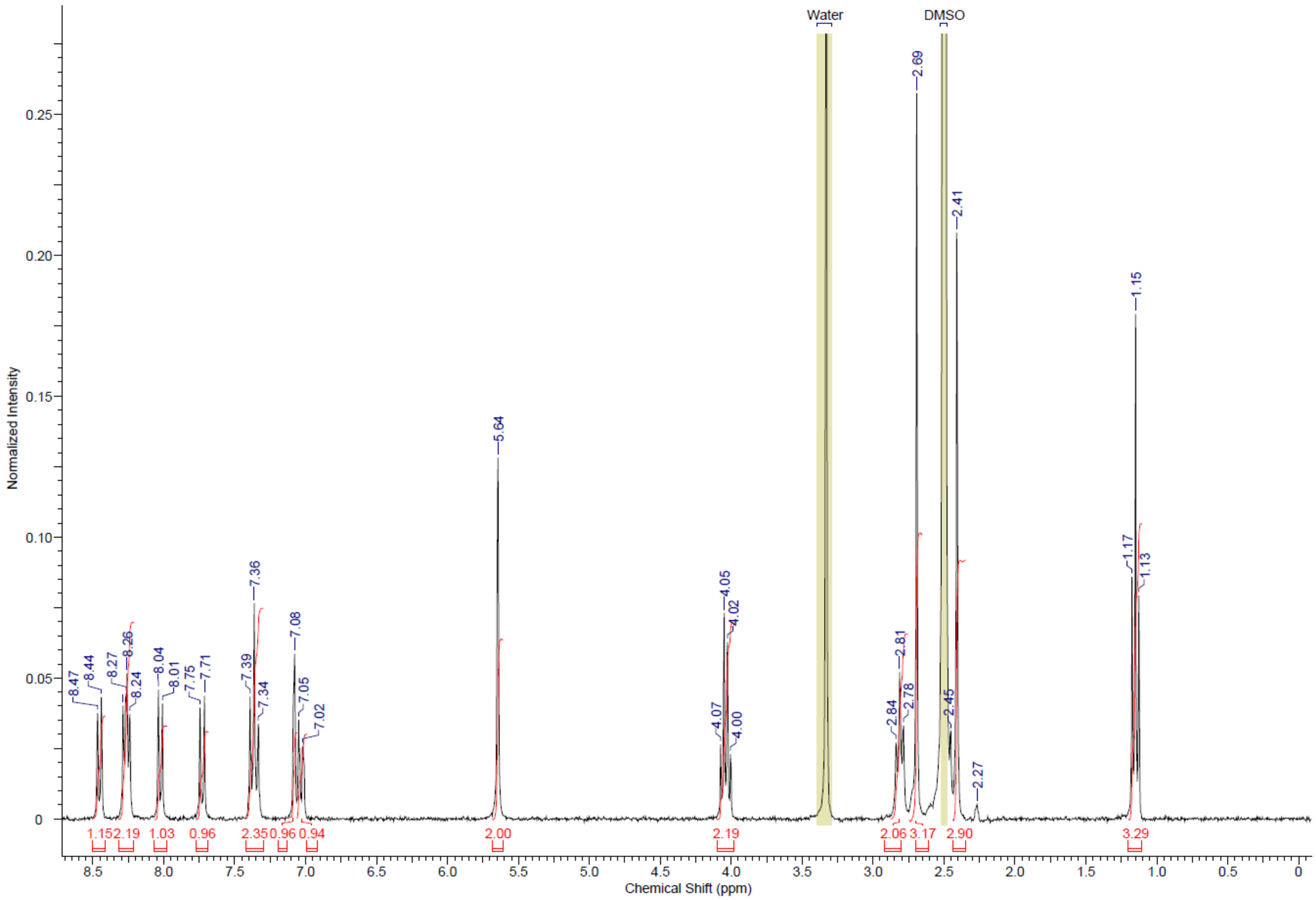

Supporting Information Figure 29: ^{1}H NMR spectrum of **Scaff10-10**, 300 MHz, DMSO-d_6.

4.1.16 3-[7-[2-[6-(4-Fluorophenyl)-2-methyl-3-pyridyl]-2-oxo-ethoxy]-4-methyl-2-oxo-chromen-3-yl]propanoic acid (**Scaff10-11**)

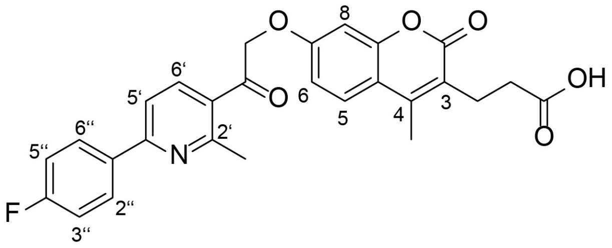

Ethyl 3-[7-[2-[6-(4-fluorophenyl)-2-methyl-3-pyridyl]-2-oxo-ethoxy]-4-methyl-2-oxo-chromen-3-yl]propanoate (**Scaff10-10**) (50 mg, 0.10 mmol) was saponificated in 2 ml 1 M sodium hydroxide at 55 °C for 3 h. Dropwise addition of 1 M H_2SO_4 resulted in precipitation of the product. The precipitated solid was filtered. The final product gave a yellowish solid.

Due to the pure yield, no ^{1}H NMR spectrum was obtained.

ESI-MS: cal. 476.15, exp. 476.1 ($[M+H]^+$)

HPLC: 98 % purity at 254 nm

t_R (HPLC): 6.62 min; λ_{max} = 310 nm

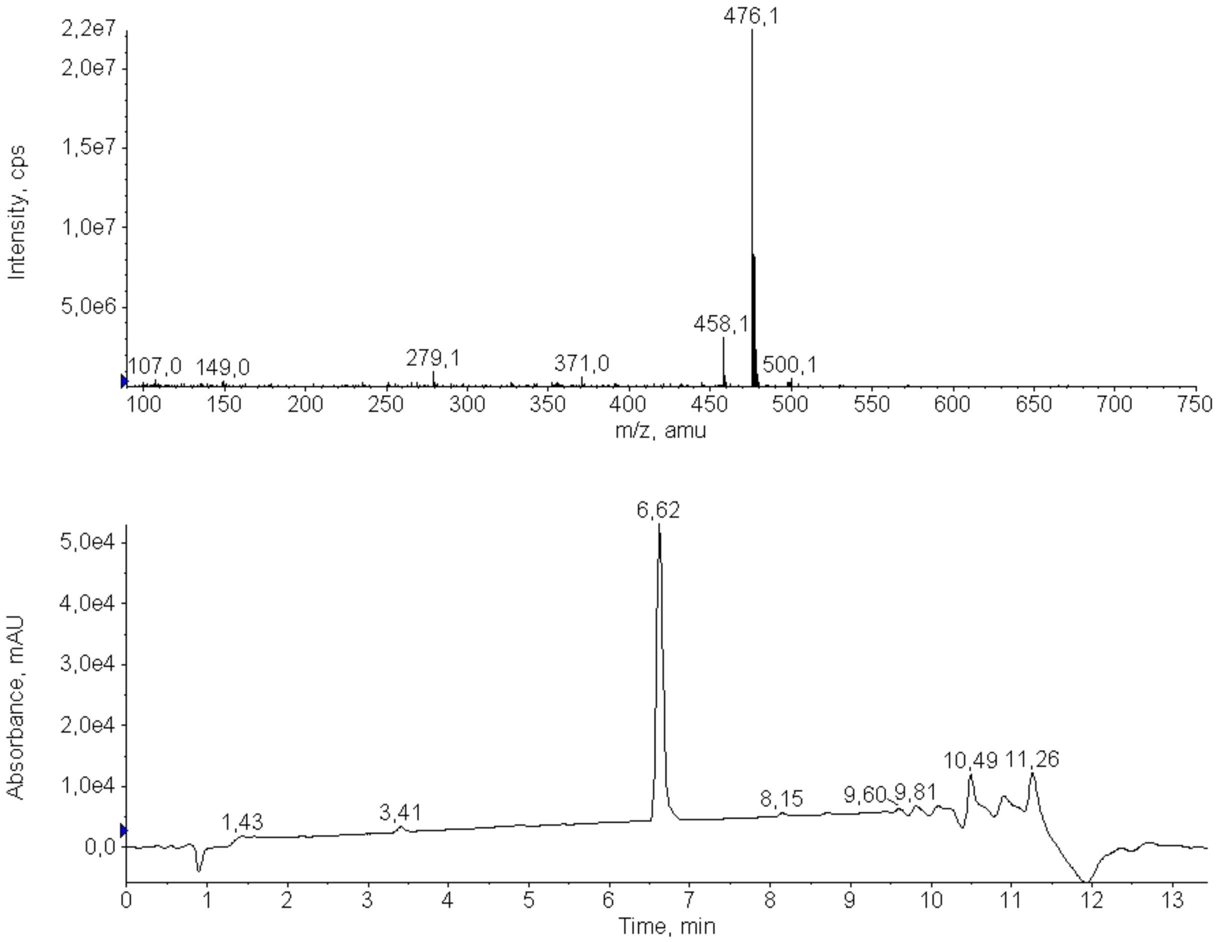

Supporting Information Figure 30: LC/MS analysis of **Scaff10-11**. Upper panel: Mass spectrum. Lower panel: according chromatogram.

4.1.17 3,4-Dimethyl-7-[2-(2-naphthyl)-2-oxo-ethoxy]chromen-2-one (**Scaff10-12**)

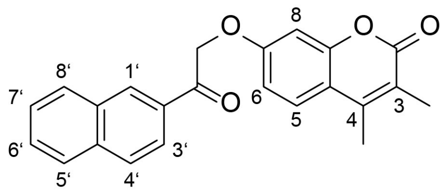

7-Hydroxy-3,4-dimethyl-chromen-2-one (**5b**) (100 mg, 0.50 mmol) was solved in 5 ml acetone and preactivated with potash in excess (182 mg, 1.31 mmol) at 55 °C. The α-haloketone 2-bromo-1-(2-naphthyl)ethanone (**2e**) was added (137 mg, 0.55 mmol). The mixture was refluxed at the same temperature. The final product after purification gave an ivory white solid in 77 % yield (145 mg).

^{1}H-NMR (300 MHz, DMSO-d_6): δ(ppm) = 2.02 – 2.12 (s, 3H, -C3-CH_3), 2.30 – 2.42 (s, 3H, -C4-CH_3), 5.79 – 5.91 (s, 2H, -CO-C**H_2**-OAr), 7.00 – 7.08 (dd, J = 2.44 Hz, 8.55 Hz, 1H, H-6), 7.08 – 7.12 (d, J = 2.44 Hz, 1H, H-8), 7.61 – 7.75 (m, 3H, H-5, -3', -6'), 7.98 – 8.13 (m, 4H, H-4', -5', -7', -1'), 8.76 – 8.80 (s, 1H, H-8')

ESI-MS: cal. 359.12, exp. 359.1 ([M+H]$^+$)

HPLC: 95 % purity at 254 nm

t_R (HPLC): 7.16 min; λ_{max} = 295 nm and 318 nm

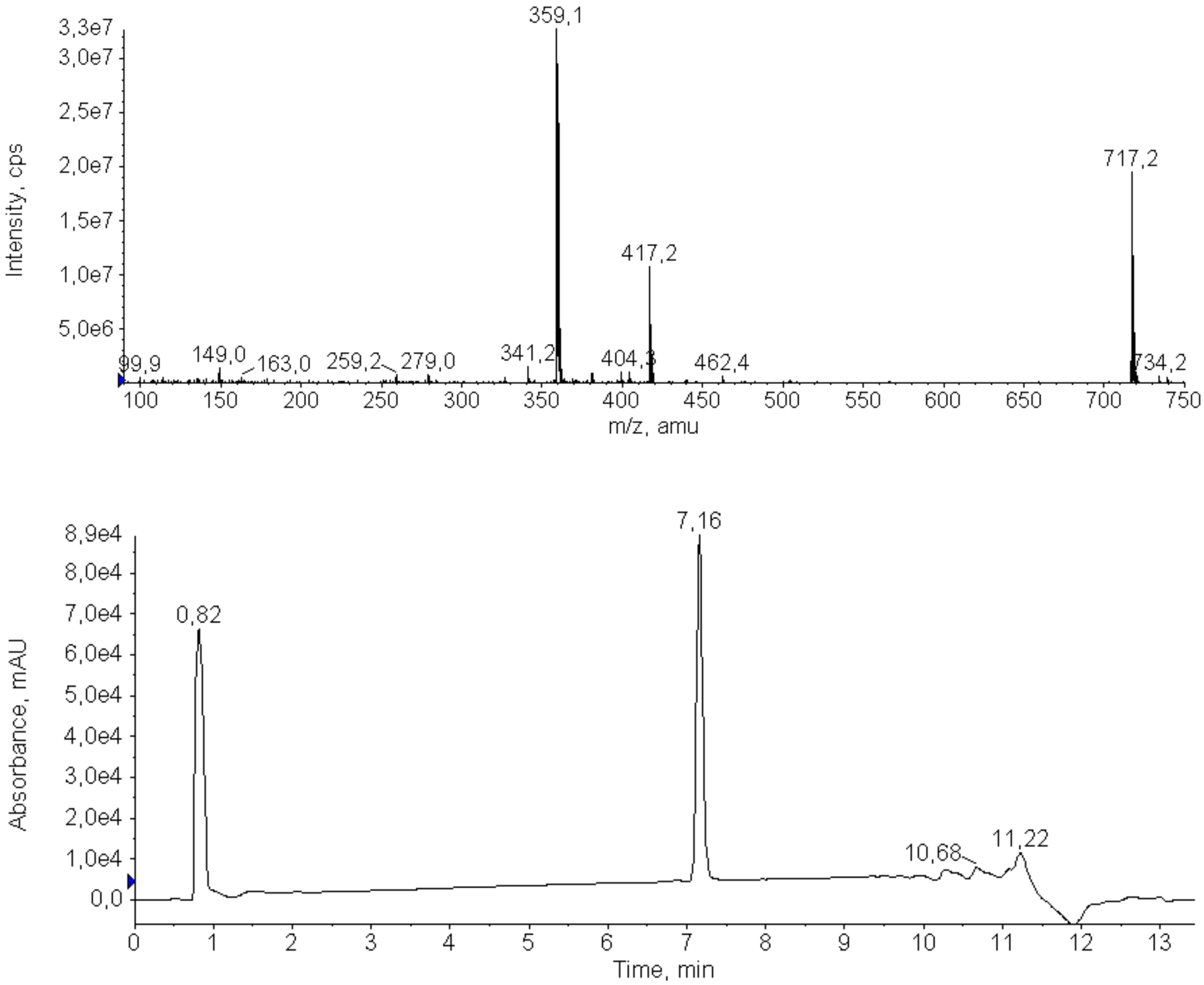

Supporting Information Figure 31: LC/MS analysis of **Scaff10-12**. Upper panel: Mass spectrum. Lower panel: according chromatogram.

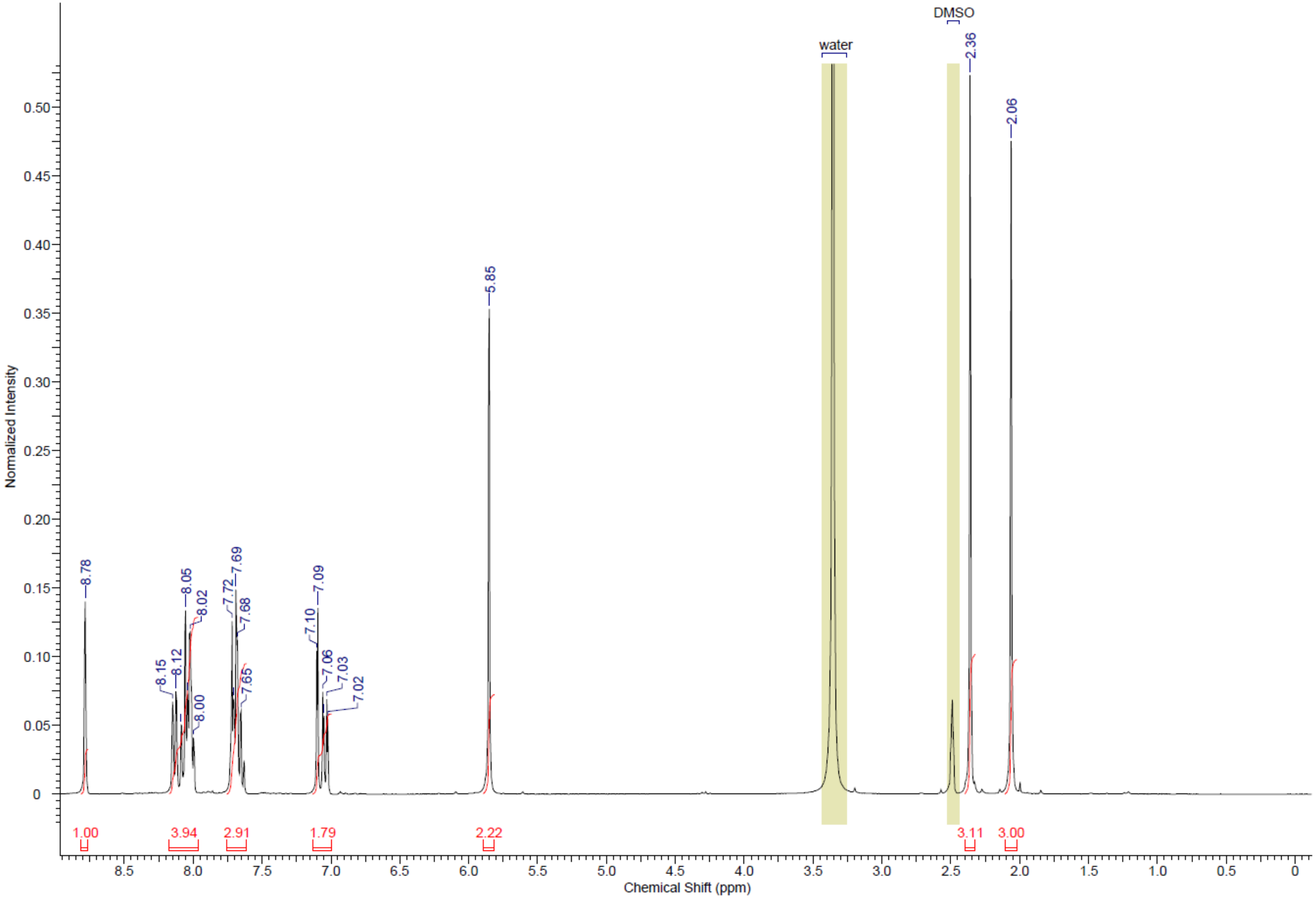

Supporting Information Figure 32: ^{1}H NMR spectrum of **Scaff10-12**, 300 MHz, DMSO-d_6.

4.1.18 3,4-Dimethyl-7-[2-oxo-2-(4-phenylphenyl)ethoxy]chromen-2-one (**Scaff10-13**)

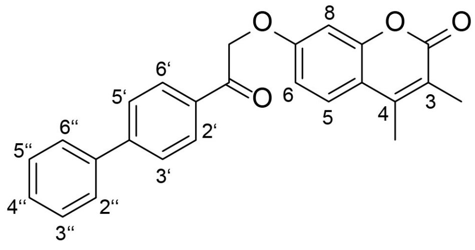

7-Hydroxy-3,4-dimethyl-chromen-2-one (**5b**) (100 mg, 0.50 mmol) was solved in 5 ml acetone and preactivated with potash in excess (182 mg, 1.31 mmol) at 55 °C. The α-haloketone 2-bromo-1-(4-phenylphenyl)ethanone (**2f**) was added (138 mg, 0.55 mmol). The mixture was refluxed at the same temperature. The final product after purification gave an orange-yellowish solid in 92 % yield (177 mg).

^{1}H NMR (300 MHz, DMSO-d_6): δ(ppm) = 2.06 – 2.14 (s, 3H, -C3-CH_3), 2.37 – 12.43 (s, 3H, -C4-CH_3), 5.76 – 5.81 (s, 2H, -CO-C**H_2**-OAr), 7.02 – 7.08 (dd, J = 2.44 Hz, 8.54 Hz, 1H, H-6), 7.09 – 7.11 (d, J = 2.44 Hz, 1H, H-8), 7.44 – 7.59 (m, 3H, H-3'', -4'', -5''), 7.71 – 7.76 (d, J = 8.54 Hz, 1H, H-5), 7.78 – 7.83 (d, J = 7.32 Hz, 2H, H-2'', -6''), 7.89 – 7.94 (d, J = 8.54 Hz, 2H, H-3', 5'), 8.12 – 8.18 (d, J = 8.55 Hz, 2H, H-2', 6')

ESI-MS: cal. 385.14, exp. 385.1 ($[M+H]^+$)

HPLC: 98 % purity at 254 nm

t_R (HPLC): 7.55 min; λ_{max} = 293 nm and 310 nm

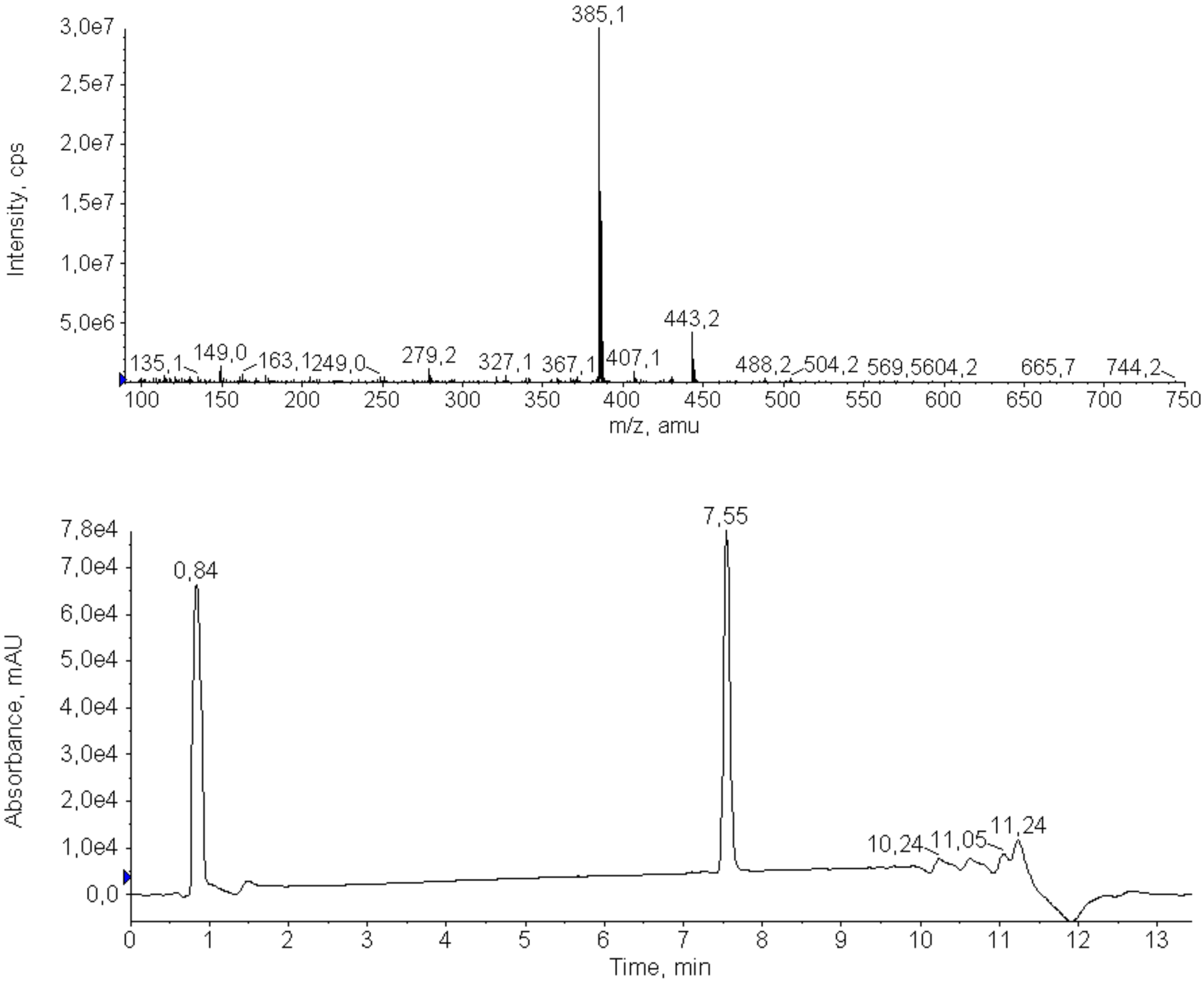

Supporting Information Figure 33: LC/MS analysis of **Scaff10-13**. Upper panel: Mass spectrum. Lower panel: according chromatogram.

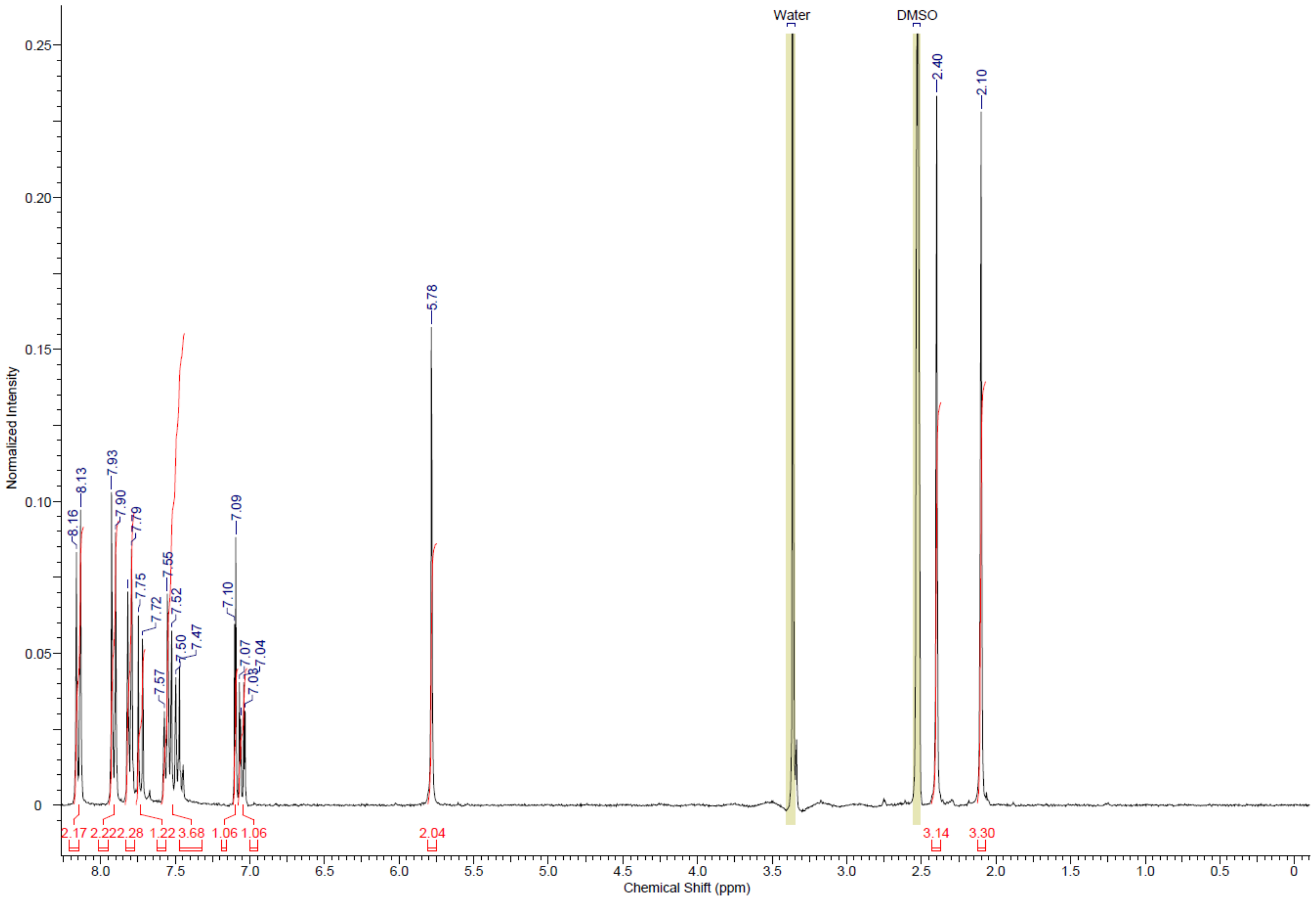

Supporting Information Figure 34: ^{1}H NMR spectrum of **Scaff10-13**, 300 MHz, DMSO-d_6.

4.1.19 3,4-Dimethyl-7-[2-oxo-2-(4-phenoxyphenyl)ethoxy]chromen-2-one (**Scaff10-14**)

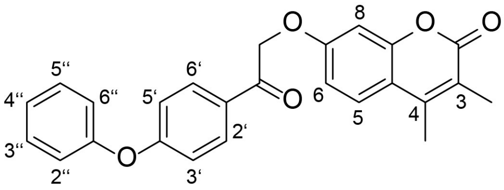

7-Hydroxy-3,4-dimethyl-chromen-2-one (**5b**) (50 mg, 0.26 mmol) was solved in 3 ml acetone and preactivated with potash in excess (106 mg, 0.76 mmol) at 55 °C. The α-haloketone 2-bromo-1-(4-phenoxyphenyl)ethanone (**2g**) was added (96 mg, 0.33 mmol). The mixture was refluxed at the same temperature for 16 h. The final product after purification gave a white solid in 97 % yield (101 mg).

^{1}H NMR (300 MHz, DMSO-d_6): δ(ppm) = 2.01 – 2.13 (s, 3H, -C3-CH_3), 2.30 – 2.42 (s, 3H, -C4-CH_3), 5.61 – 5.72 (s, 2H, -CO-C**H_2**-OAr), 6.95 – 7.04 (m, 2H, H-6, -8), 7.61 – 7.75 (m, 4H, H-3', -5', -2'', -6''), 7.22 – 7.30 (t, J = 7.33 Hz, 1H, H-4''), 7.43 – 7.51 (t, J = 7.33 Hz, 2H, H-3'', -5''), 7.66 – 7.72 (d, J = 8.55 Hz, 1H, H-5), 8.02 – 8.09 (d, J = 9.77 Hz, 2H, H-2', -6')

ESI-MS: cal. 401.13, exp. 401.1 ($[M+H]^+$)

HPLC: 95 % purity at 254 nm

t_R (HPLC): 7.63 min; λ_{max} = 277 nm and 315 nm

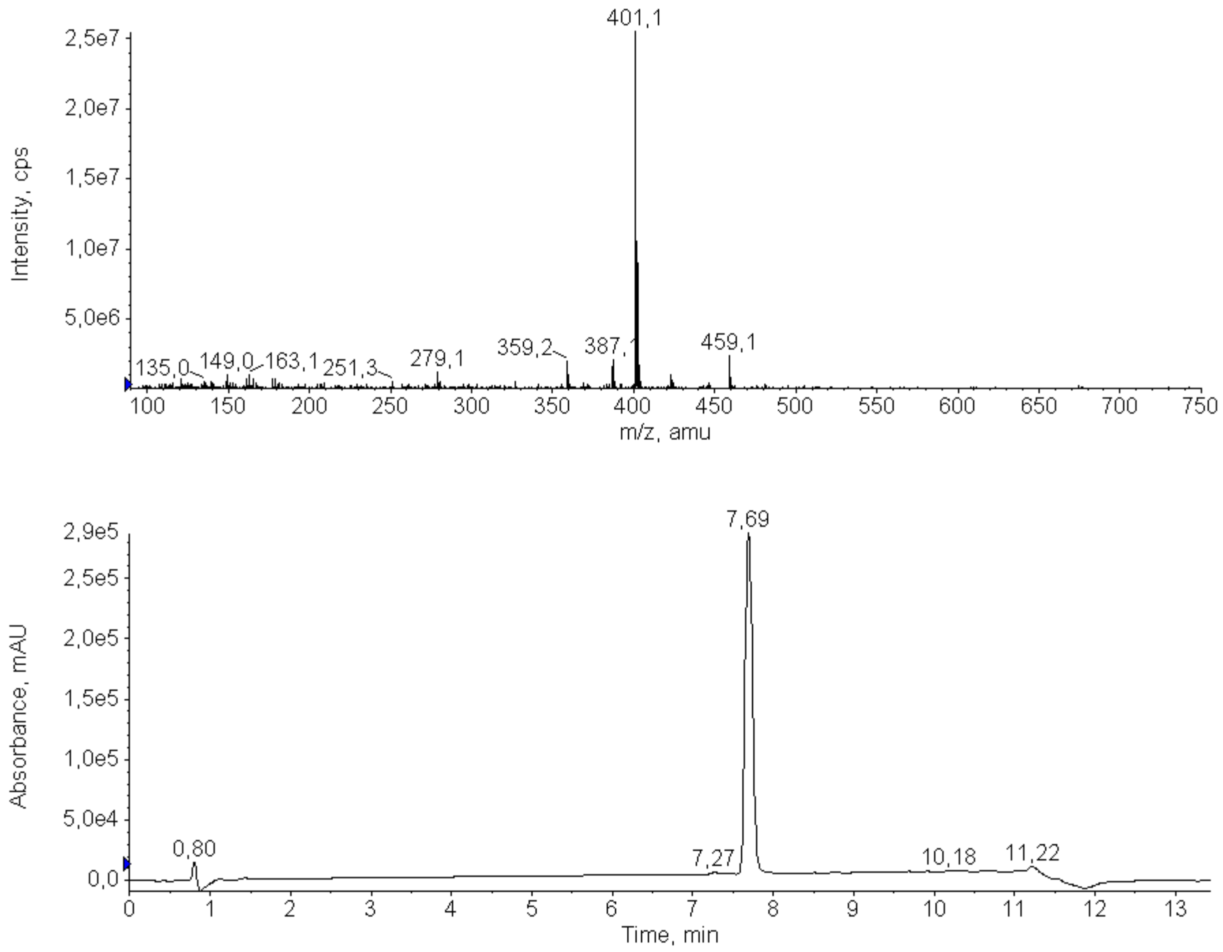

Supporting Information Figure 35: LC/MS analysis of **Scaff10-14**. Upper panel: Mass spectrum. Lower panel: according chromatogram.

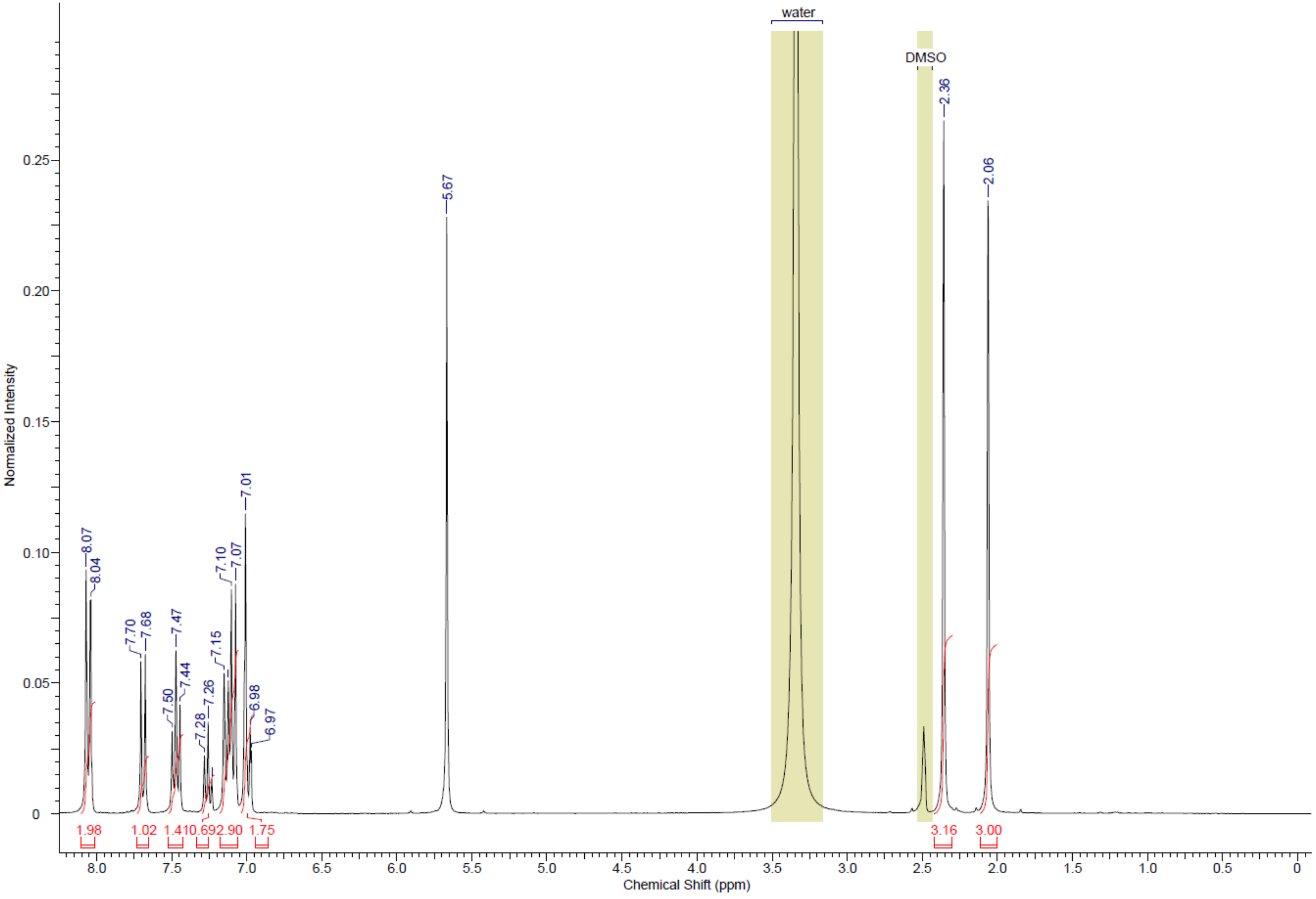

Supporting Information Figure 36: ^{1}H NMR spectrum of **Scaff10-14**, 300 MHz, DMSO-d_6.

4.1.20 7-[2-(6-Methoxy-2-naphthyl)-2-oxo-ethoxy]-3,4-dimethyl-chromen-2-one (**Scaff10-15**)

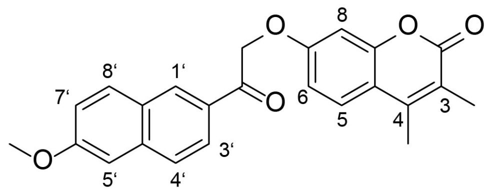

7-Hydroxy-3,4-dimethyl-chromen-2-one (**5b**) (50 mg, 0.26 mmol) was solved in 5 ml acetone and preactivated with potash in excess (106 mg, 0.76 mmol) at 55 °C. The α-haloketone 2-bromo-1-(6-methoxy-2-naphthyl)ethanone (**2h**) was added (92 mg, 0.33 mmol). The mixture was refluxed at the same temperature. The final product after purification gave a white solid.

^{1}H NMR (300 MHz, DMSO-d_6): δ(ppm) = 2.06 – 2.13 (s, 3H, -C3-CH_3), 2.37 – 2.42 (s, 3H, -C4-CH_3), 3.92 – 3.99 (s, 3H, -OCH_3), 5.81 – 5.86 (s, 2H, -CO-C**H_2**-OAr), 7.03 – 7.09 (dd, J= 2.44 Hz, 8.54 Hz, 1H, H-6), 7.09 – 7.15 (d, J = 2.44 Hz, 1H, H-8), 7.29 – 7.35 (dd, J = 2.44 Hz, 8.45 Hz, 1H, H-7'), 7.45 – 7.48 (d, J = 2.44 Hz, 1H, H-5'), 7.70 – 7.76 (d, J = .54 Hz, 1H, H-5), 7.94 – 8.09 (m, 3H, H-3', -4', -8'), 8.70 – 8.74 (s, 1H, H-1')

ESI-MS: cal. 389.13, exp. 389.0 ($[M+H]^+$)

HPLC: 95 % purity at 254 nm

t_R (HPLC): 7.13 min; λ_{max} = 273 nm and 322 nm

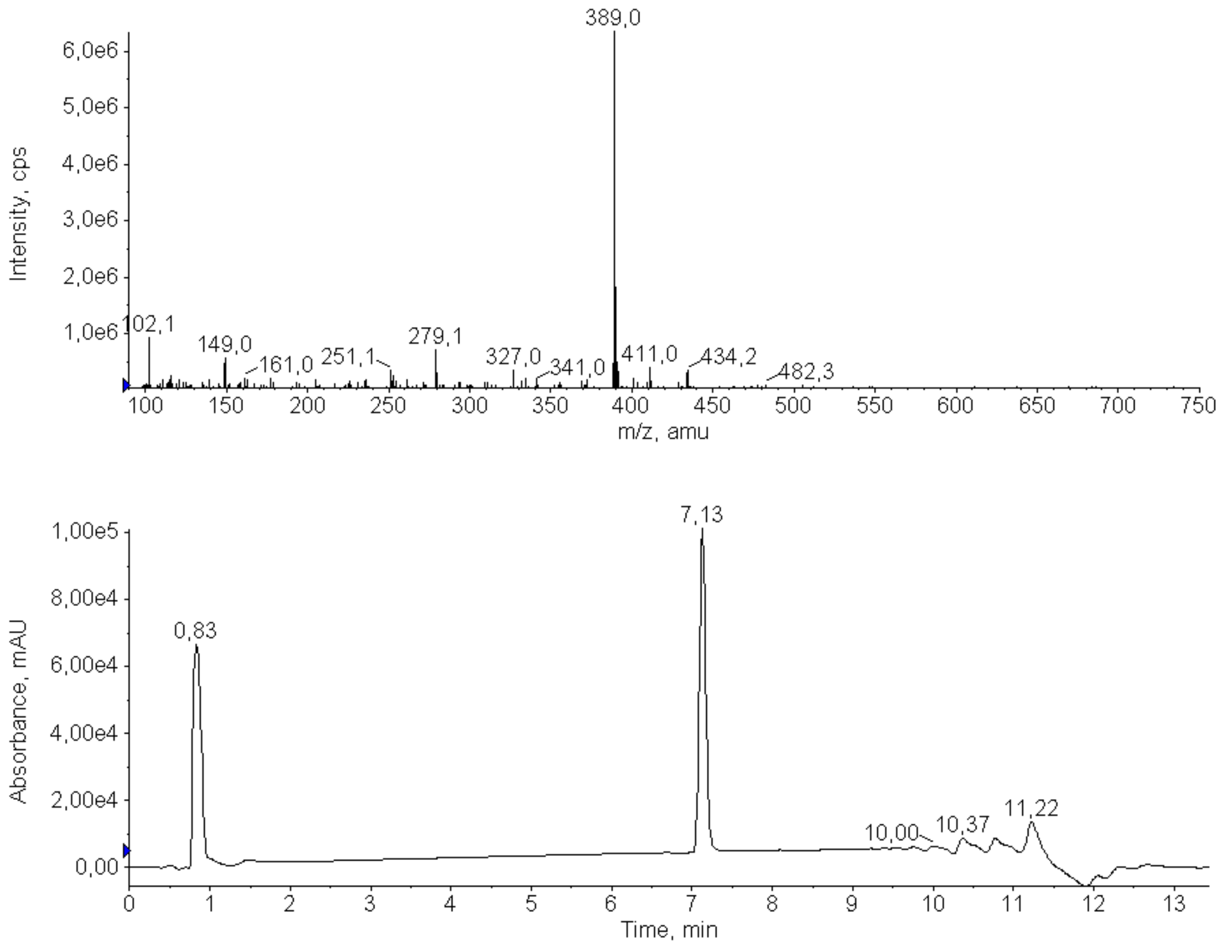

Supporting Information Figure :. LC/MS analysis of **Scaff10-15**. Upper panel: Mass spectrum. Lower panel: according chromatogram.

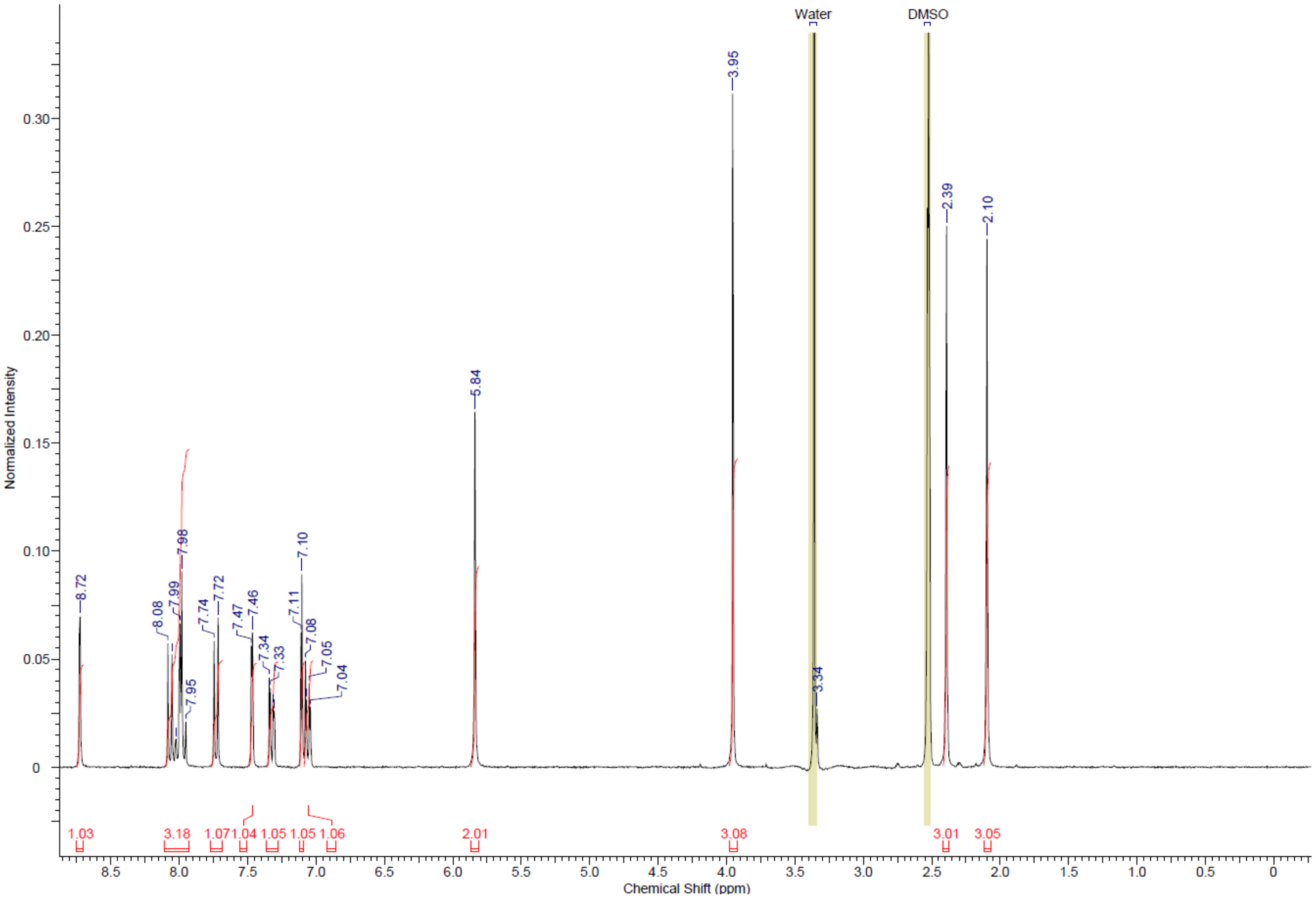

Supporting Information Figure 37: ^{1}H NMR spectrum of **Scaff10-15**, 300 MHz, DMSO-d$_6$.

4.1.21 Ethyl 3-[4-methyl-2-oxo-7-[2-oxo-2-(4-phenoxyphenyl)ethoxy]chromen-3-yl]propanoate (**Scaff10-17**)

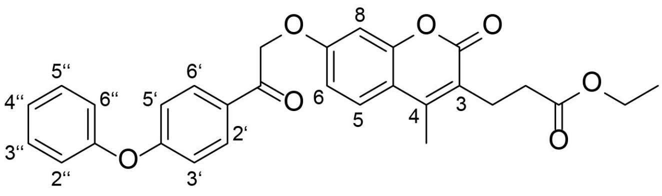

Ethyl 3-(7-hydroxy-4-methyl-2-oxo-chromen-3-yl)propanoate (**5a**) (100 mg, 0.36 mmol) was solved in 6 ml dry acetone and preactivated with potash in excess (125 mg, 0.90 mmol) at 55 °C. The α-haloketone 2-bromo-1-(4-phenoxyphenyl)ethanone (**2g**) was added (115 mg, 0.39 mmol). The mixture was refluxed at the same temperature for 16 h. The product was precipitated using 1 M H_2SO_4 and recrystallised in ethanol. The final product gave a dark brown solid.

^{1}H NMR (300 MHz, DMSO-d_6): δ(ppm) = 1.09 – 1.23 (t, J = 7.32 Hz, 3H, -CH_2-C**H_3**), 2.34 – 2.43 (s, 3H, Ar-CH_3), 2.43 – 2.51 (t, J = 8.54 Hz, 2H, -CH_2-C**H_2**-COOEt), 2.75 – 2.87 (t, J = 8.54 Hz, 2H, -C**H_2**-CH_2-COOEt), 3.97 – 4.10 (q, J = 7.32 Hz, 2H, -C**H_2**-CH_3), 5.64 – 5.73 (s, 2H, -CO-C**H_2**-OAr), 6.98 – 7.01 (d, J = 2.44 Hz, 1H, H6), 7.01 – 7.06 (s, 1H, H-8), 7.06 – 7.12 (d, J = 9.77 Hz, 2H, H-3'', -5''), 7.12 – 7.19 (d, J = 8.54 Hz, 2H, H-2'', -6''), 7.22 – 7.31 (t, J = 7.32 Hz, 1H, H-4''), 7.43 – 7.52 (t, J = 8.54 Hz , 2H, H-3', -5'), 7.68 – 7.75 (d, J = 8.54 Hz, 1H, H-5), 8.02 – 8.10 (d, J = 8.55 Hz, 2H, H-2', -6')

ESI-MS: cal. 487.17, exp. 487.1 ($[M+H]^+$)

HPLC: 98 % purity at 254 nm

t_R (HPLC): 7.40 min; λ_{max} = 279 nm and 317 nm

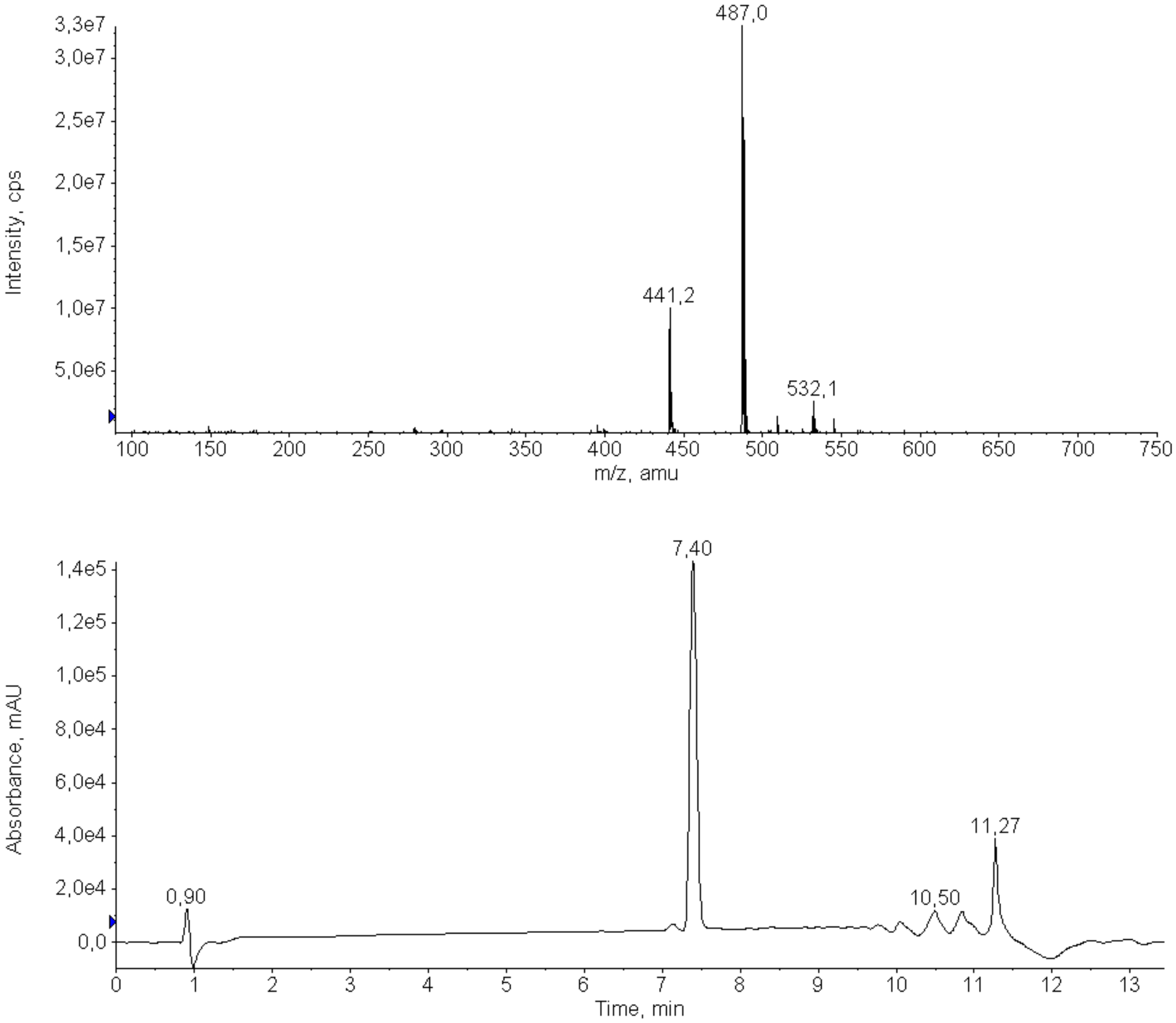

Supporting Information Figure 38: LC/MS analysis of **Scaff10-17**. Upper panel: Mass spectrum. Lower panel: according chromatogram.

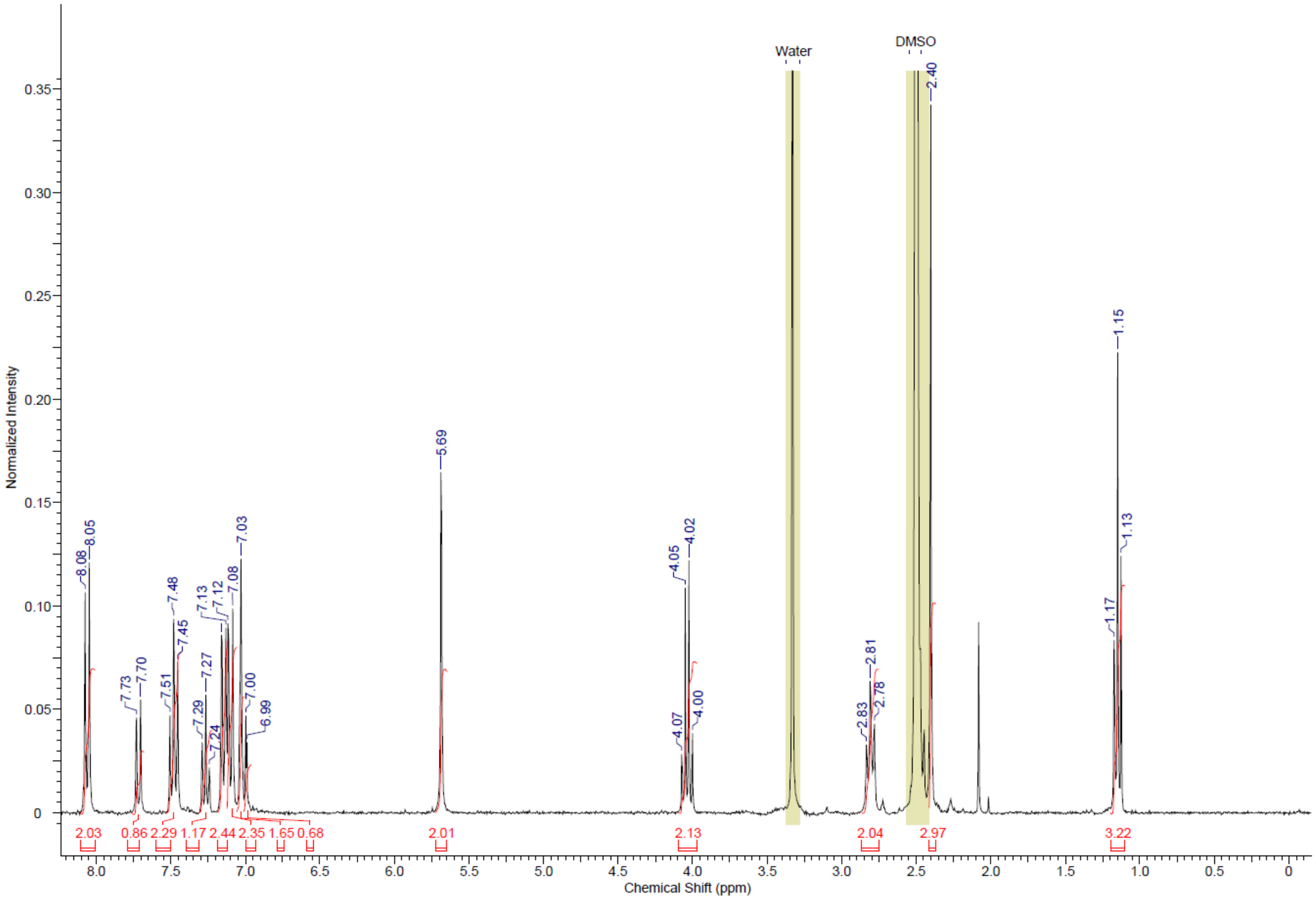

Supporting Information Figure 39: ^{1}H NMR spectrum of **Scaff10-17**, 300 MHz, DMSO-d_6.

4.1.22 3-[5-Methyl-3-(2-naphthyl)-7-oxo-furo[3,2-*g*]chromen-6-yl]propanoic acid (**Scaff10-25**)

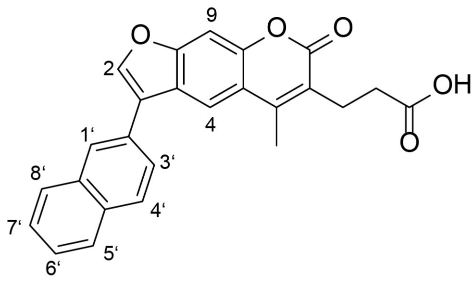

Ethyl 3-(7-hydroxy-4-methyl-2-oxo-chromen-3-yl)propanoate (**5a**) (50 mg, 0.18 mmol) was solved in 6 ml dry acetone and preactivated with potash in excess (249 mg, 1.80 mmol) at 55 °C. The α-haloketone 2-bromo-1-(2-naphthyl)ethanone (**2e**) was added (164 mg, 0.72 mmol). The mixture was refluxed at the same temperature for 2 h. The product was precipitated using 1 M H_2SO_4. The precipitated solid was filtered and purified using preparative HPLC with the following gradient: 30 % to 95 % ACN in H_2O in 60 min. The product eluted at t_R = 15 min and was obtained after lyophilisation as yellowish solid in 22 % yield (3 mg).

Due to the pure yield, no ^{1}H NMR spectrum was obtained.

ESI-MS: cal. 399.12, exp. 399.04 ($[M+H]^+$)

HPLC: 95 % purity at 254 nm

t_R (HPLC): 8.9 min; λ_{max} = 257 nm and 308 nm

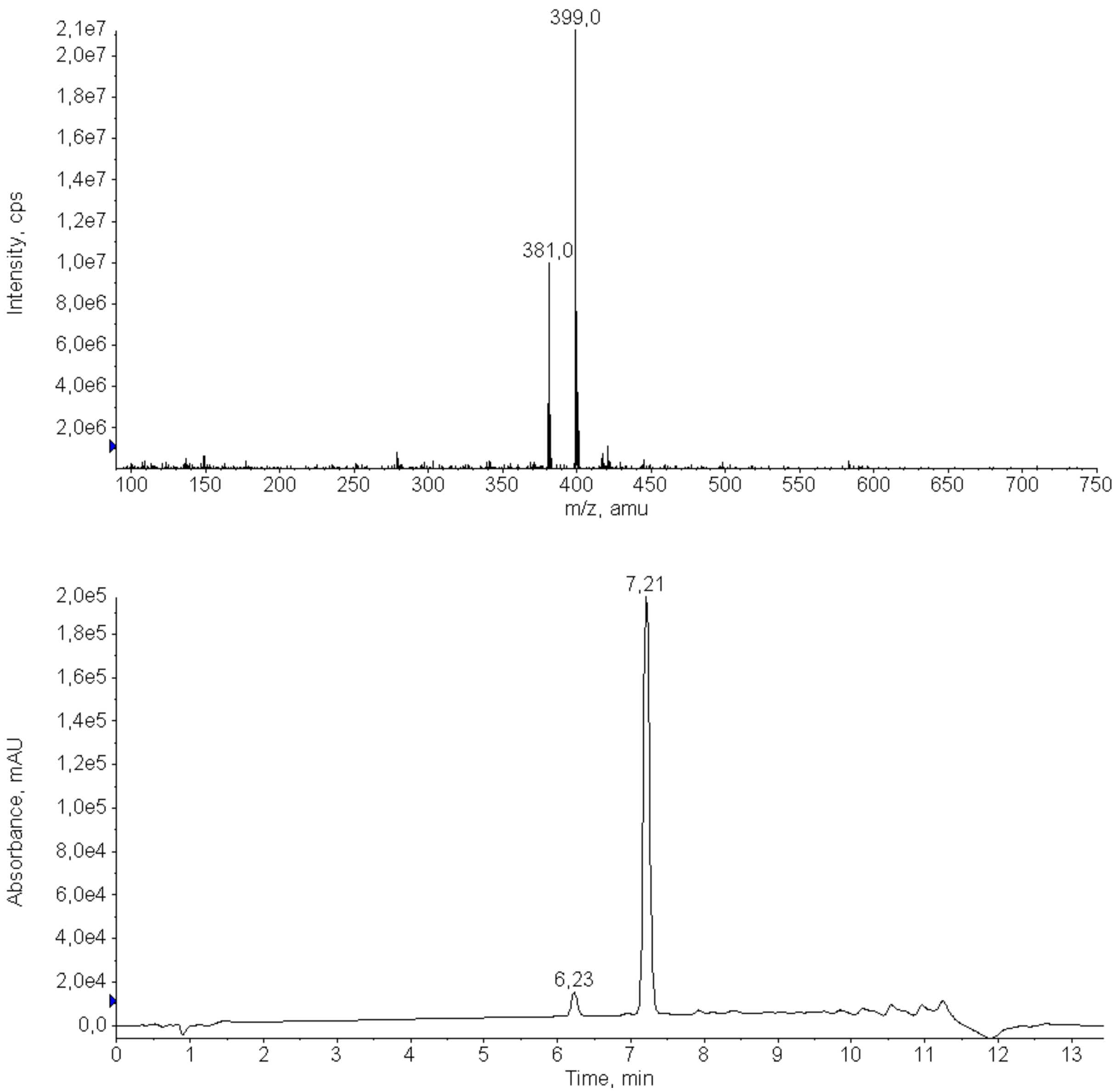

Supporting Information Figure 40: LC/MS analysis of **Scaff10-25**. Upper panel: Mass spectrum. Lower panel: according chromatogram.

4.1.23 3-[3-(4-Methoxyphenyl)-5-methyl-7-oxo-furo[3,2-*g*]chromen-6-yl]-*N*,*N*-dimethyl-propanamide (**Scaff10-26**)

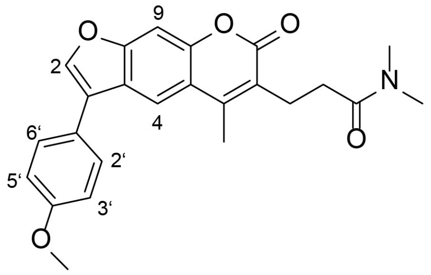

3-[3-(4-Methoxyphenyl)-5-methyl-7-oxo-furo[3,2-*g*]chromen-6-yl]propanoic acid (**Scaff10-8**) (11 mg, 0.03 mmol) was condensed with dimethylamine hydrochloride in excess (7.11 mg, 0.09 mmol) catalysed by HATU (16.6 mg, 0.04 mmol) in 1 ml DMF and triethylamine as base (8.83 mg, 0.09 mmol) at 25 °C for 3.5 h. The product was isolated using preparative HPLC with the following gradient: 5 % to 80 % ACN in H_2O in 90 min. The product eluted at t_R = 65 min and was obtained after lyophilisation as yellow solid in 30 % yield (3.5 mg).

^{1}H NMR (300 MHz, DMSO-d_6): δ(ppm) = 2.39 - 2.46 (m, 2H, -CH_2-C**H_2**-CON$(CH_3)_2$), 2.53 – 2.58 (s, 3H, Ar-CH_3), 2.76 – 2.86 (m, 5H, -N-(C**H_3**)$_2$, -C**H_2**-CH_2-CON$(CH_3)_2$), 2.91 – 2.99 (s, 3H, -N-(C**H_3**)$_2$), 3.78 – 3.88 (s, 3H, -OCH_3), 7.06 – 7.16 (d, J = 8.55 Hz, 2H, H-3', -5'), 7.70 – 7.79 (d, J = 8.54 Hz, 2H, H-2', -6'), 8.10 – 8.17 (s, 1H, H-4), 8.35 – 8.41 (s, 1H, H-2)

ESI-MS: cal. 406.16, exp. 406.08 ($[M+H]^+$)

HPLC: 99 % purity at 254 nm

t_R (HPLC): 6.45 min; λ_{max} = 253 nm and 308 nm

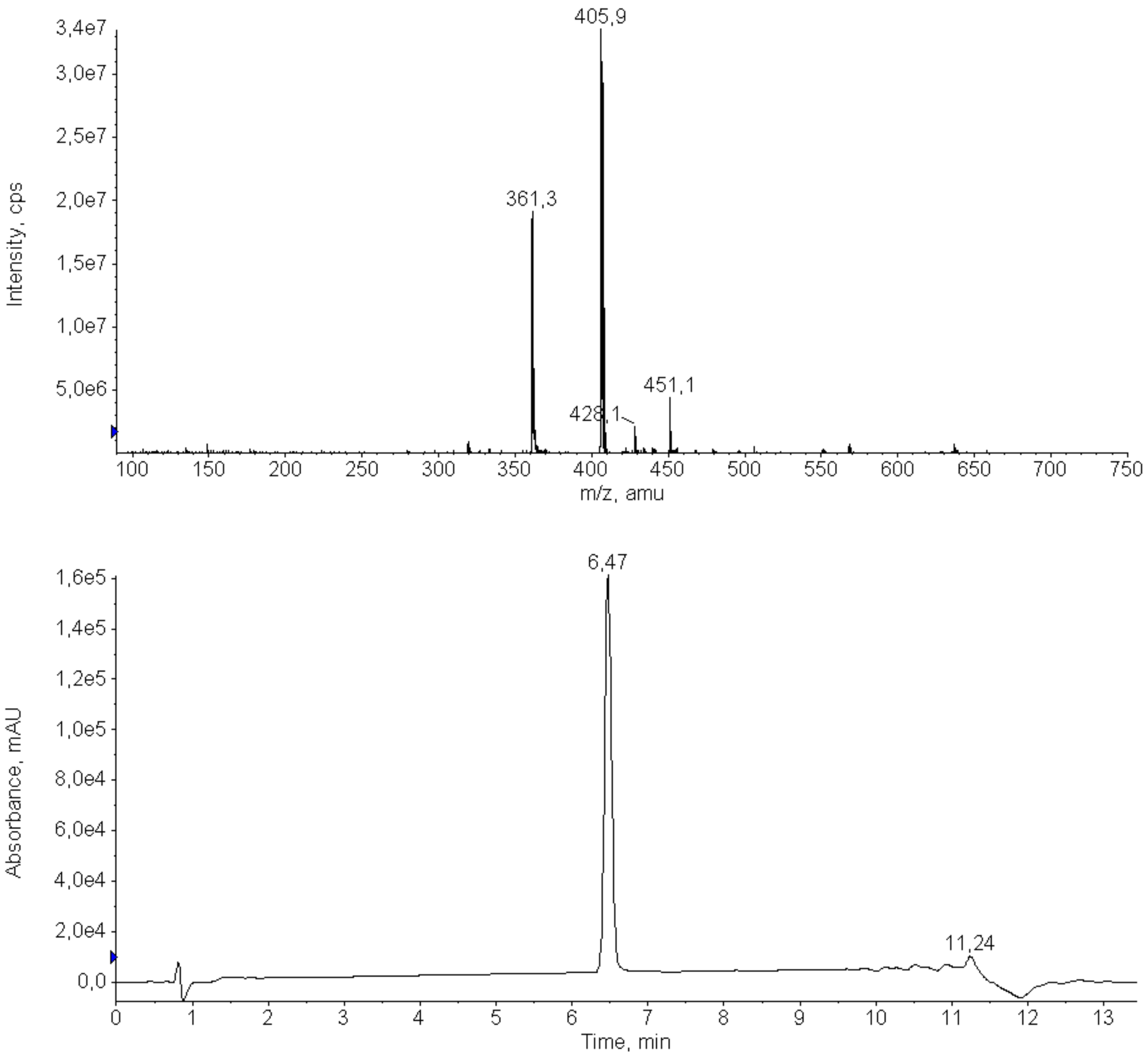

Supporting Information Figure 41: LC/MS analysis of **Scaff10-26**. Upper panel: Mass spectrum. Lower panel: according chromatogram.

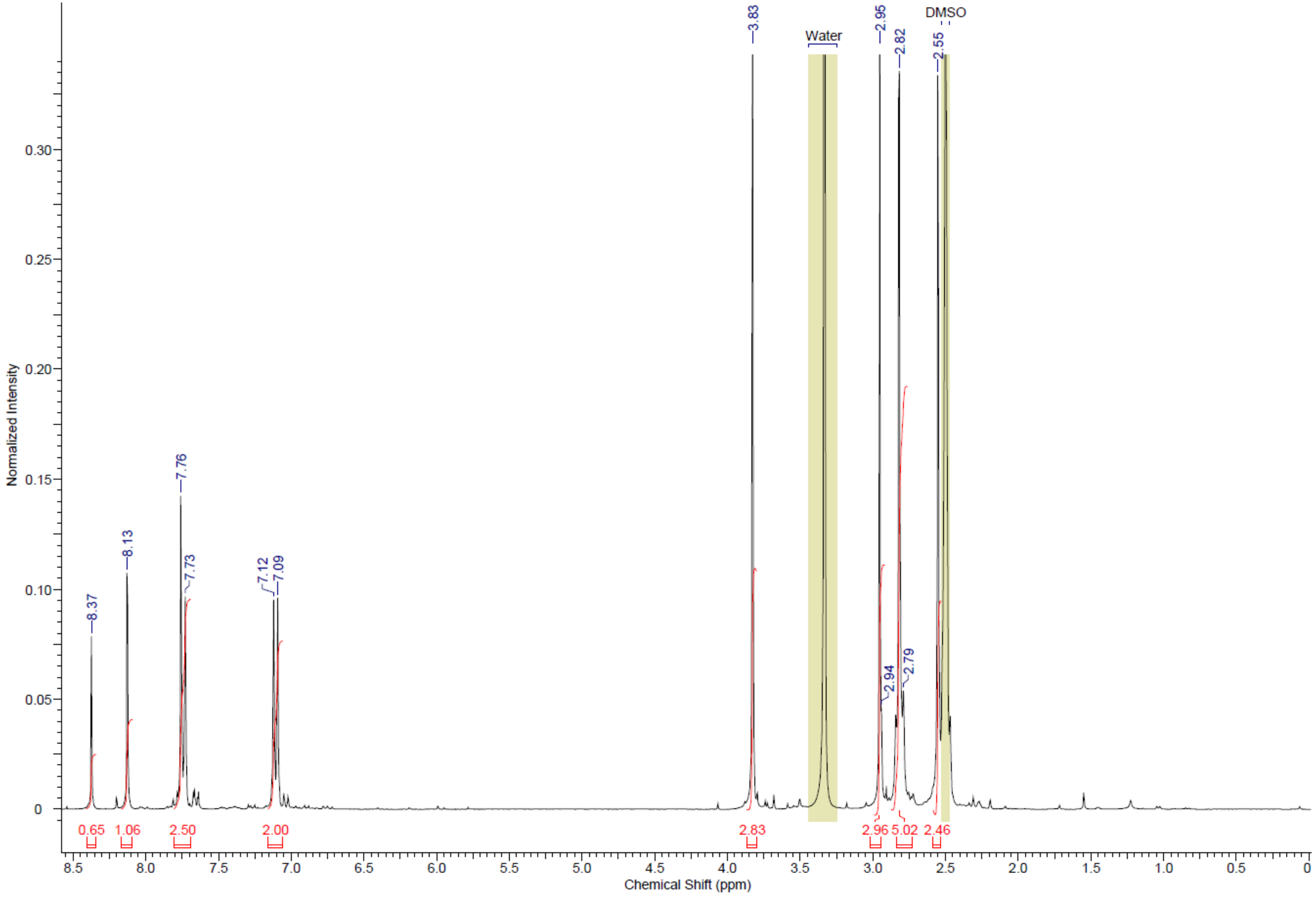

Supporting Information Figure 42: ^{1}H NMR spectrum of **Scaff10-26**, 300 MHz, DMSO-d_6.

4.1.24 3-[3-(4-Methoxyphenyl)-5-methyl-7-oxo-furo[3,2-*g*]chromen-6-yl]-*N*-(3-pyridyl)propanamide (**Scaff10-27**)

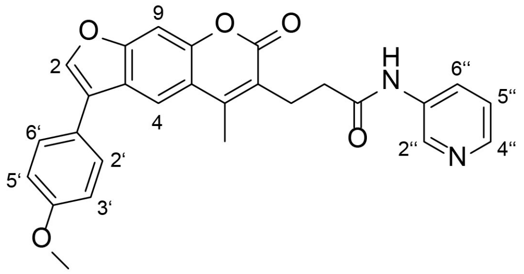

3-[3-(4-Methoxyphenyl)-5-methyl-7-oxo-furo[3,2-*g*]chromen-6-yl]propanoic acid (**Scaff10-8**) (11 mg, 0.03 mmol) was condensed with 3-aminopyridine in excess (4.1 mg, 0.044 mmol) catalysed by HATU (16.6 mg, 0.04 mmol) in 1 ml DMF and triethylamine as base (8.83 mg, 0.09 mmol) at 25 °C for 3.5 h. The product was isolated using preparative HPLC with the following gradient: 5 % to 80 % ACN in H_2O in 90 min. The product eluted at t_R = 67 min and was obtained after lyophilisation as light yellow solid in 48 % yield (6.4 mg).

^{1}H NMR (300 MHz, DMSO-d_6): δ(ppm) = 2.40 – 2.47 (s, 3H, Ar-CH_3), 2.51 – 2.63 (t, J = 7.32 Hz, 2H, -CH_2-C**H_2**-CONH-), 2.81 – 2.97 (t, J = 7.32 Hz, 2H, -C**H_2**-CH_2-CONH-), 3.74 – 3.85 (s, 3H, -OCH_3), 7.00 – 7.10 (d, J = 8.55 Hz, 2H, H-3', -5'), 7.20 – 7.32 (m, 1H, H-5''), 7.63 – 7.69 (m, 2H, H-2', -6'), 8.02 – 8.08 (s, 1H, H-4), 8.14 – 8.20 (m, 1H, H-6''), 8.29 – 8.34 (s, 1H, H-2), 8.61 – 8.64 (m, 1H, H-4''), 10.10 – 10.17 (s, 1H, NH)

ESI-MS: cal. 455.16, exp. 455.04 ($[M+H]^+$)

HPLC: 99 % purity at 254 nm

t_R (HPLC): 5.11 min; λ_{max} = 249 nm and 308 nm

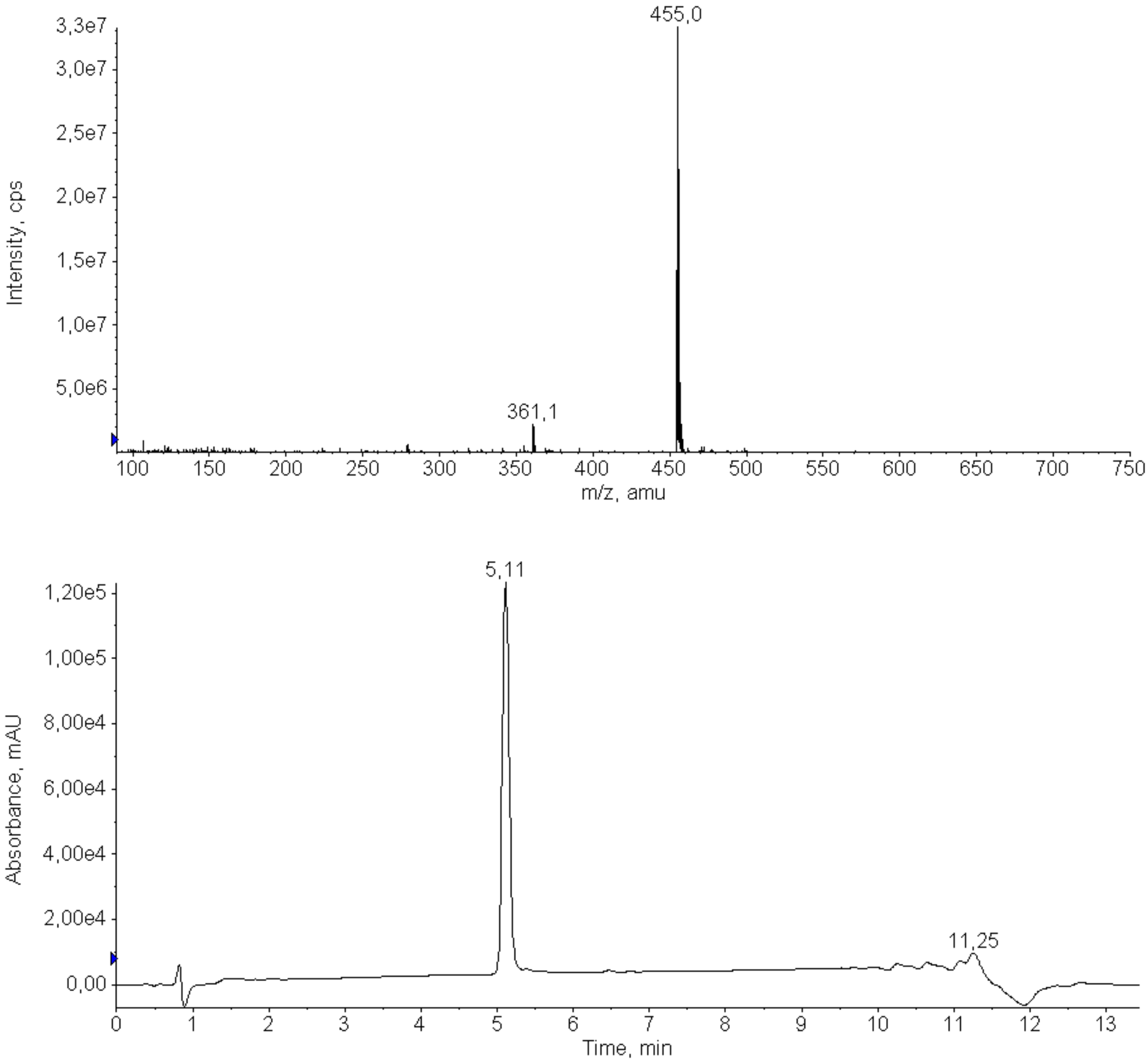

Supporting Information Figure 43: LC/MS analysis of **Scaff10-27**. Upper panel: Mass spectrum. Lower panel: according chromatogram.

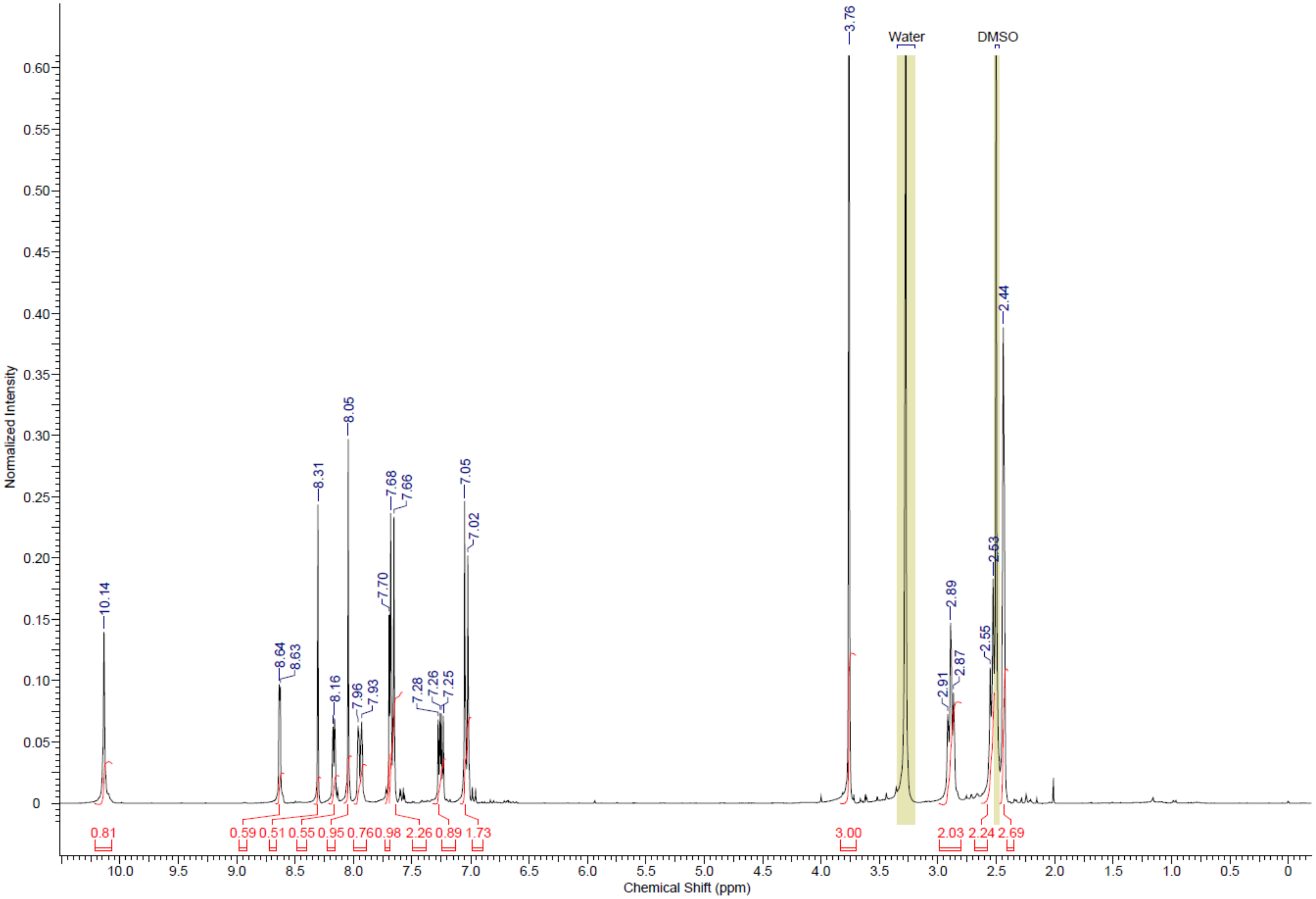

Supporting Information Figure 44: ^{1}H NMR spectrum of **Scaff10-27**, 300 MHz, DMSO-d_6.

4.1.25 Ethyl 3-[4-methyl-2-oxo-7-[2-oxo-2-[4-(trifluoromethyl)phenyl]ethoxy]chromen-3-yl]propanoate (**Scaff10-28**)

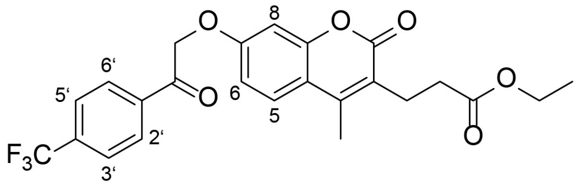

Ethyl 3-(7-hydroxy-4-methyl-2-oxo-chromen-3-yl)propanoate (**5a**) (200 mg, 0.72 mmol) was solved in 15 ml dry acetone and preactivated with potash in excess (249 mg, 1.80 mmol) at 55 °C. The α-haloketone 2-bromo-1-(4-trifluoromethylphenyl)ethanone (**2i**) was added (164 mg, 0.72 mmol). The mixture was refluxed at the same temperature for 2 h. Ethyl 3-(7-(2-(4-methoxyphenyl)-2-oxoethoxy)-4-methyl-2-oxo-chromen-3-yl)propanoate (**Scaff10-28**) was precipitated using 1 M H_2SO_4 and purified using preparative HPLC with the following gradient: 40 % to 95 % ACN in H_2O in 80 min. The product eluted at t_R = 48 min and was obtained after lyophilisation as ivory white solid in 5 % yield (16 mg).

^{1}H NMR (300 MHz, DMSO-d_6): δ(ppm) = 1.09 – 1.22 (t, J = 7.32 Hz, 3H, -CH_2-C**H₃**), 2.38 – 2.43 (s, 3H, Ar-CH_3), 2.44 – 2.49 (t, J = 8.54 Hz, 2H, -CH_2-C**H₂**-COOEt), 2.76 – 2.86 (t, J = 8.54 Hz, 2H, -C**H₂**-CH_2-COOEt), 3.98 – 4.09 (q, J = 7.32 Hz, 2H, -C**H₂**-CH_3), 5.76 – 5.82 (s, 2H, -CO-C**H₂**-OAr), 7.00 – 7.08 (d, J = 8.54 Hz, 1H, H-6), 7.10 – 7.13 (s, 1H, H-8), 7.69 – 7.76 (d, J = 8.55 Hz, 1H, H-5), 7.93 – 8.01 (d, J = 7.32 Hz , 2H, H-2', -6'), 8.19 – 8.26 (d, J = 8.54 Hz, 2H, H-3', -5')

ESI-MS: cal. 463.13, exp. 463.1 ($[M+H]^+$)

HPLC: 97 % purity at 254 nm

t_R (HPLC): 7.48 min; $λ_{max}$ = 290 nm and 319 nm

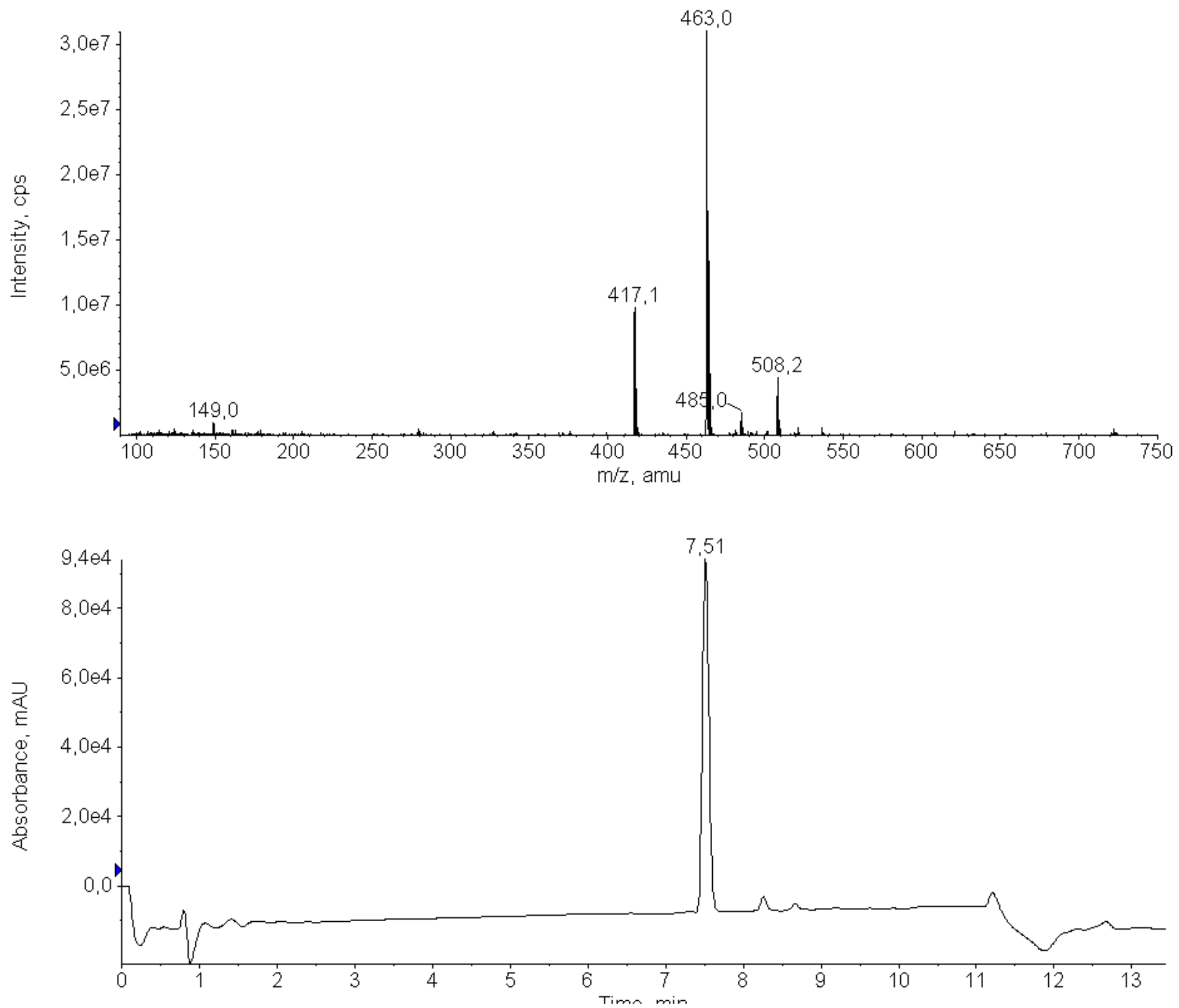

Supporting Information Figure 45: LC/MS analysis of **Scaff10-28**. Upper panel: Mass spectrum. Lower panel: according chromatogram.

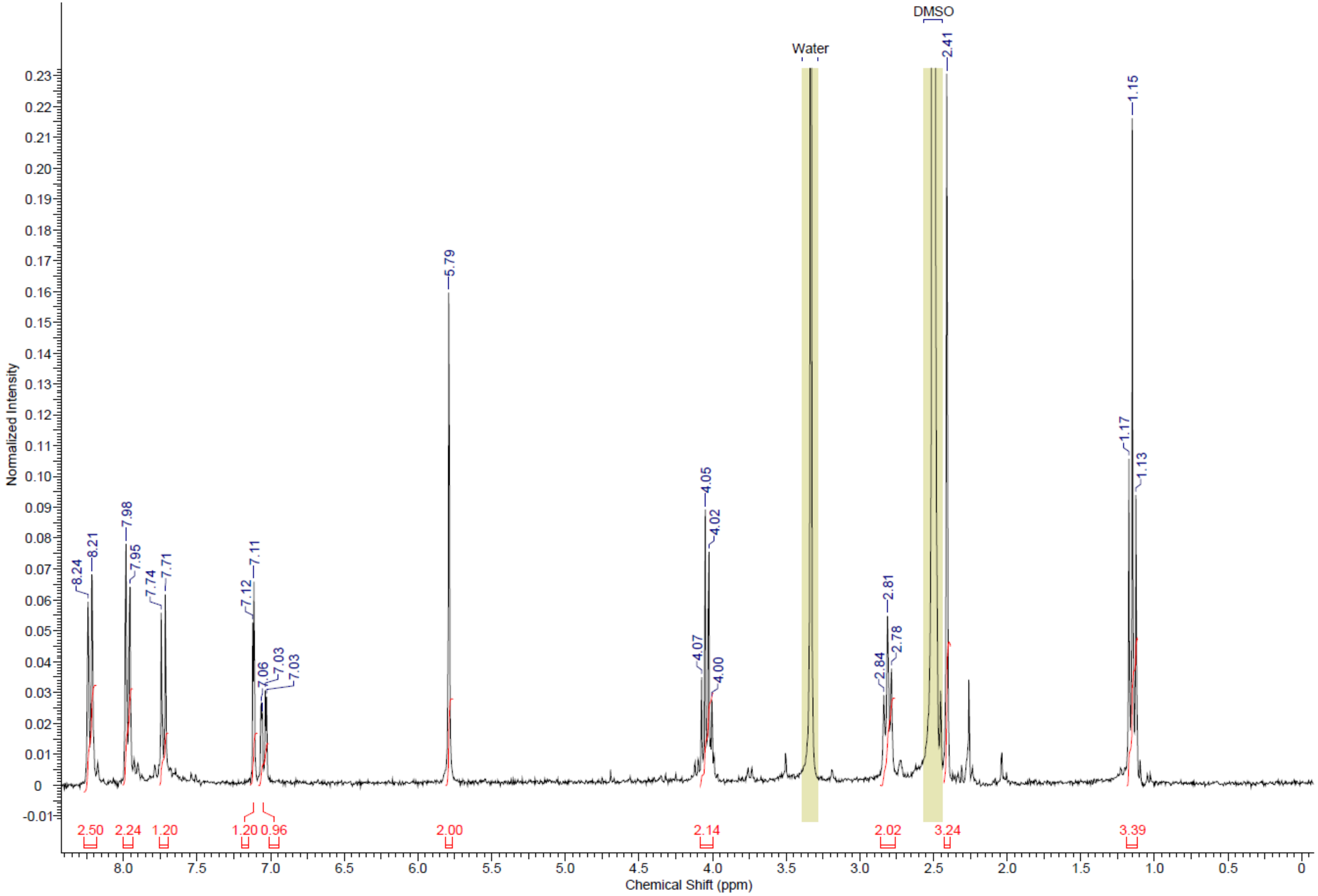

Supporting Information Figure 46: ^{1}H NMR spectrum of **Scaff10-28**, 300 MHz, DMSO-d_6.

4.1.26 3-[4-Methyl-2-oxo-7-[2-oxo-2-[4-(trifluoromethyl)phenyl]ethoxy]chromen-3-yl]propanoic acid (**Scaff10-29**)

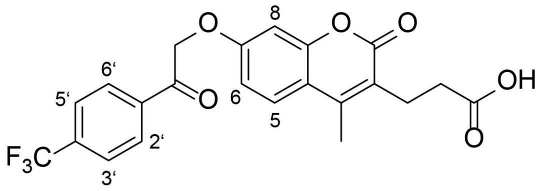

Ethyl 3-[4-methyl-2-oxo-7-[2-oxo-2-[4-(trifluoromethyl)phenyl]ethoxy]chromen-3-yl]propanoate (**Scaff10-28**) (45 mg, 0.10 mmol) was saponificated in a mixture of 2 ml ACN and 1 ml 1 M sodium hydroxide at 70 °C for 1 h. After concentration of the product through removal of solvent under reduced pressure, the product was purified using preparative HPLC with the following gradient: 10 % to 90 % ACN in H_2O in 80 min. The product eluted at t_R = 27 min and was obtained after lyophilisation as yellowish solid in 18 % yield (8 mg).

^{1}H NMR (300 MHz, DMSO-d_6): δ(ppm) = 1.88 – 2.02 (t, J = 7.32 Hz, 2H, -CH_2-C**H_2**-COOH), 2.36 – 2.44 (s, 3H, Ar-CH_3), 2.63 – 2.72 (t, J = 7.32 Hz, 2H, -C**H_2**-CH_2-COOH), 5.73 – 5.81 (s, 2H, -CO-C**H_2**-OAr), 6.98 – 7.05 (dd, J = 2.44 Hz, 8.55 Hz, 1H, H-6), 7.06 – 7.10 (d, J = 2.44 Hz, 1H, H-8), 7.65 – 7.71 (d, J = 8.54 Hz, 1H, H-5), 7.93 – 8.00 (d, J = 8.54 Hz , 2H, H-3', -5'), 8.20 – 8.27 (d, J = 8.54 Hz, 2H, H-2', -6')

ESI-MS: cal. 435.10, exp. 435.1 ($[M+H]^+$)

HPLC: 98 % purity at 254 nm

t_R (HPLC): 6.29 min; λ_{max} = 289 nm and 320 nm

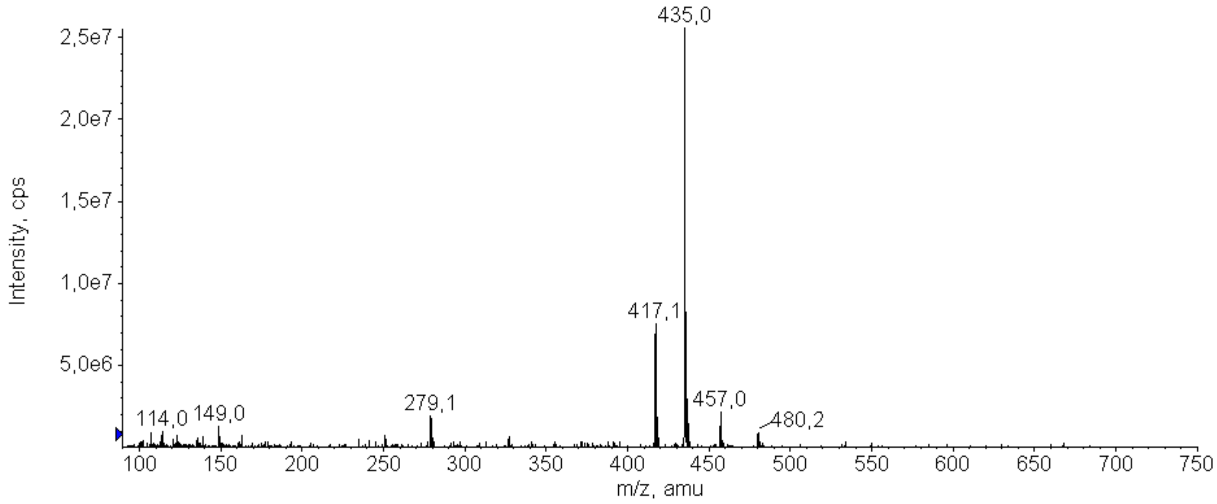

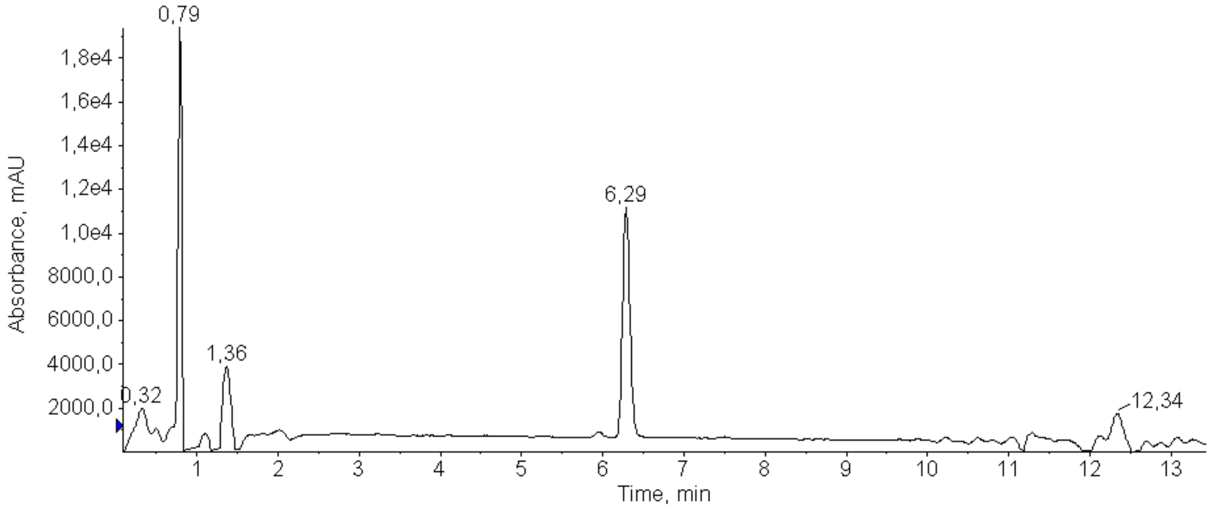

Supporting Information Figure 47: LC/MS analysis of **Scaff10-29**. Upper panel: Mass spectrum. Lower panel: according chromatogram.

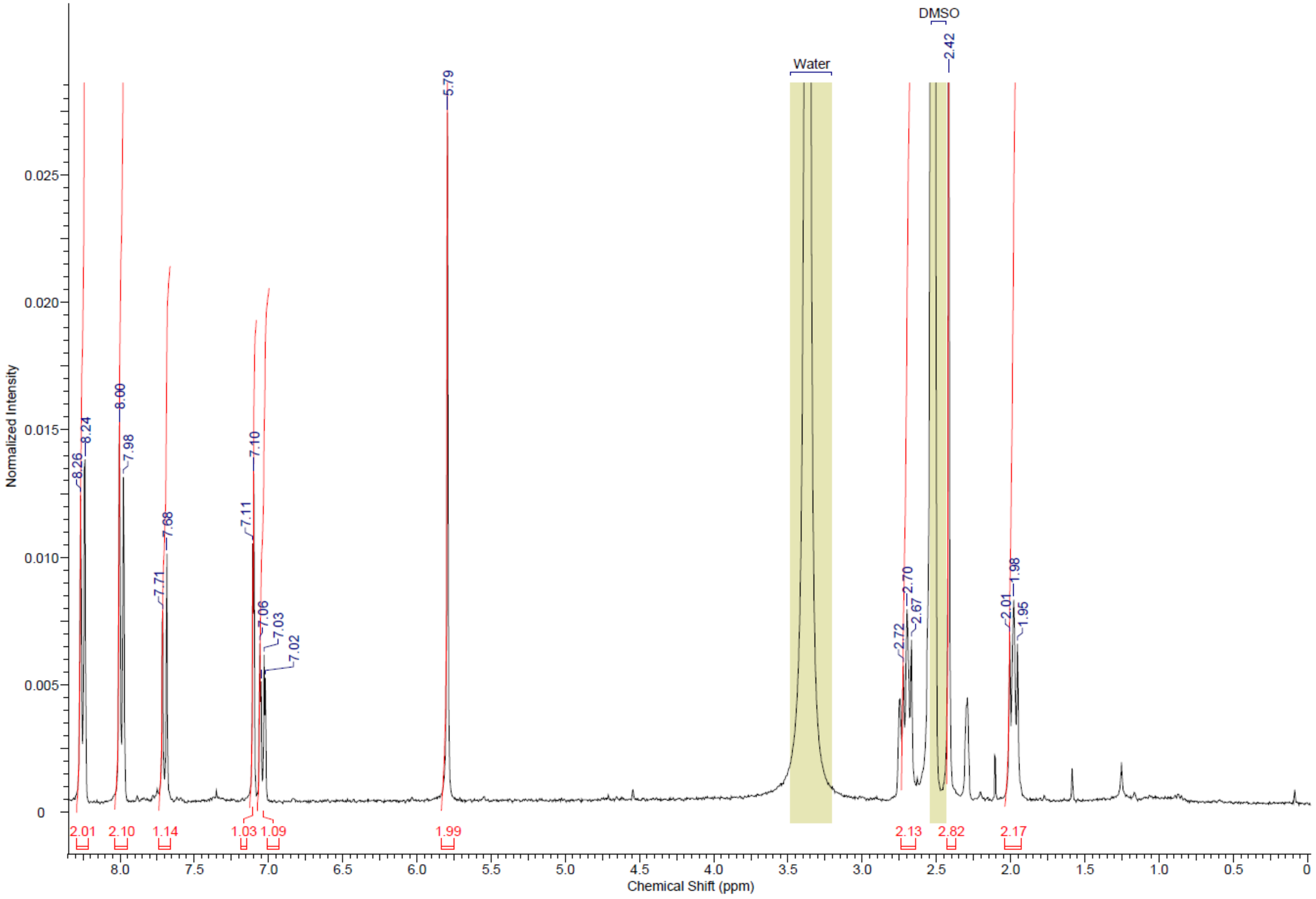

Supporting Information Figure 48: ^{1}H NMR spectrum of **Scaff10-29**, 300 MHz, DMSO-d_6.

4.1.27 3-[5-Methyl-7-oxo-3-[4-(trifluoromethyl)phenyl]furo[3,2-*g*]chromen-6-yl]propanoic acid (**Scaff10-30**)

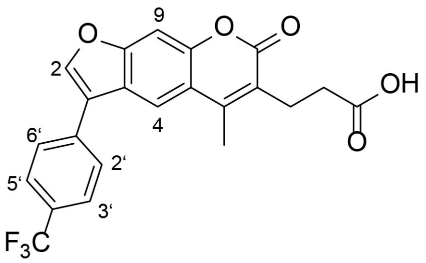

Ethyl 3-[4-methyl-2-oxo-7-[2-oxo-2-[4-(trifluoromethyl)phenyl]ethoxy]chromen-3-yl]propanoate (**Scaff10-28**) (95 mg, 0.21 mmol) was saponificated in a mixture of 3 ml dry acetone and 3 ml 1 M sodium hydroxide at 80 °C for 3.25 h. Dropwise addition of 1 M HCl resulted in precipitation of the product. The precipitated solid was filtered and purified using preparative HPLC with the following gradient: 5 % to 95 % ACN in H_2O in 80 min. The product eluted at t_R = 11.5 min and was obtained after lyophilisation as yellowish solid in 6 % yield (5 mg).

Due to the pure yield, no ^1H NMR spectrum was obtained.

ESI-MS: cal. 417.01, exp. 417.0 ($[M+H]^+$)

HPLC: 95 % purity at 254 nm

t_R (HPLC): 7.02 min; λ_{max} = 294 nm and 328 nm

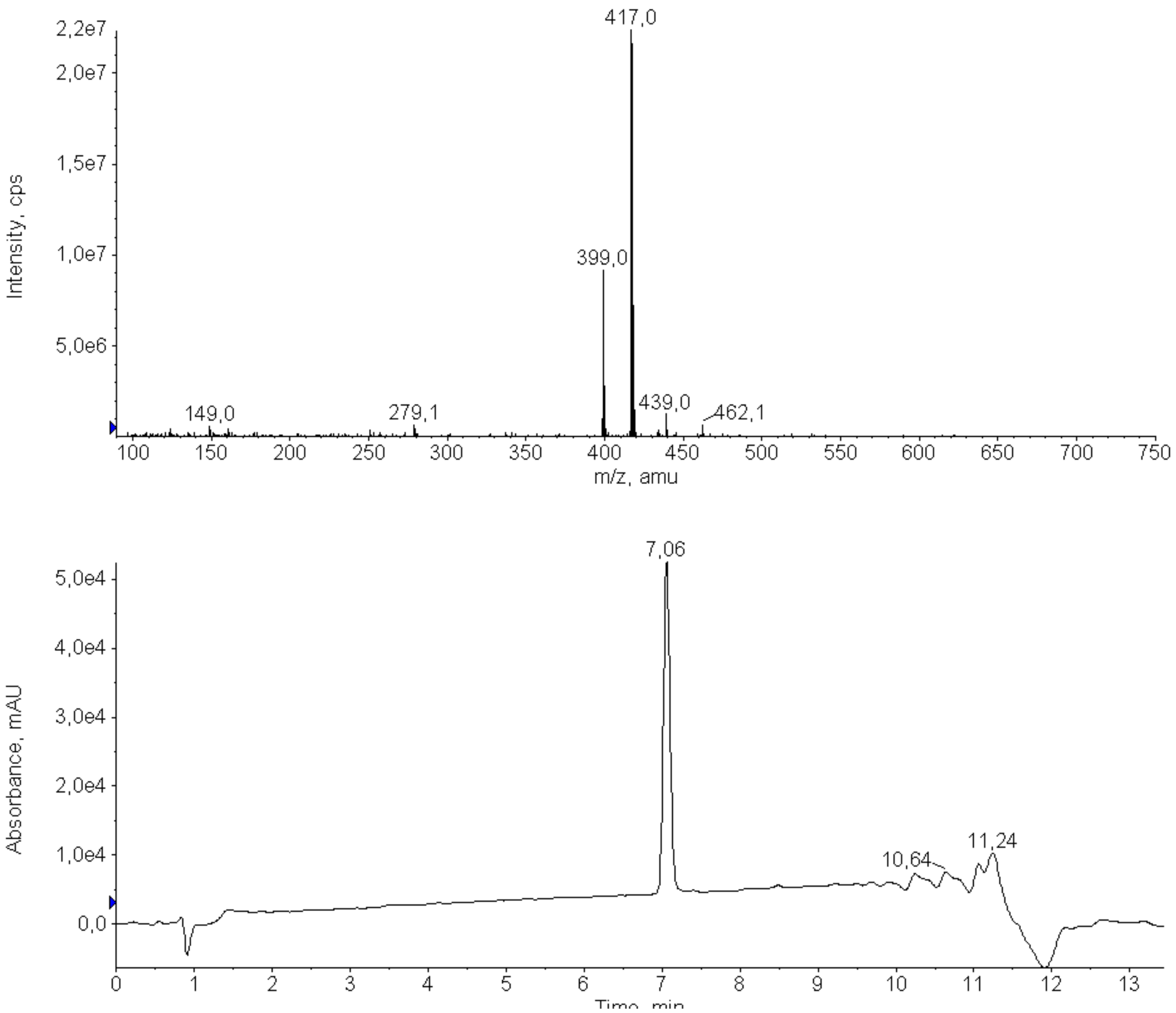

Supporting Information Figure 49: LC/MS analysis of **Scaff10-30**. Upper panel: Mass spectrum. Lower panel: according chromatogram.

4.1.28 Ethyl 3-[4-methyl-2-oxo-7-[2-oxo-2-(4-pyridyl)ethoxy]chromen-3-yl]propanoate (**Scaff10-31**)

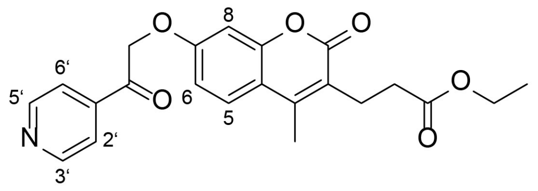

Ethyl 3-(7-hydroxy-4-methyl-2-oxo-chromen-3-yl)propanoate (5**a**) (100 mg, 0.36 mmol) was solved in 0.5 ml DMF and preactivated with potash in excess (2125 mg, 0.91 mmol) at 55 °C. The α-haloketone 2-bromo-1-(4-pyridyl)ethanone (**2j**) was added (79 mg, 0.40 mmol). The final product after purification gave an orange-yellowish solid.

^{1}H NMR (300 MHz, DMSO-d_6): δ(ppm) = 1.10 – 1.12 (t, J = 7.32 Hz, 3H, -CH_2-C**H₃**), 2.38 – 2.45 (s, 3H, Ar-CH_3), 2.47 – 2.53 (t, J = 8.54 Hz, 2H, -CH_2-C**H₂**-COOEt), 2.77 – 2.89 (t, J = 8.54 Hz, 2H, -C**H₂**-CH_2-COOEt), 3.99 – 4.11 (q, J = 7.32 Hz, 2H, -C**H₂**-CH_3), 5.72 – 5.84 (s, 2H, -CO-C**H₂**-OAr), 7.04 – 7.11 (dd, J = 2.44 Hz, 9.77 Hz, 1H, H-6), 7.13 – 7.17 (d, J = 2.44 Hz, 1H, H-8), 7.72 – 7.78 (d, J = 8.54 Hz, 1H, H-5), 7.89 – 7.95 (d, J = 6.10 Hz , 2H, H-2', -6'), 8.86 – 8.92 (d, J = 6.10 Hz, 2H, H-3', -5')

ESI-MS: cal. 396.14, exp. 396.2 ($[M+H]^+$)

HPLC: 95 % purity at 254 nm

t_R (HPLC): 5.39 min; λ_{max} = 286 nm and 318 nm

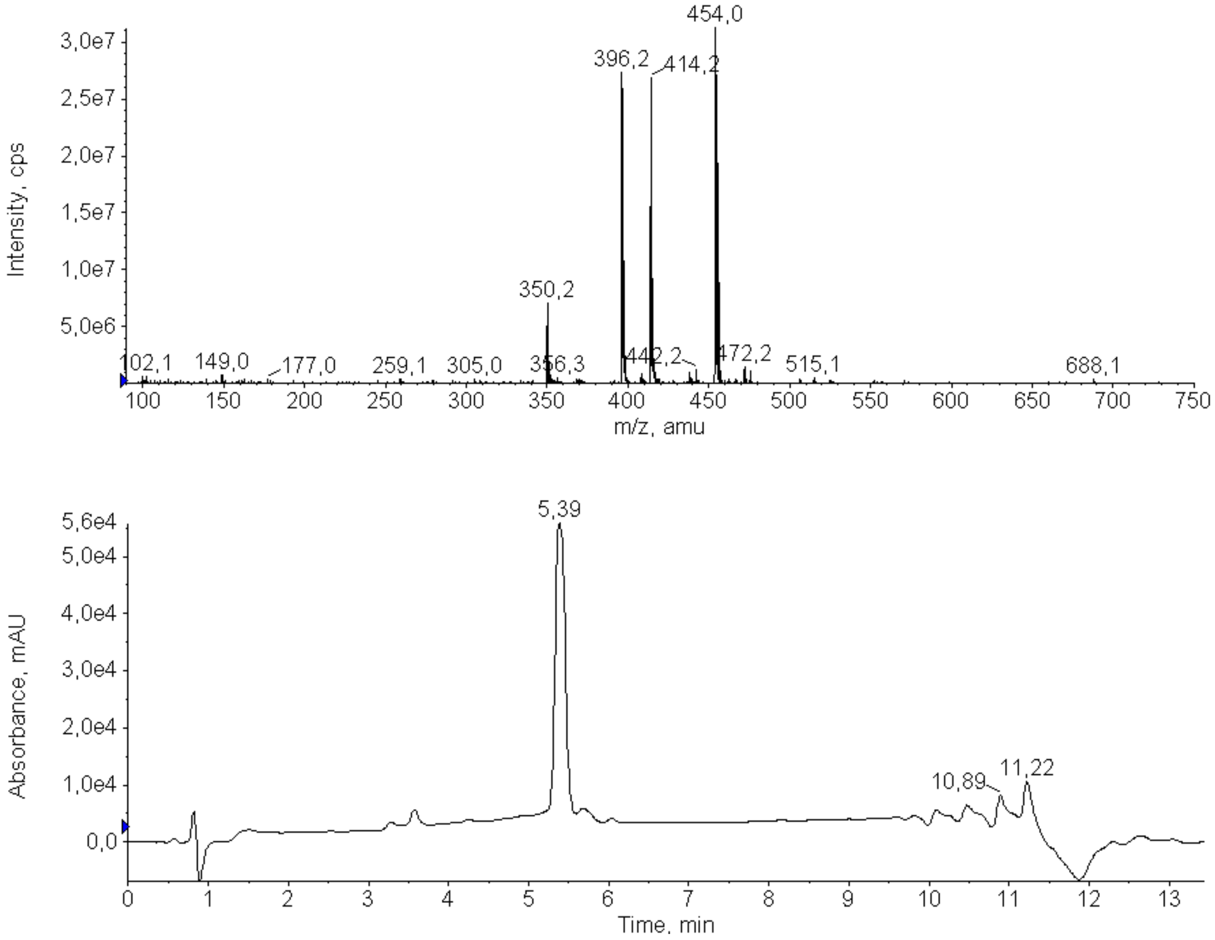

Supporting Information Figure 50: LC/MS analysis of **Scaff10-31**. Upper panel: Mass spectrum. Lower panel: according chromatogram.

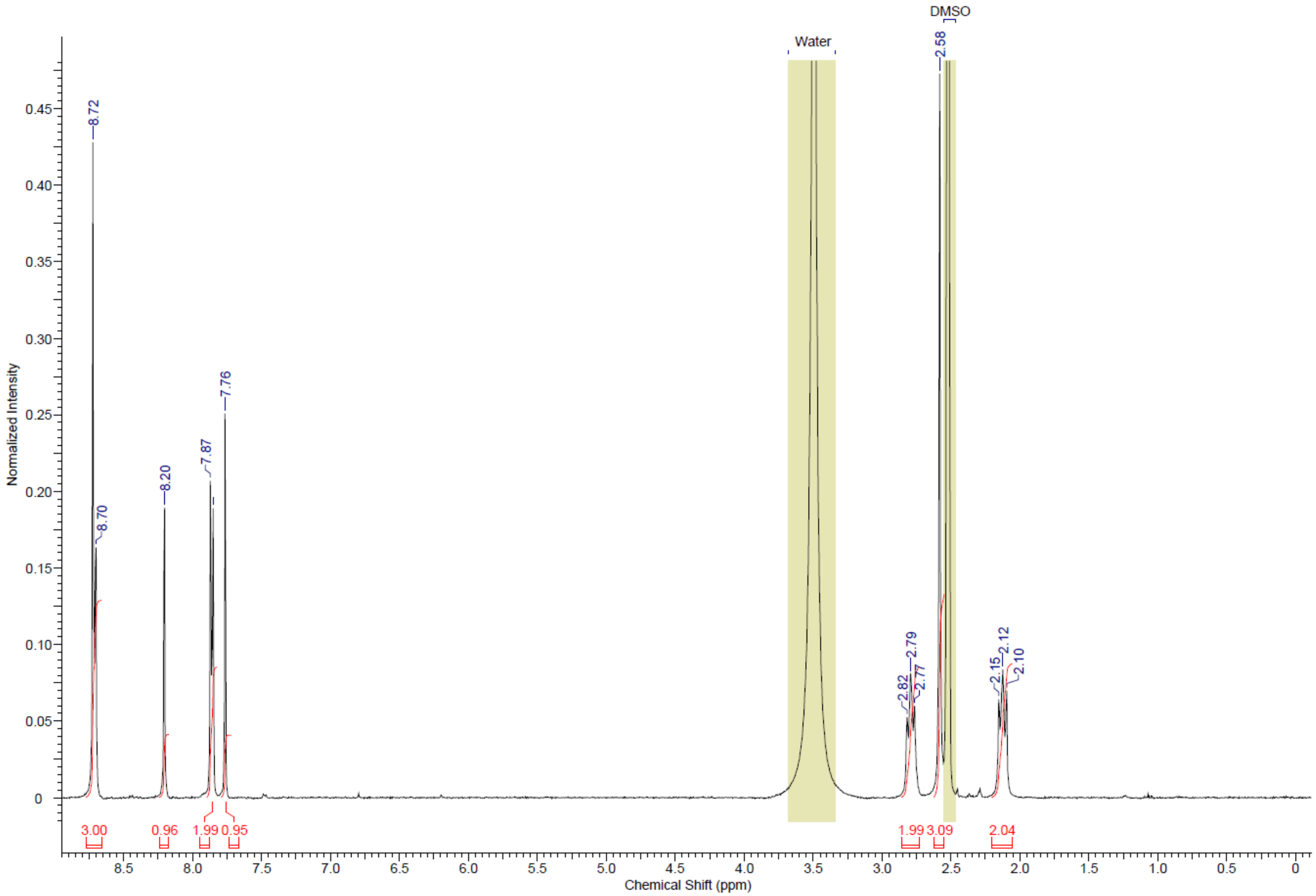

Supporting Information Figure 51: ^{1}H NMR spectrum of **Scaff10-31**, 300 MHz, DMSO-d_6.

4.1.29 3-[5-Methyl-7-oxo-3-(4-pyridyl)furo[3,2-*g*]chromen-6-yl]propanoic acid (**Scaff10-32**)

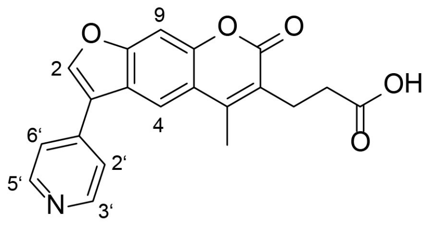

Ethyl 3-[4-methyl-2-oxo-7-[2-oxo-2-(4-pyridyl)ethoxy]chromen-3-yl]propanoate (**Scaff10-31**) (15 mg, 0.04 mmol) was saponificated 1 M sodium hydroxide at 80 °C. The final product after purification gave a bright yellow solid in 31 % yield (4 mg).

Due to the pure yield, no ^{1}H NMR spectrum was obtained.

ESI-MS: cal. 350.10, exp. 350.0 ($[M+H]^+$)

HPLC: 98 % purity at 254 nm

t_R (HPLC): 3.05 min; λ_{max} = 241 nm and 322 nm

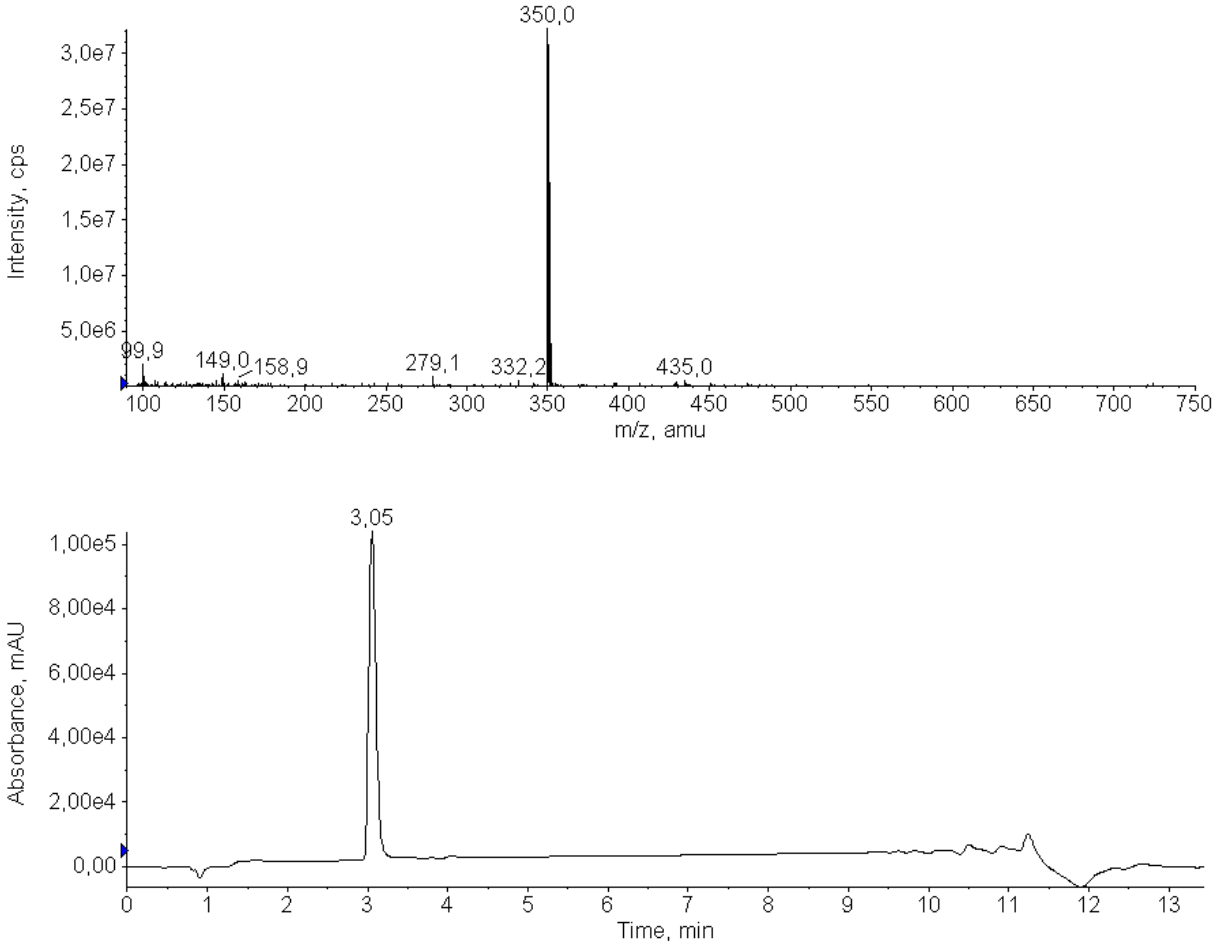

Supporting Information Figure 52: LC/MS analysis of **Scaff10-32**. Upper panel: Mass spectrum. Lower panel: according chromatogram.

4.1.30 Ethyl 3-[7-[2-[4-(dimethylamino)phenyl]-2-oxo-ethoxy]-4-methyl-2-oxo-chromen-3-yl]propanoate (**Scaff10-34**)

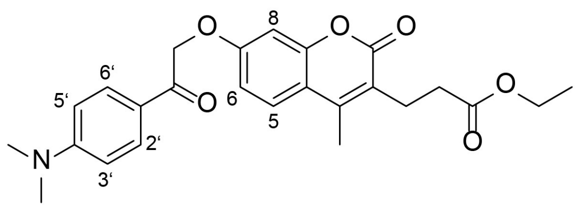

Ethyl 3-(7-hydroxy-4-methyl-2-oxo-chromen-3-yl)propanoate (**5a**) (95 mg, 0.34 mmol) was solved in 5 ml dry acetone and preactivated with potash in excess (124 mg, 0.90 mmol) at 55 °C. The α-haloketone 2-bromo-1-(4-(dimethylamino)phenyl)ethanone (**2k**) was added (88 mg, 0.36 mmol). The mixture was refluxed at the same temperature for 16 h. The product was precipitated using 1 M H_2SO_4 and recrystallised in ethanol. The final product gave an ivory white solid in 40 % yield (60 mg).

^{1}H NMR (300 MHz, DMSO-d_6): δ(ppm) = 1.11 – 1.26 (t, J = 7.32 Hz, 3H, -CH_2-C**H_3**), 2.37 – 2.46 (s, 3H, Ar-CH_3), 2.44 – 2.52 (t, J = 8.54 Hz, 2H, -CH_2-C**H_2**-COOEt), 2.76 – 2.90 (t, J = 8.54 Hz, 2H, -C**H_2**-CH_2-COOEt), 2.97 – 3.18 (s, 6H, -N$(CH_3)_2$), 4.00 – 4.15 (q, J = 7.32 Hz, 2H, -C**H_2**-CH_3), 5.52 – 5.65 (s, 2H, -CO-C**H_2**-OAr), 6.72 – 6.85 (d, J = 8.54 Hz, 2H, H-3’, -5’), 6.94 – 7.06 (m, 2H, H-8, -6), 7.68 – 7.78 (d, J = 9.77 Hz, 1H, H-5), 7.83 – 7.94 (d, J = 8.54 Hz , 2H, H-2’, -6’)

ESI-MS: cal. 438.19, exp. 438.1 ($[M+H]^+$)

HPLC: 98 % purity at 254 nm

t_R (HPLC): 6.85 min; λ_{max} = 343 nm

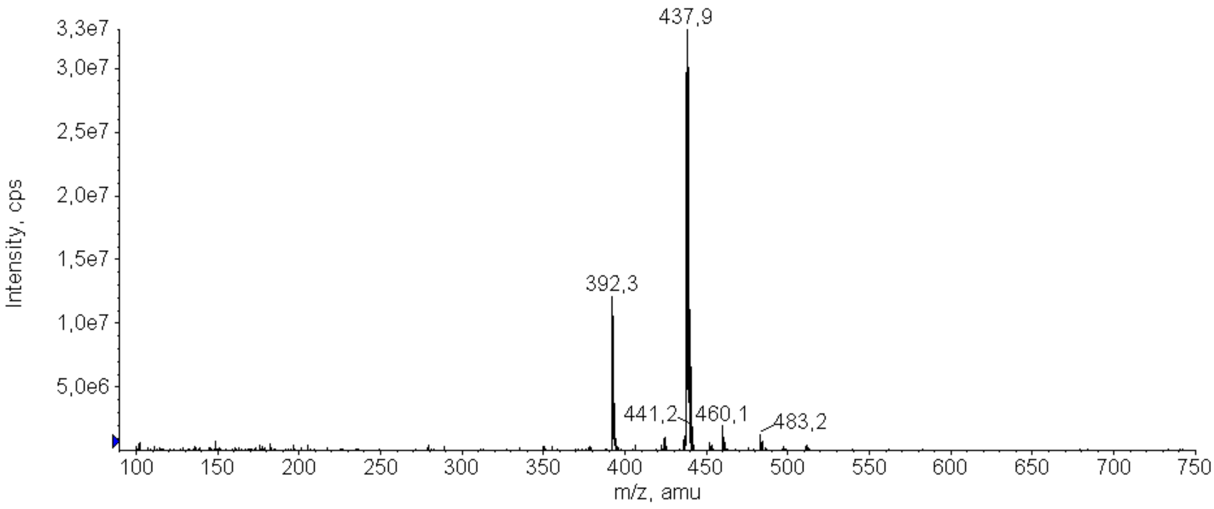

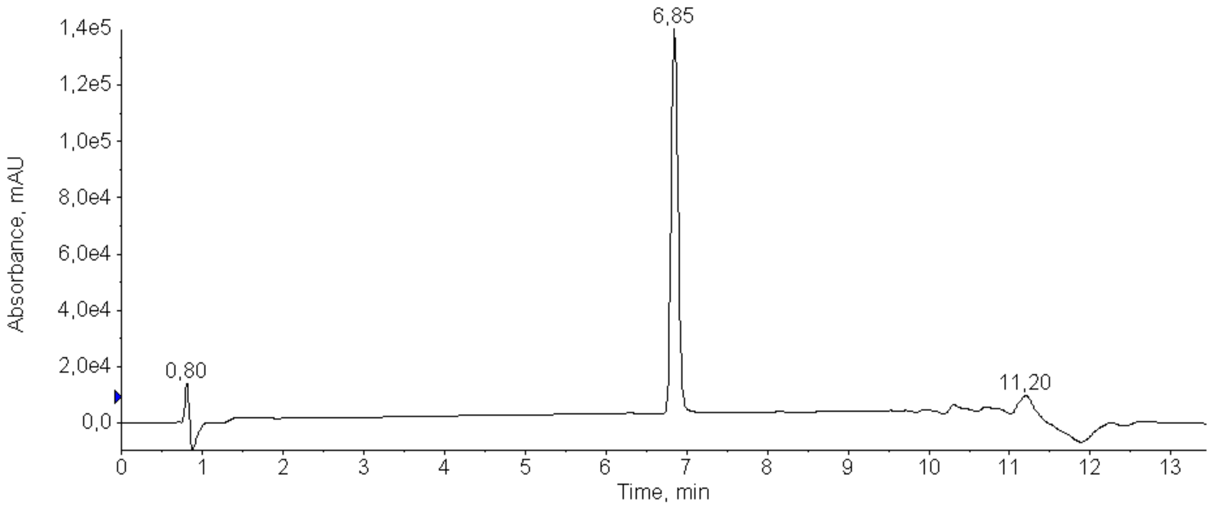

Supporting Information Figure 53: LC/MS analysis of **Scaff10-34**. Upper panel: Mass spectrum. Lower panel: according chromatogram.

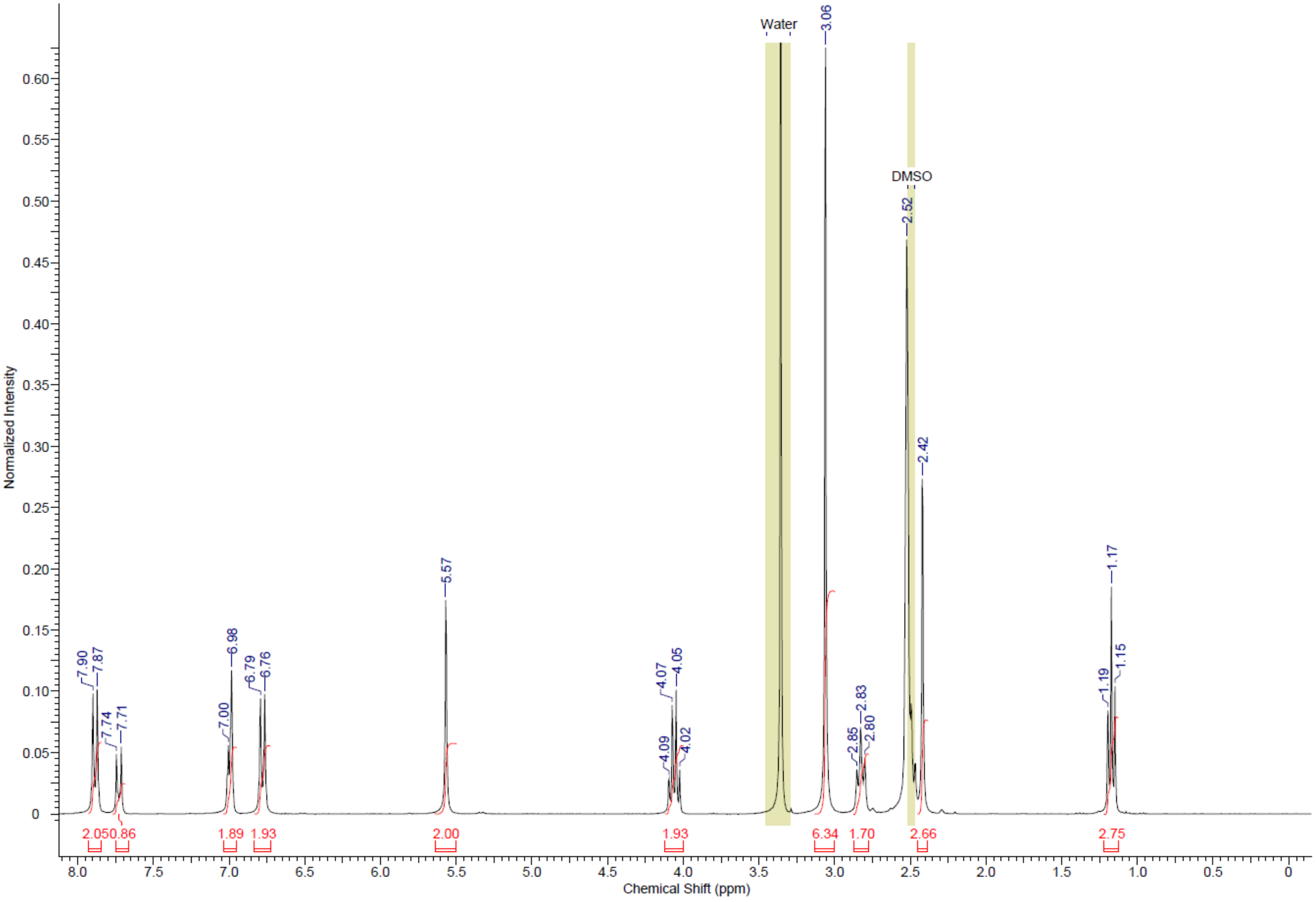

Supporting Information Figure 54: ^{1}H NMR spectrum of **Scaff10-34**, 300 MHz, DMSO-d_6.

4.1.31 3-[7-[2-[4-(Dimethylamino)phenyl]-2-oxo-ethoxy]-4-methyl-2-oxo-chromen-3-yl]propanoic acid (**Scaff10-35**)

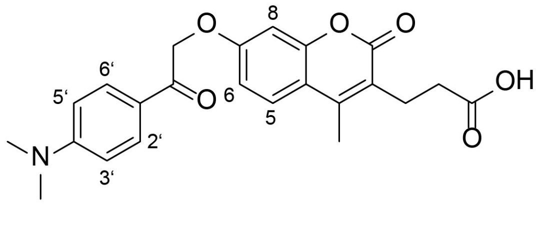

Ethyl 3-[7-[2-[4-(dimethylamino)phenyl]-2-oxo-ethoxy]-4-methyl-2-oxo-chromen-3-yl]propanoate (**Scaff10-34**) (50 mg, 0.11 mmol) was saponificated in 3 ml 1 M sodium hydroxide at 80 °C for 4 h. The product was purified using preparative HPLC with the following gradient: 10 % to 95 % ACN in H_2O in 80 min. The product was obtained after lyophilisation as light yellow solid in 26 % yield (12 mg).

^{1}H NMR (300 MHz, DMSO-d_6): δ(ppm) = 1.89 – 2.02 (t, J = 7.33 Hz, 2H, -CH_2-C$\mathbf{H_2}$-COOH), 2.33 – 2.42 (s, 3H, Ar-CH_3), 2.62 – 2.73 (t, J = 7.33 Hz, 2H, -C$\mathbf{H_2}$-CH_2-COOH), 2.97 – 3.07 (s, 6H, -N$(CH_3)_2$), 5.47 – 5.56 (s, 2H, -CO-C$\mathbf{H_2}$-OAr), 6.71 – 6.79 (d, J = 8.55 Hz, 2H, H-3', -5'), 6.90 – 6.96 (m, 2H, H-6, -8), 7.61 – 7.68 (d, J = 8.55 Hz, 1H, H-5), 7.82 – 7.89 (d, J = 8.55 Hz , 2H, H-2', -6')

ESI-MS: cal. 410.16, exp. 410.1 ($[M+H]^+$)

HPLC: 98 % purity at 254 nm

t_R (HPLC): 5.54 min; λ_{max} = 344 nm

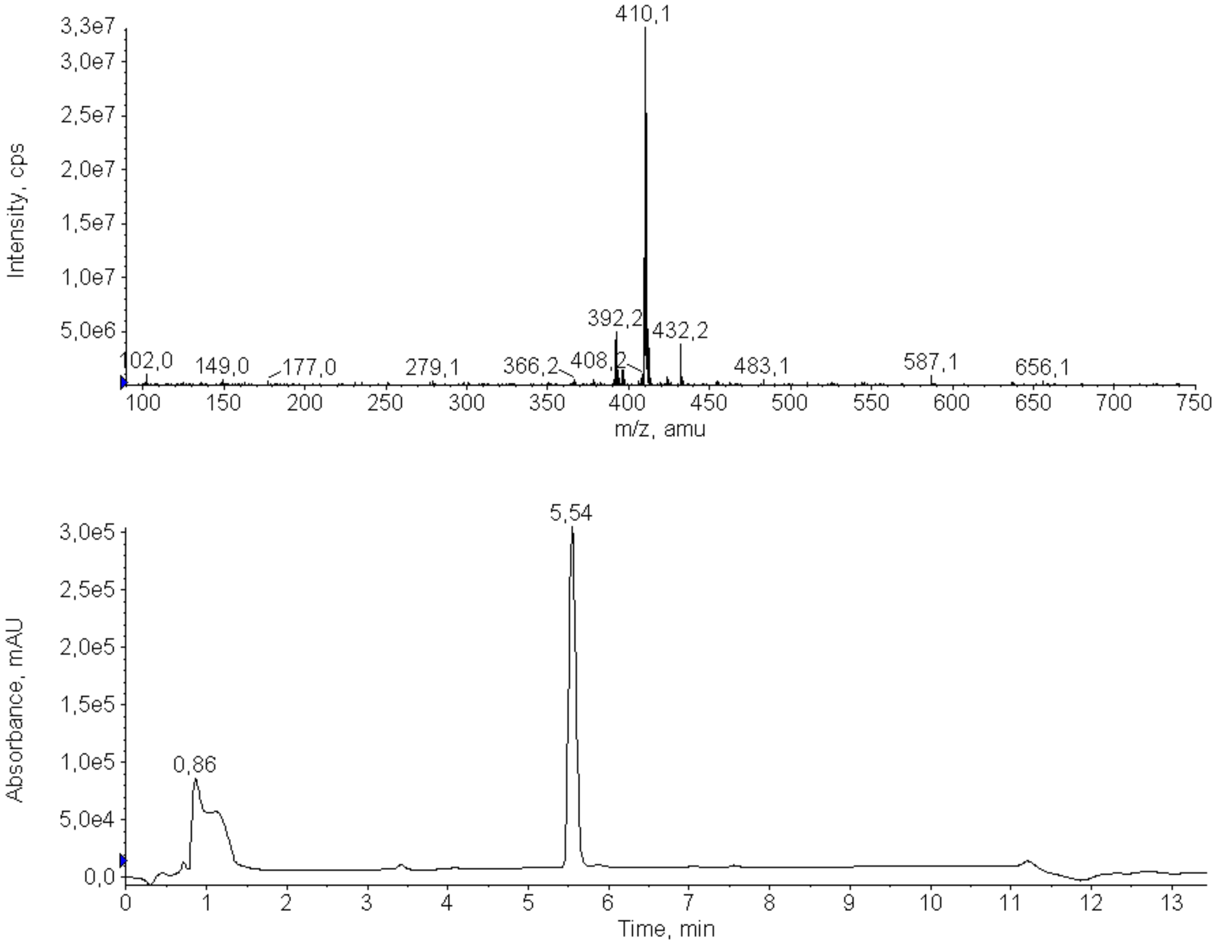

Supporting Information Figure 55: LC/MS analysis of **Scaff10-35**. Upper panel: Mass spectrum. Lower panel: according chromatogram.

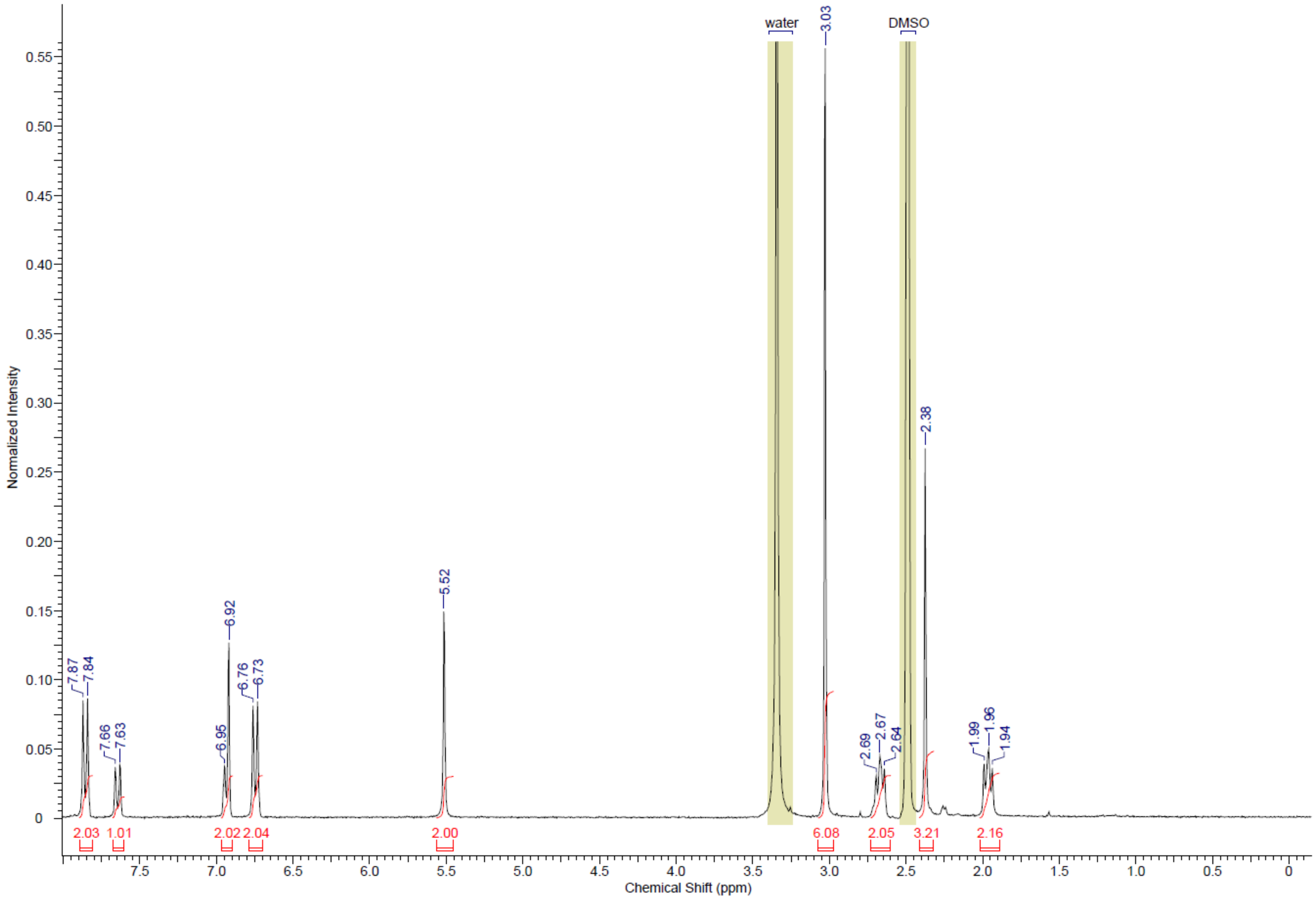

Supporting Information Figure 56: ^{1}H NMR spectrum of **Scaff10-35**, 300 MHz, DMSO-d_6.

4.1.32 3-(4-Methyl-2-oxo-7-[2-oxo-2-(4-phenoxyphenyl)ethoxy]chromen-3-yl)propanoic acid (**Scaff10-37**)

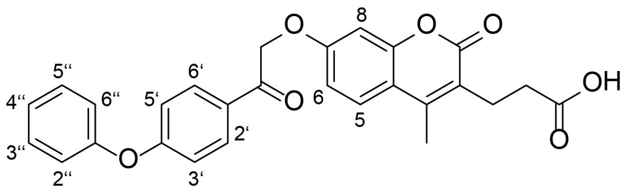

Ethyl 3-[4-methyl-2-oxo-7-[2-oxo-2-(4-phenoxyphenyl)ethoxy]chromen-3-yl]propanoate (**Scaff10-17**) (15 mg, 0.03 mmol) was saponificated in 1 M sodium hydroxide at 80 °C for 1 h. The product was purified using preparative HPLC and was obtained after lyophilisation as ivory yellow solid in 7 % yield (1 mg).

Due to the pure yield, no ^{1}H NMR spectrum was obtained.

ESI-MS: cal. 459.14, exp. 459.1 ($[M+H]^+$)

HPLC: 97 % purity at 254 nm

t_R (HPLC): 6.65 min; λ_{max} = 280 nm and 318 nm

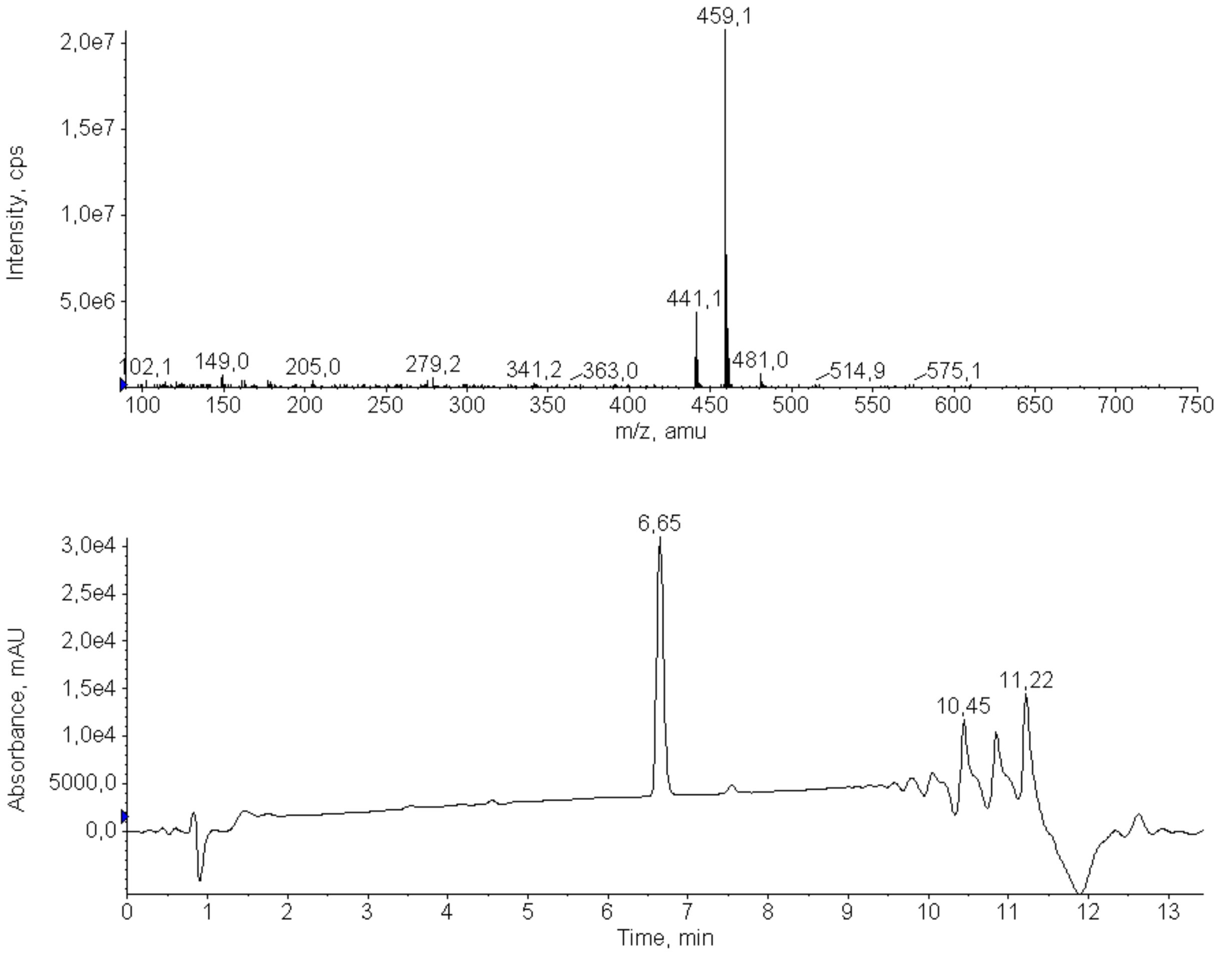

Supporting Information Figure 57: LC/MS analysis of **Scaff10-37**. Upper panel: Mass spectrum. Lower panel: according chromatogram.

4.1.33 3-[5-Methyl-7-oxo-3-(4-phenoxyphenyl)furo[3,2-*g*]chromen-6-yl]propanoic acid (**Scaff10-38**)

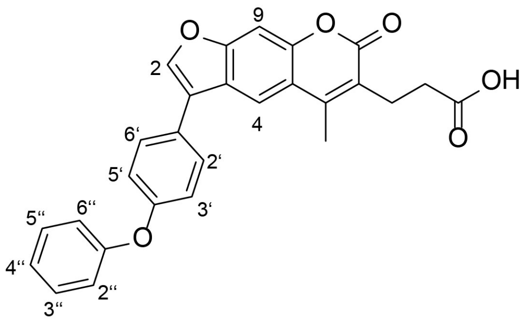

Ethyl 3-[4-methyl-2-oxo-7-[2-oxo-2-(4-phenoxyphenyl)ethoxy]chromen-3-yl]propanoate (**Scaff10-17**) (120 mg, 0.25 mmol) was saponificated in a mixture of 6 ml dry acetone, 1 ml DMSO and 6 ml 1 M sodium hydroxide at 90 °C for 1.5 h. Dropwise addition of 2 M HCl resulted in precipitation of the product. The precipitated solid was purified using preparative HPLC with the following gradient: 20 % to 95 % ACN in H_2O in 80 min. The product eluted at t_R = 30 min and was obtained after lyophilisation as yellow solid in 9 % yield (10 mg).

^1H NMR (300 MHz, DMSO-d_6): δ(ppm) = 1.94 – 2.07 (t, J = 7.32 Hz, 2H, -CH_2-C**H₂**-COOH), 2.55 – 2.62 (s, 3H, Ar-CH_3), 2.71 – 2.82 (t, J = 7.32 Hz, 2H, -C**H₂**-CH_2-COOH), 7.08 – 7.16 (d, J = 8.54 Hz, 2H, H-3', -5'), 7.15 – 7.26 (m, 3H, H-2'', -4'', -6''), 7.42 – 7.52 (t, J = 7.32 Hz, 2H, H-3'', -5''), 7.74 – 7.78 (s, 1H, H-9), 7.82 – 7.90 (d, J = 8.54 Hz, 2H, H-2', -6'), 8.12 – 8.17 (s, 1H, H-4), 8.43 – 8.46 (s, 1H, H-2)

ESI-MS: cal. 441.13, exp. 441.1 ($[M+H]^+$)

HPLC: 97 % purity at 254 nm

t_R (HPLC): 7.63 min; λ_{max} = 245 nm and 302 nm

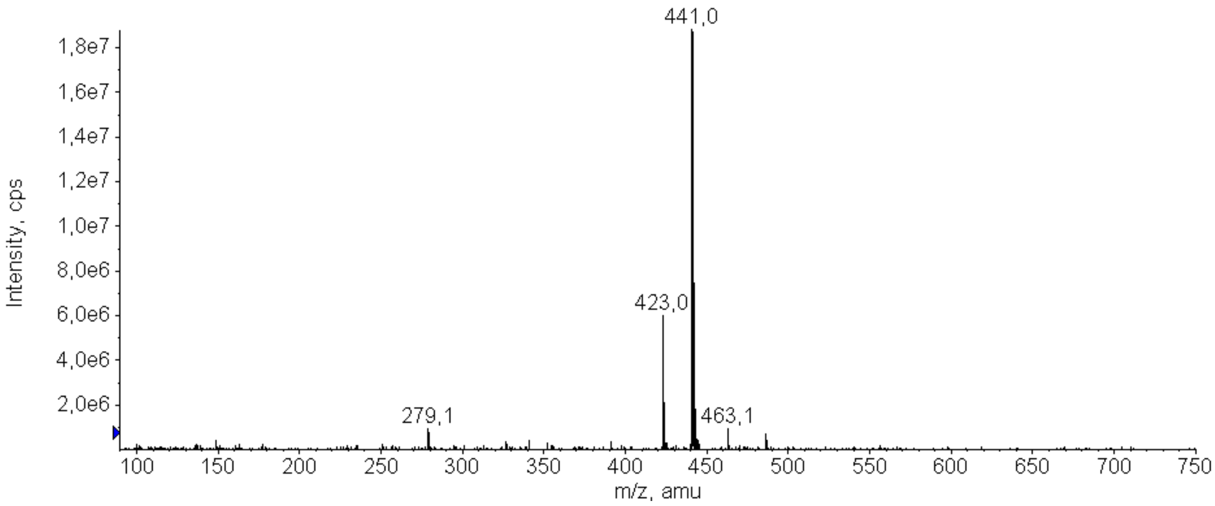

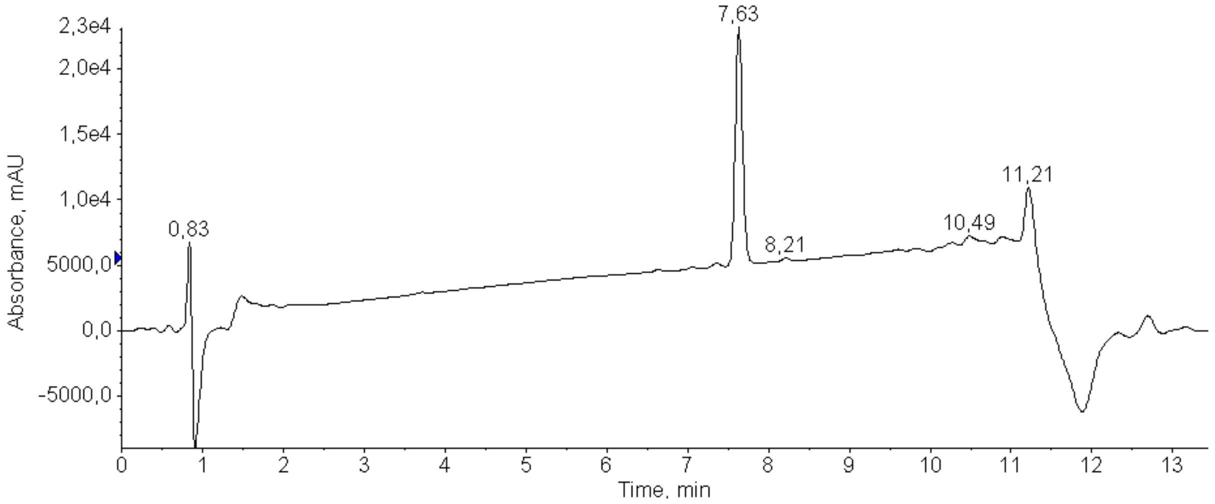

Supporting Information Figure 58: LC/MS analysis of **Scaff10-38**. Upper panel: Mass spectrum. Lower panel: according chromatogram.

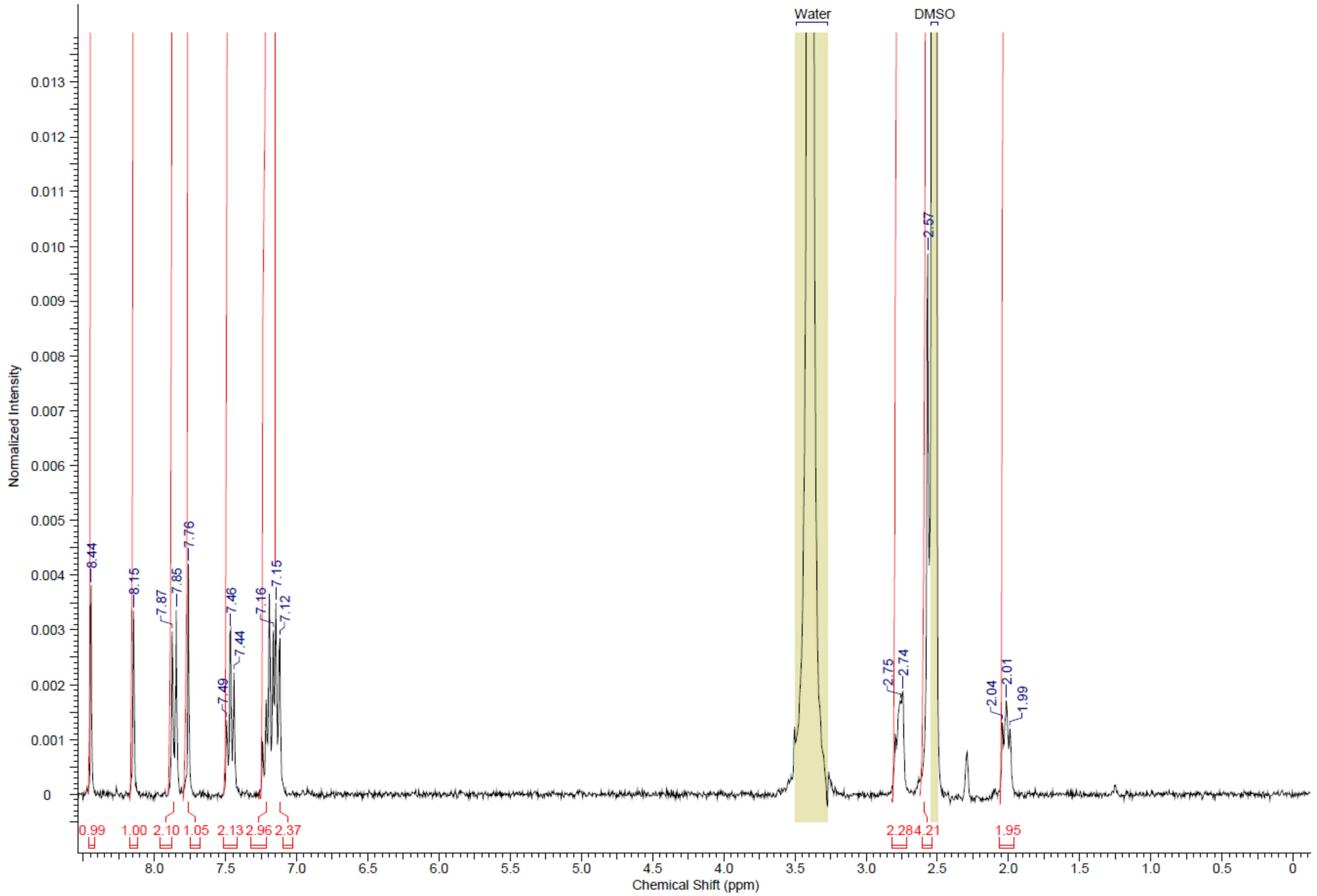

Supporting Information Figure 59: ^{1}H NMR spectrum of **Scaff10-38**, 300 MHz, DMSO-d_6.

4.1.34 Ethyl 3-[4-methyl-2-oxo-7-[2-oxo-2-(4-phenylphenyl)ethoxy]chromen-3-yl]propanoate (**Scaff10-39**)

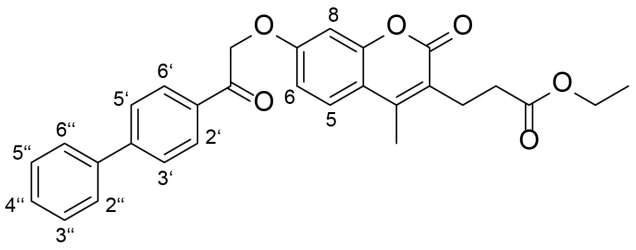

Ethyl 3-(7-hydroxy-4-methyl-2-oxo-chromen-3-yl)propanoate (**5a**) (100 mg, 0.36 mmol) was solved in 6 ml dry acetone and preactivated with potash in excess (125 mg, 0.90 mmol) at 55 °C. The α-haloketone 2-bromo-1-(4-phenylphenyl)ethanone (**2f**) was added (110 mg, 0.40 mmol). The mixture was refluxed at the same temperature for 2.5 h. The product was precipitated using 1 M H_2SO_4 and recrystallised in ethanol. The final product gave a white solid in 99 % yield (168 mg).

^{1}H NMR (300 MHz, DMSO-d_6): δ(ppm) = 1.07 – 1.24 (t, J = 7.33 Hz, 3H, -CH_2-C**H_3**), 2.34 – 2.43 (s, 3H, Ar-CH_3), 2.41 – 2.49 (t, J = 7.33 Hz, 2H, -CH_2-C**H_2**-COOEt), 2.75 – 2.89 (t, J = 7.33 Hz, 2H, -C**H_2**-CH_2-COOEt), 3.98 – 4.12 (q, J = 7.33 Hz, 2H, -C**H_2**-CH_3), 5.71 – 5.85 (s, 2H, -CO-C**H_2**-OAr), 7.00 – 7.12 (m, 2H, H-6, -8), 7.42 – 7.58 (m, 3H, H-5, -3'', -5''), 7.69 – 7.83 (m, 3H, H-2'', -4'', -6''), 7.86 – 7.95 (d, J = 8.55 Hz, 2H, H-3', -5'), 8.08 – 8.17 (d, J = 7.33 Hz, 2H, H-2', -6')

ESI-MS: cal. 471.18, exp. 471.2 ($[M+H]^+$)

HPLC: 95 % purity at 254 nm

t_R (HPLC): 7.66 min; λ_{max} = 290 nm and 306 nm

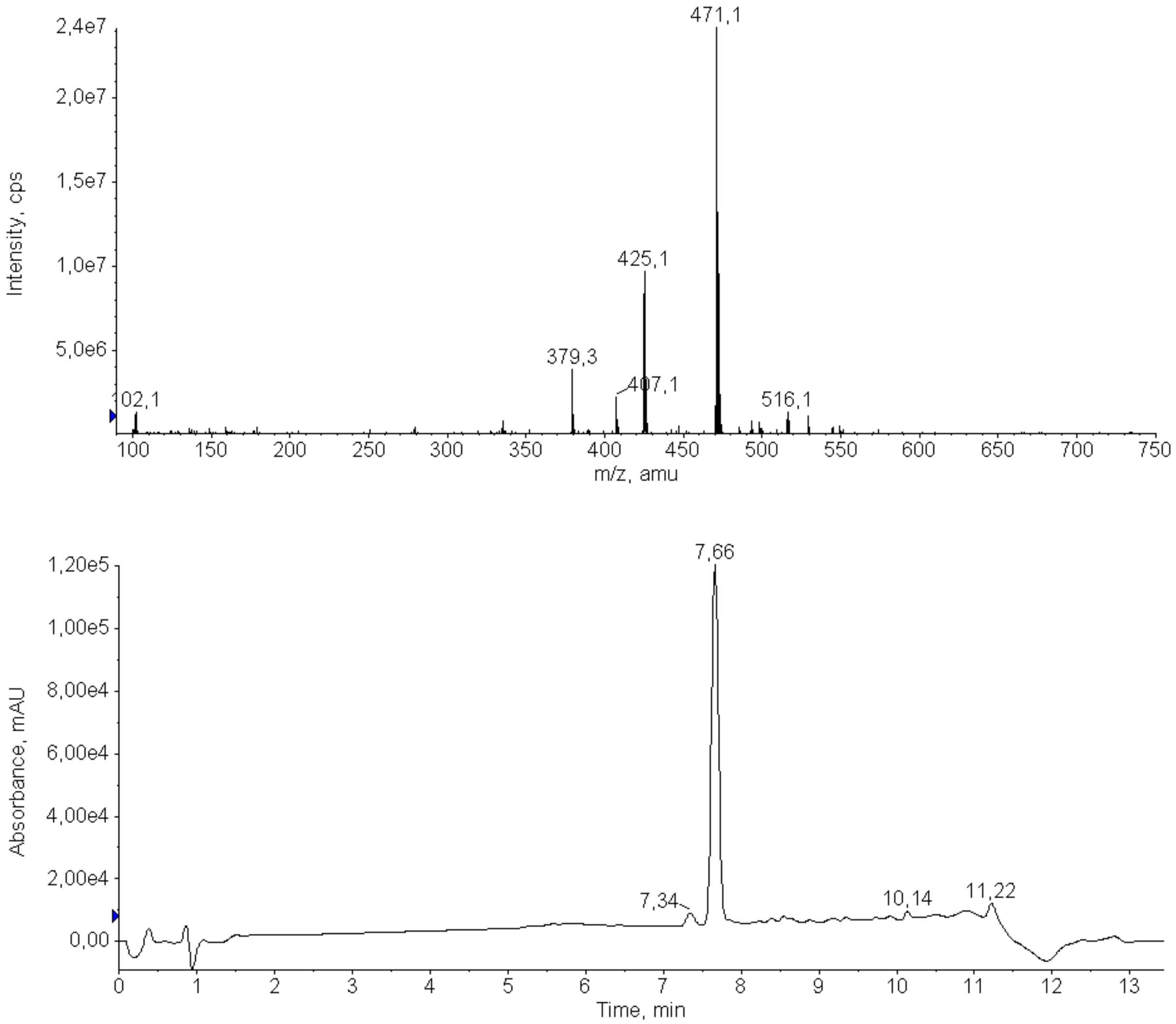

Supporting Information Figure 60: LC/MS analysis of **Scaff10-39**. Upper panel: Mass spectrum. Lower panel: according chromatogram.

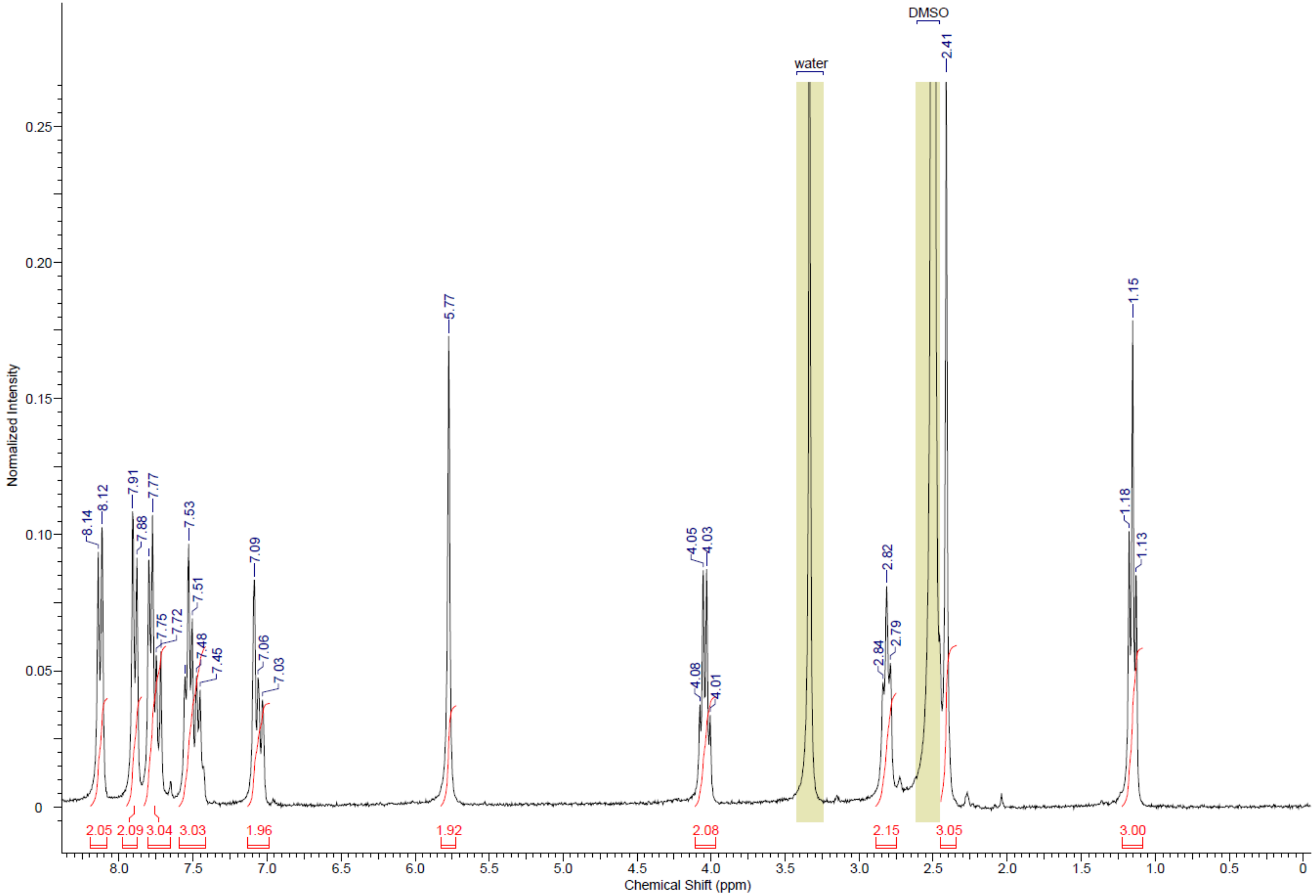

Supporting Information Figure 61: ^{1}H NMR spectrum of **Scaff10-39**, 300 MHz, DMSO-d_6.

4.1.35 3-[4-Methyl-2-oxo-7-[2-oxo-2-(4-phenylphenyl)ethoxy]chromen-3-yl]propanoic acid (**Scaff10-40**)

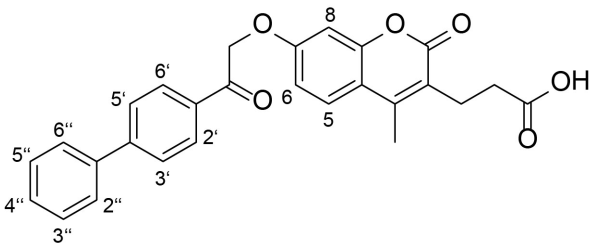

Ethyl 3-[4-methyl-2-oxo-7-[2-oxo-2-(4-phenylphenyl)ethoxy]chromen-3-yl]propanoate (**Scaff10-39**) (55 mg, 0.12 mmol) was saponificated in a mixture of 1 ml DMF and 1 ml 1 M sodium hydroxide at 55 °C for 1 h. The product was purified using preparative HPLC with the following gradient: 10 % to 90 % ACN in H_2O in 80 min. The product eluted at t_R = 40 min and was obtained after lyophilisation as white solid in 35 % yield (18 mg).

^{1}H NMR (300 MHz, DMSO-d_6): δ(ppm) = 2.02 – 2.13 (t, J = 7.32 Hz, 2H, -CH_2-C**H₂**-COOH), 2.33 – 2.43 (s, 3H, Ar-CH_3), 2.64 – 2.77 (t, J = 7.32 Hz, 2H, -C**H₂**-CH_2-COOH), 5.70 – 5.80 (s, 2H, -CO-C**H₂**-OAr), 6.98 – 7.09 (m, 2H, H-6, -8), 7.41 – 7.49 (t, J = 7.32 Hz, 1H, H-4''), 7.48 – 7.58 (t, J = 7.32 Hz, 3H, H-3'',-5''), 7.65 – 7.72 (d, J = 8.54 Hz , 1H, H-5), 7.74 – 7.83 (d, J = 7.32 Hz, 2H, H-2'', -6''), 7.86 – 7.93 (d, J = 8.55 Hz, 2H, H-2', -6'), 8.09 – 8.17 (d, J = 8.55 Hz, 2H, H-3', -5')

ESI-MS: cal. 443.14, exp. 443.1 ($[M+H]^+$)

HPLC: 98 % purity at 254 nm

t_R (HPLC): 6.69 min; λ_{max} = 293 nm and 308 nm

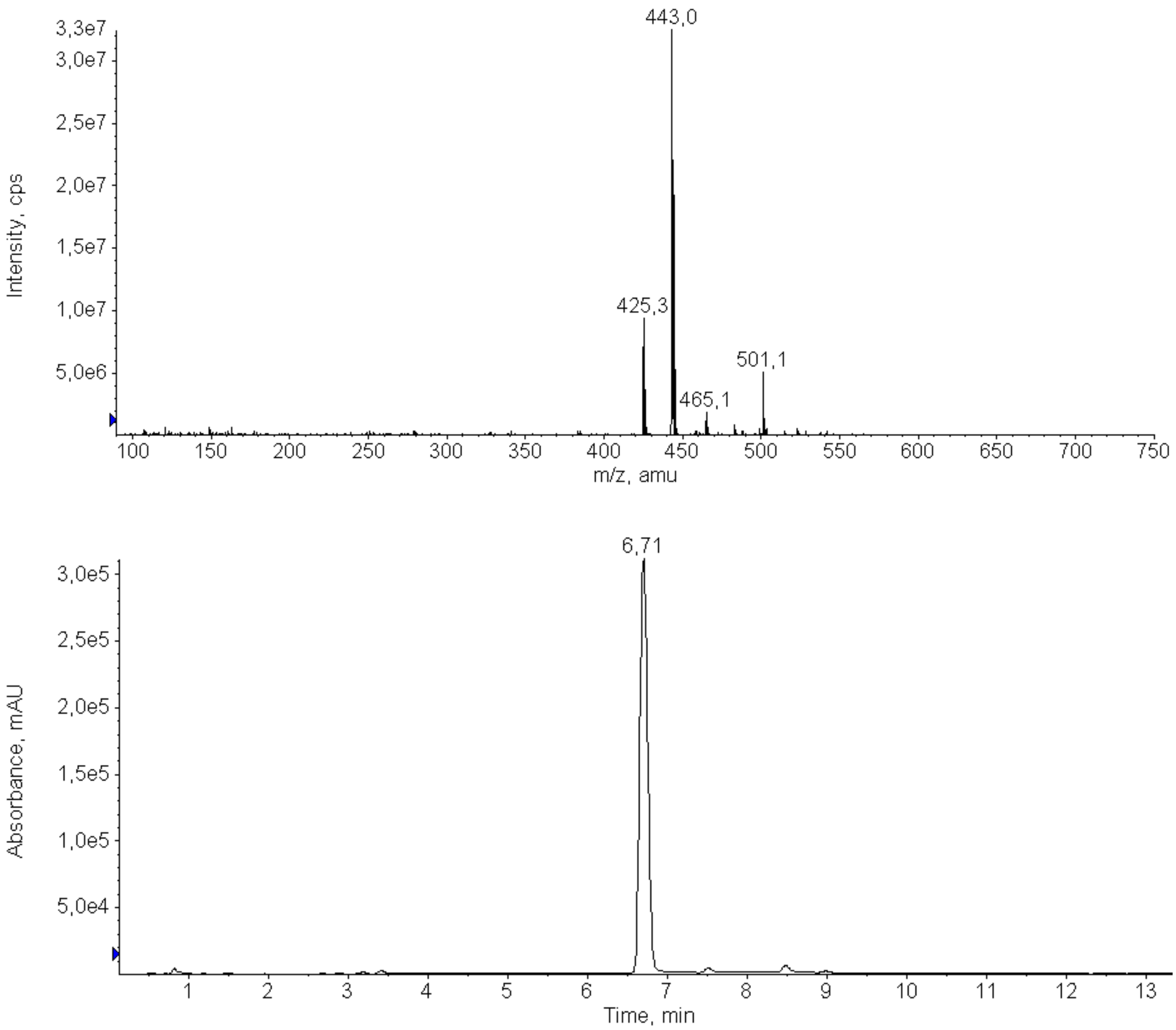

Supporting Information Figure 62: LC/MS analysis of **Scaff10-40**. Upper panel: Mass spectrum. Lower panel: according chromatogram.

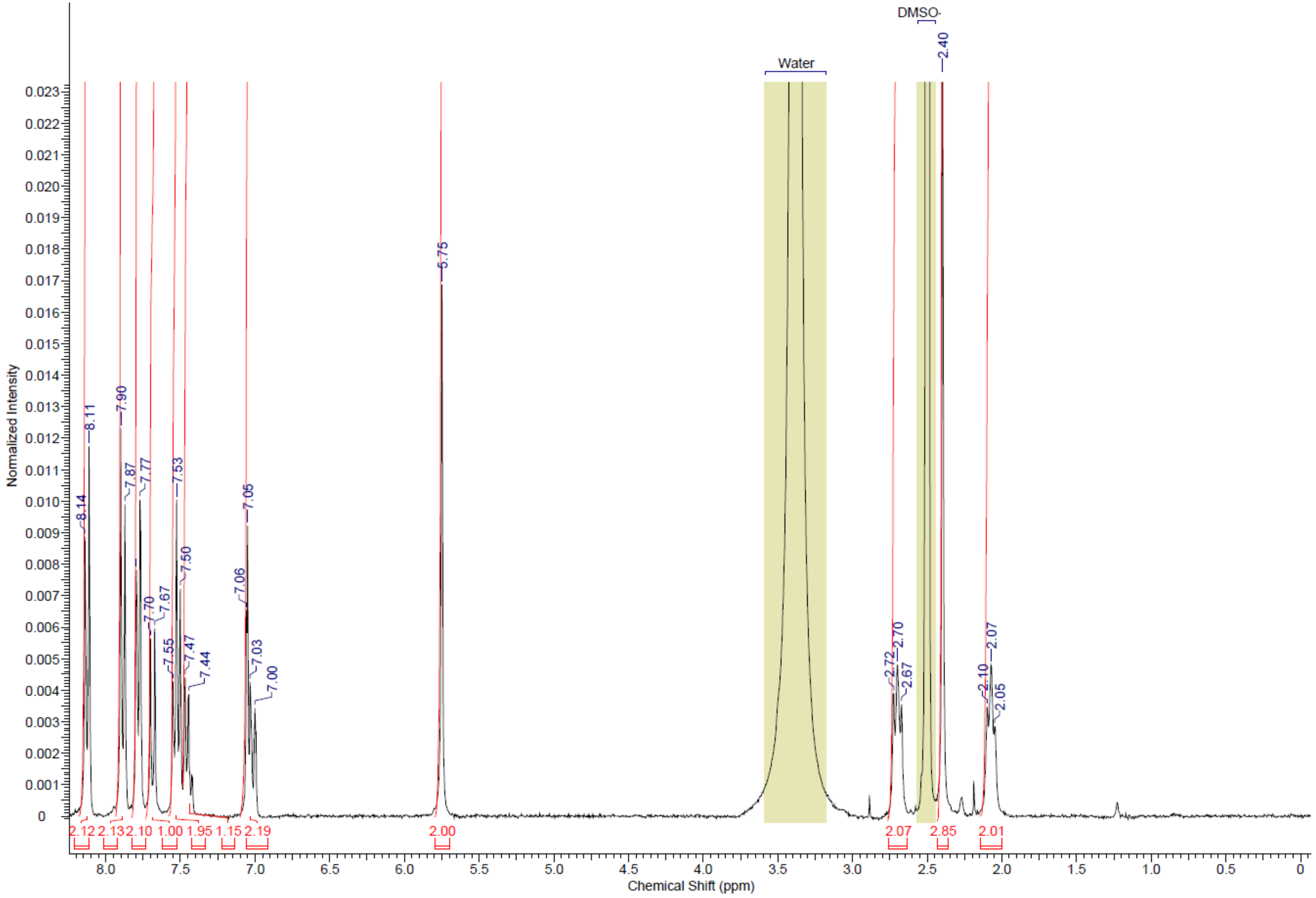

Supporting Information Figure 63: ^{1}H NMR spectrum of **Scaff10-40**, 300 MHz, DMSO-d_6.

4.1.36 3-[5-Methyl-7-oxo-3-(4-phenylphenyl)furo[3,2-*g*]chromen-6-yl]propanoic acid (**Scaff10-41**)

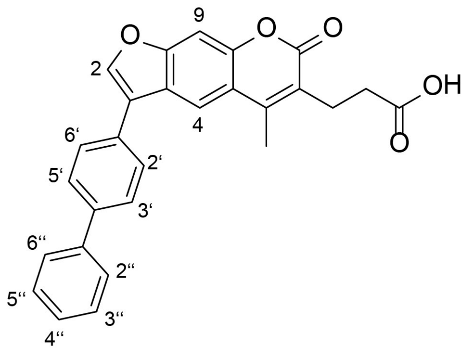

Ethyl 3-[4-methyl-2-oxo-7-[2-oxo-2-(4-phenylphenyl)ethoxy]chromen-3-yl]propanoate (**Scaff10-39**) (120 mg, 0.26 mmol) was saponificated in a mixture of 7 ml dry acetone and 7 ml 1 M sodium hydroxide at 90 °C for 1.5 h. Dropwise addition of 1 M HCl resulted in precipitation of the product. The precipitated solid was purified using preparative HPLC with the following gradient: 5 % to 95 % ACN in H_2O in 80 min. The product eluted at t_R = 34.5 min and was obtained after lyophilisation as yellowish solid in 34 % yield (37 mg).

^{1}H NMR (300 MHz, DMSO-d_6): δ(ppm) = 2.00 – 2.14 (t, J = 8.54 Hz, 2H, -CH_2-C**H₂**-COOH), 2.56 – 2.66 (s, 3H, Ar-CH_3), 2.73 – 2.86 (t, J = 8.54 Hz, 2H, -C**H₂**-CH_2-COOH), 7.37 – 7.47 (t, J = 7.32 Hz, 1H, H-4''), 7.48 – 7.57 (t, J = 7.32 Hz, 2H, H-3'', -5''), 7.74 – 7.82 (m, 3H, H-9, -2'', -6''), 7.83 – 7.90 (d, J = 7.32 Hz, 2H, H-2', -6'), 7.90 – 7.98 (d, J = 8.54 Hz, 2H, H-3', -5'), 8.18 – 8.23 (s, 1H, H-4), 8.52 – 8.56 (s, 1H, H-2)

ESI-MS: cal. 425.13, exp. 425.1 ($[M+H]^+$)

HPLC: 97 % purity at 254 nm

t_R (HPLC): 7.61 min; λ_{max} = 253 nm and 301 nm

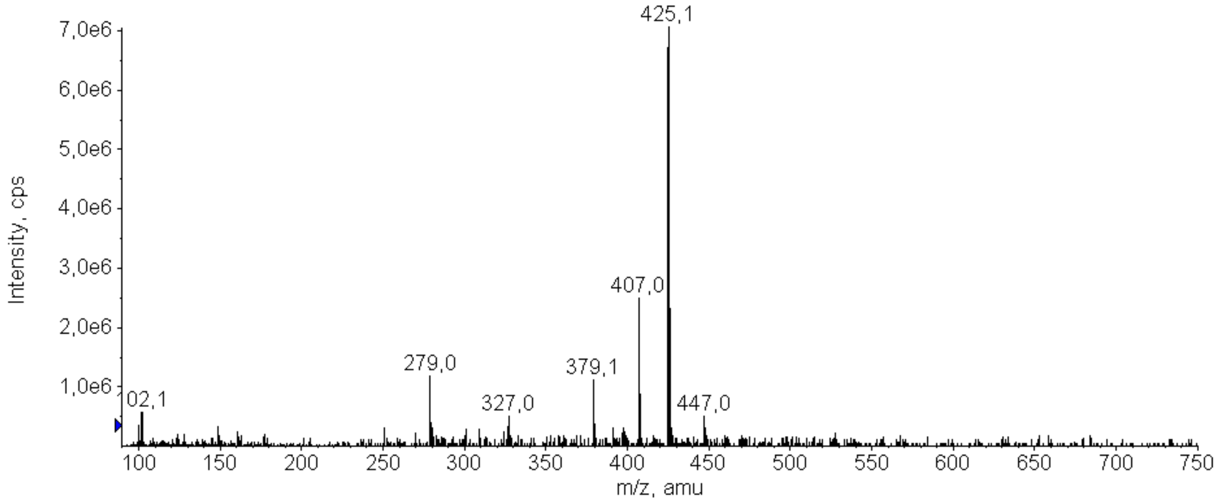

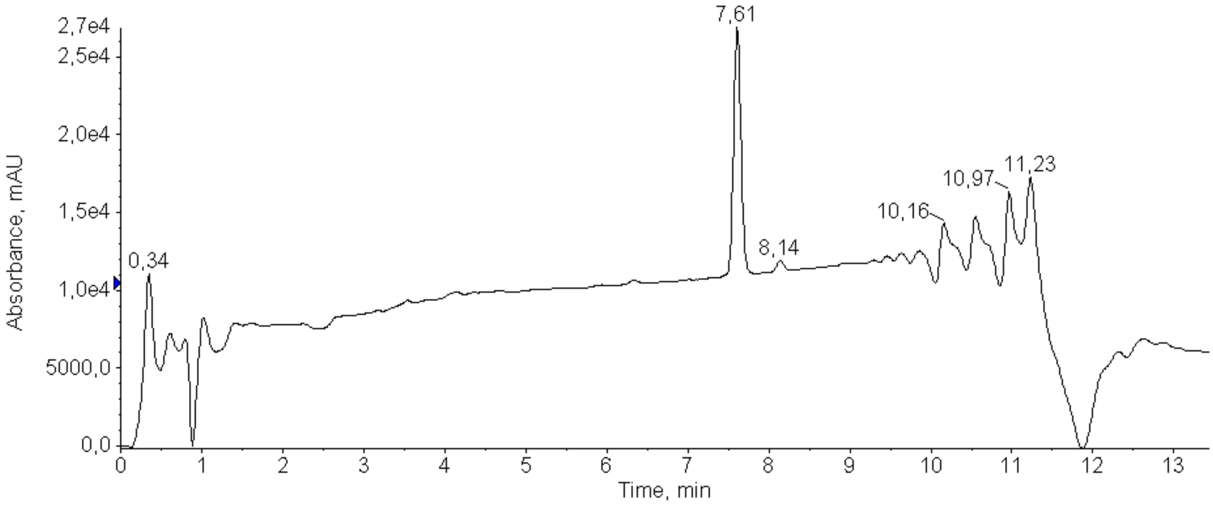

Supporting Information Figure 64. LC/MS analysis of **Scaff10-41**. Upper panel: Mass spectrum. Lower panel: according chromatogram.

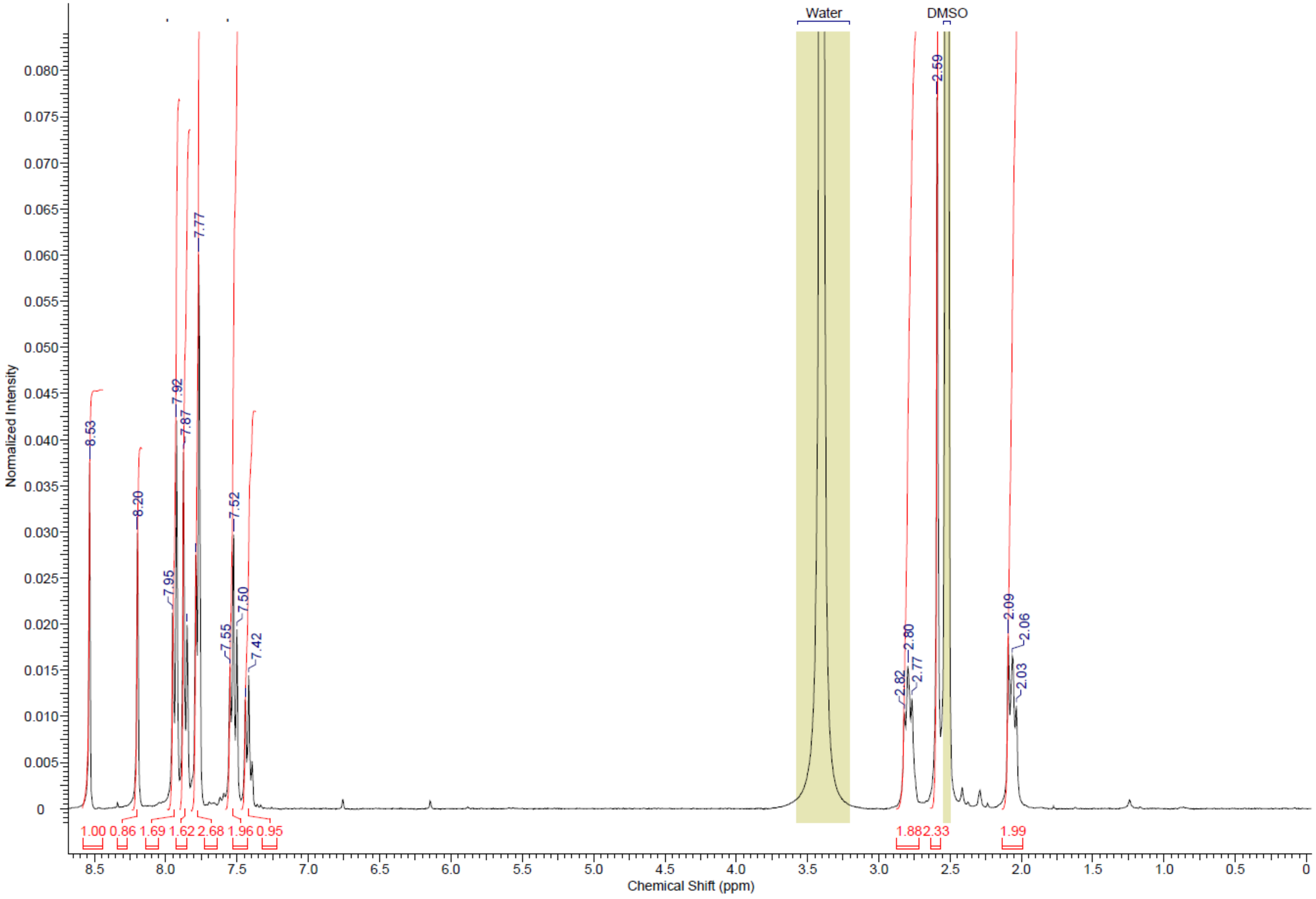

Supporting Information Figure 65: ^{1}H NMR spectrum of **Scaff10-41**, 300 MHz, DMSO-d_6.

4.1.37 Ethyl 3-(7-[2-(6-methoxy-2-naphthyl)-2-oxo-ethoxy]-4-methyl-2-oxo-chromen-3-yl)propanoate (**Scaff10-42**)

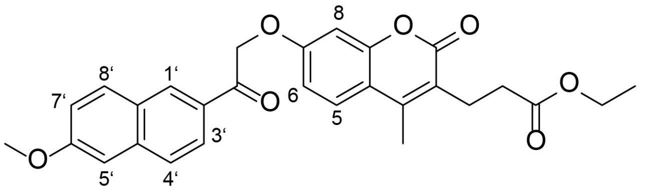

Ethyl 3-(7-hydroxy-4-methyl-2-oxo-chromen-3-yl)propanoate (**5a**) (100 mg, 0.36 mmol) was solved in acetone and preactivated with potash in excess (125 mg, 0.90 mmol) at 55 °C. The α-haloketone 2-bromo-1-(6-methoxy-2-naphthyl)ethanone (**2h**) was added (100 mg, 0.34 mmol). The product was precipitated using 1 M H_2SO_4 and washed with ethanol. The precipitated solid was purified using preparative HPLC. The final product after lyophilisation gave a bright yellow solid.

^{1}H NMR (300 MHz, DMSO-d_6): δ(ppm) = 1.12 – 1.27 (t, J = 7.32 Hz, 3H, -CH_2-C**H_3**), 2.39 – 2.46 (s, 3H, Ar-CH_3), 2.44 – 2.53 (t, J = 7.32 Hz, 2H, -CH_2-C**H_2**-COOEt), 2.77 – 2.88 (t, J = 7.32 Hz, 2H, -C**H_2**-CH_2-COOEt), 3.91 – 4.01 (s, 3H, -OCH_3), 4.10 – 4.12 (q, J = 7.32 Hz, 2H, -C**H_2**-CH_3), 5.80 – 5.92 (s, 2H, -CO-C**H_2**-OAr), 7.04 – 7.15 (m, 2H, H-5', -7'), 7.27 – 7.37 (dd, J = 2.44 Hz, 8.55 Hz, 1H, H-6), 7.44 – 7.50 (s, 1H, H-8), 7.72 – 7.79 (d, J = 9.77 Hz, 1H, H-5), 7.93 – 8.11 (m, 3H, H-3', -4', -8'), 8.70 – 8.76 (s, 1H, H-1')

ESI-MS: cal. 475.17, exp. 475.1 ($[M+H]^+$)

HPLC: 98 % purity at 254 nm

t_R (HPLC): 7.51 min; λ_{max} = 261 nm and 319 nm

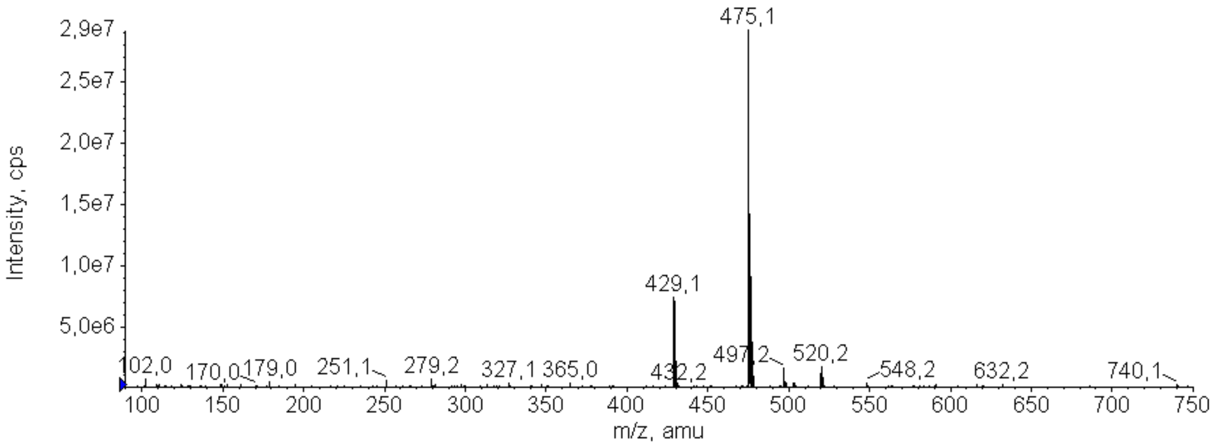

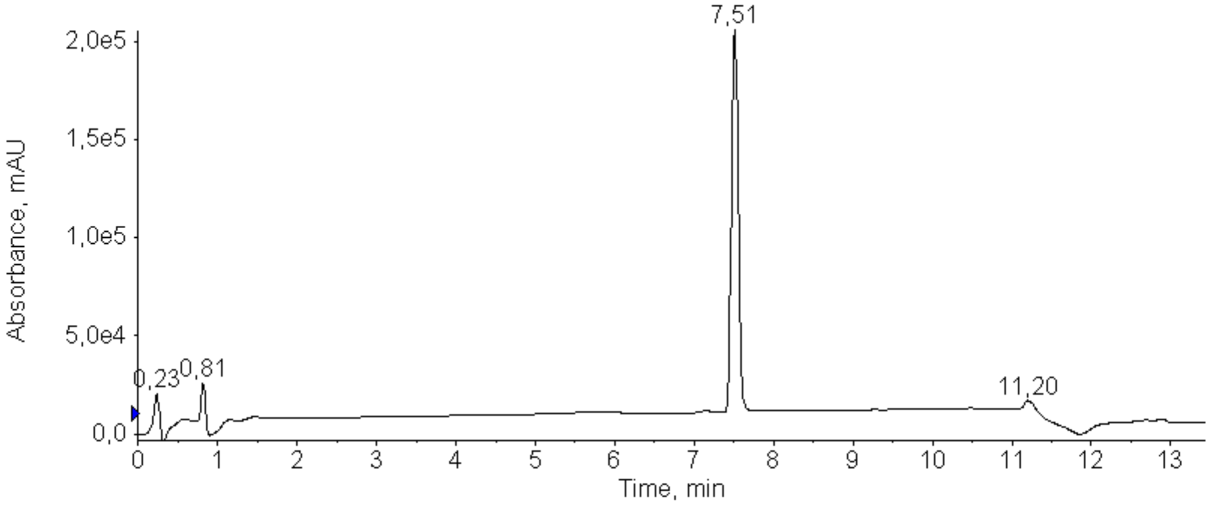

Supporting Information Figure 66: LC/MS analysis of **Scaff10-42**. Upper panel: Mass spectrum. Lower panel: according chromatogram.

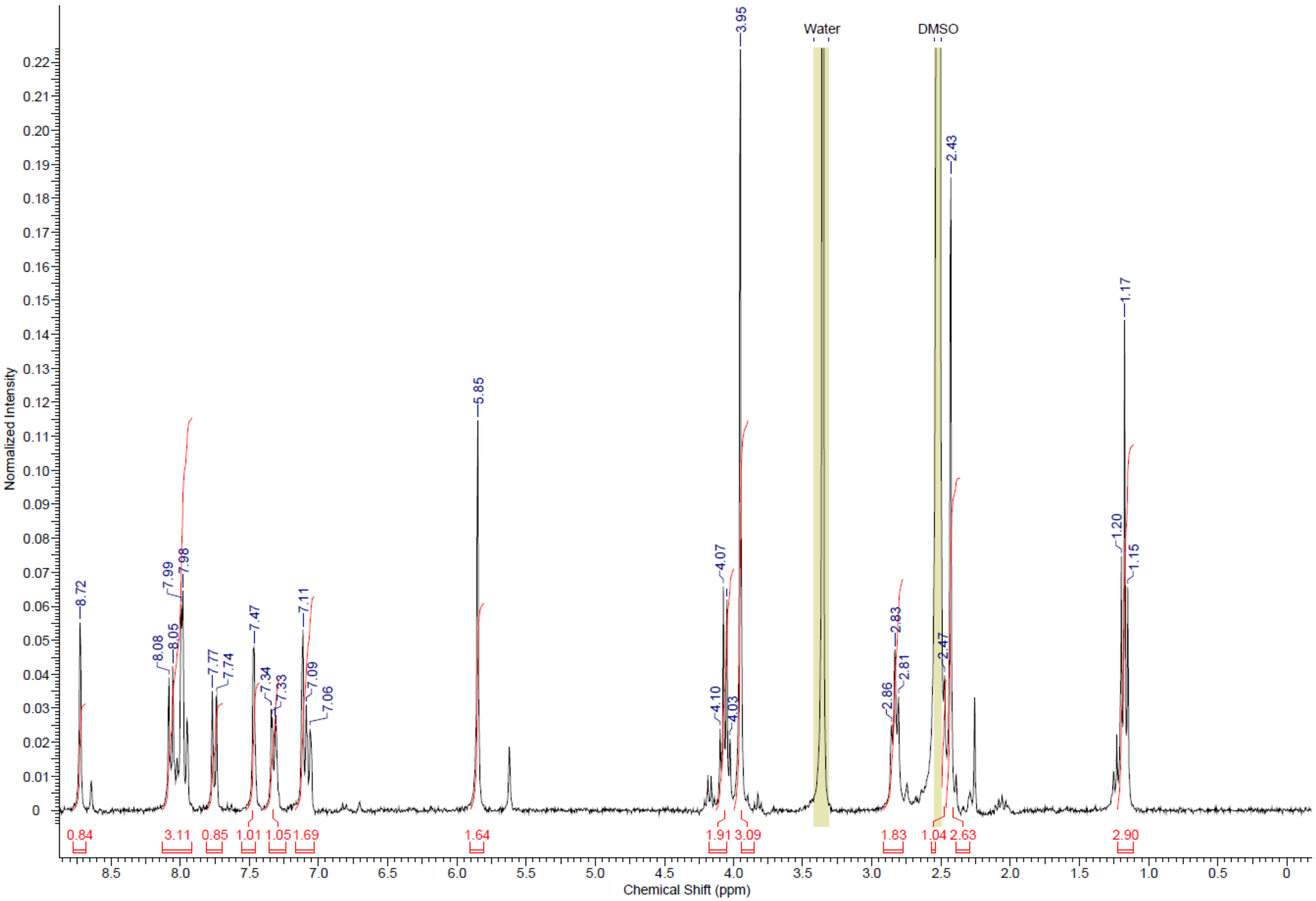

Supporting Information Figure 67: ^{1}H NMR spectrum of **Scaff10-42**, 300 MHz, DMSO-d$_6$.

4.1.38 3-(7-[2-(6-Methoxy-2-naphthyl)-2-oxo-ethoxy]-4-methyl-2-oxo-chromen-3-yl)propanoic acid (**Scaff10-43**)

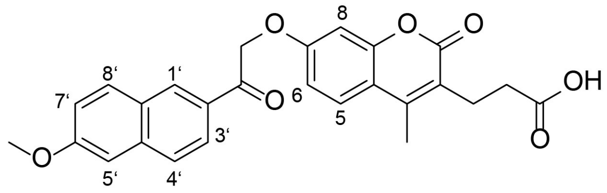

Ethyl 3-(7-[2-(6-methoxy-2-naphthyl)-2-oxo-ethoxy]-4-methyl-2-oxo-chromen-3-yl)propanoate (**Scaff10-42**) (30 mg, 0.06 mmol) was saponificated in a mixture of 1.5 ml DMF and 1.5 ml 1 M sodium hydroxide at 70 °C for 0.5 h. The product was purified using preparative HPLC. The final product after lyophilisation as white solid in 25 % yield (28 mg).

^{1}H NMR (300 MHz, DMSO-d$_6$): δ(ppm) = 1.90 – 2.04 (t, J = 7.33 Hz, 2H, -CH$_2$-C**H$_2$**-COOH), 2.32 – 2.43 (s, 3H, Ar-CH$_3$), 2.61 – 2.74 (t, J = 7.33 Hz, 2H, -C**H$_2$**-CH$_2$-COOH), 3.87 – 3.97 (s, 3H, -OCH$_3$), 5.75 – 5.86 (s, 2H, -CO-C**H$_2$**-OAr), 6.97 – 7.09 (m, 2H, H-6, -8), 7.25 – 7.33 (m, 1H, H-5), 7.41 – 7.47 (s, 1H, H-5'), 7.63 – 7.71 (d, J = 8.55 Hz, 1H, H-7'), 7.90 – 8.08 (m, 3H, H-3', -4', -8'), 8.68 – 8.74 (s, 1H, H-1')

ESI-MS: cal. 447.14, exp. 447.1 ([M+H]$^+$)

HPLC: 99 % purity at 254 nm

t_R (HPLC): 6.25 min; λ_{max} = 261 nm and 320 nm

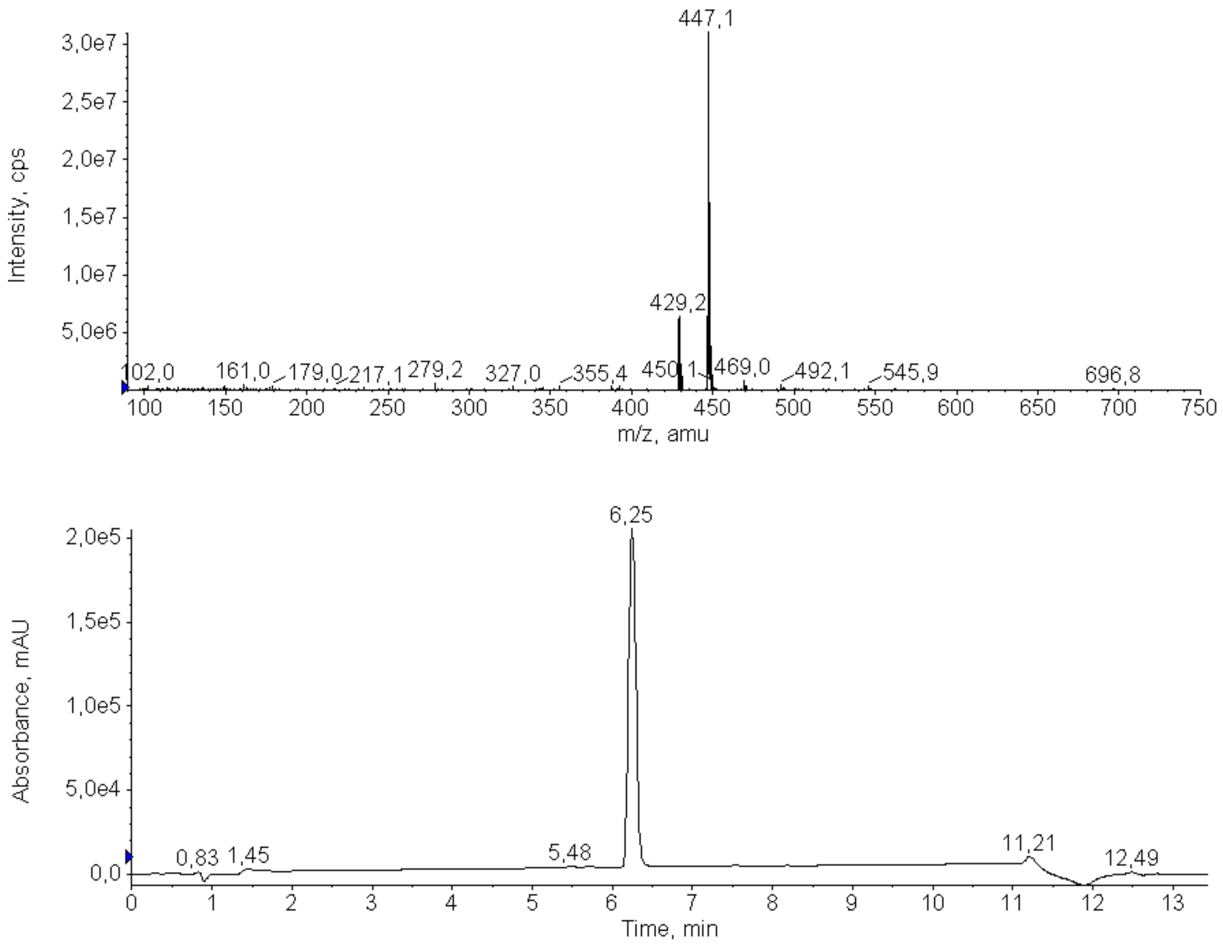

Supporting Information Figure 68: LC/MS analysis of **Scaff10-43**. Upper panel: Mass spectrum. Lower panel: according chromatogram.

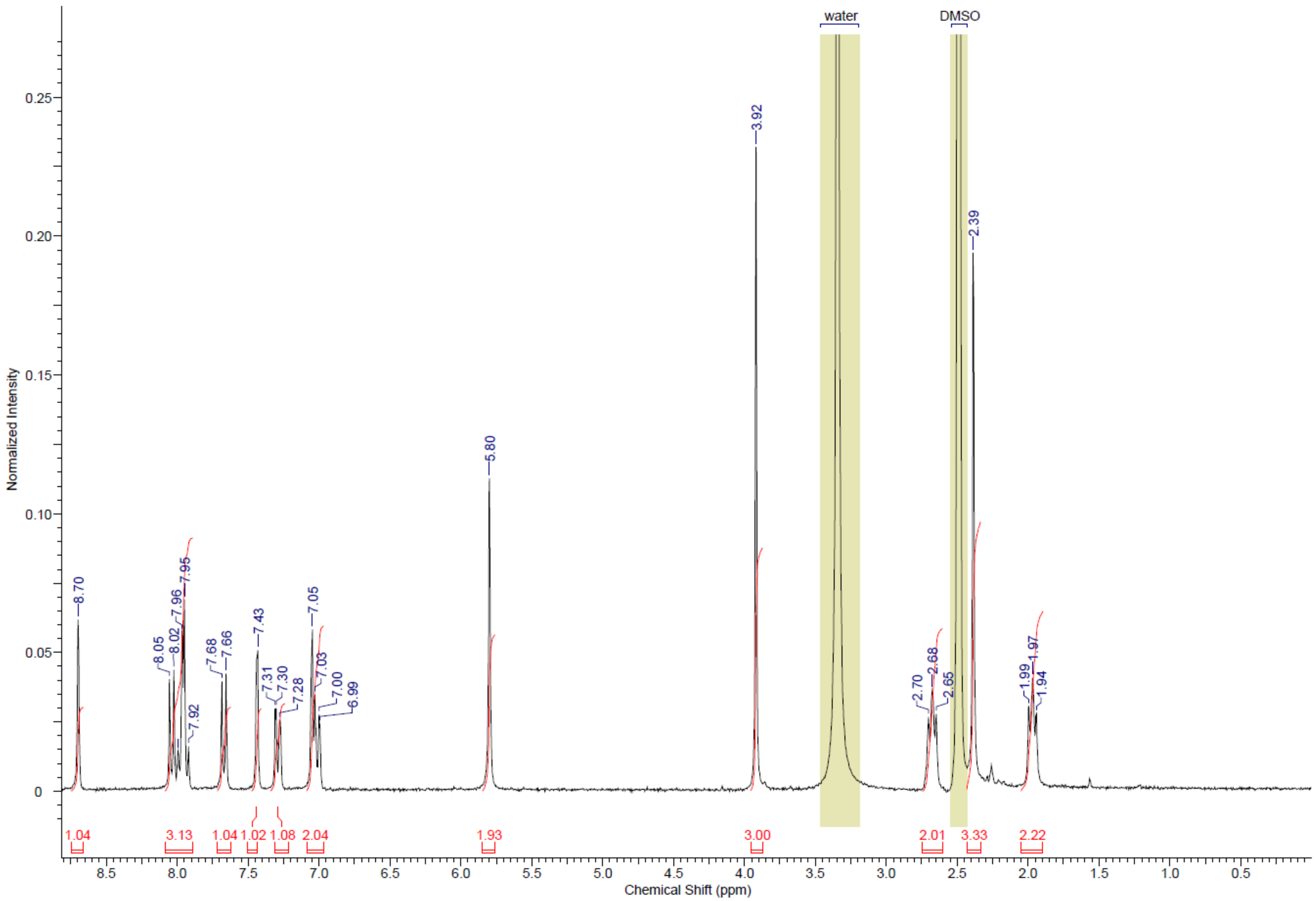

Supporting Information Figure 69: ^{1}H NMR spectrum of **Scaff10-43**, 300 MHz, DMSO-d_6.

4.1.39 3-[7-Methoxy-6-[1-(6-methoxy-2-naphthyl)vinyl]-4-methyl-2-oxo-chromen-3-yl]propanoic acid (**Scaff10-44**)

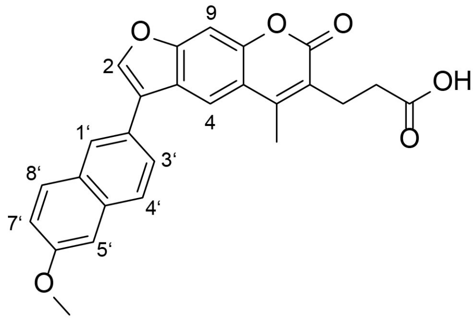

Ethyl 3-(7-[2-(6-methoxy-2-naphthyl)-2-oxo-ethoxy]-4-methyl-2-oxo-chromen-3-yl)propanoate (**Scaff10-42**) (10 mg, 0.02 mmol) was saponificated in a mixture of 0.3 ml DMF and 0.5 ml 2 M sodium hydroxide at 80 °C for 0.75 h. The final product after purificationgave a yellowish solid in 51 % yield (4 mg).

^{1}H NMR (300 MHz, DMSO-d$_6$): δ(ppm) = 1.95 – 2.07 (t, J = 8.55 Hz, 2H, -CH$_2$-C**H$_2$**-COOH), 2.54 – 2.62 (s, 3H, Ar-CH$_3$), 2.69 – 2.82 (t, J = 8.55 Hz, 2H, -C**H$_2$**-CH$_2$-COOH), 3.86 – 3.94 (s, 3H, -OCH$_3$), 7.19 – 7.25 (dd, J = 2.44 Hz, 9.77 Hz, 1H, H-7'), 7.36 – 7.40 (d, J = 2.44 Hz, 1H, H-5'), 7.73 – 7.76 (s, 1H, H-9'), 7.83 – 7.89 (d, J = 9.77 Hz, 1H, H-8'), 7.93 – 8.02 (m, 2H, H-3', -1'), 8.21 – 8.32 (m, 2H, H-4, -4'), 8.50 – 8.53 (s, 1H, H-2)

ESI-MS: cal. 429.13, exp. 429.1 ([M+H]$^+$)

HPLC: 96 % purity at 254 nm

t_R (HPLC): 7.15 min; λ_{max} = 308 nm

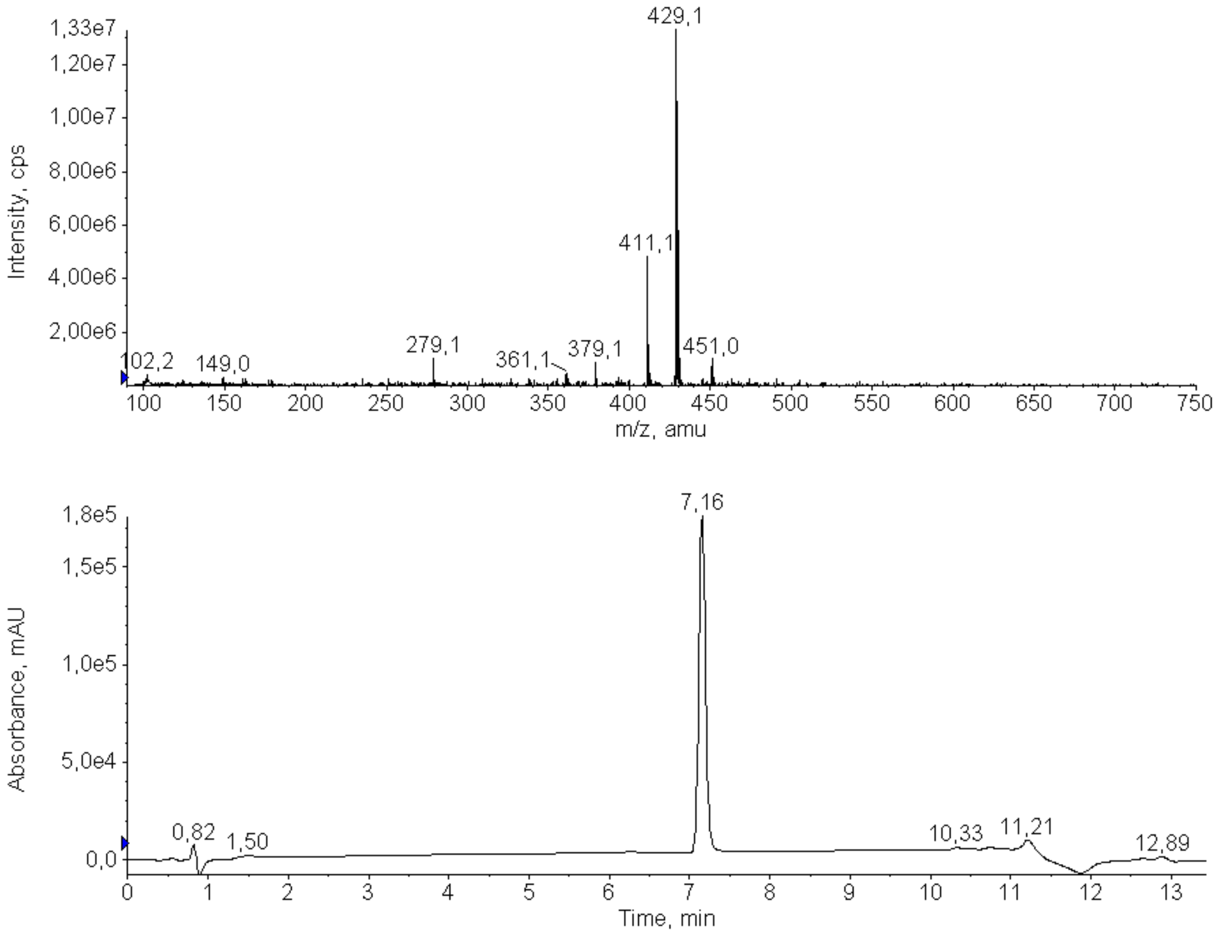

Supporting Information Figure 70: LC/MS analysis of **Scaff10-44**. Upper panel: Mass spectrum. Lower panel: according chromatogram.

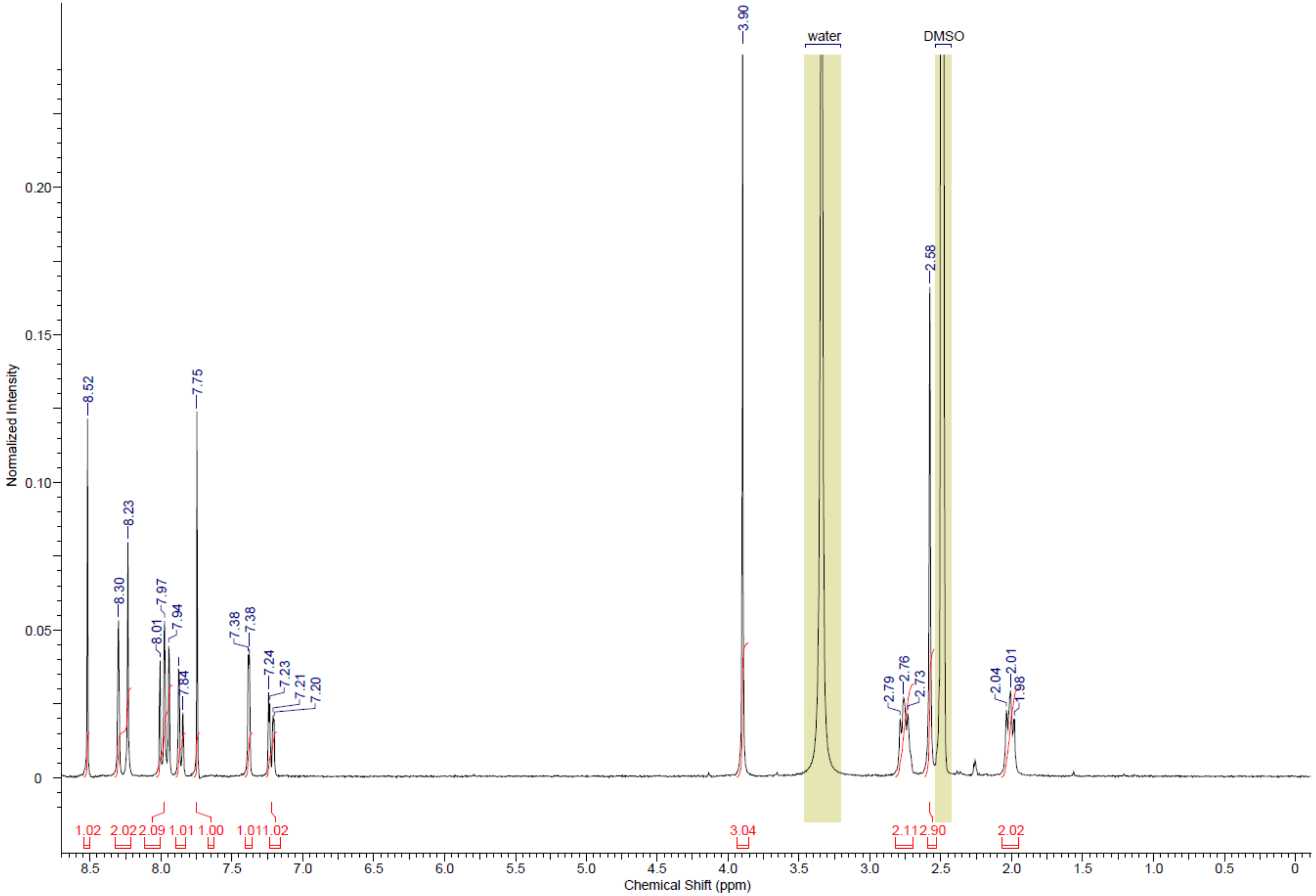

Supporting Information Figure 71: ^{1}H NMR spectrum of **Scaff10-44**, 300 MHz, DMSO-d_6.

4.1.40 3-[3-[6-(4-Fluorophenyl)-2-methyl-3-pyridyl]-5-methyl-7-oxo-furo[3,2-*g*]chromen-6-yl]propanoic acid (**Scaff10-45**)

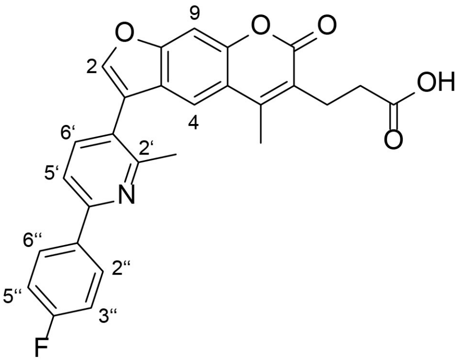

3-[7-[2-[6-(4-Fluorophenyl)-2-methyl-3-pyridyl]-2-oxo-ethoxy]-4-methyl-2-oxo-chromen-3-yl]propanoate (**Scaff10-10**) (170 mg, 0.34 mmol) was saponificated in a mixture of 7 ml dry acetone and 7 ml 1 M sodium hydroxide at 95 °C for 6.5 h. Dropwise addition of 1 M HCl resulted in precipitation of the product. The precipitated solid was filtered and recrystallised in ethanol. The final product gave an ivory white solid in 24 % yield (35 mg).

^{1}H NMR (300 MHz, DMSO-d$_6$): δ(ppm) = 1.90 – 2.11 (m, 2H, -CH$_2$-C**H$_2$**-COOH), 2.45 – 2.53 (s, 3H, -C2'-CH$_3$), 2.59 – 2.69 (s, 3H, Ar-CH$_3$), 2.69 – 2.91 (m, 2H, -C**H$_2$**-CH$_2$-COOH), 7.28 – 7.46 (t, J = 8.54 Hz, 2H, H-3'', -5''), 7.74 – 7.82 (s, 1H, H-9), 7.83 – 7.91 (s, 1H, H-4), 7.91 – 8.07 (m, 2H, H-5', -6'), 8.16 – 8.33 (m, 2H, H-2'', -6''), 8.33 – 8.45 (s, 1H, H-2)

ESI-MS: cal. 458.14, exp. 458.1 ([M+H]$^+$)

HPLC: 99 % purity at 254 nm

t_R (HPLC): 6.24 min; λ_{max} = 246 nm and 300 nm

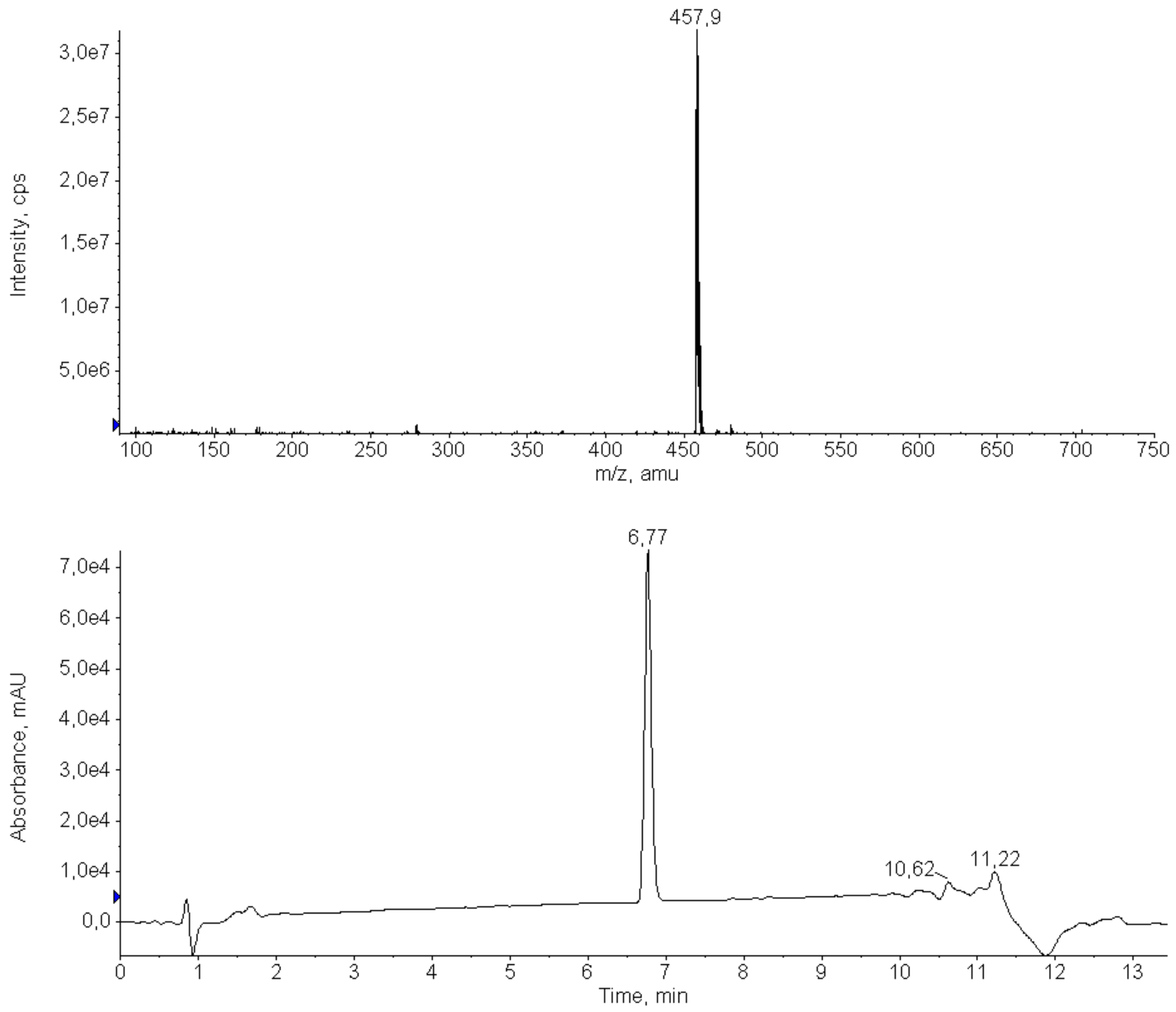

Supporting Information Figure 72: LC/MS analysis of **Scaff10-45**. Upper panel: Mass spectrum. Lower panel: according chromatogram.

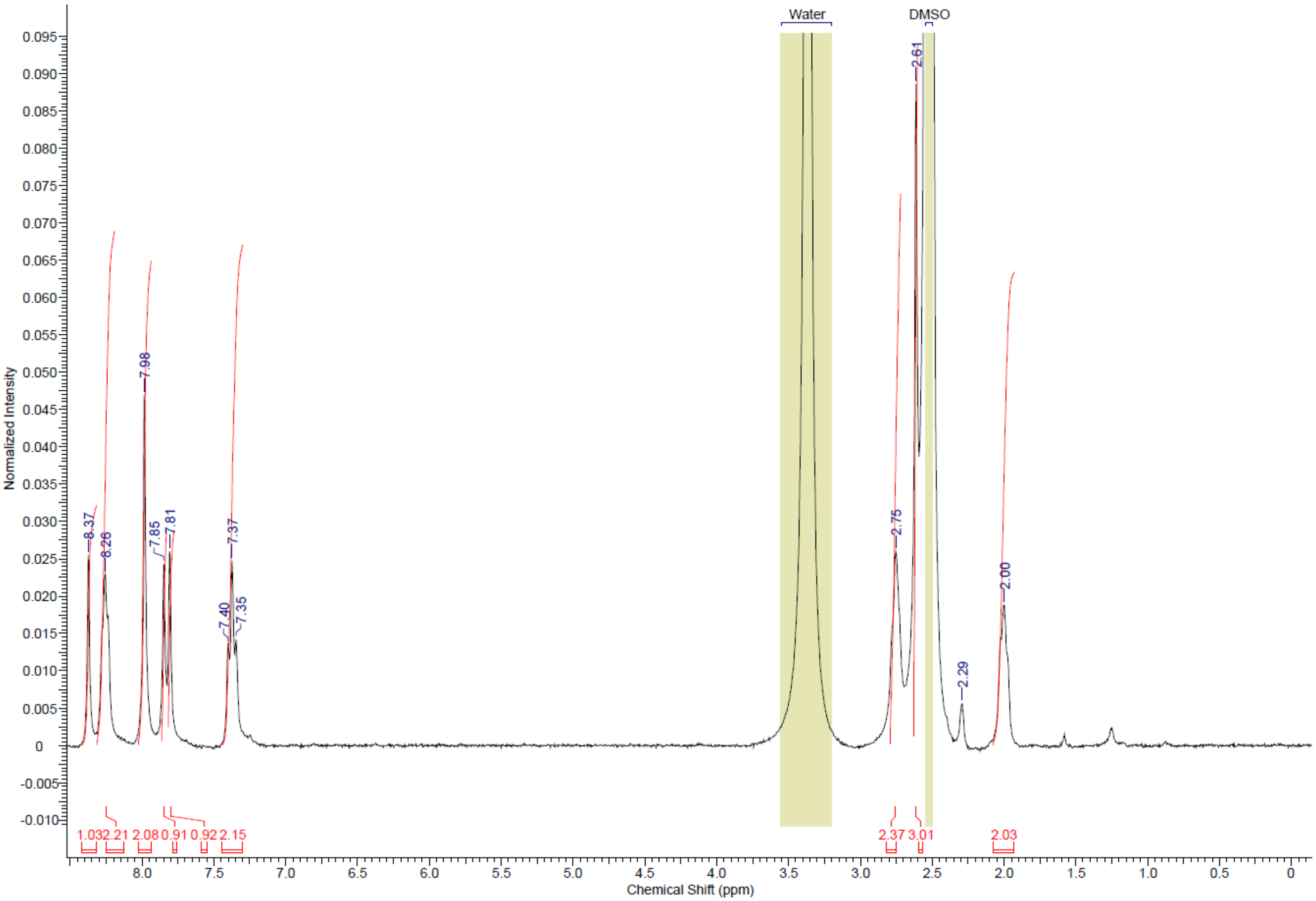

Supporting Information Figure 73: ^{1}H NMR spectrum of **Scaff10-45**, 300 MHz, DMSO-d_6.

4.1.41 5,6-Dimethyl-3-(4-phenylphenyl)furo[3,2-*g*]chromen-7-one (**Scaff10-46**)

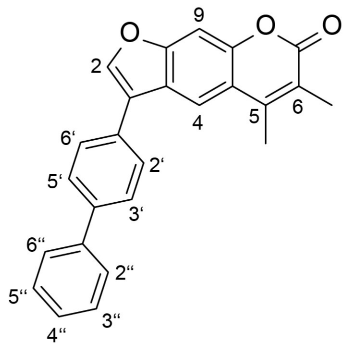

3,4-Dimethyl-7-[2-oxo-2-(4-phenylphenyl)ethoxy]chromen-2-one (**Scaff10-13**) (50 mg, 0.13 mmol) was saponificated in a mixture of 5 ml ethanol, 5 ml isopropyl alcohol and 2 ml 1 M sodium hydroxide at 70 °C for 3 h. Crystals precipitated at 4 °C overnight. The final product gave yellowish crystals in 25 % yield (12 mg).

^{1}H NMR (300 MHz, DMSO-d_6): δ(ppm) = 2.10 – 2.16 (s, 3H, -C6-CH_3), 2.55 – 2.67 (s, 3H, -C5-CH_3), 7.37 – 7.47 (t, J = 7.32 Hz, 1H, H-4''), 7.47 – 7.59 (t, J = 7.32 Hz, 2H, H-3'', -5''), 7.71 – 7-83 (m, 3H, H-9, -2'', -6''), 7.83 – 7.90 (d, J = 7.32 Hz, 2H, H-2', -6'), 7.90 – 7.99 (d, J = 7.32 Hz, 2H, H-3', -5'), 8.18 – 8.26 (s, 1H, H-4), 8.51 – 8.58 (s, 1H, H-2)

ESI-MS: cal. 367.13, exp. 367.1 ($[M+H]^+$)

HPLC: 99 % purity at 254 nm

t_R (HPLC): 8.94 min; λ_{max} = 263 nm and 306 nm

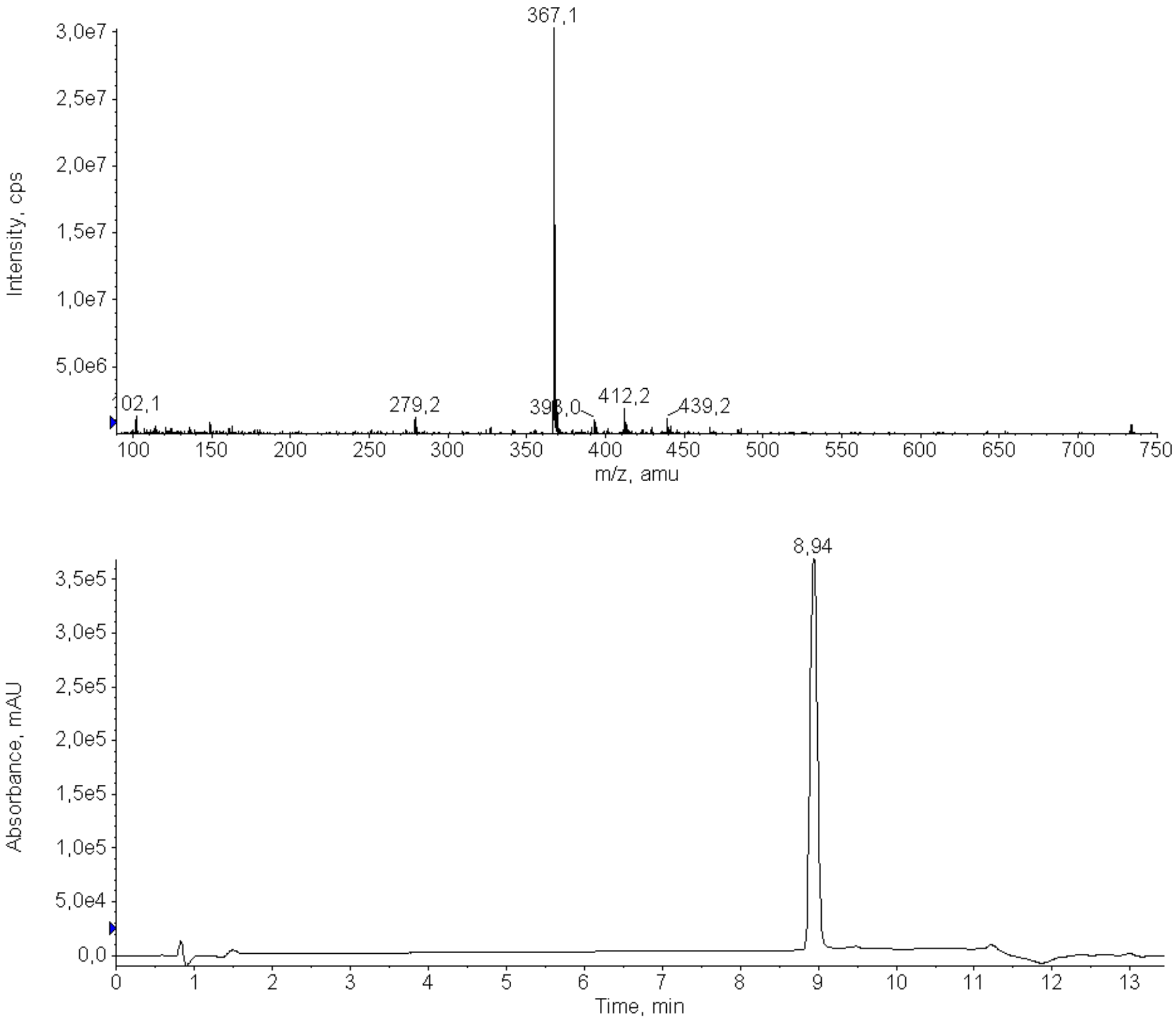

Supporting Information Figure 74: LC/MS analysis of **Scaff10-46**. Upper panel: Mass spectrum. Lower panel: according chromatogram.

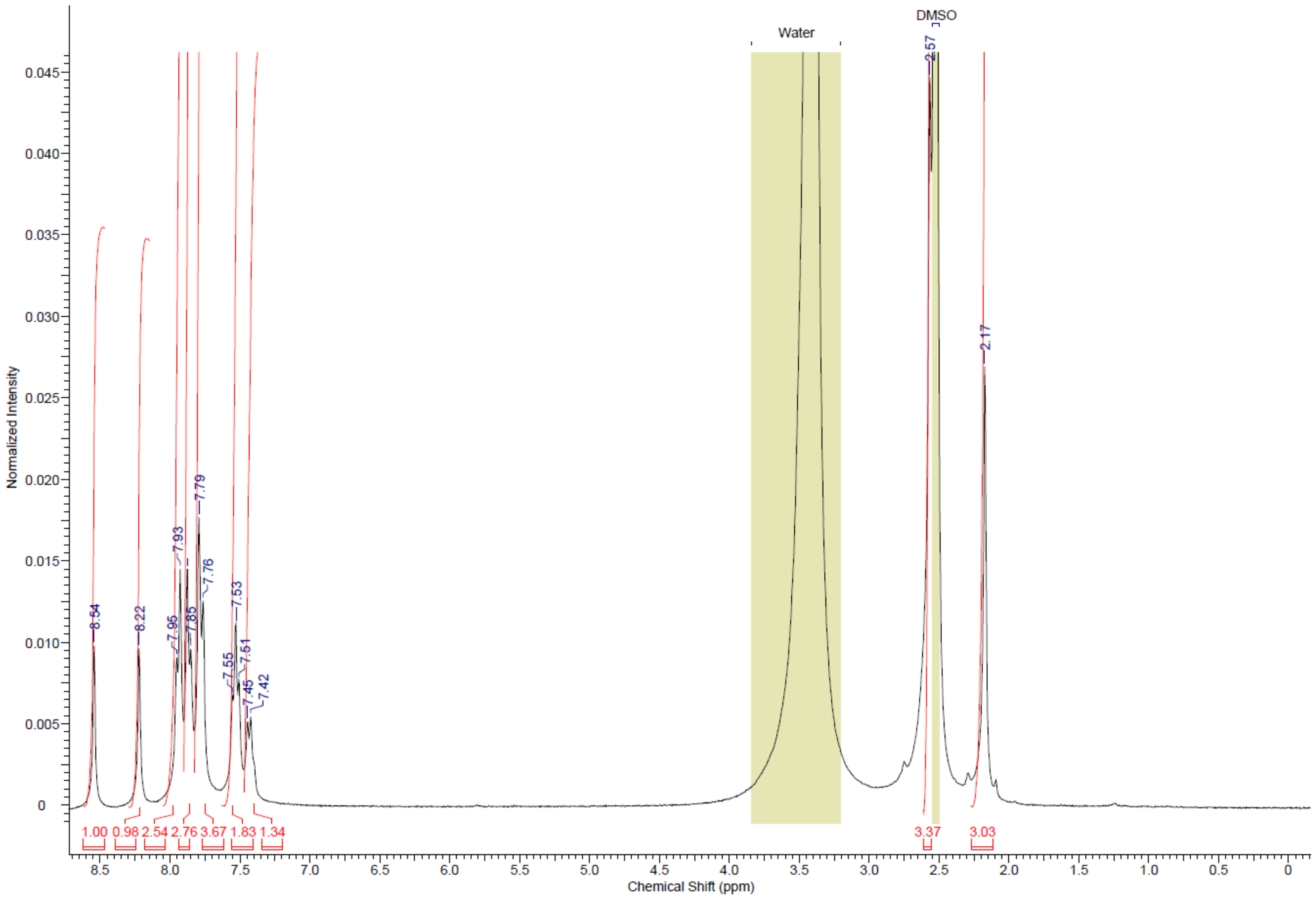

Supporting Information Figure 75: ^{1}H NMR spectrum of **Scaff10-46**, 300 MHz, DMSO-d_6.

4.1.42 3-(6-Methoxy-2-naphthyl)-5,6-dimethyl-furo[3,2-*g*]chromen-7-one (**Scaff10-47**)

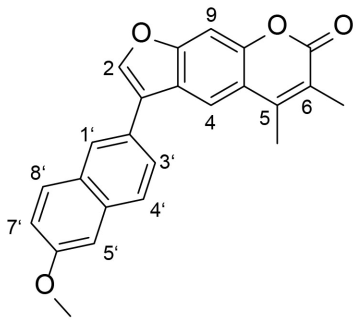

7-[2-(6-Methoxy-2-naphthyl)-2-oxo-ethoxy]-3,4-dimethyl-chromen-2-one (**Scaff10-15**) (5 mg, 0.01 mmol) was saponificated in a mixture of 0.5 ml DMSO and 0.5 ml 2 M sodium hydroxide at 70 °C. The final product after purification gave yellowish crystals.

^{1}H NMR (300 MHz, DMSO-d_6): δ(ppm) = 2.12 – 2.20 (s, 3H, -C6-CH_3), 2.53 – 2.59 (s, 3H, -C5-CH_3), 3.87 – 3.94 (s, 3H, -OCH_3), 7.19 – 7.26 (dd, J = 2.44 Hz, 8.55 Hz, 1H, H-7'), 7.37 – 7.40 (d, J = 2.44 Hz, 1H, H-5'), 7.76 – 7-80 (s, 1H, H-9), 7.84 – 7.90 (d, J = 8.55 Hz, 1H, H-8'), 7.93 – 8.03 (m, 2H, H-1', -3'), 8.25 – 8.33 (m, 2H, H-4, -4'), 8.52 – 8.55 (s, 1H, H-2)

ESI-MS: cal. 371.12, exp. 371.1 ($[M+H]^+$)

HPLC: 99 % purity at 254 nm

t_R (HPLC): 8.46 min; λ_{max} = 307 nm

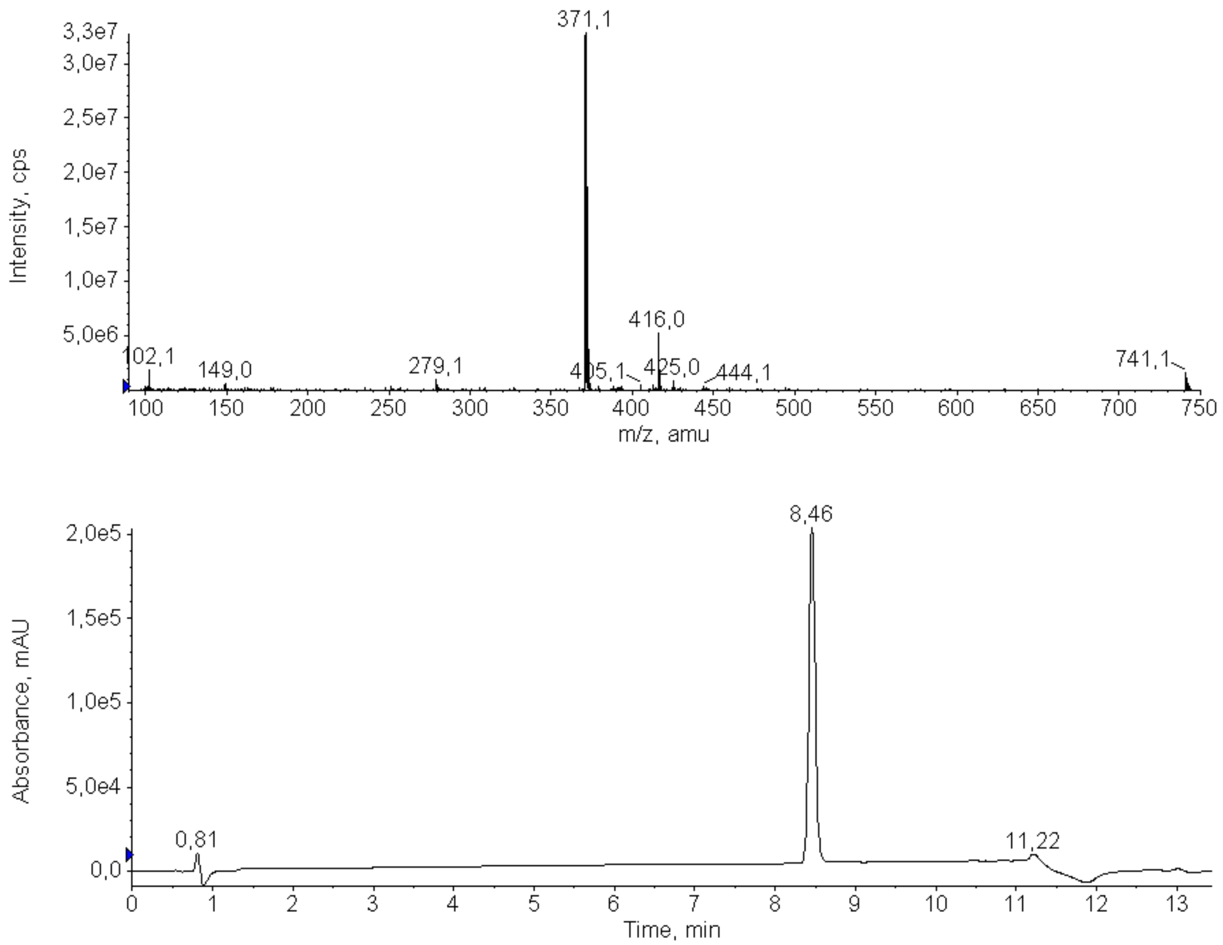

Supporting Information Figure 76: LC/MS analysis of **Scaff10-47**. Upper panel: Mass spectrum. Lower panel: according chromatogram.

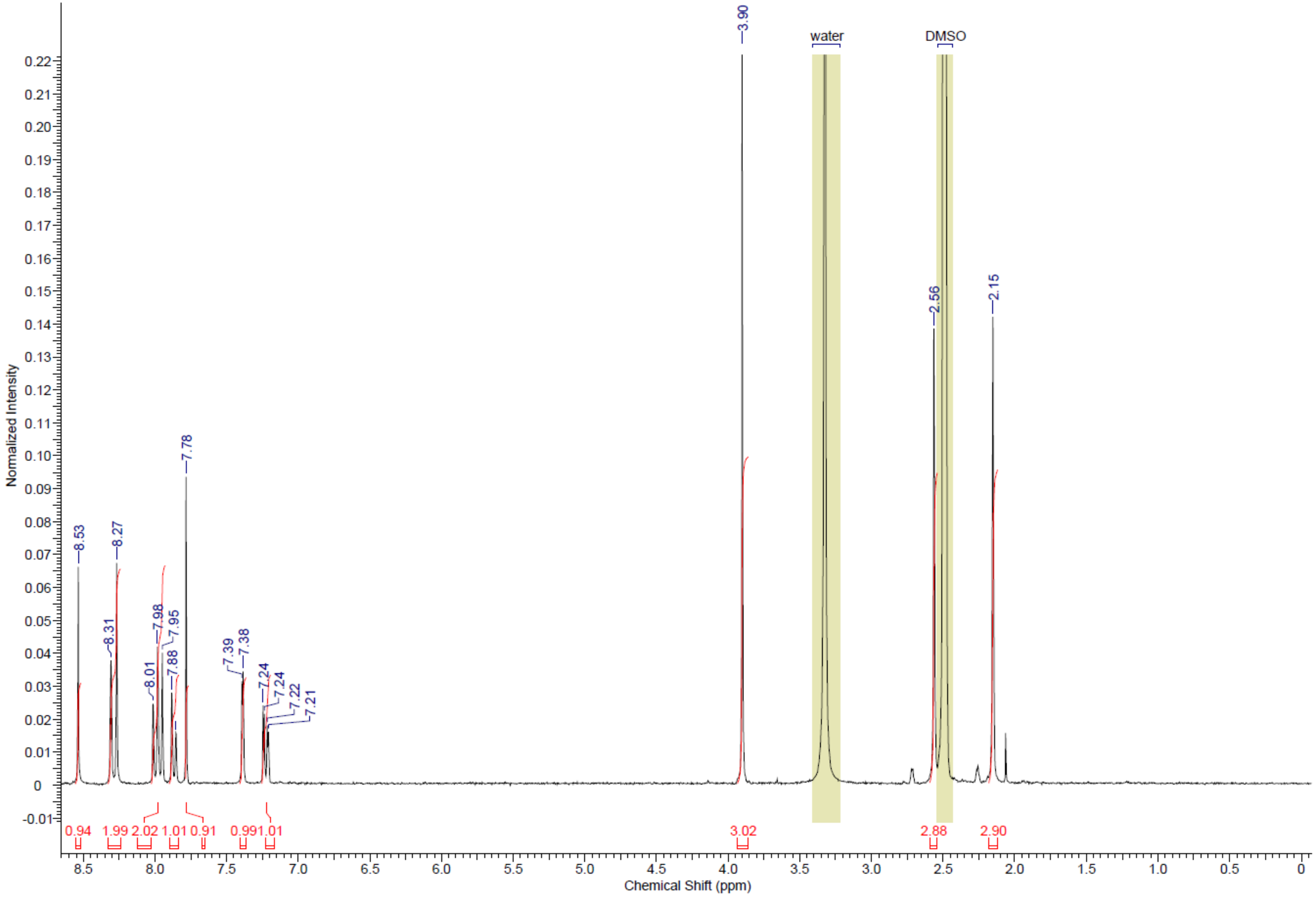

Supporting Information Figure 77: ^{1}H NMR spectrum of **Scaff10-47**, 300 MHz, DMSO-d_6.

4.1.43 Methyl 3-[3-(4-methoxyphenyl)-5-methyl-7-oxo-furo[3,2-*g*]chromen-6-yl]propanoate (**Scaff10-48**)

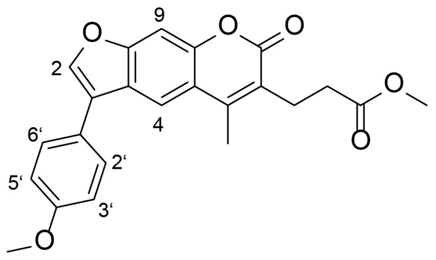

3-[3-(4-Methoxyphenyl)-5-methyl-7-oxo-furo[3,2-*g*]chromen-6-yl]propanoic acid (**Scaff10-8**) (38 mg, 0.10 mmol) was esterified in a mixture of 2 ml methanol and 0.5 ml 1 M H_2SO_4 at 80 °C for 2.5 h. The precipitated solid was recrystallised in ethanol. The final product gave an orange-yellowish solid in 15 % yield (6 mg).

^{1}H NMR (300 MHz, DMSO-d_6): δ(ppm) = 2.49 - 2.55 (t, J = 7.32 Hz, 2H, -CH_2-C**H₂**-$COOCH_3$), 2.54 – 2.59 (s, 3H, Ar-CH_3), 2.84 – 2.94 (t, J = 7.32 Hz, 2H, -C**H₂**-CH_2-$COOCH_3$), 3.55 – 3.64 (s, 3H, -COOC**H₃**), 3.80 – 3.86 (s, 3H, -OCH_3), 7.06 - 7.14 (d, J = 8.54 Hz, 2H, H-3’, -5’), 7.71 – 7.79 (m, 3H, H-7, -2’, -6’), 8.11 – 8.17 (s, 1H, H-4), 8.35 – 8.40 (s, 1H, H-2)

ESI-MS: cal. 393.13, exp. 393.1 ($[M+H]^+$)

HPLC: 98 % purity at 254 nm

t_R (HPLC): 7.20 min; λ_{max} = 248 nm and 306 nm

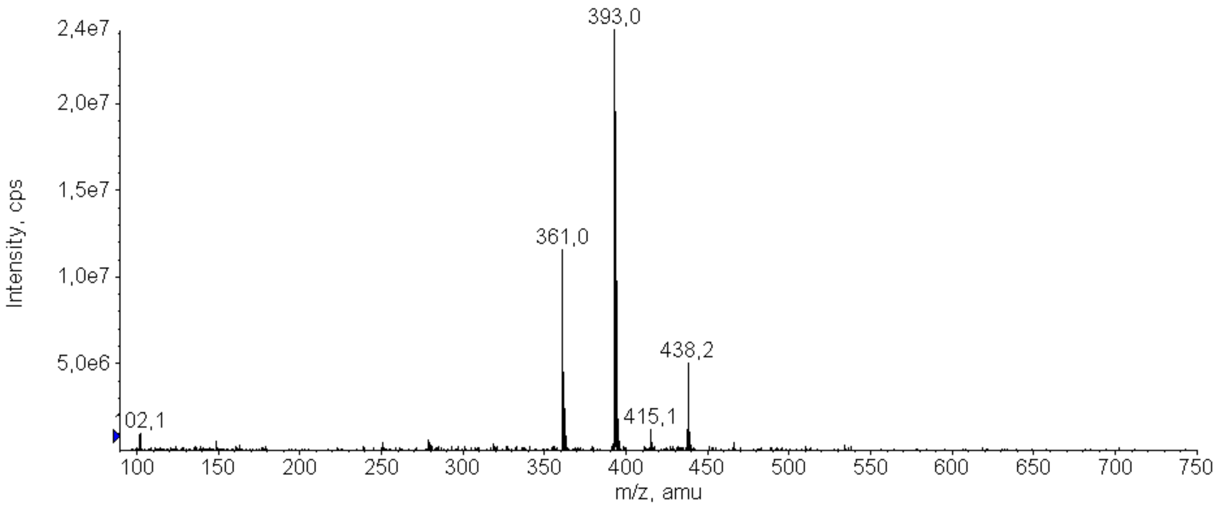

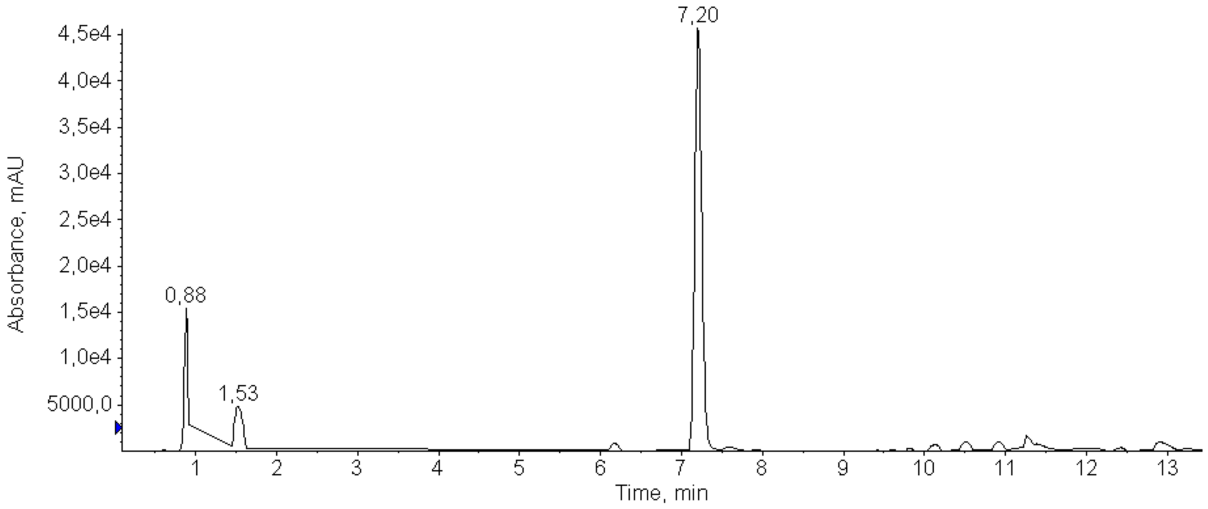

Supporting Information Figure 78: LC/MS analysis of **Scaff10-48**. Upper panel: Mass spectrum. Lower panel: according chromatogram.

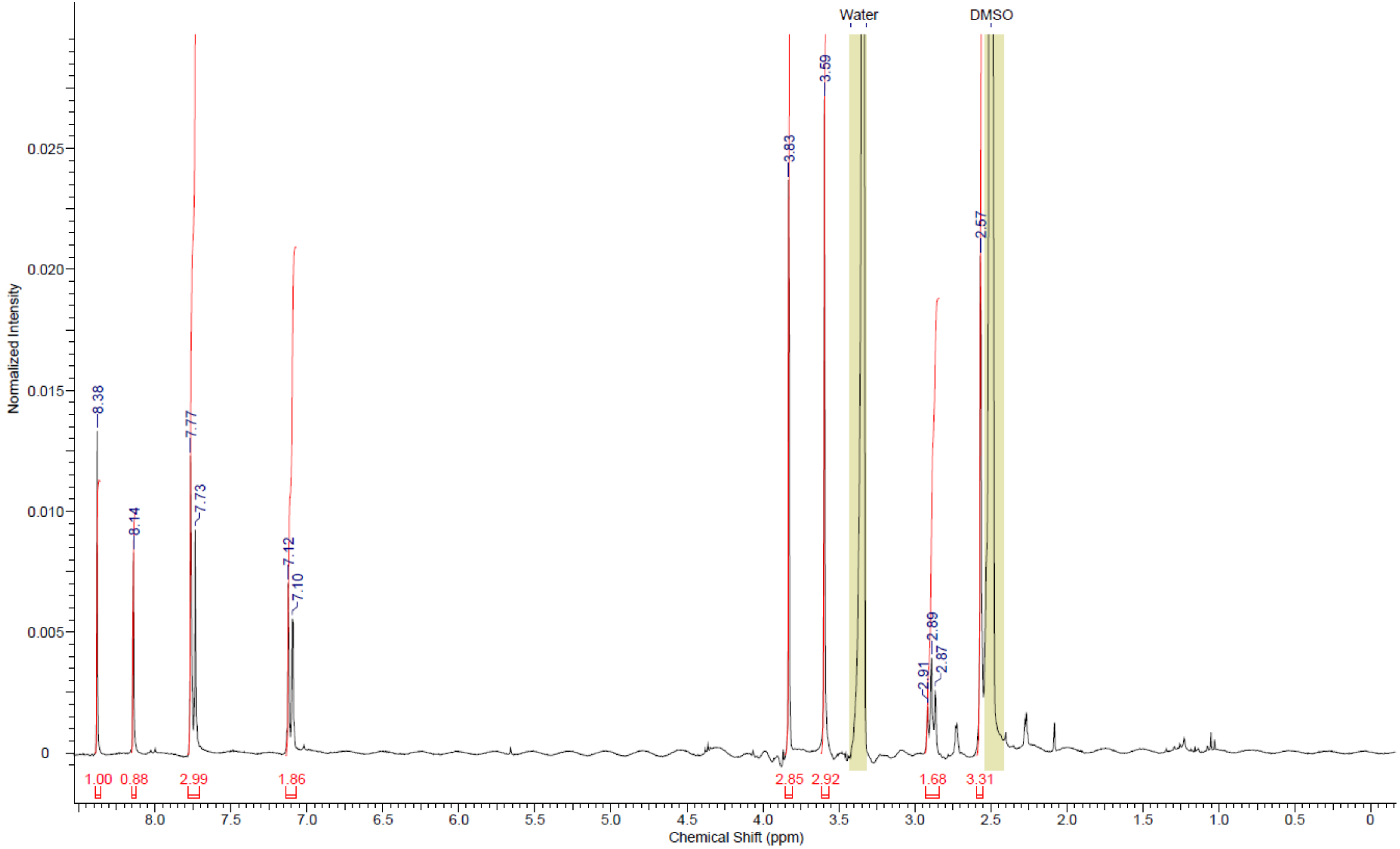

Supporting Information Figure 79: ^{1}H NMR spectrum of **Scaff10-48**, 300 MHz, DMSO-d_6.

4.1.44 3-[3-(4-Methoxyphenyl)-5-methyl-7-oxo-furo[3,2-*g*]chromen-6yl]propanehydroxamic acid (**Scaff10-51**)

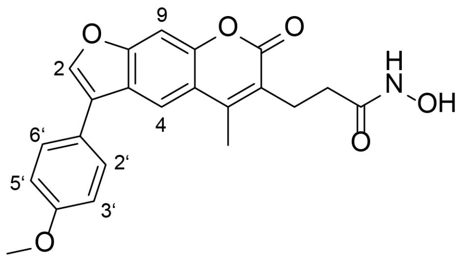

3-[3-(4-Methoxyphenyl)-5-methyl-7-oxo-furo[3,2-*g*]chromen-6-yl]propanoic acid (**Scaff10-8**) (60 mg, 0.16 mmol) was solved in 3 ml DMF and incubated with *N*,*N*,*N'*,*N'*-tetramethylfluoroformamidinium hexafluorophosphate (TFFH) (63 mg, 0.24 mmol) and TEA (48 mg, 0.48 mmol) for 5 min at 25 °C. A mixture of hydroxylamine (33 mg, 0.48 mmol), TEA (48 mg, 0.48 mmol) and 0.5 ml water was added. After 16 h at 25 °C, the precipitate was purified using preparative HPLC with the following gradient: 10 % to 90 % ACN in H_2O in 80 min. The product eluted at t_R = 47 min and was obtained after lyophilisation. An detection for hydroxamic acids was performed using iron(III)chloride in a solution of water and ethanol (1:1). Addition of Scaff10-51 resulted in the characteristic red colour of the complex formed by iron(III) and hydroxamic acids. The final product gave a yellowish solid in 6 % yield (3 mg).

^{1}H NMR (300 MHz, DMSO-d_6): δ(ppm) = 2.13 - 2.25 (t, J = 7.32 Hz, 2H, -CH_2-C**H₂**-CONHOH), 2.53 – 2.59 (s, 3H, Ar-CH_3), 2.77 – 2.91 (t, J = 7.32 Hz, 2H, -C**H₂**-CH_2-CONHOH), 3.78 – 3.89 (s, 3H, -OCH_3), 7.07 - 7.17 (d, J = 8.54 Hz, 2H, H-3', -5'), 7.70 – 7.80 (m, 3H, H-9, -2', -6'), 8.11 – 8.17 (s, 1H, H-4), 8.36 – 8.40 (s, 1H, H-2), 8.68 – 8.74 (s, 1H, -N**H**-OH), 10.41 – 10.46 (s, 1H, -NH-O**H**)

ESI-MS: cal. 394.12, exp. 394.1 ($[M+H]^+$)

HPLC: 98 % purity at 254 nm

t_R (HPLC): 5.42 min; λ_{max} = 250 nm and 307 nm

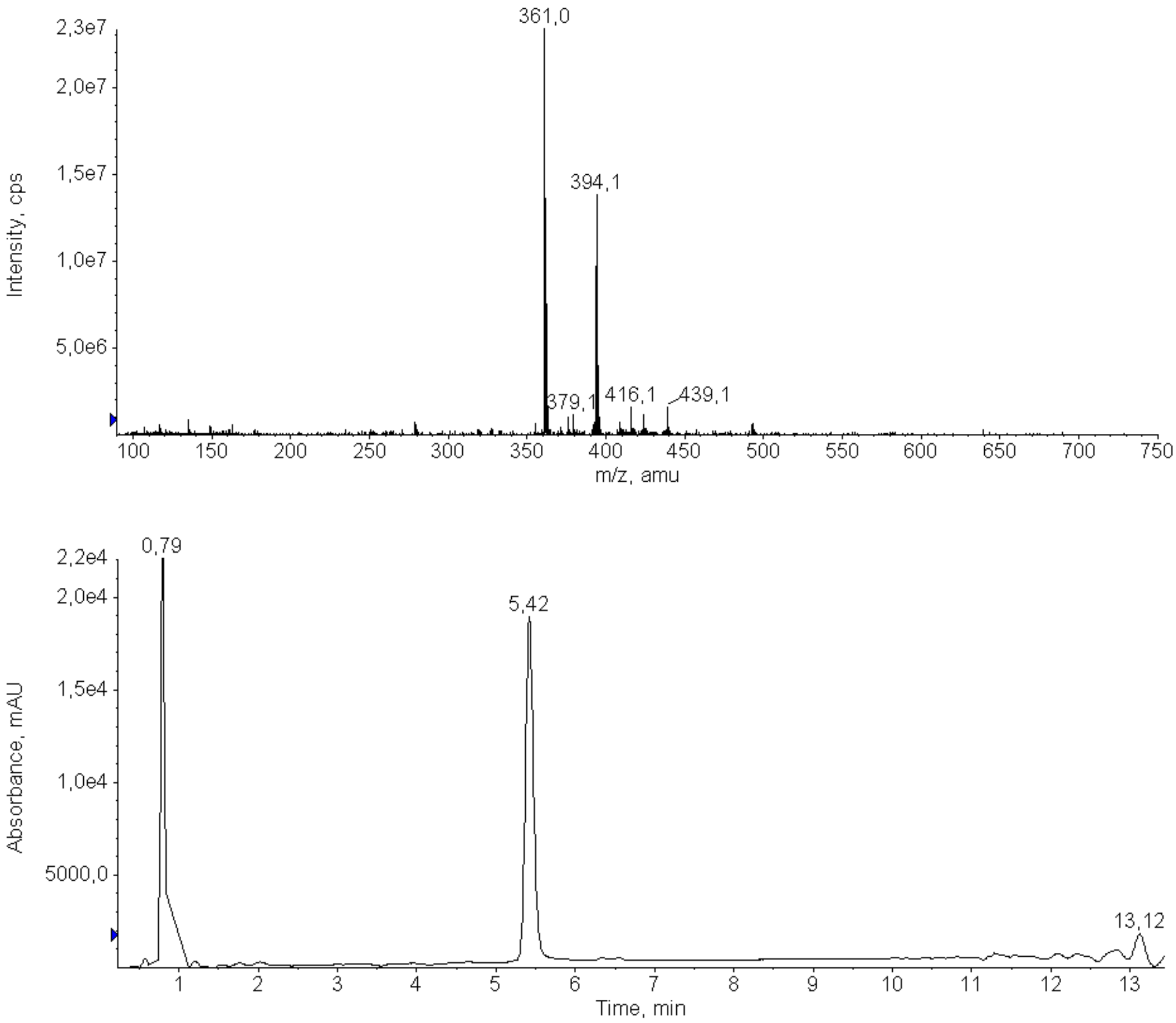

Supporting Information Figure 80: LC/MS analysis of **Scaff10-51**. Upper panel: Mass spectrum. Lower panel: according chromatogram.

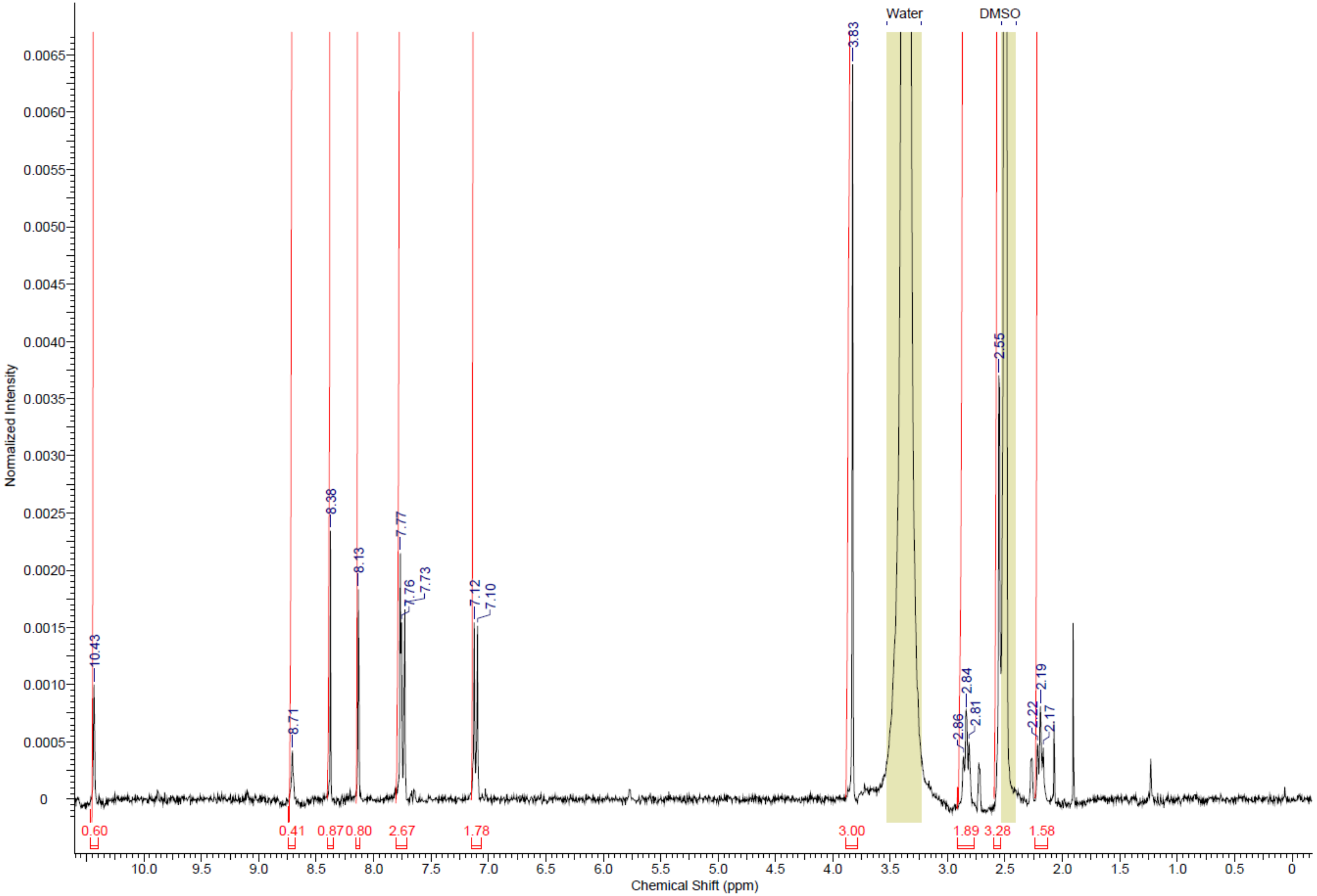

Supporting Information Figure 81: ^{1}H NMR spectrum of **Scaff10-51**, 300 MHz, DMSO-d_6.

4.1.45 Ethyl 3-[7-[2-(4-hydroxyphenyl)-2-oxo-ethoxy]-4-methyl-2-oxo-chromen-3-yl]propanoate (**Scaff10-52**)

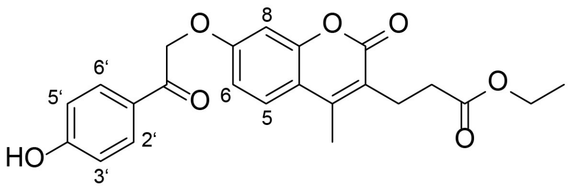

Ethyl 3-(7-hydroxy-4-methyl-2-oxo-chromen-3-yl)propanoate (**5a**) (50 mg, 0.18 mmol) was solved in acetone and preactivated with potash in excess (62 mg, 0.45 mmol) by 55 °C. The α-haloketone 2-bromo-1-(4-hydroxyphenyl)ethanone (**2l**) was added (39 mg, 0.18 mmol). The mixture was refluxed at the same temperature for 4 h. The products Scaff10-52 and Scaff10-52d were purified using preparative HPLC. The final product after purification gave a yellowish solid.

^{1}H NMR (300 MHz, DMSO-d$_6$): δ(ppm) = 1.09 – 1.19 (t, J = 7.33 Hz, 3H, -CH$_2$-C**H$_3$**), 2.34 – 2.41 (s, 3H, Ar-CH$_3$), 2.41 – 2.50 (t, J = 7.33 Hz, 2H, -CH$_2$-C**H$_2$**-COOEt), 2.74 – 2.84 (t, J = 7.33 Hz, 2H, -C**H$_2$**-CH$_2$-COOEt), 3.96 – 4.08 (q, J = 7.33 Hz, 2H, -C**H$_2$**-CH$_3$), 5.39 – 5.49 (s, 2H, -CO-C**H$_2$**-OAr), 6.43 – 6.50 (d, J = 8.55 Hz, 2H, H-3', -5'), 6.90 – 6.98 (m, 2H, H-6, -8), 7.65 – 7.73 (d, J = 8.55 Hz, 3H, H-5, -2', -6')

ESI-MS: cal. 411.14, exp. 411.1 ([M+H]$^+$)

HPLC: 98 % purity at 254 nm

t_R (HPLC): 4.97 min; λ_{max} = 287 nm and 317 nm

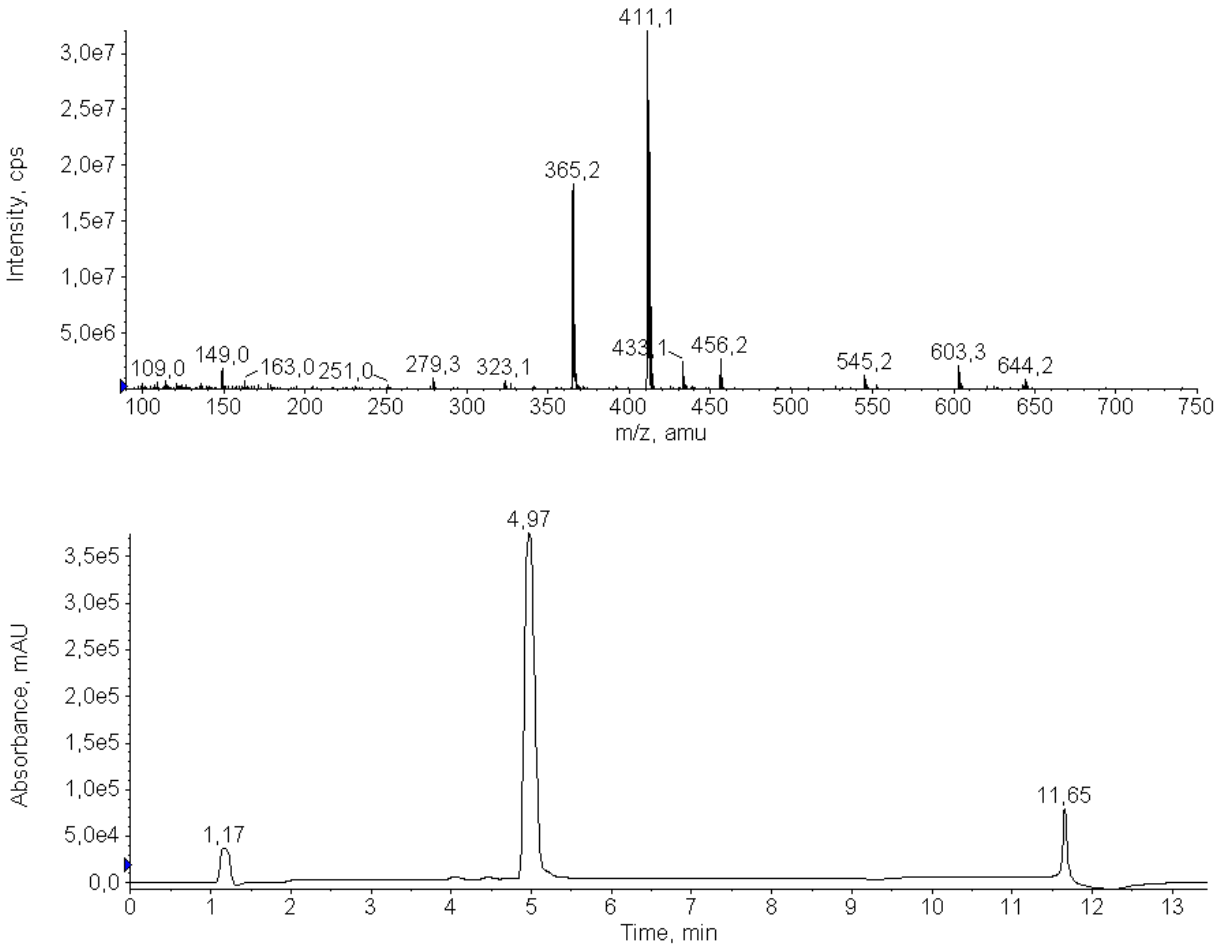

Supporting Information Figure 82: LC/MS analysis of **Scaff10-52**. Upper panel: Mass spectrum. Lower panel: according chromatogram.

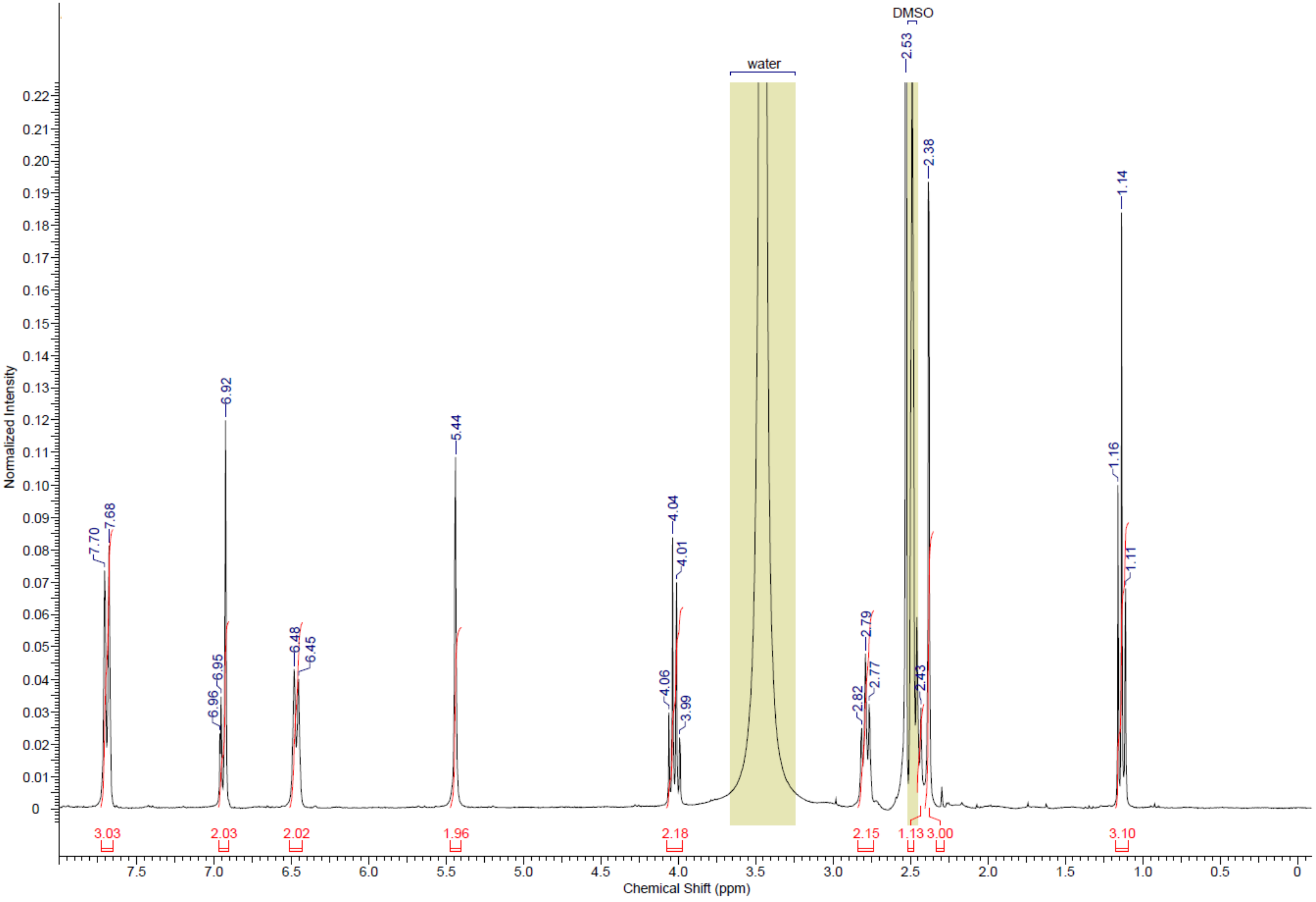

Supporting Information Figure 83: ^{1}H NMR spectrum of **Scaff10-52**, 300 MHz, DMSO-d$_6$.

4.1.46 Ethyl 3-[7-[2-[4-[2-(4-hydroxyphenyl)-2-oxo-ethoxy]phenyl]-2-oxo-ethoxy]-4-methyl-2-oxo-chromen-3-yl]propanoate (**Scaff10-52d**)

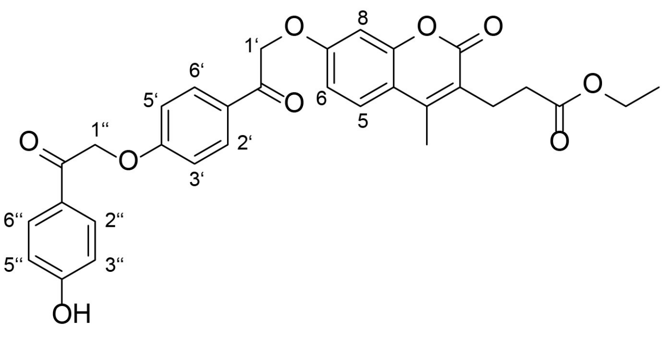

Ethyl 3-(7-hydroxy-4-methyl-2-oxo-chromen-3-yl)propanoate (**5a**) (50 mg, 0.18 mmol) was solved in acetone and preactivated with potash in excess (62 mg, 0.45 mmol) by 55 °C. The α-haloketone 2-bromo-1-(4-hydroxyphenyl)ethanone (**2l**) was added (39 mg, 0.18 mmol). The mixture was refluxed at the same temperature for 4 h. The products Scaff10-52 and Scaff10-52d were purified using preparative HPLC. The final product after purification gave a yellowish solid.

^{1}H NMR (300 MHz, DMSO-d$_6$): δ(ppm) = 1.09 – 1.18 (t, J = 7.33 Hz, 3H, -CH$_2$-C**H$_3$**), 2.35 – 2.42 (s, 3H, Ar-CH$_3$), 2.42 – 2.50 (t, J = 7.33 Hz, 2H, -CH$_2$-C**H$_2$**-COOEt), 2.75 – 2.84 (t, J = 7.33 Hz, 2H, -C**H$_2$**-CH$_2$-COOEt), 3.97 – 4.07 (q, J = 7.33 Hz, 2H, -C**H$_2$**-CH$_3$), 5.34 – 5.42 (s, 2H, H-1''), 5.60 – 5.66 (s, 2H, H-1'), 6.19 – 6.27 (d, J = 8.55 Hz, 2H, H-3'', -5''), 6.95 – 7.06 (m, 4H, H-6, -8, -3', -5'), 7.56 – 7.63 (d, J = 9.77 Hz, 2H, H-2', -6'), 7.67 – 7.73 (d, J = 9.77 Hz, 1H, H-5), 7.92 – 7.99 (d, J = 8.55 Hz , 2H, H-2'', -6'')

ESI-MS: cal. 545.18, exp. 545.1 ($[M+H]^+$)

HPLC: 98 % purity at 254 nm

t_R (HPLC): 5.35 min; $λ_{max}$ = 287 nm and 316 nm

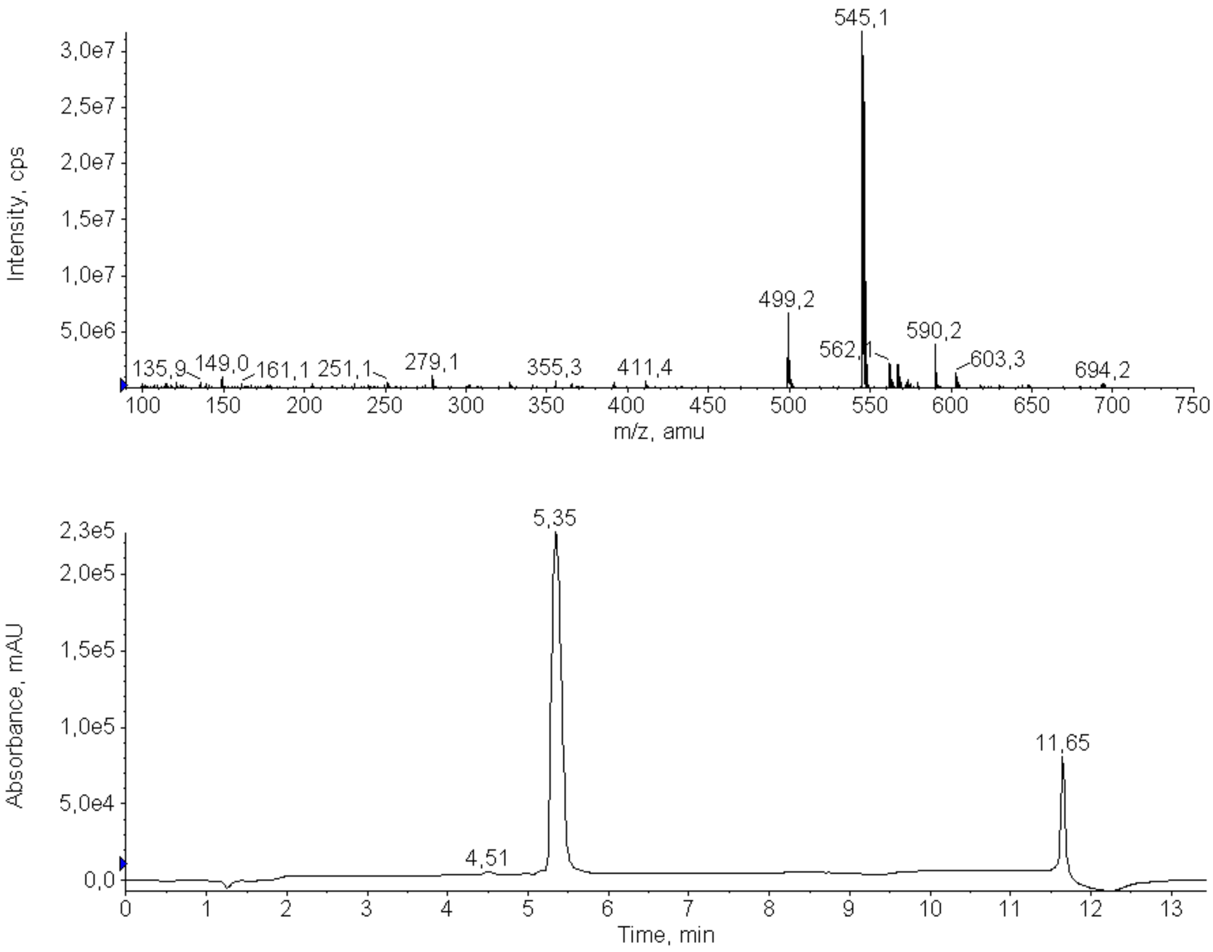

Supporting Information Figure 84: LC/MS analysis of **Scaff10-52d**. Upper panel: Mass spectrum. Lower panel: according chromatogram.

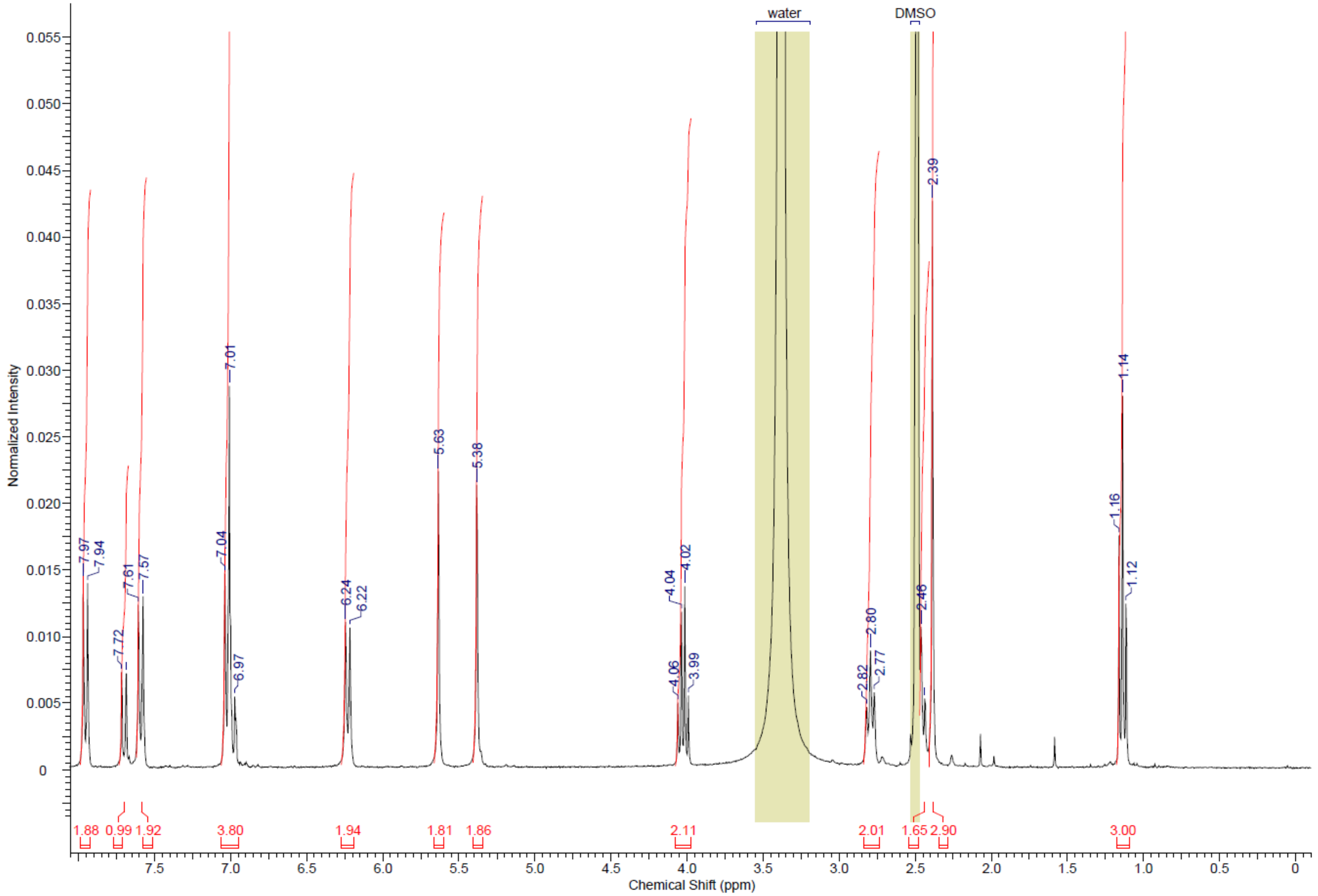

Supporting Information Figure 85: ^{1}H NMR spectrum of **Scaff10-52d**, 300 MHz, DMSO-d$_6$.

4.1.47 Ethyl 3-(4-methyl-2-oxo-7-phenacyloxy-chromen-3-yl)propanoate (**Scaff10-56**)

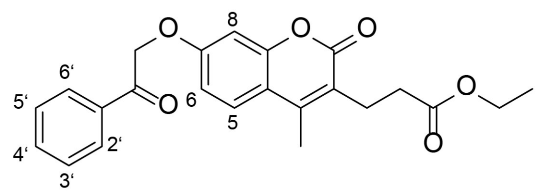

Ethyl 3-(7-hydroxy-4-methyl-2-oxo-chromen-3-yl)propanoate (5**a**) (100 mg, 0.36 mmol) was solved in 4 ml dry acetone and preactivated with potash in excess (125 mg, 0.90 mmol) by 55 °C. The α-haloketone 2-bromo-1-phenylethanone (**2m**) was added (72 mg, 0.36 mmol). The mixture was refluxed at the same temperature for 2 h. The product was precipitated using 1 M H_2SO_4 and purified using preparative HPLC with the following gradient: 5 % to 90 % ACN in H_2O in 80 min. The product eluted at t_R = 61 min and was obtained after lyophilisation as white solid in 5 % yield (7 mg).

^{1}H NMR (300 MHz, DMSO-d_6): δ(ppm) = 1.09 – 1.22 (t, J = 7.32 Hz, 3H, -CH_2-C**H₃**), 2.37 – 2.43 (s, 3H, Ar-CH_3), 2.43 – 2.50 (t, J = 7.32 Hz, 2H, -CH_2-C**H₂**-COOEt), 2.75 – 2.87 (t, J = 7.32 Hz, 2H, -C**H₂**-CH_2-COOEt), 3.98 – 4.09 (q, J = 7.32 Hz, 2H, -C**H₂**-CH_3), 5.70 – 5.79 (s, 2H, -CO-C**H₂**-OAr), 6.98 – 7.09 (m, 2H, H-6, -8), 7.53 – 7.63 (t, J = 8.54 Hz, 2H, H-3', -5'), 7.67 – 7.76 (m, 2H, H-5, -4'), 8.00 – 8.07 (d, J = 7.32 Hz , 2H, H-2', -6')

ESI-MS: cal. 395.14, exp. 395.1 ([M+H]$^+$)

HPLC: 99 % purity at 254 nm

t_R (HPLC): 6.77 min; λ_{max} = 285 nm and 320 nm

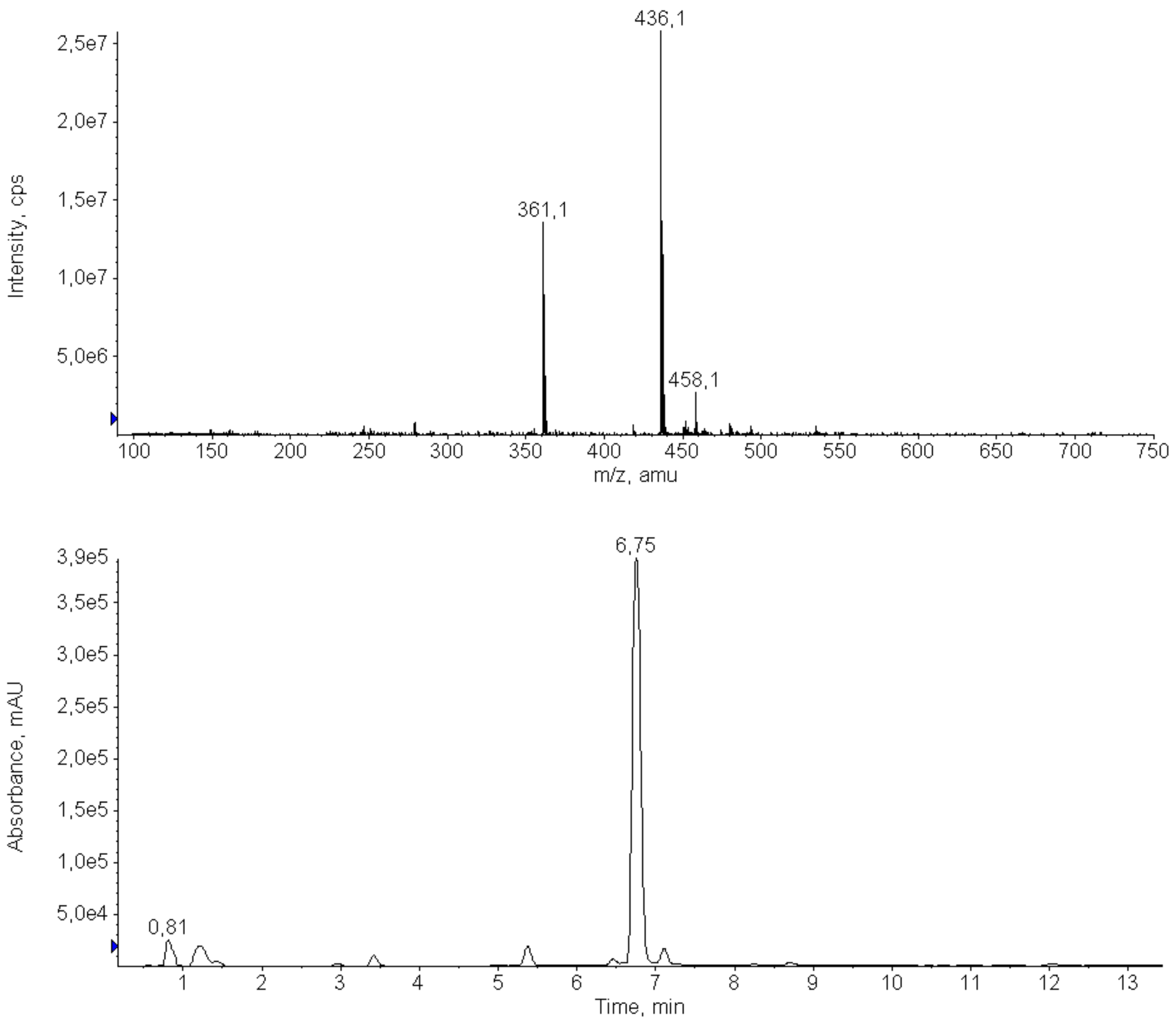

Supporting Information Figure 86: LC/MS analysis of **Scaff10-56**. Upper panel: Mass spectrum. Lower panel: according chromatogram.

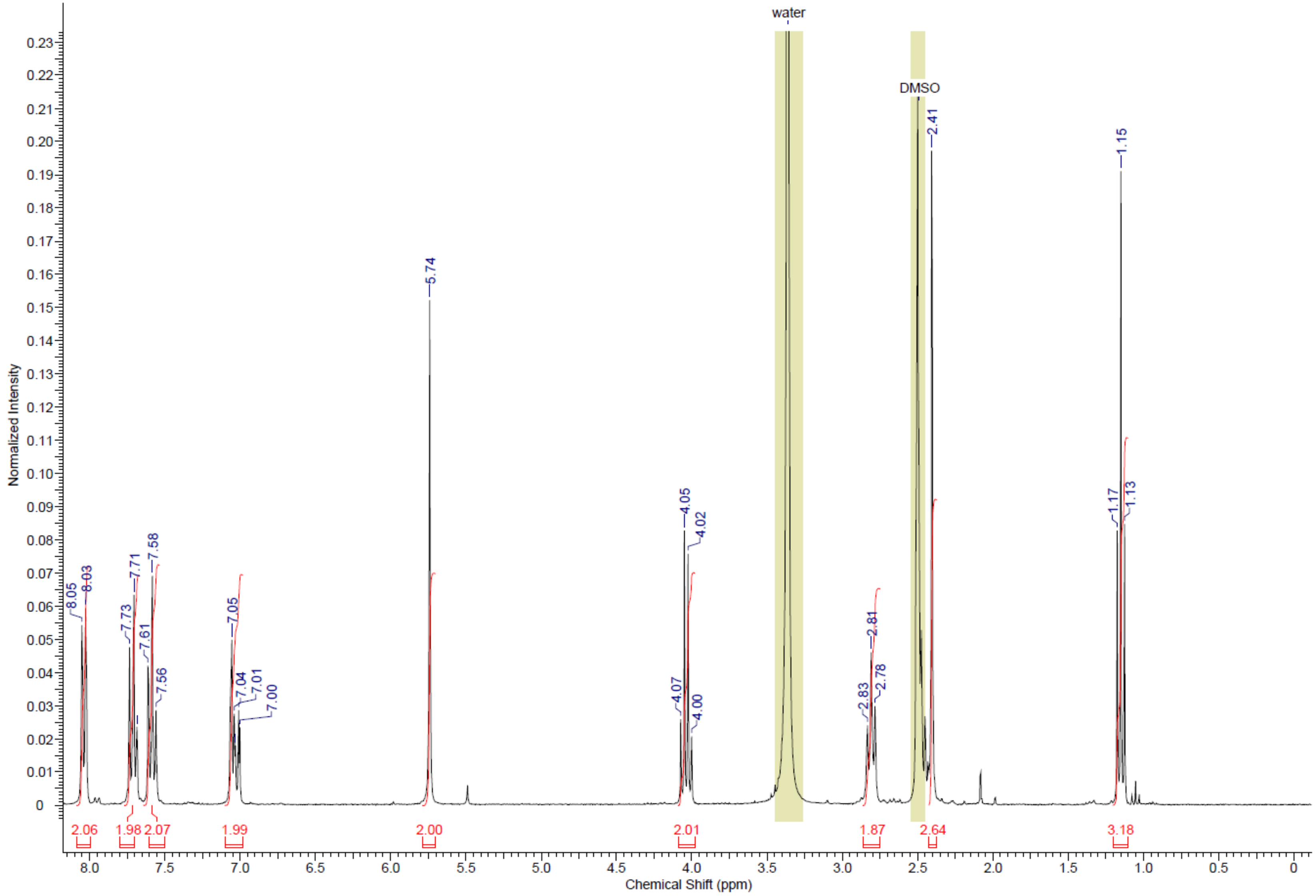

Supporting Information Figure 87: ^{1}H NMR spectrum of **Scaff10-56**, 300 MHz, DMSO-d_6.

4.1.48 3-(5-Methyl-7-oxo-3-phenyl-furo[3,2-*g*]chromen-6-yl)propanoic acid (**Scaff10-58**)

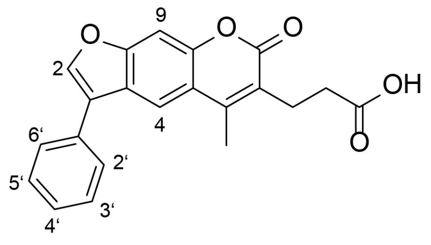

Ethyl 3-(4-methyl-2-oxo-7-phenacyloxy-chromen-3-yl)propanoate (**Scaff10-56**) (200 mg, 0.51 mmol) was saponificated in a mixture of 1 ml dry acetone and 4 ml 1 M sodium hydroxide at 60 °C for 1.5 h. Dropwise addition of 1 M HCl resulted in precipitation of the product. The precipitated solid was purified using preparative HPLC with the following gradient: 5 % to 90 % ACN in H_2O in 80 min. The product eluted at t_R = 27 min and was obtained after lyophilisation as orange-yellowish solid in 56 % yield (100 mg).

^{1}H NMR (300 MHz, DMSO-d_6): δ(ppm) = 1.96 – 2.11 (t, J = 7.32 Hz, 2H, -CH_2-C**H_2**-COOH), 2.52 – 2.60 (s, 3H, Ar-CH_3), 2.71 – 2.83 (t, J = 8.54 Hz, 2H, -C**H_2**-CH_2-COOH), 7.38 – 7.47 (t, J = 7.32 Hz, 1H, H-4'), 7.50 – 7.59 (t, J = 7.32 Hz, 2H, H-3', -5'), 7.71 – 7.74 (s, 1H, H-9), 7.77 – 7.84 (d, J = 7.32 Hz, 2H, H-2', -6'), 8.08 – 8.14 (s, 1H, H-4), 8.42 – 8.47 (s, 1H, H-2)

ESI-MS: cal. 349.10, exp. 349.1 ($[M+H]^+$)

HPLC: 99 % purity at 254 nm

t_R (HPLC): 6.39 min; λ_{max} = 246 nm and 300 nm

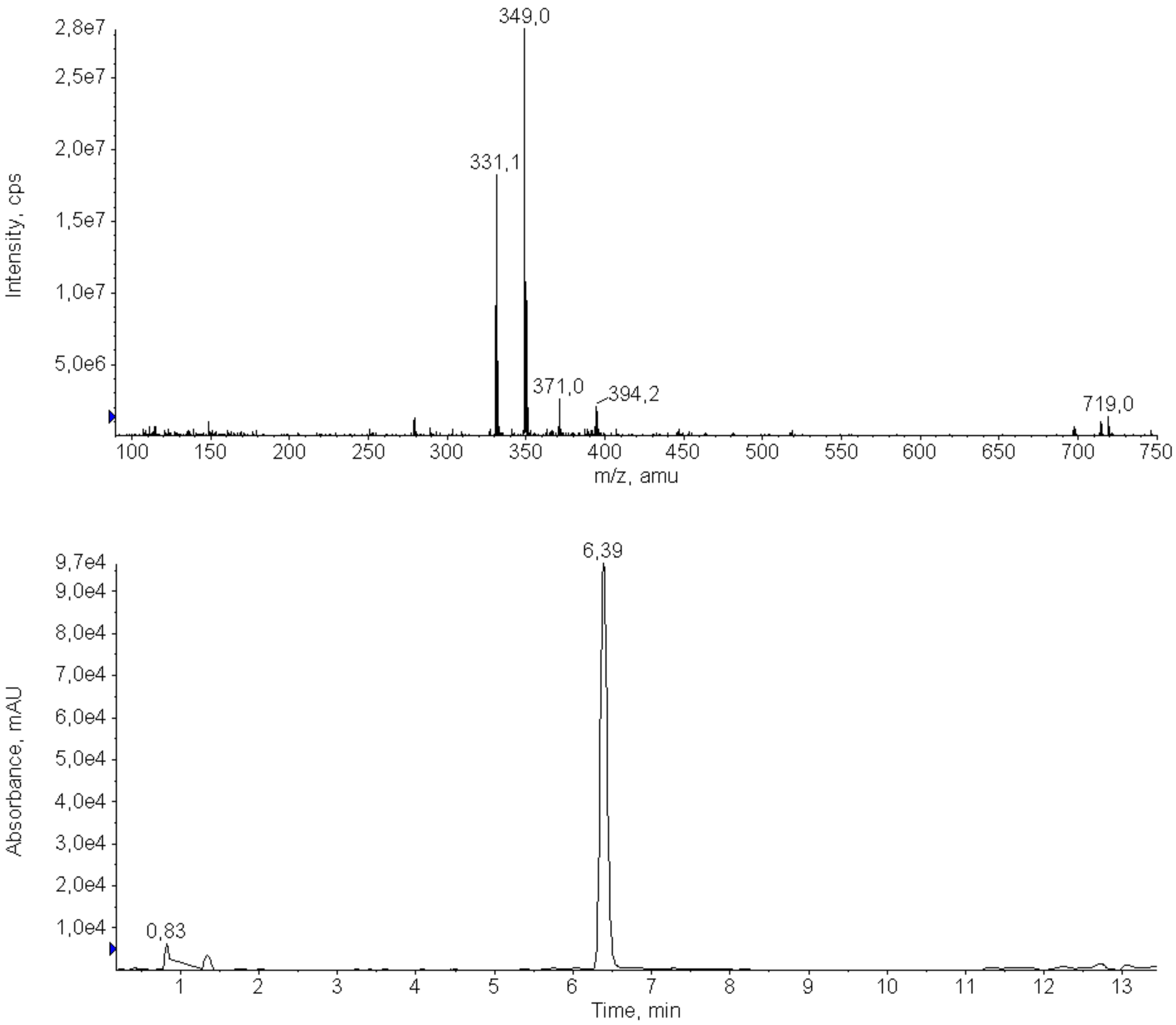

Supporting Information Figure 88: LC/MS analysis of **Scaff10-58**. Upper panel: Mass spectrum. Lower panel: according chromatogram.

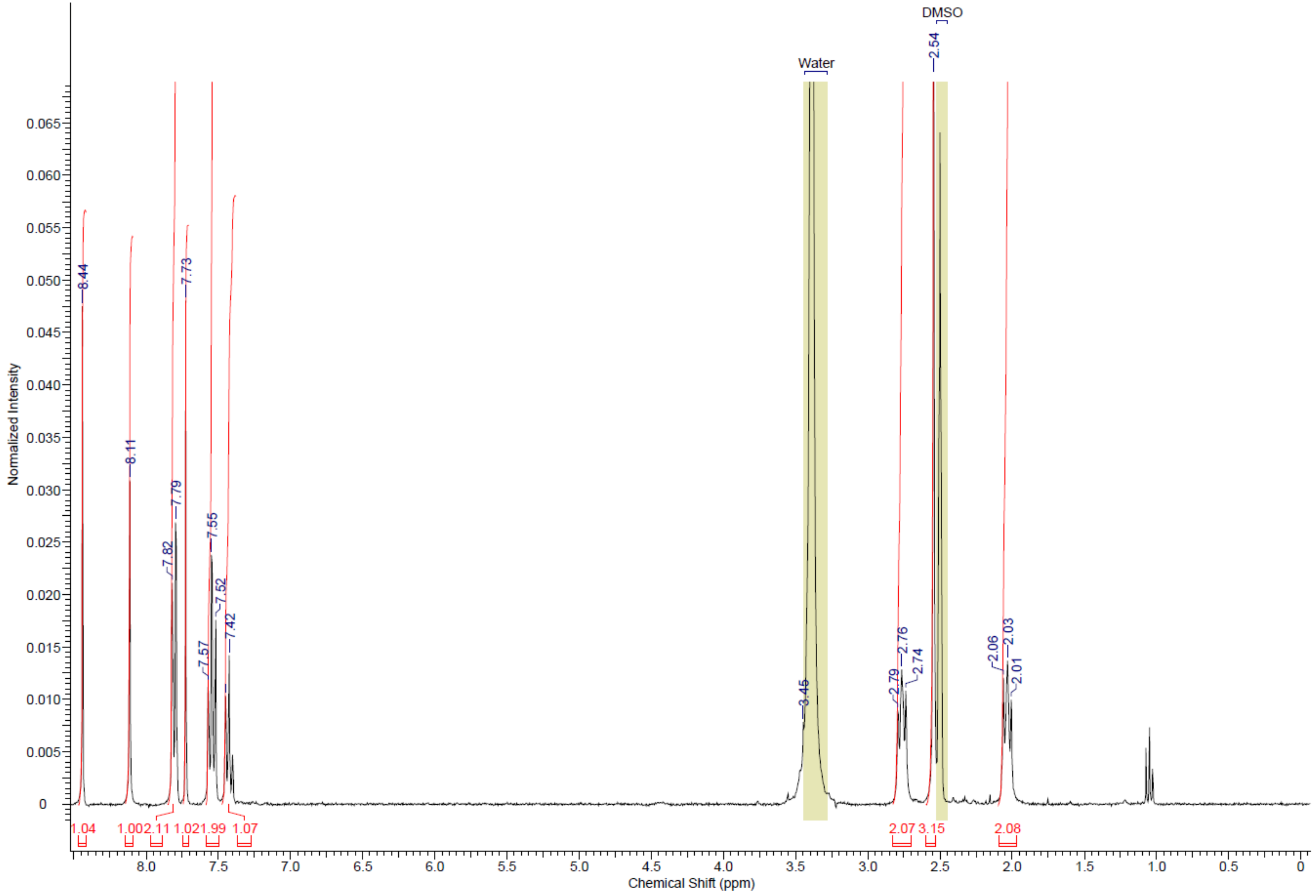

Supporting Information Figure 89: ^{1}H NMR spectrum of **Scaff10-34**, 300 MHz, DMSO-d_6.

4.1.49 Ethyl 3-[7-[(4-methoxyphenyl)methoxy]-4-methyl-2-oxo-chromen-3-yl]propanoate (**Scaff10-59**)

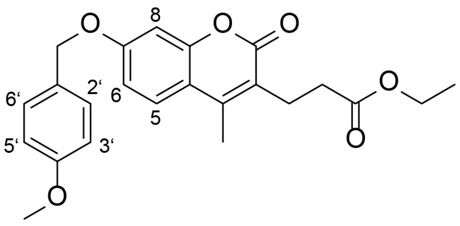

Ethyl 3-(7-hydroxy-4-methyl-2-oxo-chromen-3-yl)propanoate (**5a**) (50 mg, 0.18 mmol) was solved in acetone and preactivated with potash in excess (62 mg, 0.45 mmol) by 55 °C. The α-haloketone 1-(bromomethyl)-4-methoxy-benzene (**2n**) was added (36 mg, 0.18 mmol). The mixture was refluxed at the same temperature for 16 h. The product was precipitated using 1 M H_2SO_4. The final product after purification gave a white solid.

^{1}H NMR (300 MHz, DMSO-d_6): δ(ppm) = 1.09 – 1.17 (t, J = 7.33 Hz, 3H, -CH_2-C**H_3**), 2.36 – 2.40 (s, 3H, Ar-CH_3), 2.42 – 2.49 (t, J = 7.33 Hz, 2H, -CH_2-C**H_2**-COOEt), 2.74 – 2.84 (t, J = 7.33 Hz, 2H, -C**H_2**-CH_2-COOEt), 3.71 – 3.78 (s, 3H, -OCH_3), 3.98 – 4.08 (q, J = 7.33 Hz, 2H, -C**H_2**-CH_3), 5.09 – 5.15 (s, 2H, -O-C**H_2**-Ar), 6.91 – 7.04 (m, 4H, H-6, -8, -3', -5'), 7.36 – 7.42 (d, J = 9.77 Hz, 2H, H-2', -6'), 7.67 – 7.72 (d, J = 8.55 Hz , 1H, H-5)

ESI-MS: cal. 397.16, exp. 397.1 ($[M+H]^+$)

HPLC: 90 % purity at 254 nm

t_R (HPLC): 7.44 min; $λ_{max}$ = 282 nm and 323 nm

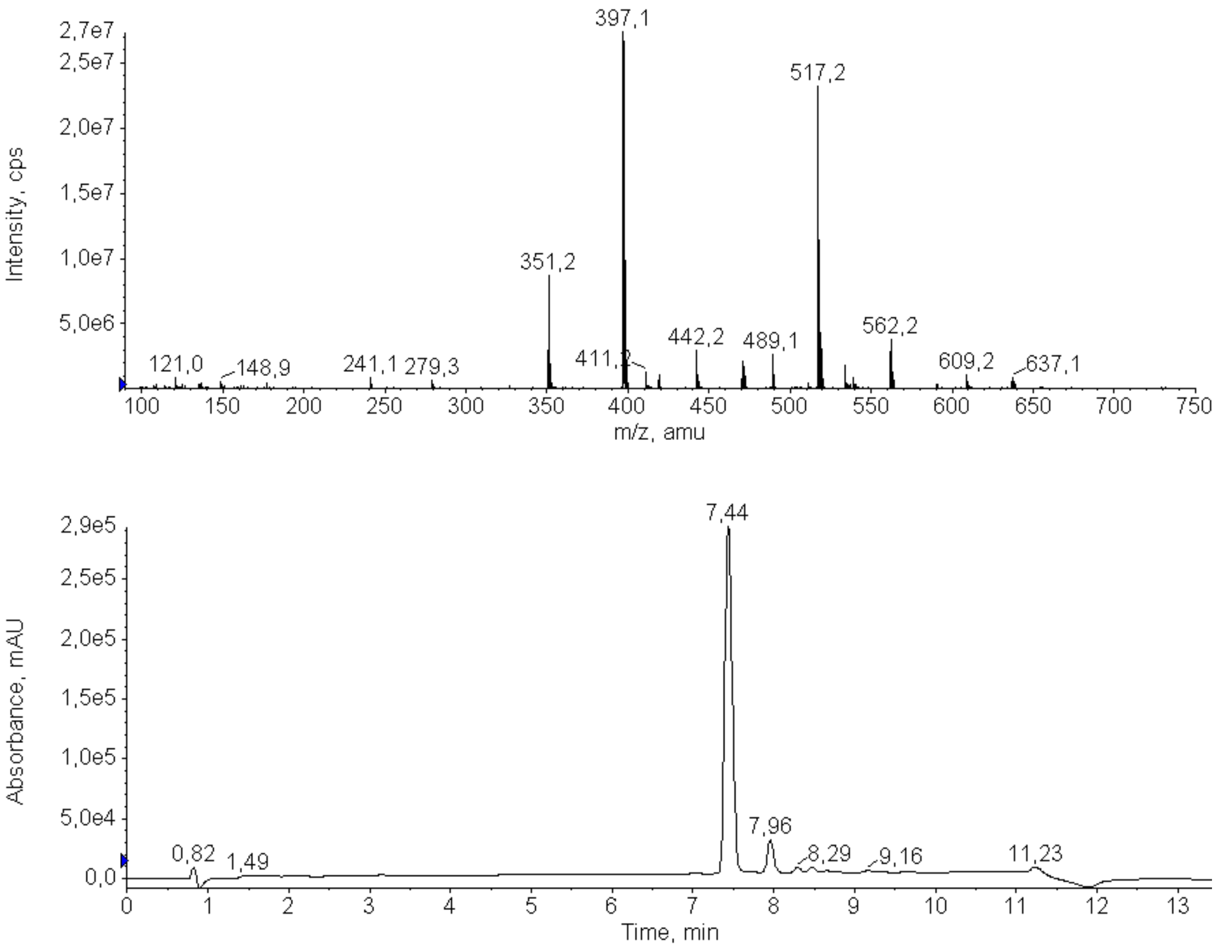

Supporting Information Figure 90.: LC/MS analysis of **Scaff10-59**. Upper panel: Mass spectrum. Lower panel: according chromatogram.

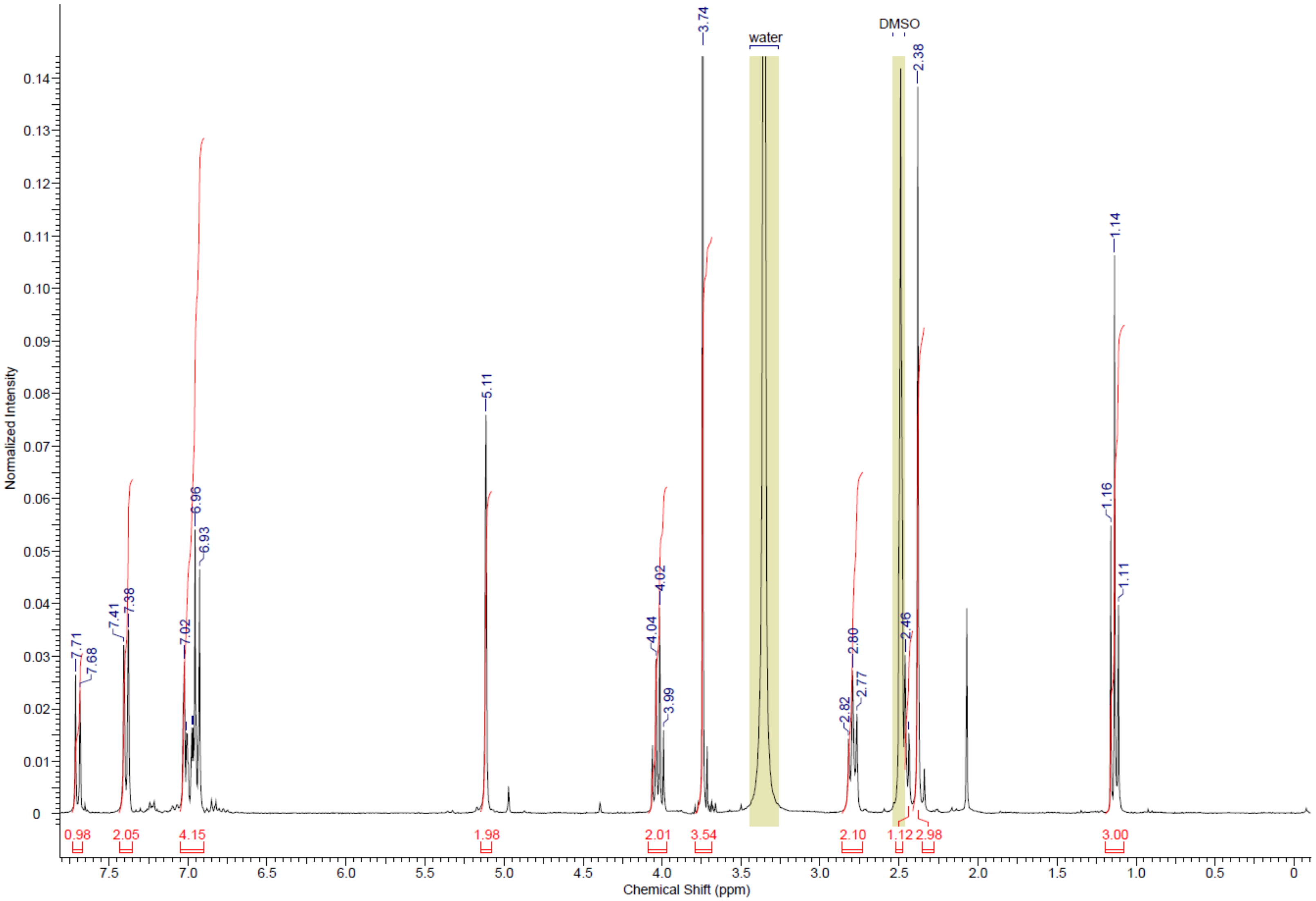

Supporting Information Figure 91: ^{1}H NMR spectrum of **Scaff10-59**, 300 MHz, DMSO-d_6.

4.1.50 3-[7-[(4-Methoxyphenyl)methoxy]-4-methyl-2-oxo-chromen-3-yl]propanoic acid (**Scaff10-60**)

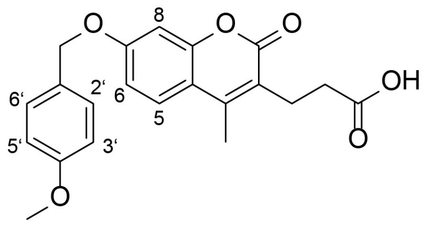

Ethyl 3-[7-[(4-methoxyphenyl)methoxy]-4-methyl-2-oxo-chromen-3-yl]propanoate (**Scaff10-59**) (50 mg, 0.13 mmol) was saponificated in a mixture of ethanol and 2 ml 1 M sodium hydroxide at 80 °C for 1 h. Dropwise addition of 1 M H_2SO_4 resulted in precipitation of the product. The precipitated solid was filtered. The final product after purification gave a white solid.

^{1}H NMR (300 MHz, DMSO-d_6): δ(ppm) = 2.31 – 2.44 (m, 5H, Ar-CH_3, -CH_2-C**H_2**-COOH), 2.69 – 2.83 (t, J = 7.33 Hz, 2H, -C**H_2**-CH_2-COOH), 3.70 – 3.79 (s, 3H, -OCH_3), 5.07 – 5.17 (s, 2H, -O-C**H_2**-Ar), 6.90 – 7.05 (m, 4H, H-6, -8, -3’, -5’), 7.36 – 7.43 (d, J = 8.55 Hz, 2H, H-2’, -6’), 7.67 – 7.73 (d, J = 8.55 Hz , 1H, H-5)

ESI-MS: cal. 369.13, exp. 369.1 ([M+H]+)

HPLC: 98 % purity at 254 nm

t_R (HPLC): 6.01 min; λ_{max} = 283 nm and 322 nm

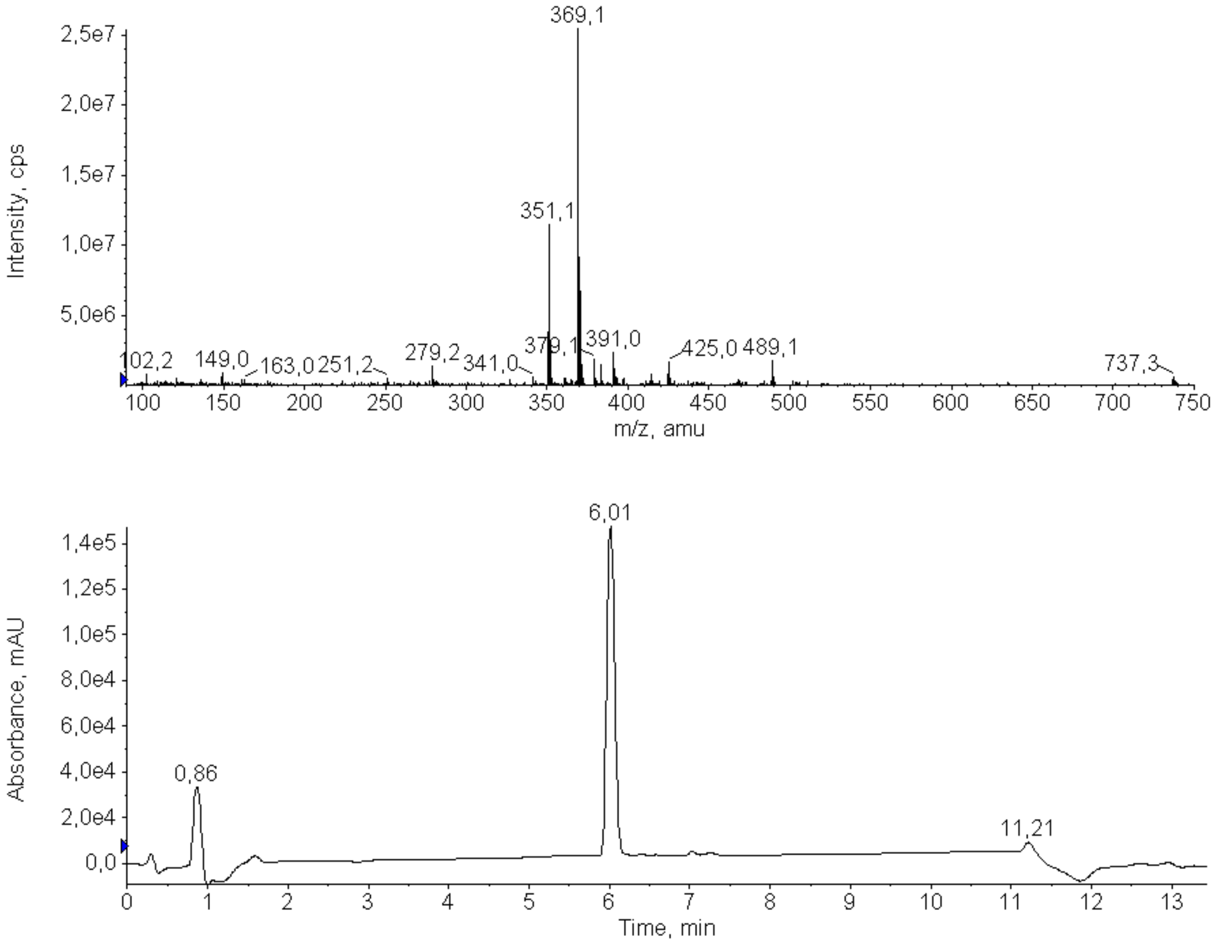

Supporting Information Figure 92: LC/MS analysis of **Scaff10-60**. Upper panel: Mass spectrum. Lower panel: according chromatogram.

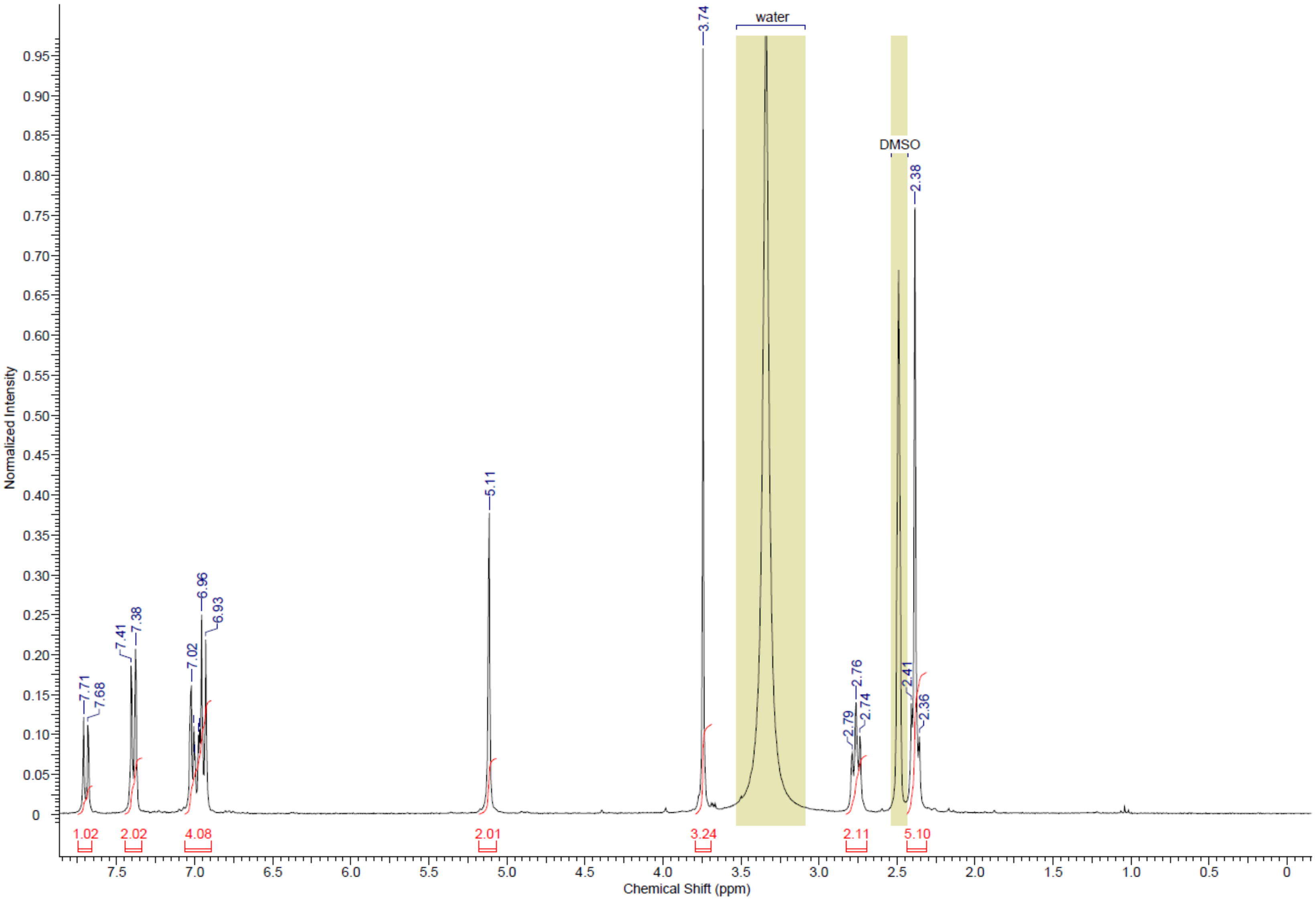

Supporting Information Figure 93: ^{1}H NMR spectrum of **Scaff10-60**, 300 MHz, DMSO-d_6.

4.1.51 2-[3-[3-(4-Methoxyphenyl)-5-methyl-7-oxo-furo[3,2-*g*]chromen-6-yl]propanoylamino]acetic acid (**Scaff10-61**)

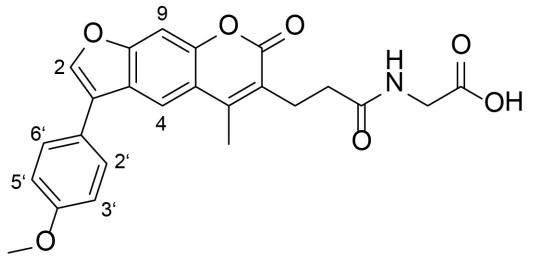

3-[3-(4-Methoxyphenyl)-5-methyl-7-oxo-furo[3,2-*g*]chromen-6-yl]propanoic acid (**Scaff10-8**) (50 mg, 0.13 mmol) was incubated with diisopropylcarbodiimide (DIC) (20 mg, 0.16 mmol) and 1-hydroxybenzotriazole (HOBt) (22 mg, 0.16 mmol) solved in 3 ml DMF for 5 min at 25 °C. A mixture of glycin-tert-butylester (67 mg, 0.40 mmol), TEA (40 mg, 0.4 mmol) and 1 ml DMF was added. After 24 h at 25 °C, 4 ml of water, 2 ml 1 M NH_4Cl, 2 ml 1 M $NaHCO_3$ and 10 ml ethyl acetate were added. In order to remove the tert-butyl moiety, the organic layer was mixed with 5 ml methylene chloride, 4.5 ml trifluoro acetic acid and 0.5 ml water. After 1 h at 25 °C, the solution was neutralised using $NaHCO_3$. Solvent was removed under reduced pressure. The solid was resolved in ethyl acetate and extracted in citric acid (pH 3). Further purification of the organic layer was performed using preparative HPLC with the following gradient: 5 % to 90 % ACN in H_2O in 80 min. The product eluted at t_R = 33 min and was obtained after lyophilisation as white solid in 6 % yield (3.5 mg).

^{1}H NMR (300 MHz, DMSO-d_6): δ(ppm) = 2.28 – 2.42 (t, J = 7.32 Hz, 3H, -CH_2-C**H₂**-CONH-), 2.52 – 2.58 (s, 3H, Ar-CH_3), 2.80 – 2.82 (t, J = 7.32 Hz, 2H, -CH_2-C**H₂**-CONH-), 3.68 – 3.77 (d, J = 6.10 Hz, 2H, -CONH-C**H₂**-COOH), 3.78 – 3.89 (s, 3H, -OCH_3), 7.07 – 7.15 (d, J = 8.54 Hz, 2H, H-3’, -5’), 7.70 – 7.78 (m, 3H, H-9, -2’, -6’), 8.10 – 8.14 (s, 1H, H-4), 8.22 – 8.30 (t, J = 6.10 Hz , 1H, -CONH-), 8.36 -8.40 (s, 1H, H-2)

ESI-MS: cal. 436.14, exp. 436.1 ($[M+H]^+$)

HPLC: 97 % purity at 254 nm

t_R (HPLC): 5.63 min; λ_{max} = 250 nm and 306 nm

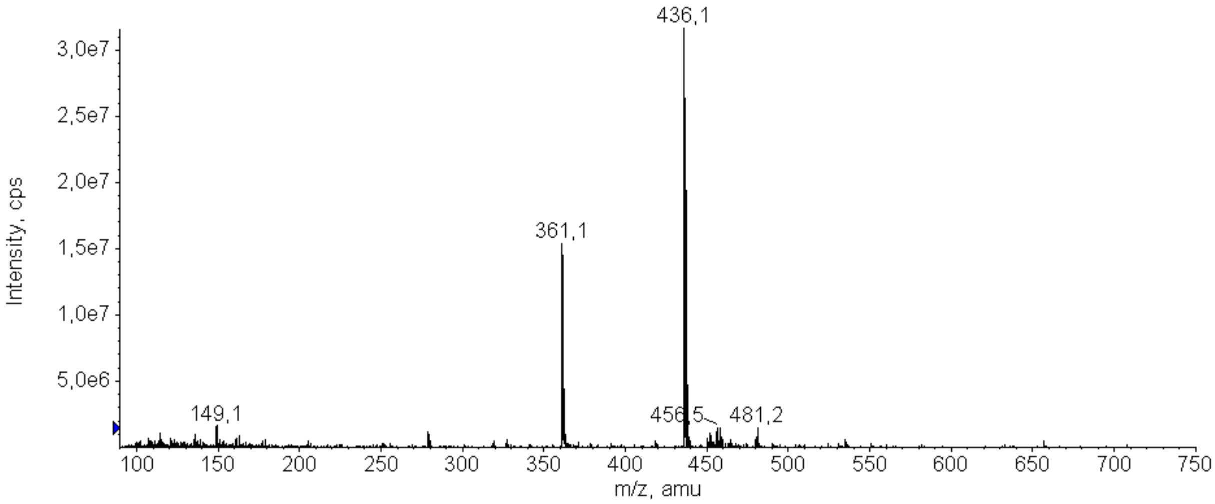

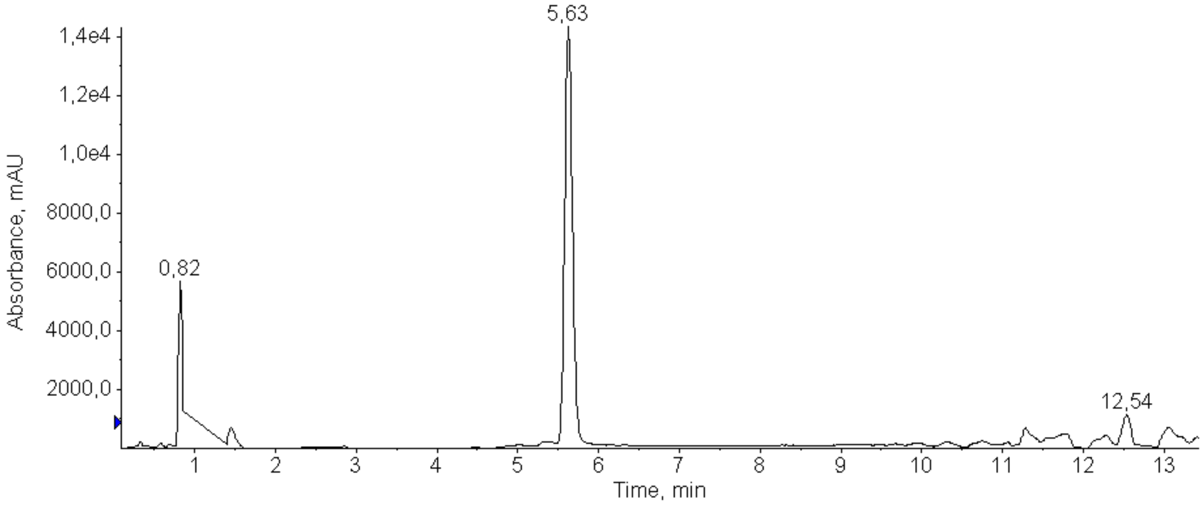

Supporting Information Figure 94: LC/MS analysis of **Scaff10-61**. Upper panel: Mass spectrum. Lower panel: according chromatogram.

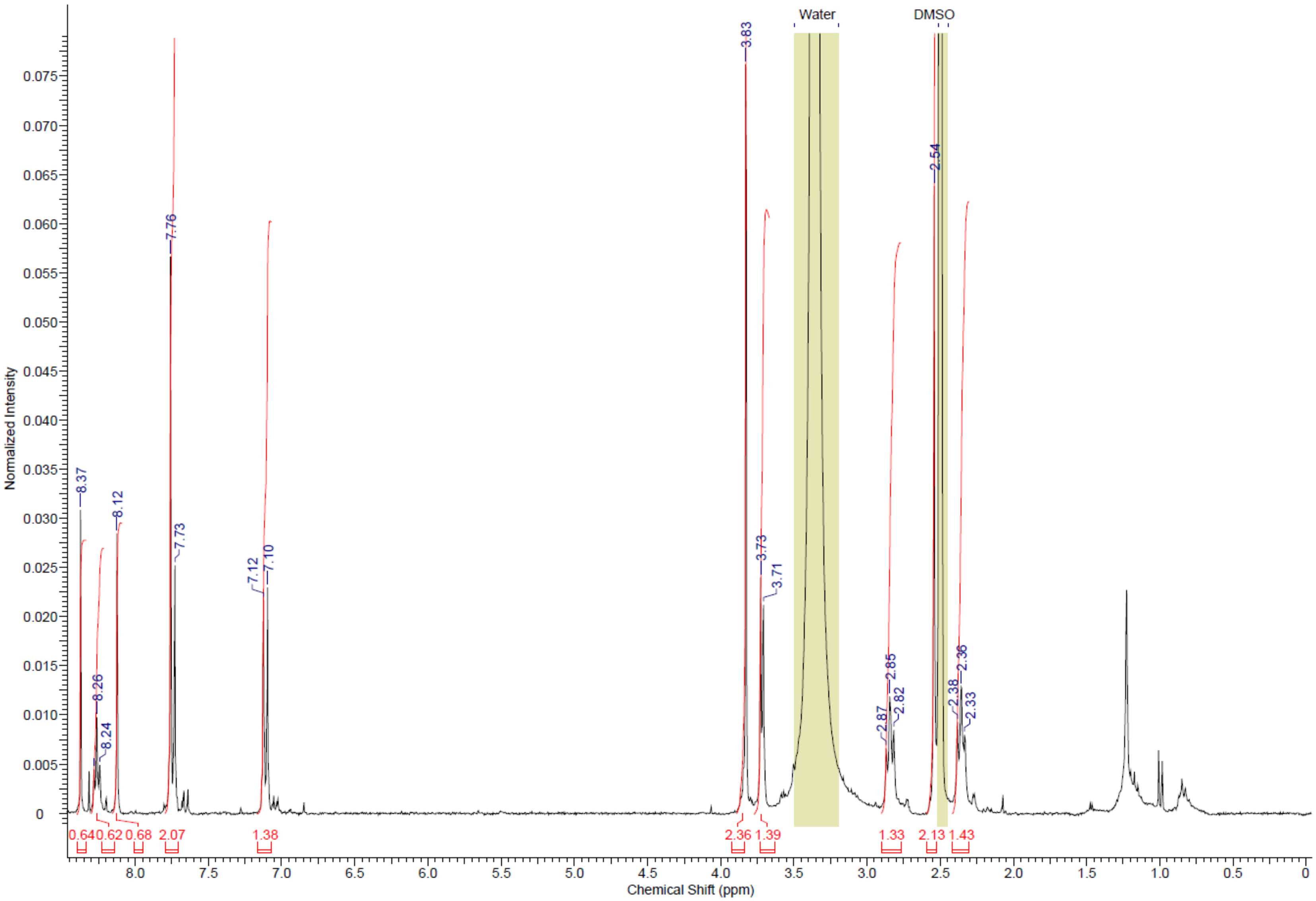

Supporting Information Figure 95: ^{1}H NMR spectrum of **Scaff10-61**, 300 MHz, DMSO-d$_6$.

4.1.52 Ethyl 3-[7-[2-[4-(3-methoxyphenyl)phenyl]-2-oxo-ethoxy]-4-methyl-2-oxo-chromen-3-yl]propanoate (**Scaff10-62**)

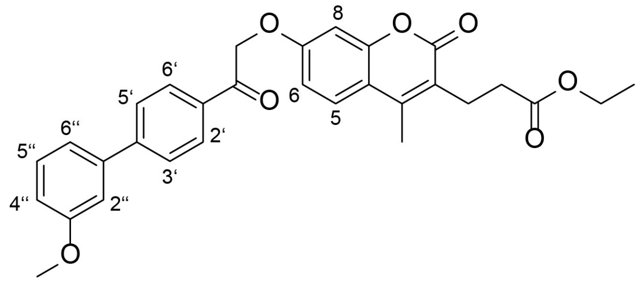

Ethyl 3-(7-hydroxy-4-methyl-2-oxo-chromen-3-yl)propanoate (**5a**) (196 mg, 0.71 mmol) was solved in 8 ml dry acetone and preactivated with potash in excess (245 mg, 1.77 mmol) at 55 °C. The α-haloketone 2-iodo-1-[4-(3-methoxyphenyl)phenyl]ethanone (**2a**) was added (250 mg, 0.71 mmol). The mixture was refluxed at the same temperature for 0.5 h. The product was precipitated using 1 M H_2SO_4 and purified using preparative HPLC with the following gradient: 25 % to 95 % ACN in H_2O in 80 min. The product eluted at t_R = 62.5 min and was obtained after lyophilisation as white solid in 6 % yield (21 mg).

^{1}H NMR (300 MHz, DMSO-d_6): δ(ppm) = 1.08 – 1.20 (t, J = 7.32 Hz, 3H, -CH_2-C**H₃**), 2.34 – 2.43 (s, 3H, Ar-CH_3), 2.42 – 2.54 (t, J = 8.54 Hz, 2H, -CH_2-C**H₂**-COOEt), 2.76 – 2.87 (t, J = 7.32 Hz, 2H, -C**H₂**-CH_2-COOEt), 3.80 – 3.88 (s, 3H, -OCH_3), 3.99 – 4.10 (q, J = 7.32 Hz, 2H, -C**H₂**-CH_3), 5.73 – 5.80 (s, 2H, -CO-C**H₂**-OAr), 6.99 – 7.11 (m, 3H, H-6, -8, -4''), 7.28 – 7.37 (m, 2H, H-2'', -6''), 7.40 – 7.48 (t, J = 7.32 Hz, 1H, H-5''), 7.70 – 7.77 (d, J = 9.77 Hz , 1H, H-5), 7.86 – 7.93 (d, J = 8.54 Hz, 2H, H-3', -5'), 8.08 – 8.15 (d, J = 8.55 Hz, 2H, H-2', -6')

ESI-MS: cal. 501.19, exp. 501.1 ($[M+H]^+$)

HPLC: 98 % purity at 254 nm

t_R (HPLC): 7.86 min; λ_{max} = 292 nm and 310 nm

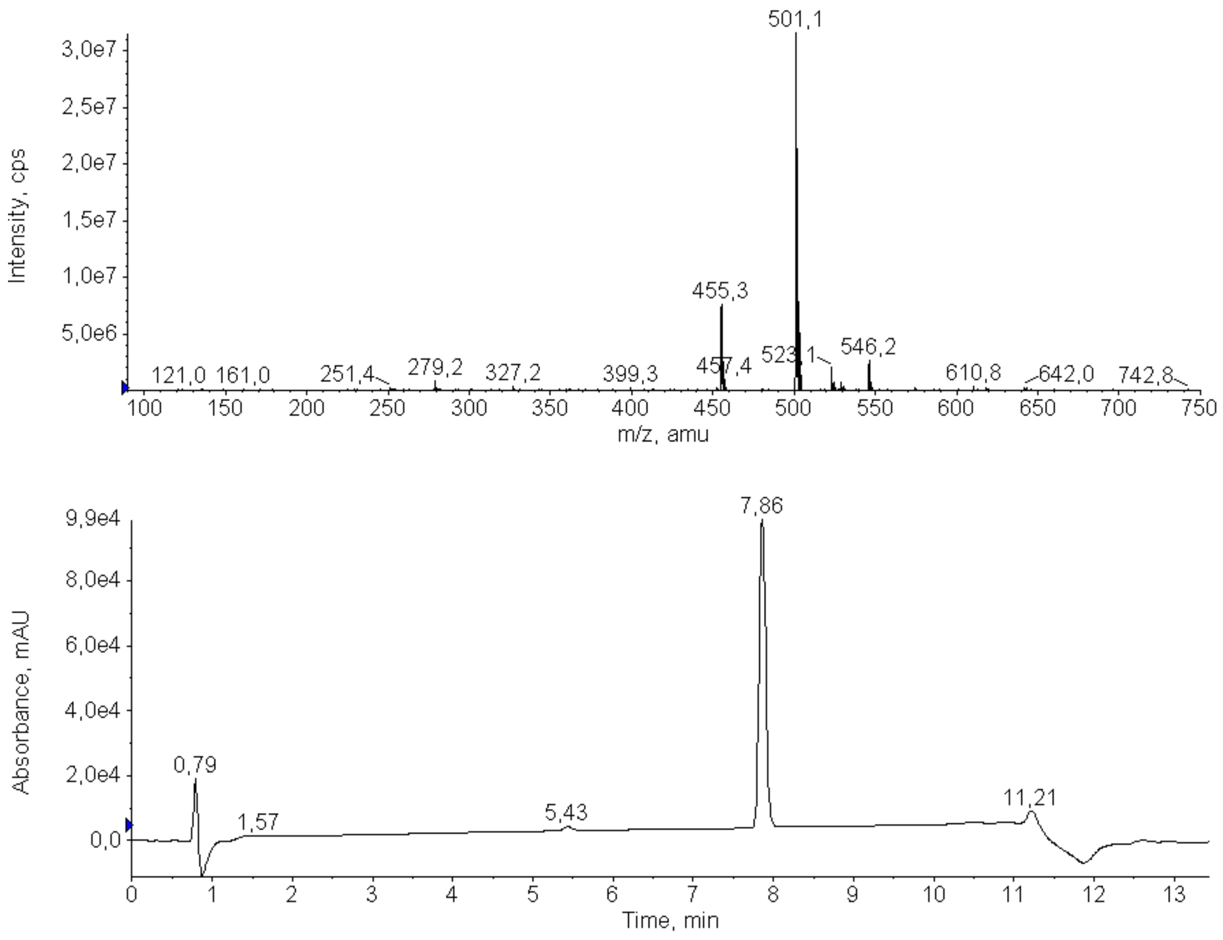

Supporting Information Figure 96.: LC/MS analysis of **Scaff10-62**. Upper panel: Mass spectrum. Lower panel: according chromatogram.

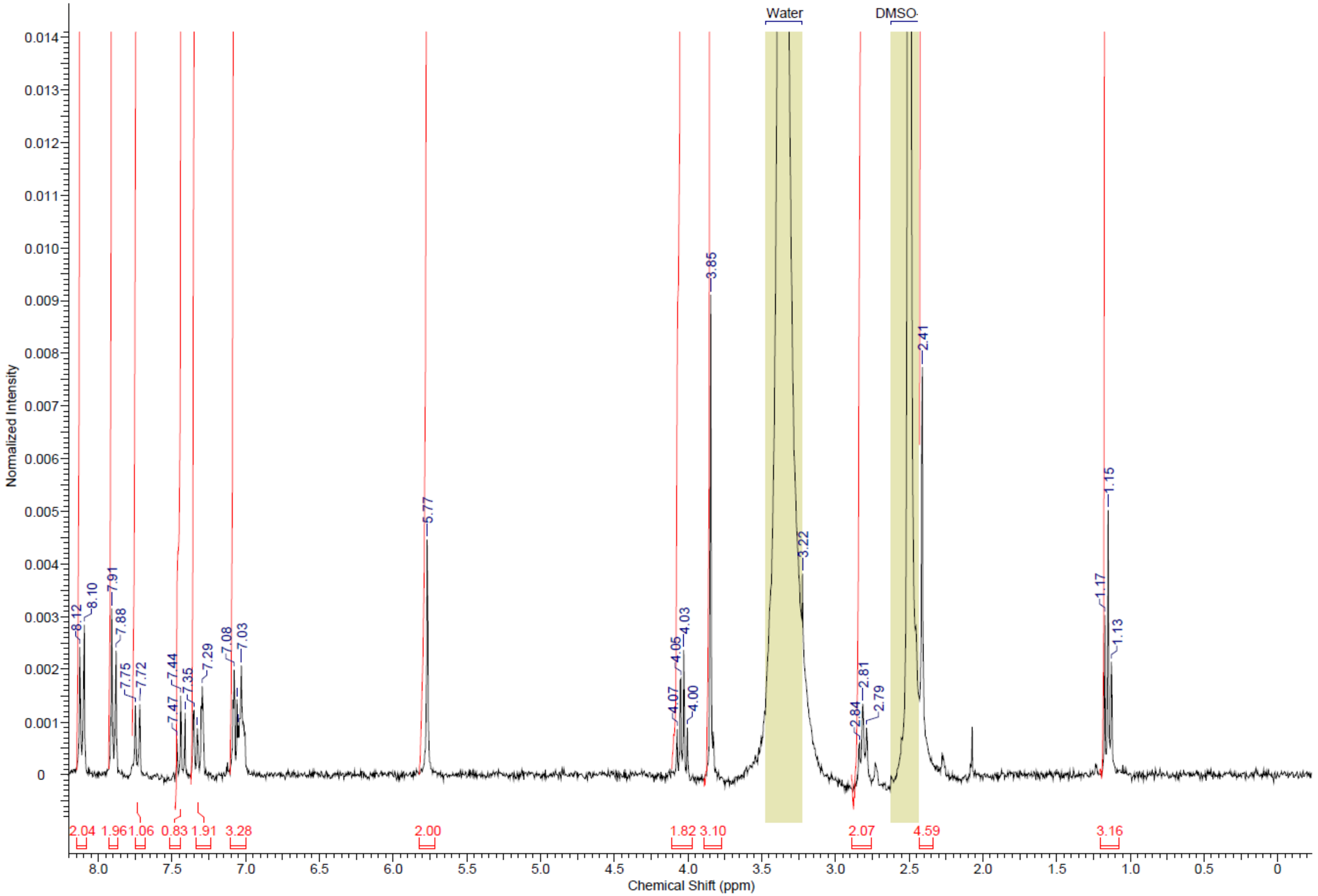

Supporting Information Figure 97: ^{1}H NMR spectrum of **Scaff10-62**, 300 MHz, DMSO-d_6.

4.1.53 3-[7-[2-[4-(3-Methoxyphenyl)phenyl]-2-oxo-ethoxy]-4-methyl-2-oxo-chromen-3-yl]propanoic acid (**Scaff10-63**)

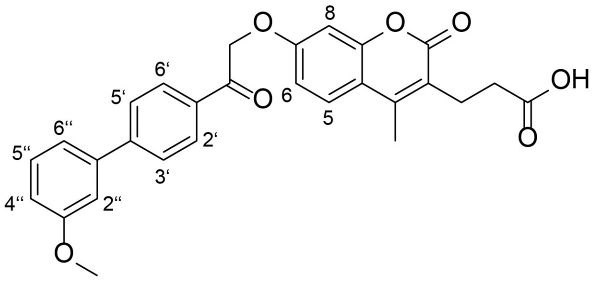

Ethyl 3-[7-[2-[4-(3-methoxyphenyl)phenyl]-2-oxo-ethoxy]-4-methyl-2-oxo-chromen-3-yl] propanoate (**Scaff10-62**) (50 mg, 0.10 mmol) was saponificated in a mixture of 0.6 ml DMF and 1 ml 1 M sodium hydroxide at 95 °C for 0.3 h. Dropwise addition of 1 M H_2SO_4 resulted in precipitation of the product. The precipitated solid was filtered and purified using preparative HPLC with the following gradient: 20 % to 90 % ACN in H_2O in 80 min. The product eluted at t_R = 55 min and was obtained after lyophilisation as white solid in 26 % yield (12 mg).

^{1}H NMR (300 MHz, DMSO-d_6): δ(ppm) = 2.32 – 2.45 (t, J = 7.32 Hz, 2H, -CH_2-C**H₂**-COOH), 2.39 – 2.44 (s, 3H, Ar-CH_3), 2.73 – 2.84 (t, J = 7.32 Hz, 2H, -C**H₂**-CH_2-COOH), 3.79 – 3.89 (s, 3H, -OCH_3), 5.72 – 5.80 (s, 2H, -CO-C**H₂**-OAr), 6.97 – 7.11 (m, 3H, H-6, -8, -4''), 7.27 – 7.37 (m, 2H, H-2'', -6''), 7.40 – 7.48 (t, J = 8.55 Hz, 1H, H-5''), 7.69 – 7.77 (d, J = 8.54 Hz, 1H, H-5), 7.86 – 7.93 (d, J = 8.54 Hz, 2H, H-3', -5'), 8.08 – 8.15 (d, J = 8.55 Hz, 2H, H-2', -6')

ESI-MS: cal. 473.16, exp. 473.1 ($[M+H]^+$)

HPLC: 99 % purity at 254 nm

t_R (HPLC): 6.64 min; λ_{max} = 291 nm and 308 nm

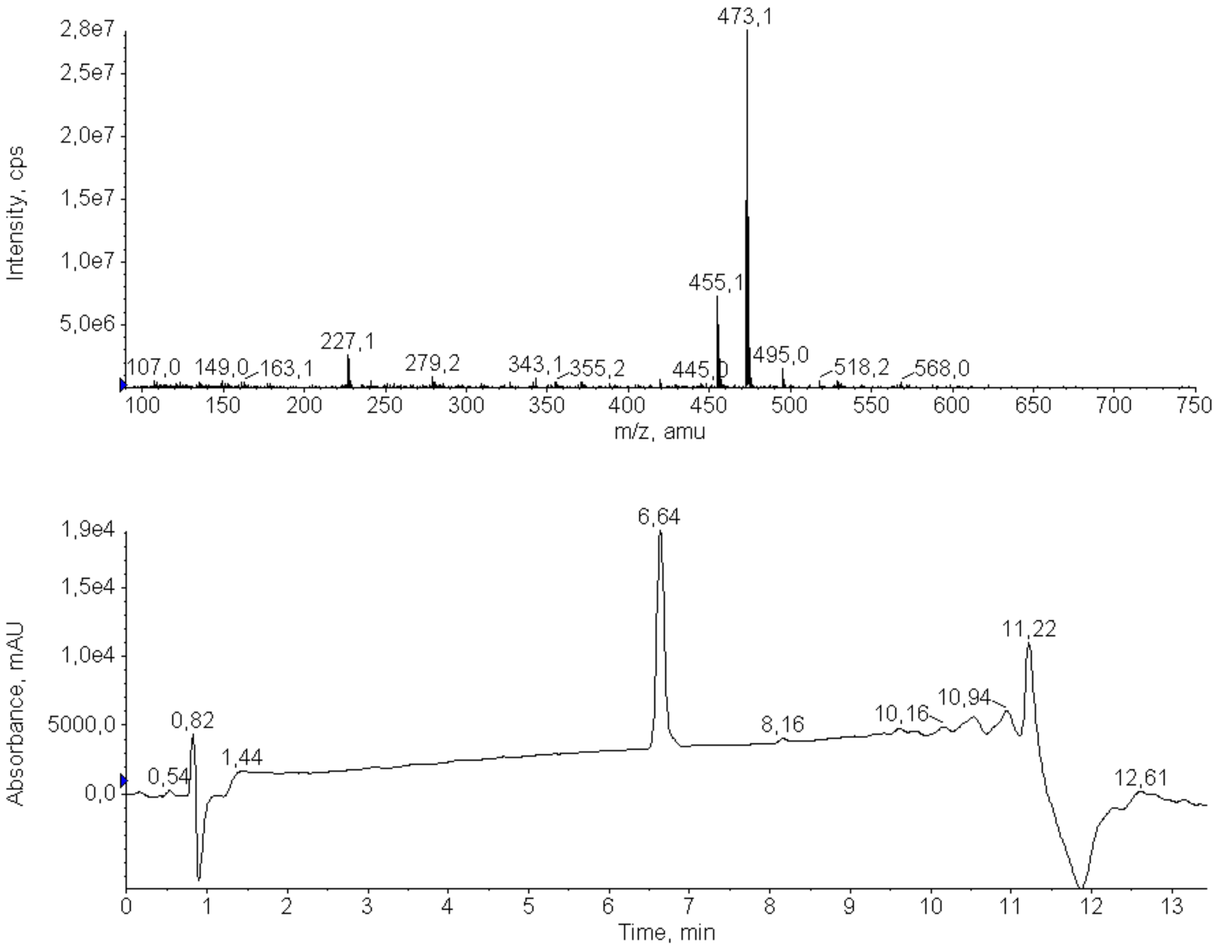

Supporting Information Figure 98: LC/MS analysis of **Scaff10-63**. Upper panel: Mass spectrum. Lower panel: according chromatogram.

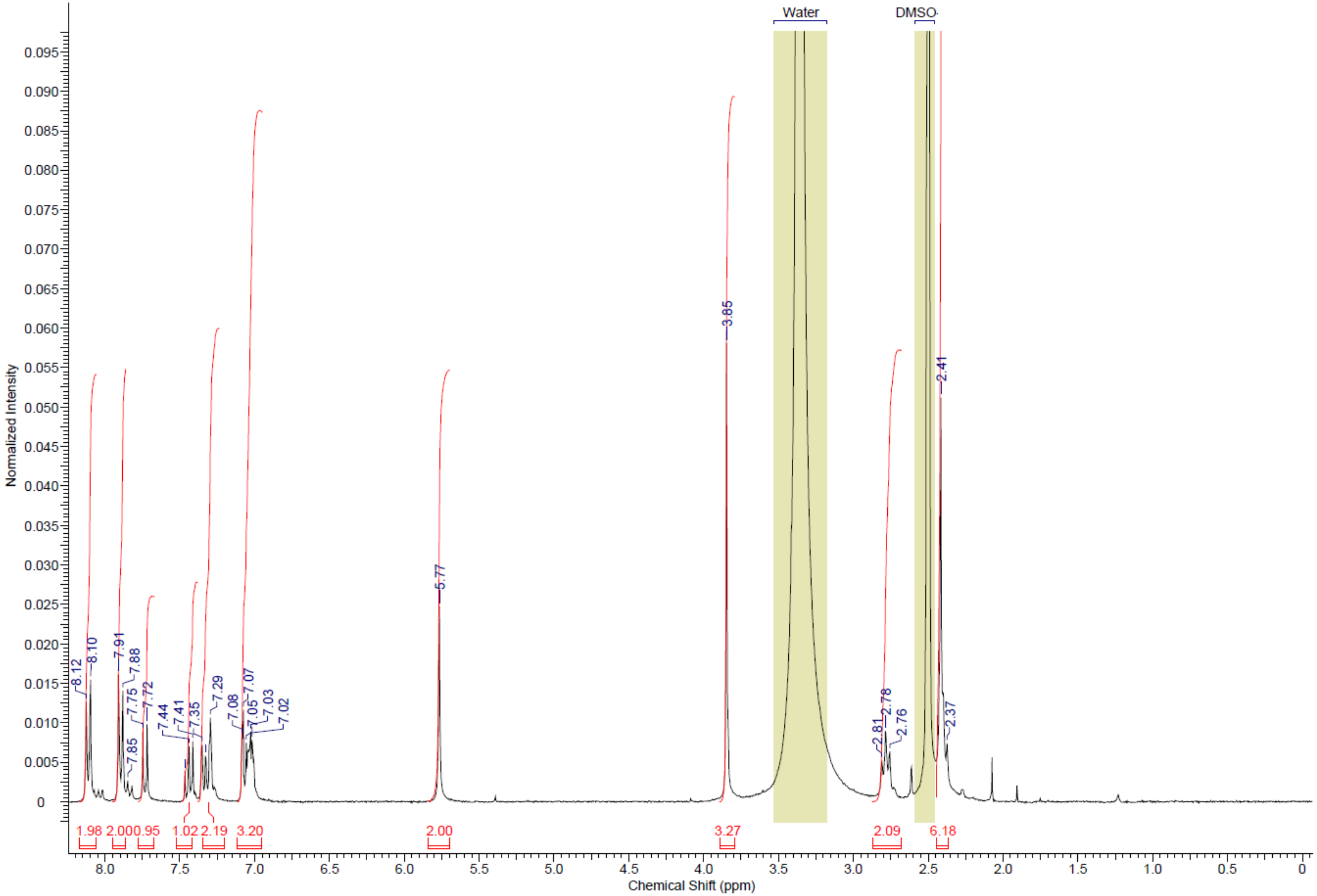

Supporting Information Figure 99: ^{1}H NMR spectrum of **Scaff10-63**, 300 MHz, DMSO-d_6.

4.1.54 3-[3-[4-(3-Methoxyphenyl)phenyl]-5-methyl-7-oxo-furo[3,2-*g*]chromen-6-yl]propanoic acid (**Scaff10-64**)

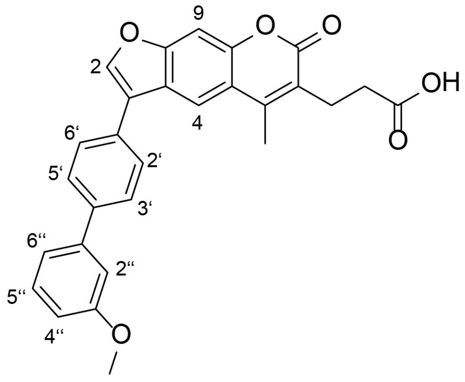

Ethyl 3-[7-[2-[4-(3-methoxyphenyl)phenyl]-2-oxo-ethoxy]-4-methyl-2-oxo-chromen-3-yl] propanoate (**Scaff10-62**) (115 mg, 0.23 mmol) was saponificated in a mixture of 1.5 ml DMF and 3 ml 3 M sodium hydroxide at 90 °C for 1 h. The product was purified using preparative HPLC with the following gradient: 25 % to 95 % ACN in H_2O in 80 min. The product eluted at t_R = 43 min and was obtained after lyophilisation as yellowish solid in 21 % yield (22 mg).

^{1}H NMR (300 MHz, DMSO-d$_6$): δ(ppm) = 2.34 – 2.47 (t, J = 7.32 Hz, 2H, -CH$_2$-C**H$_2$**-COOH), 2.54 – 2.65 (s, 3H, Ar-CH$_3$), 2.76 – 2.94 (t, J = 7.32 Hz, 2H, -C**H$_2$**-CH$_2$-COOH), 3.77 – 396 (s, 3H, -OCH$_3$), 6.93 – 7.01 (d, J = 7.32 Hz, 1H, H-4''), 7.23 – 7.36 (m, 2H, H-2'', -6''), 7.37 – 7.47 (m, 1H, H-5''), 7.77 – 7.81 (s, H1, H-9), 7.82 – 7.88 (d, J = 8.54 Hz, 2H, H-3', -5'), 7.88 – 7.96 (d, J = 8.54 Hz, 2H, H-2', -6'), 8.19 – 8.26 (s, 1H, H-4), 8.51 – 8.56 (s, 1H, H-2)

ESI-MS: cal. 455.14, exp. 455.1 ([M+H]$^+$)

HPLC: 99 % purity at 254 nm

t_R (HPLC): 7.54 min; λ_{max} = 251 nm and 302 nm

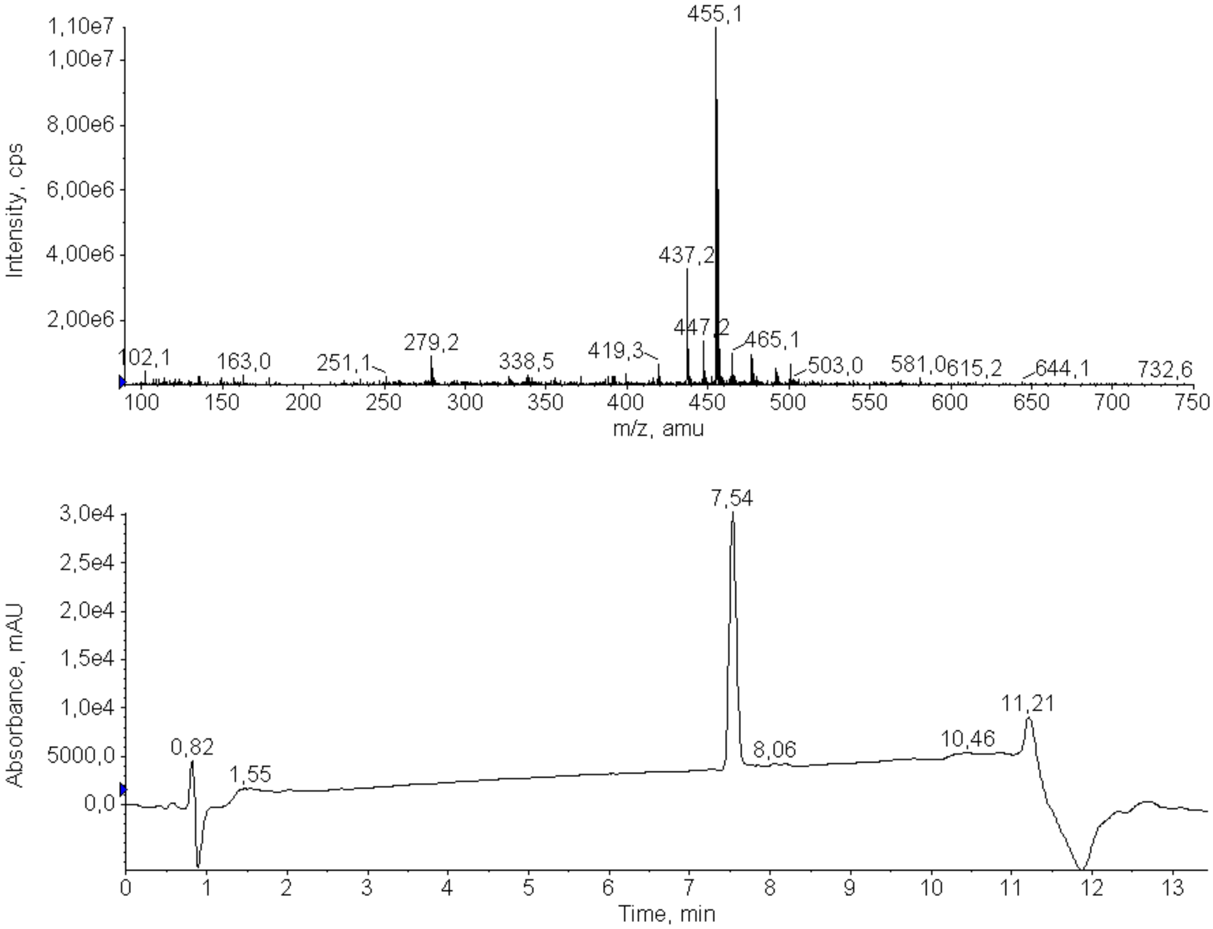

Supporting Information Figure 100: LC/MS analysis of **Scaff10-64**. Upper panel: Mass spectrum. Lower panel: according chromatogram.

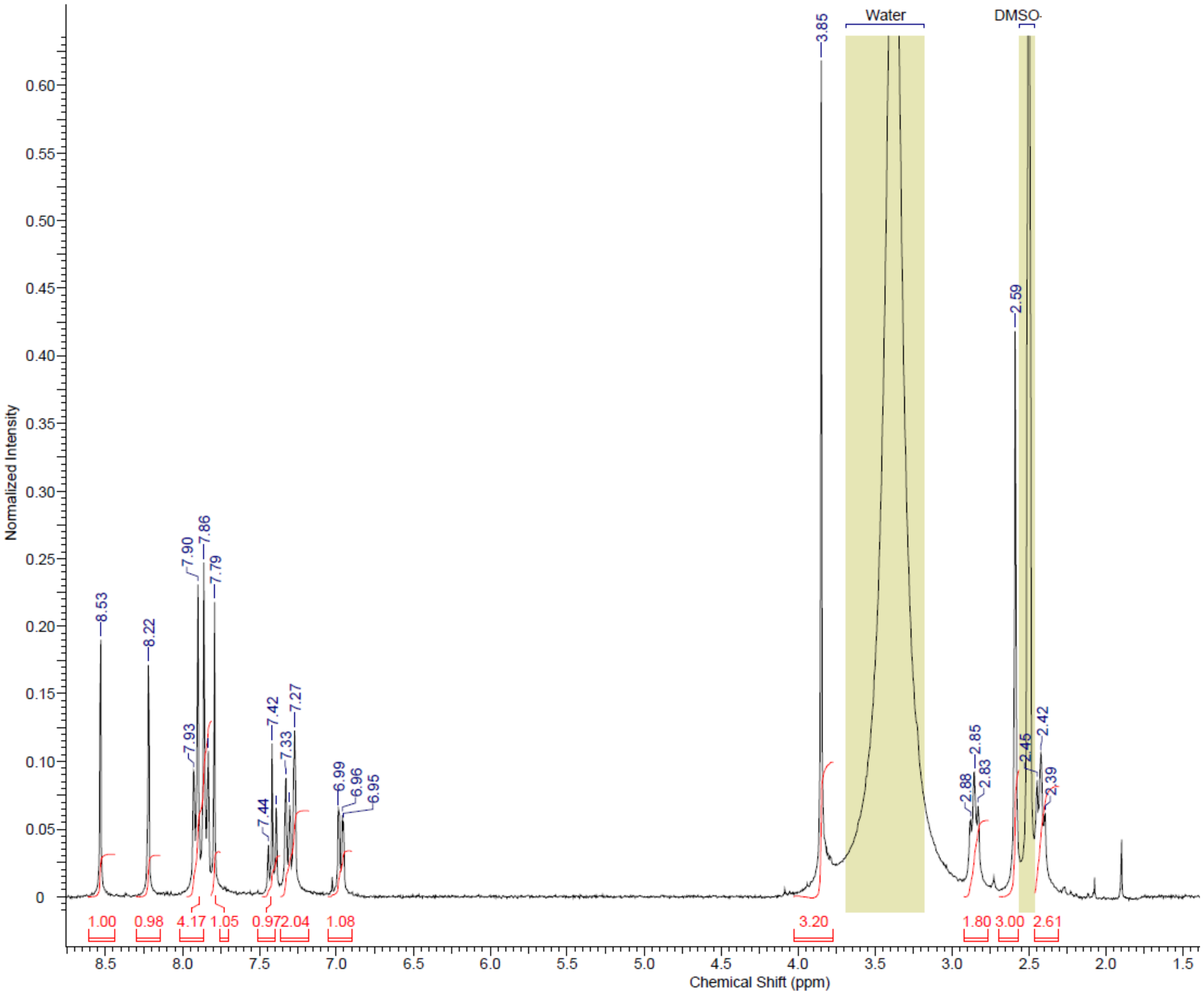

Supporting Information Figure 101: ^{1}H NMR spectrum of **Scaff10-64**, 300 MHz, DMSO-d$_6$.
